# A Pd-Catalyzed [4 + 2] Annulation Approach to Fluorinated N-Heterocycles

**DOI:** 10.1021/acs.orglett.1c00752

**Published:** 2021-03-24

**Authors:** Víctor García-Vázquez, Larry Hoteite, Christopher P. Lakeland, David W. Watson, Joseph P. A. Harrity

**Affiliations:** † Department of Chemistry, University of Sheffield, Sheffield, S3 7HF, United Kingdom; ‡ Medicinal Chemistry, Oncology R&D Research and Early Development, AstraZeneca Cambridge Science Park, Unit 310 Darwin Building, Cambridge, CB4 0WG, United Kingdom

## Abstract

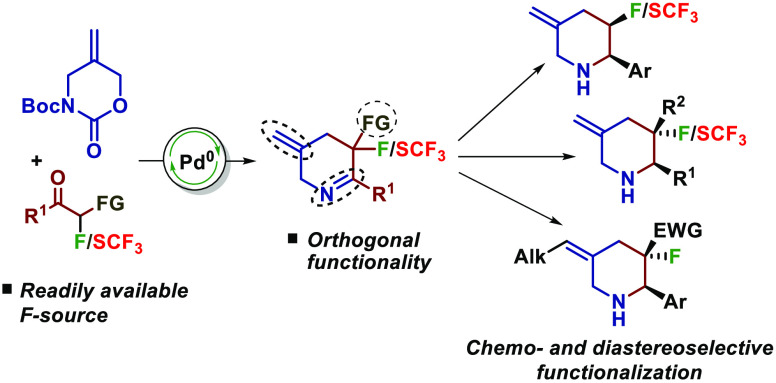

3-Fluoro- and trifluoromethylthio-piperidines represent important building blocks for discovery chemistry. We report a simple and efficient method to access analogs of these compounds that are armed with rich functionality allowing them to be chemoselectively derivatized with high diastereocontrol.

Fluorine-containing molecules exhibit a variety of useful properties in pharmaceuticals, agrochemicals, and materials science.^1−3^ In particular, the introduction of a C–F bond into a bioactive compound can have a dramatic impact on both the physical and chemical properties of the molecule.^4^ Additionally, nitrogen heterocycles are one of the most highly represented motifs within FDA approved small molecule drugs, with the piperidine ring as the most prevalent example of this class.^5^ Among other modifications of this cyclic amine, the selective incorporation of a fluorine atom at the 3-position of the piperidine scaffold has been demonstrated to be an effective strategy to improve the pharmacological properties of a number of biologically active compounds targeting SYK,^6^ CGRP,^7^ and MET kinase^8^ (Figure 1). In these cases, the fluorine atom plays a key role in preventing metabolism, as well as modulating the basicity of the nitrogen atom. 3-Fluoropiperidines have also been investigated as radiotracers for NR2B NMDA receptor visualization.^9^

**Figure 1:**
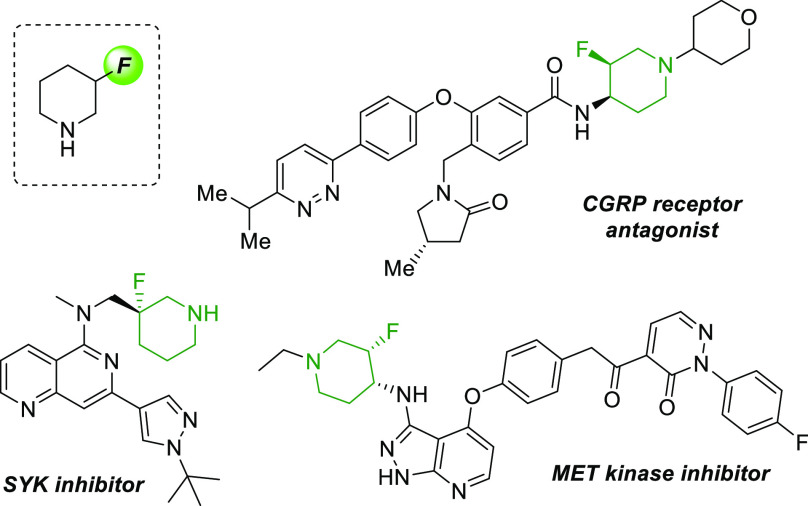
Prominent bioactive 3-fluoropiperidines.

In spite of the importance of 3-fluoropiperidine derivatives, only a few general strategies exist to access these compounds. The electrophilic fluorination of piperidone derived enol equivalents has been reported,^10^ but this method faces regioselectivity issues when applied to nonsymmetrical scaffolds. The deoxofluorination of alkoxypiperidines has also been reported, but these reactions require extensive prefunctionalization and exhibit poor atom economy.^11^ A particularly prevalent strategy relies on the intramolecular aminofluorination of olefins using Pd-catalysis^12^ or hypervalent iodine reagents.^13^ However, these methods commonly employ strong oxidizing agents or rely on the use of stoichiometric quantities of toxic^13b^ or expensive reagents.^13c^ A recent report on the direct hydrogenation of fluorinated pyridines^14^ provides a diastereoselective synthetic pathway for the synthesis of fluoropiperidines, but the high H_2_ pressure required reduces operational simplicity. We envisioned that our recently reported [4 + 2] annulation strategy to N-heterocycles^15^ could offer a powerful route to 3-fluoropiperidines using readily available α-fluoro-β-ketoester starting materials.^16^ Advantages of this method would include its highly modular nature, allowing for the rapid construction of the piperidine core. Moreover, the heterocycle products contain orthogonal functionality that would allow their elaboration to new products through multiple vectors (Scheme 1).

**Scheme 1:**
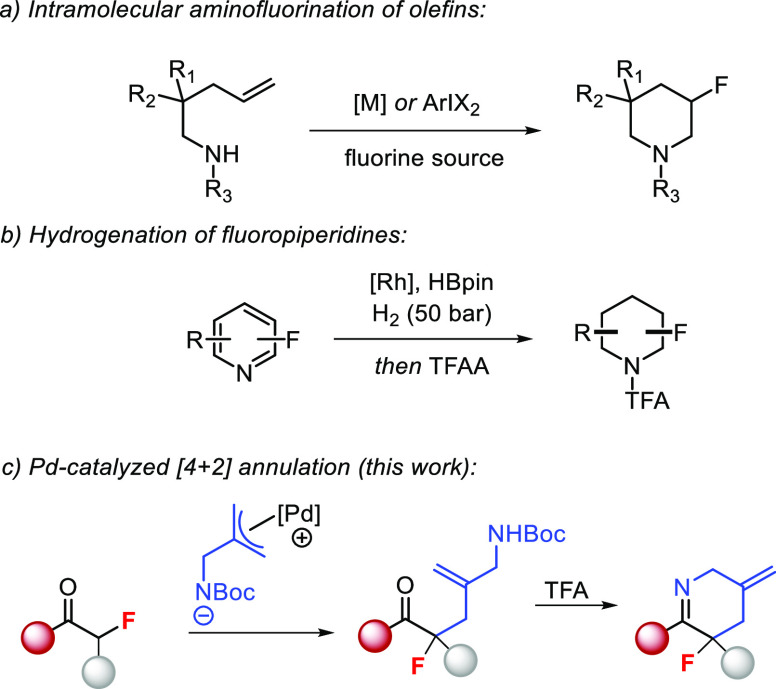
Synthetic Routes to 3-Fluoropiperidines

We began our studies by investigating the allylation/condensation reaction of readily available α-fluoro-β-ketoester **1a** and cyclic carbamate **2** as shown in Scheme 2. Subjecting this substrate to 5 mol % of Pd(dba)_2_ and 15 mol % of ligand **L1** followed by treatment of the intermediate with TFA led smoothly to the desired 3-fluoropiperidine **4a** in high yield (Scheme 2a). Moreover, this product was also obtained in comparable yield employing a one-pot procedure without isolation of intermediate **3**. To our delight, scaling up the reaction to multigram quantities yielded **4a** with similar results. We then examined the applicability of this methodology to a number of α-fluoro-β-ketoesters. Aryl substituted imines with either electron-withdrawing or electron-donating groups at the *para*-position on the aryl ring gave excellent yields (**4b**–**f**). Substitution at other points on the aryl ring such as *ortho*-methyl (**4g**) and naphthyl (**4h**) are also well tolerated. The heterocyclic thiophenyl containing substrate **1i** was also converted into the corresponding piperidine imine **4i** in 78% yield. In addition to α-fluoro-β-ketoesters, numerous other fluorinated nucleophiles could be employed in the allylation/condensation sequence including α-fluoro-β-ketonitriles (**4j**), α-fluoro-β-ketosulfones (**4k**), and α-fluoro-β-ketoamides (**4l**). Unfortunately, however, α-fluoroketones bearing alkyl groups were not transformed to the corresponding heterocycles, and a complex mixture of products was instead produced. Furthermore, this sequence could also be applied to alkyl substituted α-fluoro-β-ketoesters in a regioselective manner (Scheme 2b). Simple alkyl groups containing various levels of substitution at the α-position (Me, 1°, 2°, and 3°) afforded the piperidine imines **4n**–**4r** in high yields. Finally, a derivative of l-proline was evaluated, with **4s** obtained in good yield and with moderate diastereoselectivity. The excellent functional group tolerance of this reaction sequence serves to highlight the mild nature of this procedure.

**Scheme 2:**
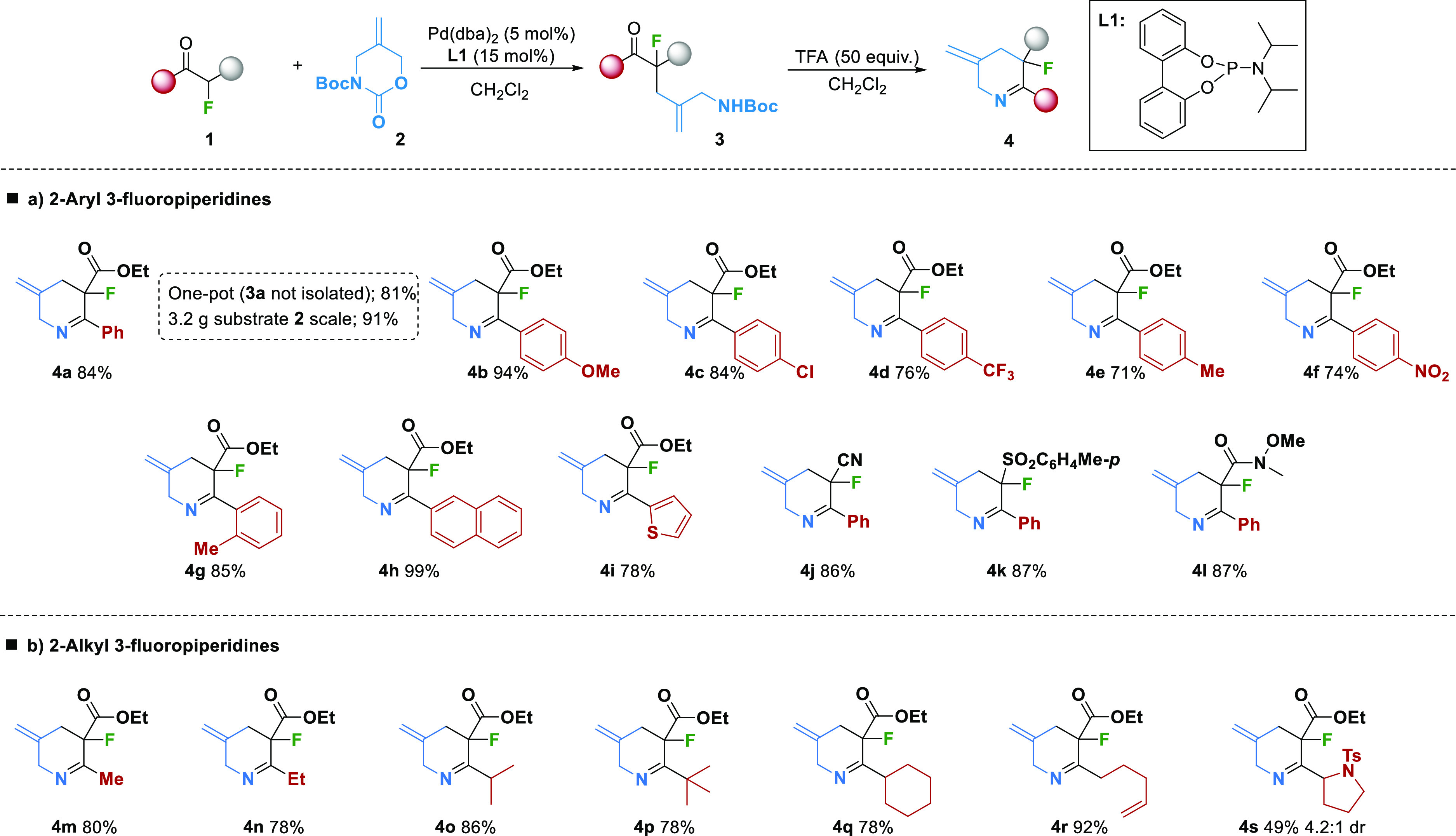
Reaction Scope Reaction conditions: **1** (0.3 mmol), **2** (0.2 mmol), Pd(dba)_2_ (10 μmol, 5 mol %), **L1** (30 μmol, 15 mol %), DCM (0.1 M), rt, 18 h under N_2_.

We next turned our attention to demonstrating that the functionalized 3-fluoropiperidines were versatile intermediates for organic chemistry (Scheme 3). First, a chemoselective reduction of **4a** using NaBH(OAc)_3_ in acetic acid solvent produced saturated piperidine **5** with high diastereoselectivity. Subsequent protection of **5** using di-*tert*-butyl dicarbonate then gave compound **6** in 88% yield, with the X-ray structure of **6** (CCDC 2063492) providing the relative configuration of the major diastereoisomer obtained in this process. Interestingly, a chemoselective reduction of the ester moiety was also achieved using LiAlH_4_ to give **7** in moderate yield. A hydrolytic decarboxylation using aq. HCl and heating afforded fluoropiperidine **8**, which could then be reduced using NaBH_4_ to provide saturated piperidine **9** in 77% yield as a single diastereoisomer following column chromatography.^17^ Notably, this decarboxylation circumvents the limitation associated with the poor reactivity of α-fluoroketones in the allylation/condensation cascade. Selective functionalization of the exocyclic alkene is also possible; cross-metathesis produced **10** as a mixture of geometric isomers. The ability to selectively functionalize each functional handle in piperidine imines **4** demonstrates their utility as synthetic intermediates.

**Scheme 3:**
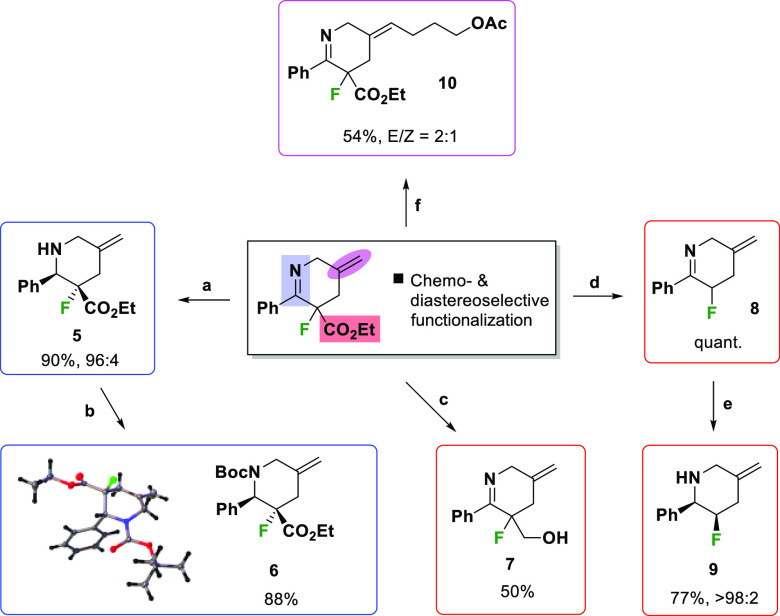
Chemoselective Functionalization of 3-Fluoropiperidine Imines Reagents and conditions: (a) NaBH(OAc)_3_ (1.5 equiv), AcOH, rt, 18 h (90%); (b) Boc_2_O (2.0 equiv), Et_3_N (2.0 equiv), THF, rt, 18 h (88%); (c) LiAlH_4_ (2.0 equiv), THF, rt, 3.5 h (50%); (d) aq. HCl (15 equiv), 100 °C, 1 h (quant.); (e) NaBH_4_ (2.0 equiv), MeOH, 0 °C to rt, 18 h (77%); (f) Hoveyda–Grubbs second Gen. (5 mol %), pent-4-en-1-yl acetate (3 equiv), DCM, 25 °C 18 h then reflux, 3 h (54%).

The suitability of our method for accessing useful fluorinated heterocycles suggested that it might be adapted to allow the incorporation of trifluoromethylthio (SCF_3_) groups. In this regard, and to the best of our knowledge, only two examples of 3-SCF_3_-substituted piperidines have been reported.^18^ Due to its electron-withdrawing nature and high lipophilicity, the SCF_3_ moiety can significantly modulate the pharmacological properties of bioactive compounds.^19^ Nevertheless, the availability of synthetic methods that deliver saturated *N*-trifluoromethyl-thiolated six-membered heterocycles is scarce, and those that are documented suffer from limited substrate scope.^20^

Our efforts to employ the [4 + 2] annelation sequence to α-SCF_3_-ketones is summarized in Scheme 4. Aryl substituted ketones proved to be excellent substrates for this transformation, generating a range of 3-SCF_3_-substituted piperidines under mild conditions. Unfortunately, these products proved to be unstable to chromatography, and so we used a borohydride reduction step prior to isolation. Accordingly, 2-aryl 3-trifluoromethylthio-piperidines **13a**–**g** were isolated in excellent yields over three steps, and with very high *cis*-stereocontrol. X-ray crystal structure analysis of aryl substituted products **13a** (CCDC 2063487) and **13e** (CCDC 2063489) confirmed the relative stereochemistry of the major diastereomer in these cases, and the stereochemistry of all other aryl-substituted products was assigned by inference. Unfortunately, however, 2-aryl-substituted ketones containing electron-withdrawing groups (4-nitrophenyl and 4-trifluoromethylphenyl) were found to decompose during the TFA-mediated deprotection–condensation step. Finally, α-SCF_3_-propiophenone led to a more substituted analog **13h**, while the potential to access 2-alkyl piperidine products was confirmed in one case, albeit in low yield.

**Scheme 4:**
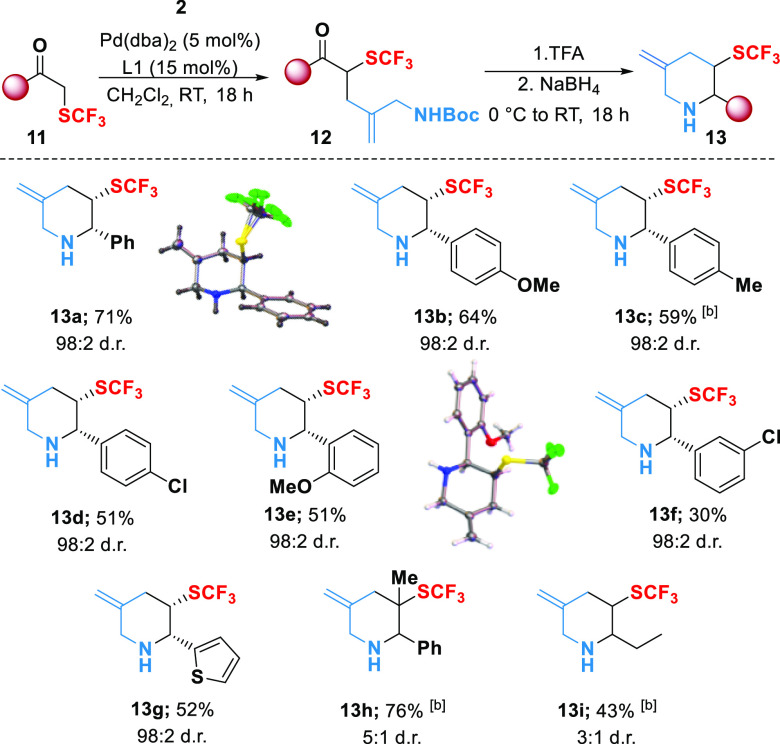
Synthesis of 3-SCF_3_-Substituted Piperidines Reagents and conditions: **11** (0.7 mmol), **2** (0.47 mmol), Pd(dba)_2_ (23 μmol, 5 mol %), **L1** (70 μmol, 15 mol %), CH_2_Cl_2_ (0.1 M), RT, 18 h under N_2_. Heated at 40 °C.

In conclusion, we report that 3-fluoropiperidines bearing orthogonal imine, ester, and alkene functionality can be readily prepared and chemoselectively derivatized, providing a powerful approach to these important substructures. Moreover, this method can be extended to provide the first general means to incorporate the 3-trifluoromethylthio-group into piperidines, offering a new and efficient entry into these important scaffolds.

## Supporting Information Available

Details of experimental procedures and spectroscopic data. NMR spectral data included (PDF)

## Accession Codes

CCDC 2063487, 2063489, and 2063492 contain the supplementary crystallographic data for this paper. These data can be obtained free of charge via www.ccdc.cam.ac.uk/data_request/cif, or by emailing data_request@ccdc.cam.ac.uk, or by contacting The Cambridge Crystallographic Data Centre, 12 Union Road, Cambridge CB2 1EZ, UK; fax: +44 1223 336033.
