# Supplementary material for: A Pd-Catalyzed [4 + 2] Annulation Approach to Fluorinated N-Heterocycles
Source: Org Lett. 2021 Mar 24;23(7):2811–5. doi: 10.1021/acs.orglett.1c00752 (PMC8041373; doi:10.1021/acs.orglett.1c00752)
Supplement: Supplementary file 1 — ol1c00752_si_001.pdf [file ol1c00752_si_001.pdf]

Supporting Information

A Pd-Catalyzed [4 + 2] Annulation Approach to Fluorinated N-Heterocycles

Víctor García-Vázquez,†[a] Larry Hoteite,†[a] Christopher P. Lakeland,†[a] David W. Watson[b] and Joseph P. A. Harrity*[a]

[a] Department of Chemistry, University of Sheffield, Sheffield, S3 7HF, United Kingdom

[b] Medicinal Chemistry, Oncology R&D Research and Early Development, AstraZeneca Cambridge Science Park, Unit 310 Darwin Building, Cambridge, CB4 0WG, United Kingdom

† These authors contributed equally

** Email: j.harrity@sheffield.ac.uk*

Table of Contents

General Considerations........ S2

General Procedures S3

Scale Up Experiment........ S4

Experimental Procedures........ S5

  Substrates and Ligands S5

  Allylation Products S12

  Condensation Products........ S24

NMR Spectra S36

Stereochemical Assignment of 9........ S198

Crystallographic Data........ S200

References........ S206

General Considerations

All reactions were carried out in flame-dried glassware equipped with a magnetic stir bar under nitrogen atmosphere, unless stated otherwise. Solvents were purified using a PureSolv MD purification system and transferred under nitrogen. A DrySyn block combined with a temperature probe was used as the heating source, where required. Infrared (IR) spectra were recorded on a Perkin Elmer Paragon FTIR spectrometer (ν_{max} in cm^{-1}). Samples were recorded neat as thin films. ^{1}H-NMR spectra were recorded on a Bruker AVIII HD 400 (400 MHz), Bruker AVI 400 (400 MHz) or Bruker AMX400 (400 MHz). Chemical shifts are reported in parts per million (ppm) from tetramethylsilane, using the residual protic solvent resonance as the internal reference: ($CHCl_3$: δ 7.26) unless otherwise stated. Data are reported as follows: chemical shift (integration, multiplicity (s = singlet, d = doublet, t = triplet, q = quartet, br = broad, m = multiplet), coupling constant (Hz)). ^{13}C-NMR spectra were recorded on a Bruker AVIII HD 400 (101 MHz), Bruker AVI 400 (101 MHz) or Bruker AMX-400 (101 MHz) with broadband proton decoupling. Chemical shifts are reported in ppm from trimethylsilane with the solvent as the internal reference ($CDCl_3$: δ 77.16). ^{19}F-NMR spectra were recorded on a Bruker AV III HD 400 (377 MHz) and are uncorrected. High resolution mass spectra (HRMS) recorded for accurate mass analysis, were performed on either a Micromass LCT operating in electrospray mode (TOF, ES^+) or a Micromass Prospec operating in FAB (FAB^+), EI (EI^+) or CI (CI^+) mode. Thin layer chromatography (TLC) was performed on aluminium-backed plates pre coated with silica (0.2 mm, Merck 60 F_{254}) which were developed using standard visualizing agents: UV light or potassium permanganate. Flash chromatography was performed on silica gel (Merck 40-63 µm). Melting points were recorded on Gallenkamp melting point apparatus and are uncorrected.

General Procedures

General Procedure A (GPA)

To a suspension of sodium hydride (60% dispersion in mineral oil, 2.5 equiv.) in THF (0.5 M) under nitrogen was added ketone (1 equiv.) and diethyl carbonate (2.8 equiv.) and the resulting suspension heated to reflux until complete consumption of the ketone substrate. The reaction was then cooled to room temperature, diluted with AcOH (0.1 mL per mmol) and water (4 mL per mmol) and extracted with DCM (3 x 4 mL per mmol). The combined organic layers were then dried over anhydrous magnesium sulfate and concentrated under vacuum. The crude oil was then dissolved in acetonitrile (0.5 M) under nitrogen and selectfluor (1.2 equiv.) was added and the resulting mixture heated to 50 °C overnight. After cooling to room temperature the reaction was diluted with water (4 mL per mmol) and extracted with ethyl acetate (3 x 4 mL per mmol). The combined organic layers were then dried over anhydrous magnesium sulfate and concentrated under vacuum. Purification by flash silica column chromatography (FCC) afforded the target fluorinated ketoesters.

General Procedure B (GPB)

To a solution of ethyl fluoroacetate (1 equiv.) and diphenylphosphinic chloride (1 equiv.) in dry THF (0.125 M) at -78 °C was added dropwise a solution of lithium bis(trimethylsilyl)amide (1.0 M in hexanes, 2 equiv.). After 10 minutes, the corresponding acyl chloride (1.1 equiv.) was added dropwise. The mixture was then stirred overnight at RT. After completion, the reaction was diluted with a saturated solution of ammonium chloride (4 mL per mmol) and extracted with ethyl acetate (3x 4 mL per mmol). The combined organic layers were then dried over anhydrous magnesium sulfate and concentrated under vacuum. Purification by flash silica column chromatography (FCC) afforded the target fluorinated ketoesters.

General Procedure C (GPC)

To a solution of the corresponding β-ketoester (1 equiv.) in MeCN (0.5 M) under nitrogen was added selectfluor (1.2 equiv.) and the resulting mixture heated to 50 °C overnight. After cooling to room temperature the reaction was diluted with water (4 mL per mmol) and extracted with ethyl acetate (3 x 4 mL per mmol). The combined organic layers were then dried over anhydrous magnesium sulfate and concentrated under vacuum. Purification by flash silica column chromatography (FCC) afforded the target fluorinated ketoesters.

General Procedure D (GPD)

Solution 1: To a solution of carboxylic acid (1.1 equiv.) in DCM (0.33 M) under nitrogen at 0 °C was added $(COCl)_2$ (1.1 equiv.) and 1 drop of DMF and the solution stirred at room temperature for 1.5 hours. The solvent was then removed under vacuum and the residue dissolved in THF (0.36 M) under nitrogen.

Solution 2: To a solution of ethyl fluoroacetate (1 equiv.) and diphenylphosphinic chloride (1 equiv.) in dry THF (0.2 M) at -78 °C was added dropwise a solution of lithium bis(trimethylsilyl)amide (1.0 M in hexanes, 3 equiv.). After 10 minutes **solution 1** was added dropwise and the reaction mixture was then allowed to warm to room temperature and stirred overnight. The mixture was then diluted with sat. NH4Cl (25 mL), extracted with EtOAc (4 x 25 mL) and the combined organic layers dried over anhydrous magnesium sulfate and concentrated under vacuum. Purification by flash silica column chromatography (FCC) afforded the target fluorinated ketoesters.

General Procedure E (GPE)

A flame-dried sealed tube was charged with $Pd(dba)_2$ (5 mol%), *N*,*N*-diisopropyldibenzo[*d*,*f*][1,3,2]dioxaphosphepin-6-amine (15 mol%) and *tert*-butyl 5-methylene-2-oxo-1,3-oxazinane-3-carboxylate (1 equiv.) under nitrogen. Anhydrous CH_2Cl_2 (5 mL per mmol) was then added and the mixture stirred at room temperature for 10 minutes. A solution of ketoester (1.5 equiv.) in CH_2Cl_2 (5 mL per mmol) was then added and the reaction stirred at room temperature overnight. The reaction was then concentrated under vacuum and purified by flash silica column chromatography.

General Procedure F (GPF)

To a solution of allyation product (1 eq) in DCM (0.1M) was added TFA (75 equiv.) and the resulting mixture stirred at room temperature for 1.5 hours. The mixture was then basified to pH 8 using sat. $NaHCO_3$ and stirred for 45 minutes before extraction with DCM (3 x 25 mL). The combined organic layers were then dried over anhydrous magnesium sulfate and concentrated under vacuum to afford the title piperidines.

SCF_3-substituted starting materials **11a** - **11i** were synthesised in a single step from commercially available α-haloketones following reported procedure.[1]

General Procedure G (GPG)

To a solution of the allylated product (1 eq.) in CH_2Cl_2 (0.1 M) was added TFA (75 equiv.) and the resulting mixture was stirred at room temperature for 1 hour. The reaction mixture was concentrated under reduced pressure and residual TFA was removed by azeotropic distillation with toluene (3 x 1 mL). The iminium salt was dissolved in MeOH (0.2 M) and cooled to 0 °C. $NaBH_4$ (4 eq.) was added and the reaction was then allowed to warm to room temperature and stirred for 16 h under nitrogen. The reaction mixture was diluted with a saturated solution of $NaHCO_3$ (15 mL) and extracted with EtOAc (4 x 15 mL). The combined organic layers were dried over anhydrous $MgSO_4$, filtered and concentrated under reduced pressure. The residue was then subjected to silica gel FCC (20% EtOAc in 40-60 petroleum ether) to give the corresponding 3-SCF_3-piperidine.

Scale Up Experiment

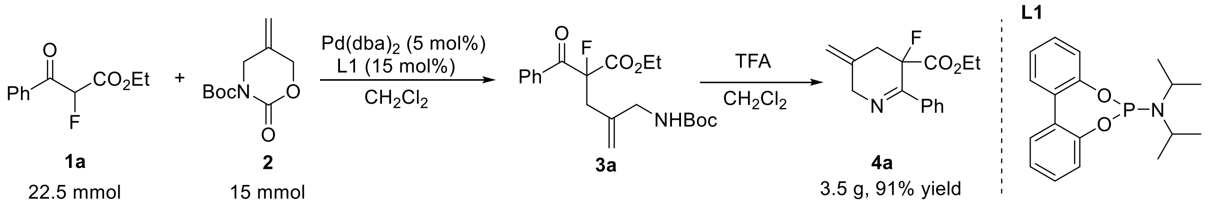

A flame-dried 500 mL round bottom flask was charged with $Pd(dba)_2$ (431 mg, 5 mol%), *N*,*N*-diisopropyldibenzo[*d*,*f*][1,3,2]dioxaphosphepin-6-amine (708 mg, 15 mol%) and *tert*-butyl 5-methylene-2-oxo-1,3-oxazinane-3-carboxylate (3.2 g, 15 mmol) under nitrogen. Anhydrous CH_2Cl_2 (75 mL) was then added and the mixture stirred at room temperature for 10 minutes. A solution of ethyl 2-fluoro-3-oxo-3-phenylpropanoate (**1a**) (4.72 g, 22.5 mmol) in CH_2Cl_2 (75 mL) was then added and the reaction stirred at room temperature overnight. The reaction was then concentrated under vacuum and purified by flash silica column chromatography (20 % ethyl acetate in petroleum ether). The collected intermediate was then dissolved in CH_2Cl_2 (150 mL) and TFA (86 mL, 75 equiv.) was added to the mixture. After stirring at room temperature for 1.5 hours, the mixture was basified to pH 8 using sat. $NaHCO_3$ and stirred for 45 more minutes before extraction with CH_2Cl_2 (3x 150 mL).

The combined organic layers were then dried over anhydrous magnesium sulfate and concentrated under vacuum to afford ethyl 3-fluoro-5-methylene-2-(phenyl) 3,4,5,6-tetrahydropyridine-3-carboxylate (**4a**) as a yellow oil (3.5 g, 91% yield over two steps). The product showed satisfactory spectroscopic data (vide infra).

Experimental Procedures

Substrates and Ligands

2-(iodomethyl)prop-2-en-1-ol (s1)[2]

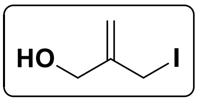

To a solution of 2-methylene-1,3-propanediol (22.0 g, 250 mmol), triphenylphosphine (72.1 g, 275 mmol) and imidazole (18.7 g, 275 mmol) in a mixture of DCM (277 mL) and EtOAc (277 mL) at 0 °C under nitrogen was added iodine (63.4 g, 250 mmol) portionwise and the resulting mixture stirred at room temperature in the dark for 24 hours. The solvent was then removed under vacuum (**CAUTION**: alkylating agents) and the residue purified by FCC (gradient from 15-20% EtOAc in 40-60 petroleum ether) to afford 2-(iodomethyl)prop-2-en-1-ol (**s1**) as a yellow oil (28.2 g, 57%).

^{1}H NMR (400 MHz, $CDCl_3$) δ 5.38 (dd, J= 1.5, 1.0 Hz, 1H), 5.23 (dd, J= 2.5, 1.5 Hz, 1H), 4.34 (d, J= 5.5 Hz, 2H), 4.00 (d, J= 1.0 Hz, 2H), 2.14 (t, J= 5.5 Hz, 1H); **^{13}C NMR (101 MHz, $CDCl_3$)** δ 145.8, 114.2, 63.9, 5.8.

tert-butyl 5-methylene-2-oxo-1,3-oxazinane-3-carboxylate (2)[2]

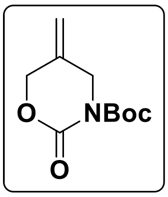

To a suspension of silver cyanate (32.0 g, 214 mmol) in toluene (375 mL) under nitrogen was added 2-(iodomethyl)prop-2-en-1-ol (**s1**) (28.2 g, 142 mmol) in toluene (30 mL) and the resulting mixture heated at reflux for 24 hours. After cooling to room temperature the reaction was filtered through celite, the filter pad washed with Et_2O (3 x 100 mL) and the combined filtrates concentrated under vacuum. The crude solid and 4-dimethylaminopyridine (3.5 g, 28 mmol) were then dissolved in DCM (215 mL) under nitrogen and the resulting mixture cooled to 0 °C. Di-*tert*-butyl dicarbonate (62.0 g, 284 mmol) was then added slowly and the resulting mixture heated at 25 °C for 24 hours. Removal of the solvent under vacuum and purification by FCC (gradient from 20-25% EtOAc in 40-60 petroleum ether) afforded *tert*-butyl 5-methylene-2-oxo-1,3-oxazinane-3-carboxylate (**2**) as a colourless oil which converted to a white solid upon standing in the freezer (17.6 g, 58%).

^{1}H NMR (400 MHz, $CDCl_3$) δ 5.18 (d, J = 6.0 Hz, 2H), 4.64 (s, 2H), 4.29 (t, J= 2.0 Hz, 2H), 1.52 (s, 9H); **^{13}C NMR (100 MHz, $CDCl_3$)** δ152.0, 150.9, 134.7, 113.0, 83.9, 69.9, 48.7, 28.0.

N,*N*-diisopropyldibenzo[d,f][1,3,2]dioxaphosphepin-6-amine (L1)[2]

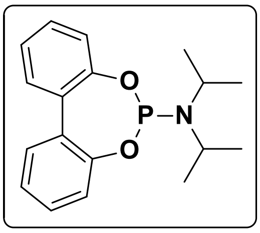

To a flame-dried RBF containing THF (168 mL) at 0 °C under nitrogen was added PCl_3 (3.7 g, 27 mmol) dropwise over 15 minutes. Et_3N (14 g, 134 mmol) was then added dropwise over 15 minutes followed by the addition of diisopropylamine (2.7 g, 27 mmol) dropwise over 30 minutes. The resulting mixture was then warmed to room temperature and stirred for 3 hours before cooling to 0 °C. 2,2′-Biphenol (5.0 g, 27 mmol) was then added portionwise and the reaction mixture stirred at room temperature

overnight. The volatiles were then removed under vacuum (**CAUTION**: toxic) and the resulting residue purified by FCC (gradient from 0-20% DCM in 40-60 petroleum ether) to afford *N*,*N*-diisopropyldibenzo[d,f][1,3,2]dioxaphosphepin-6-amine (**L1**) as a white solid (7.4 g, 87%).

^{1}H NMR (400 MHz, CDCl$_3$) δ 7.46 (dd, J= 7.5, 1.5 Hz, 2H), 7.33 (td, J= 7.5, 1.5 Hz, 2H), 7.24 –7.16 (m, 4H), 3.58 –3.44 (dhept., 10.5 Hz, J= 7.0 Hz, 2H), 1.22 (d, J= 7.0 Hz, 12H); **^{13}C NMR (101 MHz, CDCl$_3$)** δ 151.9 (d, *J* = 5.5 Hz), 131.0 (d, *J* = 3.0 Hz), 129.8, 129.1, 124.3, 122.3, 44.7 (d, *J* = 12.5 Hz), 24.6 (d, *J* = 8.0 Hz); **^{31}P NMR (162 MHz, CDCl$_3$)** δ 152.2 – 151.8 (m).

pent-4-en-1-yl acetate (s2)[3]

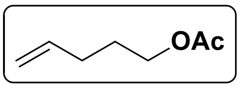

To a solution of 4-penten-1-ol (0.86 g, 10 mmol) and 4-dimethylaminopyridine (0.24 g, 2.0 mmol) in pyridine (20 mL) under nitrogen was added acetyl chloride (1.6 g, 20 mmol) and the resulting mixture heated at 40 °C for 4 hours. After cooling to room temperature the mixture was diluted with H_2O (10 mL) and sat. $NaHCO_3$ (10 mL) and extracted with Et_2O (4 x 25 mL). The combined organic layers were then washed with 5% $CuSO_4$ (8 x 10 mL) and 1.0 M HCl (3 x 25 mL) and dried over anhydrous $MgSO_4$. Removal of the volatiles under vacuum afforded pent-4-en-1-yl acetate (**s2**) (1.1 g, 84%)(CAUTION: stench).

^{1}H NMR (400 MHz, CDCl$_3$) δ 5.79 (ddt, *J* = 17.0, 10.0, 6.5 Hz, 1H), 5.06 – 4.94 (m, 2H), 4.06 (t, *J* = 6.5 Hz, 2H), 2.11 (dd, *J* = 14.5, 7.5 Hz, 2H), 2.03 (s, 3H), 1.76 – 1.67 (m, 2H); **^{13}C NMR (101 MHz, CDCl$_3$)** δ 171.3, 137.6, 115.4, 64.0, 30.1, 27.9, 21.1.

ethyl 2-fluoro-3-oxo-3-phenylpropanoate (1a)[4]

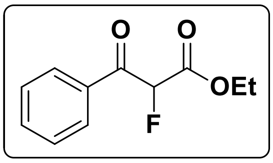

Following GPA using acetophenone (2.50 g, 21 mmol), diethylcarbonate (6.8 g, 58 mmol), sodium hydride (60% dispersion in mineral oil, 2.1 g, 52 mmol), selectfluor (8.8 g, 25 mmol) and acetic acid (2.5 mL) with FCC (10% EtOAc in 40-60 petroleum ether) afforded ethyl 2-fluoro-3-oxo-3-phenylpropanoate (**1a**) as a yellow oil (3.6 g, 82%).

^{1}H NMR (400 MHz, CDCl$_3$) δ 8.05 – 8.00 (m, 2H), 7.66 – 7.59 (m, 1H), 7.52 – 7.46 (m, 2H), 5.87 (d, *J* = 49.0 Hz, 1H), 4.34 – 4.22 (m, 2H), 1.24 (t, *J* = 7.0 Hz, 3H); **^{13}C NMR (101 MHz, CDCl$_3$)** δ 189.6 (d, $^{2}J_{C-F}$ = 20.0 Hz), 165.0 (d, $^{2}J_{C-F}$ = 24.0 Hz), 134.63, 133.5 (d, $^{3}J_{C-F}$ =2.0 Hz), 129.6 (d, $^{4}J_{C-F}$ =3.5 Hz), 128.9, 90.1 (d, $^{1}J_{C-F}$ =197.5 Hz), 62.8, 14.0; **^{19}F NMR (377 MHz, CDCl$_3$)** δ -190.4 (d, *J* = 49.0 Hz); **FTIR:** ν_{max}/cm^{-1} (neat) 2984, 1758, 1692, 1597, 1449, 1241, 1096, 1014 cm^{-1}.

ethyl 2-fluoro-3-(4-methoxyphenyl)-3-oxopropanoate (1b)[5]

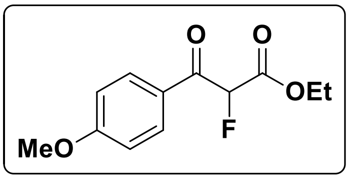

Following GPA using 4-methoxyacetophenone (1.5 g, 10 mmol), diethylcarbonate (3.3 g, 28 mmol), sodium hydride (60% dispersion in mineral oil, 1.0 g, 25 mmol), selectfluor (4.3 g, 12 mmol) and acetic acid (1 mL) with FCC (gradient from 0-10% EtOAc in 40-60 petroleum ether) afforded ethyl 2-fluoro-3-(4-methoxyphenyl)-3-oxopropanoate (**1b**) as an orange oil (1.8 g, 73%).

^{1}H NMR (400 MHz, CDCl$_3$) δ 8.05 – 8.00 (m, 2H), 6.97 – 6.93 (m, 2H), 5.81 (d, *J* = 49.0 Hz, 1H), 4.34 – 4.21 (m, 2H), 3.87 (s, 3H), 1.25 (t, *J* = 7.0 Hz, 3H); **^{13}C NMR (101 MHz, CDCl$_3$)** δ 187.9 (d, $^{2}J_{C-F}$ = 20.0 Hz), 165.3 (d, $^{2}J_{C-F}$ = 24.5 Hz), 164.7, 132.1 (d, $^{3}J_{C-F}$ = 3.5 Hz), 126.4 (d, $^{4}J_{C-F}$ = 2.0 Hz), 114.2, 90.1 (d, $^{1}J_{C-F}$ = 197.0 Hz), 62.7, 55.7, 14.0; **^{19}F NMR (376 MHz, CDCl$_3$)** δ -189.6 (d, *J* = 49.0 Hz).

ethyl 2-fluoro-3-(4-chlorophenyl)-3-oxopropanoate (1c)[6]

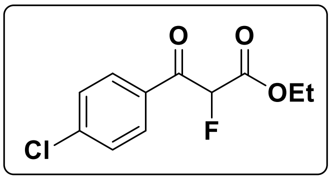

Following GPB using ethyl fluoroacetate (0.53 g, 5 mmol), diphenylphosphinic chloride (1.7 g, 5 mmol), lithium bis(trimethylsilyl)amide (1 M solution in Hexane, 1.67 g, 10 mmol and 4-chlorobenzoyl chloride (0.96 g, 5.5 mmol) with FCC (gradient from 10-20% EtOAc in 40-60 petroleum ether) afforded ethyl 2-fluoro-3-(4-chlorophenyl)-3-oxopropanoate (**1c**) as a pale yellow oil (0.4 g, 33%).

^{1}H NMR (400 MHz, $CDCl_3$): δ 8.00 – 7.94 (m, 2H), 7.53 – 7.41 (m, 2H), 5.81 (d, *J* = 49.0 Hz, 1H), 4.35 – 4.23 (m, 2H), 1.26 (t, *J* = 7.0 Hz, 3H); **^{13}C NMR (101 MHz, $CDCl_3$)** δ 188.6 (d, $^{2}J_{C-F}$ = 20.5 Hz), 164.8 (d, $^{2}J_{C-F}$ = 24.0 Hz), 141.4, 131.7, 131.1 (d, $^{3}J_{C-F}$ = 3.5 Hz), 129.4, 90.4 (d, $^{1}J_{C-F}$ = 198.0 Hz), 63.0, 14.1; **^{19}F NMR (377 MHz, $CDCl_3$)**: δ -190.0 (d, *J* = 49.0 Hz).

ethyl 2-fluoro-3-(4-trifluoromethylphenyl)-3-oxopropanoate (1d)

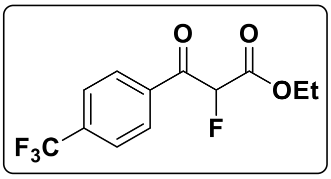

Following GPB using ethyl fluoroacetate (0.53 g, 5 mmol), diphenylphosphinic chloride (1.7 g, 5 mmol), lithium bis(trimethylsilyl)amide (1 M solution in Hexane, 1.67 g, 10 mmol and 4-trifluoromethylbenzoyl chloride (1.15 g, 5.5 mmol) with FCC (gradient from 10-20% EtOAc in 40-60 petroleum ether) afforded ethyl 2-fluoro-3-(4-trifluoromethylphenyl)-3-oxopropanoate (**1d**) as a yellow oil (0.43 g, 31%).

^{1}H NMR (400 MHz, $CDCl_3$): δ 8.15 (d, *J* = 8.5 Hz, 2H), 7.76 (d, *J* = 8.5 Hz, 2H), 5.85 (d, *J* = 49.0 Hz, 1H), 4.34 – 4.26 (m, 2H), 1.26 (t, *J* = 7.0 Hz, 3H); **^{13}C NMR (101 MHz, $CDCl_3$)** δ 189.1 (d, $^{2}J_{C-F}$ = 21.0 Hz), 164.6 (d, $^{2}J_{C-F}$ = 24.0 Hz), 135.9, 135.5, 130.0 (d, $^{3}J_{C-F}$ = 4.0 Hz), 126.0 (d, $^{4}J_{C-F}$ = 3.5 Hz), 123.3 (q, $^{1}J_{C-F}$ = 273.0 Hz), 90.4 (d, $^{1}J_{C-F}$ = 199.0 Hz), 63.1, 14.0; **^{19}F NMR (376 MHz, $CDCl_3$)**: δ -63.5, -190.5 (d, *J* = 49.0 Hz); **FTIR:** ν_{max}/cm^{-1} (neat) 2987, 1761, 1704, 1412, 1325, 1170, 1128, 1066, 889, 853 cm^{-1}; **HRMS (ESI^{+}):** calculated for $C_{12}H_{11}F_4O_3$ (ES^{+})(+H^{+}): 279.0639. Found: 279.0647.

ethyl 2-fluoro-3-(4-methylphenyl)-3-oxopropanoate (1e)[7]

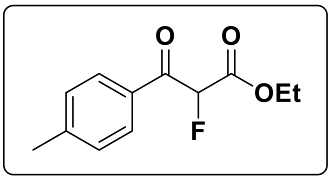

Following GPB using ethyl fluoroacetate (0.53 g, 5 mmol), diphenylphosphinic chloride (1.7 g, 5 mmol), lithium bis(trimethylsilyl)amide (1 M solution in Hexane, 1.67 g, 10 mmol and 4-methylbenzoyl chloride (0.85 g, 5.5 mmol) with FCC (gradient from 10-20% EtOAc in 40-60 petroleum ether) afforded ethyl 2-fluoro-3-(4-methylphenyl)-3-oxopropanoate (**1e**) as a colourless oil (0.60 g, 55%).

^{1}H NMR (400 MHz, $CDCl_3$): δ 7.94 (d, *J* = 7.5 Hz, 2H), 7.29 (d, *J* = 8.0 Hz, 2H), 5.84 (d, *J* = 49.0 Hz, 1H), 4.35 – 4.23 (m, 2H), 2.42 (s, 3H), 1.25 (t, *J* = 7.0 Hz, 3H); **^{13}C NMR (101 MHz, $CDCl_3$)** δ 189.2 (d, $^{2}J_{C-F}$ = 20.0 Hz), 165.2 (d, $^{2}J_{C-F}$ = 24.0 Hz), 145.9, 131.0, 129.8 (d, $^{3}J_{C-F}$ = 3.5 Hz), 129.7, 90.2 (d, $^{1}J_{C-F}$ = 197.4 Hz), 62.8, 22.0, 14.1; **^{19}F NMR (377 MHz, $CDCl_3$):** δ -190.2 (d, *J* = 49.0 Hz).

ethyl 2-fluoro-3-(4-nitrophenyl)-3-oxopropanoate (1f)[8]

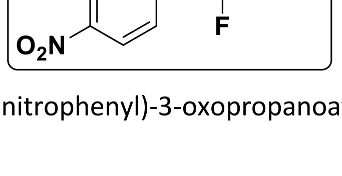

Following GPB using ethyl fluoroacetate (0.53 g, 5 mmol), diphenylphosphinic chloride (1.7 g, 5 mmol), lithium bis(trimethylsilyl)amide (1 M solution in Hexane, 1.67 g, 10 mmol and 4-nitrobenzoyl chloride (1.0 g, 5.5 mmol) with FCC (gradient from 10-20% EtOAc in 40-60 petroleum ether) afforded ethyl 2-fluoro-3-(4-nitrophenyl)-3-oxopropanoate (**1f**) as a orange oil (0.35 g, 27%).

^{1}**H NMR (400 MHz, CDCl$_3$)**: δ 8.35 (d, *J* = 9.0 Hz, 2H), 8.22 (d, *J* = 8.5 Hz, 2H), 5.84 (d, *J* = 49.0 Hz, 1H), 4.33 (m, 2H), 1.28 (t, *J* = 7.0 Hz, 3H); ^{13}**C NMR (101 MHz, CDCl$_3$)** δ 188.7 (d, $^{2}J_{C-F}$ = 21.5 Hz), 164.3 (d, $^{2}J_{C-F}$ = 24.0 Hz), 151.1, 137.8 (d, $^{3}J_{C-F}$ = 2.5 Hz), 130.8 (d, $^{4}J_{C-F}$ = 4.0 Hz), 124.1, 90.6 (d, $^{1}J_{C-F}$ = 199.0 Hz), 63.3, 14.1; ^{19}**F NMR (377 MHz, CDCl$_3$)**: δ -190.2 (d, *J* = 49.0 Hz).

ethyl 2-fluoro-3-(2-methylphenyl)-3-oxopropanoate (1g)

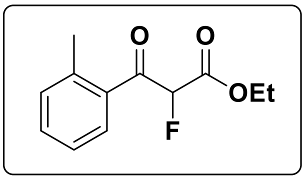

Following GPB using ethyl fluoroacetate (0.53 g, 5 mmol), diphenylphosphinic chloride (1.7 g, 5 mmol), lithium bis(trimethylsilyl)amide (1 M solution in Hexane, 1.67 g, 10 mmol and 2-methylbenzoyl chloride (0.85 g, 5.5 mmol) with FCC (gradient from 10-20% EtOAc in 40-60 petroleum ether) afforded ethyl 2-fluoro-3-(2-methylphenyl)-3-oxopropanoate (**1g**) as a colourless oil (0.13 g, 11%).

^{1}**H NMR (400 MHz, CDCl$_3$)** δ 7.76 – 7.70 (m, 1H), 7.46 – 7.40 (m, 1H), 7.31 – 7.26 (m, 2H), 5.82 (d, *J* = 49.0 Hz, 1H), 4.31 – 4.22 (m, 2H), 2.49 (s, 3H), 1.22 (t, *J* = 7.1 Hz, 3H); ^{13}**C NMR (101 MHz, CDCl$_3$)**: δ 192.5 (d, $^{2}J_{C-F}$ = 20.5 Hz), 164.9 (d, $^{2}J_{C-F}$ = 24.0 Hz), 140.2, 133.3 (m), 132.8, 132.2, 129.8 (d, $^{4}J_{C-F}$ = 4.5 Hz), 125.7, 90.3 (d, $^{1}J_{C-F}$ = 198.5 Hz), 62.6, 21.1, 13.9; ^{19}**F NMR (377 MHz, CDCl$_3$)**: δ -189.2 (dd, *J* = 49.0, 2.0Hz); **FTIR:** ν_{max}/cm^{-1} (neat) 2985, 1759, 1699, 1457, 1265, 1236, 1106, 1014, 735, 703 cm^{-1}; **HRMS (ESI$^+$):** calculated for $C_{12}H_{14}FO_3$ (ES$^+$)(+H$^+$): 225.0921 Found: 225.0922.

ethyl 2-fluoro-3-(naphthalen-2-yl)-3-oxopropanoate (1h)

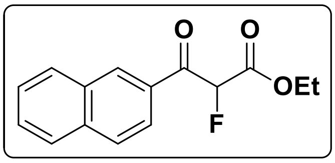

Following GPA using 2-acetylnaphthalene (0.85 g, 5 mmol), diethylcarbonate (1.7 g, 14 mmol), sodium hydride (60% dispersion in mineral oil, 0.50 g, 13 mmol), selectfluor (2.1 g, 6 mmol) and acetic acid (0.5 mL) with FCC (gradient from 20-50% DCM in 40-60 petroleum ether) afforded ethyl 2-fluoro-3-(naphthalen-2-yl)-3-oxopropanoate (**1h**) as a yellow oil (1.1 g, 84%).

^{1}**H NMR (400 MHz, CDCl$_3$)** δ 8.62 (s, 1H), 8.05 (dd, *J* = 8.5, 1.0 Hz, 1H), 7.99 (dd, *J* = 8.0, 0.5 Hz, 1H), 7.94 – 7.86 (m, 2H), 7.64 (ddd, *J* = 8.0, 7.0, 1.5 Hz, 1H), 7.58 (ddd, *J* = 8.0, 7.0, 1.5 Hz, 1H), 5.99 (d, *J* = 49.0 Hz, 1H), 4.37 – 4.25 (m, 2H), 1.26 (t, *J* = 7.0 Hz, 3H); ^{13}**C NMR (101 MHz, CDCl$_3$)** δ 189.5 (d, $^{2}J_{C-F}$ = 20.0 Hz), 165.2 (d, $^{2}J_{C-F}$ = 24.0 Hz), 136.2, 132.4 (d, $^{4}J_{C-F}$ = 4.5 Hz), 130.8 (d, $^{3}J_{C-F}$ = 2.0 Hz), 130.1, 129.5, 128.9, 128.0, 127.2, 124.3 (2C), 90.3 (d, $^{1}J_{C-F}$ = 197.5 Hz), 62.85, 14.09; ^{19}**F NMR (376 MHz, CDCl$_3$)** δ -189.6 (d, *J* = 49.0 Hz); **FTIR:** ν_{max}/cm^{-1} (neat) 3061, 2983, 1759, 1687, 1626, 1467, 1256, 1229, 1188, 1097 1019 cm^{-1}; **HRMS (ESI$^+$):** calculated for $C_{15}H_{13}FO_3$ (ES$^+$)(+H$^+$): 261.0921. Found: 261.0926.

ethyl 2-fluoro-3-oxo-3-(thiophen-2-yl)propanoate (1i)[9]

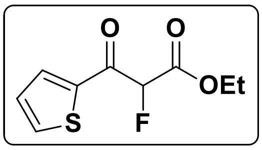

Following GPA using 2-acetylthiophene (1.3 g, 10 mmol), diethylcarbonate (3.3 g, 28 mmol), sodium hydride (60% dispersion in mineral oil, 1.0 g, 25 mmol), selectfluor (4.3 g, 12 mmol) and acetic acid (1 mL) with FCC (gradient from 30-60% DCM in 40-60 petroleum ether) afforded ethyl 2-fluoro-3-oxo-3-(thiophen-2-yl)propanoate (**1i**) as an orange oil (1.7 g, 81%).

^{1}**H NMR (400 MHz, CDCl$_3$)** δ 8.03 – 7.99 (m, 1H), 7.79 (dd, *J* = 5.0, 1.0 Hz, 1H), 7.18 (dd, *J* = 5.0, 4.0 Hz, 1H), 5.69 (d, *J* = 49.0 Hz, 1H), 4.36 – 4.23 (m, 2H), 1.27 (t, *J* = 7.0 Hz, 3H); ^{13}**C NMR (101 MHz, CDCl$_3$)** δ 182.5 (d, $^{2}J_{C-F}$ = 22.0 Hz), 164.7 (d, $^{2}J_{C-F}$ = 24.5 Hz), 139.6 (d, $^{3}J_{C-F}$ = 3.0 Hz), 136.4, 135.5 (d, $^{4}J_{C-F}$ = 7.0 Hz), 128.8, 90.6 (d, $^{1}J_{C-F}$ = 199.0 Hz), 62.91, 14.06; ^{19}**F NMR (377 MHz, CDCl$_3$)** δ -189.2 (dd, *J* = 49.0, 1.5 Hz); **FTIR:** ν_{max}/cm^{-1} (neat) 3106, 2984, 1756, 1665, 1410, 1251, 1202, 1095, 1061, 1018 cm^{-1}.

2-fluoro-3-oxo-3-phenylpropanenitrile (1j)[10]

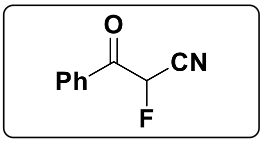

Following GPB using ethyl fluoroacetonitrile (0.3 g, 5 mmol), diphenylphosphinic chloride (1.7 g, 5 mmol), lithium bis(trimethylsilyl)amide (1 M solution in Hexane, 1.67 g, 10 mmol and benzoyl chloride (0.77 g, 5.5 mmol) with FCC (gradient from 10-20% EtOAc in 40-60 petroleum ether) afforded 2-fluoro-3-oxo-3-phenylpropanenitrile (**1j**) as a yellow oil (0.3 g, 37%).

^{1}H NMR (400 MHz, $CDCl_3$): δ 8.00 (d, J = 8.0 Hz, 2H), 7.72 (t, J = 7.5 Hz, 1H), 7.57 (t, J = 8.0 Hz, 2H), 6.14 (d, J = 46.7 Hz, 1H); **^{13}C NMR (101 MHz, $CDCl_3$)** δ 184.9 (d, $^2J_{C\text{-}F}$ = 20.0 Hz), 135.7, 131.7, 129.5, 129.4 (d, $^3J_{C\text{-}F}$ = 3.0 Hz), 112.4 (d, $^2J_{C\text{-}F}$ = 30.0 Hz), 79.7 (d, $^1J_{C\text{-}F}$ = 197.2 Hz); **^{19}F NMR (377 MHz, $CDCl_3$)** δ -192.0 (d, J = 46.5 Hz).

1-phenyl-2-(phenylsulfonyl)ethanone (s3)[11]

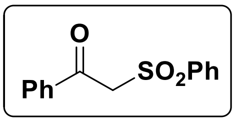

To a solution of acetophenone (1.2 g, 10 mmol) in THF (25 mL) under nitrogen at -78 °C was added LiHMDS (1.0 M in hexanes, 12 mL, 12 mmol) and the solution was stirred at -78 °C for 30 minutes. Acetic anhydride (2.0 g, 20 mmol) was then added and the resulting mixture was stirred for 30 minutes before being warmed to room temperature and stirred for a further 2 hours. The mixture was then diluted with sat. $NaHCO_3$ (30 mL) and extracted with EtOAc (4 x 30 mL). The combined organic layers were then washed with brine (30 mL), dried over anhydrous magnesium sulfate and concentrated under vacuum. The crude residue was then purified by FCC (gradient from 20-30% DCM in 40-60 petroleum ether).

The purified oil and sodium *p*-toluenesulfinate (1.5 g, 7.5 mmol) were then dissolved in MeCN (15 mL) and H_2O (4 mL) before the addition of iodine (1.6 g, 6.2 mmol). The resulting mixture was heated at 70 °C overnight and then cooled to room temperature and diluted with water (30 mL). The product was extracted with EtOAc (3 x 30 mL) and the combined organic layers dried over anhydrous magnesium sulfate and concentrated under vacuum. Purification by FCC (30% EtOAc in 40-60 petroleum ether) afforded 1-phenyl-2-(phenylsulfonyl)ethanone (**s3**) as a yellow solid (1.1 g, 39%).

^{1}H NMR (400 MHz, $CDCl_3$) δ 7.97 – 7.92 (m, 2H), 7.79 – 7.74 (m, 2H), 7.65 – 7.59 (m, 1H), 7.51 – 7.46 (m, 2H), 7.36 – 7.31 (m, 2H), 4.71 (s, 2H), 2.44 (s, 3H); **^{13}C NMR (101 MHz, $CDCl_3$)** δ 188.3, 145.5, 135.9, 135.9, 134.5, 130.0, 129.5, 129.0, 128.8, 63.7, 21.9.

2-fluoro-1-phenyl-2-(phenylsulfonyl)ethanone (1k)[12]

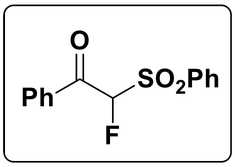

To a solution of sodium hydride (60% dispersion in mineral oil, 73 mg, 1.8 mmol) in THF (3.6 mL) under nitrogen at 0 °C was added 1-phenyl-2-(phenylsulfonyl)ethanone (**s3**) (0.50 g, 1.8 mmol) in THF (3.6 mL) dropwise and the solution was stirred at 0 °C for 2 hours. Selectfluor (0.65 g, 1.8 mmol) was then added in 1 portion and the resulting mixture was stirred at room temperature overnight. The reaction mixture was then diluted with water (30 mL) and extracted with DCM (3 x 30mL). The combined organic layers were then washed with brine (30 mL), dried over anhydrous magnesium sulfate and concentrated under vacuum. Purification by FCC (gradient from 50-100% DCM in 40-60 petroleum ether) afforded 2-fluoro-1-phenyl-2-(phenylsulfonyl)ethanone (**1k**) as a white solid (0.37 g, 71%).

^{1}H NMR (400 MHz, $CDCl_3$) δ 8.03 (d, J = 7.5 Hz, 2H), 7.75 (d, J = 8.5 Hz, 2H), 7.71 – 7.65 (m, 1H), 7.56 – 7.50 (m, 2H), 7.37 (d, J = 8.0 Hz, 2H), 6.32 (d, J = 48.0 Hz, 1H), 2.47 (s, 3H); **^{13}C NMR (101 MHz,**

CDCl₃) δ 186.7 (d, $^{2}J_{C\text{-}F}$ = 17.5 Hz), 146.8, 135.1, 134.1, 131.6, 130.1, 130.0, 129.9 (d, $^{3}J_{C\text{-}F}$ = 2.5 Hz), 128.9, 100.3 (d, $^{1}J_{C\text{-}F}$ = 231.5 Hz), 21.9; **^{19}F NMR (376 MHz, $CDCl_3$)** δ -179.6 (d, *J* = 48.0 Hz).

N-methoxy-*N*-methyl-3-oxo-3-phenylpropanamide (s4)[13]

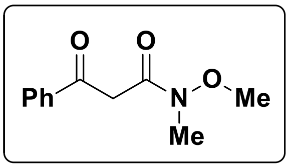

To a solution of diisopropylamine (2.2 g, 21 mmol) in THF (29 mL) under nitrogen at -78 °C was added *n*-butyllithium (2.4 M in hexanes, 8.1 mL, 19 mmol) and the resulting solution stirred at -78 °C for 1 hour. A solution of *N*-methoxy-*N*-methylacetamide (1.0 g, 9.7 mmol) in THF (10 mL) was then added and the reaction mixture stirred for 1 hour before the addition of benzoyl chloride (1.4 g, 9.7 mmol). After stirring at -78 °C for 4 hours the mixture was warmed to room temperature, diluted with 1M HCl (50 mL) and extracted with EtOAc (4 x 40 mL). The combined organic layers were then dried over anhydrous magnesium sulfate and concentrated under vacuum. Purification by FCC (30% EtOAc in 40-60 petroleum ether) afforded *N*-methoxy-*N*-methyl-3-oxo-3-phenylpropanamide (**s4**) as an orange oil (1.8 g, 90%)(2:1, keto:enol).

Keto: ^{1}H NMR (400 MHz, $CDCl_3$) δ 8.01 – 7.93 (m, 2H), 7.52 – 7.39 (m, 3H), 4.13 (s, 2H), 3.65 (s, 3H), 3.23 (s, 3H); **Enol: ^{1}H NMR (400 MHz, $CDCl_3$)** δ 14.26 (s, 1H), 7.84 – 7.77 (m, 2H), 7.62 – 7.55 (m, 2H), 7.50 – 7.39 (m, 1H), 6.08 (s, 1H), 3.75 (s, 3H), 3.26 (s, 3H); **^{13}C NMR (101 MHz, $CDCl_3$)** δ 193.6, 172.8, 171.6, 168.6, 136.4, 134.5, 133.7, 131.0, 128.8, 128.5, 126.1, 84.5, 61.6, 61.5, 44.6, 32.3, 32.1.

2-fluoro-*N*-methoxy-*N*-methyl-3-oxo-3-phenylpropanamide (1l)

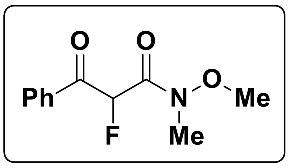

Following GPC using *N*-methoxy-*N*-methyl-3-oxo-3-phenylpropanamide (**s4**) (1.4 g, 6.8 mmol) and selectfluor (2.9 g, 8.2 mmol) with FCC (gradient from 25-30% EtOAc in 40-60 petroleum ether) afforded 2-fluoro-*N*-methoxy-*N*-methyl-3-oxo-3-phenylpropanamide (**1l**) as a yellow oil (1.4 g, 90%).

^{1}H NMR (400 MHz, $CDCl_3$) δ 8.04 (d, *J* = 8.0 Hz, 2H), 7.62 (t, *J* = 7.5 Hz, 1H), 7.49 (t, *J* = 7.5 Hz, 2H), 6.14 (d, *J* = 48.5 Hz, 1H), 3.64 (s, 3H), 3.23 (s, 3H); **^{13}C NMR (101 MHz, $CDCl_3$)** δ 190.2 (d, $^{2}J_{C\text{-}F}$ = 20.5 Hz), 165.9 (d, $^{2}J_{C\text{-}F}$ = 22.0 Hz), 134.4, 134.0, 129.4 (d, $^{3}J_{C\text{-}F}$ = 3.0 Hz), 128.9, 90.0 (d, $^{1}J_{C\text{-}F}$ = 190.5 Hz), 61.7, 32.6; **^{19}F NMR (376 MHz, $CDCl_3$)** δ -190.9 (d, *J* = 48.5 Hz); **FTIR:** ν_{max}/cm^{-1} (neat) 3008, 2947, 1685, 1680, 1450, 1340, 1219, 1053, 972 cm^{-1}; **HRMS (ESI^+):** calculated for $C_{11}H_{13}FNO_3$ (ES^+)(+H^+): 226.0874. Found: 226.0867.

ethyl 2-fluoro-3-oxobutanoate (1m)[14]

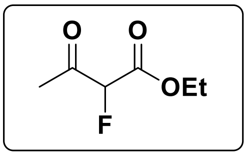

Following GPC using ethyl 3-oxobutanoate (0.65 g, 5 mmol) and selectfluor (2.13 g, 6 mmol) with FCC (gradient from 10-20% EtOAc in 40-60 petroleum ether) afforded ethyl 2-fluoro-3-oxobutanoate (**1m**) as a colourless oil (0.5 g, 77%).

^{1}H NMR (400 MHz, $CDCl_3$) δ 5.19 (d, *J* = 49.5 Hz, 1H), 4.34 – 4.27 (m, 2H), 2.34 (d, *J* = 4.0 Hz, 3H), 1.32 (t, *J* = 7.0 Hz, 3H); **^{13}C NMR (101 MHz, $CDCl_3$)** δ 199.2 (d, $^{2}J_{C\text{-}F}$ = 24.0 Hz), 164.1 (d, $^{2}J_{C\text{-}F}$ = 24.0 Hz), 91.6 (d, $^{1}J_{C\text{-}F}$ = 198.0 Hz), 62.9, 26.2, 14.1; **^{19}F NMR (377 MHz, $CDCl_3$):** δ -193.1 (dq, *J* = 49.5, 4.0 Hz).

ethyl 2-fluoro-3-oxopentanoate (1n)[15]

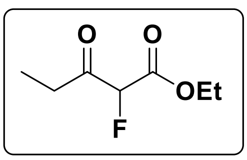

Following GPC using ethyl 3-oxopentanoate (0.72 g, 5 mmol) and selectfluor (2.13 g, 6 mmol) with FCC (gradient from 10-20% EtOAc in 40-60 petroleum

ether) afforded ethyl 2-fluoro-3-oxopentanoate (**1n**) as a colourless oil (0.7 g, 86%).

^{1}H NMR (400 MHz, CDCl$_3$): δ 5.21 (d, *J* = 49.5 Hz, 1H), 4.30 (q, *J* = 7.0 Hz, 2H), 2.81 – 2.61 (m, 2H), 1.32 (t, *J* = 7.0 Hz, 3H), 1.10 (t, *J* = 7.0 Hz, 3H); **^{13}C NMR (101 MHz, CDCl$_3$)** δ 202.0 (d, $^{2}J_{C-F}$ = 23.0 Hz), 164.4 (d, $^{2}J_{C-F}$ = 24.0 Hz), 91.4 (d, $^{1}J_{C-F}$ = 198.5 Hz), 62.8, 32.0, 14.1, 6.9; **^{19}F NMR (376 MHz, CDCl$_3$)**: δ -195.1 (d, *J* = 49.4 Hz).

ethyl 2-fluoro-4-methyl-3-oxopentanoate (1o)[16]

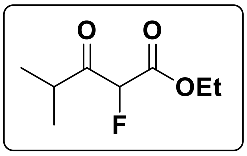

Following GPC using ethyl 4-methyl-3-oxo-pentanoate (0.79 g, 5 mmol) and selectfluor (2.13 g, 6 mmol) with FCC (gradient from 10-20% EtOAc in 40-60 petroleum ether) afforded ethyl 2-fluoro-4-methyl-3-oxopentanoate (**1o**) as a colourless oil (0.62 g, 70%).

^{1}H NMR (400 MHz, CDCl$_3$): δ 5.29 (d, *J* = 49.0 Hz, 1H), 4.30 (q, *J* = 7.0 Hz, 2H), 3.16 – 3.05 (m, 1H), 1.31 (t, *J* = 7.0 Hz, 3H), 1.15 (d, *J* = 3.5 Hz, 3H), 1.13 (d, *J* = 3.5 Hz, 3H); **^{13}C NMR (101 MHz, CDCl$_3$)** δ 205.0 (d, $^{2}J_{C-F}$ = 22.0 Hz), 164.5 (d, $^{2}J_{C-F}$ = 24.0 Hz), 90.8 (d, $^{1}J_{C-F}$ = 198.0 Hz), 62.7, 37.1, 17.9, 17.5, 14.1; **^{19}F NMR (376 MHz, CDCl$_3$)**: δ -195.7 (dd, *J* = 49.5, 2.5 Hz).

ethyl 2-fluoro-4,4-dimethyl-3-oxopentanoate (1p)

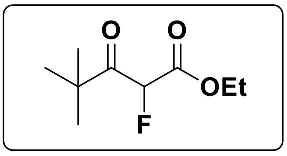

Following GPB using ethyl fluoroacetate (0.53 g, 5 mmol), diphenylphosphinic chloride (1.7 g, 5 mmol), lithium bis(trimethylsilyl)amide (1 M solution in Hexane, 1.67 g, 10 mmol and 4-2,2-dimethylpropanoyl chloride (0.66 g, 5.5 mmol) with FCC (gradient from 10-20% EtOAc in 40-60 petroleum ether) afforded ethyl 2-fluoro-4,4-dimethyl-3-oxopentanoate (**1p**) as a yellow oil (0.43 g, 31%).

^{1}H NMR (400 MHz, CDCl$_3$): δ 5.47 (d, *J* = 49.0 Hz, 1H), 4.30 (q, *J* = 7.0 Hz, 2H), 1.31 (t, *J* = 7.0 Hz, 3H), 1.25 (d, *J* = 1.0 Hz, 9H); **^{13}C NMR (101 MHz, CDCl$_3$)** δ 205.3 (d, $^{2}J_{C-F}$ = 18.0 Hz), 165.1 (d, $^{2}J_{C-F}$ = 23.5 Hz), 89.3 (d, $^{1}J_{C-F}$ = 198.0 Hz), 62.6, 44.7 $^{3}J_{C-F}$ = 2.0 Hz, 25.9 (d, $^{4}J_{C-F}$ = 2.0 Hz), 14.2; **^{19}F NMR (377 MHz, CDCl$_3$)**: δ -190.6 (d, *J* = 49.0 Hz); **FTIR:** ν_{max}/cm^{-1} (neat) 2975, 1760, 1717, 1479, 1369, 1213, 1094, 1017, 939, 736 cm^{-1}; **HRMS (ESI$^+$):** calculated for $C_9H_{16}FO_3$ (ES$^+$)(+H$^+$): 191.1078. Found: 191.1081.

ethyl 3-cyclohexyl-2-fluoro-3-oxopropanoate (1q)

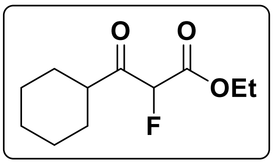

Following GPA using 1-cyclohexylethan-1-one (0.7 g, 5 mmol), diethylcarbonate (1.7 g, 14 mmol), sodium hydride (60% dispersion in mineral oil, 0.3 g, 12.5 mmol), selectfluor (2.13 g, 6 mmol) and acetic acid (0.5 mL) with FCC (10% EtOAc in 40-60 petroleum ether) afforded ethyl 3-cyclohexyl-2-fluoro-3-oxopropanoate (**1q**) as a colourless oil (0.52 g, 48%).

^{1}H NMR (400 MHz, CDCl$_3$): δ 5.26 (d, *J* = 49.5 Hz, 1H), 4.30 (q, *J* = 7.0 Hz, 2H), 2.96 – 2.77 (m, 1H), 1.96 – 1.59 (m, 6H), 1.45 – 1.16 (m, 7H); **^{13}C NMR (101 MHz, CDCl$_3$)** δ 203.8 (d, $^{2}J_{C-F}$ = 21.5 Hz), 164.4 (d, $^{2}J_{C-F}$ = 24.0 Hz), 90.7 (d, $^{1}J_{C-F}$ = 197.5 Hz), 62.5, 46.5, 28.1, 27.6, 25.7, 25.5, 25.3, 14.0; **^{19}F NMR (376 MHz, CDCl$_3$)** δ -195.7 (dd, *J* = 49.5, 3.0 Hz); **FTIR:** ν_{max}/cm^{-1} (neat) 2932, 2857, 1757, 1725, 1450, 1261, 1146, 1096, 1023, 993 cm^{-1}. **HRMS (ESI$^+$):** calculated for $C_{11}H_{18}FO_3$ (ES$^+$)(+H$^+$): 217.1234 Found: 217.1233.

ethyl 2-fluoro-3-oxooct-7-enoate (1r)

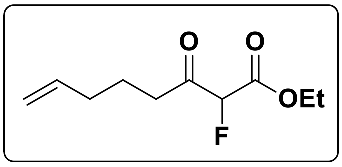

Following GPD using hex-5-enoic acid (0.25 g, 2.2 mmol), $(COCl)_2$ (0.31 g, 2.4 mmol), ethyl fluoroacetate (0.21 g, 2.0 mmol), diphenylphosphinic chloride (0.47 g, 2.0 mmol) and LiHMDS (6.0 mL, 6.0 mmol) with FCC (gradient from 10-50% EtOAc in 40-60 petroleum ether) afforded ethyl 2-fluoro-3-oxooct-7-enoate (**1r**) as a yellow oil (95 mg, 24%).

^{1}H NMR (400 MHz, $CDCl_3$) δ 5.74 (ddt, J = 17.0, 10.0, 6.5 Hz, 1H), 5.18 (d, J = 49.5 Hz, 1H), 5.05 – 4.96 (m, 2H), 4.29 (q, J = 7.0 Hz, 2H), 2.75 – 2.59 (m, 2H), 2.07 (q, J = 7.0 Hz, 2H), 1.72 (p, J = 7.0 Hz, 2H), 1.31 (t, J = 7.0 Hz, 3H); **^{13}C NMR (101 MHz, $CDCl_3$)** δ 201.3 (d, $^{2}J_{C-F}$ = 23.0 Hz), 164.3 (d, $^{2}J_{C-F}$ = 24.0 Hz), 137.6, 115.8, 91.5 (d, $^{1}J_{C-F}$ = 198.5 Hz), 62.8, 37.7, 32.9, 21.8, 14.1; **^{19}F NMR (376 MHz, $CDCl_3$)** δ -194.8 (d, J = 49.5 Hz); **FTIR:** ν_{max}/cm^{-1} (neat) 2980, 2940, 1758, 1732, 1371, 1258, 1133, 1096, 1016 cm^{-1}; **HRMS (ESI^{+}):** calculated for $C_{10}H_{16}FO_3$ (ES^{+})(+H^{+}): 203.1078. Found: 203.1080.

ethyl 2-fluoro-3-oxo-3-(1-tosylpyrrolidin-2-yl)propanoate (1s)

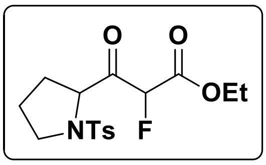

Following GPD using *N*-tosyl-*L*-proline (0.59 g, 2.2 mmol), $(COCl)_2$ (0.31 g, 2.4 mmol), ethyl fluoroacetate (0.21 g, 2.0 mmol), diphenylphosphinic chloride (0.47 g, 2.0 mmol) and LiHMDS (6.0 mL, 6.0 mmol) with FCC (20% EtOAc in 40-60 petroleum ether) afforded ethyl 2-fluoro-3-oxo-3-(1-tosylpyrrolidin-2-yl)propanoate (**1s**) as a yellow oil (0.24 g, 33%)(1:1 mixture of diastereoisomers).

^{1}H NMR (400 MHz, $CDCl_3$) δ 7.78 – 7.69 (m, 2H), 7.38 – 7.29 (m, 2H), 5.78 – 5.45 (m, 1H), 4.93 – 4.56 (m, 1H), 4.41 – 4.20 (m, 2H), 3.55 – 3.39 (m, 1H), 3.34 – 3.22 (m, 1H), 2.48 – 2.40 (m, 3H), 2.09 – 1.72 (m, 3H), 1.70 – 1.60 (m, 1H), 1.40 – 1.20 (m, 3H); **^{13}C NMR (101 MHz, $CDCl_3$)** δ 199.3 (d, $^{2}J_{C-F}$ = 21.0 Hz), 198.9 (d, $^{2}J_{C-F}$ = 21.0 Hz), 164.2 (d, $^{2}J_{C-F}$ = 24.0 Hz), 164.2 (d, $^{2}J_{C-F}$ = 23.0 Hz), 144.3, 144.2, 134.2, 134.1, 130.0, 129.8, 127.8, 127.7, 90.5 (d, $^{1}J_{C-F}$ = 195.5 Hz), 90.4 (d, $^{1}J_{C-F}$ = 195.5 Hz), 64.2 (2C), 63.1, 63.0, 49.0 (2C), 29.7 – 29.4 (m, 2C), 25.0 – 24.7 (m, 2C), 21.7 (2C), 14.1 (2C); **^{19}F NMR (376 MHz, $CDCl_3$)** δ -197.4 – -197.7 (m); **FTIR:** ν_{max}/cm^{-1} (neat) 2983, 1744, 1340, 1201, 1156, 1092, 1017 cm^{-1}; **HRMS (ESI^{+}):** calculated for $C_{16}H_{21}FNO_5S$ (ES^{+})(+H^{+}): 358.1119. Found: 358.1118.

Allylation Products

ethyl 2-benzoyl-4-{[(*tert*-butoxycarbonyl)amino]methyl}-2-fluoropent-4-enoate (3a)

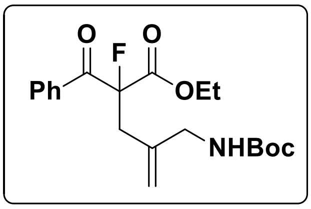

Following GPE with ethyl 2-fluoro-3-oxo-3-phenylpropanoate (**1a**) (63 mg, 0.3 mmol), *tert*-butyl 5-methylene-2-oxo-1,3-oxazinane-3-carboxylate (43 mg, 0.2 mmol), $Pd(dba)_2$ (6 mg, 0.01 mmol) and *N*,*N*-diisopropyldibenzo[*d*,*f*][1,3,2]dioxaphosphepin-6-amine (10 mg, 0.03 mmol) in CH_2Cl_2 (2 mL) with FCC (20 % ethyl acetate in petroleum ether) afforded ethyl 2-benzoyl-4-{[(*tert*-butoxycarbonyl)amino]methyl}-2-fluoropent-4-enoate (**3a**) as a pale yellow oil (74 mg, 97%).

^{1}H NMR (400 MHz, $CDCl_3$): δ 8.02 (d, J = 8.0 Hz, 2H), 7.58 (t, J = 7.5 Hz, 1H), 7.45 (t, J = 8.0 Hz, 2H), 5.14 (s, 1H), 5.03 (s, 1H), 4.77 (br, 1H), 4.35 – 4.16 (m, 2H), 3.76 (d, J = 4.0 Hz, 2H), 3.14 (dd, J = 33.0, 15.0 Hz, 1H), 2.97 (dd, J = 18.5, 15.5 Hz, 1H), 1.43 (s, 9H), 1.20 (t, J = 7.0 Hz, 3H); **^{13}C NMR (101 MHz, $CDCl_3$)** δ 191.3 (d, $^{2}J_{C-F}$ = 26.0 Hz), 167.1 (d, $^{2}J_{C-F}$ = 26.0 Hz), 155.9, 139.8, 134.1, 133.8 (d, $^{3}J_{C-F}$ = 3.0 Hz), 129.9 (d, $^{4}J_{C-F}$ = 5.5 Hz), 128.8, 116.2, 100.0 (d, $^{1}J_{C-F}$ = 200.0 Hz), 79.5, 62.9, 45.8, 38.2 (d, $^{2}J_{C-F}$ = 20.5 Hz), 28.5, 14.1; **^{19}F NMR (377 MHz, $CDCl_3$)**: δ -157.6 (dd, J = 33.0, 18.5 Hz); **FTIR:** ν_{max}/cm^{-1} (neat)

2981, 2931, 1755, 1697, 1509, 1449, 1267, 1240, 1168, 908, 729, 694 cm^{-1}; **HRMS (ESI$^+$):** calculated for $C_{20}H_{26}FNO_5Na$ (ES$^+$)(+Na$^+$): 402.1687. Found: 402.1701.

ethyl 4-{[(*tert*-butoxycarbonyl)amino]methyl}-2-fluoro-2-(4-methoxybenzoyl)pent-4-enoate (3b)

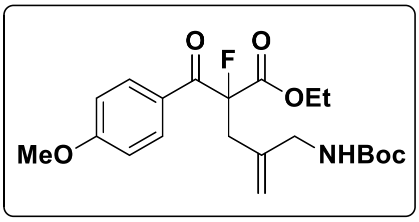

Following GPE with ethyl 2-fluoro-3-(4-methoxyphenyl)-3-oxopropanoate (**1b**) (72 mg, 0.3 mmol), *tert*-butyl 5-methylene-2-oxo-1,3-oxazinane-3-carboxylate (43 mg, 0.2 mmol), Pd(dba)$_2$ (6 mg, 0.01 mmol) and *N*,*N*-diisopropyldibenzo[*d*,*f*][1,3,2]dioxaphosphepin-6-amine (10 mg, 0.03 mmol) in CH_2Cl_2 (2 mL) with FCC (20 % ethyl acetate in petroleum ether) afforded ethyl 4-{[(*tert*-butoxycarbonyl)amino]methyl}-2-fluoro-2-(4-methoxybenzoyl)pent-4-enoate (**3b**) as an orange oil (78 mg, 95%).

^{1}H NMR (400 MHz, CDCl$_3$): δ 8.04 (dd, *J* = 9.0, 1.5 Hz, 2H), 6.91 (d, *J* = 9.0 Hz, 2H), 5.12 (s, 1H), 5.02 (s, 1H), 4.79 (br, 1H), 4.34 – 4.11 (m, 2H), 3.86 (s, 3H), 3.75 (s, 1H), 3.13 (dd, *J* = 34.0, 15.0 Hz, 1H), 2.94 (dd, *J* = 18.0, 15.5 Hz, 1H), 1.43 (s, 9H), 1.19 (t, *J* = 7.0 Hz, 3H); **^{13}C NMR (101 MHz, CDCl$_3$)** δ 189.4 (d, $^2J_{C-F}$ = 25.0 Hz), 167.4 (d, $^2J_{C-F}$ = 26.0 Hz), 164.3, 155.9, 139.9, 132.5 (d, $^4J_{C-F}$ = 6.0 Hz), 126.5 (d, $^3J_{C-F}$ = 3.5 Hz), 116.0, 114.1, 100.0 (d, $^1J_{C-F}$ = 200.5 Hz), 79.5, 62.7, 55.6, 45.8, 38.2 (d, $^2J_{C-F}$ = 20.5 Hz), 28.5, 14.1; **^{19}F NMR (377 MHz, CDCl$_3$)**: δ -156.9 (dd, *J* = 34.0, 18.0 Hz). **FTIR:** ν_{max}/cm^{-1} (neat) 2979, 1756, 1687, 1601, 1512, 1252, 1175, 1027, 848, 764 cm^{-1}; **HRMS (ESI$^+$):** calculated for $C_{21}H_{28}FNO_6Na$ (ES$^+$)(+Na$^+$): 432.1793. Found: 432.1799.

ethyl 4-{[(*tert*-butoxycarbonyl)amino]methyl}-2-fluoro-2-(4-chloromethylbenzoyl)pent-4-enoate (3c)

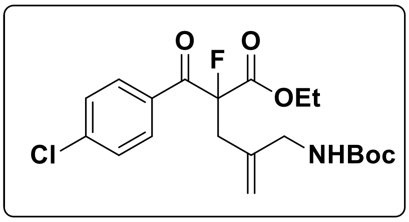

Following GPE with ethyl 2-fluoro-3-(4-chloromethylphenyl)-3-oxopropanoate (**1c**) (73 mg, 0.3 mmol), *tert*-butyl 5-methylene-2-oxo-1,3-oxazinane-3-carboxylate (43 mg, 0.2 mmol), Pd(dba)$_2$ (6 mg, 0.01 mmol) and *N*,*N*-diisopropyldibenzo[*d*,*f*][1,3,2]dioxaphosphepin-6-amine (10 mg, 0.03 mmol) in CH_2Cl_2 (2 mL) with FCC (20 % ethyl acetate in petroleum ether) afforded ethyl 4-{[(*tert*-butoxycarbonyl)amino]methyl}-2-fluoro-2-(4-chloromethylbenzoyl)pent-4-enoate (**3c**) as a colourless oil (74 mg, 89%).

^{1}H NMR (400 MHz, CDCl$_3$): δ 7.98 (dd, *J* = 8.5, 1.5 Hz, 2H), 7.44 – 7.39 (m, 2H), 5.13 (s, 1H), 5.02 (s, 1H), 4.76 (br, 1H), 4.31 – 4.14 (m, 2H), 3.74 (d, *J* = 5.0 Hz, 2H), 3.12 (dd, *J* = 33.0, 15.5 Hz, 1H), 2.95 (dd, *J* = 19.0, 15.5 Hz, 1H), 1.42 (s, 9H), 1.20 (t, *J* = 7.0 Hz, 3H); **^{13}C NMR (101 MHz, CDCl$_3$)** δ 190.2 (d, $^2J_{C-F}$ = 26.0 Hz), 166.8 (d, $^2J_{C-F}$ = 26.0 Hz), 155.9, 140.8, 139.7, 132.0 (d, $^3J_{C-F}$ = 3.5 Hz), 131.4 (d, $^4J_{C-F}$ = 6.0 Hz), 129.2, 116.2, 100.0 (d, $^1J_{C-F}$ = 200.0 Hz), 79.6, 63.0, 45.8, 38.1 (d, $^2J_{C-F}$ = 20.5 Hz), 28.5, 14.1; **^{19}F NMR (377 MHz, CDCl$_3$):** δ -157.7 (dd, *J* = 33.0, 19.0 Hz); **FTIR:** ν_{max}/cm^{-1} (neat) 2925, 1752, 1700, 1589, 1275, 1261, 1170, 750 cm^{-1}; **HRMS (ESI$^+$):** calculated for $C_{20}H_{25}ClFNO_5Na$ (ES$^+$)(+Na$^+$): 436.1297 Found: 436.1312.

ethyl 4-{[(*tert*-butoxycarbonyl)amino]methyl}-2-fluoro-2-(4-trifluoromethylbenzoyl) pent-4-enoate (3d)

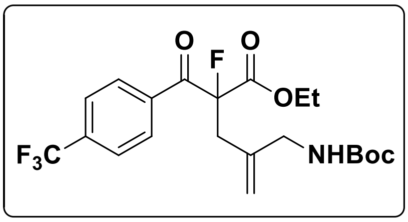

Following GPE with ethyl 2-fluoro-3-(4-trifluoromethylphenyl)-3-oxopropanoate (**1d**) (84 mg, 0.3 mmol), *tert*-butyl 5-

methylene-2-oxo-1,3-oxazinane-3-carboxylate (43 mg, 0.2 mmol), Pd(dba)$_2$ (6 mg, 0.01 mmol) and *N*,*N*-diisopropyldibenzo[*d*,*f*][1,3,2]dioxaphosphepin-6-amine (10 mg, 0.03 mmol) in CH_2Cl_2 (2 mL) with FCC (20 % ethyl acetate in petroleum ether) afforded ethyl 4-{[(*tert*-butoxycarbonyl)amino]methyl}-2-fluoro-2-(4-trifluoromethylbenzoyl)pent-4-enoate (**3d**) as a pale yellow oil (76 mg, 85%).

^{1}H NMR (400 MHz, $CDCl_3$): δ 8.13 (d, *J* = 8.0 Hz, 2H), 7.71 (d, *J* = 8.5 Hz, 2H), 5.14 (s, 1H), 5.03 (s, 1H), 4.76 (br, 1H), 4.29 – 4.13 (m, 2H), 3.75 (d, *J* = 5.5 Hz, 1H), 3.13 (dd, *J* = 32.5, 15.5 Hz, 1H), 2.97 (dd, *J* = 19.5, 15.5 Hz, 1H), 1.42 (s, 9H), 1.21 (t, *J* = 7.0 Hz, 3H); **^{13}C NMR (101 MHz, $CDCl_3$)** δ 190.8 (d, $^2J_{C\text{-}F}$ = 26.5 Hz), 166.6 (d, $^2J_{C\text{-}F}$ = 26.0 Hz), 155.9, 139.6, 136.5, 135.2 (q, $^2J_{C\text{-}F}$ = 33.0 Hz), 130.3 (d, $^4J_{C\text{-}F}$ = 6.0 Hz), 125.8 (d, $^3J_{C\text{-}F}$ = 3.5 Hz), 123.5 (q, $^1J_{C\text{-}F}$ = 273.0 Hz), 116.3, 100.1 (d, $^1J_{C\text{-}F}$ = 200.0 Hz), 79.6, 63.2, 45.8, 38.1 (d, $^2J_{C\text{-}F}$ = 20.5 Hz), 28.5, 14.1; **^{19}F NMR (377 MHz, $CDCl_3$)**: δ -63.4 (s), -158.2 (dd, *J* = 32.5, 19.5 Hz); **FTIR:** ν_{max}/cm^{-1} (neat) 2986, 1758, 1715, 1638, 1409, 1325, 1314, 1124, 1115, 1066, 849, 701 cm^{-1}; **HRMS (ESI$^+$):** calculated for $C_{21}H_{25}F_4NO_5Na$ (ES$^+$)(+Na$^+$): 470.1561. Found: 470.1556.

ethyl 4-{[(*tert*-butoxycarbonyl)amino]methyl}-2-fluoro-2-(4-methylbenzoyl)pent-4-enoate (3e)

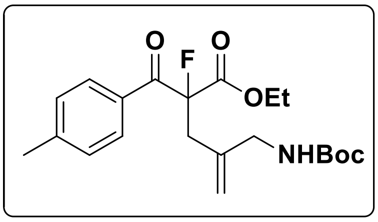

Following GPE with ethyl 2-fluoro-3-(4-methylphenyl)-3-oxopropanoate (**1e**) (67 mg, 0.3 mmol), *tert*-butyl 5-methylene-2-oxo-1,3-oxazinane-3-carboxylate (43 mg, 0.2 mmol), Pd(dba)$_2$ (6 mg, 0.01 mmol) and *N*,*N*-diisopropyldibenzo[*d*,*f*][1,3,2]dioxaphosphepin-6-amine (10 mg, 0.03 mmol) in CH_2Cl_2 (2 mL) with FCC (20 % ethyl acetate in petroleum ether) afforded ethyl 4-{[(*tert*-butoxycarbonyl)amino]methyl}-2-fluoro-2-(4-methylbenzoyl)pent-4-enoate (**3e**) as a colourless oil (60 mg, 76%).

^{1}H NMR (400 MHz, $CDCl_3$): δ 7.93 (d, *J* = 7.0 Hz, 2H), 7.24 (d, *J* = 8.0 Hz, 2H), 5.13 (s, 1H), 5.02 (s, 1H), 4.78 (br, 1H), 4.21 (tdd, *J* = 12.0, 7.0, 3.5 Hz, 2H), 3.75 (s, 2H), 3.13 (dd, *J* = 33.5, 15.5 Hz, 1H), 2.95 (dd, *J* = 18.0, 15.5 Hz, 1H), 2.40 (s, 3H), 1.43 (s, 9H), 1.19 (t, *J* = 7.0 Hz, 3H); **^{13}C NMR (101 MHz, $CDCl_3$)** δ 190.7 (d, (d, $^2J_{C\text{-}F}$ = 25.5 Hz), 167.2 (d, (d, $^2J_{C\text{-}F}$ = 26.0 Hz), 155.9, 145.3, 139.9, 131.2 (d, (d, $^3J_{C\text{-}F}$ = 3.5 Hz), 130.1 (d, (d, $^4J_{C\text{-}F}$ = 5.5 Hz), 129.5, 116.0, 99.9 (d, (d, $^1J_{C\text{-}F}$ = 200.5 Hz), 79.5, 62.8, 45.8, 38.2 (d, (d, $^2J_{C\text{-}F}$ = 20.5 Hz), 28.5, 21.9, 14.1; **^{19}F NMR (377 MHz, $CDCl_3$)**: δ -157.3 (dd, *J* = 33.5, 18.5 Hz); **FTIR:** ν_{max}/cm^{-1} (neat) 2924, 1752, 1701, 1697, 1606, 1507, 1366, 1275, 1166, 1044, 764 cm^{-1}; **HRMS (ESI$^+$):** calculated for $C_{21}H_{28}FNO_5Na$ (ES$^+$)(+Na$^+$): 416.1844. Found: 416.1827.

ethyl 4-{[(*tert*-butoxycarbonyl)amino]methyl}-2-fluoro-2-(4-nitrobenzoyl)pent-4-enoate (3f)

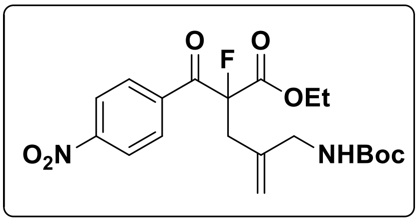

Following GPE with ethyl 2-fluoro-3-(4-nitrophenyl)-3-oxopropanoate (**1f**) (77 mg, 0.3 mmol), *tert*-butyl 5-methylene-2-oxo-1,3-oxazinane-3-carboxylate (43 mg, 0.2 mmol), Pd(dba)$_2$ (6 mg, 0.01 mmol) and *N*,*N*-diisopropyldibenzo[*d*,*f*][1,3,2]dioxaphosphepin-6-amine (10 mg, 0.03 mmol) in CH_2Cl_2 (2 mL) with FCC (20 % ethyl acetate in petroleum ether) afforded ethyl 4-{[(*tert*-butoxycarbonyl)amino]methyl}-2-fluoro-2-(4-nitrobenzoyl)pent-4-enoate (**3f**) as an orange oil (69 mg, 81%).

^{1}H NMR (400 MHz, $CDCl_3$): δ 8.29 (d, *J* = 8.0 Hz, 2H), 8.18 (d, *J* = 8.5 Hz, 2H), 5.15 (s, 1H), 5.03 (s, 1H), 4.74 (br, 1H), 4.32 – 4.19 (m, 1H), 3.75 (d, *J* = 5.0 Hz, 2H), 3.13 (dd, *J* = 32.0, 15.0 Hz, 1H), 2.98 (dd, *J* = 20.0, 15.5 Hz, 1H), 1.43 (s, 9H), 1.23 (t, *J* = 7.0 Hz, 3H); **^{13}C NMR (101 MHz, $CDCl_3$)** δ 190.7 (d, $^2J_{C\text{-}F}$ =

27.0 Hz), 166.3 (d, $^{2}J_{C\text{-}F}$ = 26.0 Hz), 155.9, 150.7, 139.4, 138.4 (d, $^{3}J_{C\text{-}F}$ = 3.5 Hz), 131.0 (d, $^{4}J_{C\text{-}F}$ = 6.0 Hz), 123.9, 116.4, 100.2 (d, $^{1}J_{C\text{-}F}$ = 200.0 Hz), 79.7, 63.3, 45.8, 38.0 (d, $^{2}J_{C\text{-}F}$ = 20.5 Hz), 28.5, 14.1; **^{19}F NMR (377 MHz, CDCl$_3$)**: δ -158.3 (dd, *J* = 32.0, 20.0 Hz).); **FTIR:** ν_{max}/cm^{-1} (neat) 3338, 1704, 1636, 1526, 1349, 1275, 1261, 1169,764, 750 cm^{-1}; **HRMS (ESI$^+$):** calculated for $C_{20}H_{25}FN_2O_7Na$ (ES$^+$)(+Na$^+$): 447.1538. Found: 447.1535.

ethyl 4-{[(*tert*-butoxycarbonyl)amino]methyl}-2-fluoro-2-(2-methylbenzoyl)pent-4-enoate (3g)

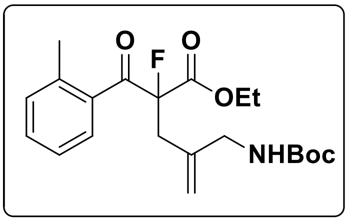

Following GPE with ethyl 2-fluoro-3-(2-methylphenyl)-3-oxopropanoate (**1g**) (67 mg, 0.3 mmol), *tert*-butyl 5-methylene-2-oxo-1,3-oxazinane-3-carboxylate (43 mg, 0.2 mmol), Pd(dba)$_2$ (6 mg, 0.01 mmol) and *N*,*N*-diisopropyldibenzo[*d*,*f*][1,3,2]dioxaphosphepin-6-amine (10 mg, 0.03 mmol) in CH_2Cl_2 (2 mL) with FCC (20 % ethyl acetate in petroleum ether) afforded ethyl 4-{[(*tert*-butoxycarbonyl)amino]methyl}-2-fluoro-2-(2-methylbenzoyl)pent-4-enoate (**3g**) as a colourless oil (67 mg, 86%).

^{1}H NMR (400 MHz, CDCl$_3$): δ 7.67 (dd, *J* = 7.5, 3.0 Hz, 1H), 7.38 (td, *J* = 7.5, 1.0 Hz, 1H), 7.23 (dd, *J* = 15.0, 7.0 Hz, 2H), 5.13 (s, 1H), 5.05 (s, 1H), 4.77 (br, 1H), 4.25 (qd, *J* = 7.0, 2.0 Hz, 2H), 3.73 (d, *J* = 5.5 Hz, 2H), 3.14 – 2.95 (m, 2H), 2.41 (s, 3H), 1.43 (s, 9H), 1.24 (t, *J* = 7.0 Hz, 3H); **^{13}C NMR (101 MHz, CDCl$_3$)** δ 195.4 (d, $^{2}J_{C\text{-}F}$ = 27.0 Hz), 166.8 (d, $^{2}J_{C\text{-}F}$ = 26.0 Hz), 155.9, 139.8, 139.3, 134.3 (d, $^{3}J_{C\text{-}F}$ =2.5 Hz), 132.1, 132.0, 129.0 (d, (d, $^{4}J_{C\text{-}F}$ = 9.0 Hz), 125.5, 116.1, 100.3 (d, $^{1}J_{C\text{-}F}$ = 202.0 Hz), 79.5, 62.9, 45.8, 38.6 (d, $^{2}J_{C\text{-}F}$ = 20.5 Hz), 28.5, 20.9, 14.1; **^{19}F NMR (377 MHz, CDCl$_3$)**: δ -158.1 (dd, *J* = 29.0, 22.0 Hz); **FTIR:** ν_{max}/cm^{-1} (neat) 2980, 2929, 1749, 1704, 1699, 1505, 1367, 1265, 1236, 1167, 1049, 735, 703 cm^{-1}; **HRMS (ESI$^+$):** calculated for $C_{21}H_{28}FNO_5Na$ (ES$^+$)(+Na$^+$): 416.1844. Found: 416.1864.

ethyl 2-(2-naphthoyl)-4-(((*tert*-butoxycarbonyl)amino)methyl)-2-fluoropent-4-enoate (3h)

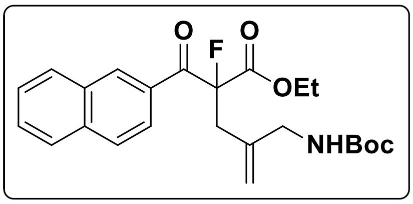

Following GPE using ethyl 2-fluoro-3-(naphthalen-2-yl)-3-oxopropanoate (**1h**) (78 mg, 0.30 mmol), *tert*-butyl 5-methylene-2-oxo-1,3-oxazinane-3-carboxylate (43 mg, 0.20 mmol), Pd(dba)$_2$ (5.8 mg, 10 μmol) and *N*,*N*-diisopropyldibenzo[*d*,*f*][1,3,2]dioxaphosphepin-6-amine (9.5 mg, 30 μmol) with FCC (50-100% DCM in 40-60 petroleum ether) afforded ethyl 2-(2-naphthoyl)-4-(((*tert*-butoxycarbonyl)amino)methyl)-2-fluoropent-4-enoate (**3h**) as a yellow oil (86 mg, quant.).

^{1}H NMR (400 MHz, CDCl$_3$) δ 8.65 (s, 1H), 8.03 (dt, *J* = 8.5, 1.5 Hz, 1H), 7.97 (d, *J* = 8.0 Hz, 1H), 7.87 (t, *J* = 8.5 Hz, 2H), 7.62 (ddd, *J* = 8.0, 7.0, 1.5 Hz, 1H), 7.55 (ddd, *J* = 8.0, 7.0, 1.5 Hz, 1H), 5.16 (s, 1H), 5.07 (s, 1H), 4.79 (br, 1H), 4.34 – 4.15 (m, 2H), 3.80 (br, 2H), 3.29 – 2.97 (m, 2H), 1.44 (s, 9H), 1.20 (t, *J* = 7.0 Hz, 3H); **^{13}C NMR (101 MHz, CDCl$_3$)** δ 191.0 (d, $^{2}J_{C\text{-}F}$ = 25.5 Hz), 167.2 (d, $^{2}J_{C\text{-}F}$ = 26.0 Hz), 155.9, 139.9, 136.0, 132.4 (d, $^{4}J_{C\text{-}F}$ = 8.0 Hz), 131.0 (2C), 130.3, 129.3, 128.6, 127.8, 127.1, 124.9 (d, $^{3}J_{C\text{-}F}$ = 3.0 Hz), 116.1, 100.2 (d, $^{1}J_{C\text{-}F}$ = 200.5 Hz), 62.9, 45.8, 38.3 (d, $^{2}J_{C\text{-}F}$ = 20.5 Hz), 28.5 (3C), 14.1; **^{19}F NMR (376 MHz, CDCl$_3$)** δ -156.8 (dd, *J* = 33.0, 18.5 Hz); **FTIR:** ν_{max}/cm^{-1} (neat) 3416, 2979, 1754, 1691, 1627, 1506, 1366, 1246, 1165, 909 cm^{-1}; **HRMS (ESI$^+$):** calculated for $C_{24}H_{28}FNO_5Na$ (ES$^+$)(+Na$^+$): 452.1844. Found: 452.1859.

ethyl 4-{[(*tert*-butoxycarbonyl)amino]methyl}-2-fluoro-2-(thiophene-2-carbonyl)pent-4-enoate (3i)

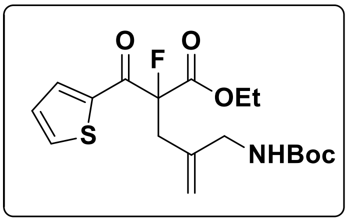

Following GPE with ethyl 2-fluoro-3-oxo-3-(thiophen-2-yl)propanoate (**1i**) (65 mg, 0.3 mmol), *tert*-butyl 5-methylene-2-oxo-1,3-oxazinane-3-carboxylate (43 mg, 0.2 mmol), Pd(dba)$_2$ (6 mg, 0.01 mmol) and *N*,*N*-diisopropyldibenzo[*d*,*f*][1,3,2]dioxaphosphepin-6-amine (10 mg, 0.03 mmol) in CH_2Cl_2 (2 mL) with FCC (20 % ethyl acetate in petroleum ether) afforded ethyl 4-{[(*tert*-butoxycarbonyl)amino]methyl}-2-fluoro-2-(thiophene-2-carbonyl)pent-4-enoate (**3i**) as an orange oil (63 mg, 82%).

^{1}H NMR (400 MHz, CDCl$_3$): δ 8.01 (t, *J* = 3.0 Hz, 1H), 7.73 (d, *J* = 5.0 Hz, 1H), 7.13 (t, *J* = 4.5 Hz, 1H), 5.12 (s, 1H), 5.04 (s, 1H), 4.78 (br, 1H), 4.29 – 4.15 (m, 2H), 3.74 (d, *J* = 5.0 Hz, 2H), 3.11 (dd, *J* = 32.0, 15.5 Hz, 1H), 2.95 (dd, *J* = 20.0, 15.5 Hz, 1H), 1.42 (s, 9H), 1.21 (t, *J* = 7.0 Hz, 3H); **^{13}C NMR (101 MHz, CDCl$_3$)** δ 184.3 (d, $^{2}J_{C-F}$ = 26.5 Hz), 166.5 (d, $^{2}J_{C-F}$ = 26.5 Hz), 155.9, 139.6, 139.5 (d, $^{3}J_{C-F}$ = 3.0 Hz), 136.1 (d, $^{5}J_{C-F}$ = 2.0 Hz), 135.7 (d, $^{4}J_{C-F}$ = 10.5 Hz), 128.8 (d, $^{5}J_{C-F}$ = 1.5 Hz), 116.1, 100.0 (d, $^{1}J_{C-F}$ = 200.5 Hz), 79.5, 62.9, 45.8, 37.8 (d, $^{2}J_{C-F}$ = 20.0 Hz), 28.5, 14.1; **^{19}F NMR (377 MHz, CDCl$_3$)**: δ -158.3 (dd, *J* = 32.0, 20.0 Hz); **FTIR:** ν_{max}/cm^{-1} (neat) 2979, 1756, 1705, 1512, 1410, 1366, 1249, 1169, 1064, 911, 859, 732 cm^{-1}; **HRMS (ESI$^+$):** calculated for $C_{18}H_{24}FNO_5SNa$ (ES$^+$)(+Na$^+$): 408.1251. Found: 408.1251.

tert-butyl (4-cyano-4-fluoro-2-methylene-5-oxo-5-phenylpentyl)carbamate (3j)

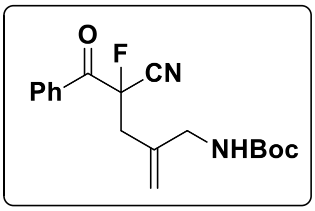

Following GPE with α-fluoro-β-oxo-benzenepropanenitrile (**1j**) (49 mg, 0.3 mmol), *tert*-butyl 5-methylene-2-oxo-1,3-oxazinane-3-carboxylate (43 mg, 0.2 mmol), Pd(dba)$_2$ (6 mg, 0.01 mmol) and *N*,*N*-diisopropyldibenzo[*d*,*f*][1,3,2]dioxaphosphepin-6-amine (10 mg, 0.03 mmol) in CH_2Cl_2 (2 mL) with FCC (20 % ethyl acetate in petroleum ether) afforded *tert*-butyl (4-cyano-4-fluoro-2-methylene-5-oxo-5-phenylpentyl)carbamate (**3j**) as a colourless oil (58 mg, 87%).

^{1}H NMR (400 MHz, CDCl$_3$) δ 8.08 (d, *J* = 8.0 Hz, 2H), 7.67 (t, *J* = 7.5 Hz, 1H), 7.52 (t, *J* = 8.0 Hz, 2H), 5.33 (s, 1H), 5.23 (s, 1H), 4.79 (br, 1H), 3.90 – 3.73 (m, 2H), 3.13 – 2.93 (m, 2H), 1.44 (s, 9H); **^{13}C NMR (101 MHz, CDCl$_3$)** δ 188.6 (d, $^{2}J_{C-F}$ = 24.5 Hz), 155.9, 137.8, 135.1, 131.9 (d, $^{3}J_{C-F}$ = 3.5 Hz), 130.3 (d, $^{4}J_{C-F}$ = 5.5 Hz), 129.1, 118.6, 114.7 (d, $^{2}J_{C-F}$ = 34.0 Hz), 92.9 (d, $^{1}J_{C-F}$ = 200.0 Hz), 79.9, 45.8, 40.2 (d, $^{2}J_{C-F}$ = 22.0 Hz), 28.5; **^{19}F NMR (377 MHz, CDCl$_3$)**: δ -152.4 (dd, *J* = 30.0, 18.0 Hz); **FTIR:** ν_{max}/cm^{-1} (neat) 2986, 1758, 1715, 1638, 1409, 1325, 1314, 1124, 1115, 1066, 849, 701 cm^{-1}; **HRMS (ESI$^+$):** calculated for $C_{18}H_{21}FN_2O_3Na$ (ES$^+$)(+Na$^+$): 355.1428. Found: 355.1443.

tert-butyl (4-fluoro-2-methylene-5-oxo-5-phenyl-4-tosylpentyl)carbamate (3k)

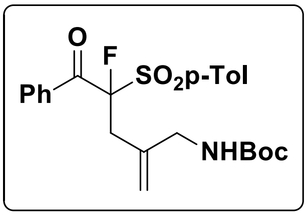

Following GPE using 2-fluoro-1-phenyl-2-(phenylsulfonyl)ethanone (**1k**) (88 mg, 0.3 mmol), *tert*-butyl 5-methylene-2-oxo-1,3-oxazinane-3-carboxylate (43 mg, 0.2 mmol), Pd(dba)$_2$ (6 mg, 0.01 mmol) and *N*,*N*-diisopropyldibenzo[*d*,*f*][1,3,2]dioxaphosphepin-6-amine (10 mg, 0.03 mmol) in CH_2Cl_2 (2 mL) with FCC (15 % ethyl acetate in petroleum ether) afforded *tert*-butyl (4-fluoro-2-methylene-5-oxo-5-phenyl-4-tosylpentyl)carbamate (**3k**) as a yellow oil (91 mg, 98%).

^{1}H NMR (400 MHz, CDCl$_3$) δ 7.79 – 7.69 (m, 4H), 7.49 (t, *J* = 7.5 Hz, 1H), 7.36 – 7.27 (m, 4H), 5.03 (s, 1H), 4.92 (s, 1H), 4.67 (br, 1H), 3.61 – 3.53 (m, 2H), 3.45 (dd, *J* = 41.0, 15.0 Hz, 1H), 2.80 (dd, *J* = 14.5, 10.0 Hz, 1H), 2.40 (s, 3H), 1.38 (s, 9H); **^{13}C NMR (101 MHz, CDCl$_3$)** δ 193.0 (d, $^{2}J_{C-F}$ = 24.5 Hz), 155.7, 146.7, 137.9, 135.5 (d, $^{3}J_{C-F}$ = 4.0 Hz), 133.7, 131.3, 130.8, 130.0, 129.8 (d, $^{4}J_{C-F}$ = 8.0 Hz), 128.3, 116.9, 112.9 (d, $^{1}J_{C-F}$ = 240.0 Hz), 79.6, 45.8, 36.1 (d, $^{2}J_{C-F}$ = 19.0 Hz), 28.4, 21.8; **^{19}F NMR (377 MHz, CDCl$_3$)** δ -

154.0 – -154.3 (m); **FTIR:** ν_{max}/cm^{-1} (neat) 3356, 2977, 1686, 1651, 1523, 1334, 1288, 1249, 1150, 1072 cm^{-1}; **HRMS (ESI⁺):** calculated for $C_{24}H_{28}FNO_5SNa$ $(ES^+)(+Na^+)$: 484.1564. Found: 484.1582.

tert-butyl (4-benzoyl-4-fluoro-5-(methoxy(methyl)amino)-2-methylene-5-oxopentyl)carbamate (3l)

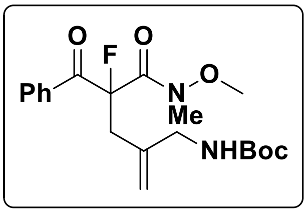

Following GPE using 2-fluoro-*N*-methoxy-*N*-methyl-3-oxo-3-phenylpropanamide (**1l**) (68 mg, 0.3 mmol), *tert*-butyl 5-methylene-2-oxo-1,3-oxazinane-3-carboxylate (43 mg, 0.2 mmol), $Pd(dba)_2$ (6 mg, 0.01 mmol) and *N*,*N*-diisopropyldibenzo[*d*,*f*][1,3,2]dioxaphosphepin-6-amine (10 mg, 0.03 mmol) in CH_2Cl_2 (2 mL) with FCC (15 % ethyl acetate in petroleum ether) afforded *tert*-butyl (4-benzoyl-4-fluoro-5-(methoxy(methyl)amino)-2-methylene-5-oxopentyl)carbamate (**3l**) as a yellow oil (71 mg, 90%).

^{1}H NMR (400 MHz, CDCl$_3$) δ 7.99 (d, *J* = 8.5 Hz, 2H), 7.60 – 7.53 (m, 1H), 7.49 – 7.41 (m, 2H), 5.07 (s, 1H), 4.91 (s, 1H), 4.83 (br, 1H), 3.76 (ddd, *J* = 21.5, 16.5, 6.0 Hz, 2H), 3.42 (s, 3H), 3.20 – 3.07 (m, 4H), 2.98 (dd, *J* = 27.0, 15.0 Hz, 1H), 1.43 (s, 9H); **^{13}C NMR (101 MHz, CDCl$_3$)** δ 192.3 (d, $^2J_{C\text{-}F}$ = 24.5 Hz), 156.0, 139.9, 134.5 (d, $^3J_{C\text{-}F}$ = 3.0 Hz), 133.6, 129.5 (d, $^4J_{C\text{-}F}$ = 5.5 Hz), 128.7, 116.7, 100.2 (d, $^1J_{C\text{-}F}$ = 198.0 Hz), 79.4, 61.4, 45.8, 38.4 (d, $^2J_{C\text{-}F}$ = 21.0 Hz), 33.3, 28.5; **^{19}F NMR (376 MHz, CDCl$_3$)** δ -154.4 – -154.7 (m); **FTIR:** ν_{max}/cm^{-1} (neat) 3364, 2978, 2939, 1682, 1509, 1448, 1365, 1246, 1167, 986 cm^{-1}; **HRMS (ESI⁺):** calculated for $C_{20}H_{27}FN_2O_5Na$ $(ES^+)(+Na^+)$: 417.1796. Found: 417.1808.

ethyl 2-acetyl-4-{[(*tert*-butoxycarbonyl)amino]methyl}-2-fluoropent-4-enoate (3m)

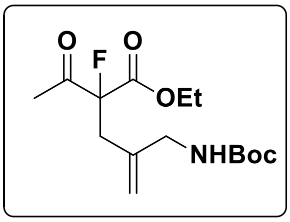

Following GPE with ethyl 2-fluoro-3-oxobutanoate (**1m**) (44 mg, 0.3 mmol), *tert*-butyl 5-methylene-2-oxo-1,3-oxazinane-3-carboxylate (43 mg, 0.2 mmol), $Pd(dba)_2$ (6 mg, 0.01 mmol) and *N*,*N*-diisopropyldibenzo[*d*,*f*][1,3,2]dioxaphosphepin-6-amine (10 mg, 0.03 mmol) in CH_2Cl_2 (2 mL) with FCC (15 % ethyl acetate in petroleum ether) afforded ethyl 2-acetyl-4-{[(*tert*-butoxycarbonyl)amino]methyl}-2-fluoropent-4-enoate (**3m**) as a colourless oil (56 mg, 88%).

^{1}H NMR (400 MHz, CDCl$_3$): δ 5.10 (s, 1H), 4.99 (s, 1H), 4.73 (br, 1H), 4.24 (q, *J* = 7.0 Hz, 2H), 3.77 – 3.62 (m, 2H), 2.94 – 2.73 (m, 2H), 2.29 (d, *J* = 5.0 Hz, 3H), 1.43 (s, 9H), 1.28 (t, *J* = 7.0 Hz, 3H); **^{13}C NMR (101 MHz, CDCl$_3$)** δ 201.7 (d, $^2J_{C\text{-}F}$ = 29.5 Hz), 165.8 (d, $^2J_{C\text{-}F}$ = 25.5 Hz), 155.9, 139.5, 115.8, 100.4 (d, $^1J_{C\text{-}F}$ = 200.0 Hz), 79.6, 62.9, 45.8, 37.4 (d, $^2J_{C\text{-}F}$ = 20.0 Hz), 28.5, 26.0, 14.1; **^{19}F NMR (377 MHz, CDCl$_3$):** δ -163.6 – -163.7 (m); **FTIR:** ν_{max}/cm^{-1} (neat) 2979, 1755, 1716, 1514, 1367, 1248, 1169, 861 cm^{-1}; **HRMS (ESI⁺):** calculated for $C_{15}H_{24}FNO_5Na$ $(ES^+)(+Na^+)$: 340.1531 Found: 340.1541.

ethyl 4-({[(*tert*-butoxy)carbonyl]amino}methyl)-2-fluoro-2-propanoylpent-4-enoate (3n)

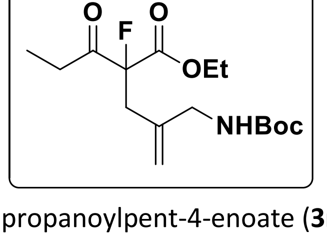

Following GPE with ethyl 2-fluoro-3-oxopentanoate (**1n**) (49 mg, 0.3 mmol), *tert*-butyl 5-methylene-2-oxo-1,3-oxazinane-3-carboxylate (43 mg, 0.2 mmol), $Pd(dba)_2$ (6 mg, 0.01 mmol) and *N*,*N*-diisopropyldibenzo[*d*,*f*][1,3,2]dioxaphosphepin-6-amine (10 mg, 0.03 mmol) in CH_2Cl_2 (2 mL) with FCC (15 % ethyl acetate in petroleum ether) afforded ethyl 4-({[(*tert*-butoxy)carbonyl]amino}methyl)-2-fluoro-2-propanoylpent-4-enoate (**3n**) as a pale yellow oil (55 mg, 83%).

^{1}H NMR (400 MHz, CDCl$_3$): δ 5.09 (s, 1H), 4.98 (s, 1H), 4.72 (br, 1H), 4.23 (q, *J* = 7.0 Hz, 2H), 3.73 – 3.62 (m, 2H), 3.02 – 2.72 (m, 2H), 2.68 – 2.58 (m, 2H), 1.43 (s, 9H), 1.27 (t, *J* = 7.0 Hz, 3H), 1.05 (t, *J* = 7.0 Hz, 3H); **^{13}C NMR (101 MHz, CDCl$_3$)** δ 204.5 (d, *J* = 27.0 Hz), 166.0 (d, *J* = 25.5 Hz), 155.9, 139.6, 115.8, 100.6 (d, *J* = 199.5 Hz), 79.6, 62.8, 45.8, 37.6 (d, *J* = 20.0 Hz), 31.7, 28.5, 14.1, 7.1 (d, *J* = 2.0 Hz); **^{19}F NMR (376 MHz, CDCl$_3$)**: δ -165.8 – -166.1 (m); **FTIR:** ν_{max}/cm^{-1} (neat) 2981, 1755, 1717, 1514, 1275, 1268, 1169, 764, 750 cm^{-1}; **HRMS (ESI$^+$):** calculated for $C_{16}H_{26}FNO_5Na$ (ES$^+$)(+Na$^+$): 354.1687 Found: 354.1680.

ethyl 4-({[(*tert*-butoxy)carbonyl]amino}methyl)-2-fluoro-2-(2-methylpropanoyl)pent-4-enoate (3o)

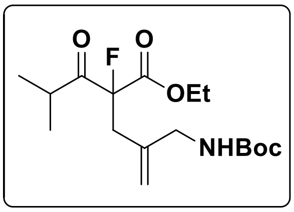

Following GPE with ethyl 2-fluoro-4-methyl-3-oxopentanoate (**1o**) (53 mg, 0.3 mmol), *tert*-butyl 5-methylene-2-oxo-1,3-oxazinane-3-carboxylate (43 mg, 0.2 mmol), Pd(dba)$_2$ (6 mg, 0.01 mmol) and *N,N*-diisopropyldibenzo[*d,f*][1,3,2]dioxaphosphepin-6-amine (10 mg, 0.03 mmol) in CH_2Cl_2 (2 mL) with FCC (15 % ethyl acetate in petroleum ether) afforded ethyl 4-({[(*tert*-butoxy)carbonyl]amino}methyl)-2-fluoro-2-(2-methylpropanoyl)pent-4-enoate (**3o**) as a colourless oil (63 mg, 92%).

^{1}H NMR (400 MHz, CDCl$_3$) δ 5.09 (s, 1H), 4.98 (s, 1H), 4.72 (br, 1H), 4.24 (q, *J* = 7.0 Hz, 2H), 3.78 – 3.61 (m, 2H), 3.20 – 3.07 (m, 1H), 2.90 (dd, *J* = 27.5, 15.0 Hz, 1H), 2.78 (dd, *J* = 23.5, 15.0 Hz, 1H), 1.43 (s, 9H), 1.27 (t, *J* = 7.0 Hz, 3H), 1.12 – 1.05 (m, 6H); **^{13}C NMR (101 MHz, CDCl$_3$)**: δ 204.8 (d, $^2J_{C\text{-}F}$ = 26.0 Hz), 166.0 (d, $^2J_{C\text{-}F}$ = 25.5 Hz), 155.8, 139.5, 115.8, 100.7 (d, $^1J_{C\text{-}F}$ = 201.0 Hz), 79.4, 62.7, 45.7, 37.9 (d, $^2J_{C\text{-}F}$ = 20.0 Hz), 36.3, 28.4, 18.5, 17.8, 14.0; **^{19}F NMR (376 MHz, CDCl$_3$)**: δ -167.1 – -167.4 (m); **FTIR:** ν_{max}/cm^{-1} (neat) 2977, 1753, 1716, 1511, 1366, 1247, 1166, 1097, 1042, 909, 859 cm^{-1}; **HRMS (ESI$^+$):** calculated for $C_{17}H_{28}FNO_5Na$ (ES$^+$)(+Na$^+$): 368.1844. Found: 368.1829.

ethyl 4-{[(*tert*-butoxycarbonyl)amino]methyl}-2-(2,2-dimethylpropanoyl)-2-fluoropent-4-enoate (3p)

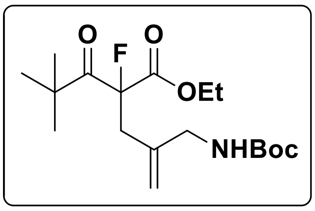

Following GPE with ethyl 2-fluoro-3-(2,2-dimethylpropanoyl)-3-oxopropanoate (**1p**) (57 mg, 0.3 mmol), *tert*-butyl 5-methylene-2-oxo-1,3-oxazinane-3-carboxylate (43 mg, 0.2 mmol), Pd(dba)$_2$ (6 mg, 0.01 mmol) and *N,N*-diisopropyldibenzo[*d,f*][1,3,2]dioxaphosphepin-6-amine (10 mg, 0.03 mmol) in CH_2Cl_2 (2 mL) with FCC (20 % ethyl acetate in petroleum ether) afforded ethyl 4-{[(*tert*-butoxycarbonyl)amino]methyl}-2-(2,2dimethylpropanoyl)-2-fluoropent-4-enoate (**3p**) as a colourless oil (58 mg, 81%).

^{1}H NMR (400 MHz, CDCl$_3$): δ 5.09 (s, 1H), 4.99 (s, 1H), 4.74 (br, 1H), 4.23 (q, *J* = 7.0 Hz, 2H), 3.70 (d, *J* = 5.5 Hz, 2H), 2.93 (dd, *J* = 29.5, 15.0 Hz, 1H), 2.77 (dd, *J* = 21.5, 15.0 Hz, 1H), 1.43 (s, 9H), 1.27 (t, *J* = 7.0 Hz, 3H), 1.20 (d, *J* = 1.5 Hz, 9H); **^{13}C NMR (101 MHz, CDCl$_3$)** δ 206.7 (d, $^2J_{C\text{-}F}$ = 25.5 Hz), 166.4 (d, $^2J_{C\text{-}F}$ = 25.5 Hz), 155.9, 139.9, 116.1, 102.1 (d, $^1J_{C\text{-}F}$ = 204.5 Hz), 79.5, 62.7, 45.8, 45.4 (d, $^3J_{C\text{-}F}$ = 3.5 Hz), 39.3 (d, $^2J_{C\text{-}F}$ = 20.5 Hz), 28.5, 26.4 (d, $^4J_{C\text{-}F}$ = 4.5 Hz), 14.2; **^{19}F NMR (377 MHz, CDCl$_3$)**: δ -164.6 (dd, *J* = 28.5, 22.0 Hz); **FTIR:** ν_{max}/cm^{-1} (neat) 2928, 1716, 1696, 1576, 1367, 1275, 1260, 764 cm^{-1}; **HRMS (ESI$^+$):** calculated for $C_{18}H_{30}FNO_5Na$ (ES$^+$)(+Na$^+$): 382.2000 Found: 382.2014.

ethyl 4-({[(*tert*-butoxy)carbonyl]amino}methyl)-2-cyclohexanecarbonyl-2-fluoropent-4-enoate (3q)

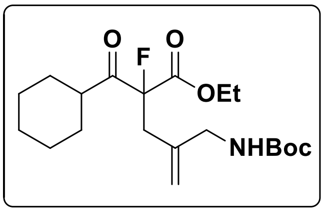

Following GPE with ethyl 3-cyclohexyl-2-fluoro-3-oxopropanoate (**1q**) (65 mg, 0.3 mmol), *tert*-butyl 5-methylene-2-oxo-1,3-oxazinane-3-carboxylate (43 mg, 0.2 mmol), Pd(dba)$_2$ (6 mg, 0.01 mmol) and *N*,*N*-diisopropyldibenzo[*d*,*f*][1,3,2]dioxaphosphepin-6-amine (10 mg, 0.03 mmol) in CH_2Cl_2 (2 mL) with FCC (15 % ethyl acetate in petroleum ether) afforded ethyl 4-({[(*tert*-butoxy)carbonyl]amino}methyl)-2-cyclohexanecarbonyl-2-fluoropent-4-enoate (**3q**) as a colourless oil (62 mg, 81%).

^{1}H NMR (400 MHz, CDCl$_3$): δ 5.08 (s, 1H), 4.97 (s, 1H), 4.72 (br, 1H), 4.22 (q, *J* = 7.0 Hz, 2H), 3.69 (s, 2H), 2.96 – 2.67 (m, 3H), 1.86 – 1.62 (m, 6H), 1.42 (s, 9H), 1.35 – 1.20 (m, 7H); **^{13}C NMR (101 MHz, CDCl$_3$)** δ 206.4 (d, $^2J_{C-F}$ = 25.0 Hz), 166.1 (d, $^2J_{C-F}$ = 25.5 Hz), 155.9, 139.7, 115.9, 100.8 (d, $^1J_{C-F}$ = 200.5 Hz), 79.6, 62.7, 46.1, 45.8, 37.9 (d, $^2J_{C-F}$ = 20.5 Hz), 28.8 (d, $^5J_{C-F}$ = 1.0 Hz), 28.5, 27.9 (d, $^5J_{C-F}$ = 1.0 Hz), 25.7 (d, $^4J_{C-F}$ = 4.5 Hz), 25.3, 14.2; **^{19}F NMR (376 MHz, CDCl$_3$)**: δ -167.1 – -167.4 (m); **FTIR:** ν_{max}/cm^{-1} (neat) 2929, 1754, 1718, 1507, 1367, 1244, 1172 cm^{-1}; **HRMS (ESI$^+$):** calculated for $C_{20}H_{32}FNO_5Na$ (ES$^+$)(+Na$^+$): 408.2157 Found: 408.2142.

ethyl 2-(2-(((*tert*-butoxycarbonyl)amino)methyl)allyl)-2-fluoro-3-oxooct-7-enoate (3r)

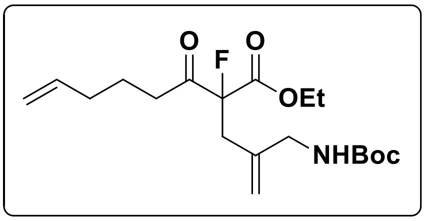

Following GPE using ethyl 2-fluoro-3-oxooct-7-enoate (**1r**) (61 mg, 0.3 mmol), *tert*-butyl 5-methylene-2-oxo-1,3-oxazinane-3-carboxylate (43 mg, 0.2 mmol), Pd(dba)$_2$ (6 mg, 0.01 mmol) and *N*,*N*-diisopropyldibenzo[*d*,*f*][1,3,2]dioxaphosphepin-6-amine (10 mg, 0.03 mmol) in CH_2Cl_2 (2 mL) with FCC (15 % ethyl acetate in petroleum ether) afforded ethyl 2-(2-(((*tert*-butoxycarbonyl)amino)methyl)allyl)-2-fluoro-3-oxooct-7-enoate (**3r**) as a yellow oil (70 mg, 94%).

^{1}H NMR (400 MHz, CDCl$_3$) δ 5.74 (ddt, *J* = 17.0, 10.0, 6.5 Hz, 1H), 5.10 (s, 1H), 5.04 – 4.95 (m, 3H), 4.71 (s, 1H), 4.24 (q, *J* = 7.0 Hz, 2H), 3.78 – 3.61 (m, 2H), 2.90 (dd, *J* = 27.0, 15.5 Hz, 1H), 2.78 (dd, *J* = 24.0, 15.5 Hz, 1H), 2.68 – 2.62 (m, 2H), 2.05 (q, *J* = 7.5 Hz, 2H), 1.72 – 1.64 (m, 2H), 1.44 (s, 9H), 1.28 (t, *J* = 7.0 Hz, 3H); **^{13}C NMR (101 MHz, CDCl$_3$)** δ 203.6 (d, $^2J_{C-F}$ = 28.0 Hz), 165.9 (d, $^2J_{C-F}$ = 25.5 Hz), 155.8, 139.5, 137.7, 115.7, 115.5, 100.5 (d, $^1J_{C-F}$ = 200.0 Hz), 79.5, 62.8, 45.8, 37.5 (d, $^2J_{C-F}$ = 20.0 Hz), 37.3, 32.8, 28.4, 21.9 (d, $^3J_{C-F}$ = 2.0 Hz), 14.1; **^{19}F NMR (376 MHz, CDCl$_3$)** δ -165.5 – -165.8 (m); **FTIR:** ν_{max}/cm^{-1} (neat) 3411, 2978, 2933, 1754, 1716, 1510, 1366, 1245, 1165, 1048 cm^{-1}; **HRMS (ESI$^+$):** calculated for $C_{19}H_{30}FNO_5Na$ (ES$^+$)(+Na$^+$): 394.2000. Found: 394.1991.

ethyl 4-(((*tert*-butoxycarbonyl)amino)methyl)-2-fluoro-2-(1-tosylpyrrolidine-2-carbonyl)pent-4-enoate (3s)

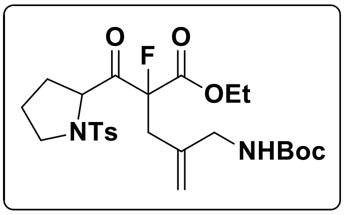

Following GPE using ethyl 2-fluoro-3-oxo-3-(1-tosylpyrrolidin-2-yl)propanoate (**1s**) (0.11g, 0.3 mmol), *tert*-butyl 5-methylene-2-oxo-1,3-oxazinane-3-carboxylate (43 mg, 0.2 mmol), Pd(dba)$_2$ (6 mg, 0.01 mmol) and *N*,*N*-diisopropyldibenzo[*d*,*f*][1,3,2]dioxaphosphepin-6-amine (10 mg, 0.03 mmol) in CH_2Cl_2 (2 mL) with FCC (15 % ethyl acetate in petroleum ether) afforded ethyl 4-(((*tert*-butoxycarbonyl)amino)methyl)-2-fluoro-2-(1-tosylpyrrolidine-2-carbonyl)pent-4-enoate (**3s**) as a yellow oil (76 mg, 72%)(4.2:1 mixture of diastereoisomers).

<u>**Major Isomer**</u>

^{1}H NMR (400 MHz, CDCl$_3$) δ 7.76 – 7.65 (m, 2H), 7.35 – 7.29 (m, 2H), 5.11 (s, 1H), 5.04 (s, 1H), 4.78 (ddd, *J* = 9.0, 4.5, 2.5 Hz, 1H), 4.71 (br, 1H), 4.39 – 4.23 (m, 2H), 3.80 – 3.62 (m, 2H), 3.50 – 3.19 (m,

2H), 3.09 – 2.77 (m, 2H), 2.42 (s, 3H), 2.08 – 1.99 (m, 1H), 1.90 – 1.74 (m, 2H), 1.72 – 1.65 (m, 1H), 1.47 – 1.40 (m, 9H), 1.35 – 1.29 (m, 3H); **^{13}C NMR (101 MHz, $CDCl_3$)** δ 200.1 (d, $^2J_{C-F}$ = 27.5 Hz), 165.1 (d, $^2J_{C-F}$ = 26.0 Hz), 155.8, 143.8, 139.6, 134.8, 129.8, 127.5, 115.8, 101.3 (d, $^1J_{C-F}$ = 196.5 Hz), 79.5, 63.5, 62.2, 48.6, 45.9, 37.0 (d, $^2J_{C-F}$ = 19.5 Hz), 29.8, 28.4, 24.8, 21.6, 14.0.

Minor Isomer

^{1}H NMR (400 MHz, $CDCl_3$) δ 7.76 – 7.65 (m, 2H), 7.35 – 7.29 (m, 2H), 5.18 (s, 1H), 5.11 (s, 1H), 4.91 (br, 1H), 4.88 (ddd, *J* = 9.0, 4.5, 3.0 Hz, 1H), 4.39 – 4.23 (m, 2H), 3.80 – 3.62 (m, 2H), 3.50 – 3.19 (m, 2H), 3.09 – 2.77 (m, 2H), 2.42 (s, 3H), 2.08 – 1.99 (m, 1H), 1.90 – 1.74 (m, 2H), 1.72 – 1.65 (m, 1H), 1.47 – 1.40 (m, 9H), 1.35 – 1.29 (m, 3H); **^{13}C NMR (101 MHz, $CDCl_3$)** δ 200.1 (d, $^2J_{C-F}$ = 27.5 Hz), 165.1 (d, $^2J_{C-F}$ = 26.0 Hz), 156.0, 143.9, 138.7, 135.2, 129.8, 127.6, 116.1, 100.5 (d, $^1J_{C-F}$ = 200.5 Hz), 79.5, 63.8, 63.1, 48.5, 45.8, 38.1 (d, $^2J_{C-F}$ = 20.0 Hz), 29.5, 28.4, 24.5, 22.6, 14.4.

^{19}F NMR (376 MHz, $CDCl_3$) δ -168.2 – -168.5 (m); **FTIR:** ν_{max}/cm^{-1} (neat) 3405, 2979, 2932, 1745, 1704, 1509, 1448, 1349, 1248, 1157, 1097 cm^{-1}; **HRMS (ESI$^+$):** calculated for $C_{25}H_{35}FN_2O_7SNa$ (ES$^+$)(+Na$^+$): 549.2041. Found: 549.2048.

tert-butyl (2-methylene-5-oxo-5-phenyl-4-((trifluoromethyl)thio)pentyl)carbamate (12a)

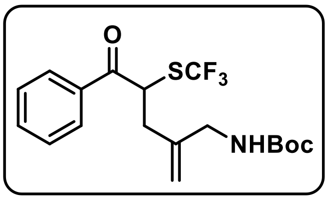

Following GPE with 1-phenyl-2-((trifluoromethyl)thio)ethan-1-one (155 mg, 0.703 mmol), *tert*-butyl 5-methylene-2-oxo-1,3-oxazinane-3-carboxylate (100 mg, 0.469 mmol), Pd(dba)$_2$ (13 mg, 0.02 mmol) and *N*,*N*-diisopropyldibenzo[*d*,*f*][1,3,2]dioxaphosphepin-6-amine (22 mg, 0.07 mmol) in CH_2Cl_2 (4.7 mL) with FCC (5 % EtOAc, 30% CH_2Cl_2 in 40-60 petroleum ether) gave *tert*-butyl (2-methylene-5-oxo-5-phenyl-4-((trifluoromethyl)thio)pentyl)carbamate (**12a**) as a yellow oil (171 mg, 93%).

^{1}H NMR (400 MHz, $CDCl_3$): δ 8.02 (d, *J* = 7.5 Hz, 2H), 7.62 (t, *J* = 7.5 Hz, 1H), 7.51 (t, *J* = 7.5 Hz, 2H), 5.15 (t, *J* = 7.0 Hz, 1H), 5.01 (s, 1H), 4.91 (s, 1H), 4.63 (br, 1H), 3.80 - 3.69 (m, 2H), 2.96 (dd, *J* = 14.5 Hz, 8.0 Hz, 1H), 2.66 (dd, *J* = 14.5 Hz, 7.0 Hz, 1H), 1.43 (s, 9H); **^{13}C NMR (101 MHz, $CDCl_3$)** δ 194.3, 155.8, 141.7, 137.6, 133.8, 133.2 (q, $^1J_{C-F}$ = 309 Hz), 128.8, 128.6, 115.5, 79.7, 44.9, 44.6, 36.1, 28.2 ; **^{19}F NMR (377 MHz, $CDCl_3$)**: δ -39.4 (s); **FTIR:** ν_{max}/cm^{-1} (neat) 3375, 2982, 2932, 1687, 1510, 1449, 1367, 1275, 1260, 1156, 1107, 750, 688; **HRMS (ESI$^+$):** *m/z* calcd. for $C_{18}H_{22}F_3NO_3SNa$: 412.1165, found: 412.1177 [*M+Na*]$^+$.

tert-butyl (5-(4-methoxyphenyl)-2-methylene-5-oxo-4-((trifluoromethyl)thio)pentyl) carbamate (12b)

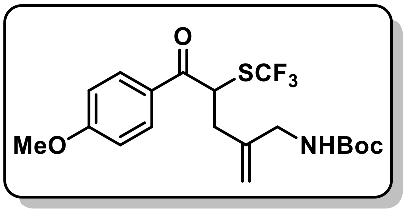

Following GPE with 1-(4-methoxyphenyl)-2-((trifluoromethyl)thio)ethan-1-one (176 mg, 0.703 mmol), *tert*-butyl 5-methylene-2-oxo-1,3-oxazinane-3-carboxylate (100 mg, 0.469 mmol), Pd(dba)$_2$ (13 mg, 0.02 mmol) and *N*,*N*-diisopropyldibenzo[*d*,*f*][1,3,2]dioxaphosphepin-6-amine (22 mg, 0.07 mmol) in CH_2Cl_2 (4.7 mL) with FCC (5 % EtOAc, 30% CH_2Cl_2 in 40-60 petroleum ether) gave *tert*-butyl (5-(4-methoxyphenyl)-2-methylene-5-oxo-4-((trifluoromethyl)thio)pentyl) carbamate (**12b**) as a yellow oil (178 mg, 90%).

^{1}H NMR (400 MHz, $CDCl_3$): δ 8.01 (d, *J* = 9.0 Hz, 2H), 6.97 (d, *J* = 9.0 Hz, 2H), 5.10 (t, *J* = 7.0 Hz, 1H), 4.99 (s, 1H), 4.90 (s, 1H), 4.63 (br, 1H), 3.89 (s, 3H), 3.80 - 3.69 (m, 2H), 2.94 (dd, *J* = 14.5 Hz, 8.5 Hz, 1H), 2.64 (dd, *J* = 14.5 Hz, 7.0 Hz, 1H), 1.43 (s, 9H); **^{13}C NMR (101 MHz, $CDCl_3$)** δ 194.4, 164.3, 155.9, 141.9, 131.2, 130.5 (q, $^1J_{C-F}$ = 308 Hz), 127.8, 115.6, 114.2, 79.7, 55.6, 44.8, 44.7, 36.6, 28.3; **^{19}F NMR**

(377 MHz, CDCl$_3$): δ -39.5 (s); **FTIR:** ν_{max}/cm^{-1} (neat) 3386, 2983, 2939, 1676, 1600, 1575, 1512, 1417, 1367, 1257, 1156, 1106, 1027, 759, 601; **HRMS (ESI$^+$):** *m/z* calcd. for $C_{19}H_{24}F_3NO_4SNa$: 442.1270, found: 442.1290 [*M+Na*]$^+$.

tert-butyl (2-methylene-5-oxo-5-(p-tolyl)-4-((trifluoromethyl)thio)pentyl)carbamate (12c)

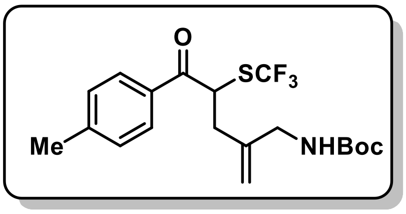

Following an adapted GPE with 1-(p-tolyl)-2-((trifluoromethyl)thio)ethan-1-one (165 mg, 0.703 mmol), *tert*-butyl 5-methylene-2-oxo-1,3-oxazinane-3-carboxylate (100 mg, 0.469 mmol), Pd(dba)$_2$ (13 mg, 0.02 mmol) and *N*,*N*-diisopropyldibenzo[*d*,*f*][1,3,2]dioxaphosphepin-6-amine (22 mg, 0.07 mmol) in CH_2Cl_2 (4.7 mL). Reaction mixture was stirred and heated to reflux (DrySyn block) for 18 h. Solvent was then evaporated under reduced pressure and crude material was subjected to silica gel FCC (5 % EtOAc, 30% CH_2Cl_2 in 40-60 petroleum ether) to give *tert*-butyl (2-methylene-5-oxo-5-(p-tolyl)-4-((trifluoromethyl)thio)pentyl)carbamate (**12c**) as a yellow oil (155 mg, 82%).

^{1}H NMR (400 MHz, CDCl$_3$): δ 7.91 (d, *J* = 8.0 Hz, 2H), 7.30 (d, *J* = 8.0 Hz, 2H), 5.09 - 5.13 (m, 1H), 5.00 (s, 1H), 4.90 (s, 1H), 4.62 (br, 1H), 3.80 - 3.69 (m, 2H), 2.94 (dd, *J* = 14.5 Hz, 8.5 Hz, 1H), 2.65 (dd, *J* = 14.5 Hz, 7.0 Hz, 1H), 2.43 (s, 3H), 1.43 (s, 9H); **^{13}C NMR (101 MHz, CDCl$_3$)** δ 195.5, 155.9, 145.2, 141.9, 132.4, 130.4 (q, $^1J_{C-F}$ = 308 Hz), 129.7, 128.9, 115.6, 79.7, 45.0, 44.8, 36.5, 28.3, 21.8; **^{19}F NMR (377 MHz, CDCl$_3$)**: δ -39.4 (s); **FTIR:** ν_{max}/cm^{-1} (neat) 3367, 2983, 2926, 1681, 1606, 1509, 1367, 1272, 1257, 1153, 1109, 1046, 762, 750, 595; **HRMS (ESI$^+$):** *m/z* calcd. for $C_{19}H_{24}F_3NO_3SNa$: 426.1321, found: 426.1333 [*M+Na*]$^+$.

tert-butyl (5-(4-chlorophenyl)-2-methylene-5-oxo-4-((trifluoromethyl)thio)pentyl) carbamate (12d)

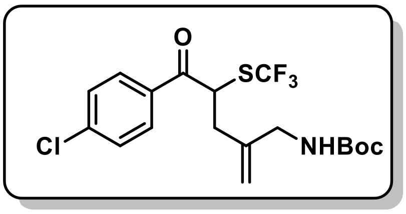

Following GPE with 1-(4-chlorophenyl)-2-((trifluoromethyl)thio) ethan-1-one (179 mg, 0.703 mmol), *tert*-butyl 5-methylene-2-oxo-1,3-oxazinane-3-carboxylate (100 mg, 0.469 mmol), Pd(dba)$_2$ (13 mg, 0.02 mmol) and *N*,*N*-diisopropyldibenzo[*d*,*f*][1,3,2]dioxaphosphepin-6-amine (22 mg, 0.07 mmol) in CH_2Cl_2 (4.7 mL) with FCC (5 % EtOAc, 30% CH_2Cl_2 in 40-60 petroleum ether) gave *tert*-butyl (5-(4-chlorophenyl)-2-methylene-5-oxo-4-((trifluoromethyl)thio)pentyl)carbamate (**12d**) as a yellow oil (189 mg, 95%).

^{1}H NMR (400 MHz, CDCl$_3$): δ 7.97 (d, *J* = 8.5 Hz, 2H), 7.48 (d, *J* = 8.5 Hz, 2H), 5.12 (t, *J* = 7.0 Hz, 1H), 5.01 (s, 1H), 4.90 (s, 1H), 4.64 (br, 1H), 3.80 (dd, *J* = 16.0 Hz, 6.0 Hz, 1H), 3.70 (dd, *J* = 16.0 Hz, 6.0 Hz, 1H), 2.93 (dd, *J* = 14.5 Hz, 8.0 Hz, 1H), 2.64 (dd, *J* = 14.5 Hz, 7.0 Hz, 1H), 1.42 (s, 9H); **^{13}C NMR (101 MHz, CDCl$_3$)** δ 194.9, 155.9, 141.8, 140.6, 133.3, 130.3 (q, $^1J_{C-F}$ = 308 Hz), 130.2, 129.3, 116.1, 79.8, 44.8, 44.7, 36.2, 28.3; **^{19}F NMR (377 MHz, CDCl$_3$)**: δ -39.3 (s); **FTIR:** ν_{max}/cm^{-1} (neat) 3363, 2981, 2930, 1688, 1590, 1508, 1367, 1275, 1260, 1157, 1108, 1050, 764, 751, 532; **HRMS (ESI$^+$):** *m/z* calcd. for $C_{18}H_{21}{}^{35}ClF_3NO_3SNa$: 446.0775, found: 446.0790 [*M+Na*]$^+$.

tert-butyl (5-(2-methoxyphenyl)-2-methylene-5-oxo-4-((trifluoromethyl)thio)pentyl) carbamate (12e)

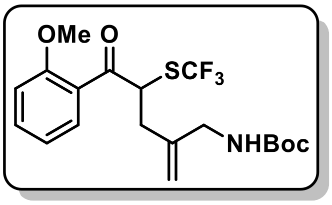

Following GPE with 1-(2-methoxyphenyl)-2-((trifluoromethyl)thio) ethan-1-one (176 mg, 0.703 mmol), *tert*-butyl 5-methylene-2-oxo-1,3-oxazinane-3-carboxylate (100 mg, 0.469 mmol), Pd(dba)$_2$ (13 mg, 0.02 mmol) and *N*,*N*-diisopropyldibenzo[*d*,*f*][1,3,2]dioxaphosphepin-6-amine (22 mg, 0.07 mmol) in CH_2Cl_2 (4.7 mL) with FCC (5 % EtOAc, 30% CH_2Cl_2 in 40-60 petroleum ether) gave *tert*-butyl (5-(2-methoxyphenyl)-2-methylene-5-oxo-4-((trifluoromethyl)thio)pentyl)carbamate (**12e**) as a yellow oil (179 mg, 91%).

^{1}H NMR (400 MHz, CDCl$_3$): δ 7.70 (dd, *J* = 8.0 Hz, 2.0 Hz, 1H), 7.54 - 7.48 (m, 1H), 7.03 (t, *J* = 7.0 Hz, 1H), 6.99 (d, *J* = 8.0 Hz, 1H), 5.27 (t, *J* = 7.0 Hz, 1H), 5.04 (s, 1H), 4.95 (s, 1H), 4.62 (br, 1H), 3.95 (s, 3H), 3.80 - 3.69 (m, 2H), 2.96 (dd, *J* = 15.0 Hz, 8.0 Hz, 1H), 2.59 (dd, *J* = 15.0 Hz, 7.0 Hz, 1H), 1.43 (s, 9H); **^{13}C NMR (101 MHz, CDCl$_3$)** δ 197.3, 158.3, 155.8, 142.1, 134.5, 131.8, 130.5 (q, $^1J_{C\text{-}F}$ = 308 Hz), 125.8, 121.1, 114.4, 111.6, 79.5, 55.6, 49.9, 44.9, 36.6, 28.3; **^{19}F NMR (377 MHz, CDCl$_3$)**: δ -39.5 (s); **FTIR:** ν_{max}/cm^{-1} (neat) 3384, 2981, 2929, 1692, 1598, 1506, 1486, 1438, 1367, 1276, 1260 1162, 1108, 1021, 764, 751, 643; **HRMS (ESI$^+$):** *m/z* calcd. for $C_{19}H_{24}F_3NO_4SNa$: 442.1270, found: 442.1290 [*M+Na*]$^+$.

tert-butyl (5-(3-chlorophenyl)-2-methylene-5-oxo-4-((trifluoromethyl)thio)pentyl) carbamate (12f)

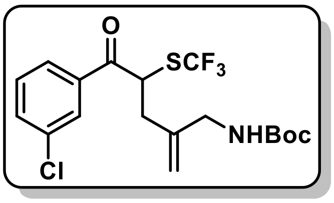

Following GPE with 1-(3-chlorophenyl)-2-((trifluoromethyl)thio)ethan-1-one (179 mg, 0.703 mmol), *tert*-butyl 5-methylene-2-oxo-1,3-oxazinane-3-carboxylate (100 mg, 0.469 mmol), Pd(dba)$_2$ (13 mg, 0.02 mmol) and *N*,*N*-diisopropyldibenzo[*d*,*f*][1,3,2]dioxaphosphepin-6-amine (22 mg, 0.07 mmol) in CH_2Cl_2 (4.7 mL) with FCC (5 % EtOAc, 30% CH_2Cl_2 in 40-60 petroleum ether) gave *tert*-butyl (5-(3-chlorophenyl)-2-methylene-5-oxo-4-((trifluoromethyl)thio)pentyl)carbamate (**12f**) as a yellow oil (174 mg, 87%).

^{1}H NMR (400 MHz, CDCl$_3$): δ 8.00 (s, 1H), 7.91 (d, *J* = 7.5 Hz, 1H), 7.59 (d, *J* = 8.0 Hz, 1H), 7.45 (t, *J* = 8.0 Hz, 1H), 5.10 (t, *J* = 7.0 Hz, 1H), 5.02 (s, 1H), 4.91 (s, 1H), 4.66 (br, 1H), 3.81 (dd, *J* = 16.0 Hz, 6.0 Hz, 1H), 3.70 (dd, *J* = 16.0 Hz, 6.0 Hz, 1H), 2.94 (dd, *J* = 14.5 Hz, 8.0 Hz, 1H), 2.65 (dd, *J* = 14.5 Hz, 7.0 Hz, 1H), 1.42 (s, 9H); **^{13}C NMR (101 MHz, CDCl$_3$)** δ 194.9, 156.0, 141.8, 136.6, 135.4, 133.9, 130.2, 130.1 (q, $^1J_{C\text{-}F}$ = 308 Hz), 128.8, 126.8, 116.0, 79.8, 45.0, 44.7, 36.1, 28.3; **^{19}F NMR (377 MHz, CDCl$_3$)**: δ -39.2 (s); **FTIR:** ν_{max}/cm^{-1} (neat) 3385, 2984, 2937, 1693, 1512, 1341, 1276, 1261, 1159, 1112, 1049, 764, 750; **HRMS (ESI$^+$):** *m/z* calcd. for $C_{18}H_{21}{}^{35}ClF_3NO_3SNa$: 446.0775, found: 446.0791 [*M+Na*]$^+$.

tert-butyl (2-methylene-5-oxo-5-(thiophen-2-yl)-4-((trifluoromethyl)thio)pentyl) carbamate (12g)

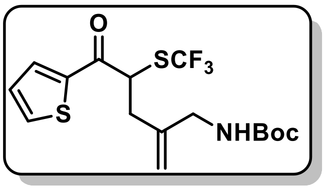

Following GPE with 1-(thiophen-2-yl)-2-((trifluoromethyl)thio)ethan-1-one (159 mg, 0.703 mmol), *tert*-butyl 5-methylene-2-oxo-1,3-oxazinane-3-carboxylate (100 mg, 0.469 mmol), Pd(dba)$_2$ (13 mg, 0.02 mmol) and *N*,*N*-diisopropyldibenzo[*d*,*f*][1,3,2]dioxaphosphepin-6-amine (22 mg, 0.07 mmol) in CH_2Cl_2 (4.7 mL) with FCC (5 % EtOAc, 30% CH_2Cl_2 in 40-60 petroleum ether) gave *tert*-butyl (2-methylene-5-oxo-5-(thiophen-2-yl)-4-((trifluoromethyl)thio)pentyl)carbamate (**12g**) as a yellow oil (150 mg, 81%).

^{1}H NMR (400 MHz, CDCl$_3$): δ 7.98 - 7.95 (m, 1H), 7.75 (d, *J* = 5.0 Hz, 1H), 7.18 (t, *J* = 5.0 Hz, 1H), 5.02 (s, 1H), 5.01 - 4.97 (m, 1H), 4.94 (s, 1H), 4.65 (br, 1H), 3.83 - 3.72 (m, 2H), 2.92 (dd, *J* = 14.5 Hz, 8.5 Hz, 1H), 2.63 (dd, *J* = 14.5 Hz, 7.0 Hz, 1H), 1.43 (s, 9H); **^{13}C NMR (101 MHz, CDCl$_3$)** δ 188.8, 156.0, 142.0, 141.6, 136.0, 133.7, 130.3 (q, $^1J_{C\text{-}F}$ = 308 Hz), 128.6, 116.2, 79.8, 46.3, 44.6, 36.4, 28.3; **^{19}F NMR (377 MHz, CDCl$_3$)**: δ -39.7 (s); **FTIR:** ν_{max}/cm^{-1} (neat) 3384, 2982, 2931, 1697, 1662, 1516, 1413, 1367, 1356, 1275, 1260 1157, 1112, 916, 857, 764, 750; **HRMS (ESI$^+$):** *m/z* calcd. for $C_{16}H_{20}F_3NO_3S_2Na$: 418.0729, found: 418.0743 [*M+Na*]$^+$.

tert-butyl (4-methyl-2-methylene-5-oxo-5-phenyl-4-((trifluoromethyl)thio)pentyl) carbamate (12h)

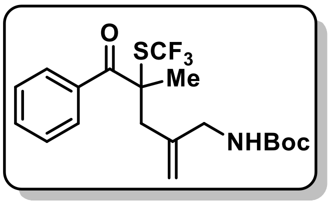

Following an adapted GPE with 1-phenyl-2-((trifluoromethyl)thio)propan-1-one (165 mg, 0.703 mmol), *tert*-butyl 5-methylene-2-oxo-1,3-oxazinane-3-carboxylate (100 mg, 0.469 mmol), Pd(dba)$_2$ (13 mg, 0.02 mmol) and *N*,*N*-diisopropyldibenzo[*d*,*f*][1,3,2]dioxaphosphepin-6-amine (22 mg, 0.07 mmol) in CH_2Cl_2 (4.7 mL). Reaction mixture was stirred and heated to reflux (DrySyn block) for 18 h. Solvent was then evaporated under reduced pressure and crude material was subjected to silica gel FCC (5 % EtOAc, 30% CH_2Cl_2 in 40-60 petroleum ether) to give *tert*-butyl (4-methyl-2-methylene-5-oxo-5-phenyl-4-((trifluoromethyl)thio)pentyl)carbamate (**12h**) as a yellow oil (159 mg, 84%).

^{1}H NMR (400 MHz, CDCl$_3$): δ 8.08 (d, *J* = 7.5 Hz, 2H), 7.54 (t, *J* = 7.5 Hz, 1H), 7.44 (t, *J* = 7.5 Hz, 2H), 5.13 (s, 1H), 4.90 (s, 1H), 4.63 (br, 1H), 3.65 (dd, *J* = 16.0 Hz, 6.0 Hz, 1H), 3.56 (dd, *J* = 16.0 Hz, 6.0 Hz, 1H), 3.08 (d, *J* = 14.5 Hz, 1H), 2.72 (d, *J* = 14.5 Hz , 1H), 1.75 (s, 3H), 1.43 (s, 9H); **^{13}C NMR (101 MHz, CDCl$_3$)** δ 198.7, 155.7, 140.5, 135.9, 132.5, 129.5, 129.4 (q, $^1J_{C\text{-}F}$ = 308 Hz), 128.4, 117.0, 79.6, 58.2, 46.2, 42.3, 28.4, 24.5; **^{19}F NMR (377 MHz, CDCl$_3$)**: δ -35.8 (s); **FTIR:** ν_{max}/cm^{-1} (neat) 3356, 2980, 2934, 1682, 1597, 1512, 1448, 1392, 1367, 1275, 1250, 1161, 1109, 1071, 971, 912, 757, 696; **HRMS (ESI$^+$):** *m/z* calcd. for $C_{19}H_{24}F_3NO_3SNa$: 426.1321, found: 426.1337 [*M+Na*]$^+$.

tert-butyl (2-methylene-5-oxo-4-((trifluoromethyl)thio)heptyl)carbamate (12i)

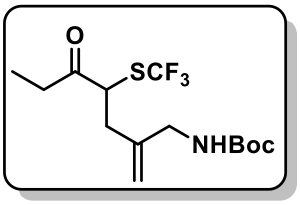

Following an adapted GPE with 1-((trifluoromethyl)thio)butan-2-one (121 mg, 0.703 mmol), *tert*-butyl 5-methylene-2-oxo-1,3-oxazinane-3-carboxylate (100 mg, 0.469 mmol), Pd(dba)$_2$ (13 mg, 0.02 mmol) and *N*,*N*-diisopropyldibenzo[*d*,*f*][1,3,2]dioxaphosphepin-6-amine (22 mg, 0.07 mmol) in CH_2Cl_2 (4.7 mL). Reaction mixture was stirred and heated to reflux (DrySyn block) for 18 h. Solvent was then evaporated under reduced pressure and crude material was subjected to silica gel FCC (5 % EtOAc, 30% CH_2Cl_2 in 40-60 petroleum ether) to give *tert*-butyl (2-methylene-5-oxo-4-((trifluoromethyl)thio)heptyl)carbamate (**12i**) as a yellow oil (136 mg, 85%).

^{1}H NMR (400 MHz, CDCl$_3$): δ 5.05 (s, 1H), 4.89 (s, 1H), 4.68 (br, 1H), 4.11 (t, *J* = 7.5 Hz, 1H), 3.78 (dd, *J* = 16.0 Hz, 6.5 Hz, 1H), 3.66 (dd, *J* = 16.0 Hz, 6.0 Hz, 1H), 2.80 - 2.57 (m, 3H), 2.44 (dd, *J* = 14.5 Hz, 7.0 Hz, 1H), 1.44 (s, 9H), 1.08 (t, *J* = 7.0 Hz, 3H); **^{13}C NMR (101 MHz, CDCl$_3$)** δ 206.6, 156.0, 141.8, 130.2 (q, $^1J_{C\text{-}F}$ = 308 Hz), 115.3, 79.4, 49.6, 44.6, 35.2, 34.0, 28.3, 7.69; **^{19}F NMR (377 MHz, CDCl$_3$)**: δ -39.7 (s); **FTIR:** ν_{max}/cm^{-1} (neat) 3363, 2981, 2935, 1702, 1513, 1457, 1392, 1367, 1275, 1254, 1158, 1116, 1048, 915, 757; **HRMS (ESI$^+$):** *m/z* calcd. for $C_{14}H_{22}F_3NO_3SNa$: 364.1165, found: 364.1181 [*M+Na*]$^+$.

Condensation Products

ethyl 3-fluoro-5-methylene-2-(phenyl)-3,4,5,6-tetrahydropyridine-3-carboxylate (4a)

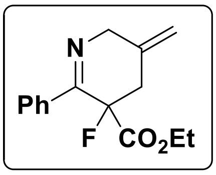

Following GPF using ethyl 2-(phenyl)-4-(((*tert*-butoxycarbonyl)amino)methyl)-2-fluoropent-4-enoate (**3a**) (74 mg, 0.19 mmol) and TFA (1.6 g, 14 mmol) afforded ethyl 3-fluoro-5-methylene-2-(phenyl)-3,4,5,6-tetrahydropyridine-3-carboxylate (**4a**) as a yellow oil (43 mg, 87%).

^{1}H NMR (400 MHz, CDCl$_3$) δ 7.72 – 7.66 (m, 2H), 7.39 – 7.31 (m, 3H), 5.09 (s, 1H), 5.02 (s, 1H), 4.64 (dd, *J* = 20.5, 5.5 Hz, 1H), 4.53 (dd, *J* = 20.5, 5.5 Hz, 1H), 4.19 – 4.06 (m, 2H), 3.00 – 2.83 (m, 2H), 1.03 (t, *J* = 7.0 Hz, 3H); **^{13}C NMR (101 MHz, CDCl$_3$)** δ 169.1 (d, $^2J_{C-F}$ = 26.5 Hz), 160.3 (d, $^2J_{C-F}$ = 18.5 Hz), 136.9, 136.3 (d, $^3J_{C-F}$ = 3.5 Hz), 130.0, 128.4, 127.2 (d, $^4J_{C-F}$ = 2.5 Hz), 112.6, 90.8 (d, $^1J_{C-F}$ = 196.0 Hz), 62.3, 56.0, 39.5 (d, $^2J_{C-F}$ = 24.0 Hz), 13.9; **^{19}F NMR (377 MHz, CDCl$_3$):** δ -146.5 – -146.7 (m); **FTIR:** ν_{max}/cm^{-1} (neat) 2983, 1754, 1635, 1447, 1322, 1262, 1076, 1061, 856, 694 cm^{-1}; **HRMS (ESI$^+$):** calculated for $C_{15}H_{17}FNO_2$ (ES$^+$)(+H$^+$): 262.1238. Found: 262.1240.

ethyl 3-fluoro-2-(4-methoxyphenyl)-5-methylidene-3,4,5,6-tetrahydropyridine-3-carboxylate (4b)

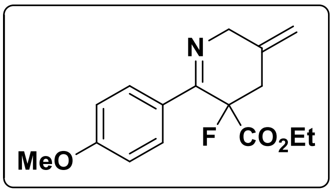

Following GPF 4-{[(*tert*-butoxycarbonyl)amino]methyl}-2-fluoro-2-(4-methoxybenzoyl)pent-4-enoate (**3b**) (78 mg, 0.18 mmol) and TFA (1.5 g, 13.5 mmol) afforded ethyl ethyl 3-fluoro-2-(4-methoxyphenyl)-5-methylidene-3,4,5,6-tetrahydropyridine-3-carboxylate (**4b**) as an orange oil (52 mg , 99%).

^{1}H NMR (400 MHz, CDCl$_3$): δ 7.67 (dd, *J* = 8.5, 1.0 Hz, 2H), 6.86 (d, *J* = 9.0 Hz, 2H), 5.07 (s, 1H), 5.00 (s, 1H), 4.60 (dd, *J* = 20.5, 5.0 Hz, 1H), 4.49 (dd, *J* = 20.5, 5.0 Hz, 1H), 4.15 (q, *J* = 7.0 Hz, 1H), 3.81 (s, 3H), 2.98 – 2.81 (m, 2H), 1.07 (t, *J* = 7.0 Hz, 3H); **^{13}C NMR (101 MHz, CDCl$_3$)** δ 169.3 (d, $^2J_{C-F}$ = 26.5 Hz), 161.1, 159.4 (d, $^2J_{C-F}$ = 18.5 Hz), 136.6 (d, $^3J_{C-F}$ = 4.0 Hz), 129.5, 128.9 (d, $^4J_{C-F}$ = 3.0 Hz), 113.7, 112.4, 91.0 (d, $^1J_{C-F}$ = 196.0 Hz), 62.3, 55.8, 55.4, 39.6 (d, $^2J_{C-F}$ = 24.0 Hz), 14.0; **^{19}F NMR (377 MHz, CDCl$_3$)**: δ -146.4 – -146.6 (m); **FTIR:** ν_{max}/cm^{-1} (neat) 2939, 1738, 1673, 1599, 1515, 1259, 1173, 1139, 1022, 838, 705 cm^{-1}; **HRMS (ESI$^+$):** calculated for $C_{16}H_{19}FNO_3$ (ES$^+$)(+H$^+$): 292.1343. Found: 292.1340.

ethyl 3-fluoro-2-(4-chlorophenyl)-5-methylidene-3,4,5,6-tetrahydropyridine-3-carboxylate (4c)

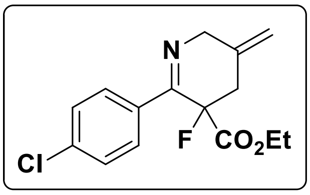

Following GPF using 4-{[(*tert*-butoxycarbonyl)amino]methyl}-2-fluoro-2-(4-chlorobenzoyl)pent-4-enoate (**3c**) (74 mg, 0.18 mmol) and TFA (1.5 g, 13.5 mmol) afforded ethyl ethyl 3-fluoro-2-(4-chlorophenyl)-5-methylidene-3,4,5,6-tetrahydropyridine-3-carboxylate (**4c**) as a colourless oil (50 mg , 94%).

^{1}H NMR (400 MHz, CDCl$_3$): δ 7.68 – 7.63 (m, 2H), 7.35 – 7.30 (m, 2H), 5.10 (s, 1H), 5.02 (s, 1H), 4.63 (dd, *J* = 21.0, 5.5 Hz, 1H), 4.51 (dd, *J* = 21.0, 5.5 Hz, 1H), 4.15 (q, *J* = 7.0 Hz, 2H), 2.99 – 2.81 (m, 2H), 1.08 (t, *J* = 7.0 Hz, 3H); **^{13}C NMR (101 MHz, CDCl$_3$)** δ 169.0 (d, $^2J_{C-F}$ = 26.5 Hz), 159.2 (d, $^2J_{C-F}$ = 18.5 Hz), 136.2, 136.0 (d, $^3J_{C-F}$ = 3.5 Hz), 135.3, 128.7 (d, $^4J_{C-F}$ = 3.0 Hz), 128.6, 112.8, 90.8 (d, $^1J_{C-F}$ = 196.0 Hz),

62.5, 56.1, 39.5 (d, $^2J_{C-F}$ = 24.0 Hz), 14.0; **^{19}F NMR (377 MHz, $CDCl_3$)**: δ -146.72 – -146.88 (m); **FTIR:** ν_{max}/cm^{-1} (neat) 2986, 1757, 1600, 1521, 1349, 1275, 1065, 852, 750 cm^{-1}; **HRMS (ESI$^+$):** calculated for $C_{15}H_{16}ClFNO_2$ (ES^+)(+H^+): 296.0848. Found: 296.0855.

ethyl 3-fluoro-2-(4-trifluorophenyl)-5-methylidene-3,4,5,6-tetrahydropyridine-3-carboxylate (4d)

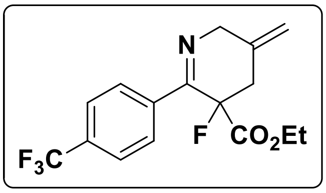

Following GPF using 4-{[(*tert*-butoxycarbonyl)amino]methyl}-2-fluoro-2-(4-trifluorobenzoyl)pent-4-enoate (**3d**) (76 mg, 0.17 mmol) and TFA (1.4 g, 12 mmol) afforded ethyl ethyl 3-fluoro-2-(4-trifluorophenyl)-5-methylidene-3,4,5,6-tetrahydropyridine-3-carboxylate (**4d**) as a pale yellow oil (50 mg , 89%).

^{1}H NMR (400 MHz, $CDCl_3$): δ 7.82 (d, *J* = 8.0 Hz, 2H), 7.61 (d, *J* = 8.5 Hz, 0H), 5.12 (s, 1H), 5.04 (s, 1H), 4.67 (dd, *J* = 21.0, 5.5 Hz, 1H), 4.55 (dd, *J* = 21.0, 5.5 Hz, 1H), 4.15 (q, *J* = 7.0 Hz, 2H), 3.02 – 2.84 (m, 2H), 1.06 (t, *J* = 7.0 Hz, 3H); **^{13}C NMR (101 MHz, $CDCl_3$)** δ 168.8 (d, $^2J_{C-F}$ = 26.5 Hz), 159.3 (d, $^2J_{C-F}$ = 19.0 Hz), 140.1, 135.8 (d, $^3J_{C-F}$ = 3.5 Hz), 131.8 (q, $^2J_{C-F}$ = 32.5 Hz), 127.7 (d, $^4J_{C-F}$ = 3.0 Hz), 125.4 (q, $^3J_{C-F}$ = 3.5 Hz), 123.6 (q, $^1J_{C-F}$ = 272.5 Hz), 113.1, 90.7 (d, $^1J_{C-F}$ = 196.0 Hz), 62.6, 56.2, 39.4 (d, $^2J_{C-F}$ = 24.0 Hz), 13.9; **^{19}F NMR (377 MHz, $CDCl_3$)** δ -62.9 (3F, s), -147.0 – -147.1 (1F, m); **FTIR:** ν_{max}/cm^{-1} (neat) 2986, 1758, 1638, 1325, 1315, 1267, 1166, 1124, 850 cm^{-1}; **HRMS (ESI$^+$):** calculated for $C_{15}H_{16}ClFNO_2$ (ES^+)(+H^+): 330.1112. Found: 330.1116.

ethyl 3-fluoro-2-(4-methylphenyl)-5-methylidene-3,4,5,6-tetrahydropyridine-3-carboxylate (4e)

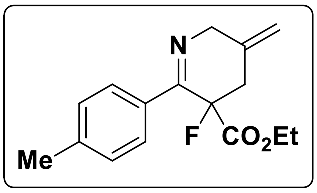

Following GPF using 4-{[(*tert*-butoxycarbonyl)amino]methyl}-2-fluoro-2-(4-methylbenzoyl)pent-4-enoate (**3e**) (60 mg, 0.15 mmol) and TFA (1.3 g, 11.4 mmol) afforded ethyl 3-fluoro-2-(4-methylphenyl)-5-methylidene-3,4,5,6-tetrahydropyridine-3-carboxylate (**4e**) as a yellow oil (39 mg , 94%).

^{1}H NMR (400 MHz, $CDCl_3$): δ 7.65 – 7.52 (m, 2H), 7.15 (d, *J* = 8.0 Hz, 2H), 5.08 (s, 1H), 5.00 (d, *J* = 1.0 Hz, 1H), 4.62 (dd, *J* = 20.5, 5.5 Hz, 1H), 4.51 (dd, *J* = 20.5, 5.5 Hz, 1H), 4.14 (q, *J* = 7.0 Hz, 2H), 3.01 – 2.80 (m, 2H), 2.34 (s, 3H), 1.07 (t, *J* = 7.0 Hz, 3H); **^{13}C NMR (101 MHz, $CDCl_3$)** δ 169.3 (d, $^2J_{C\text{-}F}$ = 26.5 Hz), 160.1 (d, $^2J_{C\text{-}F}$ = 18.5 Hz), 140.1, 136.5 (d, $^3J_{C\text{-}F}$ = 3.5 Hz), 134.1, 129.1, 127.2 (d, $^2J_{C\text{-}F}$ = 2.5 Hz), 112.4, 90.9 (d, $^1J_{C\text{-}F}$ = 196.0 Hz), 62.3, 55.9, 39.6 (d, $^2J_{C\text{-}F}$ = 24.0 Hz), 21.4, 13.9; **^{19}F NMR (377 MHz, $CDCl_3$)**: δ -146.54 – -146.70 (m); **FTIR:** ν_{max}/cm^{-1} (neat) 2984, 1755, 1633, 1611, 1445, 1267, 1186, 1063, 827, 764 cm^{-1}; **HRMS (ESI$^+$):** calculated for $C_{16}H_{19}FNO2$ (ES$^+$)(+H$^+$): 276.1394. Found: 276.1403.

ethyl 3-fluoro-2-(4-nitrophenyl)-5-methylidene-3,4,5,6-tetrahydropyridine-3-carboxylate (4f)

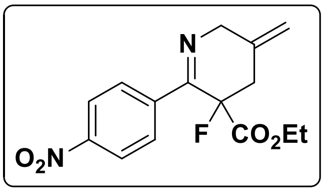

Following GPF using 4-{[(*tert*-butoxycarbonyl)amino]methyl}-2-fluoro-2-(4-nitrobenzoyl)pent-4-enoate (**3f**) (69 mg, 0.16 mmol) and TFA (1.4 g, 12 mmol) afforded ethyl ethyl 3-fluoro-2-(4-nitrophenyl)-5-methylidene-3,4,5,6-tetrahydropyridine-3-carboxylate (**4f**) as a yellow oil (45 mg , 92%).

^{1}H NMR (400 MHz, $CDCl_3$): δ 8.21 (d, *J* = 9.0 Hz, 2H), 7.89 (d, *J* = 8.0 Hz, 2H), 5.13 (s, 1H), 5.06 (s, 1H), 4.70 (dd, *J* = 21.0, 5.5 Hz, 1H), 4.58 (dd, *J* = 21.0, 5.5 Hz, 1H), 4.16 (q, *J* = 7.0 Hz, 2H), 3.04 – 2.84 (m, 2H), 1.09 (t, *J* = 7.0 Hz, 3H); **^{13}C NMR (101 MHz, $CDCl_3$)** δ 168.6 (d, $^2J_{C\text{-}F}$ = 26.5 Hz), 158.8 (d, $^2J_{C\text{-}F}$ = 18.5 Hz), 148.7, 142.4, 135.5 (d, $^3J_{C\text{-}F}$ = 4.0 Hz), 128.4 (d, $^4J_{C\text{-}F}$ = 3.0 Hz), 123.6, 113.4, 90.7 (d, $^1J_{C\text{-}F}$ = 196.5 Hz), 62.8, 56.4, 39.4 (d, $^2J_{C\text{-}F}$ = 24.0 Hz), 14.0; **^{19}F NMR (377 MHz, $CDCl_3$)**: δ -147.1 – -147.2 (m); **FTIR:** ν_{max}/cm^{-1} (neat) 2986, 1755, 1738, 1599, 1519, 1347, 1193, 850, 701 cm^{-1}; **HRMS (ESI$^+$):** calculated for $C_{15}H_{16}FN_2O_4$ (ES$^+$)(+H$^+$): 307.1089. Found: 307.1098.

ethyl 3-fluoro-2-(4-methylphenyl)-5-methylidene-3,4,5,6-tetrahydropyridine-3-carboxylate (4g)

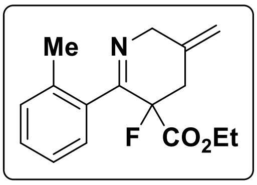

Following GPF using 4-{[(*tert*-butoxycarbonyl)amino]methyl}-2-fluoro-2-(2-methylbenzoyl)pent-4-enoate (**3g**) (67 mg, 0.18 mmol) and TFA (1.4 g, 12 mmol) afforded ethyl ethyl 3-fluoro-2-(2-methylphenyl)-5-methylidene-3,4,5,6-tetrahydropyridine-3-carboxylate (**4g**) as a colourless oil (46 mg, 99%).

^{1}H NMR (400 MHz, $CDCl_3$): δ 7.35 – 7.06 (m, 4H), 5.12 (s, 1H), 5.04 (s, 1H), 4.69 – 4.51 (m, 2H), 4.13 – 3.97 (m, 2H), 3.13 – 2.81 (m, 2H), 2.34 (s, 3H), 1.04 (t, *J* = 7.0 Hz, 3H); **^{13}C NMR (101 MHz, $CDCl_3$)** δ 168.5 (d, $^2J_{C\text{-}F}$ = 26.5 Hz), 161.9 (d, $^2J_{C\text{-}F}$ = 18.5 Hz), 136.8, 136.6, 136.0 (d, $^3J_{C\text{-}F}$ = 3.0 Hz), 130.8, 128.9, 127.2 (d, $^4J_{C\text{-}F}$ = 2.5 Hz), 125.5, 112.9, 91.1 (d, $^1J_{C\text{-}F}$ = 195.5 Hz), 62.3, 55.7, 38.9 (d, $^2J_{C\text{-}F}$ = 23.5 Hz), 19.9, 13.8; **^{19}F NMR (377 MHz, $CDCl_3$)** δ -148.2 (ddt, *J* = 25.0, 15.5, 6.0 Hz); **FTIR:** ν_{max}/cm^{-1} (neat) 2981, 1760, 1645, 1445, 1245, 1075, 1064, 1009, 903, 888, 728 cm^{-1}; **HRMS (ESI$^+$):** calculated for $C_{16}H_{19}FNO_2$ (ES$^+$)(+H$^+$): 276.1394. Found: 276.1398.

ethyl 3-fluoro-5-methylene-2-(naphthalen-2-yl)-3,4,5,6-tetrahydropyridine-3-carboxylate (4h)

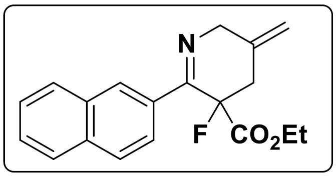

Following GPF using ethyl 2-(2-naphthoyl)-4-(((*tert*-butoxycarbonyl)amino)methyl)-2-fluoropent-4-enoate (**3h**) (80 mg, 0.19 mmol) and TFA (1.6 g, 14 mmol) afforded ethyl 3-fluoro-5-methylene-2-(naphthalen-2-yl)-3,4,5,6-tetrahydropyridine-3-carboxylate (**4h**) as a yellow oil (58 mg, 99%).

^{1}H NMR (400 MHz, $CDCl_3$) δ 8.19 (s, 1H), 7.91 – 7.79 (m, 4H), 7.53 – 7.44 (m, 2H), 5.13 (s, 1H), 5.06 (s, 1H), 4.66 (qd, *J* = 20.5, 5.5 Hz, 2H), 4.19 – 4.06 (m, 2H), 3.06 – 2.90 (m, 2H), 1.01 (t, *J* = 7.0 Hz, 3H); **^{13}C NMR (101 MHz, $CDCl_3$)** δ 169.3 (d, $^2J_{C-F}$ = 26.5 Hz), 160.1 (d, $^2J_{C-F}$ = 18.5 Hz), 136.3 (d, $^3J_{C-F}$ = 3.5 Hz), 134.2, 134.1, 132.9, 129.1, 128.1, 127.7, 127.2 (d, $^4J_{C-F}$ = 4.0 Hz), 127.1, 126.4, 124.6, 112.7, 91.0 (d, $^1J_{C-F}$ = 196.5 Hz), 62.4, 56.2, 39.7 (d, $^2J_{C-F}$ = 24.0 Hz), 13.9; **^{19}F NMR (377 MHz, $CDCl_3$)** δ -146.02 (ddt, *J* = 22.6, 18.2, 5.2 Hz); **FTIR:** $ν_{max}$/cm^{-1} (neat) 3059, 2983, 2928, 1754, 1738, 1626, 1319, 1266, 1187, 1063 cm^{-1}; **HRMS (ESI$^+$):** calculated for $C_{19}H_{19}FNO_2$ (ES$^+$)(+H$^+$): 312.1394. Found: 312.1401.

ethyl 3-fluoro-5-methylidene-2-(thiophen-2-yl)-3,4,5,6-tetrahydropyridine-3-carboxylate (4i)

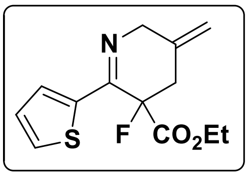

Following GPF using ethyl 4-{[(*tert*-butoxycarbonyl)amino]methyl}-2-fluoro-2-(thiophene-2-carbonyl)pent-4-enoate (**3i**) (63 mg, 0.18 mmol) and TFA (1.5 g, 13.5 mmol) afforded ethyl 3-fluoro-5-methylidene-2-(thiophen-2-yl)-3,4,5,6-tetrahydropyridine-3-carboxylate (**4i**) as an orange oil (46 mg, 95%).

^{1}H NMR (400 MHz, $CDCl_3$) δ 7.41 (t, *J* = 3.0 Hz, 1H), 7.35 (d, *J* = 5.0 Hz, 1H), 7.03 – 6.98 (m, 1H), 5.06 (s, 1H), 4.98 (s, 1H), 4.55 (dd, *J* = 20.5, 4.0 Hz, 1H), 4.46 (d, *J* = 20.5 Hz, 1H), 4.28 – 4.14 (m, 2H), 3.02 – 2.84 (m, 2H), 1.16 (t, *J* = 7.0 Hz, 3H); **^{13}C NMR (101 MHz, $CDCl_3$)** δ 168.7 (d, $^2J_{C-F}$ = 27.5 Hz), 155.4 (d, $^2J_{C-F}$ = 20.5 Hz), 141.8 (d, $^3J_{C-F}$ = 2.5 Hz), 136.4 (d, $^3J_{C-F}$ = 5.5 Hz), 128.9, 128.5 (d, $^4J_{C-F}$ = 7.0 Hz), 127.8, 112.6, 91.0 (d, $^1J_{C-F}$ = 197.0 Hz), 62.6, 55.5, 39.6 (d, $^2J_{C-F}$ = 24.0 Hz), 14.1; **^{19}F NMR (377 MHz, $CDCl_3$)** δ -147.7 – -147.8 **FTIR:** $ν_{max}$/cm^{-1} (neat) 2984, 1753, 1622, 1429, 1285, 1189, 1065, 1008, 909, 694 cm^{-1}; **HRMS (ESI$^+$):** calculated for $C_{13}H_{15}FNO_2S$ (ES$^+$)(+H$^+$): 268.0802. Found: 268.0806.

3-fluoro-5-methylidene-2-phenyl-4,6-dihydropyridine-3-carbonitrile (4j)

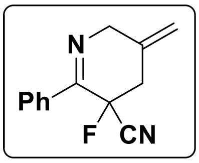

Following GPF using *tert*-butyl *N*-[4-cyano-4-fluoro-4-(4-methoxybenzoyl)-2-methylidenebutyl]carbamate (**3j**) (58 mg, 0.16 mmol) and TFA (1.4 g, 12 mmol) afforded 3-fluoro-2-(4-methoxyphenyl)-5-methylidene-3,4,5,6-tetrahydropyridine -3-carboxylate (**4j**) as a yellow oil (34 mg , 99%).

^{1}H NMR (400 MHz, $CDCl_3$): δ 7.87 (d, *J* = 8.0 Hz, 2H), 7.50 – 7.36 (m, 3H), 5.24 (s, 2H), 4.62 – 4.45 (m, 2H), 3.16 (t, *J* = 13.0 Hz, 1H), 3.03 (t, *J* = 13.0 Hz, 1H); **^{13}C NMR (101 MHz, $CDCl_3$)** δ 157.8 (d, $^2J_{C-F}$ = 22.0 Hz), 134.7, 134.2 (d, $^3J_{C-F}$ = 5.5 Hz), 130.9, 128.6, 127.8 (d, $^4J_{C-F}$ = 2.5 Hz), 116.0 (d, $^2J_{C-F}$ = 35.0 Hz), 115.5, 84.0 (d, $^1J_{C-F}$ = 196.0 Hz), 55.9, 40.9 (d, $^2J_{C-F}$ = 23.5 Hz); **^{19}F NMR (377 MHz, $CDCl_3$)**: δ -142.6 (t, *J* = 12.5 Hz);); **FTIR:** $ν_{max}$/cm^{-1} (neat) 2927, 2253, 1634, 1447, 1322, 1281, 1065, 906, 728 cm^{-1}; **HRMS (ESI$^+$):** calculated for $C_{13}H_{12}FN_2$ (ES$^+$)(+Na$^+$): 215.0979. Found: 215.0984.

5-fluoro-3-methylene-6-phenyl-5-tosyl-2,3,4,5-tetrahydropyridine (4k)

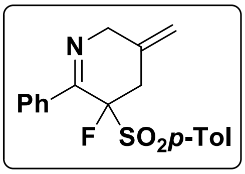

Following GPF using *tert*-butyl (4-fluoro-2-methylene-5-oxo-5-phenyl-4-tosylpentyl)carbamate (**3k**) (85 mg, 0.18 mmol) and TFA (1.1g, 9.2 mmol)

afforded 5-fluoro-3-methylene-6-phenyl-5-tosyl-2,3,4,5-tetrahydropyridine (**4k**) as a yellow oil (56 mg, 89%).

^{1}H NMR (400 MHz, CDCl$_3$) δ 7.66 – 7.57 (m, 4H), 7.35 – 7.29 (m, 1H), 7.25 – 7.15 (m, 4H), 5.13 (s, 1H), 5.03 (s, 1H), 4.84 – 4.76 (m, 1H), 4.76 – 4.67 (m, 1H), 3.17 (dd, *J* = 15.5, 6.0 Hz, 1H), 2.94 – 2.82 (m, 1H), 2.39 (s, 3H); **^{13}C NMR (101 MHz, CDCl$_3$)** δ 159.9 (d, $^{2}J_{C\text{-}F}$ = 18.0 Hz), 146.0, 136.6 (d, $^{3}J_{C\text{-}F}$ = 3.0 Hz), 135.8 (d, $^{3}J_{C\text{-}F}$ = 8.0 Hz), 131.5, 130.5, 129.8, 129.5, 129.1 (d, $^{4}J_{C\text{-}F}$ = 5.5 Hz), 127.9, 112.4, 104.5 (d, $^{1}J_{C\text{-}F}$ = 234.5 Hz), 55.9, 36.4 (d, $^{2}J_{C\text{-}F}$ = 21.0 Hz), 21.8; **^{19}F NMR (377 MHz, CDCl$_3$)** δ -141.5 – -141.6 (m); **FTIR:** ν_{max}/cm^{-1} (neat) 3061, 2924, 1670, 1596, 1413, 1317, 1146 cm^{-1}; **HRMS (ESI^{+}):** calculated for $C_{19}H_{19}FNO_2S$ (ES^{+})(+H^{+}): 344.1115. Found: 344.1121.

3-fluoro-*N*-methoxy-*N*-methyl-5-methylene-2-phenyl-3,4,5,6-tetrahydropyridine-3-carboxamide (4l)

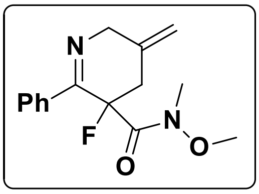

Following GPF using *tert*-butyl (4-benzoyl-4-fluoro-5-(methoxy(methyl)amino)-2-methylene-5-oxopentyl)carbamate (**3l**) (71 mg, 0.18 mmol) and TFA (1.0 g, 9.0 mmol) afforded 3-fluoro-*N*-methoxy-*N*-methyl-5-methylene-2-phenyl-3,4,5,6-tetrahydropyridine-3-carboxamide (**4l**) as an orange oil (48 mg, 97%).

^{1}H NMR (400 MHz, CDCl$_3$) δ 7.86 – 7.77 (m, 2H), 7.46 – 7.31 (m, 3H), 5.08 (s, 1H), 5.01 (s, 1H), 4.62 (dd, *J* = 19.5, 5.0 Hz, 1H), 4.35 (dd, *J* = 19.5, 7.5 Hz, 1H), 3.44 (s, 3H), 3.14 – 2.94 (m, 4H), 2.83 (dd, *J* = 21.5, 14.5 Hz, 1H); **^{13}C NMR (101 MHz, CDCl$_3$)** δ 159.8 (d, $^{2}J_{C\text{-}F}$ = 19.5 Hz), 136.7 (m), 136.1, 129.9, 128.3, 127.4 (d, $^{4}J_{C\text{-}F}$ = 2.0 Hz), 112.4, 92.1 (d, $^{1}J_{C\text{-}F}$ = 191.5 Hz), 60.9, 55.8, 38.5 (d, $^{2}J_{C\text{-}F}$ = 24.5 Hz), 33.4; **^{19}F NMR (376 MHz, CDCl$_3$)** δ -140.8 – -141.1 (m); **FTIR:** ν_{max}/cm^{-1} (neat) 2939, 1677, 1631, 1446, 1380, 1064, 972 cm^{-1}; **HRMS (ESI^{+}):** calculated for $C_{15}H_{18}FN_2O_2$ (ES^{+})(+H^{+}): 277.1347. Found: 277.1355.

ethyl 2-methyl-3-fluoro-5-methylidene-4,6-dihydropyridine-3-carboxylate (4m)

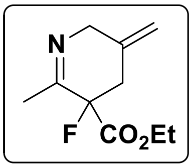

Following GPF using ethyl 2-acetyl-4-{[(*tert*-butoxycarbonyl)amino]methyl}-2-fluoropent-4-enoate (**3m**) (56 mg, 0.18 mmol) and TFA (1.5 g, 13.5 mmol) afforded ethyl 2-methyl-3-fluoro-5-methylidene-4,6-dihydropyridine-3-carboxylate (**4m**) as a yellow oil (32 mg, 91%).

^{1}H NMR (400 MHz, CDCl$_3$): δ 4.99 (d, *J* = 1.0 Hz, 1H), 4.92 (s, 1H), 4.36 – 4.14 (m, 4H), 2.91 (t, *J* = 14.0 Hz, 1H), 2.78 – 2.65 (m, 1H), 2.05 (dd, *J* = 3.5, 2.0 Hz, 3H), 1.29 (t, *J* = 7.0 Hz, 3H); **^{13}C NMR (101 MHz, CDCl$_3$)** δ 168.3 (d, $^{2}J_{C\text{-}F}$ = 27.5 Hz), 161.6 (d, $^{2}J_{C\text{-}F}$ = 21.0 Hz), 136.8 (d, $^{3}J_{C\text{-}F}$ = 6.0 Hz), 112.4, 91.7 (d, $^{1}J_{C\text{-}F}$ = 193.5 Hz), 62.5, 55.3, 38.5 (d, $^{2}J_{C\text{-}F}$ = 23.5 Hz), 21.5, 14.2; **^{19}F NMR (377 MHz, CDCl$_3$):** δ -152.5 – -152.9 (m); **FTIR:** ν_{max}/cm^{-1} (neat) 2984, 1756, 1739, 1668, 1442, 1370, 1278, 1183, 1072, 1041, 903, 759 cm^{-1}; **HRMS (ESI^{+}):** calculated for $C_{10}H_{15}FNO_2$ (ES^{+})(+H^{+}): 200.1081. Found: 200.1083.

ethyl 2-ethyl-3-fluoro-5-methylidene-4,6-dihydropyridine-3-carboxylate (4n)

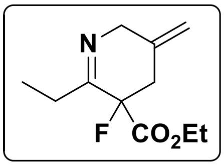

Following GPF using ethyl 4-({[(*tert*-butoxy)carbonyl]amino}methyl)-2-fluoro-2-propanoylpent-4-enoate (**3n**) (52 mg, 0.16 mmol) and TFA (1.4 g, 12 mmol) afforded ethyl 2-ethyl-3-fluoro-5-methylidene-4,6-dihydropyridine-3-carboxylate (**4n**) as a colourless oil (32 mg, 94%).

^{1}H NMR (400 MHz, CDCl$_3$): δ 4.98 (s, 1H), 4.90 (s, 1H), 4.42 – 4.15 (m, 4H), 2.90 (t, *J* = 14.0 Hz, 1H), 2.78 – 2.65 (m, 1H), 2.51 – 2.38 (m, 1H), 2.34 – 2.19 (m, 1H), 1.28 (t, *J* = 7.0 Hz, 3H), 1.09 (t, *J* = 7.5 Hz, 3H); **^{13}C NMR (101 MHz, CDCl$_3$)** δ 168.6 (d, $^{2}J_{C\text{-}F}$ = 27.0 Hz), 165.0 (d, $^{2}J_{C\text{-}F}$ = 20.5 Hz), 137.1 (d,

$^{3}J_{C-F}$ = 6.0 Hz), 112.0, 91.8 (d, $^{1}J_{C-F}$ = 194.0 Hz), 62.4, 55.2, 38.7 (d, $^{2}J_{C-F}$ = 23.5 Hz), 27.4, 14.2, 10.2; **^{19}F NMR (377 MHz, CDCl$_3$):** δ -153.4 – -153.8 (m); **FTIR:** $ν_{max}$/cm^{-1} (neat) 2981, 1756, 1738, 1665, 1446, 1369, 1274, 1178, 1067, 999, 901, 856 cm^{-1}; **HRMS (ESI$^+$):** calculated for $C_{11}H_{17}FNO_2$ (ES$^+$)(+H$^+$): 214.1238. Found: 214.1248.

ethyl 3-fluoro-5-methylidene-2-(propan-2-yl)-3,4,5,6-tetrahydropyridine-3-carboxylate (4o)

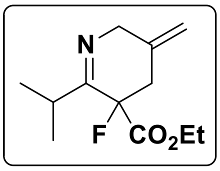

Following GPF using ethyl 4-({[(*tert*-butoxy)carbonyl]amino}methyl)-2-fluoro-2-propanoylpent-4-enoate (**3o**) (63 mg, 0.18 mmol) and TFA (1.5 g, 13.5 mmol) afforded ethyl 3-fluoro-5-methylidene-2-(propan-2-yl)-3,4,5,6-tetrahydropyridine-3-carboxylate (**4o**) as a colourless oil (32 mg, 93%).

^{1}H NMR (400 MHz, CDCl$_3$): δ 4.97 (d, *J* = 1.0 Hz, 1H), 4.89 (s, 1H), 4.43 – 4.16 (m, 4H), 2.87 (dd, *J* = 13.5, 13.0 Hz, 1H), 2.76 – 2.63 (m, 2H), 1.27 (t, *J* = 7.0 Hz, 3H), 1.12 (dd, *J* = 6.7, 0.5 Hz, 3H), 1.08 (d, *J* = 7.0 Hz, 3H); **^{13}C NMR (101 MHz, CDCl$_3$)** δ 169.1 (d, *J* = 20.0 Hz), 168.7 (d, *J* = 27.5 Hz), 137.3 (d, *J* = 6.5 Hz), 111.8, 92.3 (d, *J* = 193.5 Hz), 62.3, 55.2, 39.2 (d, *J* = 23.5 Hz), 32.8, 21.2 (d, *J* = 37.5 Hz), 14.2; **^{19}F NMR (376 MHz, CDCl$_3$)**: δ -154.7 – -154.8 (m); **FTIR:** $ν_{max}$/cm^{-1} (neat) 2975, 1755, 1661, 1446, 1272, 1222, 1072, 1011, 901, 855 cm^{-1}; **HRMS (ESI$^+$):** calculated for $C_{12}H_{19}FNO_2$ (ES$^+$)(+H$^+$): 228.1394. Found: 228.1396.

ethyl 2-tert-butyl-3-fluoro-5-methylidene-4,6-dihydropyridine-3-carboxylate (4p)

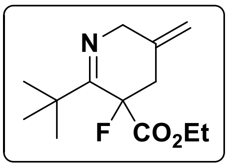

Following GPF using ethyl 4-{[(*tert*-butoxycarbonyl)amino]methyl}-2-(2,2-dimethylpropanoyl)-2-fluoropent-4-enoate (**3p**) (58 mg, 0.16 mmol) and TFA (1.4 g, 12 mmol) afforded ethyl 2-tert-butyl-3-fluoro-5-methylidene-4,6-dihydropyridine-3-carboxylate (**4p**) as a yellow oil (37 mg, 96%).

^{1}H NMR (400 MHz, CDCl$_3$): δ 4.94 (s, 1H), 4.84 (s, 1H), 4.45 – 4.28 (m, 2H), 4.27 – 4.13 (m, 2H), 2.85 – 2.67 (m, 2H), 1.25 (t, *J* = 7.0 Hz, 3H), 1.18 (d, *J* = 1.0 Hz, 9H; **^{13}C NMR (101 MHz, CDCl$_3$)** δ 170.5 (d, $^{2}J_{C-F}$ = 18.5 Hz), 168.8 (d, $^{2}J_{C-F}$ = 26.0 Hz), 138.1 (d, $^{3}J_{C-F}$ = 9.0 Hz), 111.2, 93.5 (d, $^{1}J_{C-F}$ = 199.0 Hz), 62.2, 55.1, 40.6 (d, $^{2}J_{C-F}$ = 24.0 Hz), 29.8, 28.8 (d, $^{3}{}_{C-F}$ = 3.5 Hz), 14.2; **^{19}F NMR (377 MHz, CDCl$_3$)** δ -154.7 – -154.9 (m); **FTIR:** $ν_{max}$/cm^{-1} (neat) 2960, 1754, 1725, 1690, 1574, 1545, 1367, 1273, 1202, 1090, 855, 840 cm^{-1}; **HRMS (ESI$^+$):** calculated for $C_{13}H_{21}FNO_2$ (ES$^+$)(+H$^+$): 242.1551. Found: 242.1555.

ethyl 2-cyclohexyl-3-fluoro-5-methylidene-3,4,5,6-tetrahydropyridine-3-carboxylate (4q)

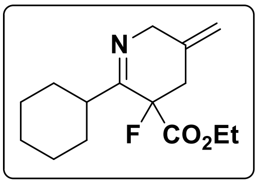

Following GPF using ethyl 4-({[(*tert*-butoxy)carbonyl]amino}methyl)-2-cyclohexanecarbonyl-2-fluoropent-4-enoate (**3q**) (62 mg, 0.16 mmol) and TFA (1.4 g, 12 mmol) afforded ethyl 2-cyclohexyl-3-fluoro-5-methylidene-3,4,5,6-tetrahydropyridine-3-carboxylate (**4q**) as a pale yellow oil (41 mg, 96%).

^{1}H NMR (400 MHz, CDCl$_3$): δ 4.97 (s, 1H), 4.89 (s, 1H), 4.45 – 4.18 (m, 4H), 2.87 (t, *J* = 13.5 Hz, 1H), 2.75 – 2.62 (m, 1H), 2.36 (t, *J* = 10.5 Hz, 1H), 1.90 – 1.60 (m, 5H), 1.48 – 1.12 (m, 8H); **^{13}C NMR (101 MHz, CDCl$_3$)** δ 168.7 (d, $^{2}J_{C-F}$ = 27.5 Hz), 168.3 (d, $^{2}J_{C-F}$ = 20.0 Hz), 137.2 (d, $^{3}J_{C-F}$ = 6.5 Hz), 111.9, 92.2 (d, $^{1}J_{C-F}$ = 194.0 Hz), 62.3, 55.2, 42.9, 39.1 (d, $^{2}J_{C-F}$ = 23.5 Hz), 31.7, 31.2, 26.4, 26.3, 26.0, 14.3; **^{19}F NMR (376 MHz, CDCl$_3$)**: δ -154.4– -154.6 (m); **FTIR:** $ν_{max}$/cm^{-1} (neat) 2930, 2853, 1756, 1659, 1668, 1448, 1273, 1075, 1010, 897 cm^{-1}; **HRMS (ESI$^+$):** calculated for $C_{15}H_{23}FNO_2$ (ES$^+$)(+H$^+$): 268.1707. Found: 268.1710.

ethyl 3-fluoro-5-methylene-2-(pent-4-en-1-yl)-3,4,5,6-tetrahydropyridine-3-carboxylate (4r)

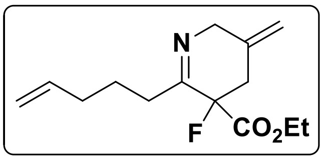

Following GPF using ethyl 2-(2-(((*tert*-butoxycarbonyl)amino)methyl)allyl)-2-fluoro-3-oxooct-7-enoate (**3r**) (70 mg, 0.19 mmol) and TFA (1.07 g, 9.4 mmol) afforded ethyl 3-fluoro-5-methylene-2-(pent-4-en-1-yl)-3,4,5,6-tetrahydropyridine-3-carboxylate (**4r**) as a yellow oil (47 mg, 98%).

^{1}H NMR (400 MHz, $CDCl_3$) δ 5.77 (ddt, *J* = 17.0, 10.0, 6.5 Hz, 1H), 5.03 – 4.87 (m, 4H), 4.41 – 4.17 (m, 4H), 2.89 (t, *J* = 14.0 Hz, 1H), 2.76 – 2.64 (m, 1H), 2.44 – 2.33 (m, 1H), 2.31 – 2.21 (m, 1H), 2.10 – 2.02 (m, 2H), 1.73 – 1.62 (m, 2H), 1.28 (t, *J* = 7.0 Hz, 3H); **^{13}C NMR (101 MHz, $CDCl_3$)** δ 168.5 (d, $^2J_{C-F}$ = 27.5 Hz), 163.9 (d, $^2J_{C-F}$ = 20.5 Hz), 138.4, 137.0 (d, $^3J_{C-F}$ = 6.0 Hz), 115.0, 112.1, 91.9 (d, $^1J_{C-F}$ = 194.0 Hz), 62.4, 55.2 (d, $^3J_{C-F}$ = 1.0 Hz), 38.7 (d, $^2J_{C-F}$ = 23.5 Hz), 33.6, 33.4, 25.1, 14.2; **^{19}F NMR (376 MHz, $CDCl_3$)** δ -153.45 – -153.7 (m); **FTIR:** ν_{max}/cm^{-1} (neat) 3078, 2980, 2936, 1756, 1738, 1665, 1272, 1177, 1066, 906 cm^{-1}; **HRMS (ESI$^+$):** calculated for $C_{14}H_{21}FNO_2$ (ES$^+$)(+H$^+$): 254.1551. Found: 254.1555.

ethyl 3-fluoro-5-methylene-2-(1-tosylpyrrolidin-2-yl)-3,4,5,6-tetrahydropyridine-3-carboxylate (4s)

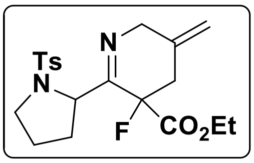

Following GPF using ethyl 4-(((*tert*-butoxycarbonyl)amino)methyl)-2-fluoro-2-(1-tosylpyrrolidine-2-carbonyl)pent-4-enoate (**3s**) (76 mg, 0.14 mmol) and TFA (0.82 g, 7.2 mmol) afforded ethyl 3-fluoro-5-methylene-2-(1-tosylpyrrolidin-2-yl)-3,4,5,6-tetrahydropyridine-3-carboxylate (**4s**) as a yellow oil (56 mg, 68%) (4.1:1 mixture of diastereoisomers).

Major Isomer

^{1}H NMR (400 MHz, $CDCl_3$) δ 7.75 – 7.65 (m, 1H), 7.33 – 7.25 (m, 1H), 4.97 (s, 1H), 4.92 (s, 1H), 4.65 – 4.48 (m, 1H), 4.43 – 4.20 (m, 2H), 3.54 – 3.25 (m, 1H), 3.02 – 2.89 (m, 1H), 2.79 – 2.55 (m, 1H), 2.41 (s, 2H), 1.98 – 1.80 (m, 2H), 1.68 – 1.55 (m, 1H), 1.34 – 1.28 (m, 2H); **^{13}C NMR (101 MHz, $CDCl_3$)** δ 167.8 (d, $^2J_{C-F}$ = 28.0 Hz), 163.8 (d, $^2J_{C-F}$ = 20.0 Hz), 143.2, 136.9 (d, $^3J_{C-F}$ = 7.5 Hz), 135.7, 129.6, 127.6, 111.9, 91.4 (d, $^1J_{C-F}$ = 192.0 Hz), 63.0, 60.6, 54.7, 48.7, 38.7 (d, $^2J_{C-F}$ = 22.5 Hz), 32.0, 24.2, 21.6, 14.1; **^{19}F NMR (376 MHz, $CDCl_3$)** δ -154.0 – -154.2 (m).

Minor Isomer

^{1}H NMR (400 MHz, $CDCl_3$) δ 7.75 – 7.65 (m, 1H), 7.33 – 7.25 (m, 1H), 4.97 (s, 1H), 4.92 (s, 1H), 4.65 – 4.48 (m, 1H), 4.43 – 4.20 (m, 2H), 3.54 – 3.25 (m, 1H), 3.02 – 2.89 (m, 1H), 2.79 – 2.55 (m, 1H), 2.41 (s, 2H), 1.98 – 1.80 (m, 2H), 1.68 – 1.55 (m, 1H), 1.34 – 1.28 (m, 2H); **^{13}C NMR (101 MHz, $CDCl_3$)** δ 167.9 (d, $^2J_{C-F}$ = 27.5 Hz), 164.1 (d, $^2J_{C-F}$ = 19.5 Hz), 143.5, 136.9 (d, $^3J_{C-F}$ = 7.5 Hz), 135.7, 129.7, 127.9, 112.1, 91.1 (d, $^1J_{C-F}$ = 194.5 Hz), 62.7, 60.6, 55.0, 49.6, 39.4 (d, $^2J_{C-F}$ = 23.0 Hz), 32.0, 24.3, 22.6, 14.1; **^{19}F NMR (376 MHz, $CDCl_3$)** δ -153.1 – -153.2 (m).

FTIR: ν_{max}/cm^{-1} (neat) 3423, 2982, 1748, 1668, 1339, 1156, 1091, 1010 cm^{-1}; **HRMS (ESI$^+$):** calculated for $C_{20}H_{26}FN_2O_4S$ (ES$^+$)(+H$^+$): 409.1592. Found: 409.1601.

ethyl 3-fluoro-5-methylene-2-phenylpiperidine-3-carboxylate (5)

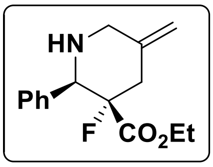

To a solution of $NaBH(OAc)_3$ (0.24 g, 1.15 mmol) in acetic acid (1.5 mL) under nitrogen was added 3-fluoro-5-methylene-2-(phenyl)-3,4,5,6-tetrahydropyridine-3-carboxylate (**4a**) (0.20 g, 0.77 mmol) and the resulting mixture stirred at 20 °C overnight. The mixture was then diluted with sat.

$NaHCO_3$ (10 mL) and extracted with EtOAc (5 x 10 mL). The combined organic layers were then dried over anhydrous $MgSO_4$, concentrated under vacuum and the residue purified by FCC (50% EtOAc in 40-60 petroleum ether) to afford (2R,3S)-ethyl 3-fluoro-5-methylene-2-phenylpiperidine-3-carboxylate (**5**) as a yellow oil (0.18 g, 90%).

^{1}H NMR (400 MHz, CDCl$_3$) δ 7.32 – 7.25 (m, 5H), 4.97 – 4.93 (m, 1H), 4.81 (s, 1H), 4.08 – 3.96 (m, 3H), 3.70 – 3.64 (m, 1H), 3.41 (d, *J* = 14.5 Hz, 1H), 2.99 – 2.92 (m, 1H), 2.80 – 2.69 (m, 1H), 2.49 (br, 1H), 1.02 (t, *J* = 7.0 Hz, 3H); **^{13}C NMR (101 MHz, CDCl$_3$)** δ 169.4 (d, $^2J_{C-F}$ = 24.5 Hz), 141.7 (d, $^3J_{C-F}$ = 8.5 Hz), 137.1, 128.2, 128.0, 127.5 (d, $^3J_{C-F}$ = 1.5 Hz), 111.4, 94.4 (d, $^1J_{C-F}$ = 200.0 Hz), 65.2, 65.0, 61.5 (d, $^2J_{C-F}$ = 27.5 Hz), 52.1, 42.5 (d, $^2J_{C-F}$ = 23.0 Hz), 14.0; **^{19}F NMR (377 MHz, CDCl$_3$)** δ -149.7 (ddd, *J* = 11.0, 7.5, 3.0 Hz); **FTIR:** ν_{max}/cm^{-1} (neat) 2983, 1724, 1466, 1372, 1228, 1192, 1072, 1040 cm^{-1}; **HRMS (ESI$^+$):** calculated for $C_{15}H_{19}FNO_2$ (ES$^+$)(+H$^+$): 264.1394. Found: 264.1396.

1-*tert*-butyl 3-ethyl 3-fluoro-5-methylene-2-phenylpiperidine-1,3-dicarboxylate (6)

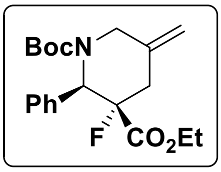

To a solution of (2R,3S)-ethyl 3-fluoro-5-methylene-2-phenylpiperidine-3-carboxylate (**5**) (0.18 g, 0.68 mmol) in THF (3.9 mL) under nitrogen was added Et3N (0.16 g, 1.5 mmol) and di-*tert*-butyl dicarbonate (0.34 g, 1.5 mmol) and the resulting mixture stirred at room temperature overnight. The reaction was then diluted with H_2O (20 mL) and extracted with DCM (4 x 20 mL). The combined organic layers were then dried over anhydrous $MgSO_4$, concentrated under vacuum and purified by FCC (4% EtOAc in 40-60 petroleum ether) to afford (2R,3S)-1-*tert*-butyl 3-ethyl 3-fluoro-5-methylene-2-phenylpiperidine-1,3-dicarboxylate (**6**) as a colourless oil (0.22 g, 88%).

^{1}H NMR (400 MHz, CDCl$_3$) δ 7.32 – 7.24 (m, 5H), 5.43 (br, 1H), 5.15 (s, 1H), 5.01 (s, 1H), 4.56 – 4.42 (m, 1H), 4.11 – 3.89 (m, 3H), 3.16 (dd, *J* = 43.5, 16.5 Hz, 1H), 2.84 – 2.72 (m, 1H), 1.34 (br, 9H), 1.04 (t, *J* = 7.0 Hz, 3H); **^{13}C NMR (101 MHz, CDCl$_3$)** δ 168.2 (d, $^2J_{C-F}$ = 23.0 Hz), 154.9, 141.1, 136.4, 128.5, 128.1, 128.0, 114.0, 94.8 (d, $^1J_{C-F}$ = 189.5 Hz), 80.6, 79.3, 62.0, 46.0, 34.3 (d, $^2J_{C-F}$ = 23.0 Hz), 28.2, 13.6; **^{19}F NMR (377 MHz, CDCl$_3$)** δ -149.2 (dt, *J* = 42.0, 13.0 Hz); **FTIR:** ν_{max}/cm^{-1} (neat) 2979, 2933, 1747, 1694, 1455, 1392, 1367, 1275, 1253, 1157, 1106, 1060, 1011. 895, 858, 756, 700; HRMS (ESI+): calculated for $C_{20}H_{26}FNO_4Na$ (ES+)(+Na$^+$): 386.1738. Found: 386.1747.

(3-fluoro-5-methylene-2-phenyl-3,4,5,6-tetrahydropyridin-3-yl)methanol (7)

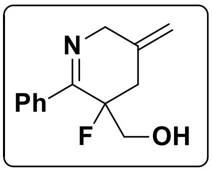

To a suspension of $LiAlH_4$ (15 mg, 0.38 mmol) in THF (2 mL) under nitrogen was added 3-fluoro-5-methylene-2-(phenyl)-3,4,5,6-tetrahydropyridine-3-carboxylate (**4a**) (50 mg, 0.19 mmol) in THF (2 mL) and the resulting mixture stirred at 20 °C for 3 hours. H_2O (0.2 mL) was then added dropwise and the reaction stirred for 15 minutes before the addition of anhydrous $MgSO_4$. After 10 minutes the reaction mixture was filtered through celite and the volatiles removed under vacuum. Purification by FCC (30% EtOAc in 40-60 petroleum ether) afforded (3-fluoro-5-methylene-2-phenyl-3,4,5,6-tetrahydropyridin-3-yl)methanol (**7**) as a yellow oil (21 mg, 50%).

^{1}H NMR (400 MHz, CDCl$_3$) δ 7.68 (d, *J* = 7.5 Hz, 2H), 7.43 – 7.33 (m, 3H), 5.04 (s, 2H), 4.61 – 4.52 (m, 1H), 4.40 (d, *J* = 20.5 Hz, 1H), 3.90 – 3.82 (m, 1H), 3.65 (dd, *J* = 25.5, 12.5 Hz, 1H), 3.06 (t, *J* = 13.0 Hz, 1H), 2.69 (t, *J* = 14.2 Hz, 1H), 2.02 (s, 1H); **^{13}C NMR (101 MHz, CDCl$_3$)** δ 165.6 (d, $^2J_{C-F}$ = 18.5 Hz), 139.1 (d, $^3J_{C-F}$ = 8.0 Hz), 137.5, 129.7, 128.3, 128.3, 111.9, 95.6 (d, $^1J_{C-F}$ = 184.5 Hz), 64.9 (d, $^2J_{C-F}$ = 26.0 Hz), 56.5, 37.0 (d, $^2J_{C-F}$ = 23.5 Hz); **^{19}F NMR (377 MHz, CDCl$_3$)** δ -155.8 – -156.0 (m); **FTIR:** ν_{max}/cm^{-1} (neat) 3241, 2926, 1628, 1445, 1325, 1090, 1011, 902 cm^{-1}; **HRMS (ESI$^+$):** calculated for $C_{13}H_{15}FNO$ (ES$^+$)(+H$^+$): 220.1132. Found: 220.1136.

5-fluoro-3-methylene-6-phenyl-2,3,4,5-tetrahydropyridine (8)

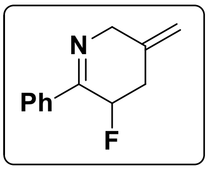

To a pressure tube containing 3-fluoro-5-methylene-2-(phenyl)-3,4,5,6-tetrahydropyridine-3-carboxylate (**4a**) (50 mg, 0.19 mmol) was added aq. HCl (6.0 M, 0.48 mL) and the resulting mixture heated at 100 °C for 1.5 h. After cooling to room temperature the reaction was basified to pH 8 with sat. $NaHCO_3$ and extracted with EtOAc (3 x 25 mL). The combined organic layers were then dried over anhydrous $MgSO_4$ and concentrated under vacuum to afford 5-fluoro-3-methylene-6-phenyl-2,3,4,5-tetrahydropyridine (**8**) as a yellow oil (0.38 g, quant.)(**CAUTION**: this compound decomposes by eliminating HF under prolonged heating (>30 °C) under vacuum).

^{1}H NMR (400 MHz, CDCl$_3$) δ 7.86 – 7.79 (m, 2H), 7.45 – 7.39 (m, 3H), 5.53 (dt, *J* = 48.5, 5.0 Hz, 1H), 5.07 (s, 1H), 5.03 (s, 1H), 4.55 (dd, *J* = 20.5, 5.5 Hz, 1H), 4.45 (dd, *J* = 20.5, 5.5 Hz, 1H), 2.90 – 2.70 (m, 2H); **^{13}C NMR (101 MHz, CDCl$_3$)** δ 162.7 (d, $^{2}J_{C\text{-}F}$ = 16.5 Hz), 137.7 (d, $^{3}J_{C\text{-}F}$ = 4.0 Hz), 137.3, 130.3, 128.5, 127.0 (d, $^{4}J_{C\text{-}F}$ = 1.5 Hz), 112.0, 84.5 (d, $^{1}J_{C\text{-}F}$ = 177.5 Hz), 56.3 (d, $^{4}J_{C\text{-}F}$ = 2.0 Hz), 35.7 (d, $^{2}J_{C\text{-}F}$ = 22.0 Hz); **^{19}F NMR (376 MHz, CDCl$_3$)** δ -170.5 – -170.9 (m); **FTIR:** ν_{max}/cm^{-1} (neat) 3061, 2923, 1632, 1447, 1319, 1015, 899 cm^{-1}; **HRMS (ESI$^+$):** calculated for $C_{12}H_{13}FN$ (ES$^+$)(+H$^+$): 190.1027. Found: 190.1034.

3-fluoro-5-methylene-2-phenylpiperidine (9)

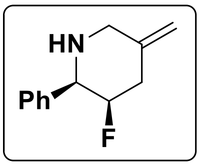

To a solution of 5-fluoro-3-methylene-6-phenyl-2,3,4,5-tetrahydropyridine (**8**) (50 mg, 0.26 mmol) in MeOH (0.52 mL) under nitrogen at 0 °C was added $NaBH_4$ (20 mg, 0.52 mmol) and the resulting mixture warmed to room temperature and stirred overnight. The reaction was then diluted with $NaHCO_3$ (10 mL) and extracted with EtOAc (5 x 10 mL). The combined organic layers were dried over anhydrous $MgSO_4$, concentrated under vacuum and the residue purified by FCC (40% EtOAc in 40-60 petroleum ether) to afford (2R,3R)-3-fluoro-5-methylene-2-phenylpiperidine (**9**) as a white solid (39 mg, 77%).

^{1}H NMR (400 MHz, CDCl$_3$) δ 7.39 – 7.21 (m, 5H), 4.97 – 4.79 (m, 3H), 3.87 (d, *J* = 30.0 Hz, 1H), 3.63 (dd, *J* = 14.0, 1.5 Hz, 1H), 3.46 (d, *J* = 14.0 Hz, 1H), 2.84 – 2.75 (m, 1H), 2.56 (dd, *J* = 45.0, 15.0 Hz, 1H), 1.93 (s, 1H); **^{13}C NMR (101 MHz, CDCl$_3$)** δ 140.3, 140.0 128.5, 127.6, 127.2, 112.1, 90.4 (d, $^{1}J_{C\text{-}F}$ = 178.5 Hz), 62.7 (d, $^{2}J_{C\text{-}F}$ = 19.0 Hz), 53.1, 38.9 (d, $^{2}J_{C\text{-}F}$ = 23.5 Hz); **^{19}F NMR (377 MHz, CDCl$_3$)** δ -197.47 (dddd, *J* = 48.5, 45.0, 30.0, 11.0 Hz); **FTIR:** ν_{max}/cm^{-1} (neat) 3291, 2937, 1656, 1467, 1261, 1100, 1022 cm^{-1}; **HRMS (ESI$^+$):** calculated for $C_{12}H_{15}FN$ (ES$^+$)(+H$^+$): 192.1183. Found: 192.1186.

ethyl 5-(4-acetoxybutylidene)-3-fluoro-2-phenyl-3,4,5,6-tetrahydropyridine-3-carboxylate (10)

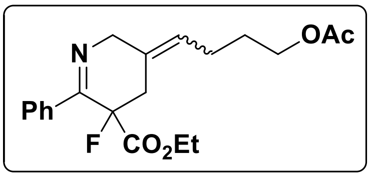

To a solution of Hoveyda-Grubbs Catalyst® 2nd Generation (6.0 mg, 9.6 µmol (5 mol%) in anhydrous, degassed DCM (0.76 mL) was added 3-fluoro-5-methylene-2-(phenyl)-3,4,5,6-tetrahydropyridine-3-carboxylate (**4a**) (50 mg, 0.19 mmol) and pent-4-en-1-yl acetate (**s2**) (68 mg, 0.57 mmol) and the resulting mixture stirred at 25 °C overnight (open to nitrogen). DCM (0.76 mL) was then added and the reaction mixture heated at reflux for 4 hours (sealed). Removal of the volatiles under vacuum and purification by FCC (20% EtOAc in 40-60 petroleum ether) afforded ethyl 5-(4-acetoxybutylidene)-3-fluoro-2-phenyl-3,4,5,6-tetrahydropyridine-3-carboxylate (**11**) as an orange oil (38 mg, 54%).

^{1}H NMR (400 MHz, CDCl$_3$) δ 7.72 – 7.63 (m, 2H), 7.43 – 7.30 (m, 3H), 5.59 (t, *J* = 7.0 Hz, 1H, **minor**), 5.36 (t, *J* = 7.5 Hz, 1H, **major**), 4.81 – 4.41 (m, 2H), 4.21 – 4.00 (m, 4H), 3.08 – 2.76 (m, 2H), 2.25 –

2.11 (m, 2H), 2.09 – 2.02 (m, 3H), 1.78 – 1.65 (m, 2H), 1.08 – 0.98 (m, 2H); **^{19}F NMR (377 MHz, $CDCl_3$)** δ -145.47 (ddt, *J* = 24.0, 18.0, 6.0 Hz, **minor**), -147.72 (ddt, *J* = 21.3, 16.5, 4.9 Hz, **major**); **FTIR:** ν_{max}/cm^{-1} (neat) 2962, 1733, 1637, 1447, 1367, 1236, 1041 cm^{-1}; **HRMS (ESI$^+$):** calculated for $C_{20}H_{25}FNO_4$ (ES$^+$)(+H$^+$): 362.1762. Found: 362.1774.

^{13}C NMR MAJOR (101 MHz, $CDCl_3$) δ 171.3, 169.2 (d, $^2J_{C-F}$ = 27.0 Hz), 160.9 (d, $^2J_{C-F}$ = 18.5 Hz), 136.9, 128.4, 127.4, 127.3, 127.2, 126.8, 90.8 (d, $^1J_{C-F}$ = 195.5 Hz), 63.7, 62.3, 51.3, 40.4 (d, $^2J_{C-F}$ = 24.0 Hz), 28.4, 23.7, 21.1, 13.9.

^{13}C NMR MINOR (101 MHz, $CDCl_3$) δ 171.3, 169.4 (d, $^2J_{C-F}$ = 26.5 Hz), 160.0 (d, $^2J_{C-F}$ = 18.0 Hz), 137.0, 130.0, 127.4, 127.2, 127.2, 126.4, 90.8 (d, $^1J_{C-F}$ = 195.5 Hz), 63.8, 62.4, 57.6, 34.0 (d, $^2J_{C-F}$ = 24.0 Hz), 28.6, 23.4, 21.1, 13.8.

5-methylene-2-phenyl-3-((trifluoromethyl)thio)piperidine (13a)

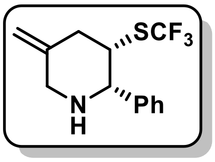

Following GPG using *tert*-butyl (2-methylene-5-oxo-5-phenyl-4-((trifluoromethyl)thio)pentyl)carbamate (**12a**) (312 mg, 0.801 mmol), TFA (6.8 g, 60.1 mmol) and $NaBH_4$ (121 mg, 3.20 mmol) with FCC (20% EtOAc in 40-60 petroleum ether) gave 5-methylene-2-phenyl-3-((trifluoromethyl)thio) piperidine (**13a**) as a white solid (166 mg, 76%).

^{1}H NMR (400 MHz, $CDCl_3$): δ 7.40 - 7.27 (m, 5H), 5.07 (s, 1H), 4.96 (s, 1H), 4.25 (s, 1H), 3.70 - 3.63 (m, 2H), 3.49 (d, *J* = 12.5 Hz, 1H), 2.90 (d, *J* = 14.0 Hz, 1H), 2.81 (d, *J* = 14.0 Hz, 1H), 2.12 (br, 1H); **^{13}C NMR (101 MHz, $CDCl_3$)** δ 140.2, 138.3, 131.3 (q, $^1J_{C-F}$ = 306 Hz), 128.3, 127.8, 126.8, 113.8, 63.7, 53.2, 50.6, 41.6; **^{19}F NMR (377 MHz, $CDCl_3$)**: δ -40.3 (s); **FTIR:** ν_{max}/cm^{-1} (neat) 3311, 2991, 2956, 2786, 1662, 1493, 1452, 1347, 1275, 1261, 1104, 1030, 954, 907, 764, 750, 699; **HRMS (ESI$^+$):** *m/z* calcd. for $C_{13}H_{14}F_3NS$: 273.0794, found: 273.0788 [*M*]$^+$.

2-(4-methoxyphenyl)-5-methylene-3-((trifluoromethyl)thio)piperidine (13b)

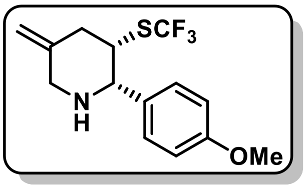

Following GPG using *tert*-butyl (5-(4-methoxyphenyl)-2-methylene-5-oxo-4-((trifluoromethyl)thio)pentyl)carbamate (**12b**) (153 mg, 0.365 mmol), TFA (3.1 g, 27.4 mmol) and $NaBH_4$ (55 mg, 1.46 mmol) with FCC (20% EtOAc in 40-60 petroleum ether) gave 2-(4-methoxyphenyl)-5-methylene-3-((trifluoromethyl)thio)piperidine (**13b**) as an amorphous white solid (79 mg, 71%).

^{1}H NMR (400 MHz, $CDCl_3$): δ 7.26 (d, *J* = 8.5 Hz, 2H), 6.88 (d, *J* = 8.5 Hz, 2H), 5.05 (s, 1H), 4.94 (s, 1H), 4.18 (s, 1H), 3.80 (s, 3H), 3.65 (d, *J* = 12.5 Hz, 1H), 3.62 - 3.59 (m, 1H), 3.48 (d, *J* = 12.5 Hz, 1H), 2.88 (d, *J* = 13.5 Hz, 1H), 2.79 (d, *J* = 13.5 Hz, 1H), 2.11 (br, 1H); **^{13}C NMR (101 MHz, $CDCl_3$)** δ 159.1, 138.6, 132.6, 131.4 (q, $^1J_{C-F}$ = 306 Hz), 127.9, 113.6, 113.5, 63.3, 55.2, 53.4, 50.8, 41.5; **^{19}F NMR (377 MHz, $CDCl_3$)**: δ -40.2 (s); **FTIR:** ν_{max}/cm^{-1} (neat) 3006, 2984, 2958, 2838, 2777, 1661, 1613, 1514, 1443, 1347, 1276, 1254, 1108, 1033, 905, 822, 764, 750; **HRMS (ESI$^+$):** *m/z* calcd. for $C_{14}H_{17}F_3NOS$: 304.0977, found: 304.0993 [*M+H*]$^+$.

5-methylene-2-(p-tolyl)-3-((trifluoromethyl)thio)piperidine (13c)

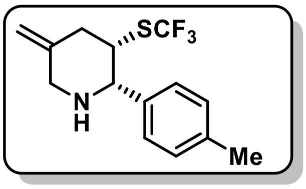

Following GPG using *tert*-butyl (2-methylene-5-oxo-5-(p-tolyl)-4-((trifluoromethyl)thio)pentyl)carbamate (**12c**) (125 mg, 0.310 mmol), TFA (2.6 g, 23.2 mmol) and $NaBH_4$ (47 mg, 1.24 mmol) with FCC (20% EtOAc in 40-60 petroleum ether) gave 5-methylene-2-(p-tolyl)-3-

((trifluoromethyl)thio)piperidine (**13c**) as an amorphous white solid (64 mg, 72%).

^{1}H NMR (400 MHz, CDCl$_3$): δ 7.22 (d, *J* = 8.0 Hz, 2H), 7.16 (d, *J* = 8.0 Hz, 2H), 5.05 (s, 1H), 4.94 (s, 1H), 4.20 (s, 1H), 3.68 - 3.61 (m, 2H), 3.48 (d, *J* = 12.5 Hz, 1H), 2.88 (d, *J* = 14.0 Hz, 1H), 2.80 (d, *J* = 14.0 Hz, 1H), 2.12 (br, 1H); **^{13}C NMR (101 MHz, CDCl$_3$)** δ 138.6, 137.4, 137.3, 131.4 (q, $^{1}J_{C-F}$ = 306 Hz), 128.9, 126.6, 113.5, 63.5, 53.3, 50.7, 41.5, 21.1; **^{19}F NMR (377 MHz, CDCl$_3$)**: δ -40.2 (s); **FTIR:** ν_{max}/cm^{-1} (neat) 3006, 2958, 2838, 2777, 1661, 1613, 1514, 1443, 1347, 1276, 1254, 1108, 1033, 905, 822, 764, 750; **HRMS (ESI$^+$):** *m/z* calcd. for $C_{14}H_{17}F_3NS$: 288.1028, found: 288.1042 [*M+H*]$^+$.

2-(4-chlorophenyl)-5-methylene-3-((trifluoromethyl)thio)piperidine (13d)

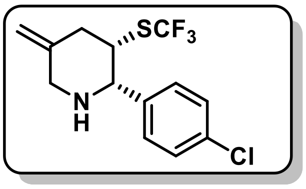

Following GPG using *tert*-butyl (5-(4-chlorophenyl)-2-methylene-5-oxo-4-((trifluoromethyl)thio)pentyl)carbamate (**12d**) (174 mg, 0.410 mmol), TFA (3.5 g, 30.8 mmol) and NaBH$_4$ (62 mg, 1.64 mmol) with FCC (20% EtOAc in 40-60 petroleum ether) gave 2-(4-chlorophenyl)-5-methylene-3-((trifluoromethyl)thio) (**13d**) as a colourless oil (68 mg, 54%).

^{1}H NMR (400 MHz, CDCl$_3$): δ 7.35 - 7.27 (m, 4H), 5.06 (s, 1H), 4.95 (s, 1H), 4.20 (s, 1H), 3.64 (d, *J* = 12.5 Hz, 1H), 3.61 - 3.58 (m, 1H), 3.47 (d, *J* = 12.5 Hz, 1H), 2.88 (d, *J* = 14.0 Hz, 1H), 2.80 (d, *J* = 14.0 Hz, 1H), 2.12 (br, 1H); **^{13}C NMR (101 MHz, CDCl$_3$)** δ 139.0, 138.2, 133.5, 131.3 (q, $^{1}J_{C-F}$ = 306 Hz), 128.4, 128.2, 113.8, 63.2, 53.2, 50.5, 41.6; **^{19}F NMR (377 MHz, CDCl$_3$)**: δ -40.4 (s); **FTIR:** ν_{max}/cm^{-1} (neat) 3008, 2989, 2954, 2794, 1661, 1493, 1447, 1276, 1261, 1110, 1014, 906, 812, 764, 750; **HRMS (ESI$^+$):** *m/z* calcd. for $C_{13}H_{14}{}^{35}ClF_3NS$: 308.0482, found: 308.0497 [*M+H*]$^+$.

2-(2-methoxyphenyl)-5-methylene-3-((trifluoromethyl)thio)piperidine (13e)

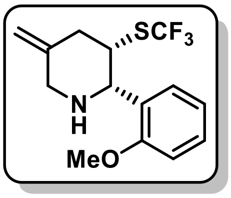

Following GPG *tert*-butyl (5-(2-methoxyphenyl)-2-methylene-5-oxo-4-((trifluoromethyl)thio)pentyl)carbamate (**12e**) (162 mg, 0.386 mmol), TFA (3.3 g, 29.0 mmol) and NaBH$_4$ (58 mg, 1.54 mmol) with FCC (20% EtOAc in 40-60 petroleum ether) gave 2-(2-methoxyphenyl)-5-methylene-3-((trifluoromethyl)thio)piperidine (**13e**) as a white solid (66 mg, 56%).

^{1}H NMR (400 MHz, CDCl$_3$): δ 7.32 (d, *J* = 7.5 Hz, 1H), 7.27 (t, *J* = 8.0 Hz, 1H), 6.97 (t, *J* = 7.5 Hz, 1H), 6.87 (d, *J* = 8.0 Hz, 1H), 5.03 (s, 1H), 4.92 (s, 1H), 4.48 (s, 1H), 3.96 - 3.91 (m, 1H), 3.86 (s, 3H), 3.65 (d, *J* = 12.5 Hz, 1H), 3.49 (d, *J* = 12.5 Hz, 1H), 2.91 (d, *J* = 14.0 Hz, 1H), 2.75 (d, *J* = 14.0 Hz, 1H), 1.67 (br, 1H); **^{13}C NMR (101 MHz, CDCl$_3$)** δ 156.1, 139.6, 131.5 (q, $^{1}J_{C-F}$ = 307 Hz), 128.8, 128.4, 127.3, 120.4, 112.9, 109.9, 57.9, 55.3, 53.7, 47.9, 41.5; **^{19}F NMR (377 MHz, CDCl$_3$)**: δ -40.4 (s); **FTIR:** ν_{max}/cm^{-1} (neat) 3008, 2989, 2957, 2839, 2777, 1661, 1603, 1493, 1466, 1348, 1276, 1260, 1108, 1028, 903, 764, 750; **HRMS (ESI$^+$):** *m/z* calcd. for $C_{14}H_{17}F_3NOS$: 304.0977, found: 304.0976 [*M+H*]$^+$.

2-(3-chlorophenyl)-5-methylene-3-((trifluoromethyl)thio)piperidine (13f)

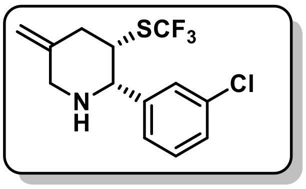

Following GPG using *tert*-butyl (5-(3-chlorophenyl)-2-methylene-5-oxo-4-((trifluoromethyl)thio)pentyl)carbamate (**12f**) (184 mg, 0.434 mmol), TFA (3.7 g, 32.6 mmol) and NaBH$_4$ (66 mg, 1.74 mmol) with FCC (20% EtOAc in 40-60 petroleum ether) gave 2-(3-chlorophenyl)-5-methylene-3-((trifluoromethyl)thio)piperidine (**13f**) as a yellow oil (47 mg, 35%).

^{1}H NMR (400 MHz, CDCl$_3$): δ 7.36 - 7.20 (m, 4H), 5.06 (s, 1H), 4.94 (s, 1H), 4.19 (s, 1H), 3.67 - 3.60 (m, 2H), 3.46 (d, *J* = 12.5 Hz, 1H), 2.88 (d, *J* = 14.0 Hz, 1H), 2.79 (d, *J* = 14.0 Hz, 1H), 1.70 (br, 1H); **^{13}C NMR (101 MHz, CDCl$_3$)** δ 142.7, 138.4, 134.2, 131.3 (q, $^{1}J_{C-F}$ = 306 Hz), 129.8, 127.9, 127.1, 125.0, 113.6, 63.3, 53.2, 50.4, 41.6; **^{19}F NMR (377 MHz, CDCl$_3$)**: δ -40.4 (s); **FTIR:** ν_{max}/cm^{-1} (neat) 3008,

2989, 2954, 2803, 1661, 1478, 1447, 1276, 1261, 1100, 1035, 907, 848, 764, 750; **HRMS (ESI⁺):** *m/z* calcd. for $C_{13}H_{14}{}^{35}ClF_3NS$: 308.0482, found: 308.0477 $[M+H]^+$.

5-methylene-2-(thiophen-2-yl)-3-((trifluoromethyl)thio)piperidine (13g)

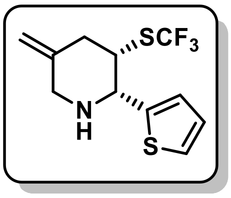

Following GPG *tert*-butyl (2-methylene-5-oxo-5-(thiophen-2-yl)-4-((trifluoromethyl)thio)pentyl)carbamate (**12g**) (124 mg, 0.313 mmol), TFA (2.7 g, 23.5 mmol) and $NaBH_4$ (47 mg, 1.25 mmol) with FCC (20% EtOAc in 40-60 petroleum ether) gave 5-methylene-2-(thiophen-2-yl)-3-((trifluoromethyl)thio) piperidine (**13g**) as an amorphous white solid (56 mg, 64%).

^{1}H NMR (400 MHz, $CDCl_3$): δ 7.26 - 7.25 (m, 1H), 7.06 - 6.98 (m, 2H), 5.04 (s, 1H), 4.95 (s, 1H), 4.52 (s, 1H), 3.71 (dd, *J* = 6.0 Hz, 3.0 Hz, 1H), 3.61 (d, *J* = 13.0 Hz, 1H), 3.48 (d, *J* = 13.0 Hz, 1H), 2.86 (d, *J* = 14.0 Hz, 1H), 2.80 (dd, *J* = 14.0 Hz, 3.0 Hz, 1H), 1.89 (br, 1H); **^{13}C NMR (101 MHz, $CDCl_3$)** δ 143.5, 138.4, 131.4 (q, $^1J_{C-F}$ = 306 Hz), 126.6, 124.7, 124.6, 113.8, 60.0, 52.9, 50.5, 41.0; **^{19}F NMR (377 MHz, $CDCl_3$):** δ -40.1 (s); **FTIR:** ν_{max}/cm^{-1} (neat) 3306, 3008, 2991, 2961, 2798, 1664, 1435, 1311, 1276, 1261, 1121, 1103, 949, 906, 764, 750, 702; **HRMS (ESI⁺):** *m/z* calcd. for $C_{11}H_{13}F_3NS_2$: 280.0436, found: 280.0439 $[M+H]^+$.

3-methyl-5-methylene-2-phenyl-3-((trifluoromethyl)thio)piperidine (13h)

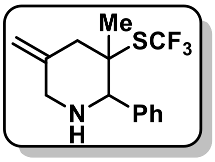

Following GPG using *tert*-butyl (4-methyl-2-methylene-5-oxo-5-phenyl-4-((trifluoromethyl)thio)pentyl)carbamate (**12h**) (122 mg, 0.302 mmol), TFA (2.6 g, 22.7 mmol) and $NaBH_4$ (46 mg, 1.21 mmol) with FCC (20% EtOAc in 40-60 petroleum ether) gave 3-methyl-5-methylene-2-phenyl-3-((trifluoromethyl)thio)piperidine (**13h**) as a yellow oil (78 mg, 90%, 5:1 d.r.). Data reported for major diastereomer only.

^{1}H NMR (400 MHz, $CDCl_3$): δ 7.42 - 7.31 (m, 5H), 5.05 (s, 1H), 4.91 (s, 1H), 3.72 - 3.67 (m, 2H), 3.48 (d, *J* = 12.0 Hz, 1H), 2.89 (d, *J* = 14.0 Hz, 1H), 2.45 (d, *J* = 14.0 Hz, 1H), 1.78 (br, 1H), 1.43 (s, 3H); **^{13}C NMR (101 MHz, $CDCl_3$)** δ 140.0, 138.4, 131.6 (q, $^1J_{C-F}$ = 307.5 Hz), 129.0, 128.3, 127.9, 112.7, 72.4, 56.8, 53.6, 48.3, 26.5; **^{19}F NMR (377 MHz, $CDCl_3$):** δ -33.5 (s); **FTIR:** ν_{max}/cm^{-1} (neat) 3033, 3005, 2988, 2785, 1661, 1494, 1455, 1340, 1275, 1261, 1096, 1074, 1032, 899, 764, 750, 700; **HRMS (ESI⁺):** *m/z* calcd. for $C_{14}H_{17}F_3NS$: 288.1028, found: 288.1015 $[M+H]^+$.

2-ethyl-5-methylene-3-((trifluoromethyl)thio)piperidine (13i)

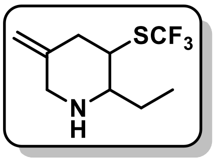

Following GPG using *tert*-butyl (2-methylene-5-oxo-4-((trifluoromethyl)thio)heptyl)carbamate (**12i**) (119 mg, 0.349 mmol), TFA (3.0 g, 26.1 mmol) and $NaBH_4$ (53 mg, 1.39 mmol) with FCC (20% EtOAc in 40-60 petroleum ether) gave 2-ethyl-5-methylene-3-((trifluoromethyl)thio)piperidine (**13i**) as a yellow oil (40 mg, 51%, 3:1 d.r.). Data reported for major diastereomer only.

^{1}H NMR (400 MHz, $CDCl_3$): δ 4.94 (s, 1H), 4.85 (s, 1H), 3.54 - 3.51 (m, 1H), 3.45 (d, *J* = 12.5 Hz, 1H), 3.30 (d, *J* = 12.5 Hz, 1H), 2.81 (t, *J* = 7.0 Hz, 1H), 2.72 - 2.63 (m, 2H), 1.74 (br, 1H), 1.64 - 1.48 (m, 2H) 0.97 (t, *J* = 7.5 Hz, 3H); **^{13}C NMR (101 MHz, $CDCl_3$)** δ 140.0, 131.9 (q, $^1J_{C-F}$ = 306 Hz), 112.6, 61.4, 52.9, 48.1, 41.4, 26.6, 10.1; **^{19}F NMR (377 MHz, $CDCl_3$)**: δ -39.4 (s); **FTIR:** ν_{max}/cm^{-1} (neat) 2966, 2939, 2880, 2791, 1661, 1437, 1275, 1261, 1141, 1099, 1004, 899, 764, 750, 704, 659; **HRMS (ESI⁺):** *m/z* calcd. for $C_9H_{15}F_3NS$: 226.0872, found: 226.0876 $[M+H]^+$.

NMR Spectra

Ethyl 2-fluoro-3-oxo-3-phenylpropanoate (1a)

^1H NMR, $CDCl_3$, 400 MHz

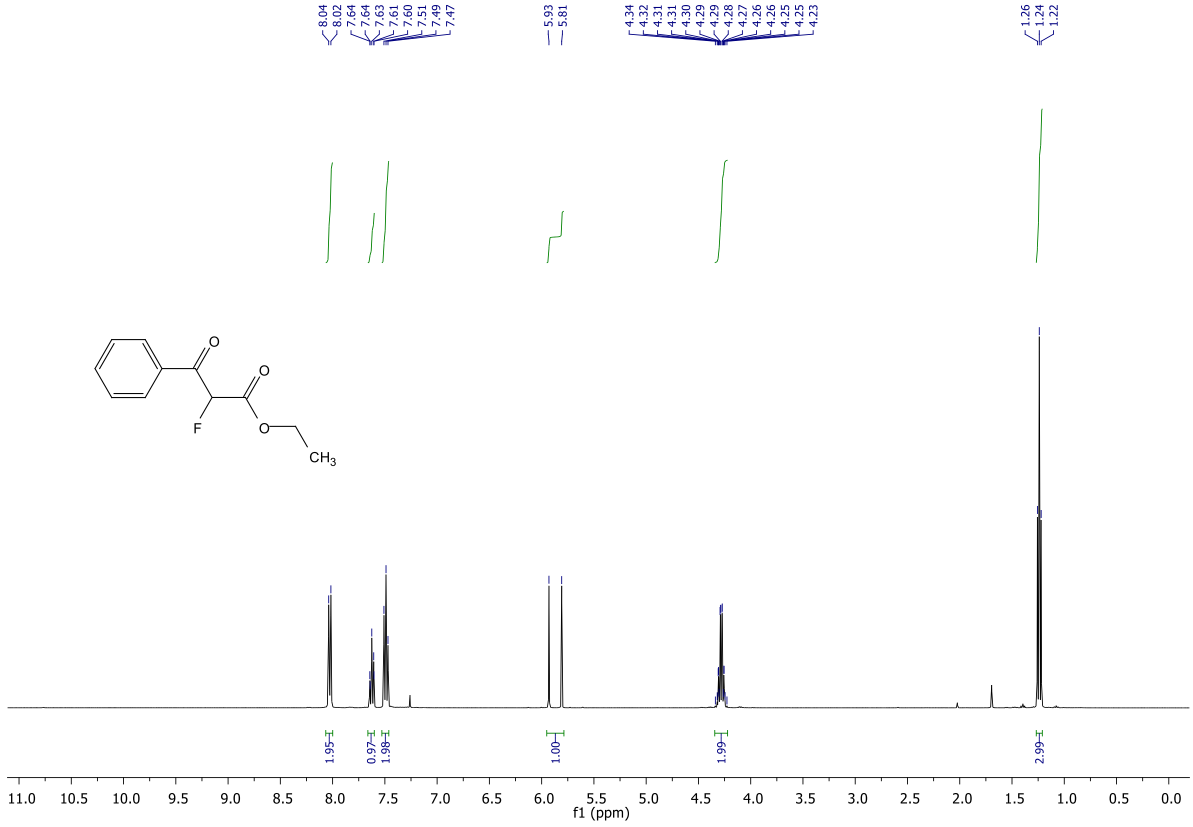

^{19}F NMR, $CDCl_3$, 377 MHz

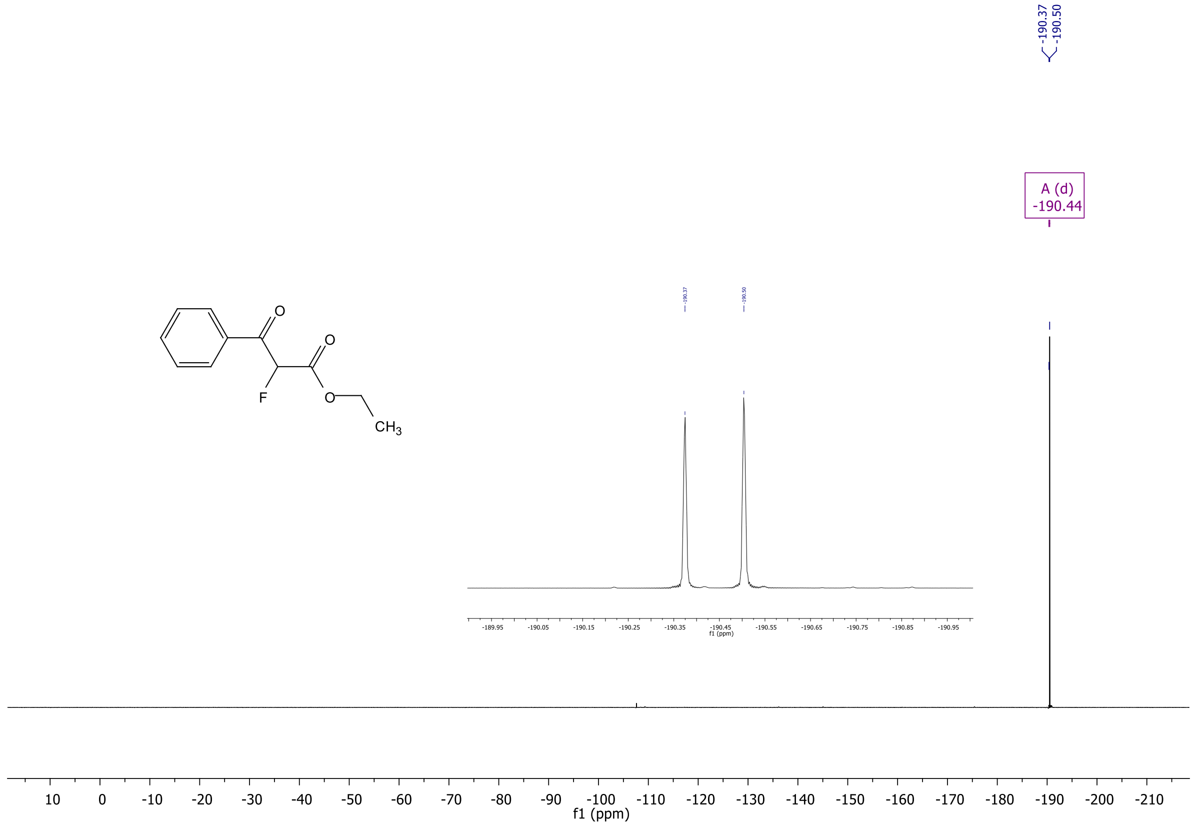

^{13}C NMR, $CDCl_3$, 101 MHz

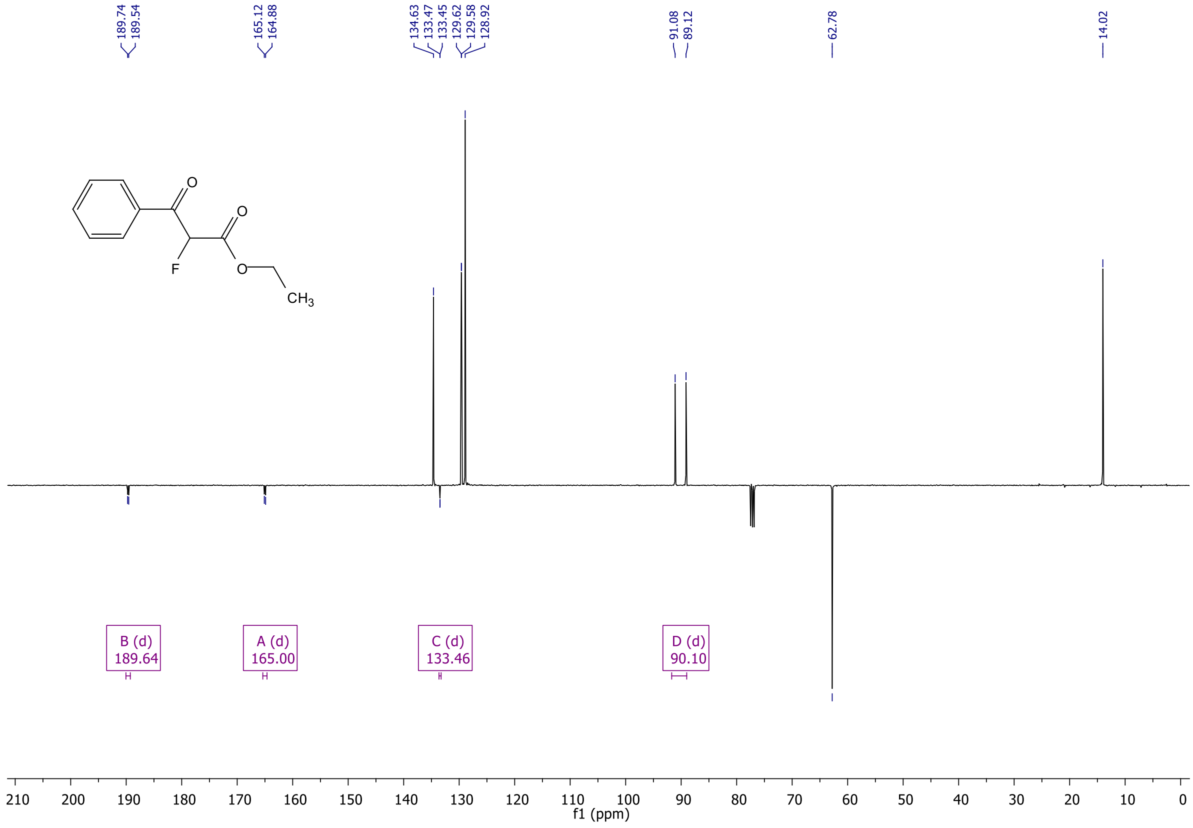

Ethyl 2-fluoro-3-(4-methoxyphenyl)-3-oxopropanoate (1b)

^{1}H NMR, $CDCl_3$, 400 MHz

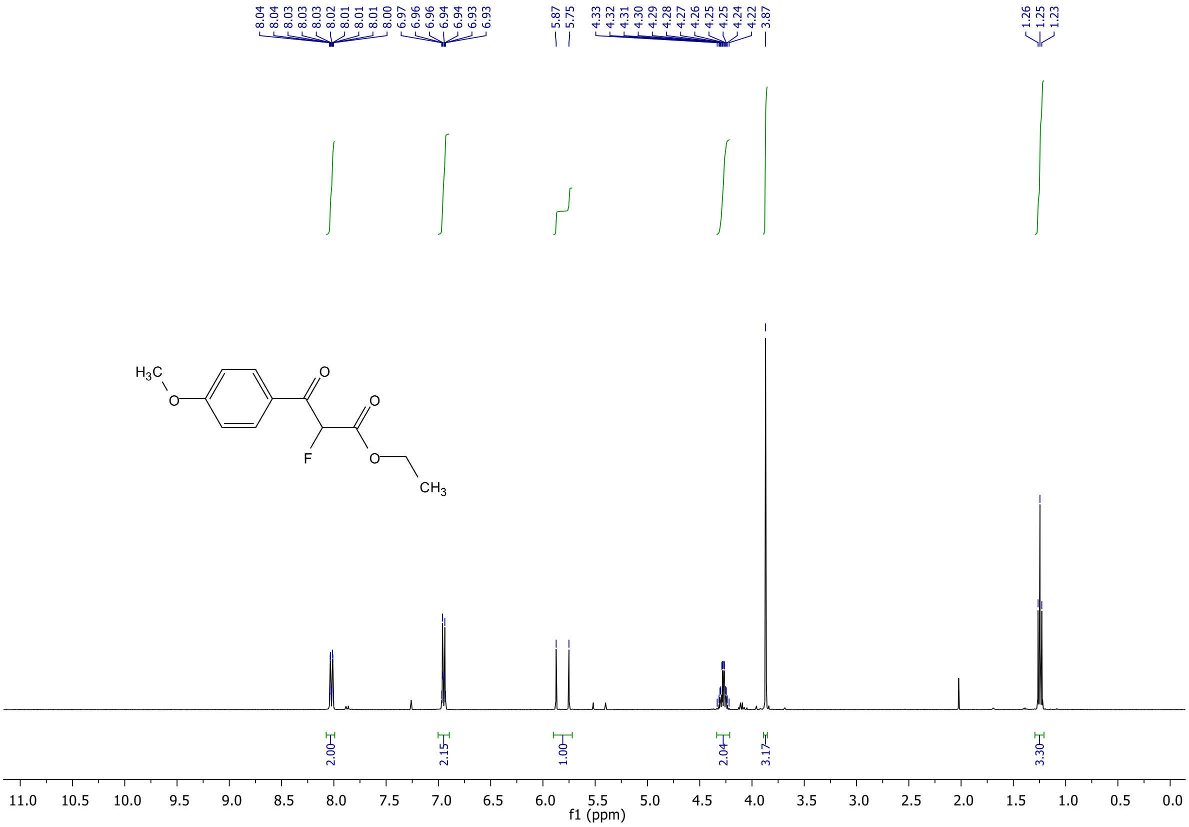

^{19}F NMR, $CDCl_3$, 377 MHz

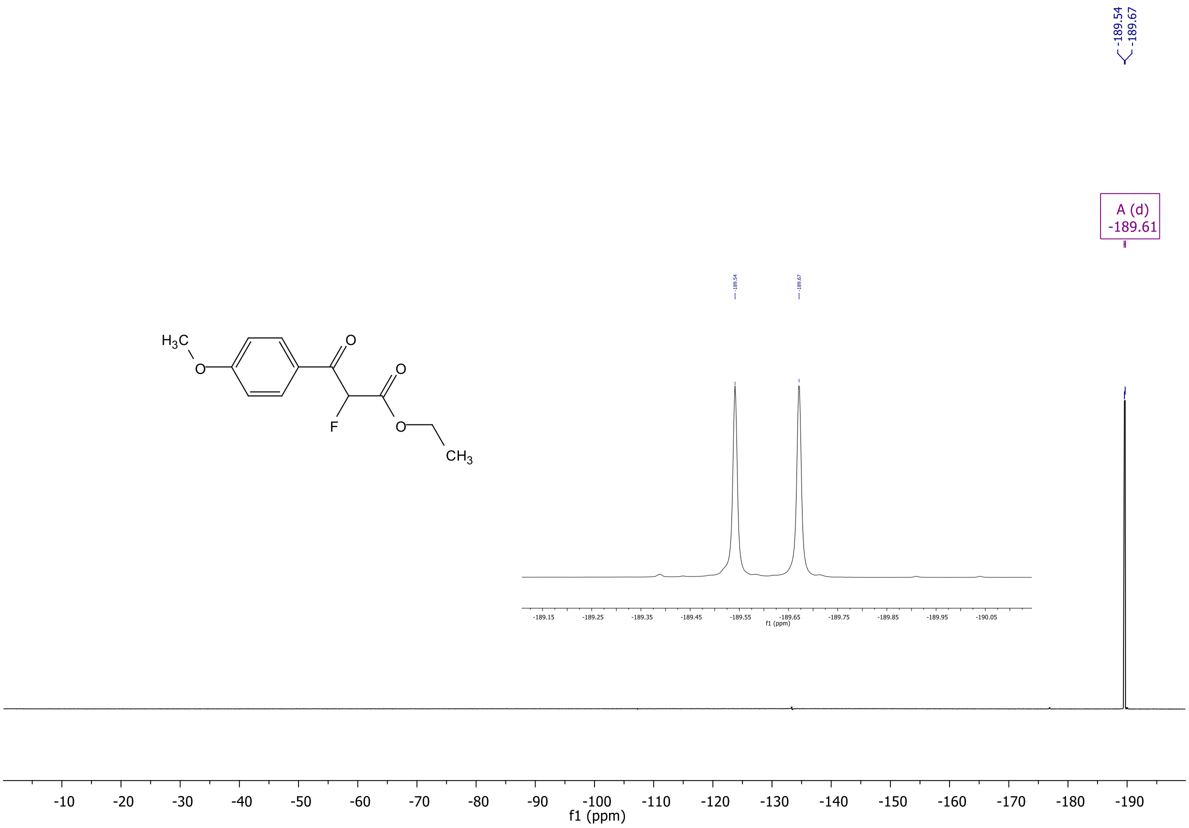

^{13}C NMR, $CDCl_3$, 101 MHz

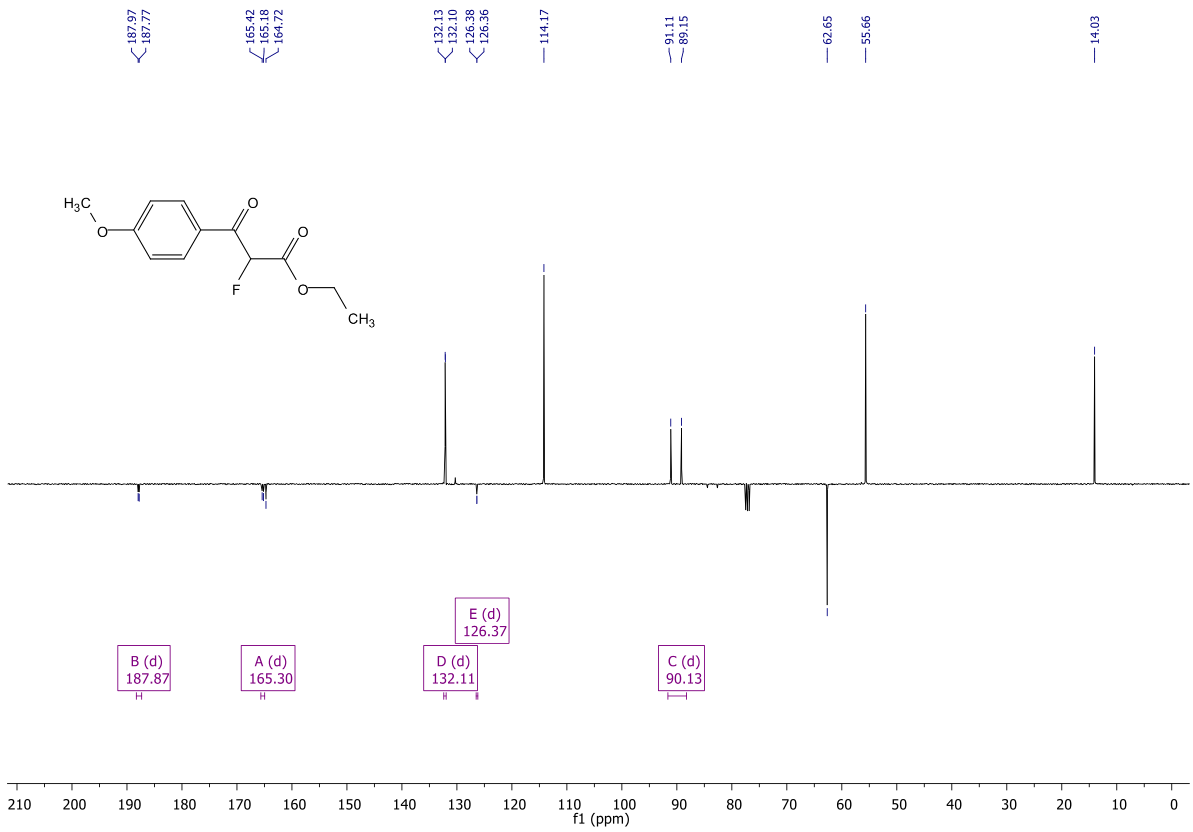
187.97
187.77
165.42
165.18
164.72
132.13
132.10
126.38
126.36
114.17
91.11
89.15
62.65
55.66
14.03
E (d)
126.37
B (d)
187.87
A (d)
165.30
D (d)
132.11
C (d)
90.13
210 200 190 180 170 160 150 140 130 120 110 100 90 80 70 60 50 40 30 20 10 0
f1 (ppm)

Ethyl 2-fluoro-3-(4-chlorophenyl)-3-oxopropanoate (1c)

^{1}H NMR, $CDCl_3$, 400 MHz

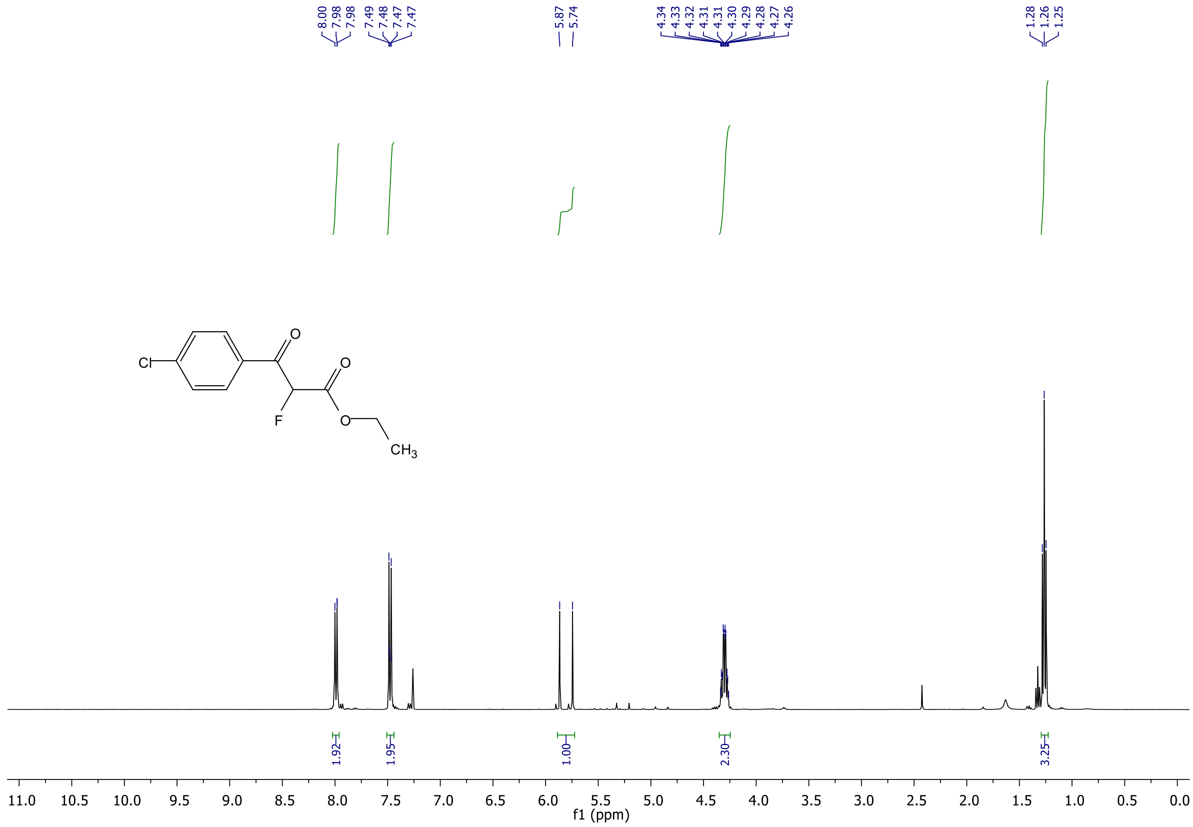

^{19}F NMR, $CDCl_3$, 377 MHz

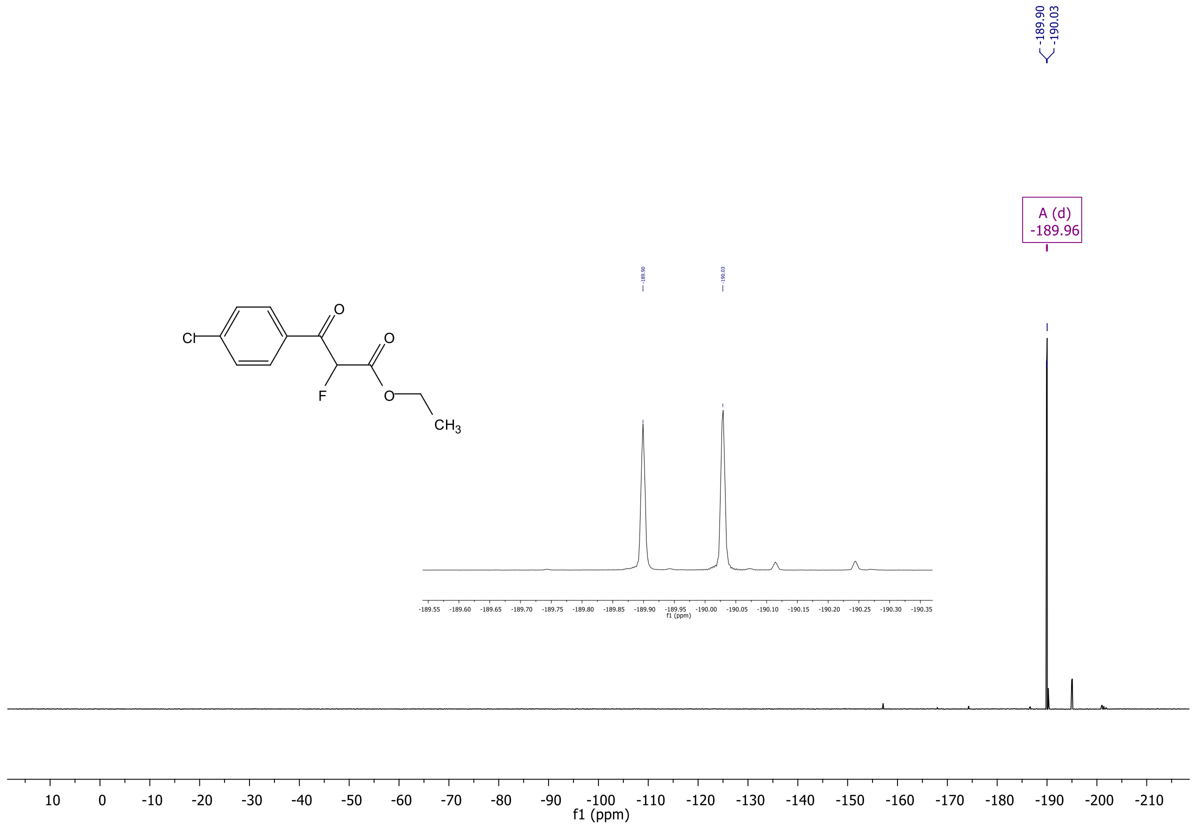

^{13}C NMR, $CDCl_3$, 101 MHz

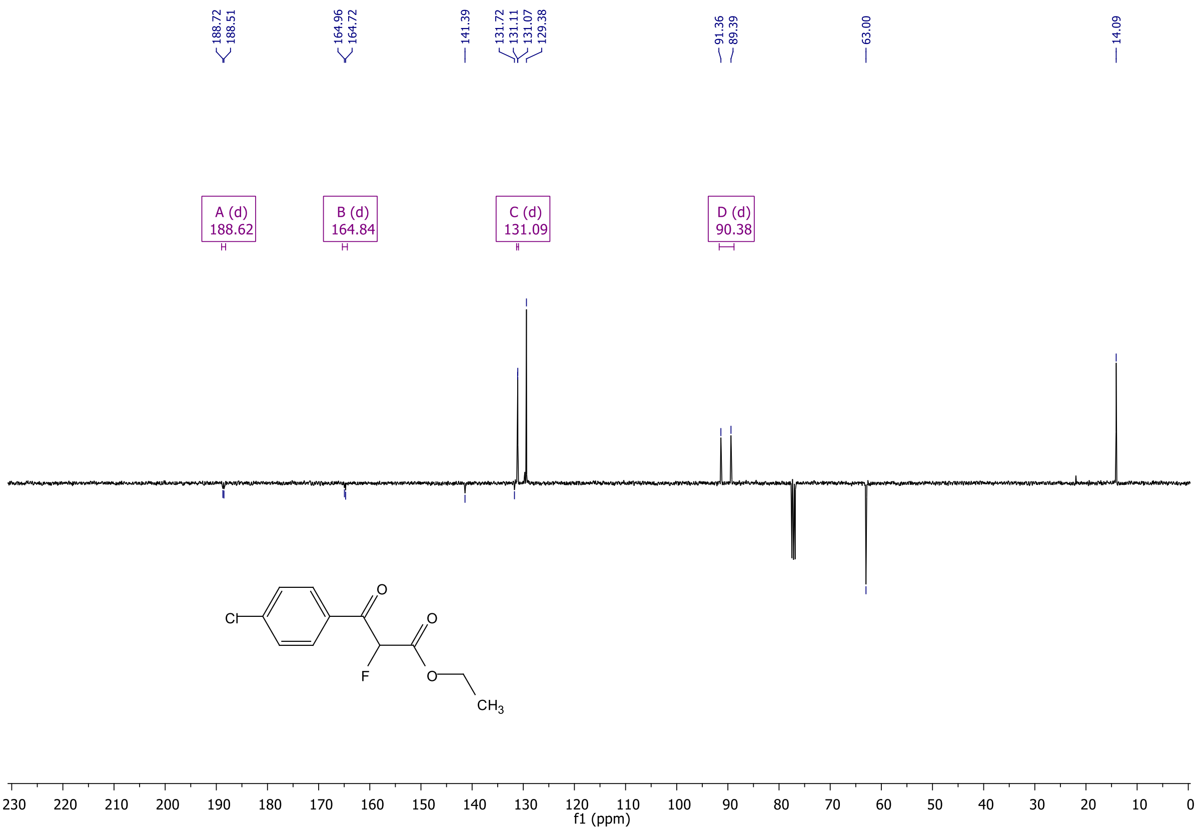

188.72
188.51
164.96
164.72
141.39
131.72
131.11
131.07
129.38
91.36
89.39
63.00
14.09
A (d)
188.62
B (d)
164.84
C (d)
131.09
D (d)
90.38
Cl
O
O
F
O
CH3
230 220 210 200 190 180 170 160 150 140 130 120 110 100 90 80 70 60 50 40 30 20 10 0
f1 (ppm)

Ethyl 2-fluoro-3-(4-trifluoromethylphenyl)-3-oxopropanoate (1d)

^{1}H NMR, $CDCl_3$, 400 MHz

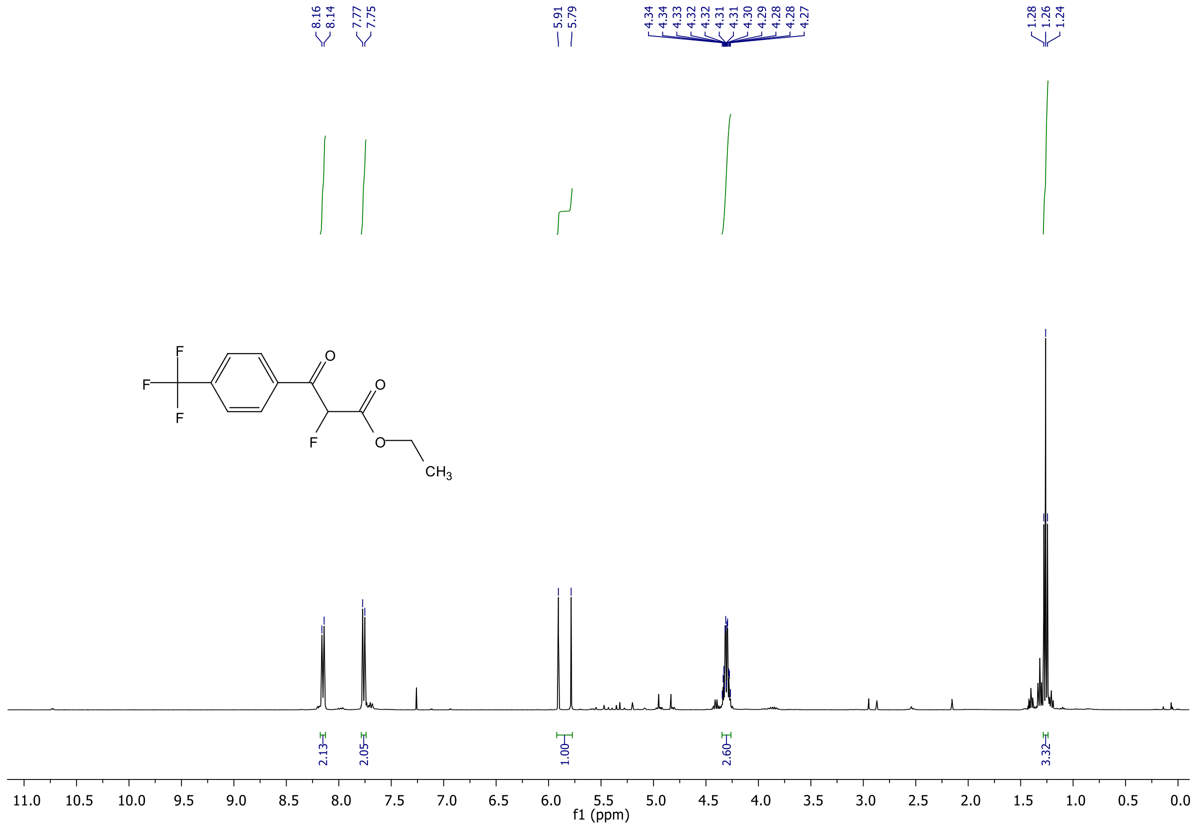

^{19}F NMR, $CDCl_3$, 377 MHz

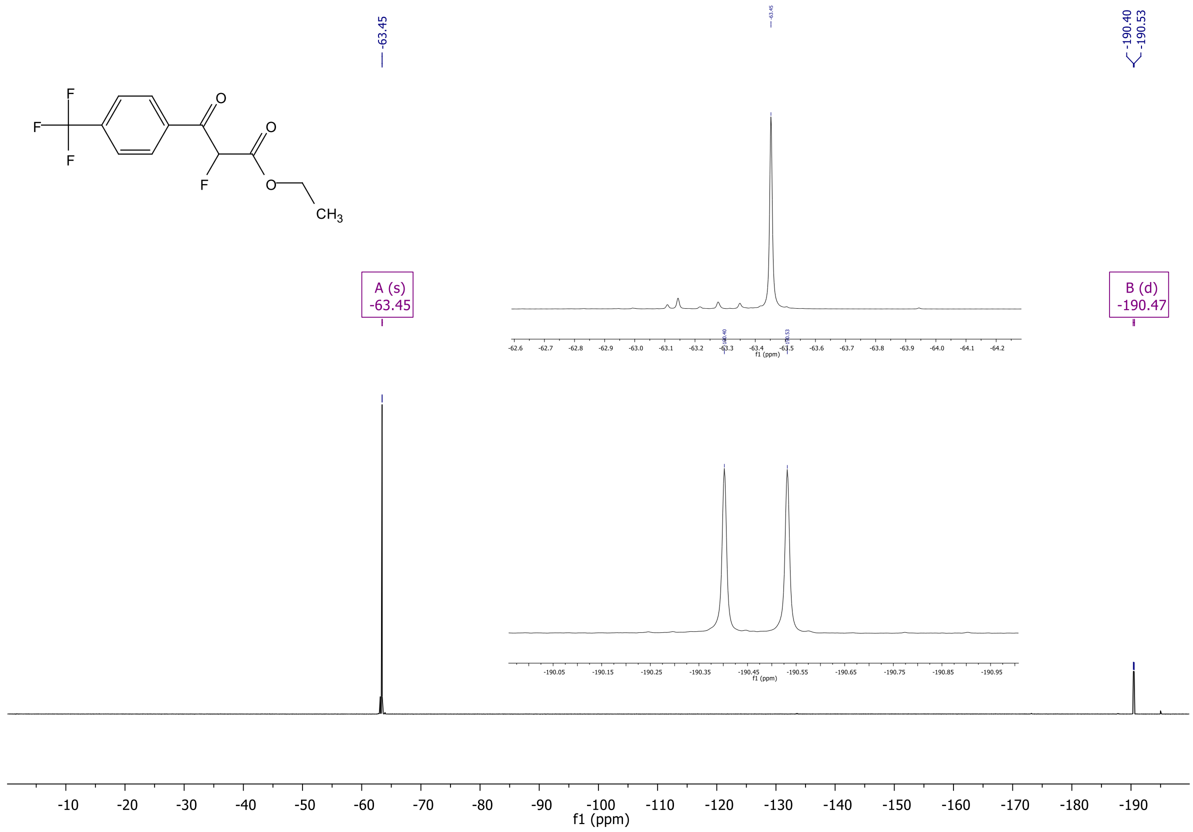

^{13}C NMR, $CDCl_3$, 101 MHz

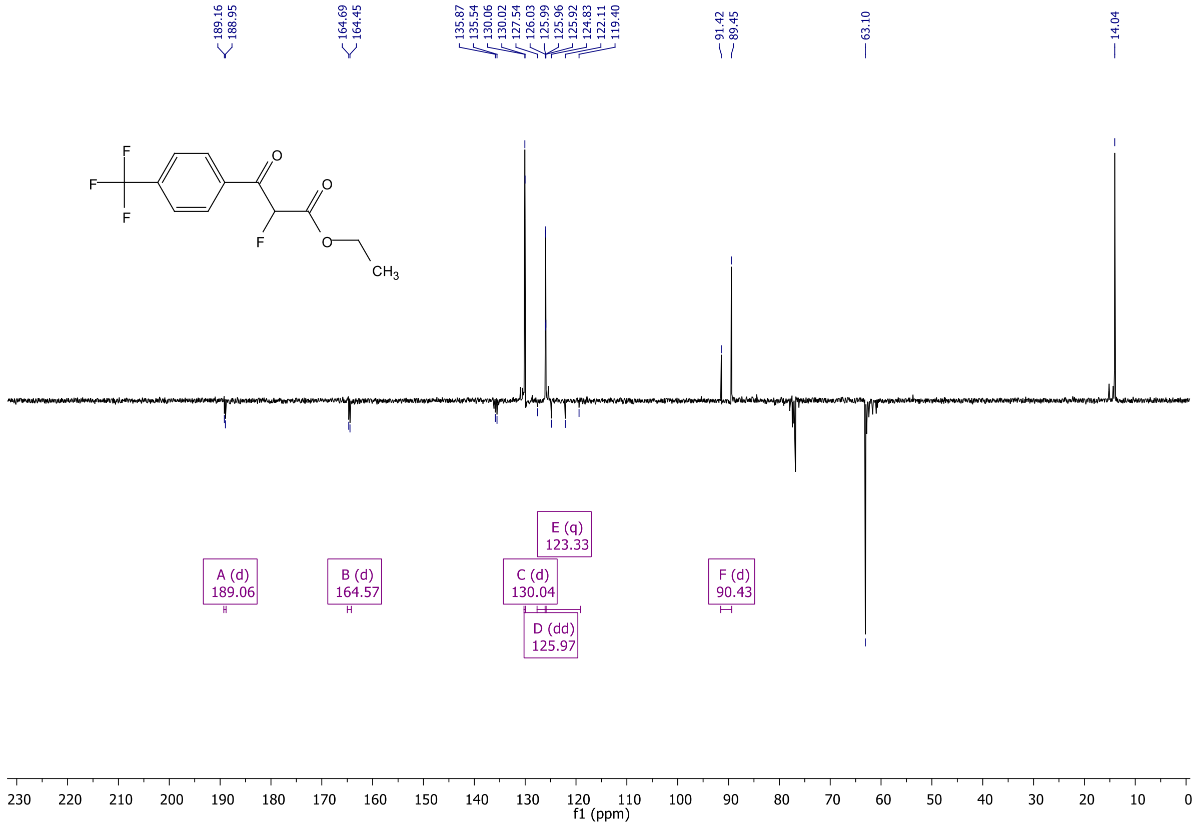

189.16
188.95
164.69
164.45
135.87
135.54
130.06
130.02
127.54
126.03
125.99
125.96
125.92
124.83
122.11
119.40
91.42
89.45
63.10
14.04
F
F
F
O
O
F
O
CH3
E (q)
123.33
A (d)
189.06
B (d)
164.57
C (d)
130.04
F (d)
90.43
D (dd)
125.97
230
220
210
200
190
180
170
160
150
140
130
120
110
100
90
80
70
60
50
40
30
20
10
0
f1 (ppm)

Ethyl 2-fluoro-3-(4-methylphenyl)-3-oxopropanoate (1e)

^1H NMR, $CDCl_3$, 400 MHz

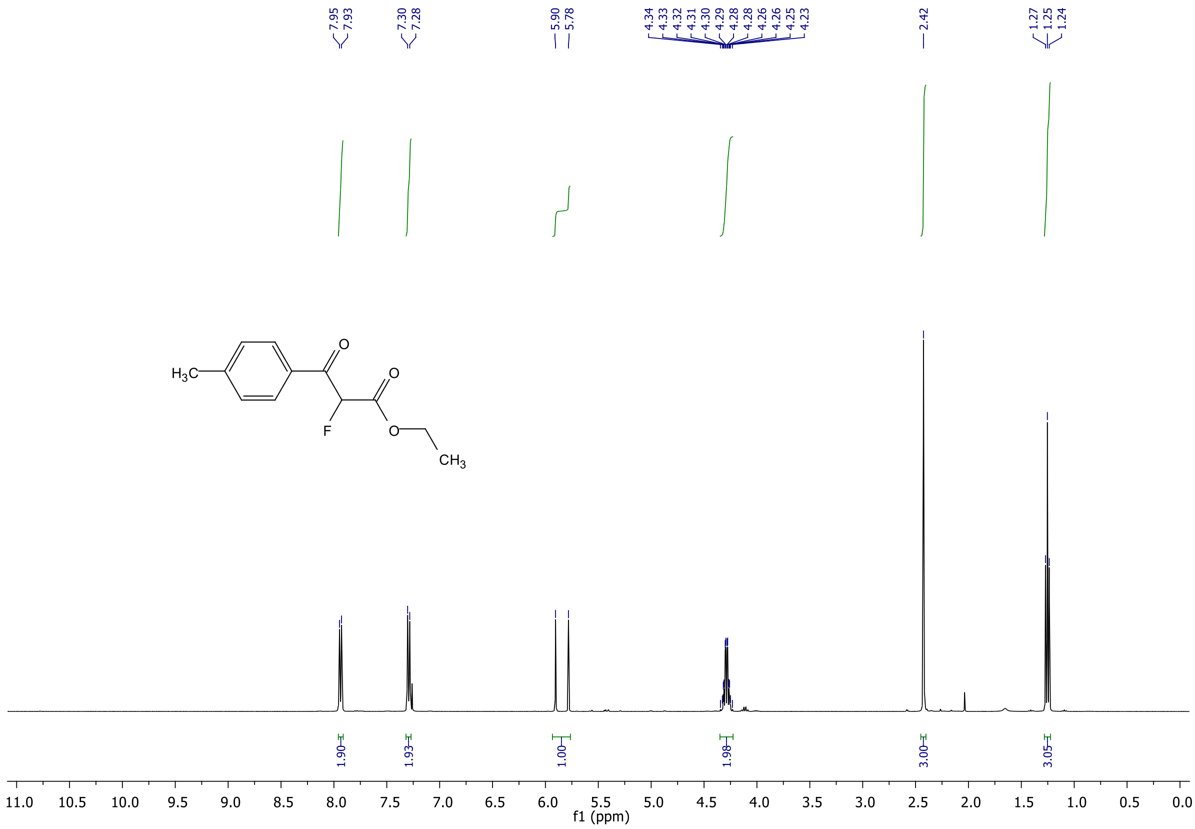

^{19}F NMR, $CDCl_3$, 377 MHz

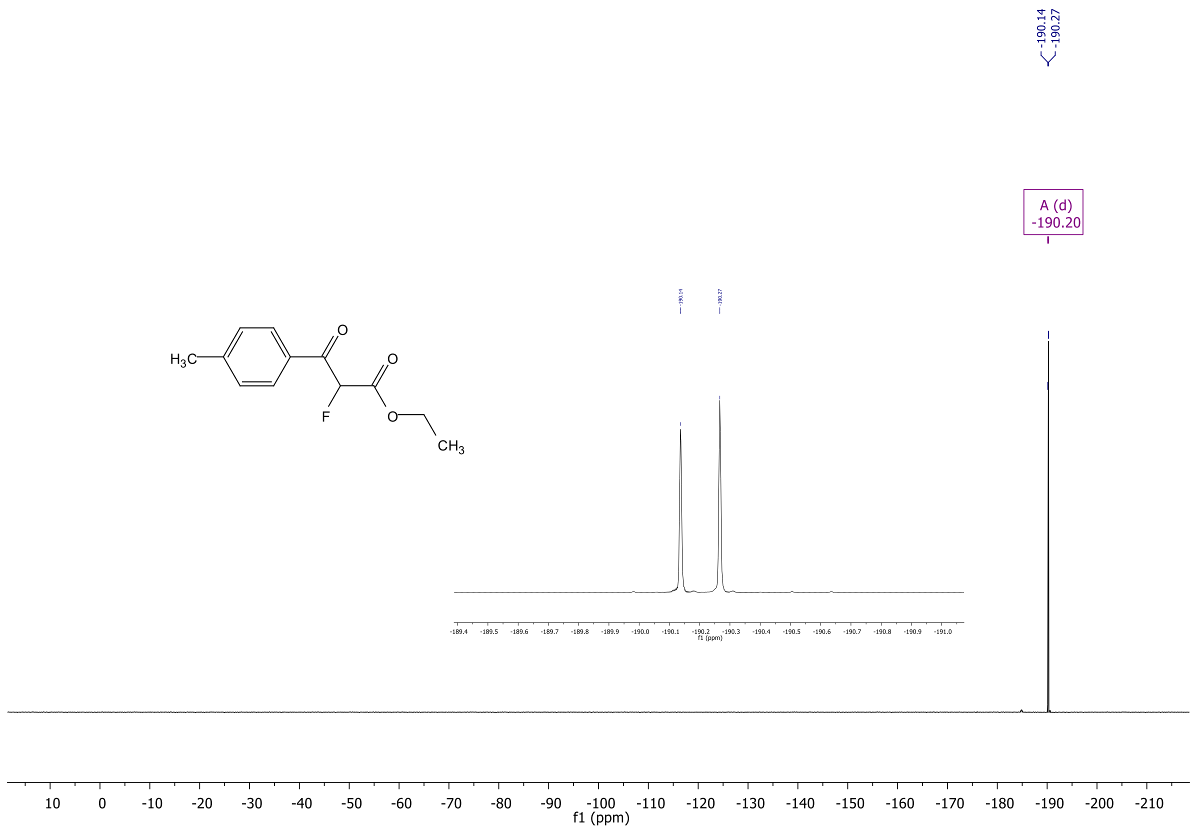

^{13}C NMR, $CDCl_3$, 101 MHz

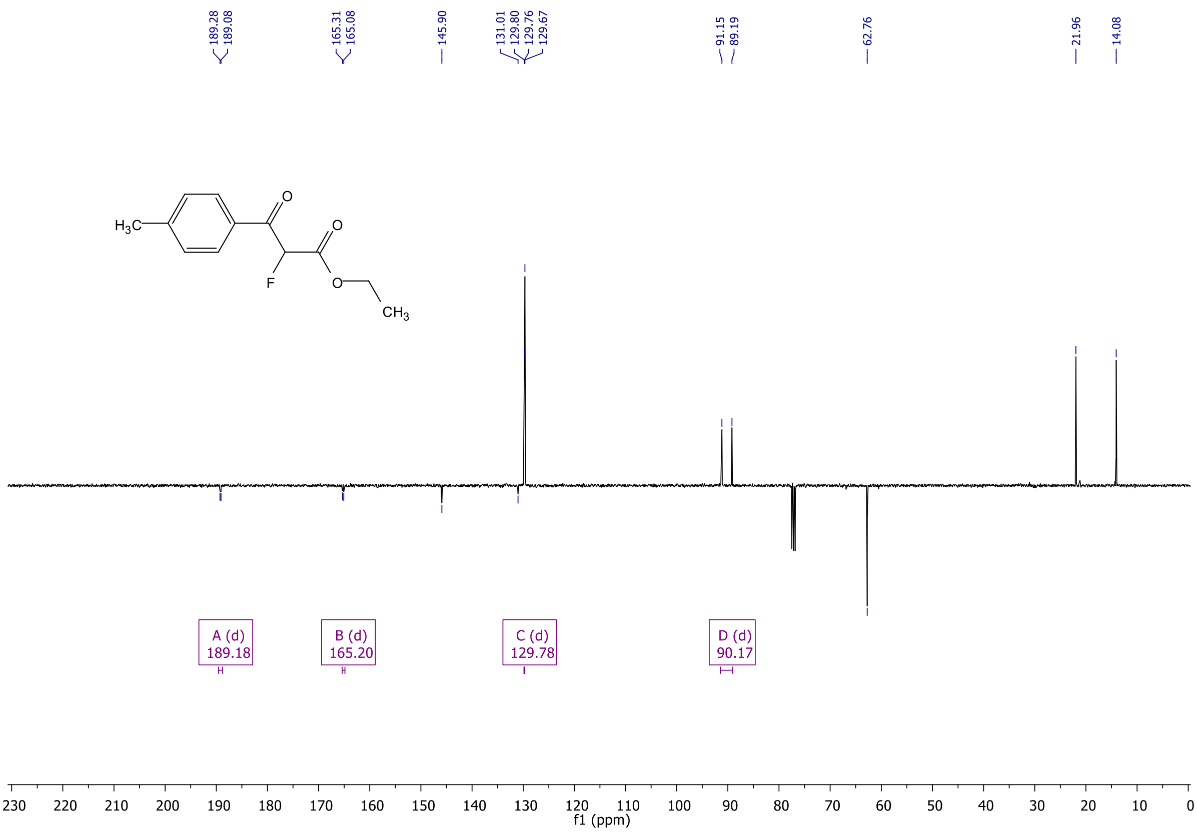

Ethyl 2-fluoro-3-(4-nitrophenyl)-3-oxopropanoate (1f)
^{1}H NMR, $CDCl_3$, 400 MHz

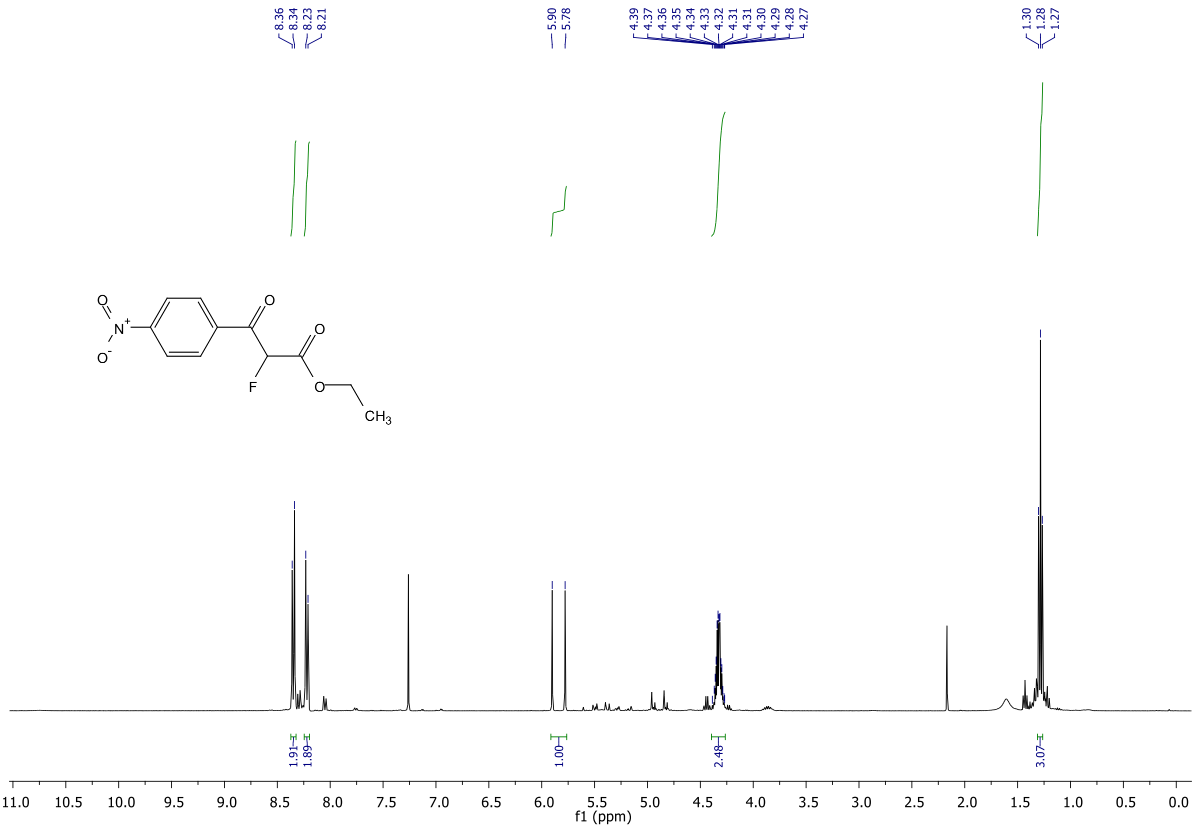

^{19}F NMR, $CDCl_3$, 377 MHz

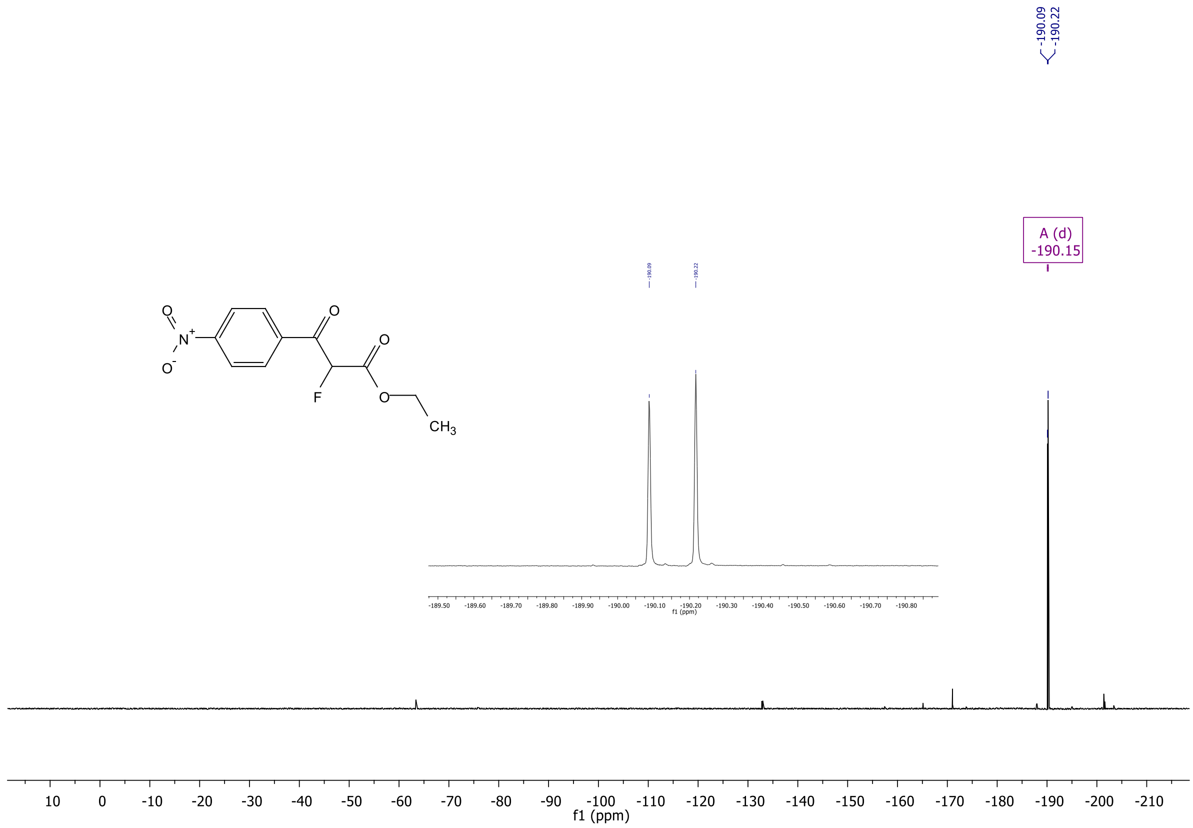

^{13}C NMR, $CDCl_3$, 101 MHz

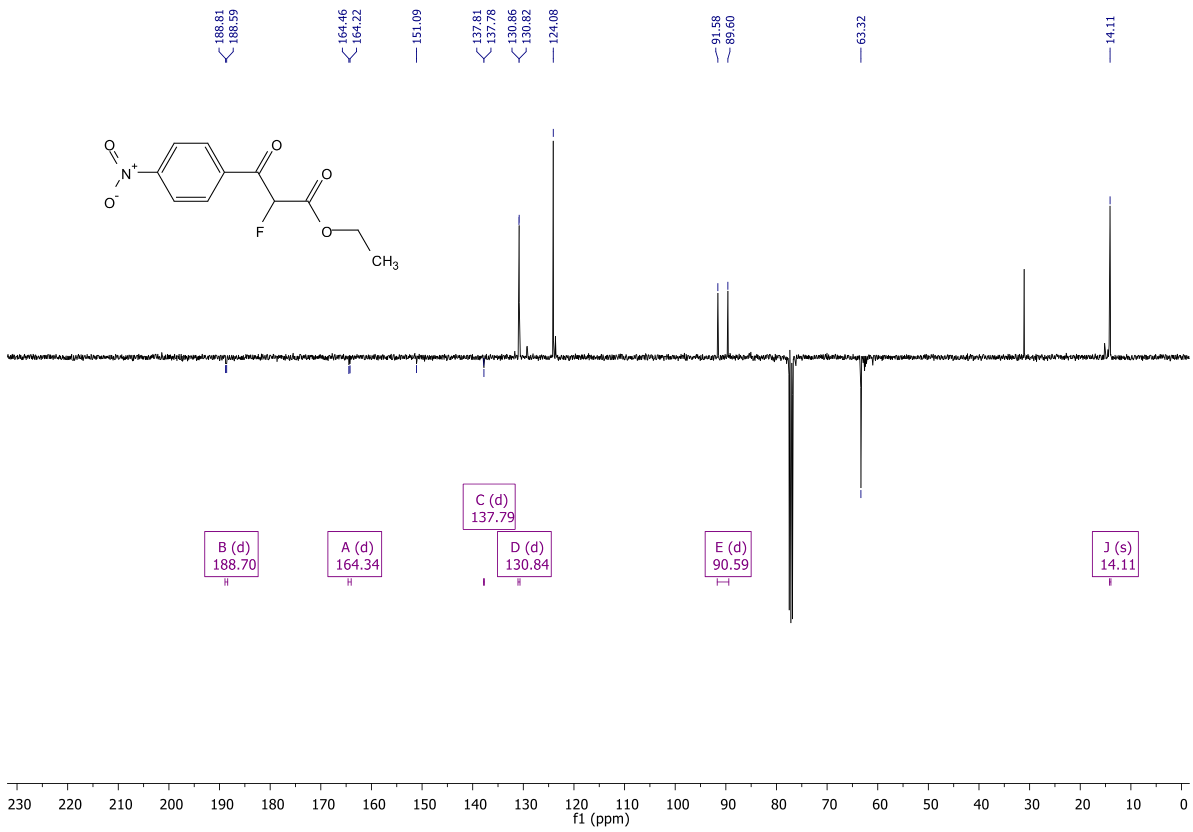

Ethyl 2-fluoro-3-(2-methylphenyl)-3-oxopropanoate (1g)

^{1}H NMR, $CDCl_3$, 400 MHz

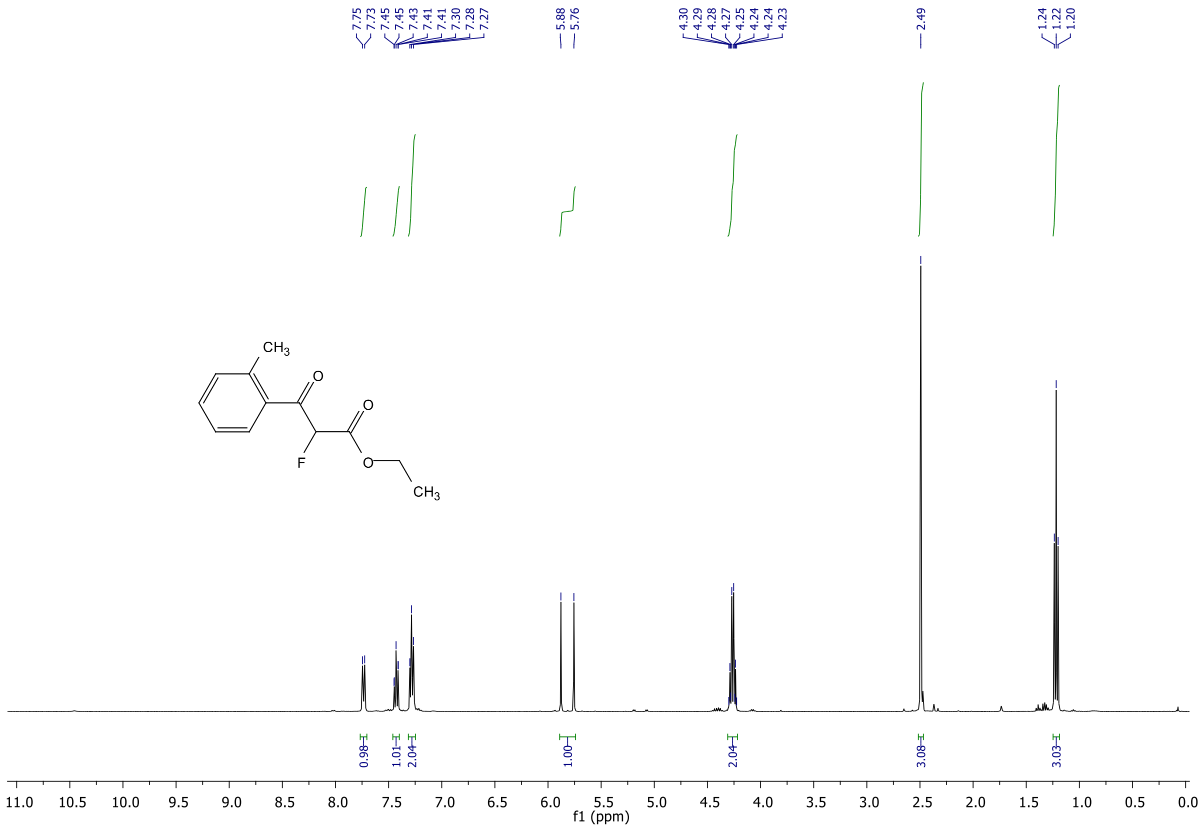

^{19}F NMR, $CDCl_3$, 377 MHz

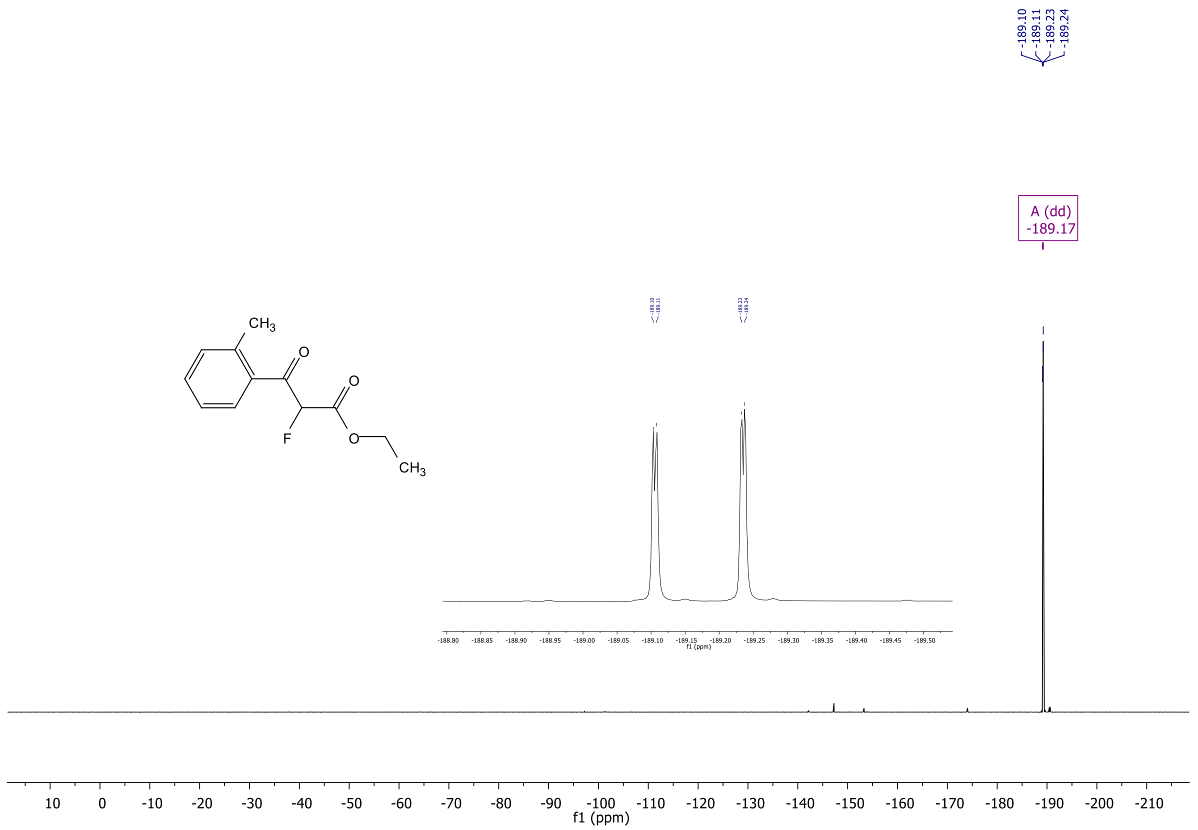

^{13}C NMR, $CDCl_3$, 101 MHz

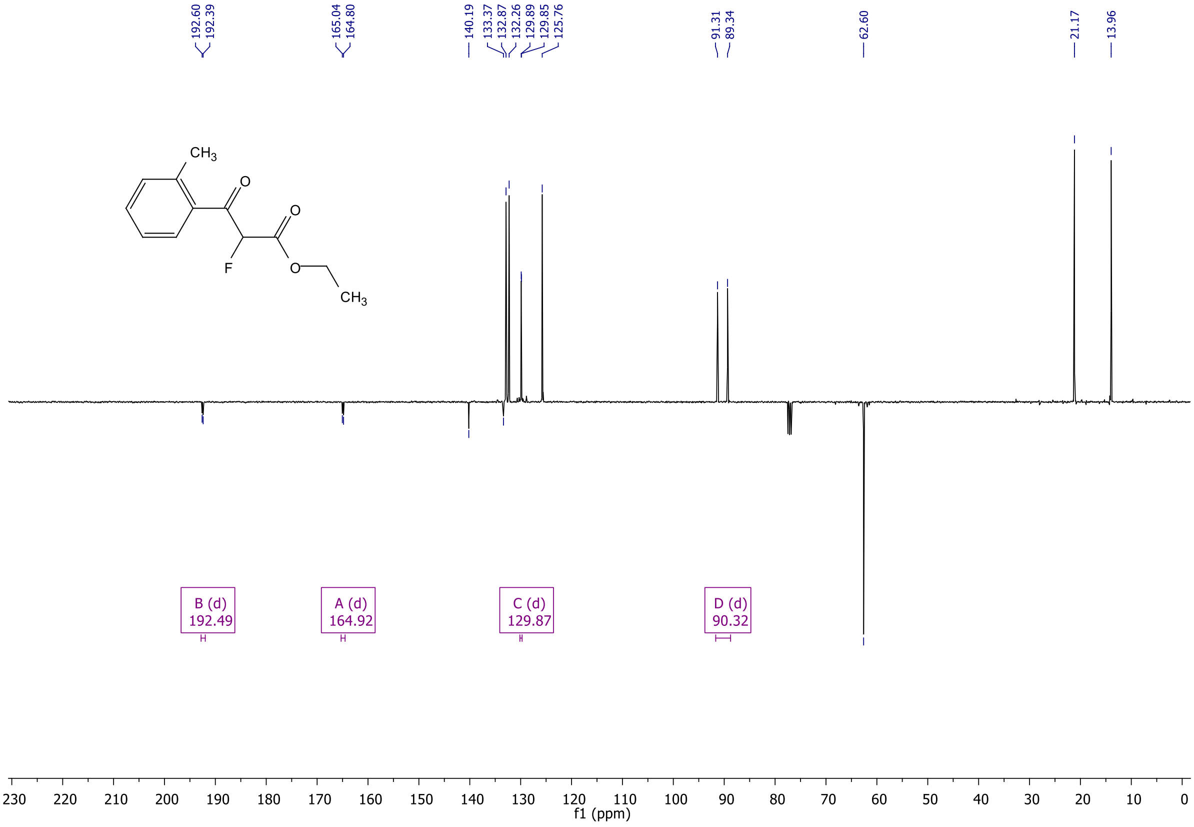

Ethyl 2-fluoro-3-(naphthalen-2-yl)-3-oxopropanoate (1h)

^{1}H NMR, $CDCl_3$, 400 MHz

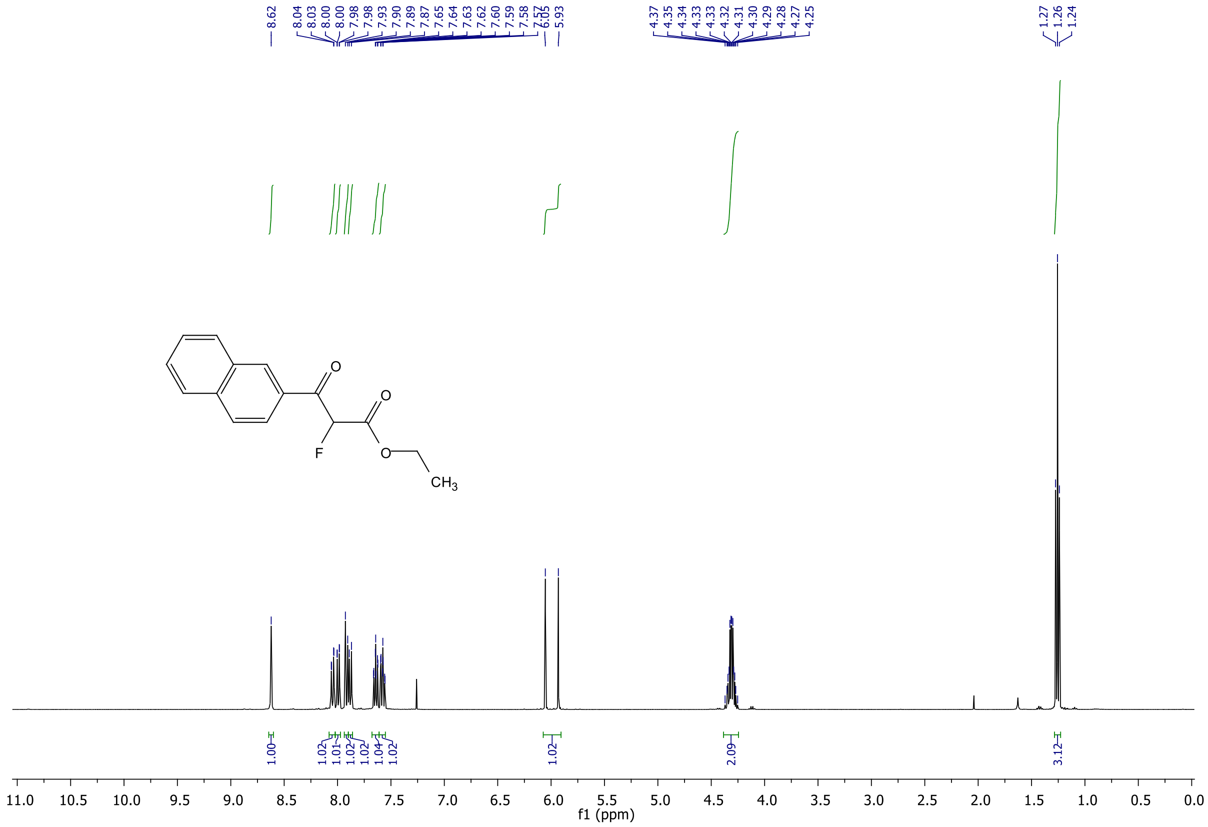

^{19}F NMR, $CDCl_3$, 377 MHz

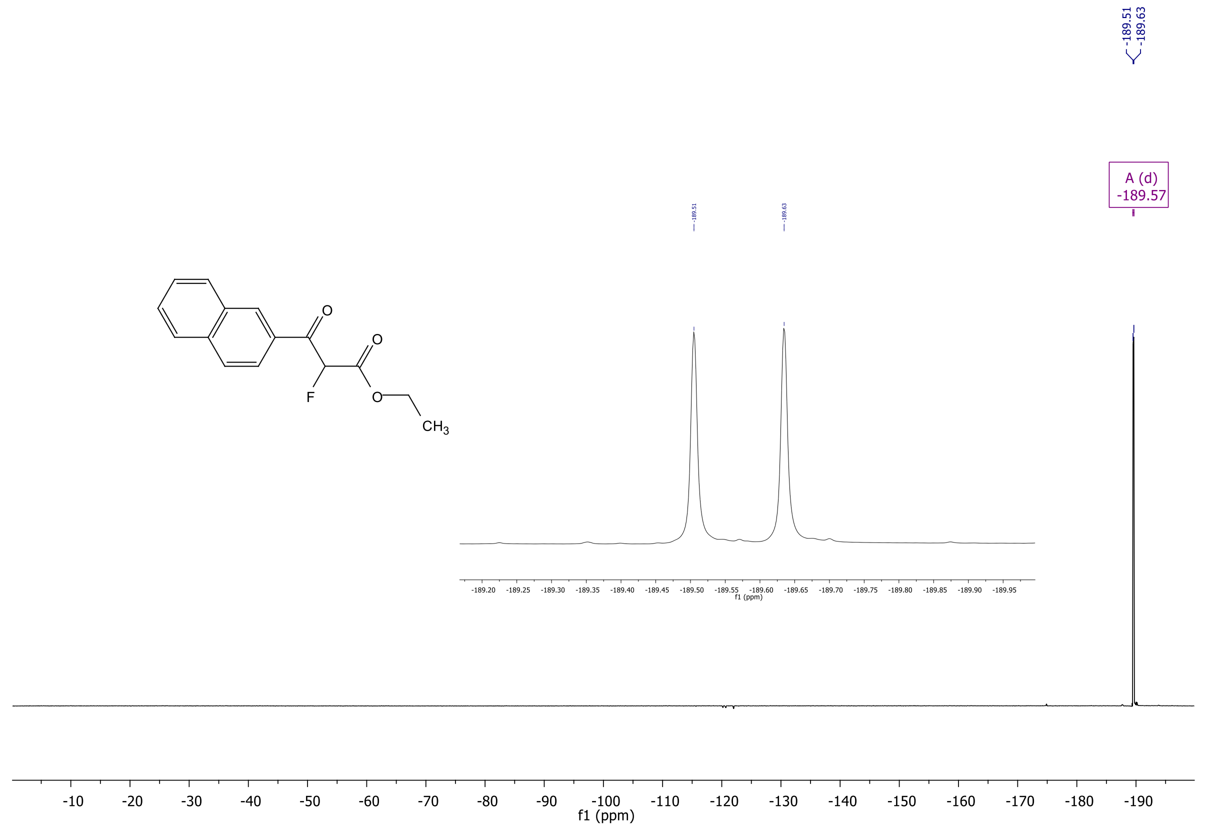

^{13}C NMR, CDCl$_3$, 101 MHz

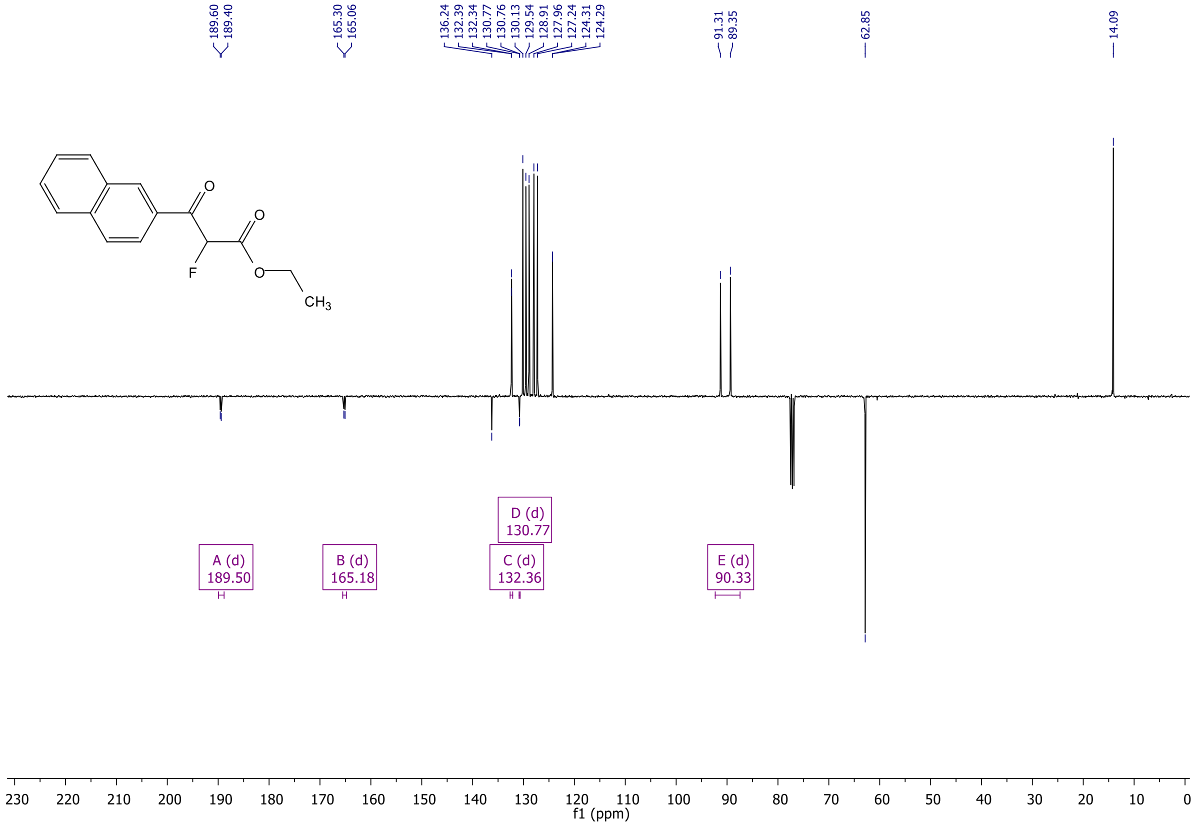

Ethyl 2-fluoro-3-oxo-3-(thiophen-2-yl)propanoate (1i)

^1H NMR, $CDCl_3$, 400 MHz

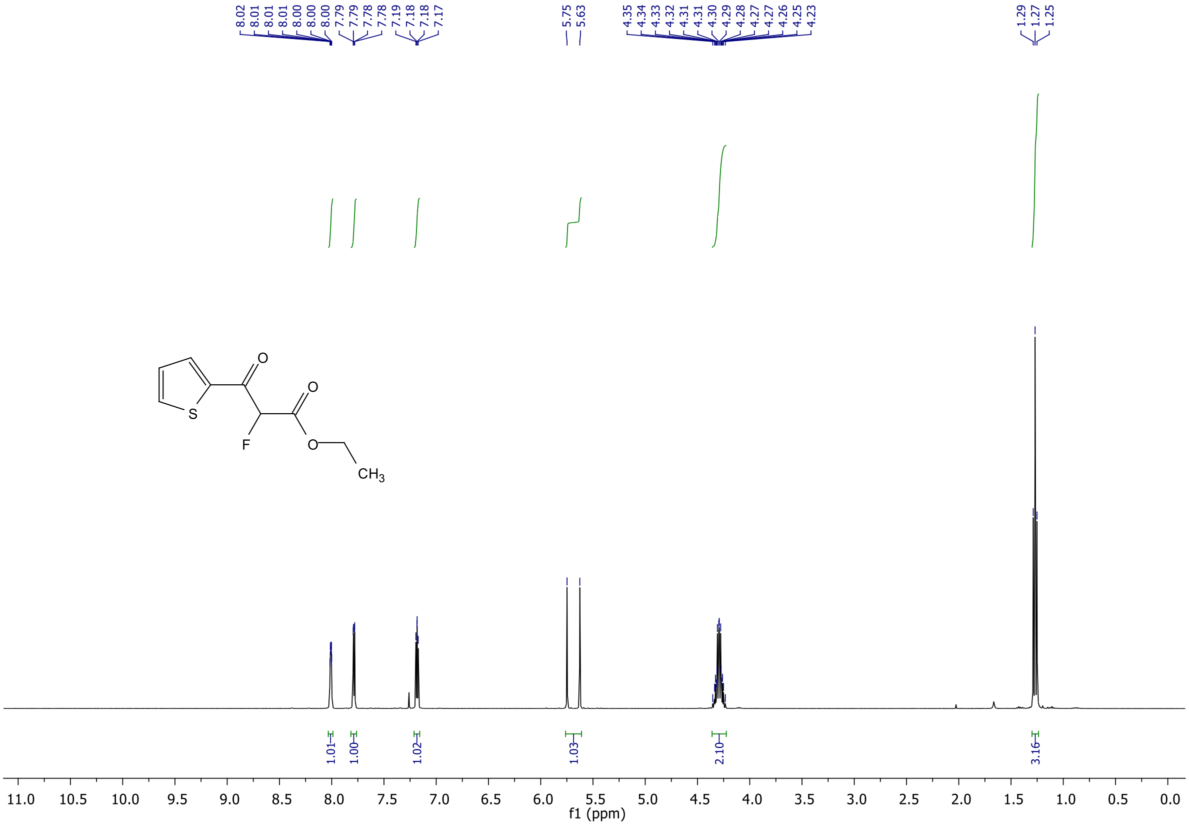

^{19}F NMR, $CDCl_3$, 377 MHz

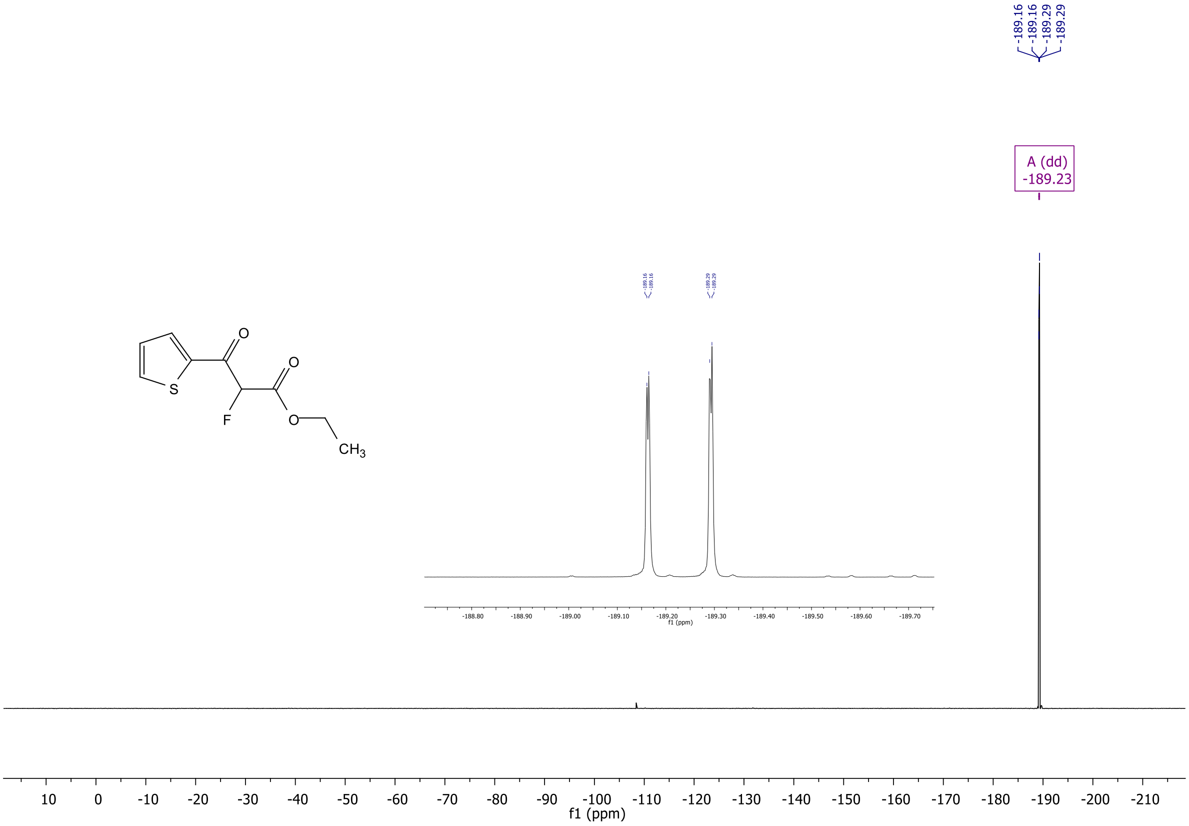

^{13}C NMR, $CDCl_3$, 101 MHz

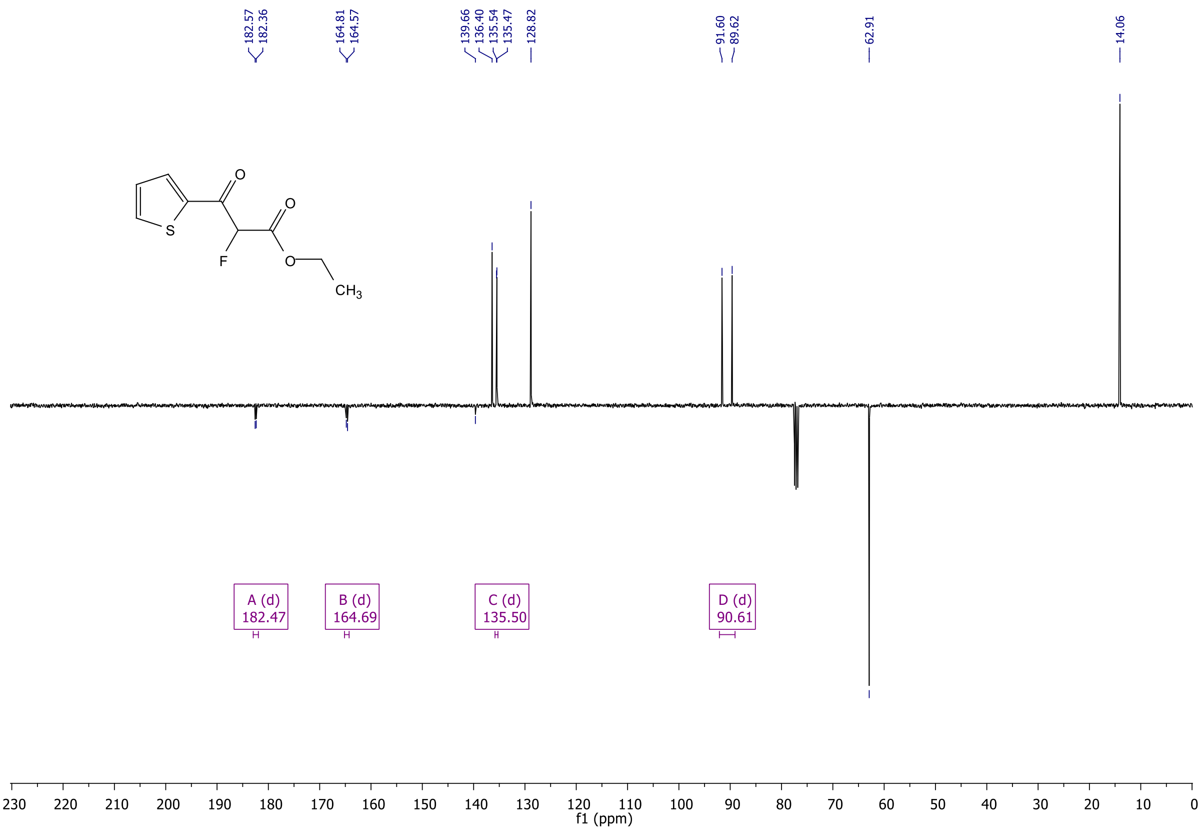

2-Fluoro-3-oxo-3-phenylpropanenitrile (1j)

^{1}H NMR, $CDCl_3$, 400 MHz

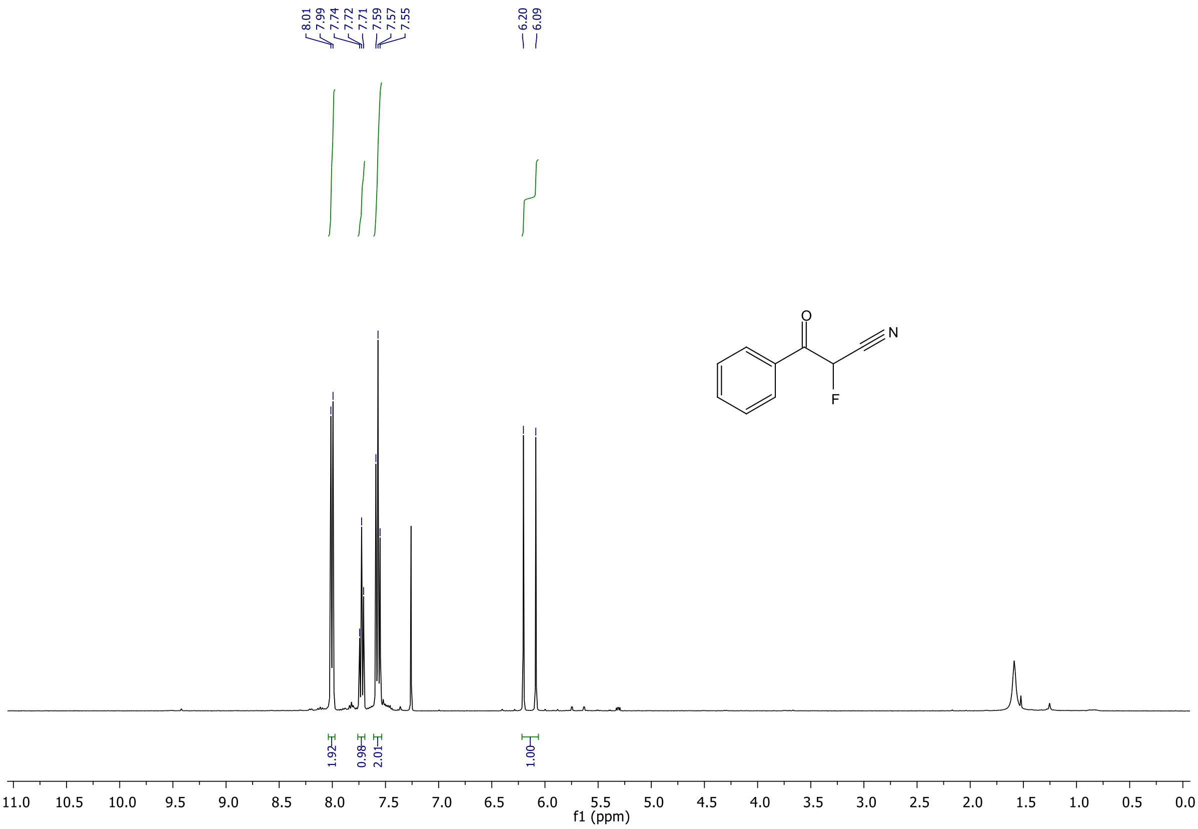

^{19}F NMR, $CDCl_3$, 377 MHz

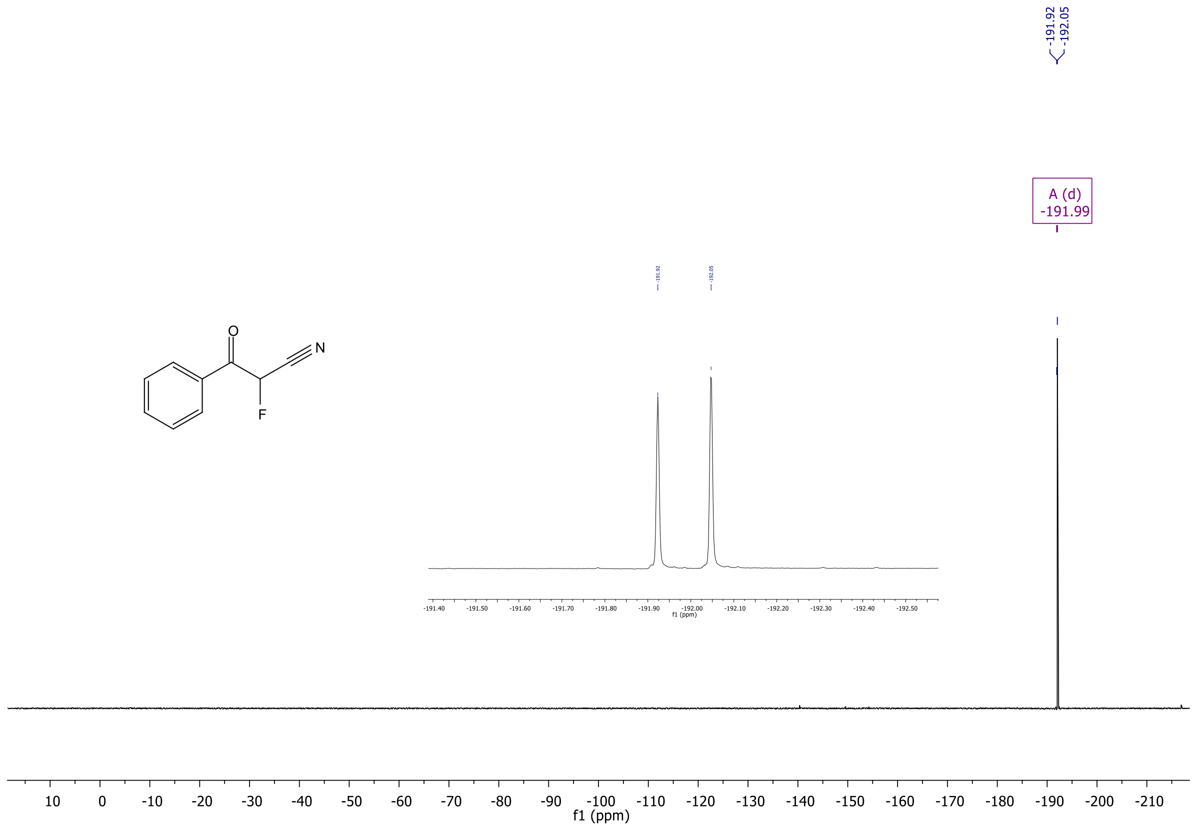

^{13}C NMR, $CDCl_3$, 101 MHz

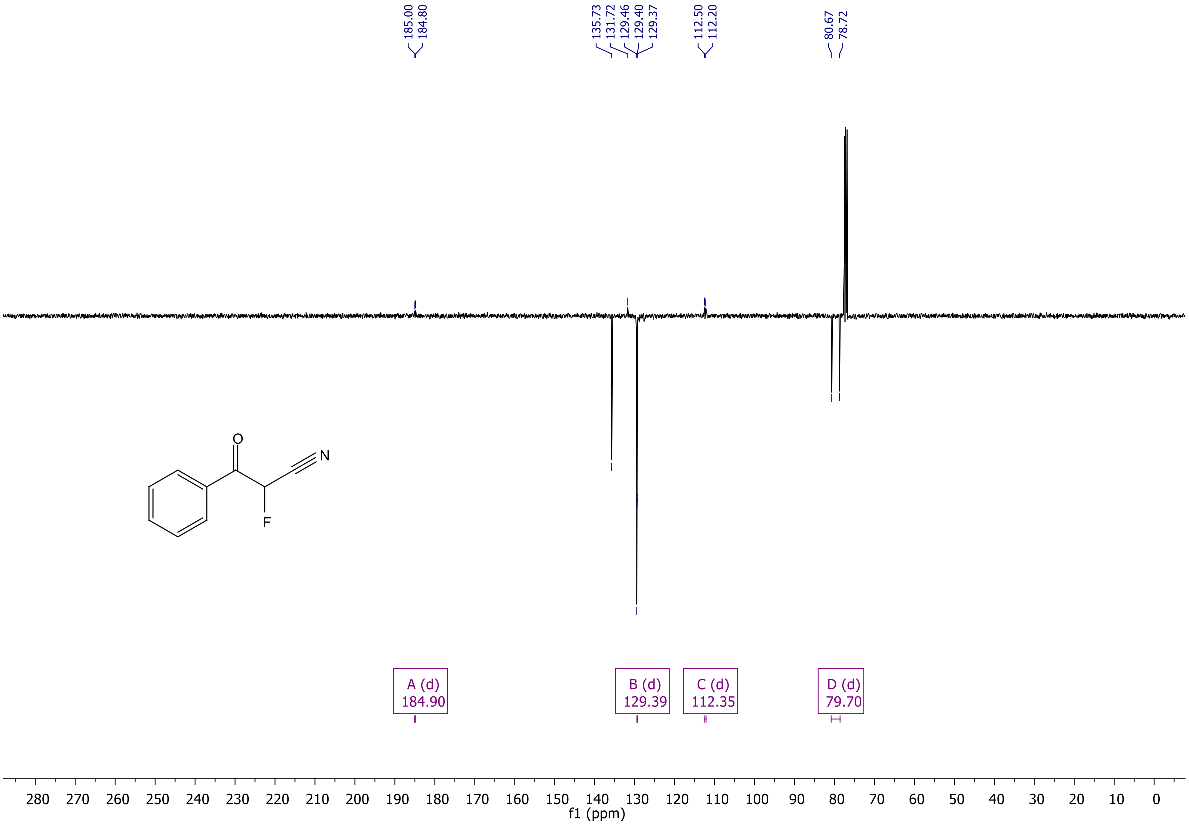

2-Fluoro-1-phenyl-2-(phenylsulfonyl)ethanone (1k)

^{1}H NMR, $CDCl_3$, 400 MHz

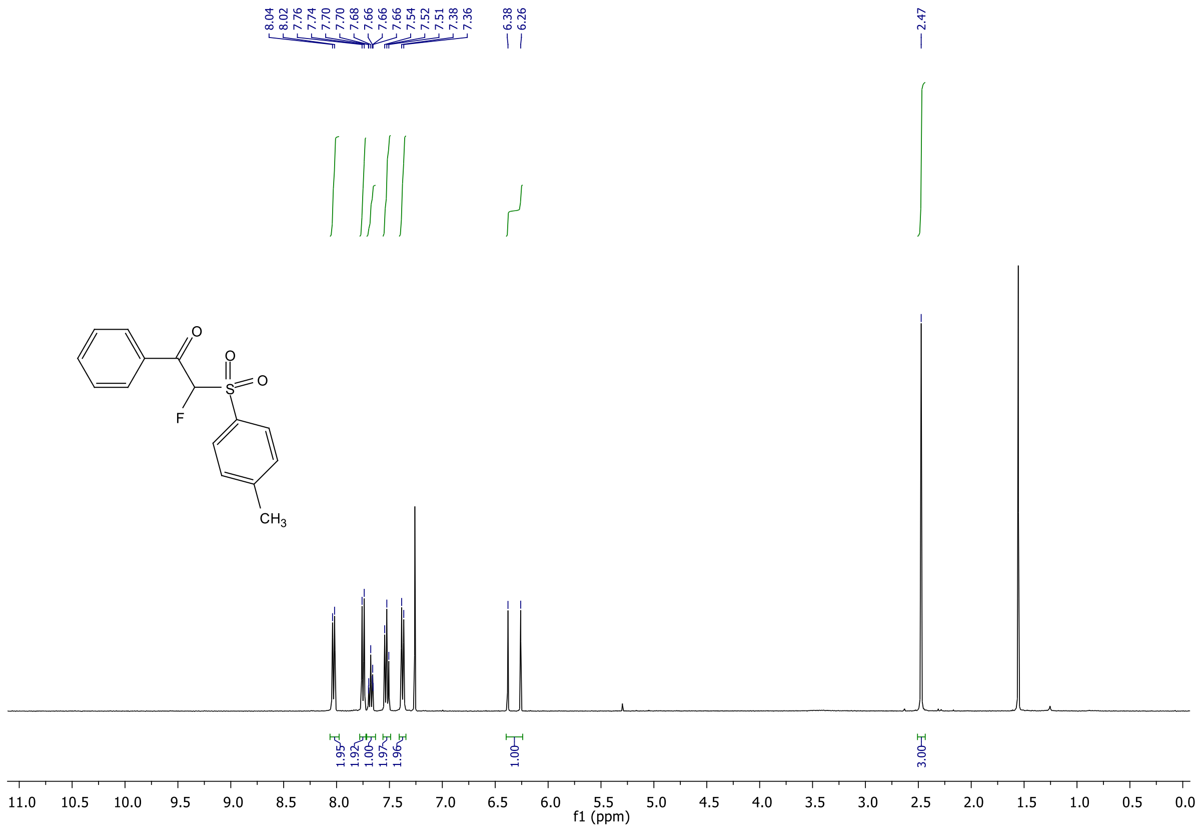

^{19}F NMR, $CDCl_3$, 377 MHz

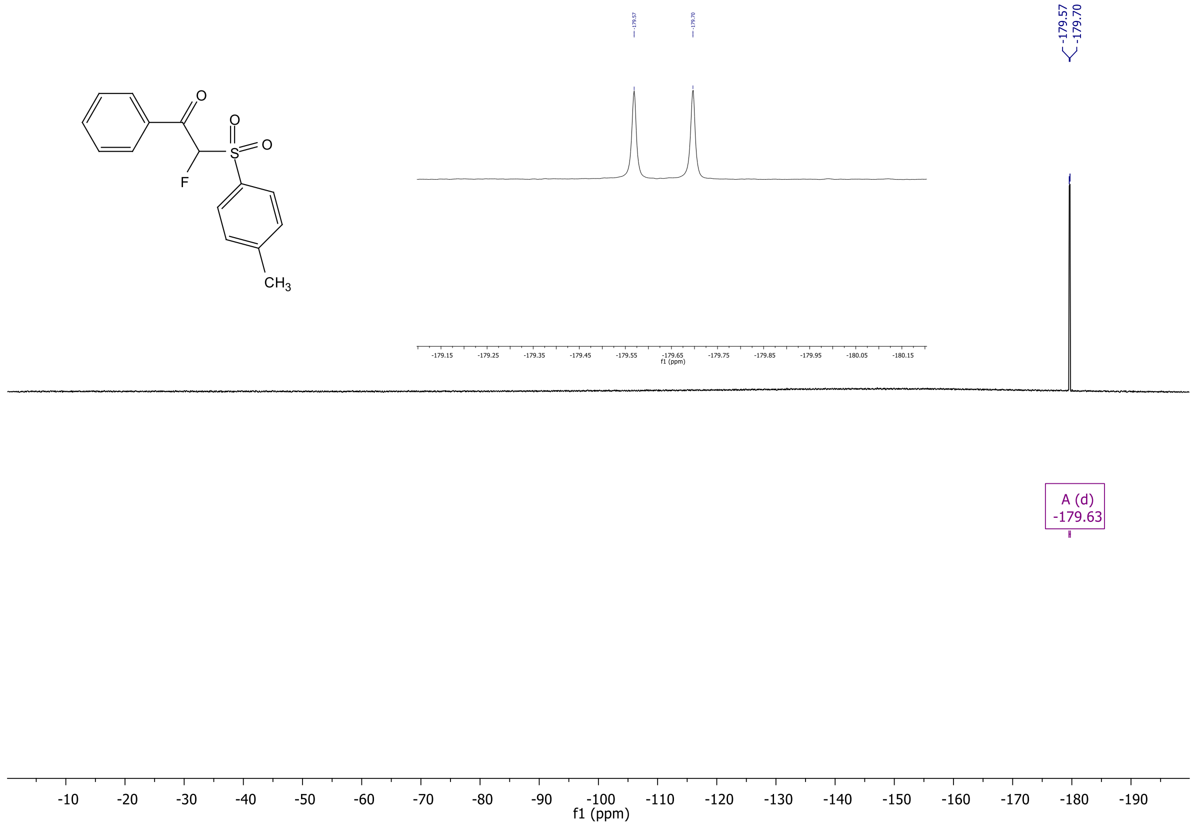

^{13}C NMR, $CDCl_3$, 101 MHz

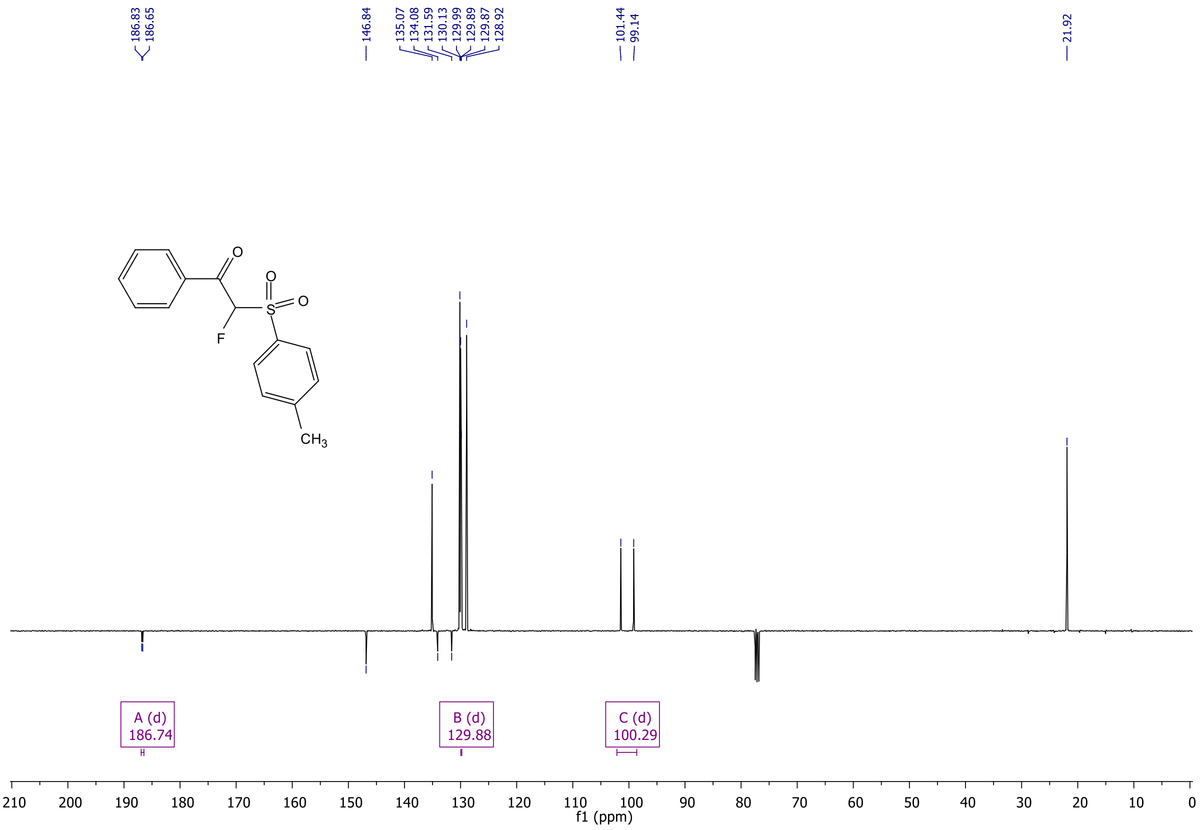

2-Fluoro-*N*-methoxy-*N*-methyl-3-oxo-3-phenylpropanamide (1l)

^{1}H NMR, $CDCl_3$, 400 MHz

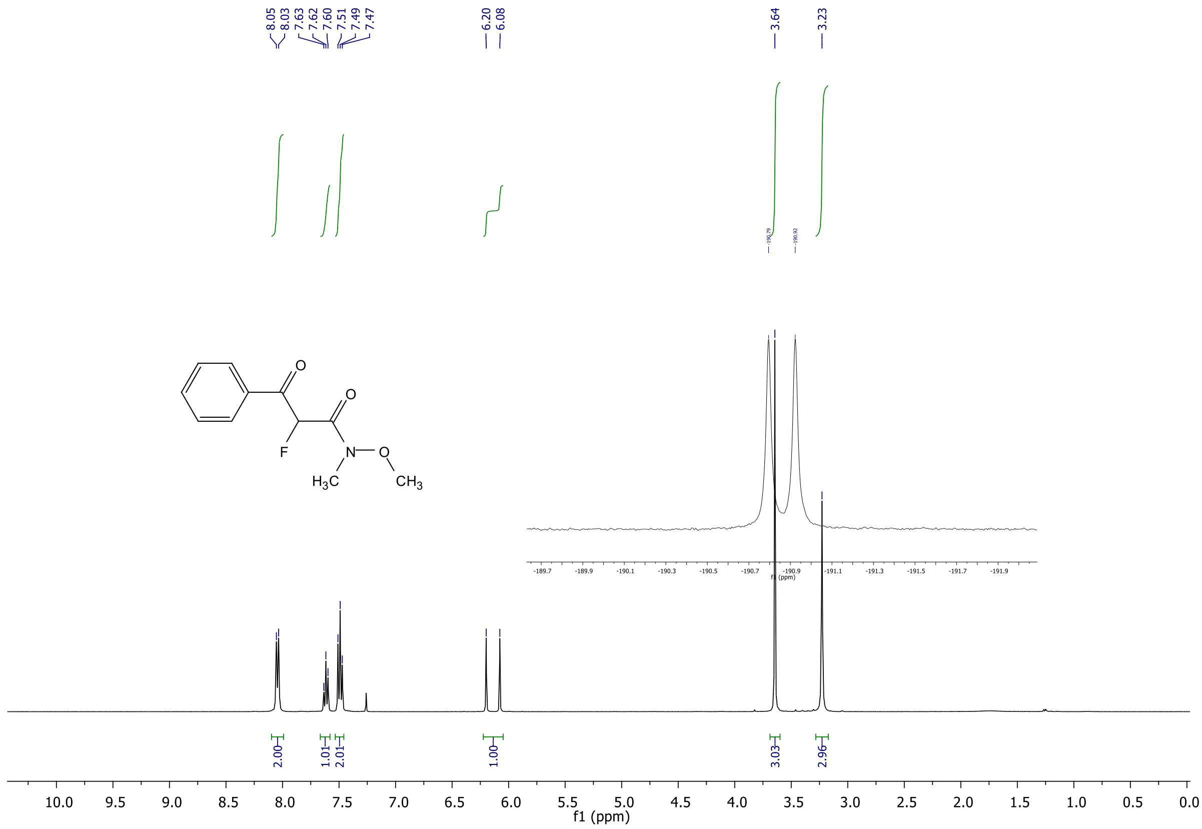

^{19}F NMR, $CDCl_3$, 377 MHz

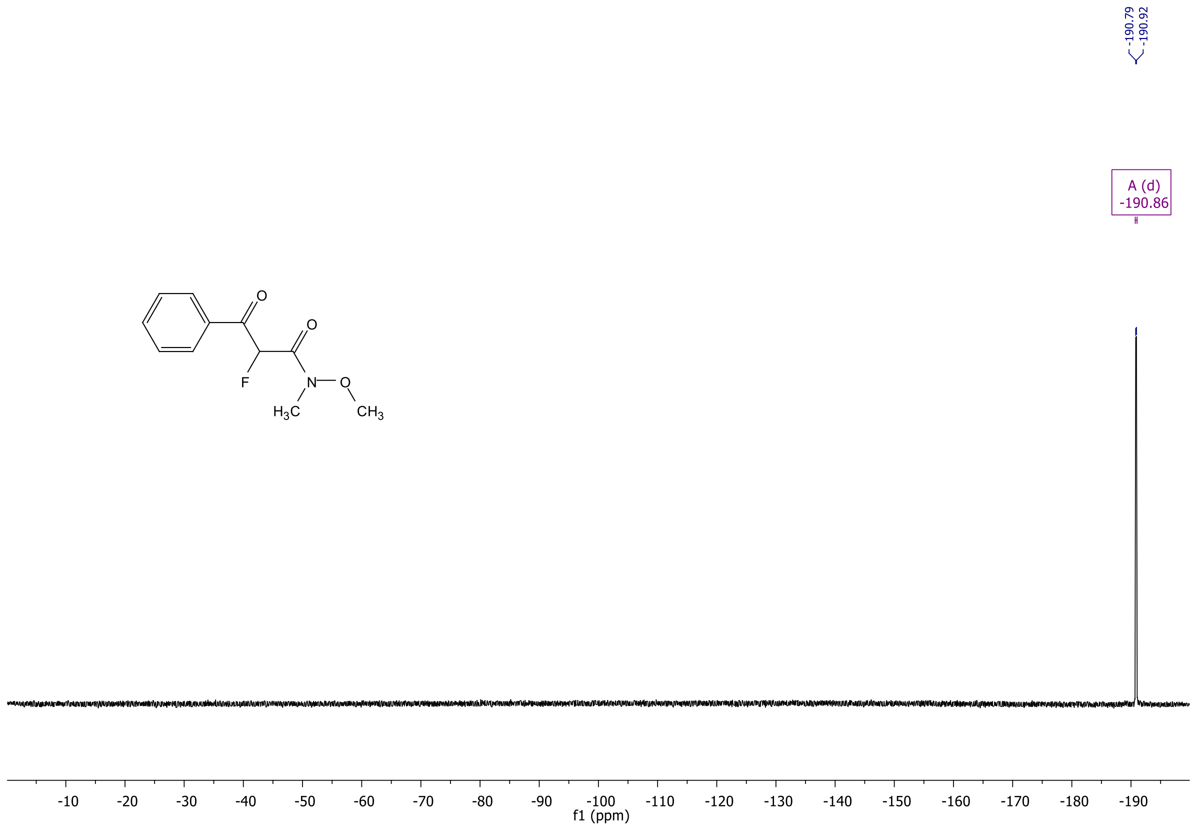

^{13}C NMR, $CDCl_3$, 101 MHz

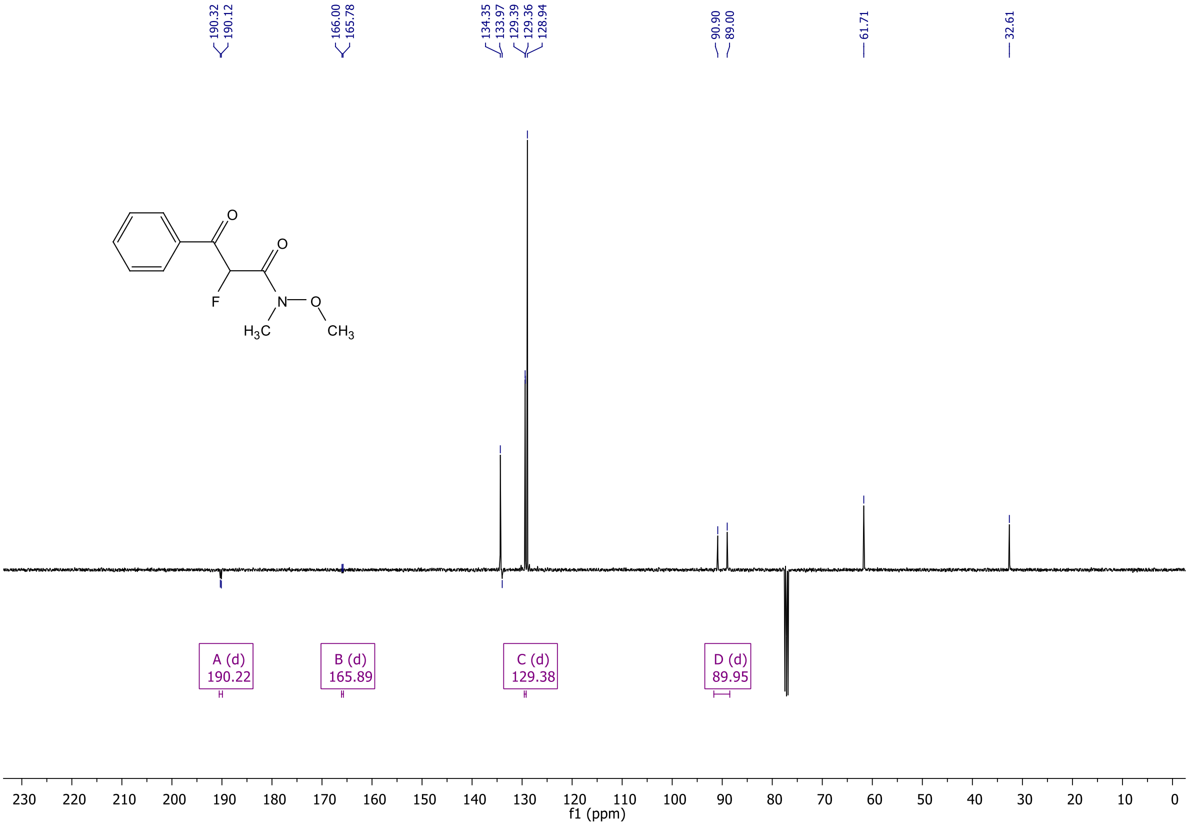

Ethyl 2-fluoro-3-oxobutanoate (1m)

^{1}H NMR, $CDCl_3$, 400 MHz

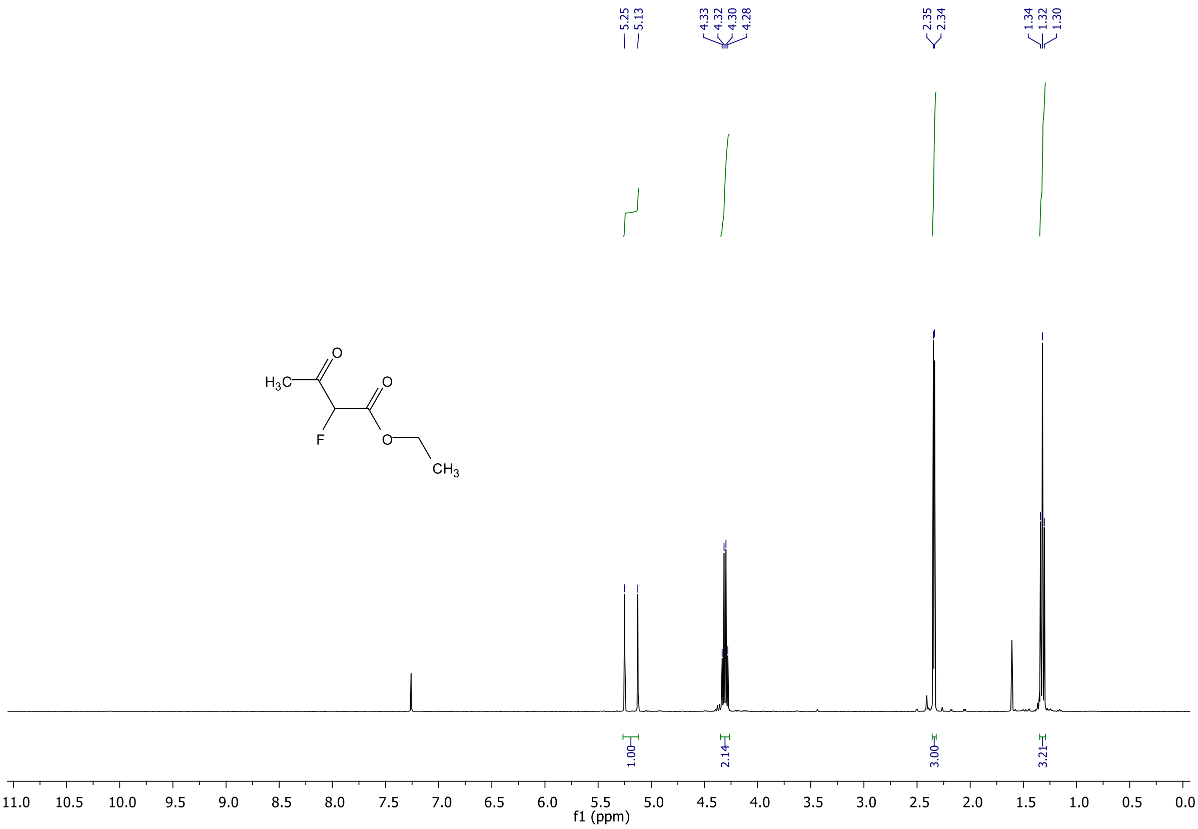

^{19}F NMR, $CDCl_3$, 377 MHz

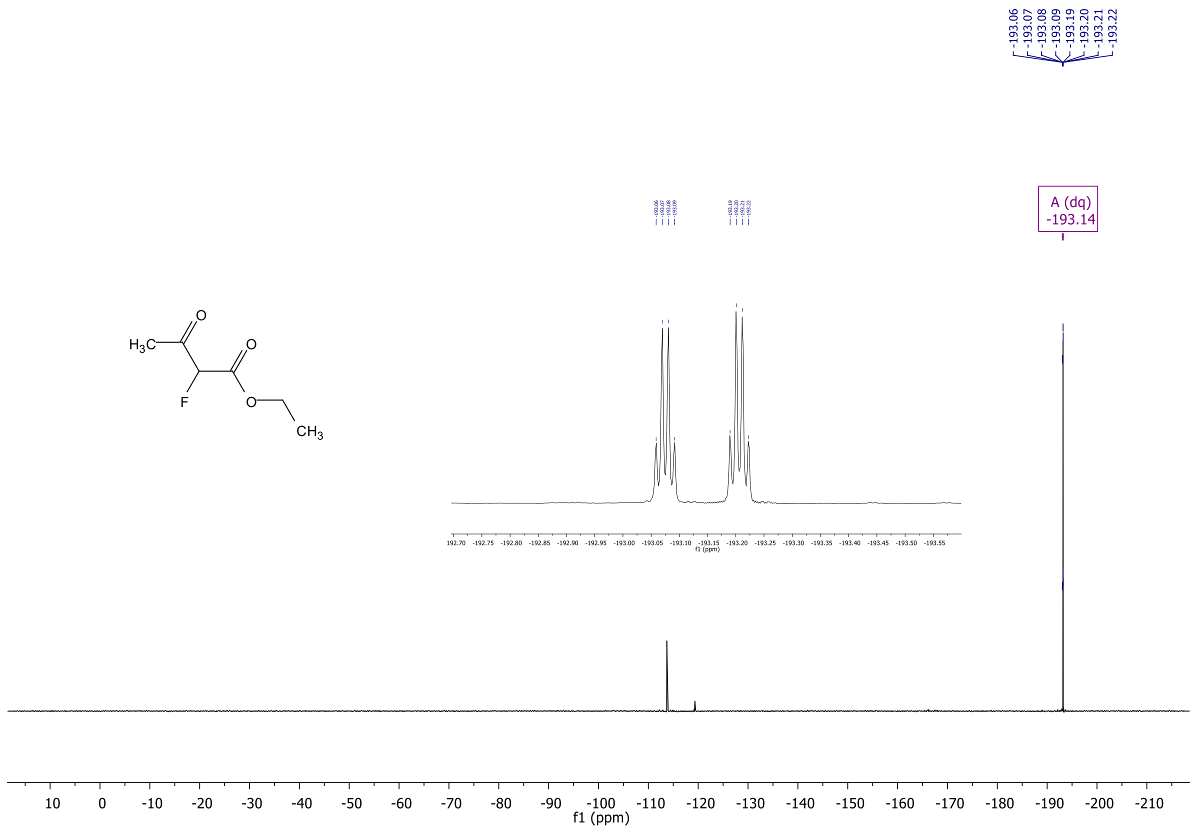

^{13}C NMR, $CDCl_3$, 101 MHz

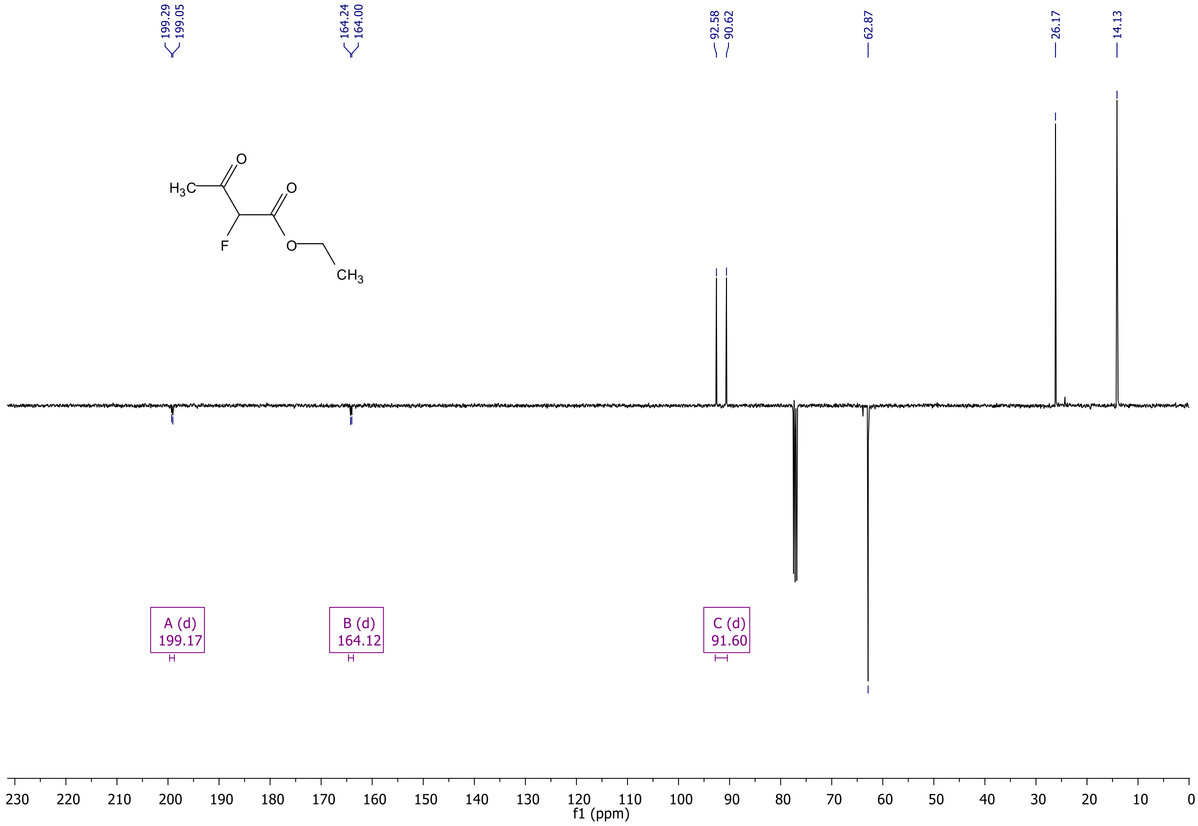

Ethyl 2-fluoro-3-oxopentanoate (1n)

^{1}H NMR, $CDCl_3$, 400 MHz

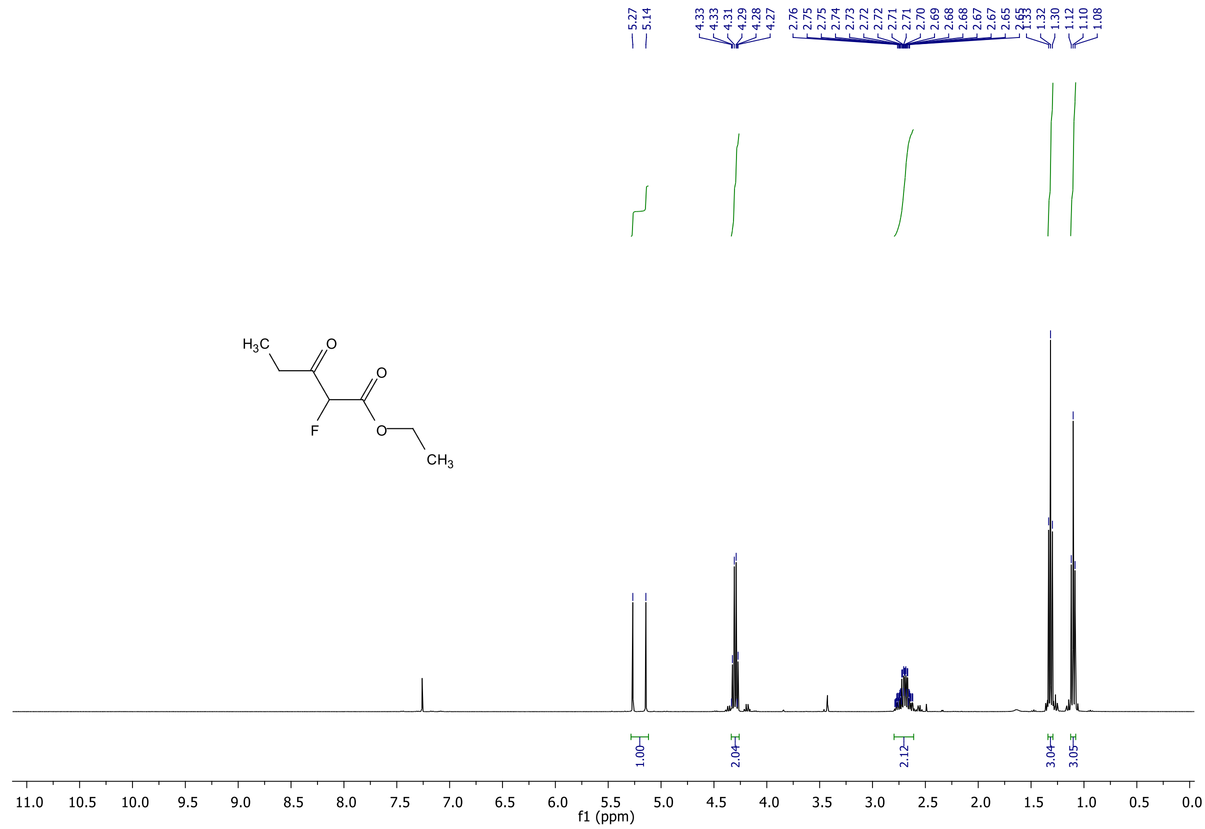

^{19}F NMR, $CDCl_3$, 377 MHz

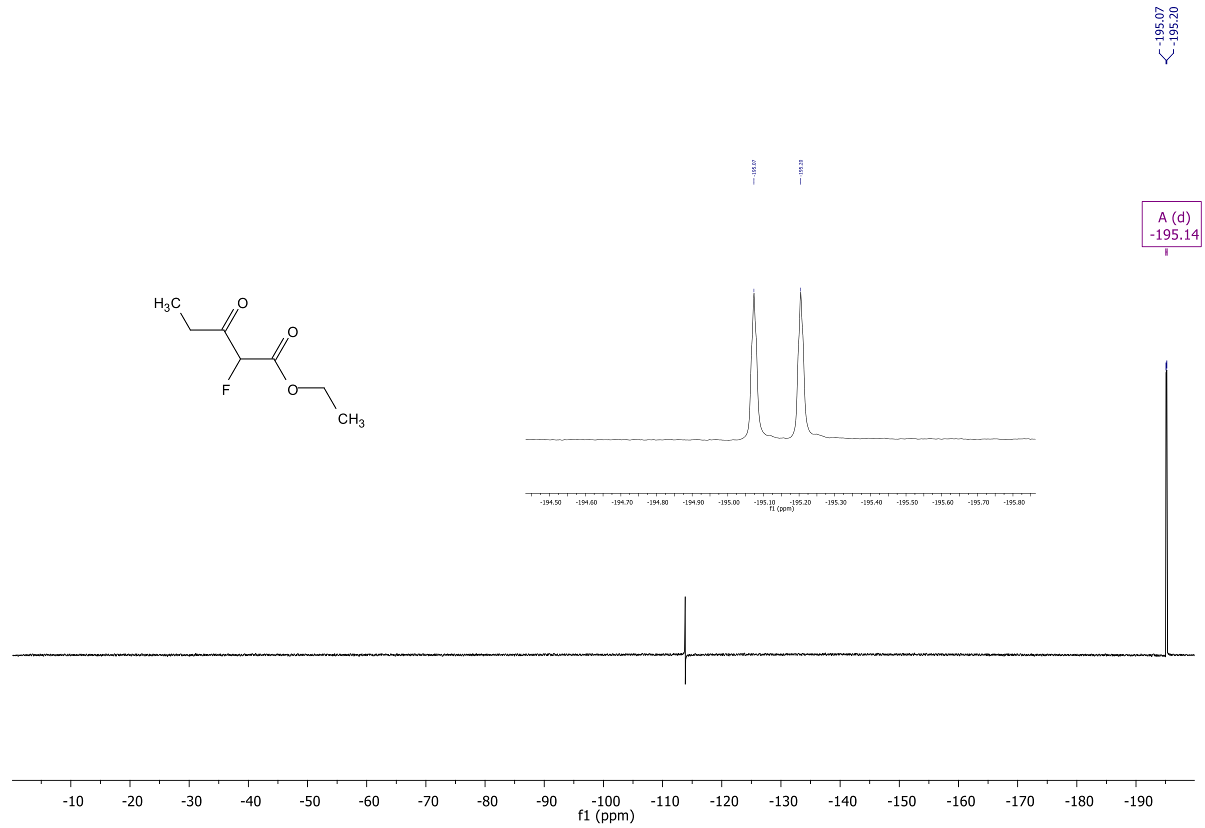

^{13}C NMR, $CDCl_3$, 101 MHz

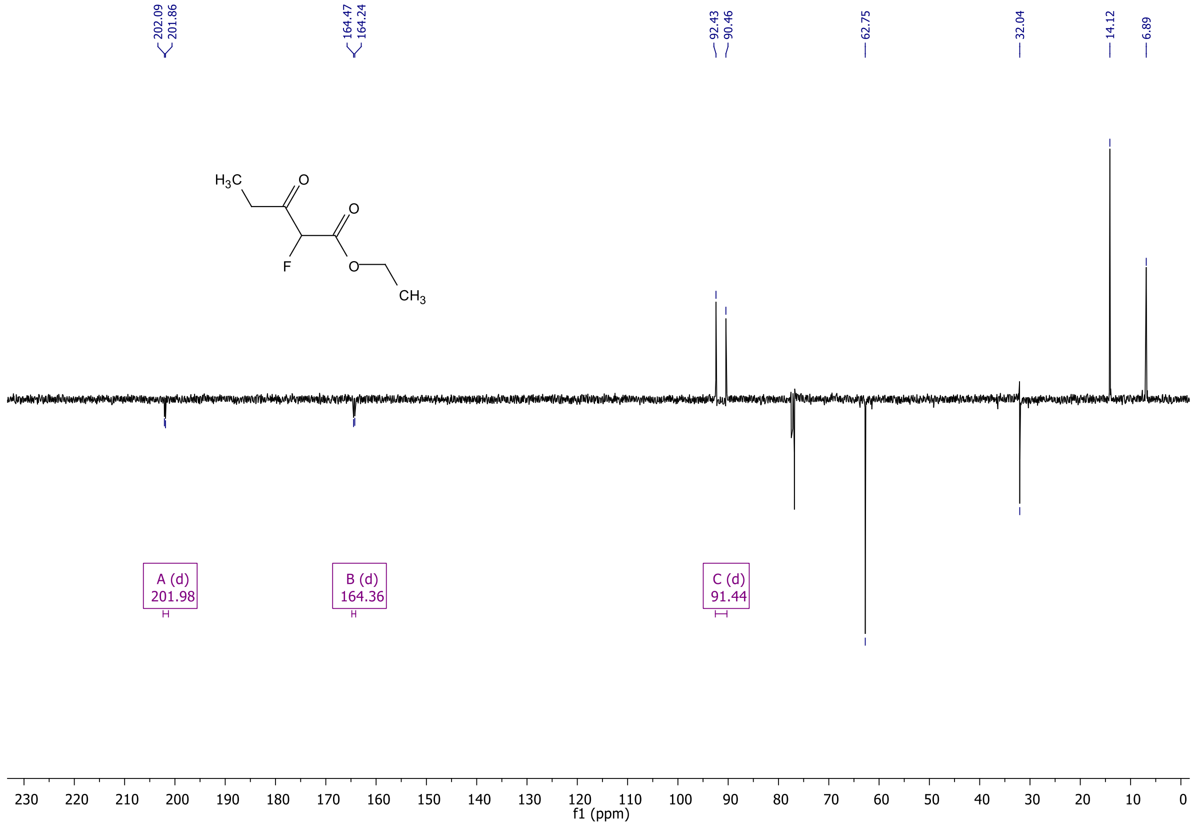

Ethyl 2-fluoro-4-methyl-3-oxopentanoate (1o)
^{1}H NMR, $CDCl_3$, 400 MHz

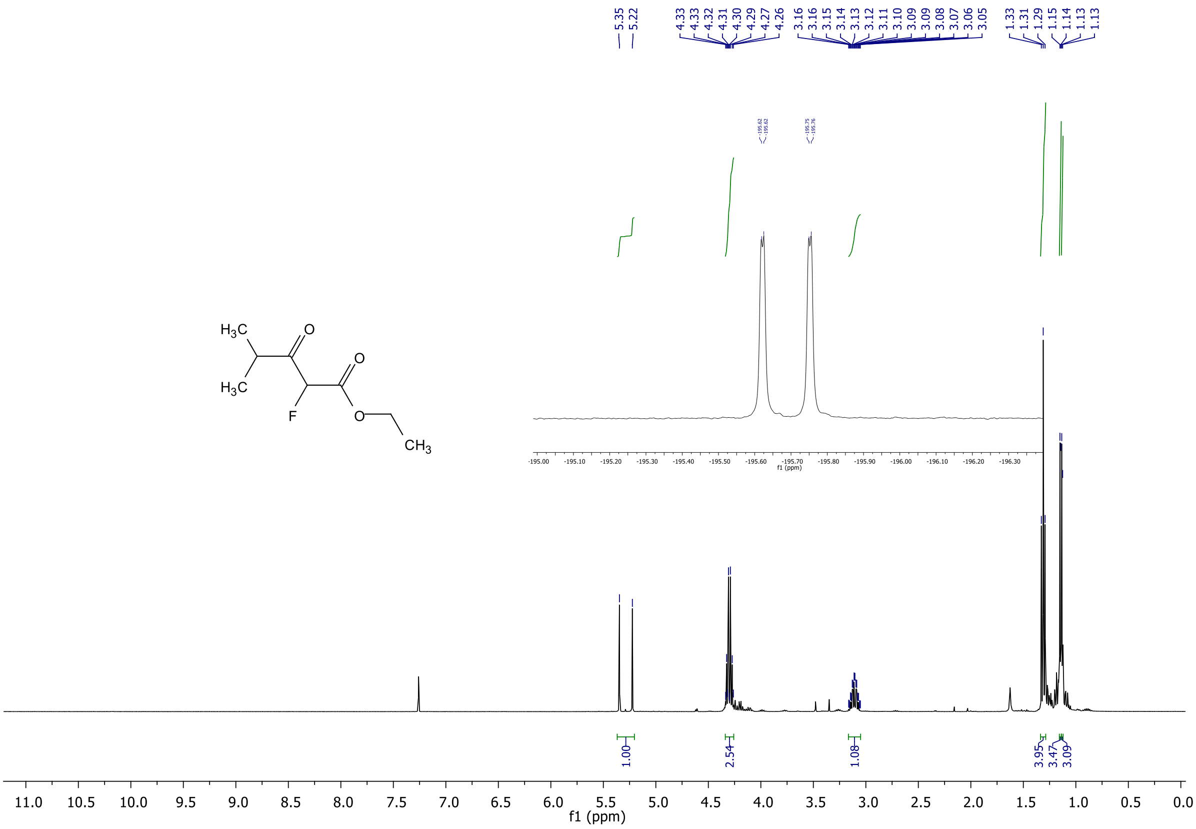

^{19}F NMR, $CDCl_3$, 377 MHz

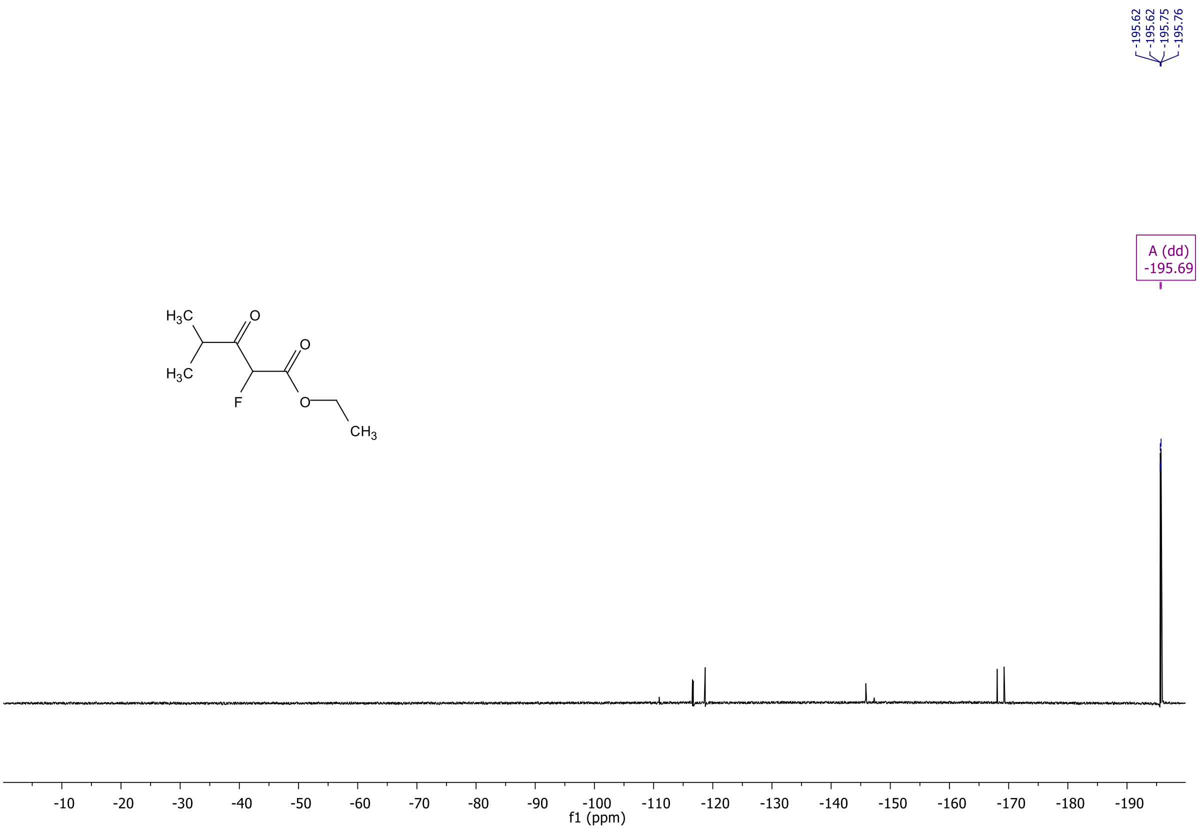

^{13}C NMR, $CDCl_3$, 101 MHz

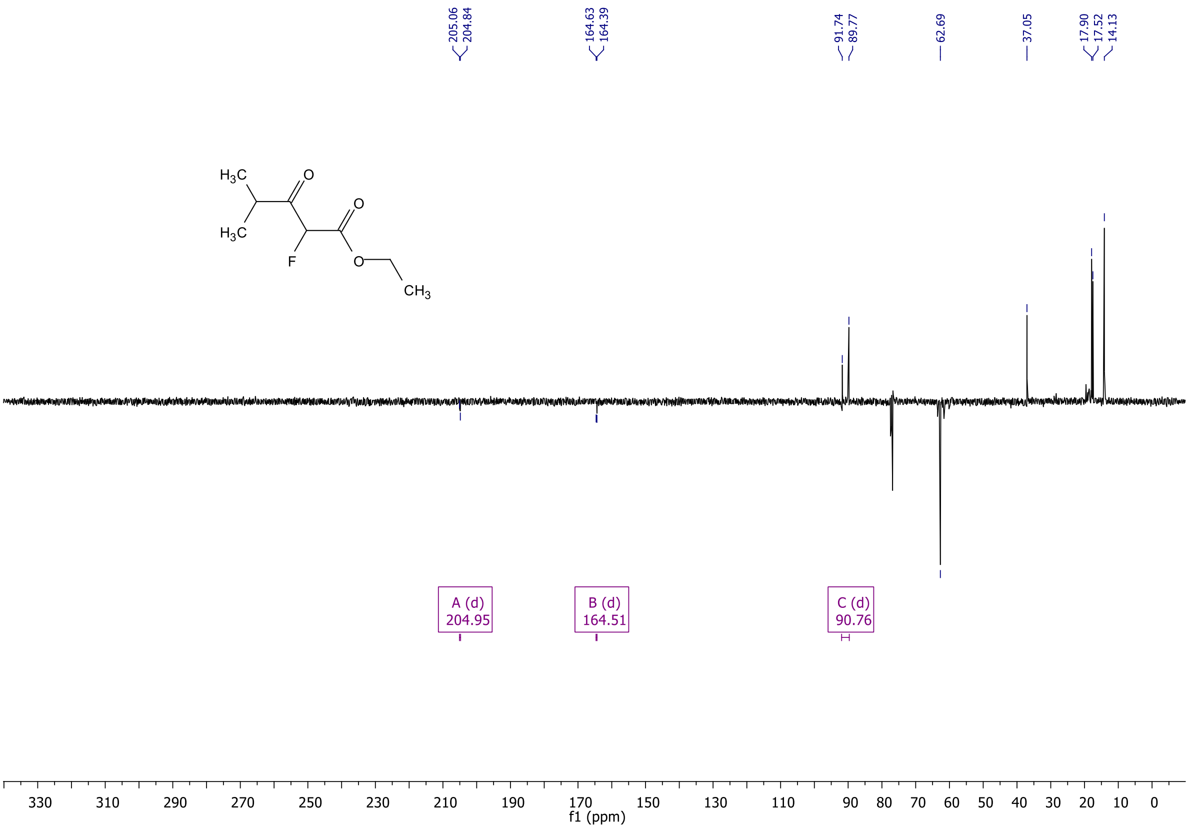

Ethyl 2-fluoro-4,4-dimethyl-3-oxopentanoate (1p)
^{1}H NMR, $CDCl_3$, 400 MHz

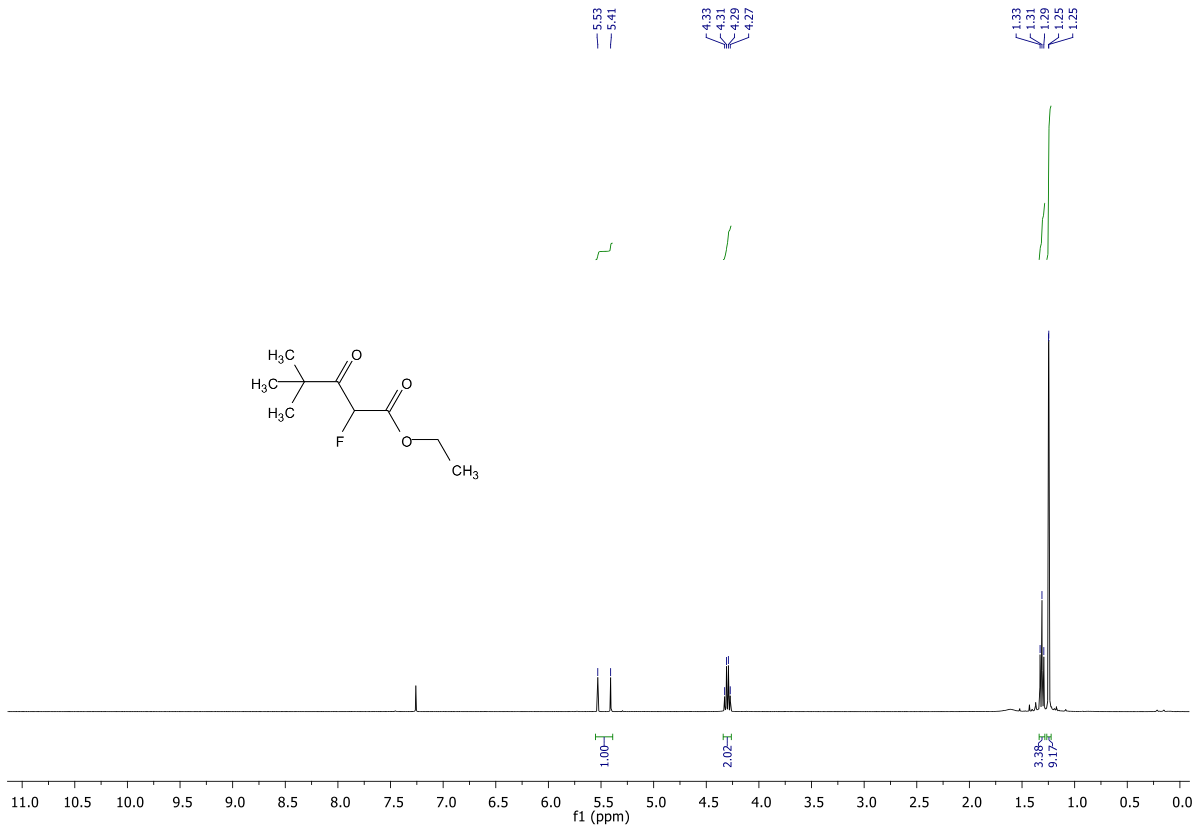

^{19}F NMR, $CDCl_3$, 377 MHz

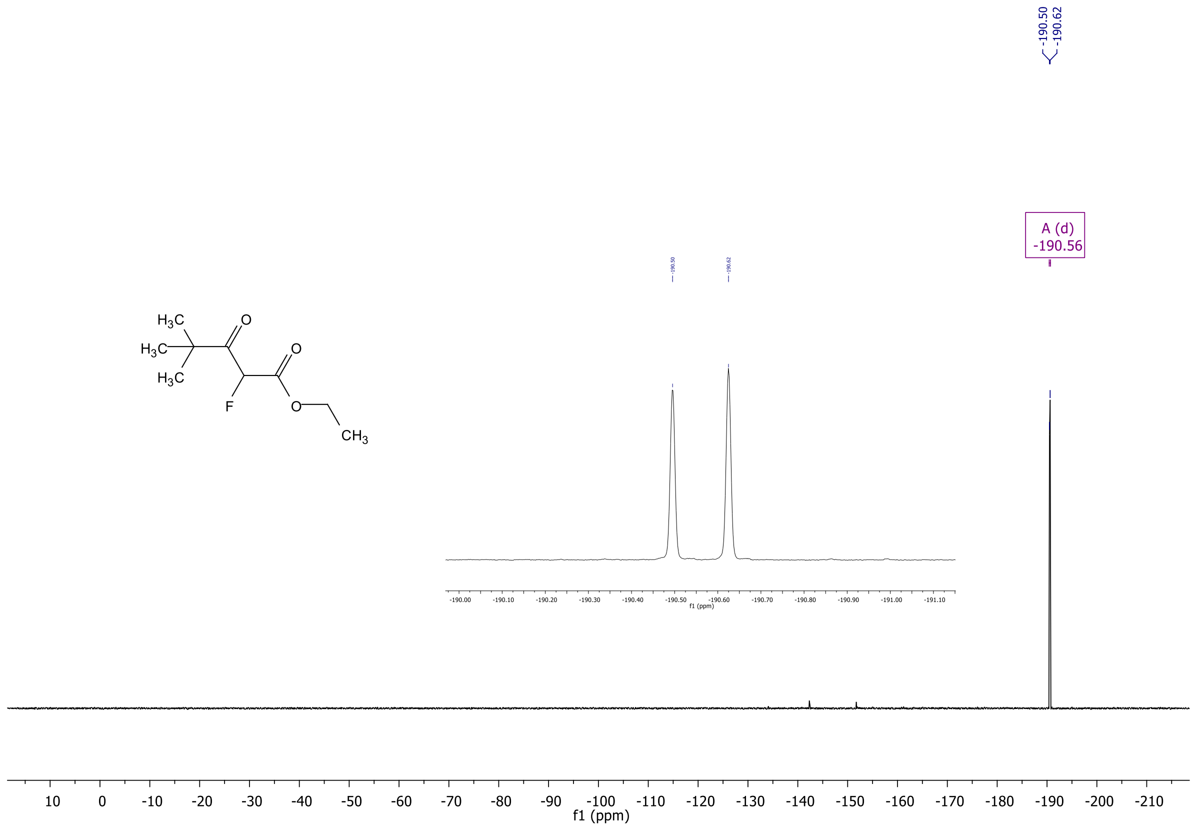

^{13}C NMR, $CDCl_3$, 101 MHz

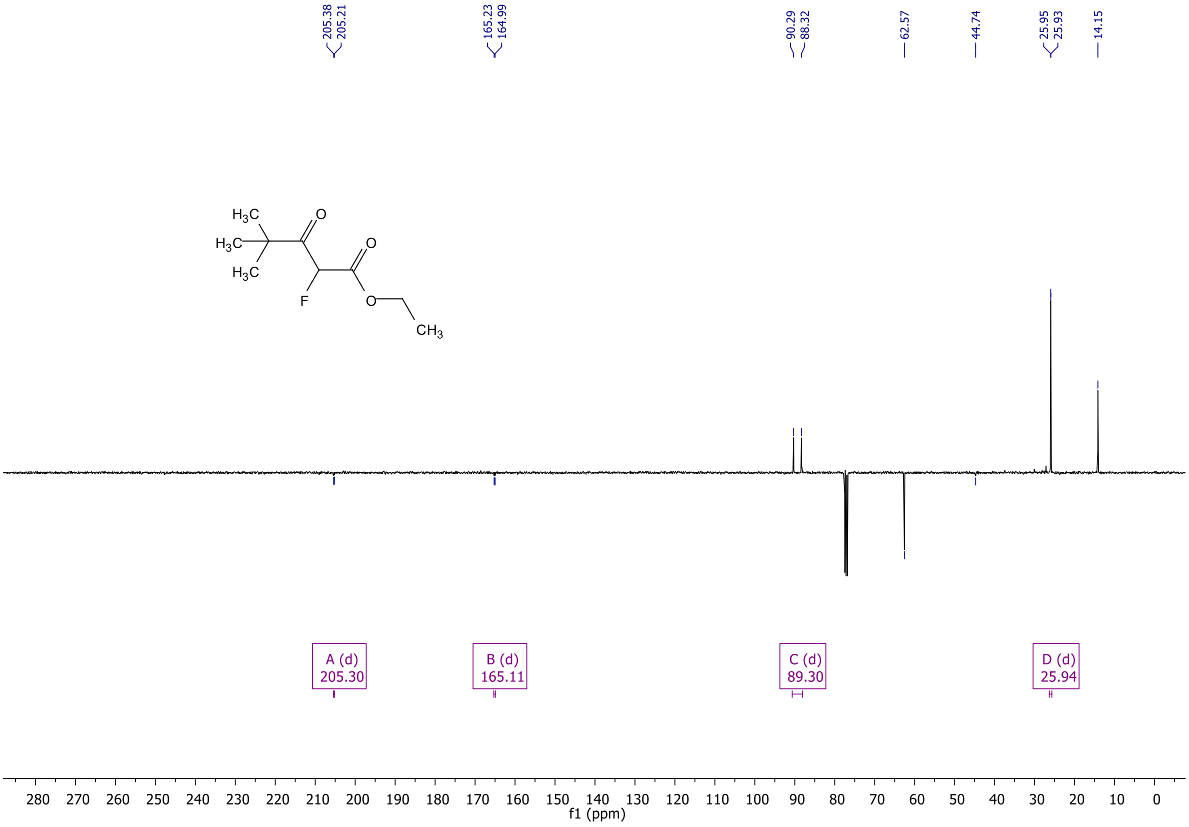

Ethyl 3-cyclohexyl-2-fluoro-3-oxopropanoate (1q)

^{1}H NMR, $CDCl_3$, 400 MHz

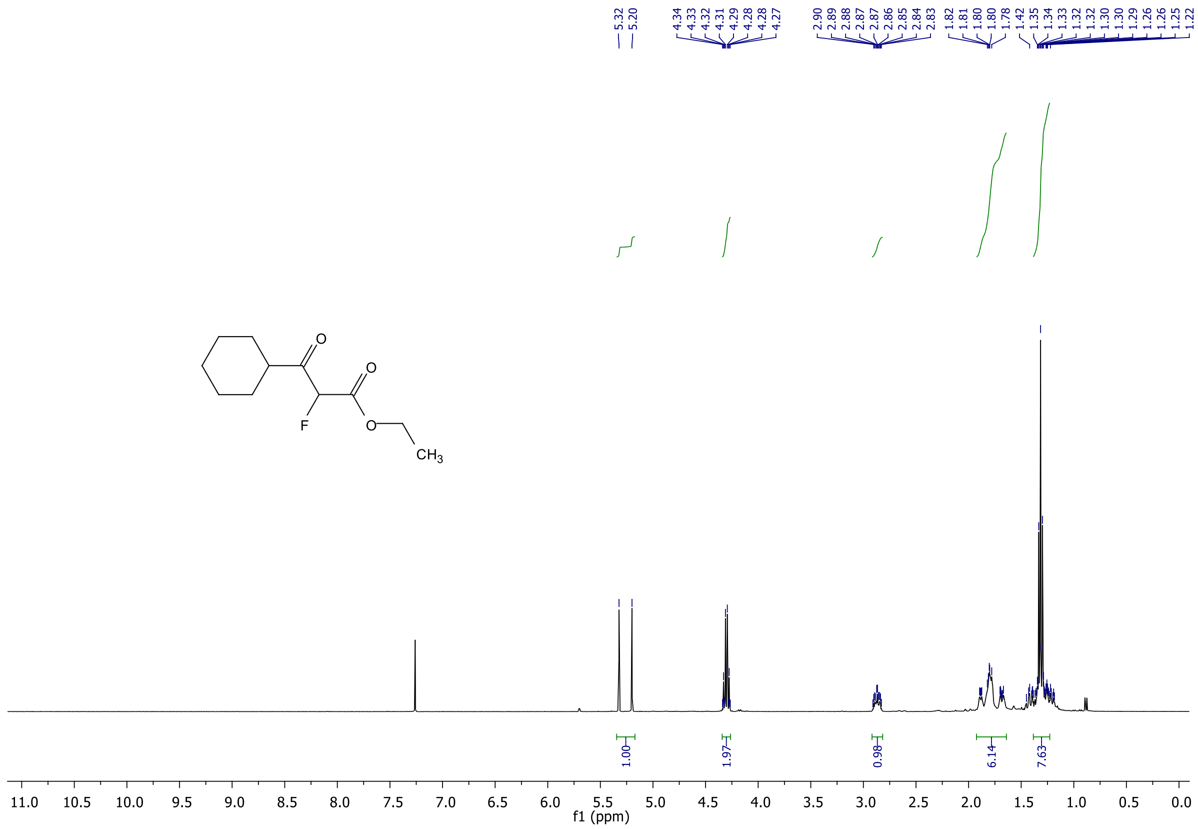

^{19}F NMR, $CDCl_3$, 377 MHz

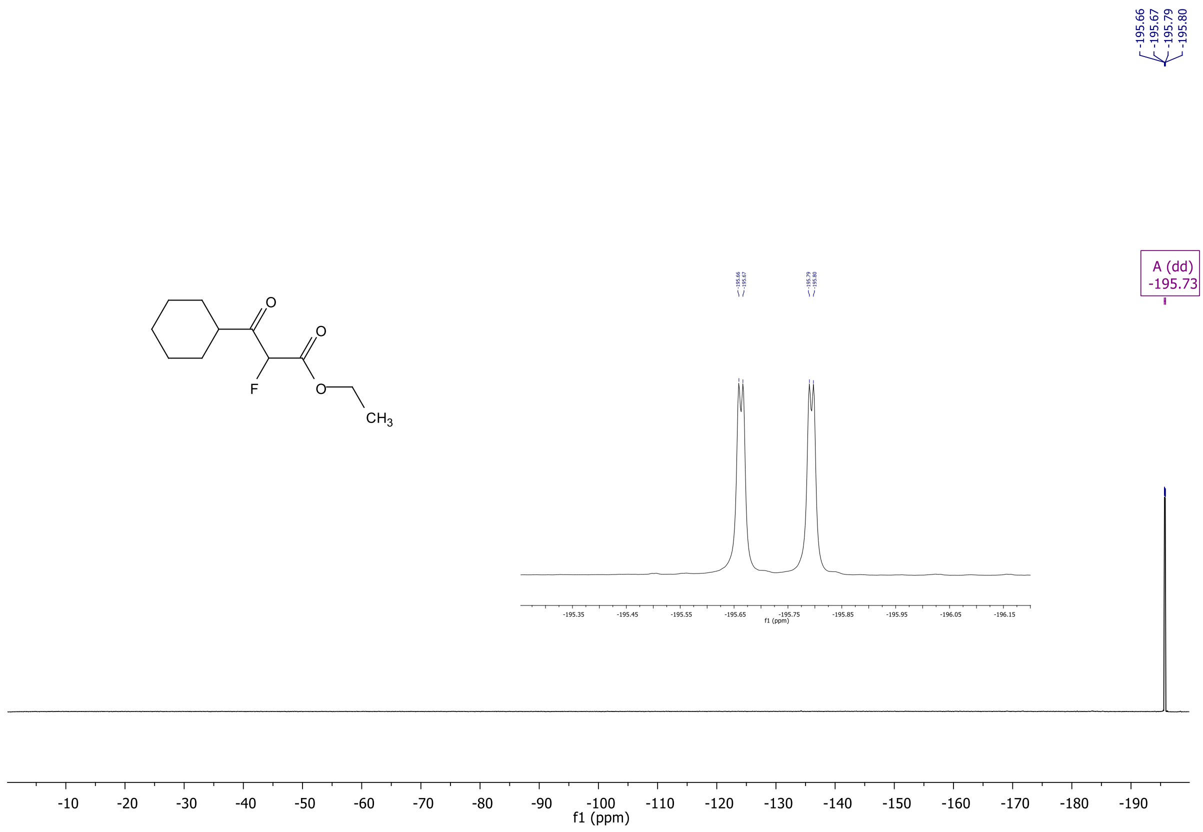

^{13}C NMR, $CDCl_3$, 101 MHz

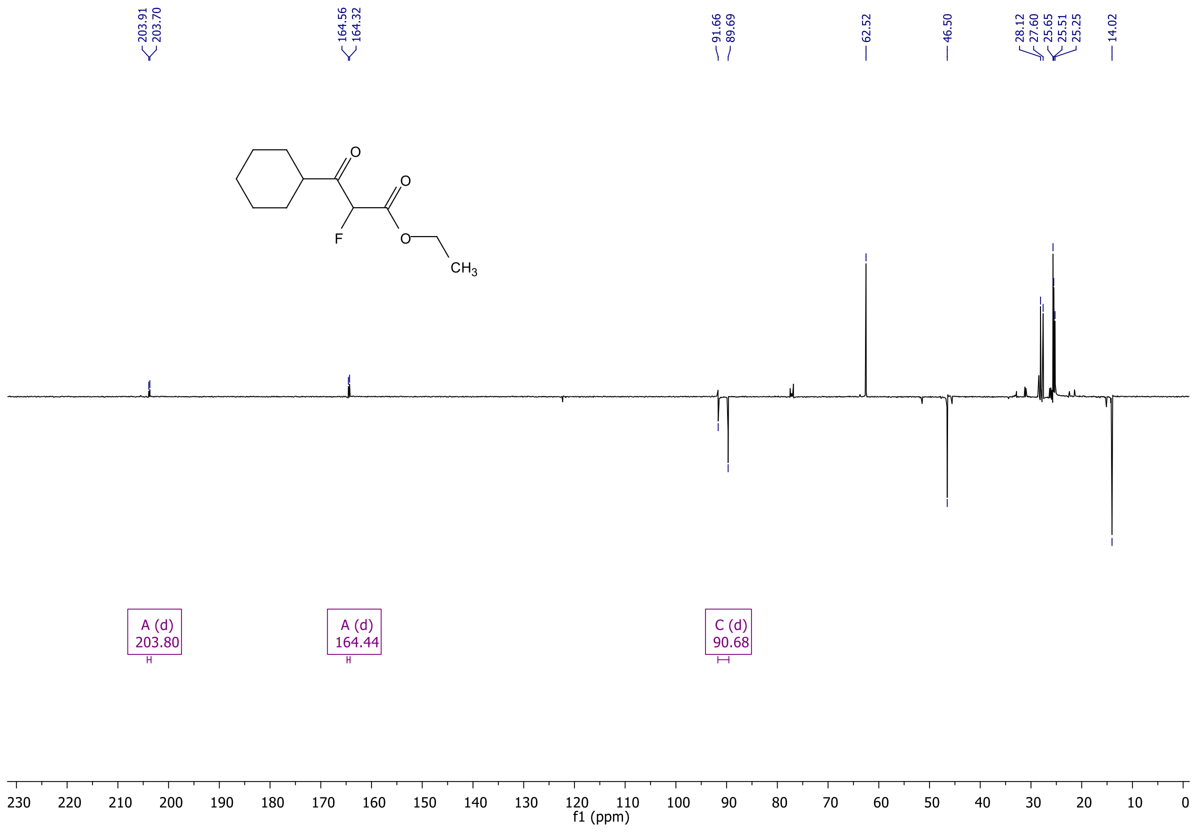

Ethyl 2-fluoro-3-oxooct-7-enoate (1r)

^{1}H NMR, $CDCl_3$, 400 MHz

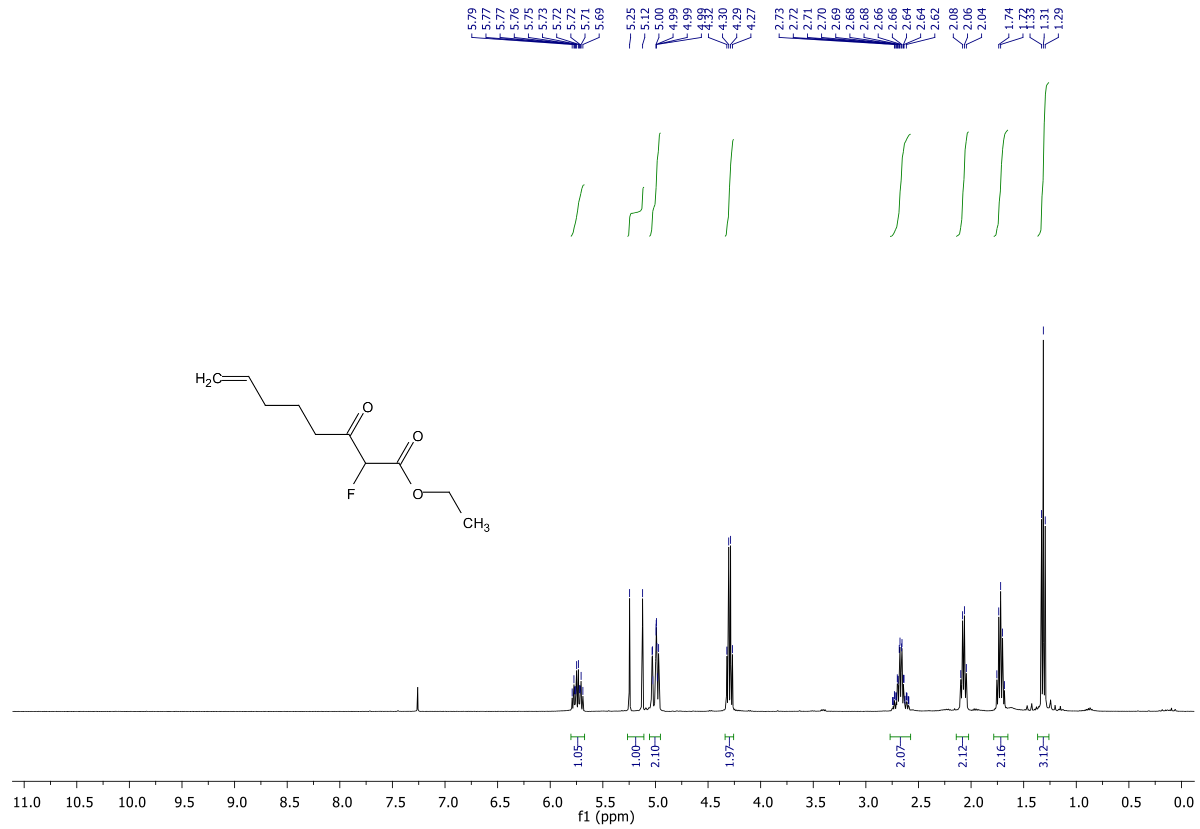

^{19}F NMR, $CDCl_3$, 377 MHz

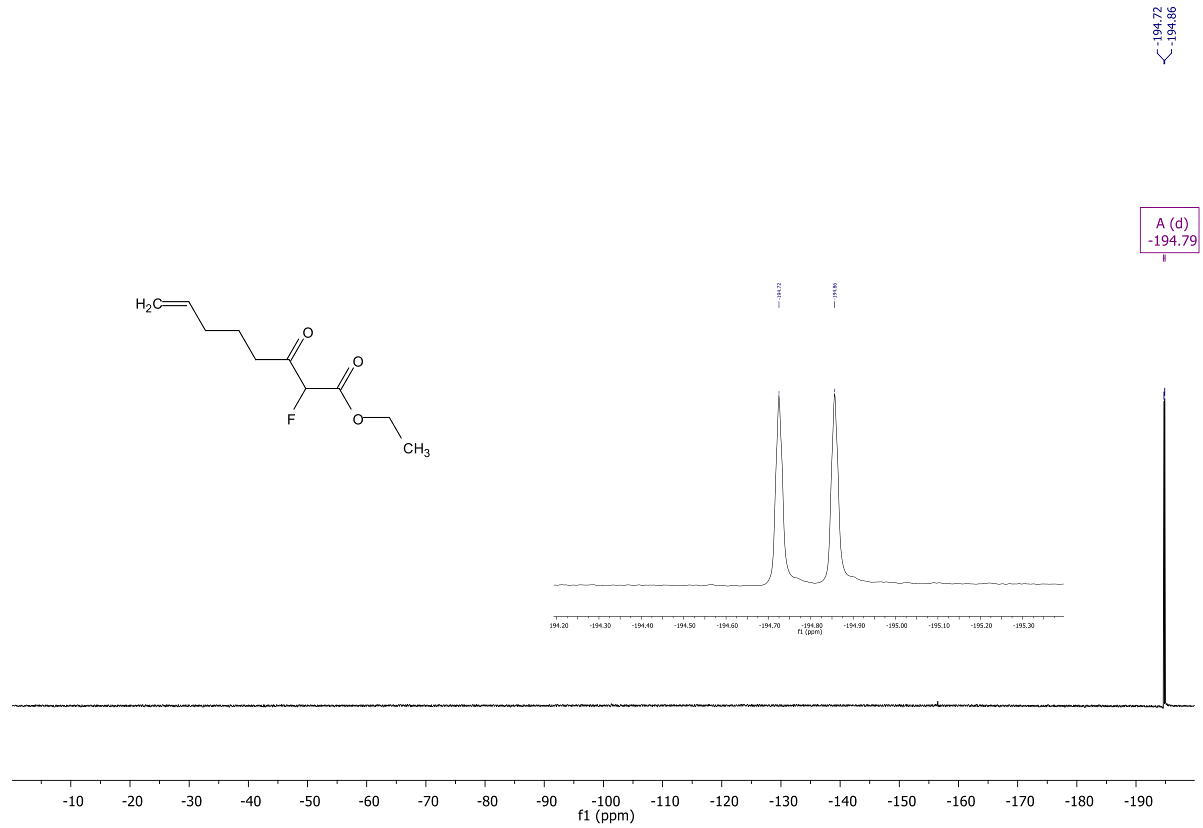

^{13}C NMR, $CDCl_3$, 101 MHz

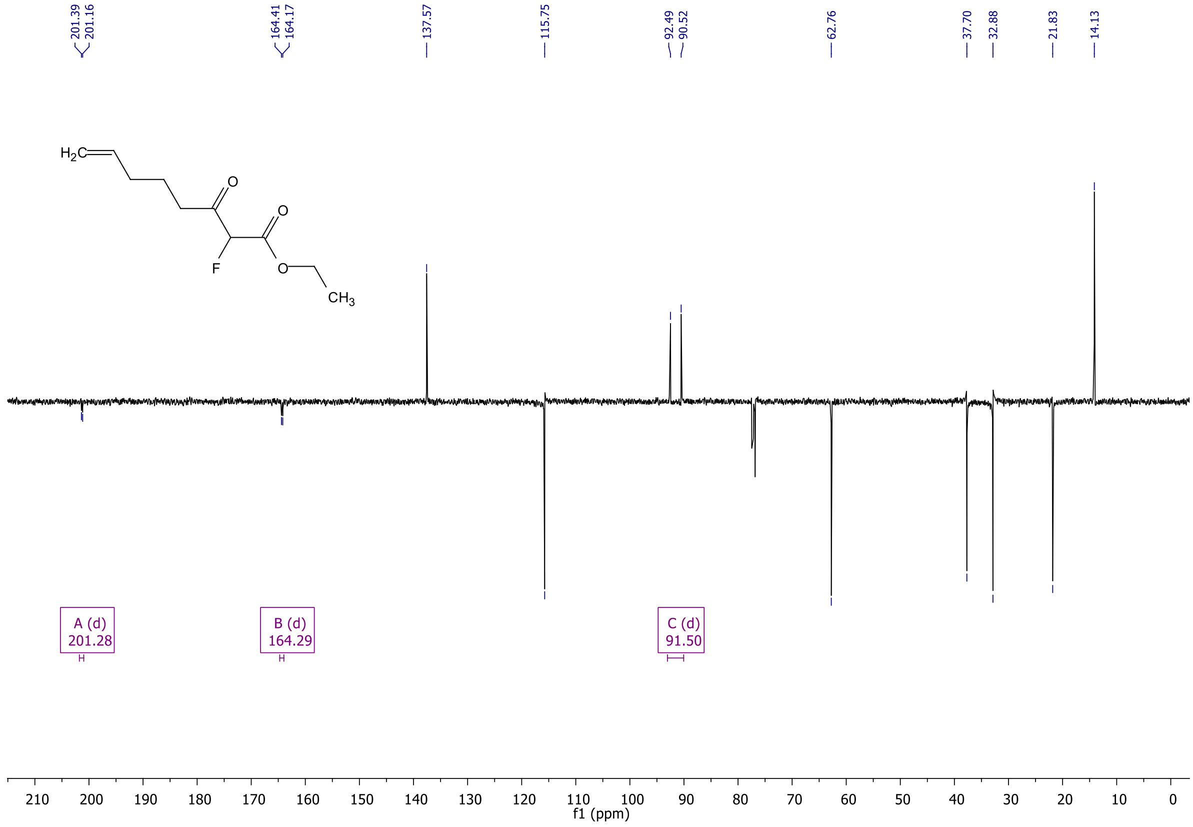

Ethyl 2-fluoro-3-oxo-3-(1-tosylpyrrolidin-2-yl)propanoate (1s)

^{1}H NMR, $CDCl_3$, 400 MHz

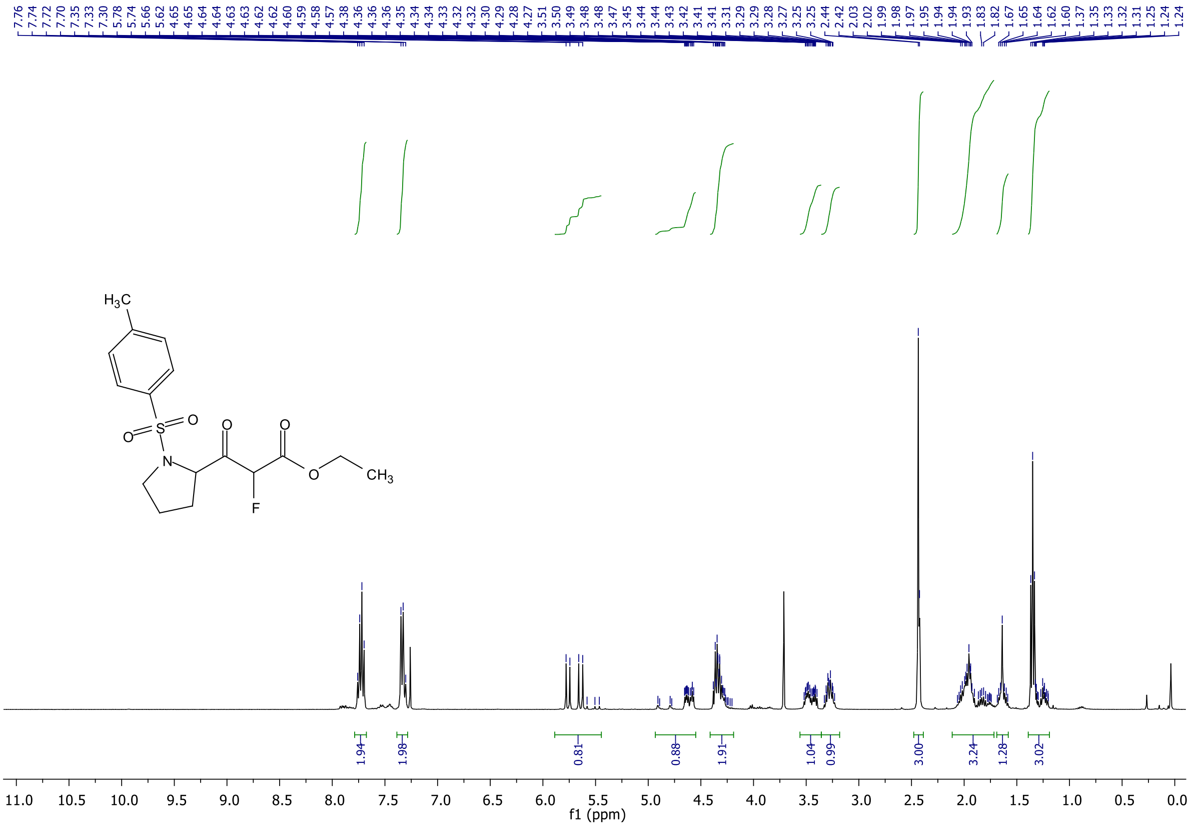

^{19}F NMR, $CDCl_3$, 377 MHz

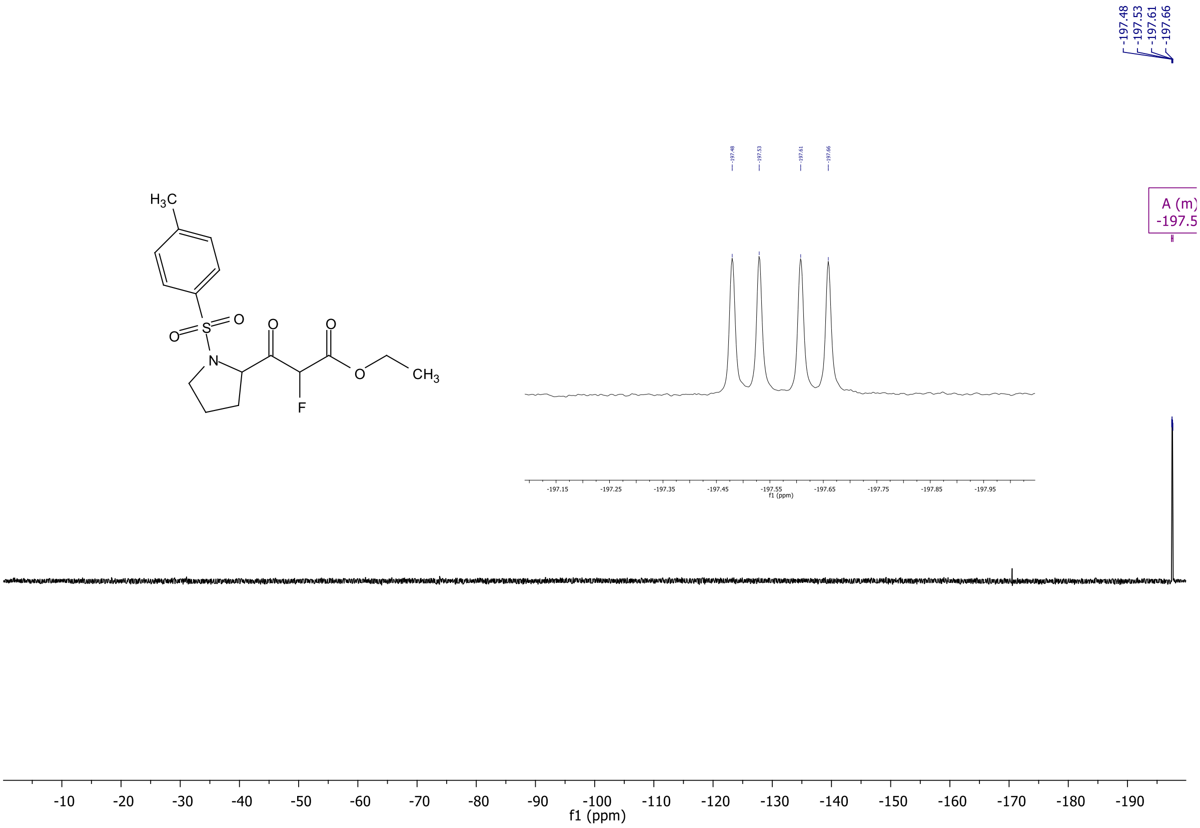

^{13}C NMR, $CDCl_3$, 101 MHz

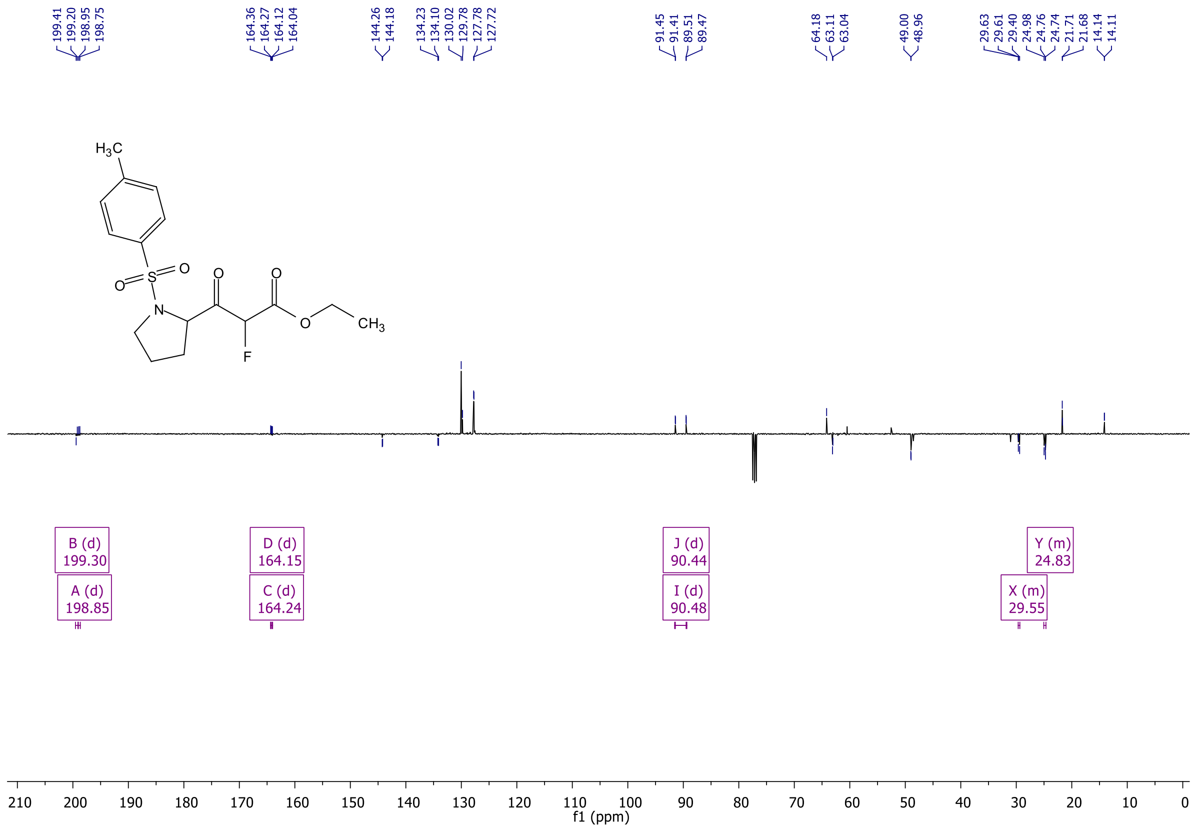

Ethyl 2-benzoyl-4-{[(*tert*-butoxycarbonyl)amino]methyl}-2-fluoropent-4-enoate (3a)

^{1}H NMR, $CDCl_3$, 400 MHz

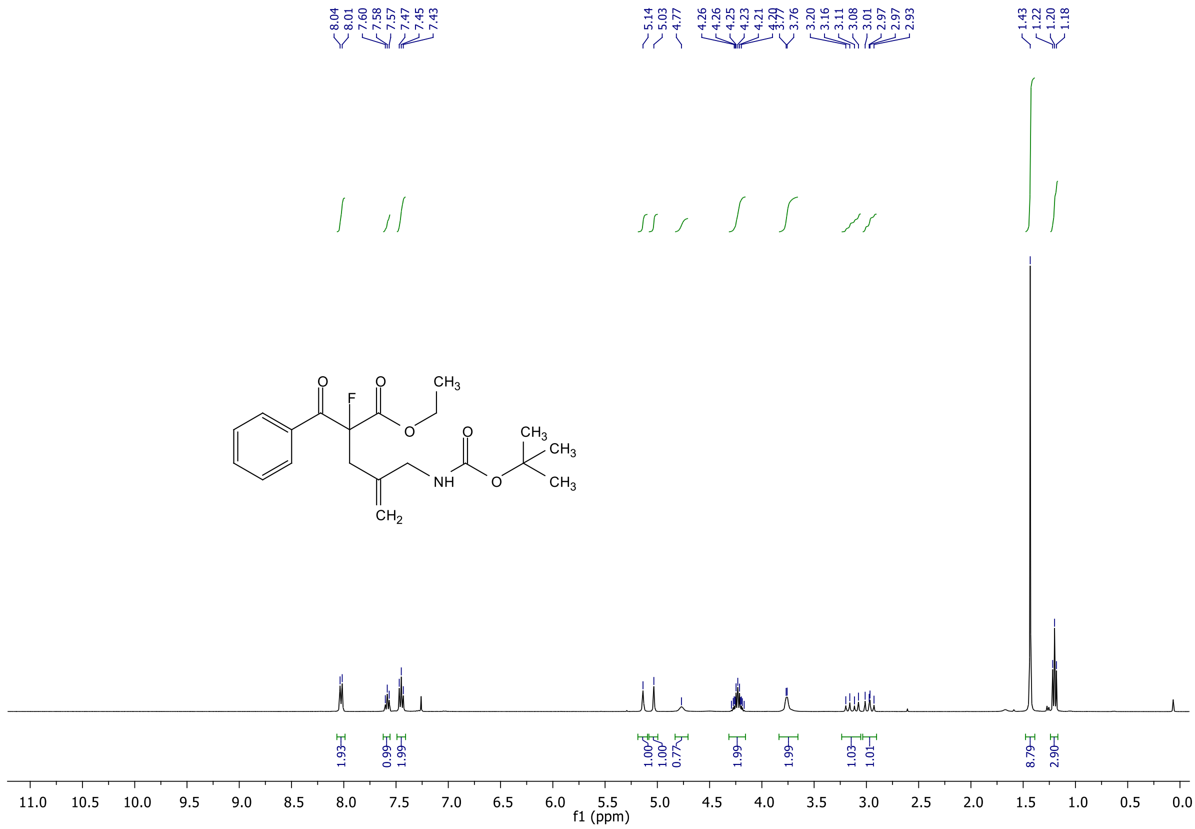

^{19}F NMR, $CDCl_3$, 377 MHz

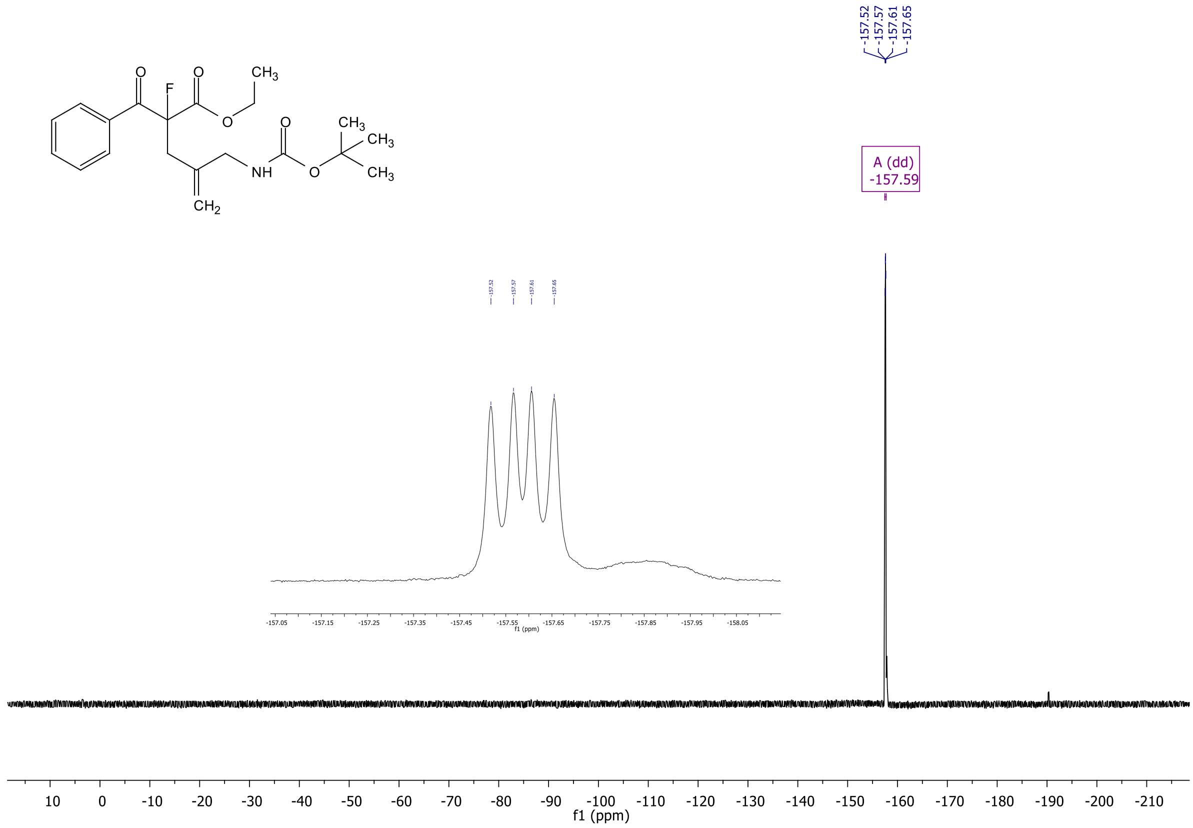

^{13}C NMR, $CDCl_3$, 101 MHz

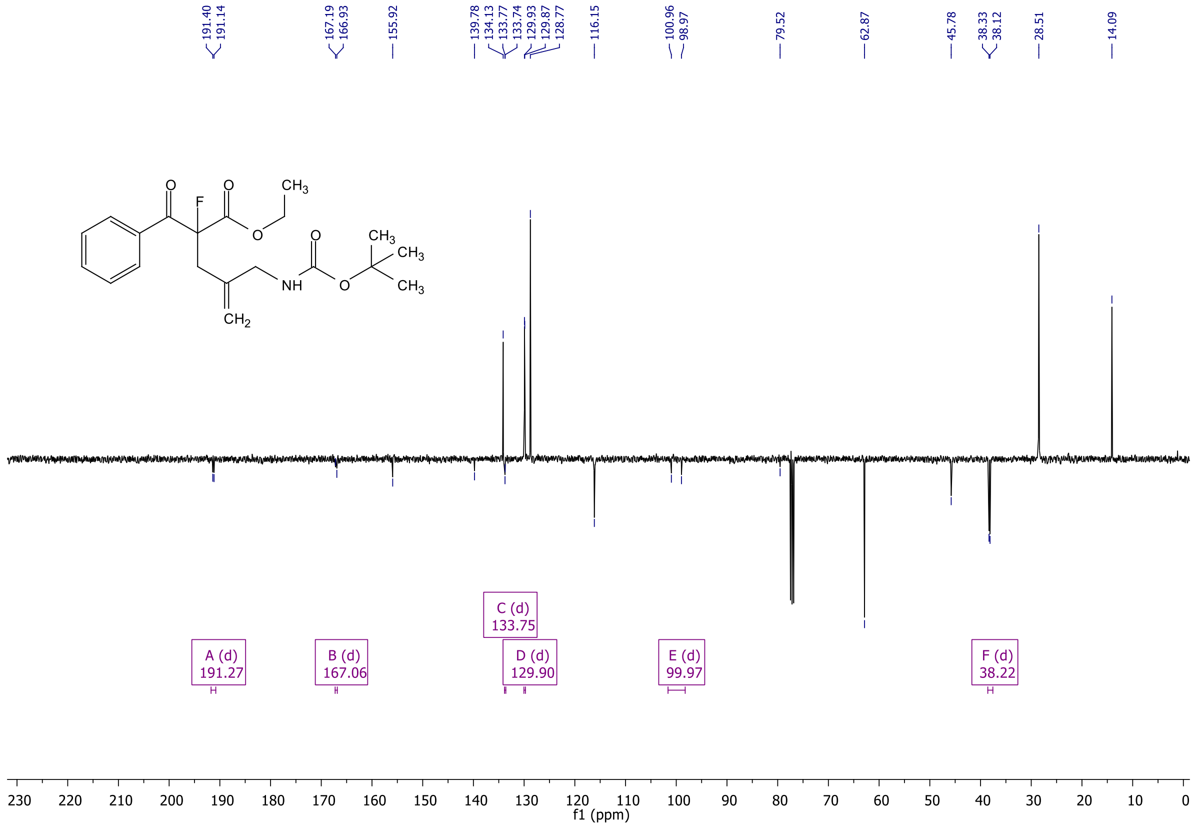

Ethyl 4-{[(*tert*-butoxycarbonyl)amino]methyl}-2-fluoro-2-(4-methoxybenzoyl)pent-4-enoate (3b)

^{1}H NMR, CDCl$_3$, 400 MHz

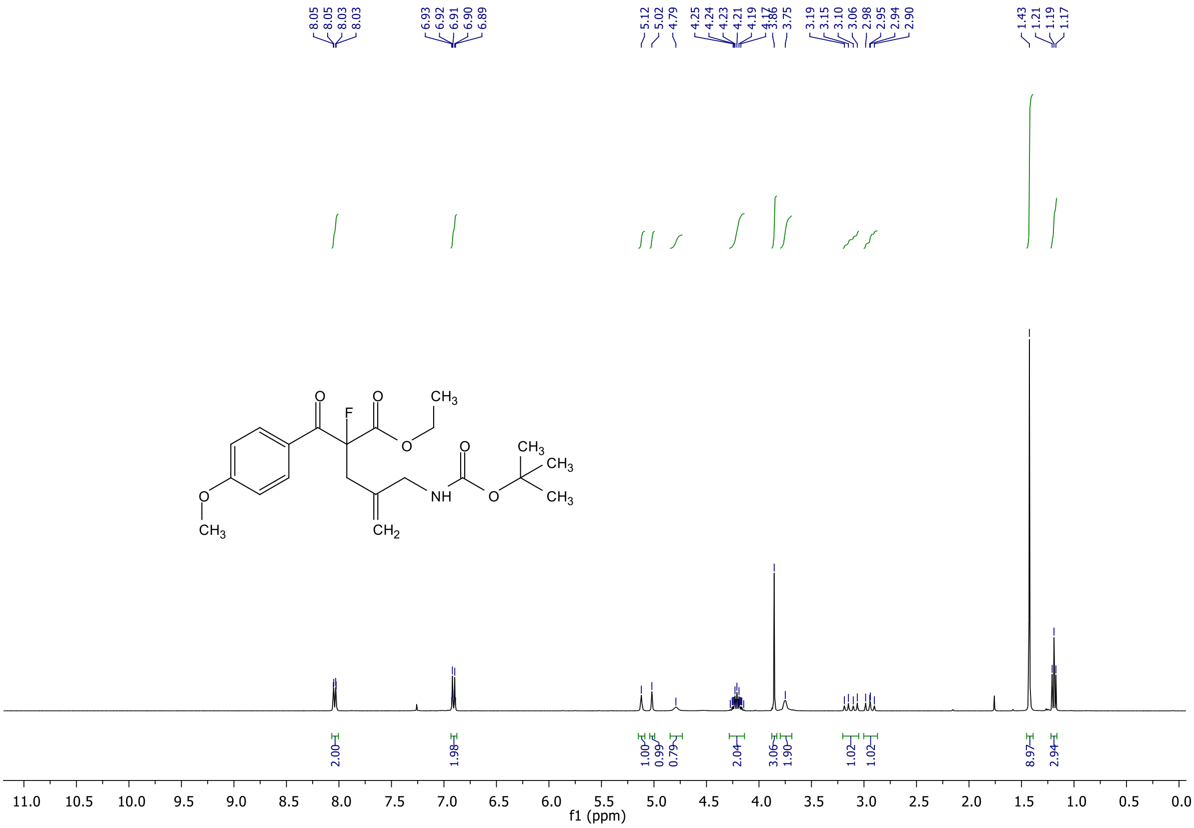

^{19}F NMR, CDCl$_3$, 377 MHz

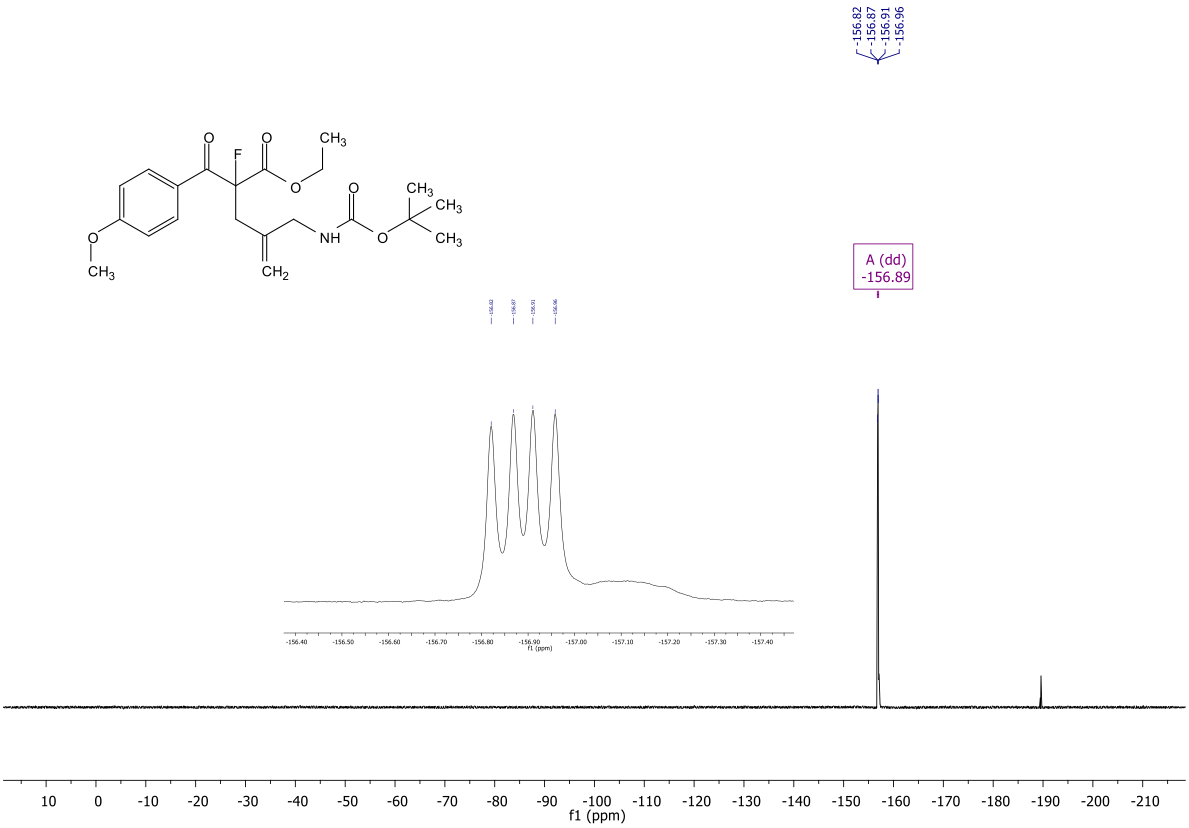

^{13}C NMR, $CDCl_3$, 101 MHz

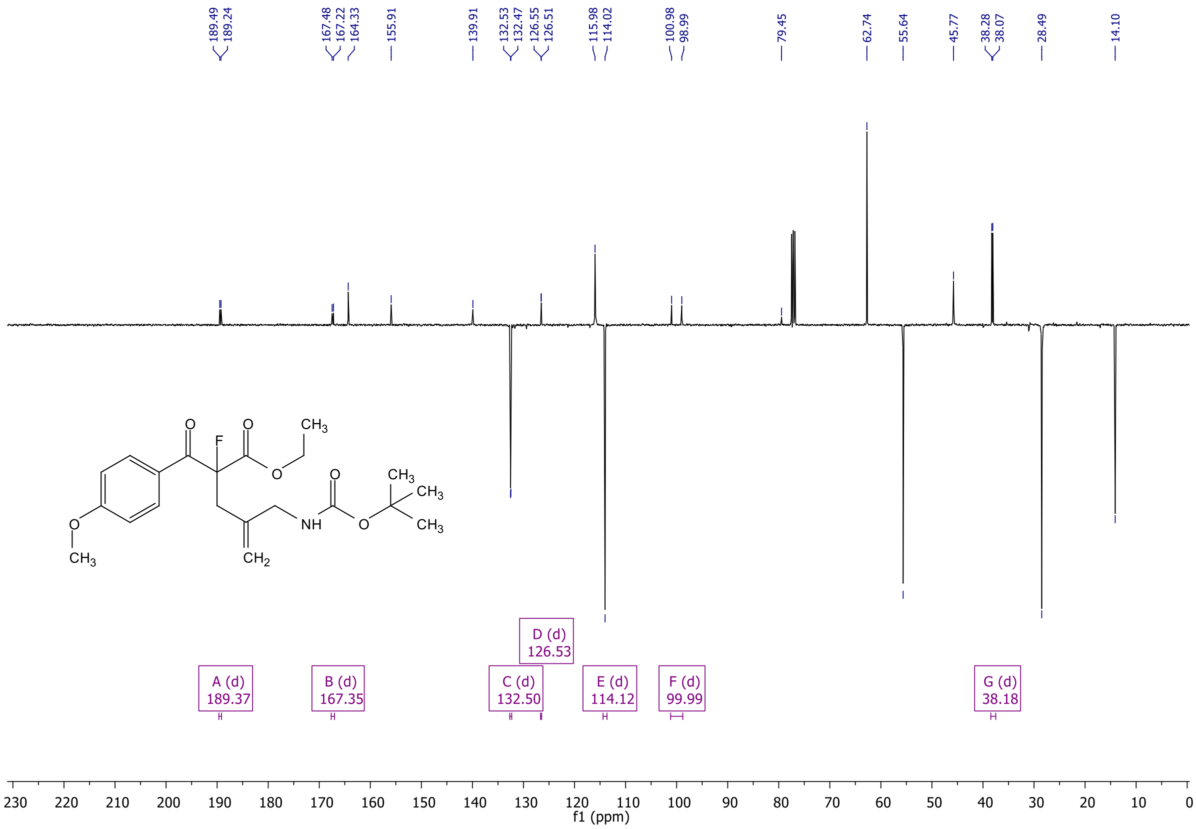

Ethyl 4-{[(*tert*-butoxycarbonyl)amino]methyl}-2-fluoro-2-(4-chloromethylbenzoyl)pent-4-enoate (3c)

^{1}H NMR, $CDCl_3$, 400 MHz

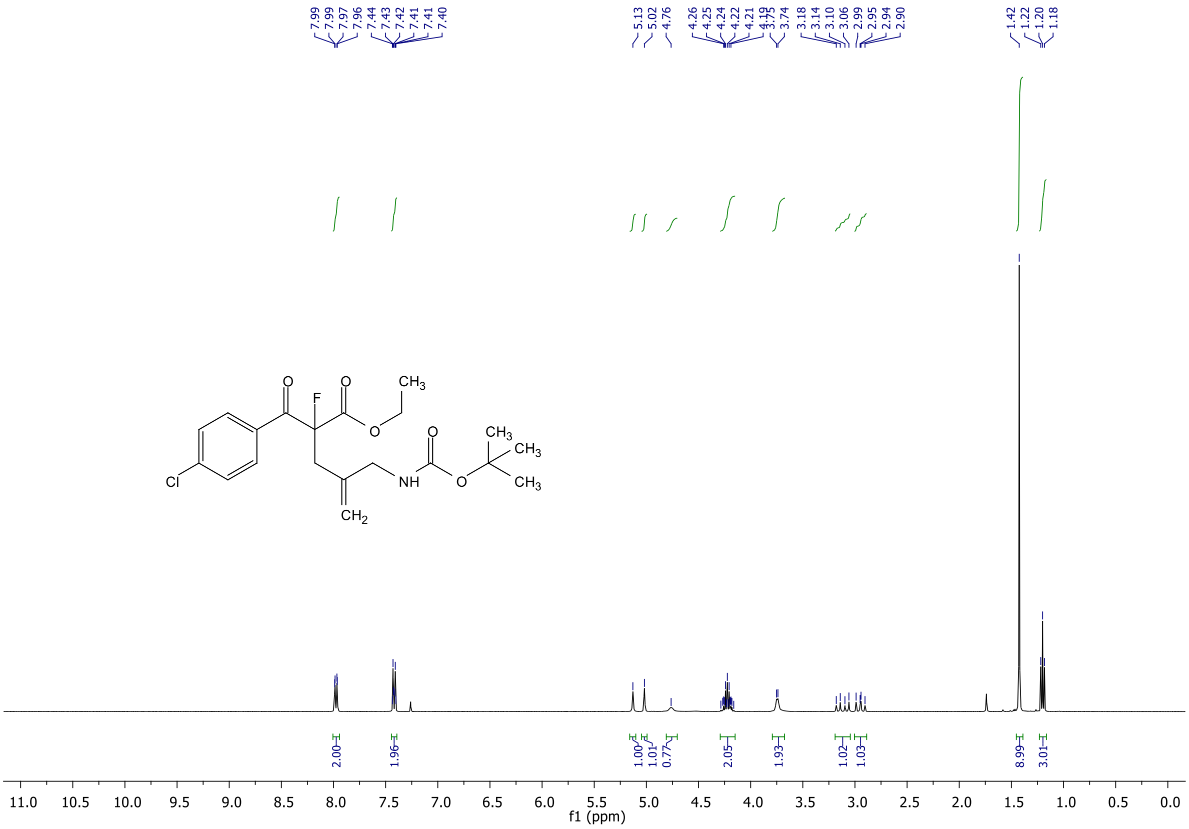

^{19}F NMR, $CDCl_3$, 377 MHz

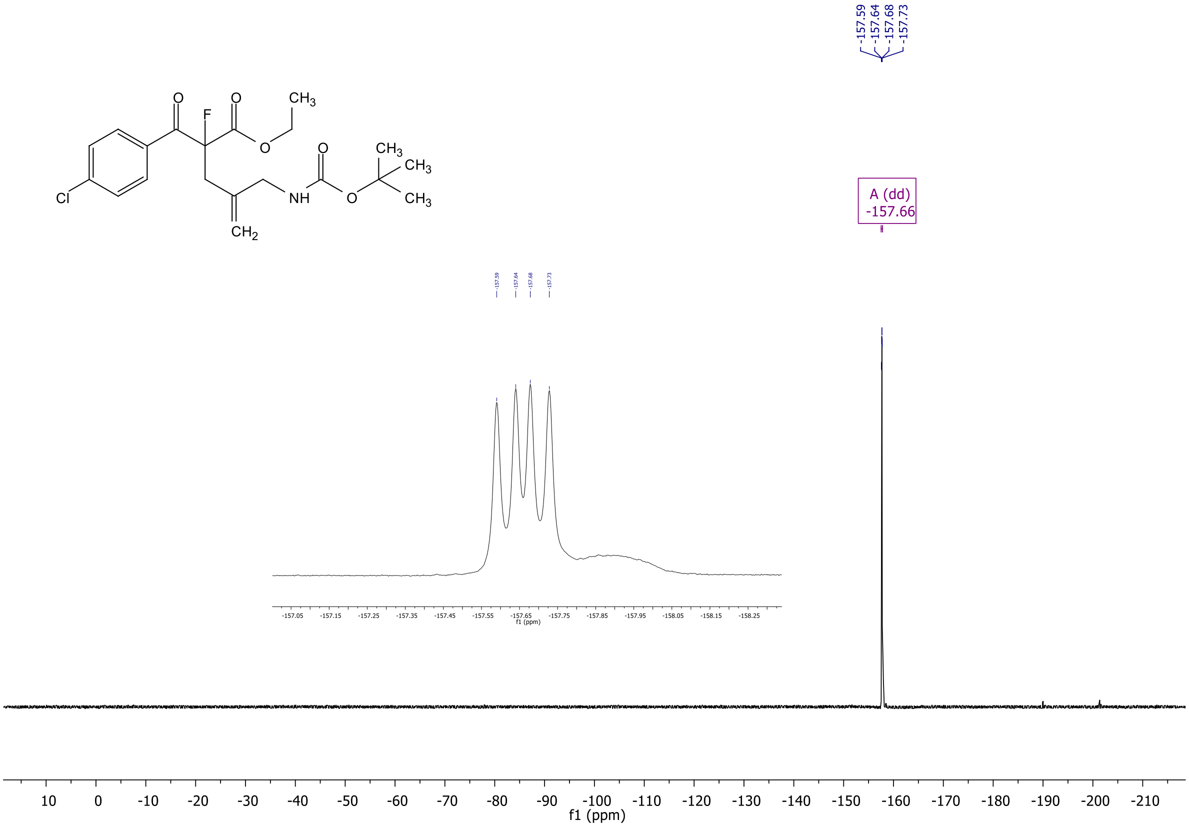

^{13}C NMR, $CDCl_3$, 101 MHz

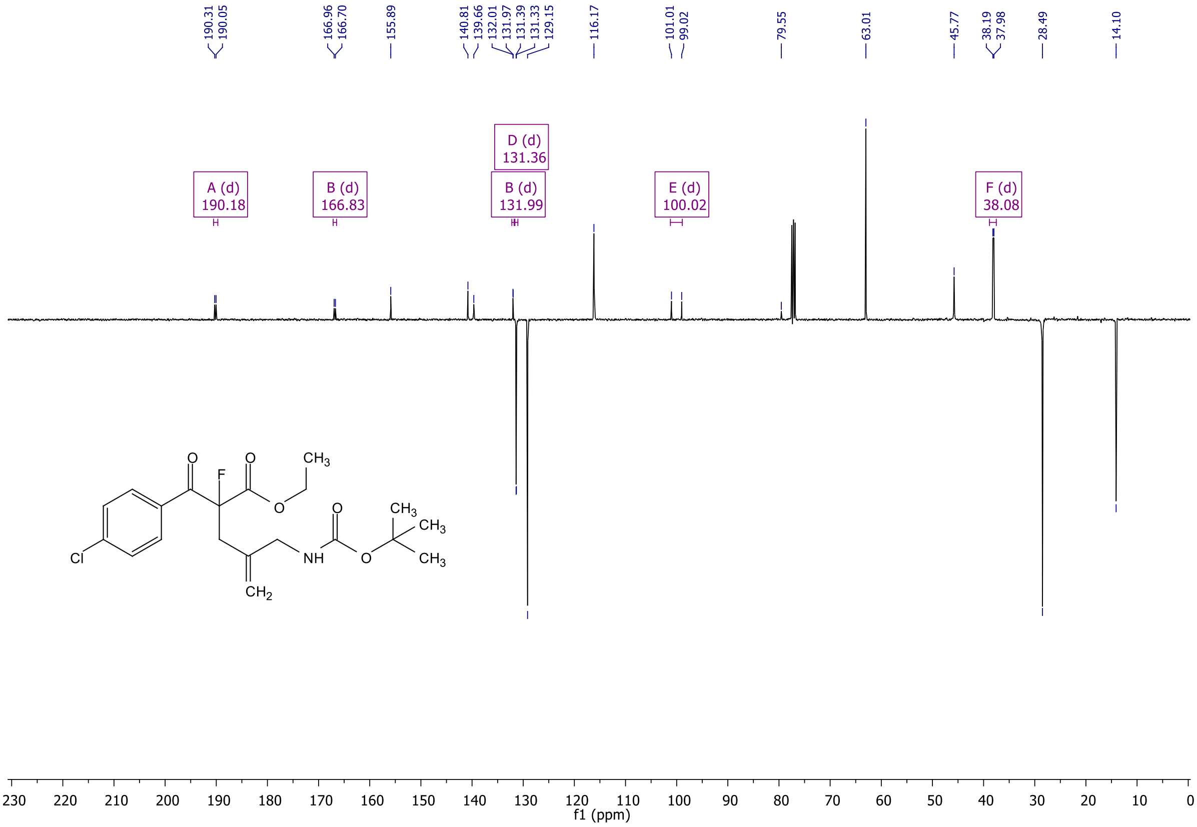

Ethyl 4-{[(*tert*-butoxycarbonyl)amino]methyl}-2-fluoro-2-(4-trifluoromethylbenzoyl) pent-4-enoate (3d)

^{1}H NMR, CDCl$_3$, 400 MHz

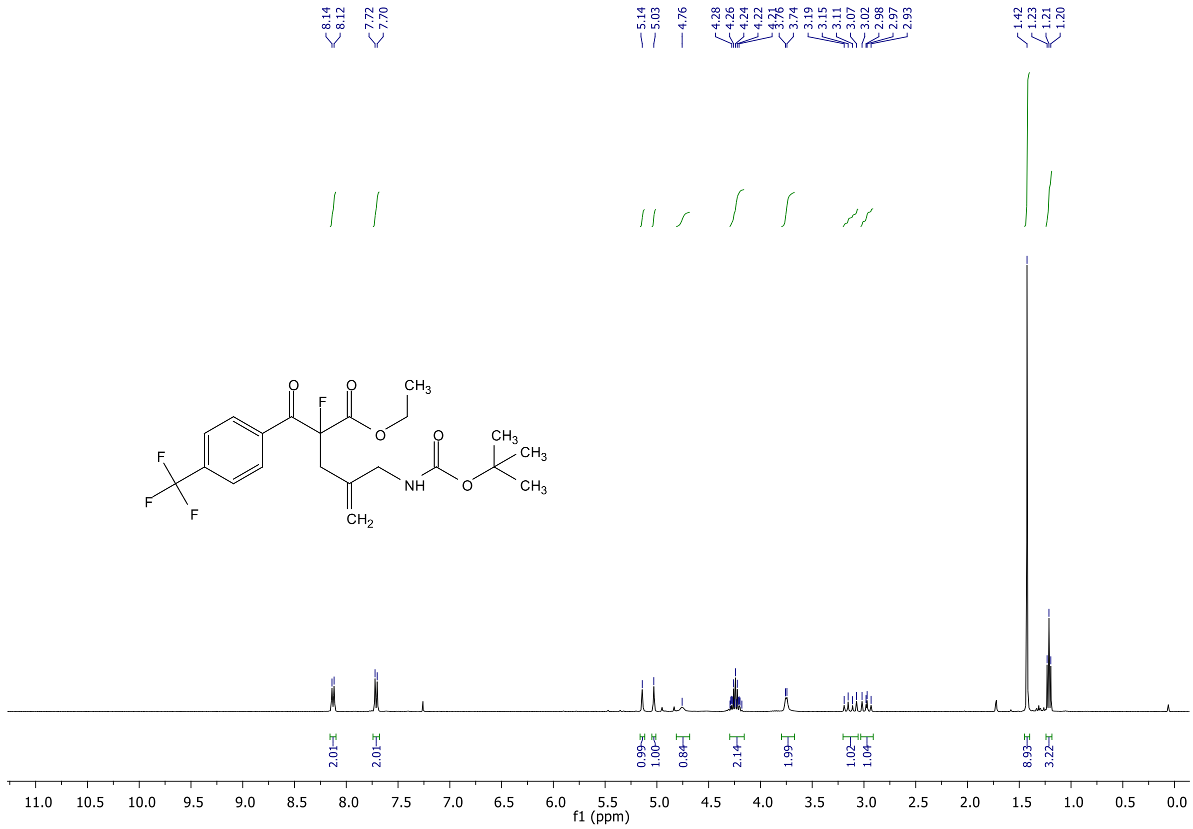

^{19}F NMR, CDCl$_3$, 377 MHz

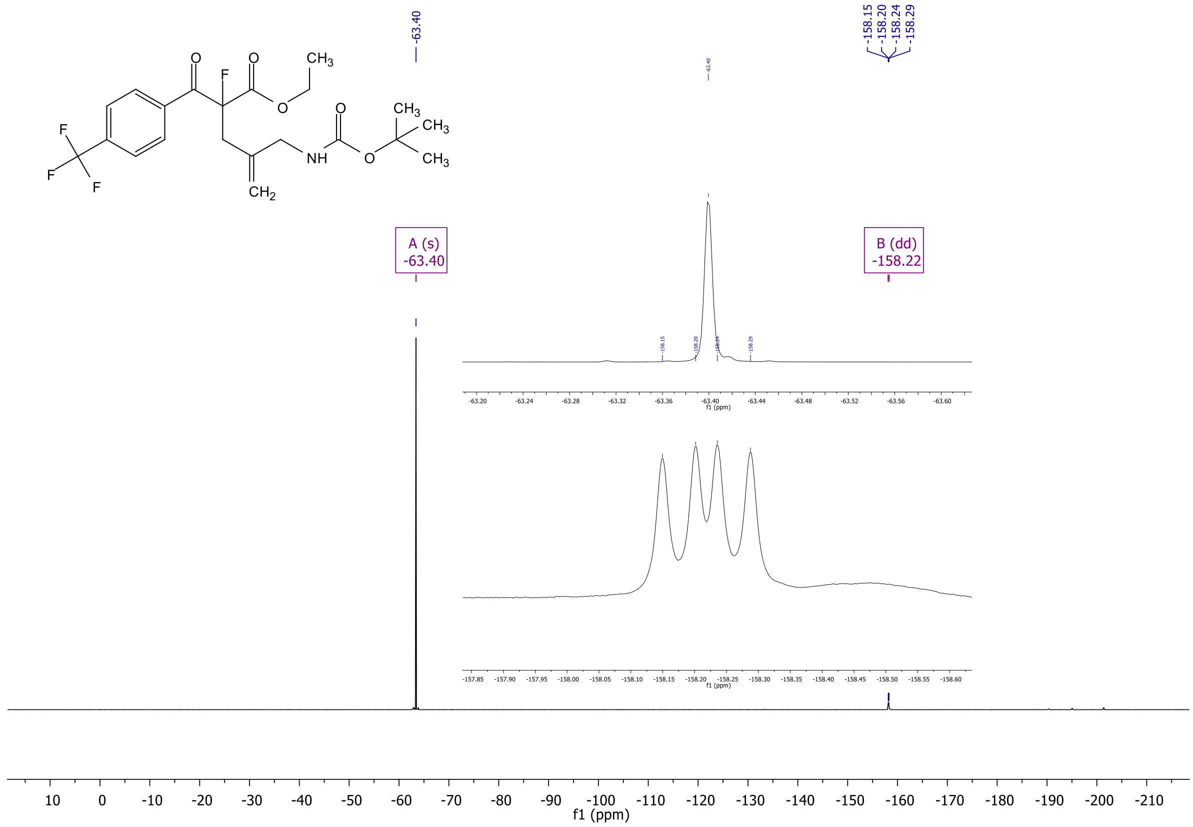

^{13}C NMR, $CDCl_3$, 101 MHz

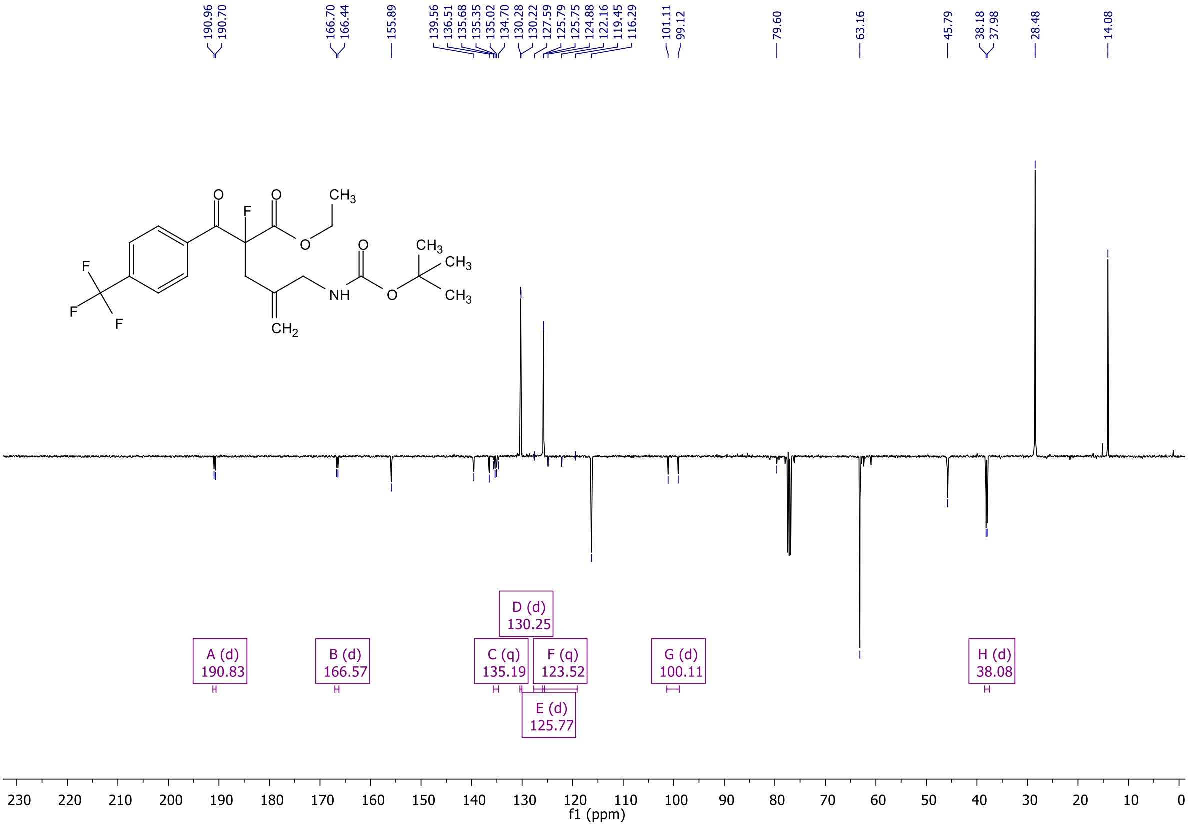

Ethyl 4-{[(*tert*-butoxycarbonyl)amino]methyl}-2-fluoro-2-(4-methylbenzoyl)pent-4-enoate (3e)

^{1}H NMR, CDCl$_3$, 400 MHz

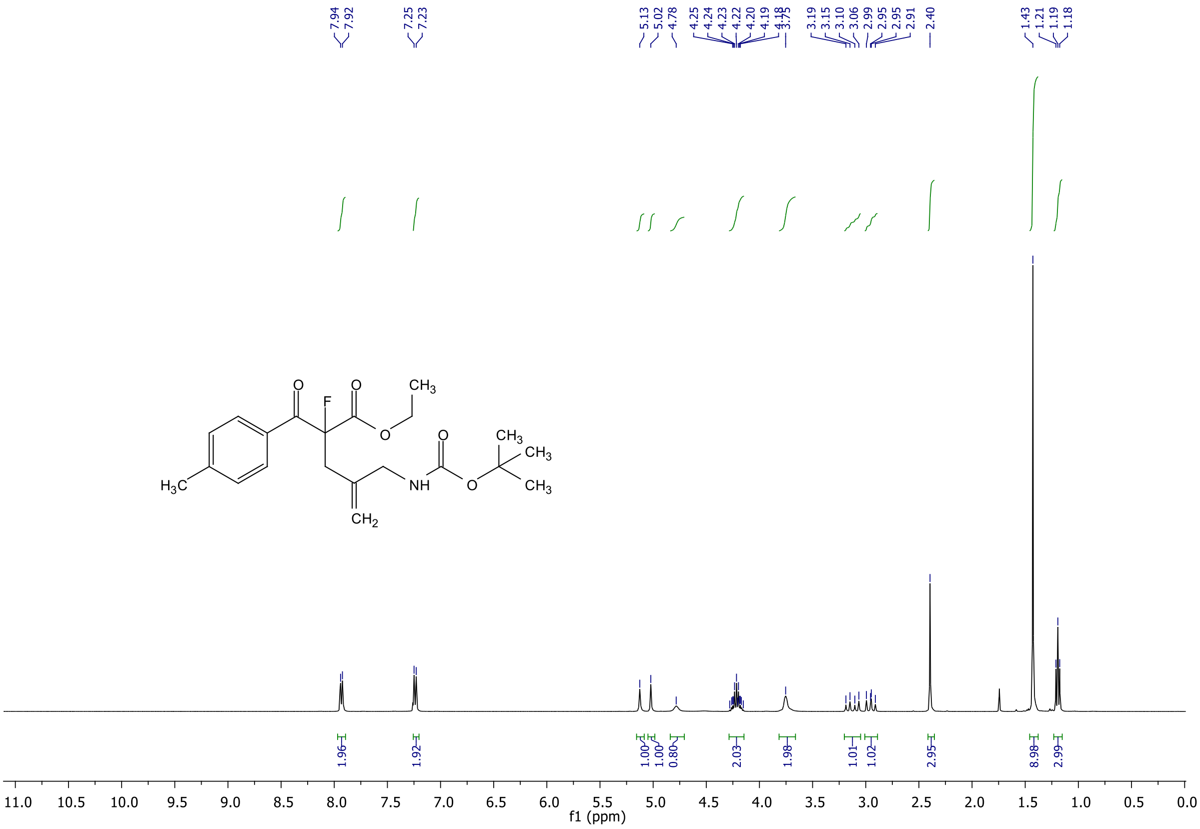

^{19}F NMR, CDCl$_3$, 377 MHz

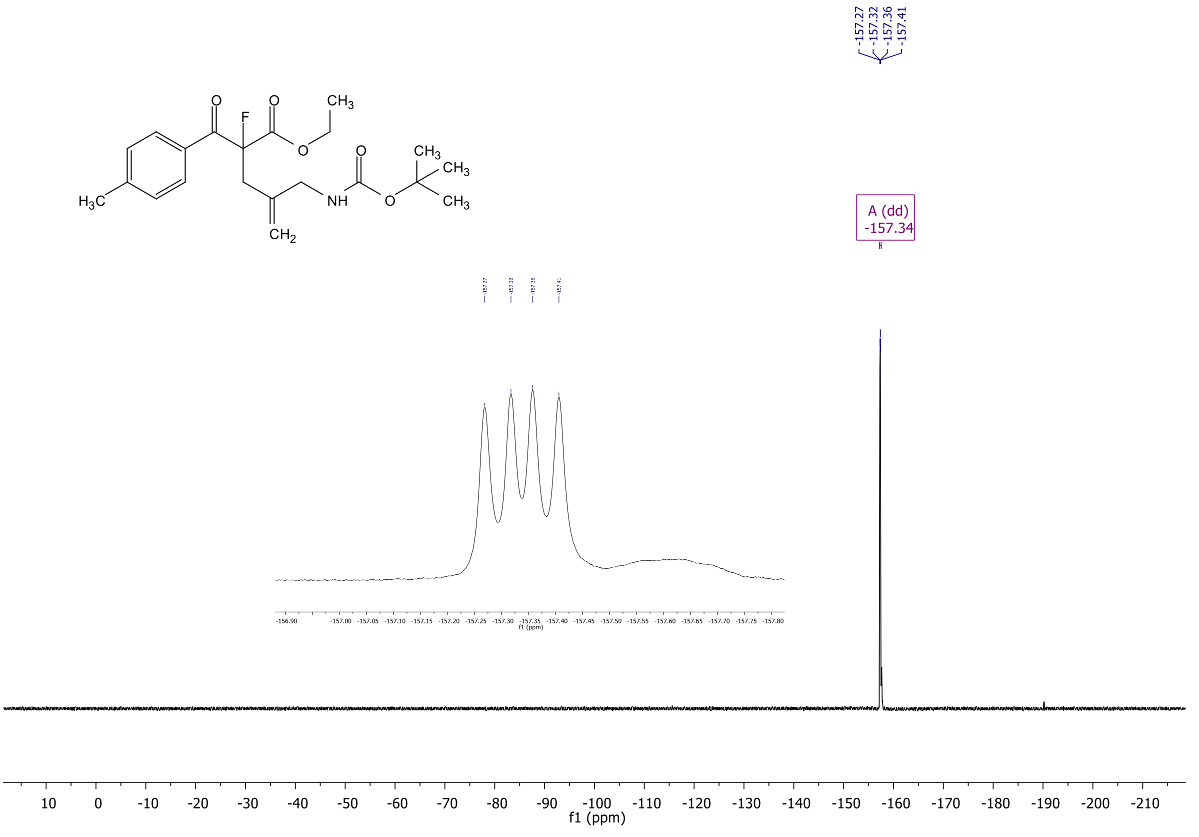

^{13}C NMR, $CDCl_3$, 101 MHz

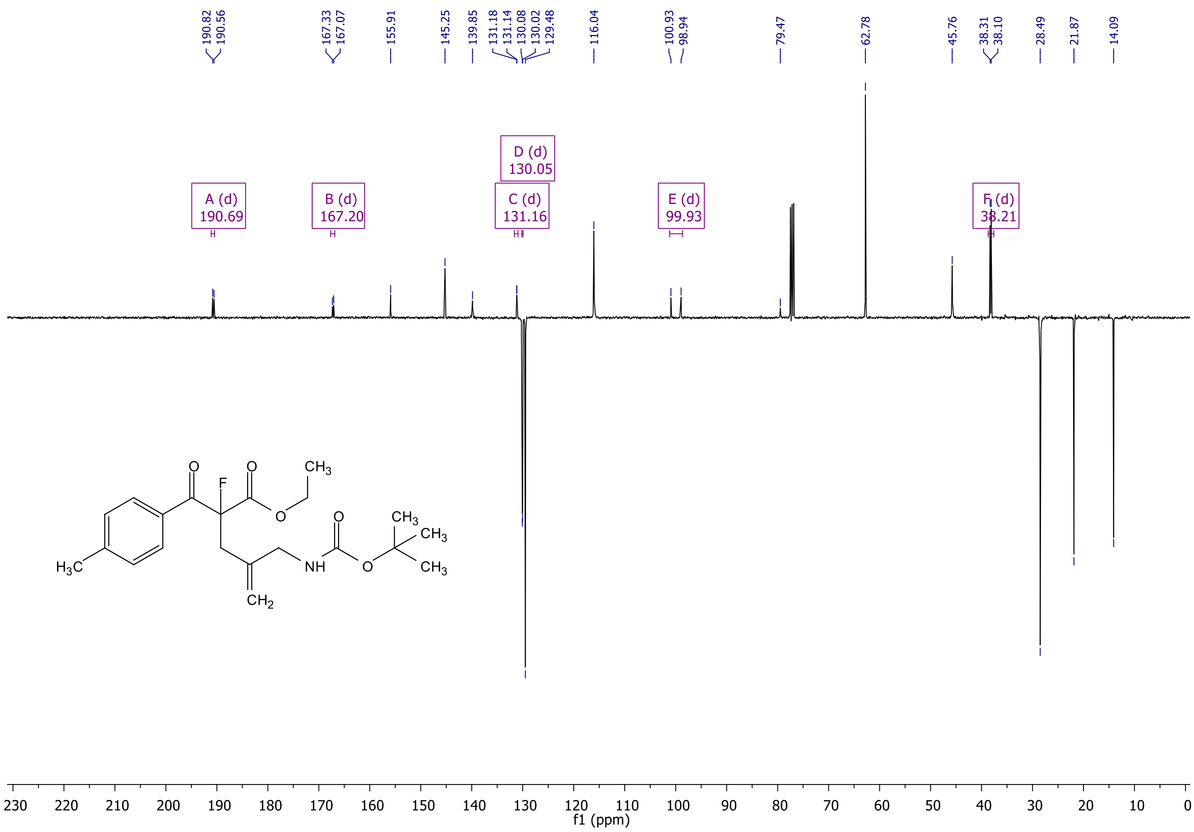

Ethyl 4-{[(*tert*-butoxycarbonyl)amino]methyl}-2-fluoro-2-(4-nitrobenzoyl)pent-4-enoate (3f)

^{1}H NMR, $CDCl_3$, 400 MHz

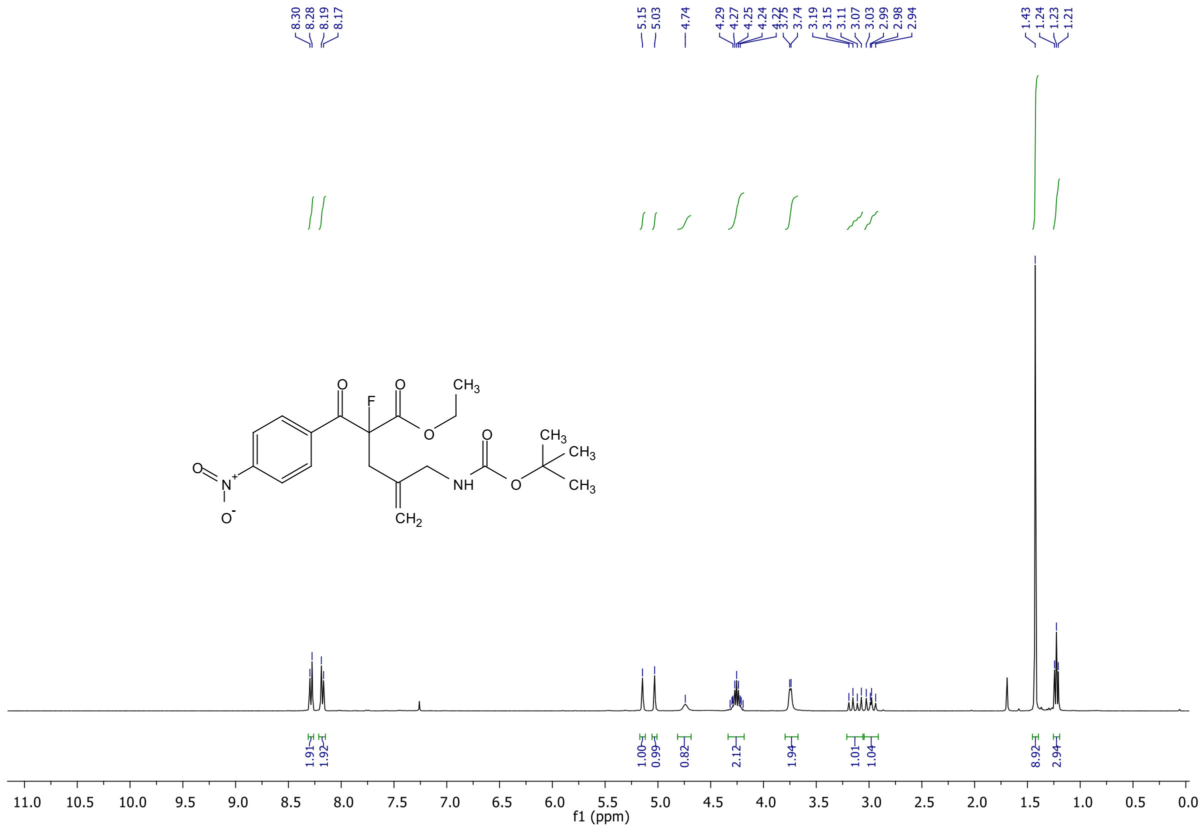

^{19}F NMR, $CDCl_3$, 377 MHz

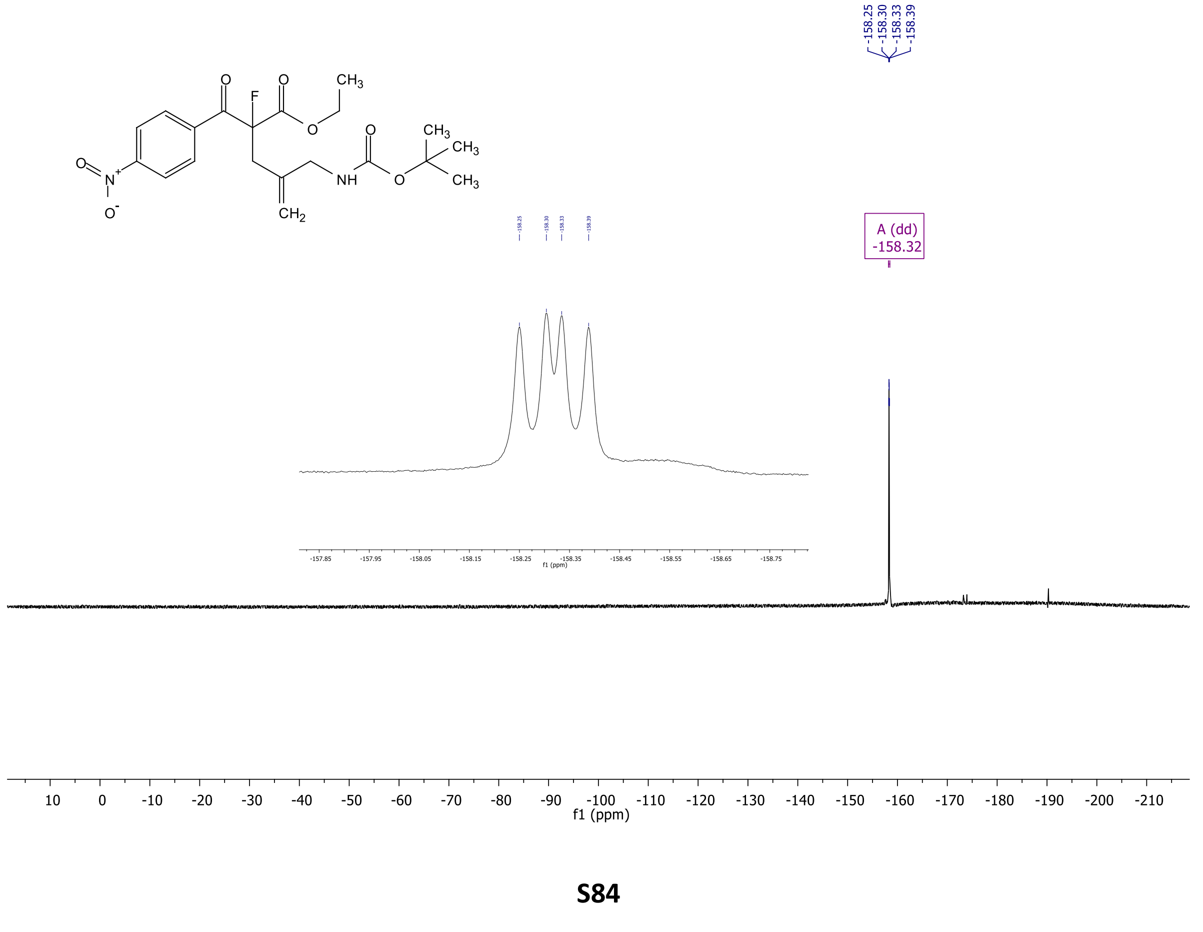

^{13}C NMR, $CDCl_3$, 101 MHz

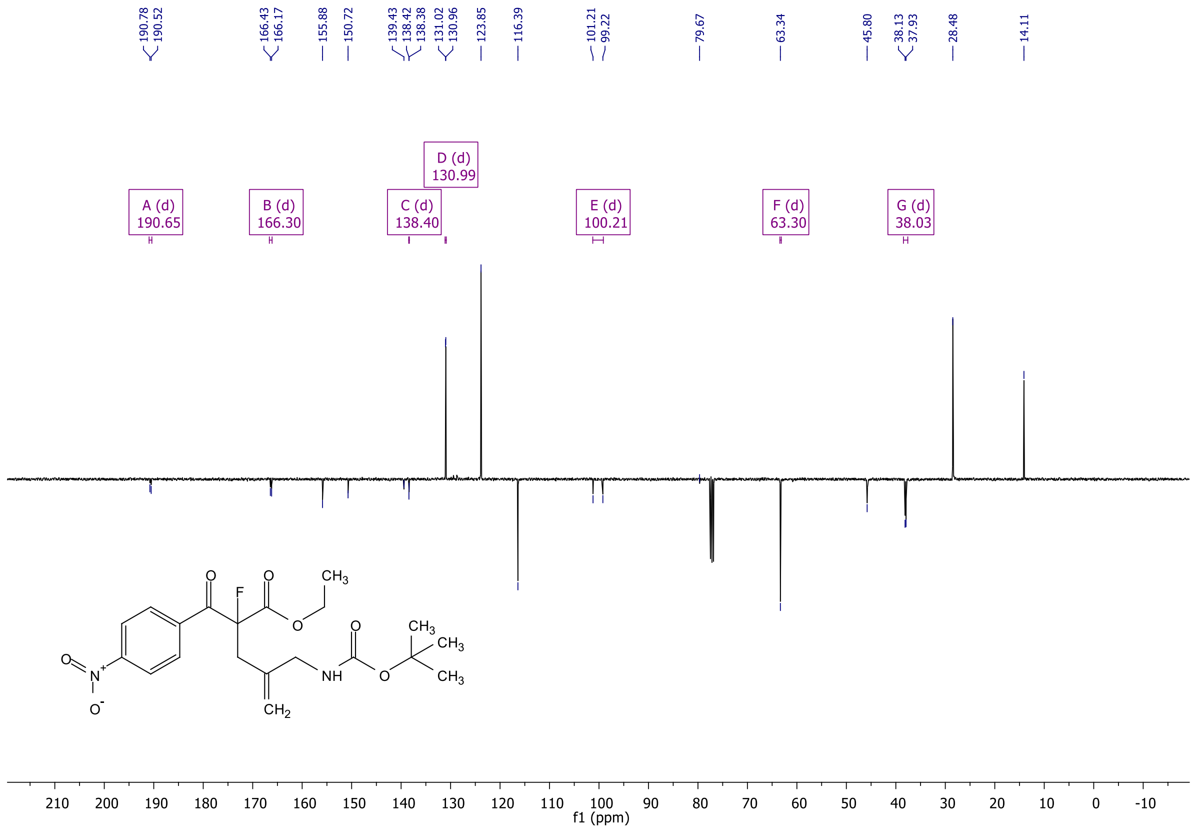

Ethyl 4-{[(*tert*-butoxycarbonyl)amino]methyl}-2-fluoro-2-(2-methylbenzoyl)pent-4-enoate (3g)

^{1}H NMR, $CDCl_3$, 400 MHz

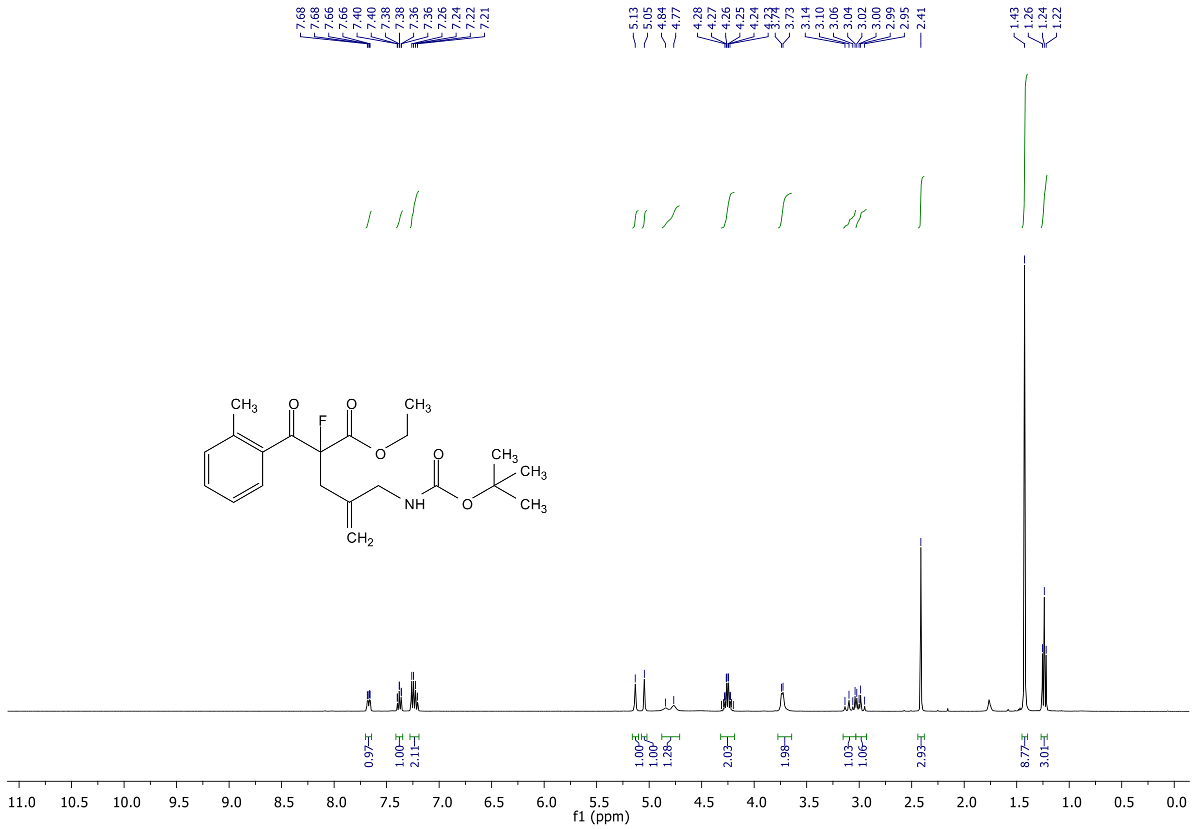

^{19}F NMR, $CDCl_3$, 377 MHz

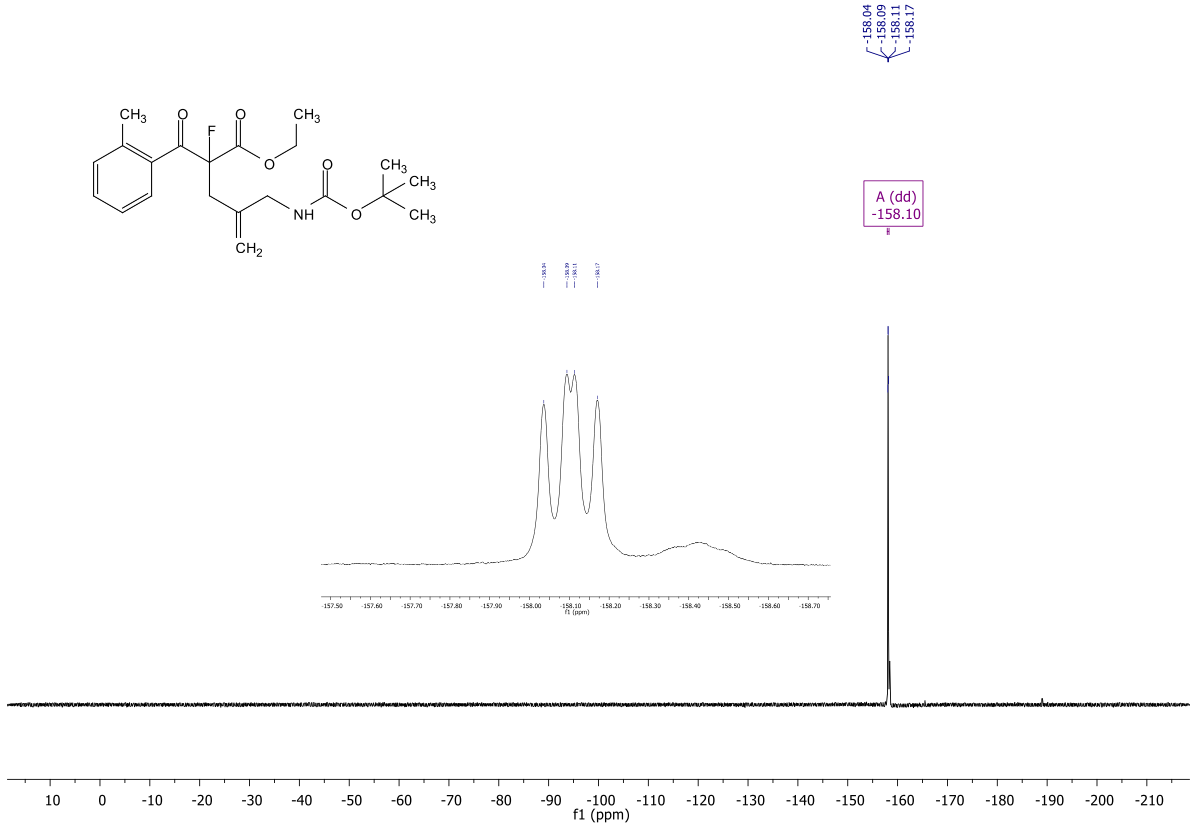

^{13}C NMR, $CDCl_3$, 101 MHz

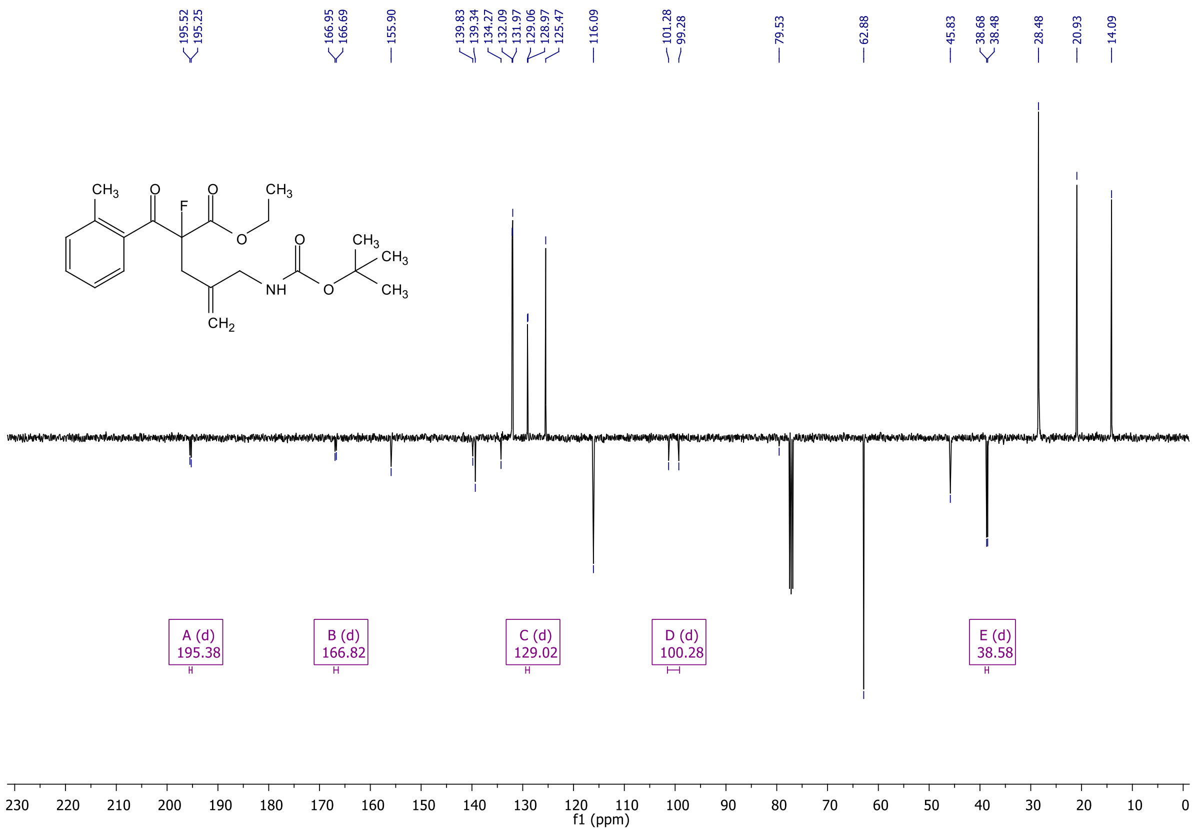

Ethyl 2-(2-naphthoyl)-4-(((*tert*-butoxycarbonyl)amino)methyl)-2-fluoropent-4-enoate (3h)

^{1}H NMR, CDCl$_3$, 400 MHz

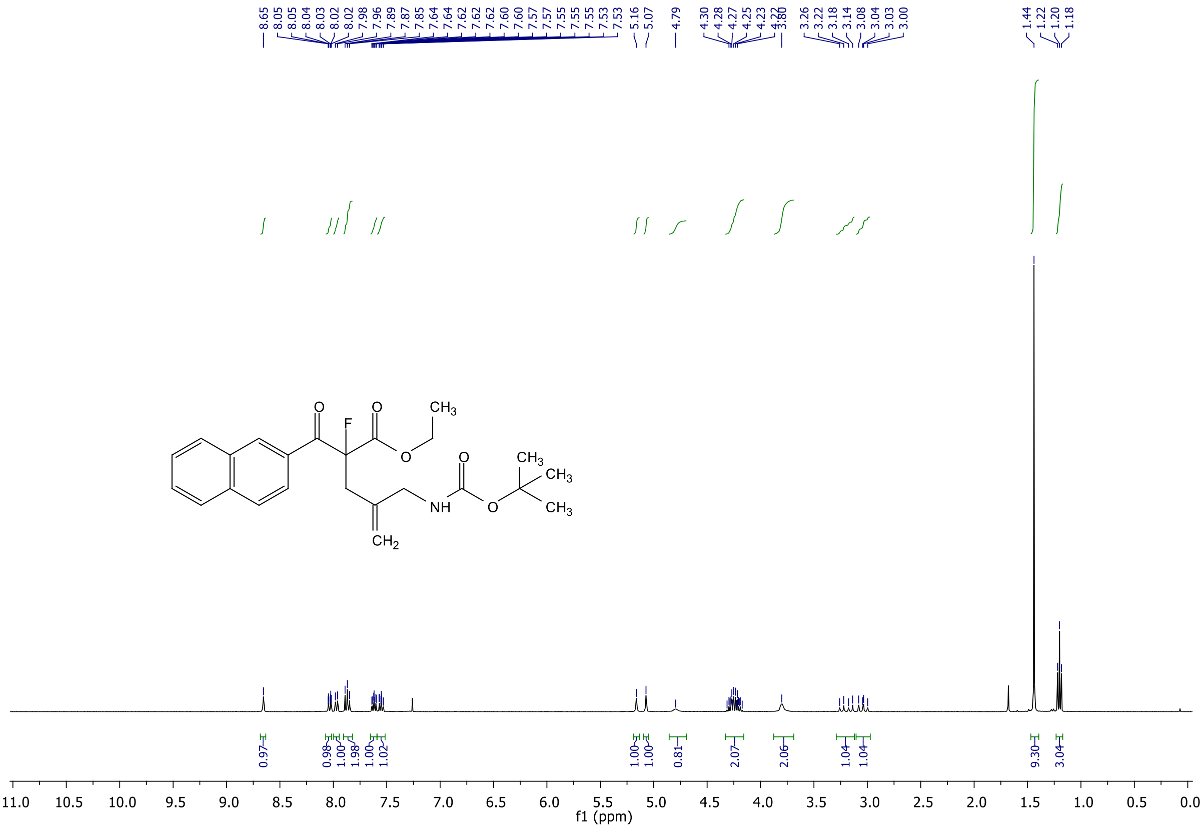

^{19}F NMR, CDCl$_3$, 377 MHz

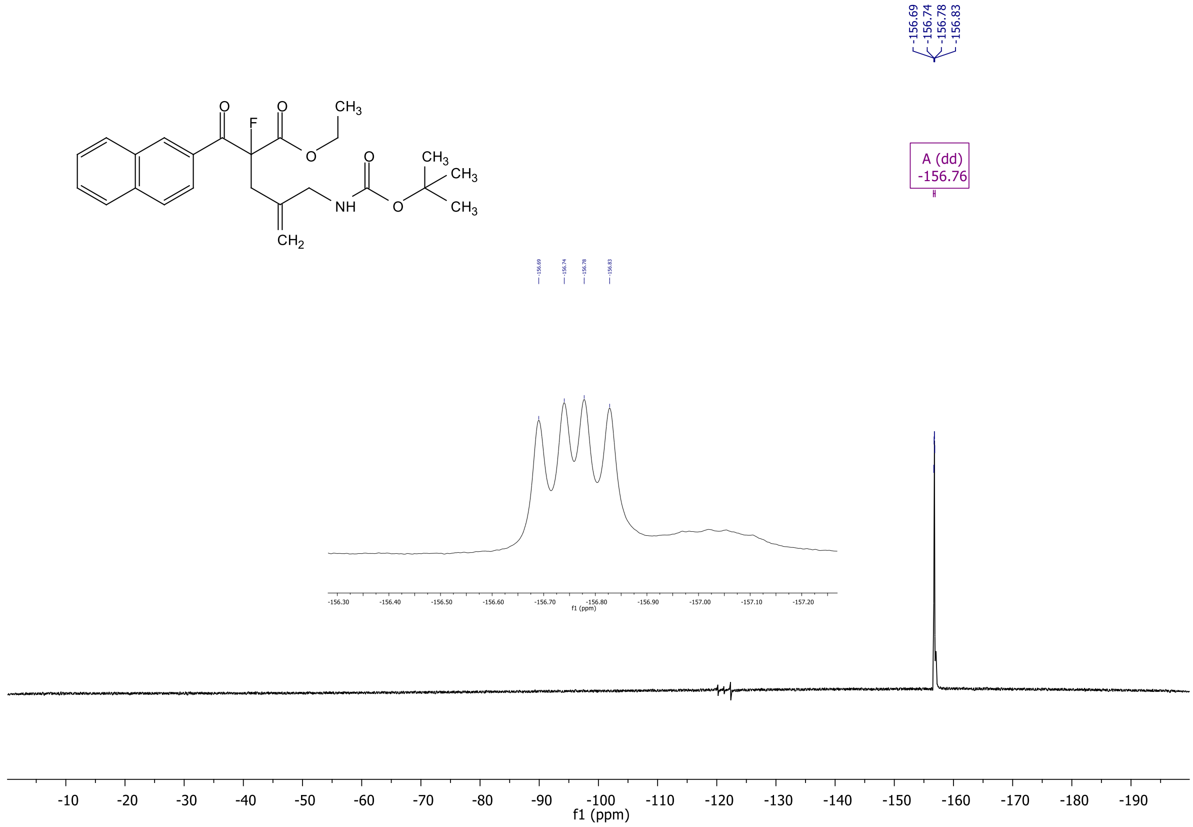

^{13}C NMR, $CDCl_3$, 101 MHz

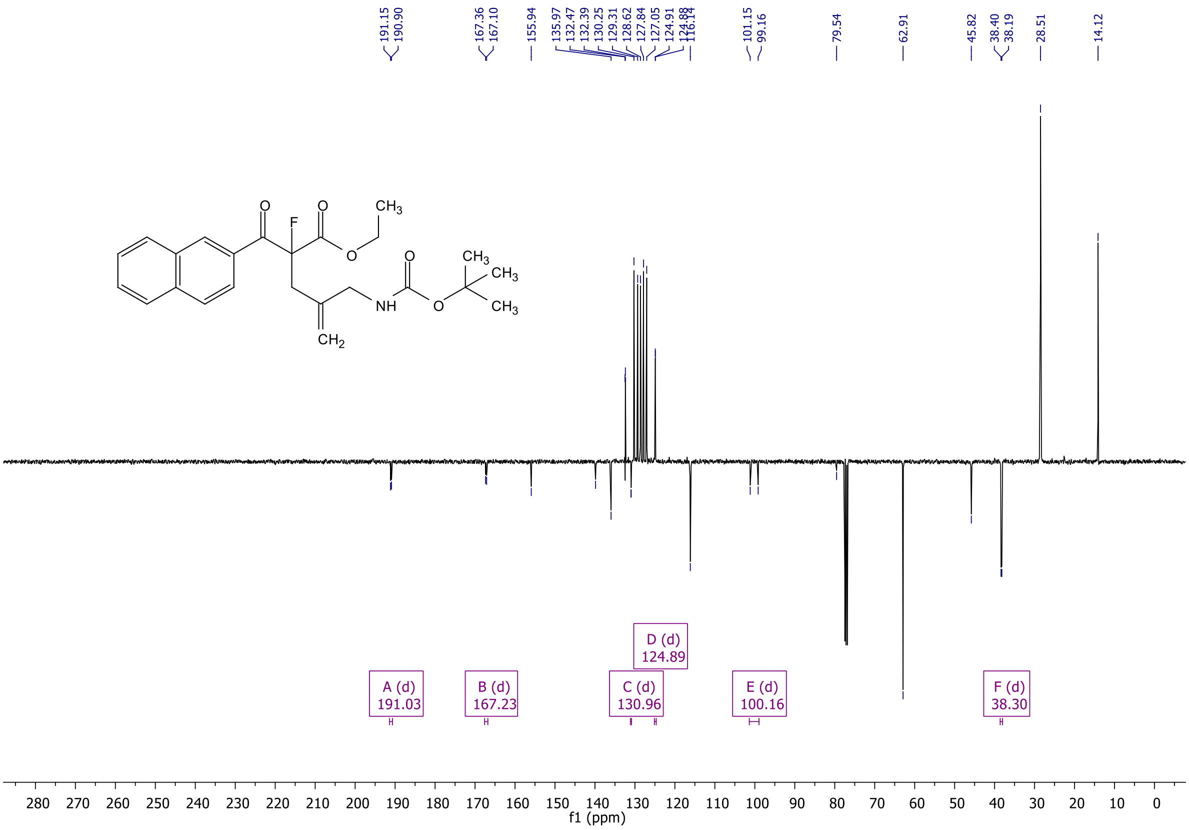

Ethyl 4-{[(*tert*-butoxycarbonyl)amino]methyl}-2-fluoro-2-(thiophene-2-carbonyl)pent-4-enoate (3i)

^{1}H NMR, $CDCl_3$, 400 MHz

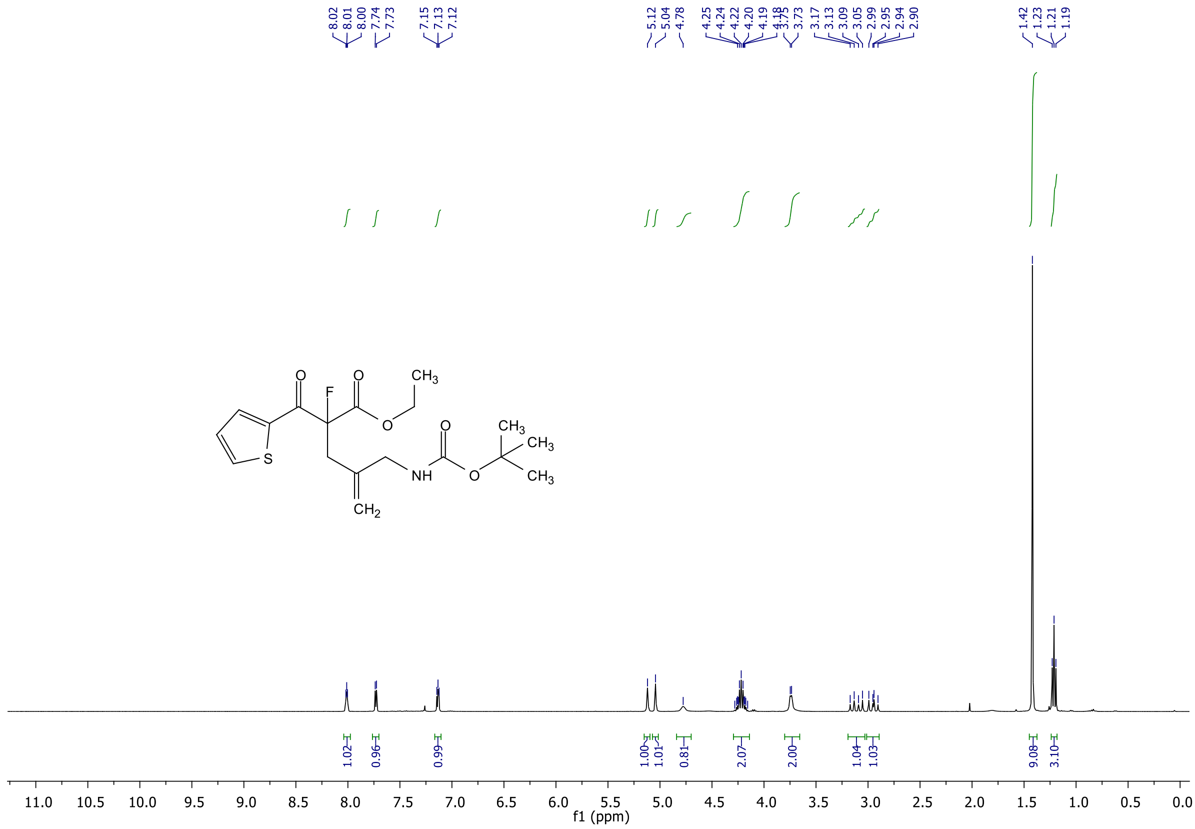

^{19}F NMR, $CDCl_3$, 377 MHz

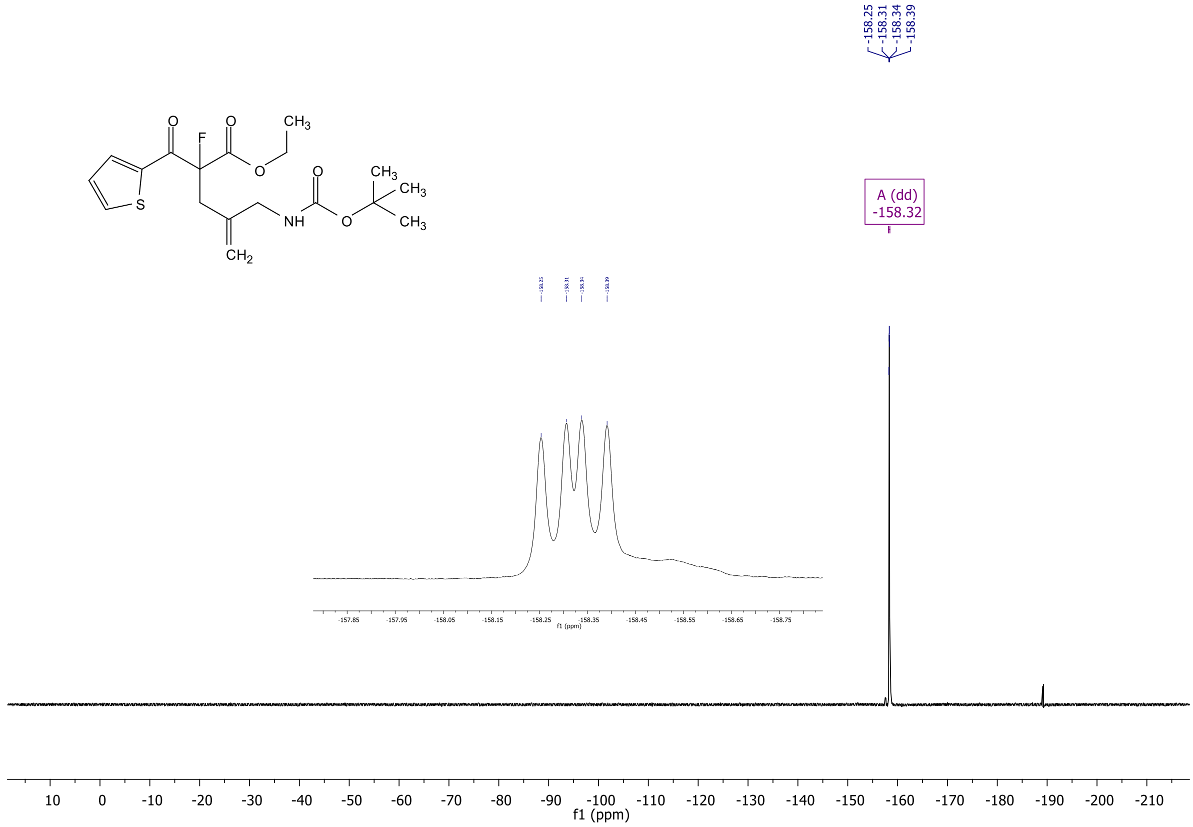

^{13}C NMR, $CDCl_3$, 101 MHz

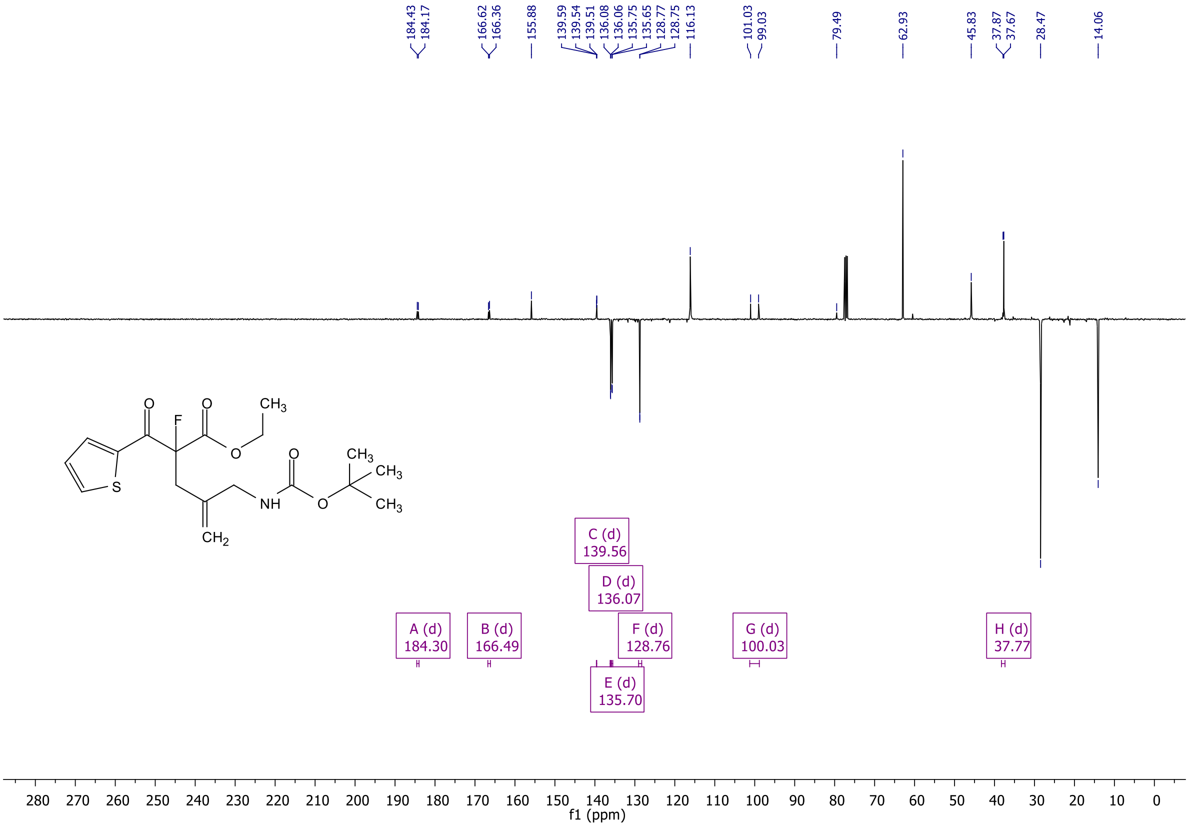

tert-Butyl (4-cyano-4-fluoro-2-methylene-5-oxo-5-phenylpentyl)carbamate (3j)

^1H NMR, $CDCl_3$, 400 MHz

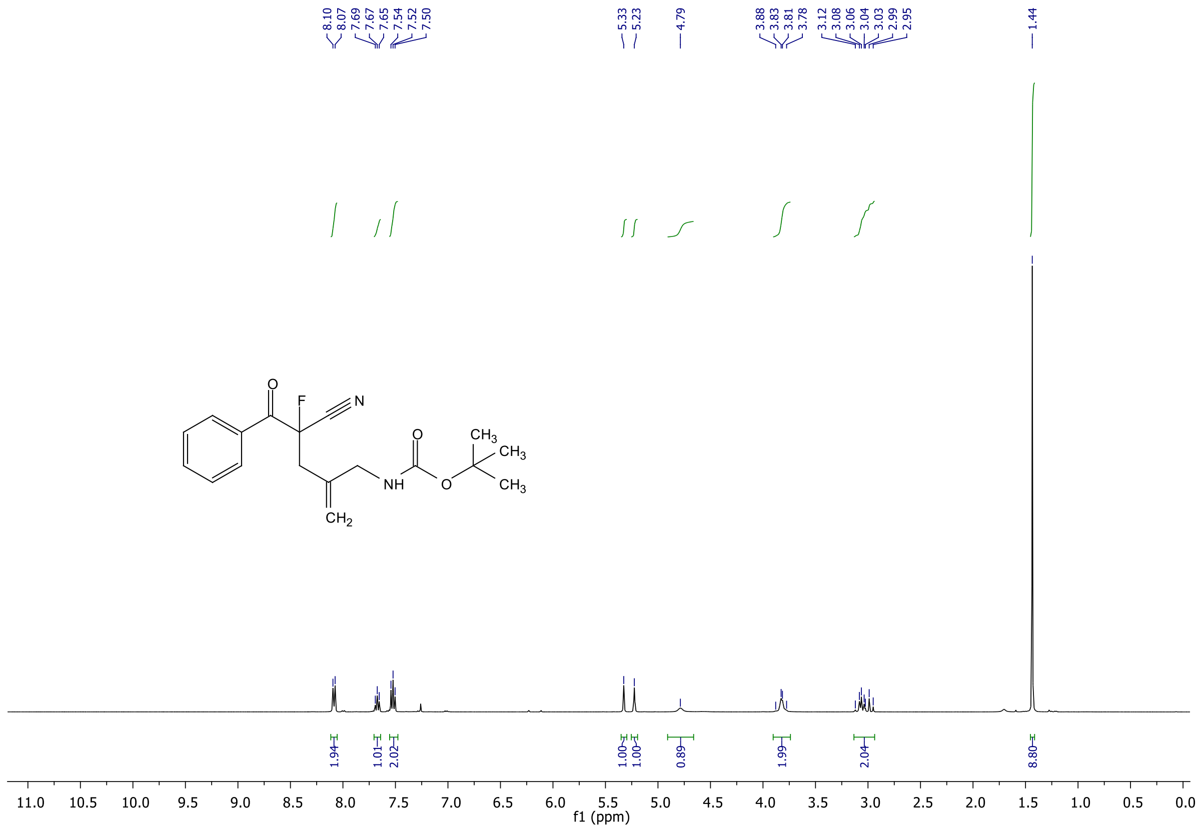

^{19}F NMR, $CDCl_3$, 377 MHz

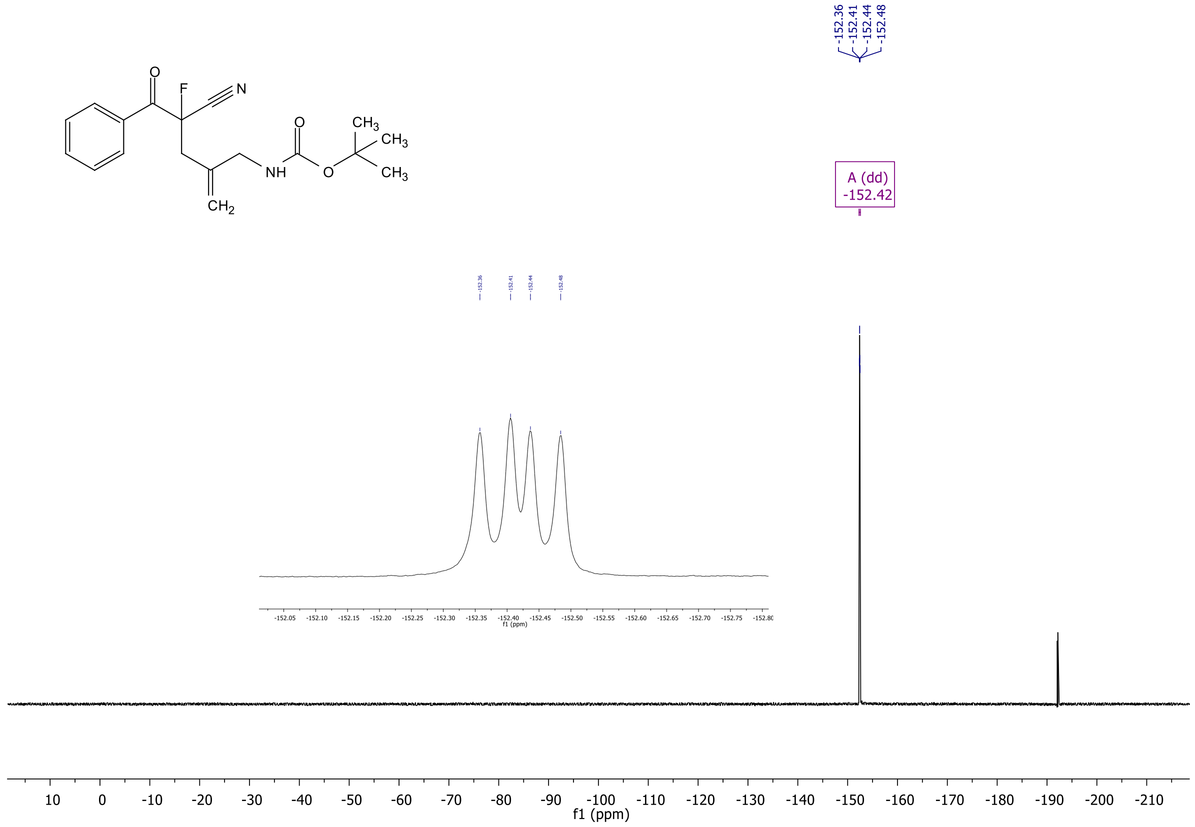

^{13}C NMR, $CDCl_3$, 101 MHz

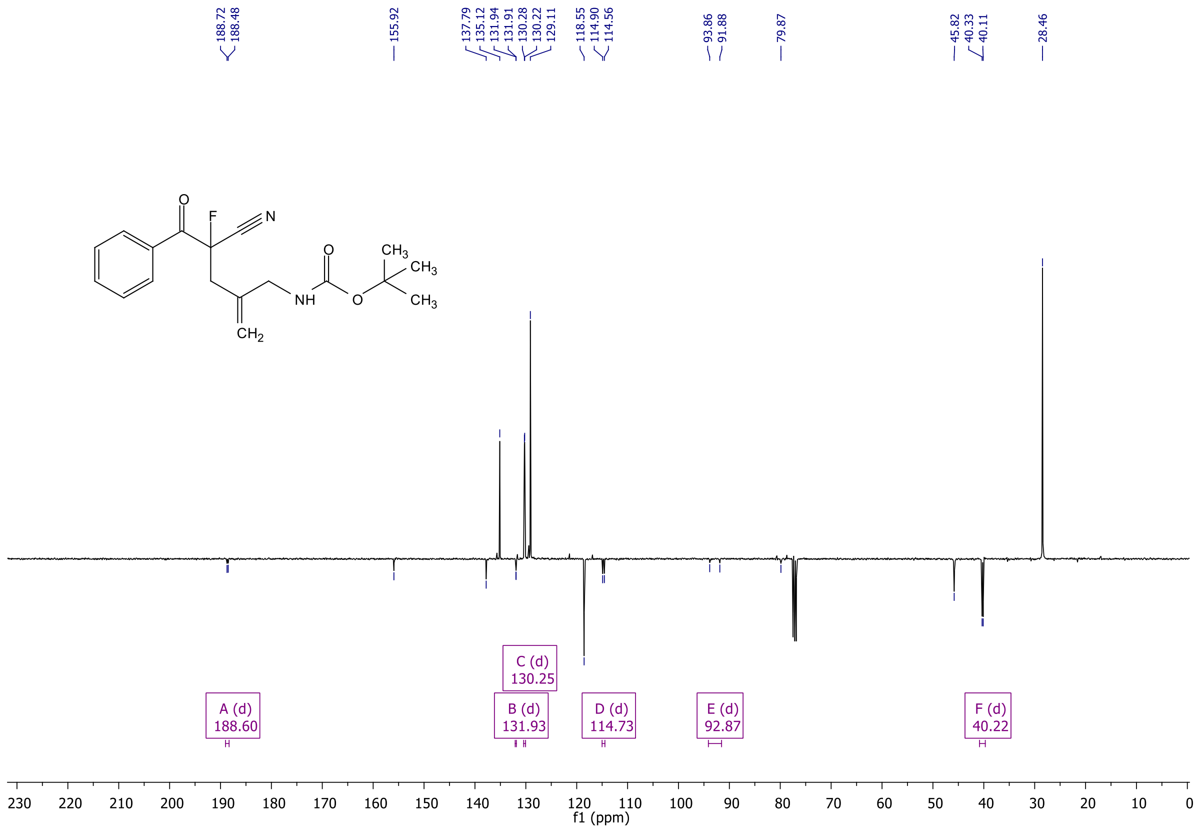

tert-Butyl (4-fluoro-2-methylene-5-oxo-5-phenyl-4-tosylpentyl)carbamate (3k)

^{1}H NMR, CDCl$_3$, 400 MHz

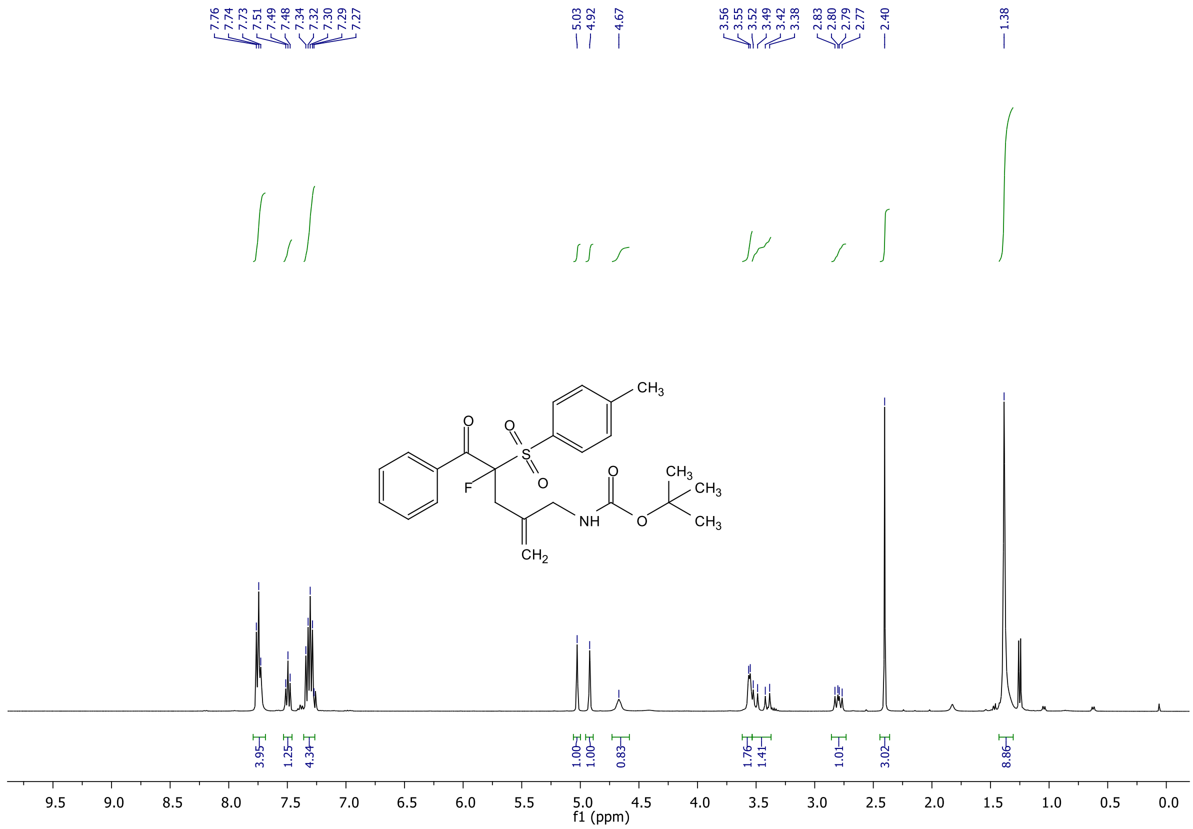

^{19}F NMR, CDCl$_3$, 377 MHz

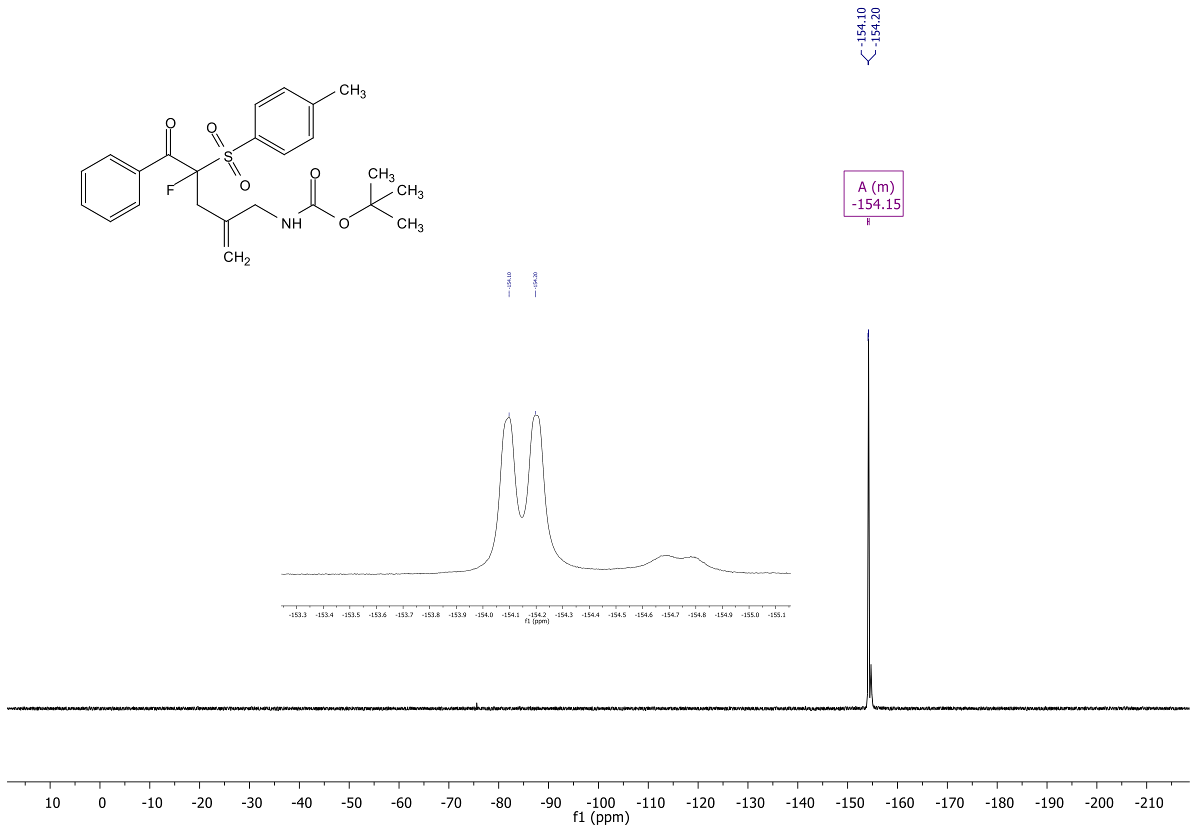

^{13}C NMR, $CDCl_3$, 101 MHz

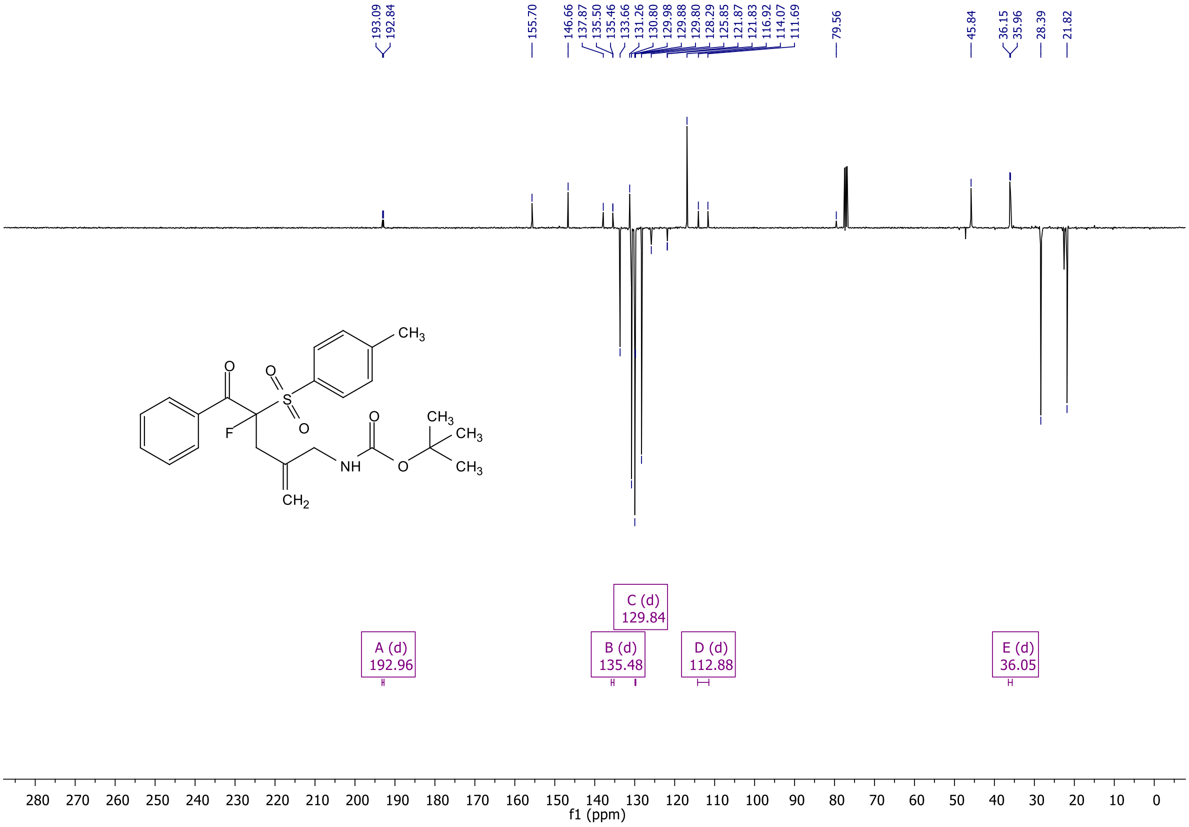

***tert*-Butyl (4-benzoyl-4-fluoro-5-(methoxy(methyl)amino)-2-methylene-5-oxopentyl)carbamate (3l)**
^{1}H NMR, CDCl$_3$, 400 MHz

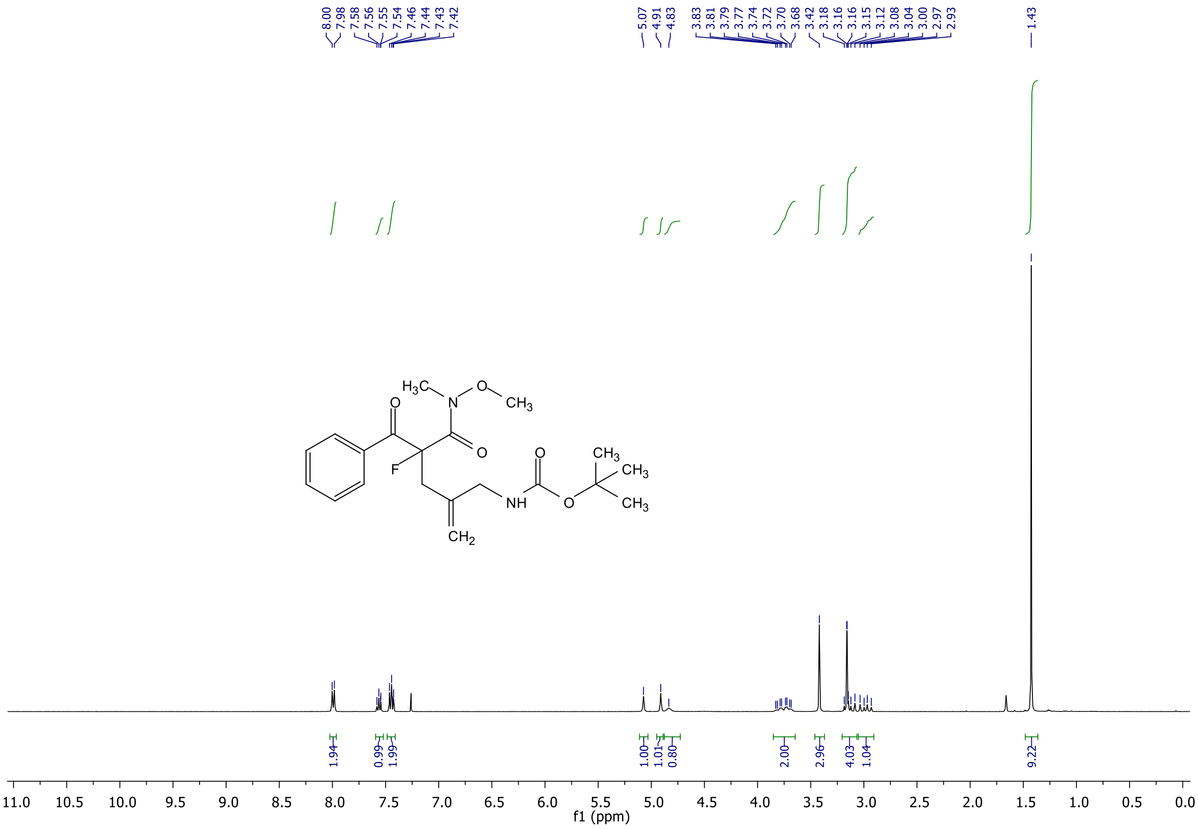

^{19}F NMR, CDCl$_3$, 377 MHz

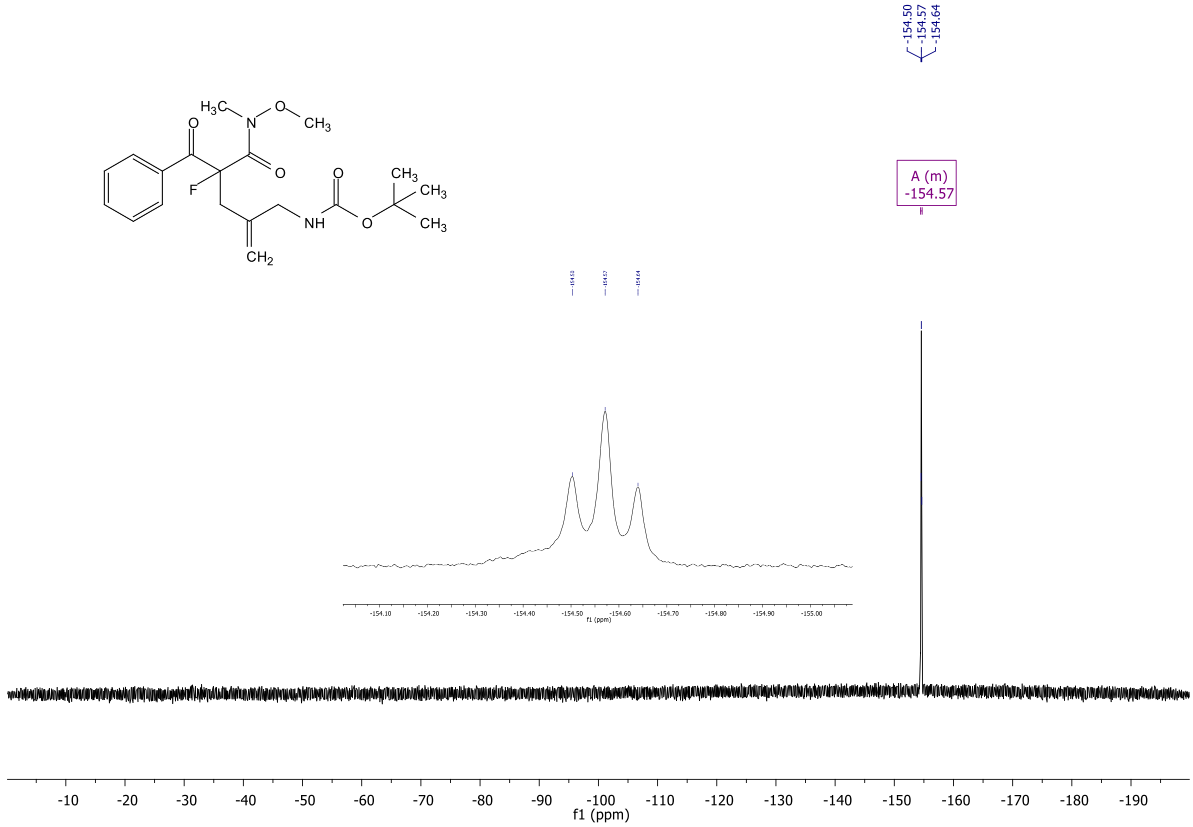

^{13}C NMR, $CDCl_3$, 101 MHz

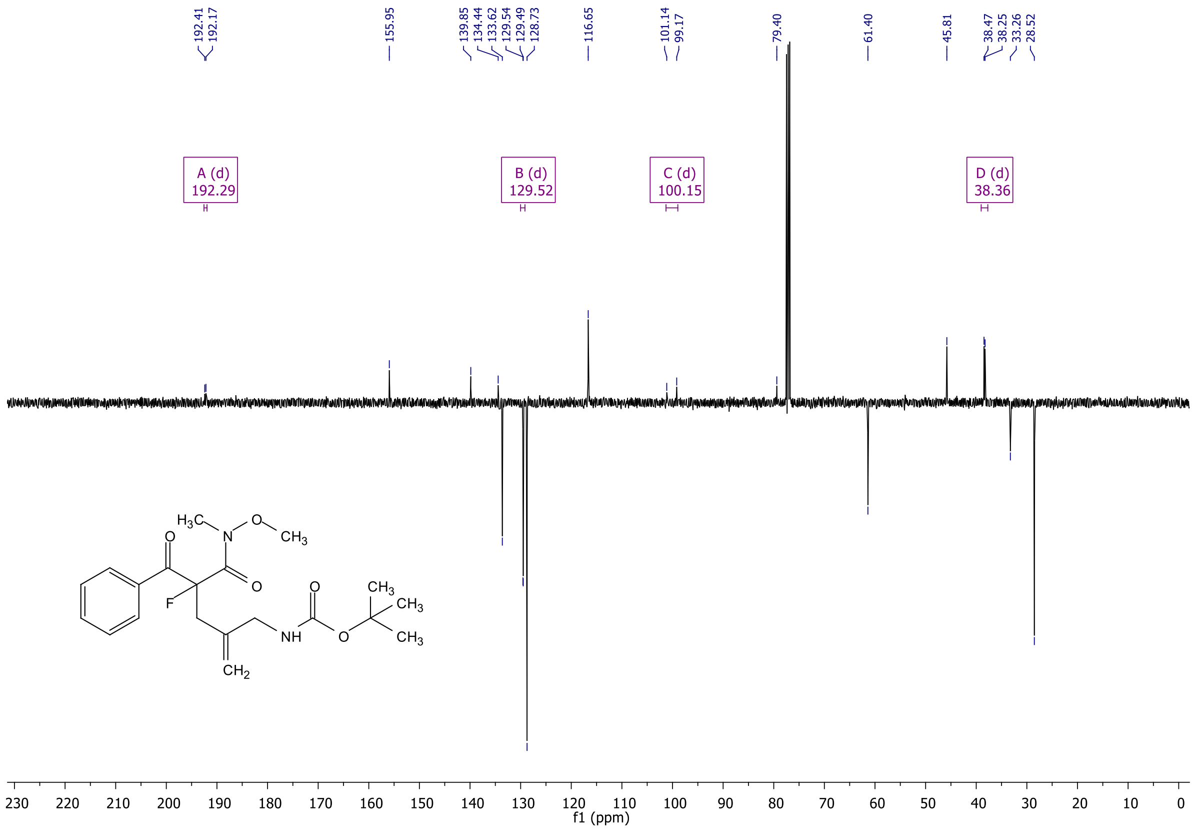

Ethyl 2-acetyl-4-{[(*tert*-butoxycarbonyl)amino]methyl}-2-fluoropent-4-enoate (3m)

^{1}H NMR, $CDCl_3$, 400 MHz

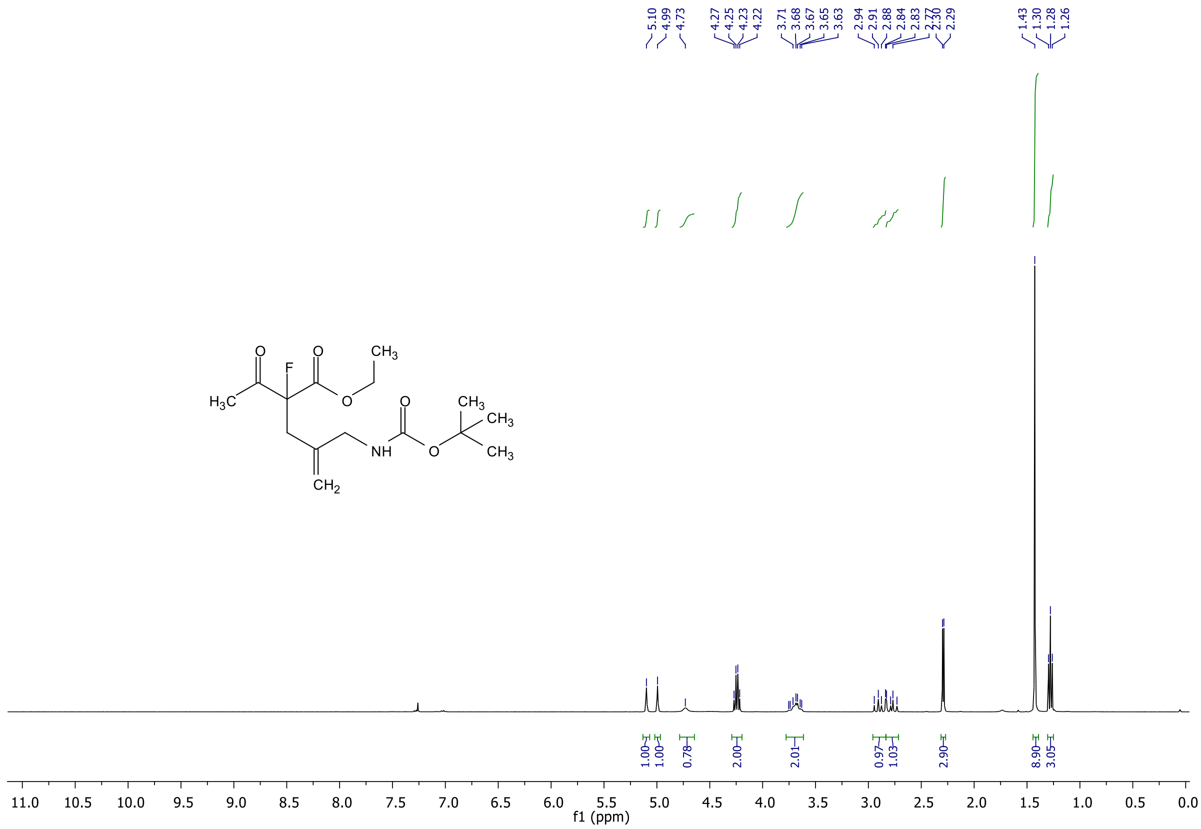

^{19}F NMR, $CDCl_3$, 377 MHz

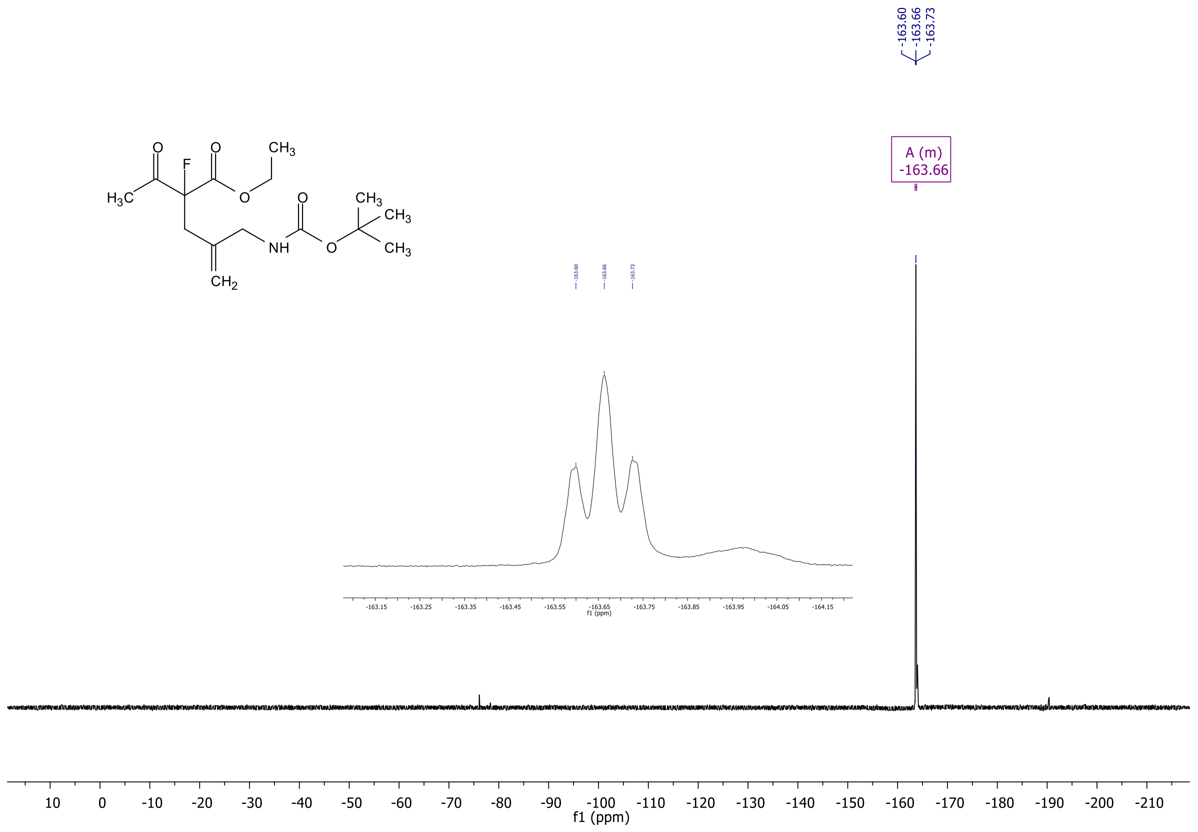

^{13}C NMR, $CDCl_3$, 101 MHz

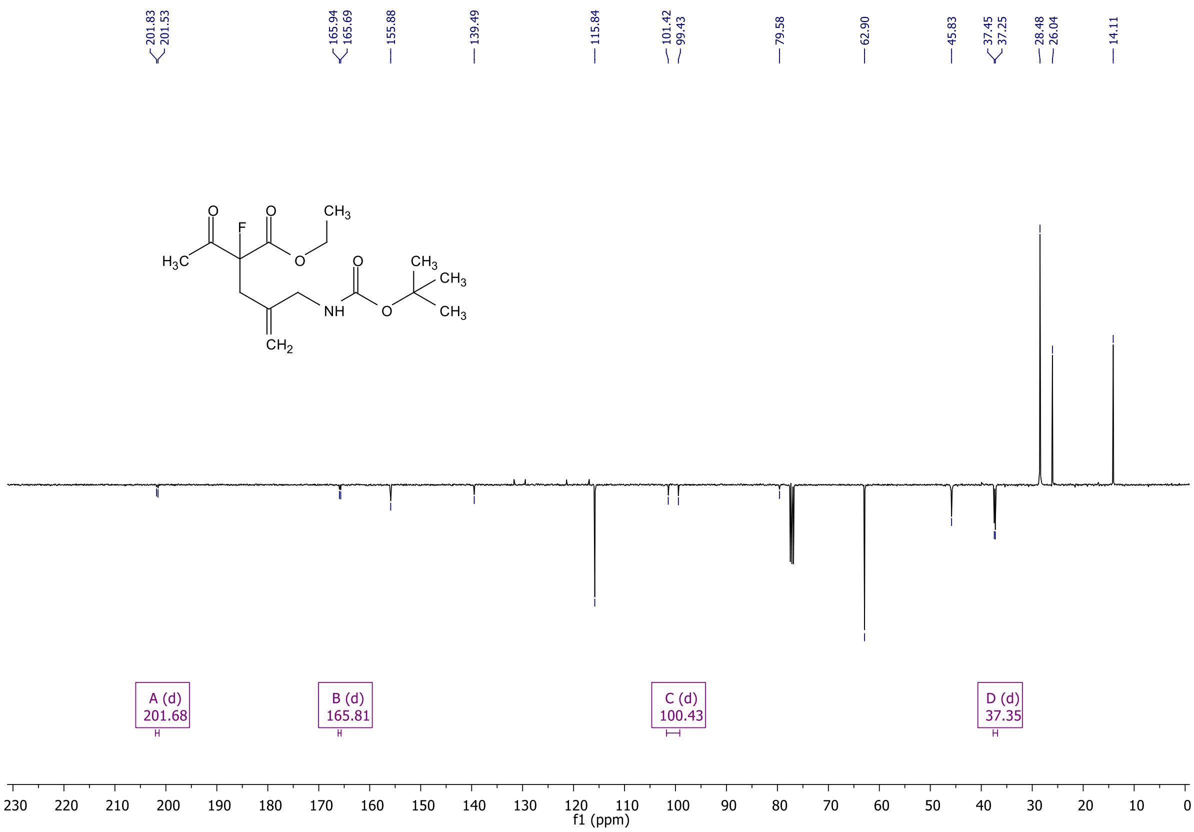

Ethyl 4-({[(*tert*-butoxy)carbonyl]amino}methyl)-2-fluoro-2-propanoylpent-4-enoate (3n)
^{1}H NMR, CDCl$_3$, 400 MHz

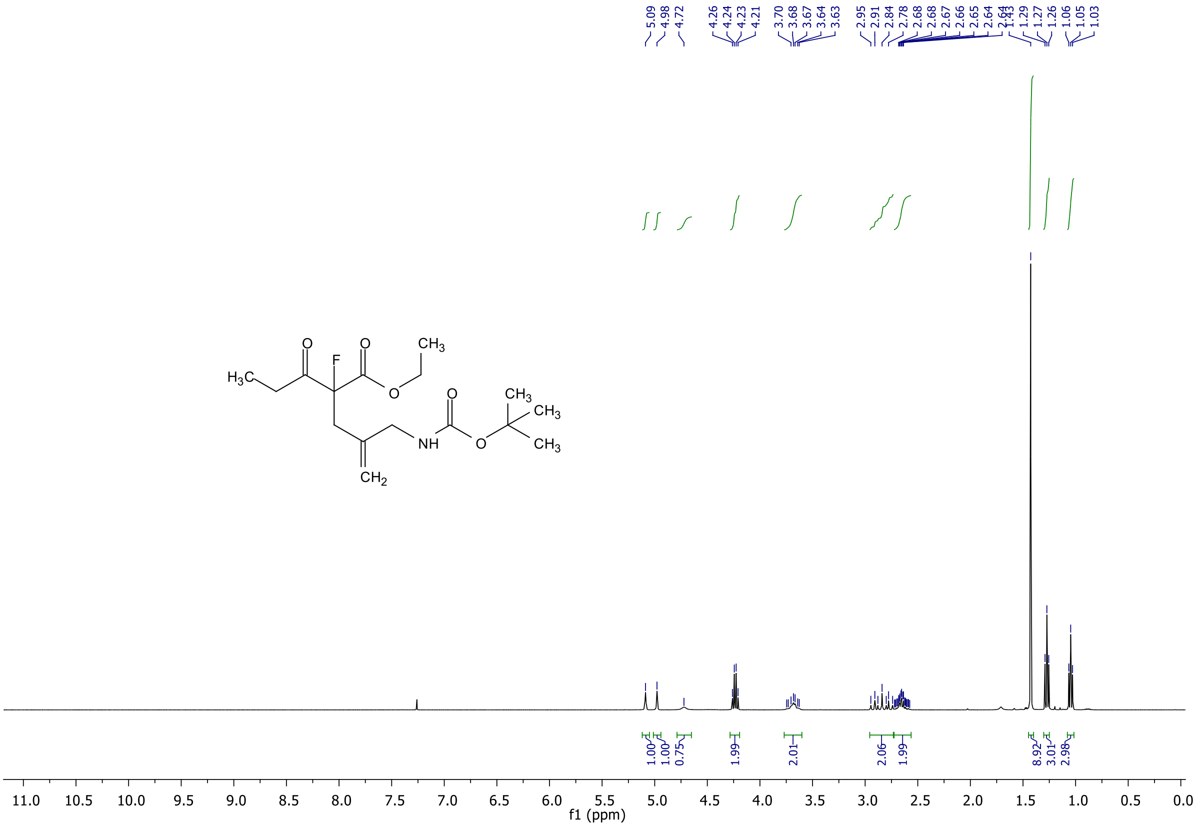

^{19}F NMR, CDCl$_3$, 377 MHz

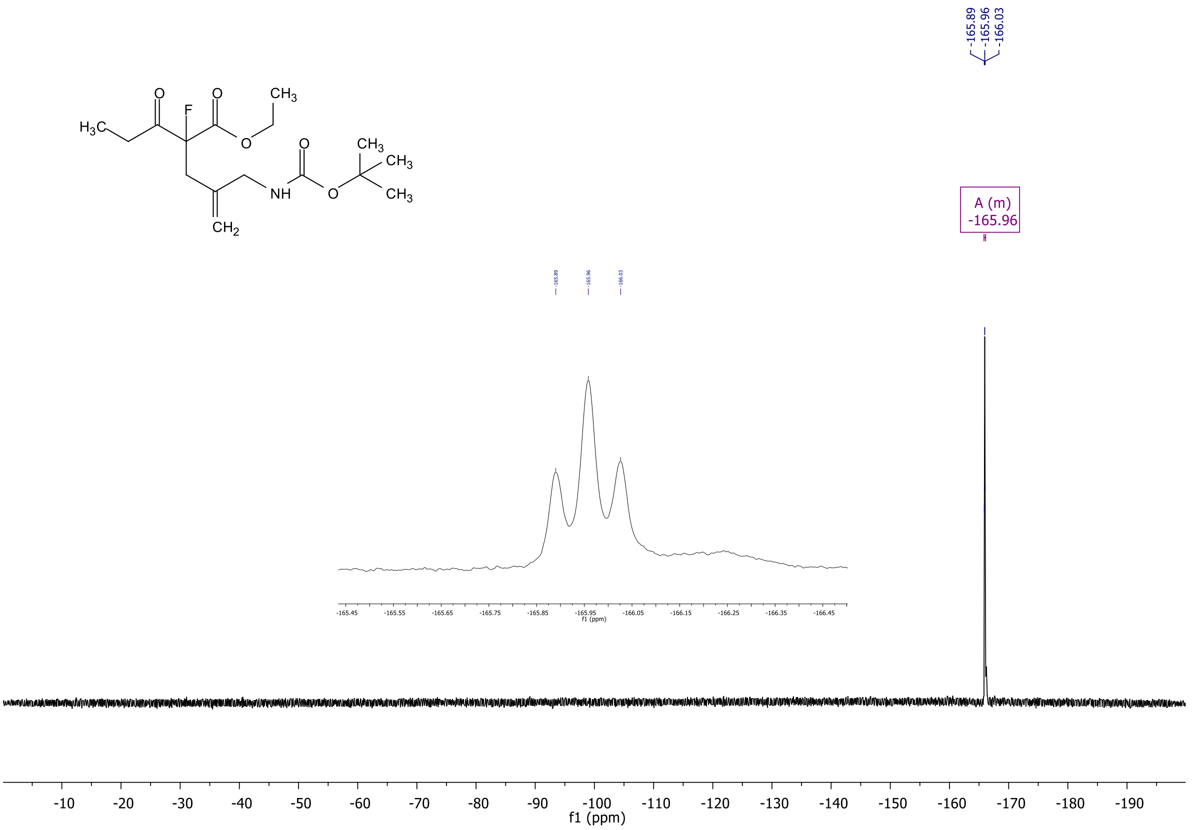

^{13}C NMR, $CDCl_3$, 101 MHz

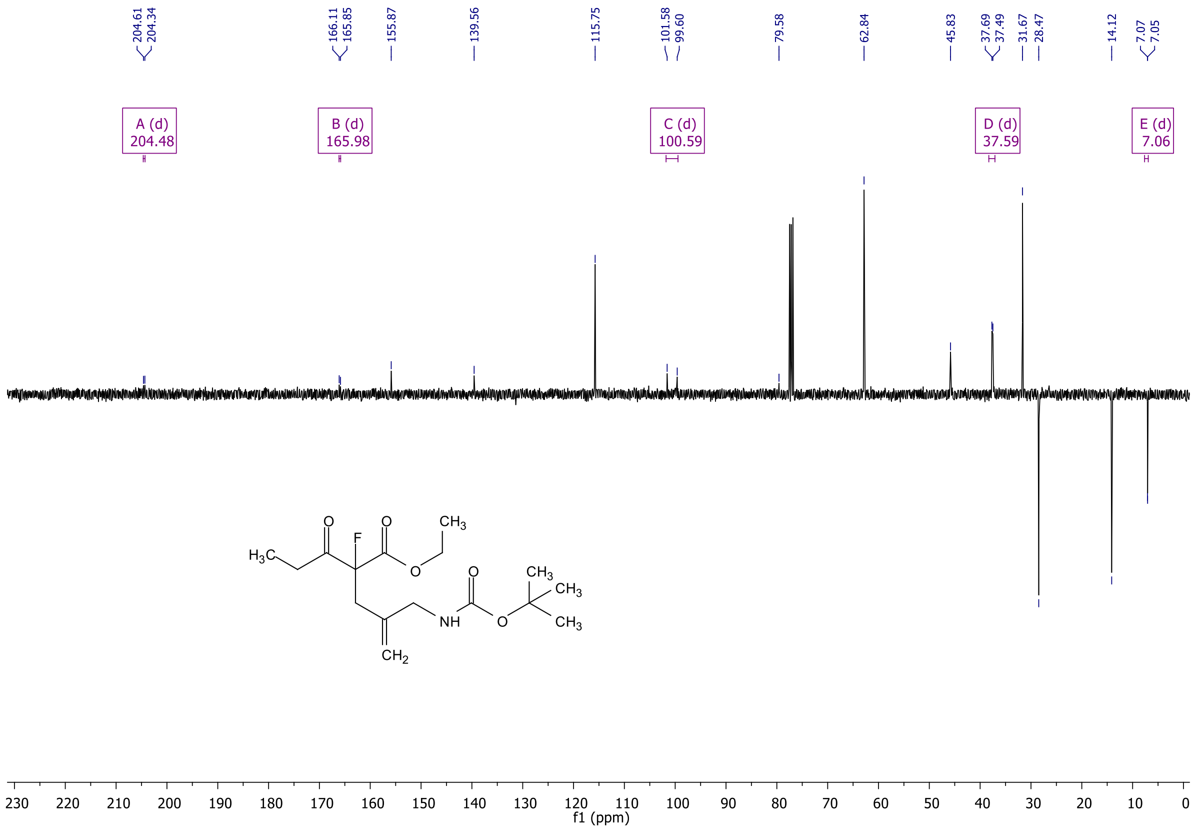

Ethyl 4-({[(*tert*-butoxy)carbonyl]amino}methyl)-2-fluoro-2-(2-methylpropanoyl)pent-4-enoate (3o)

^{1}H NMR, $CDCl_3$, 400 MHz

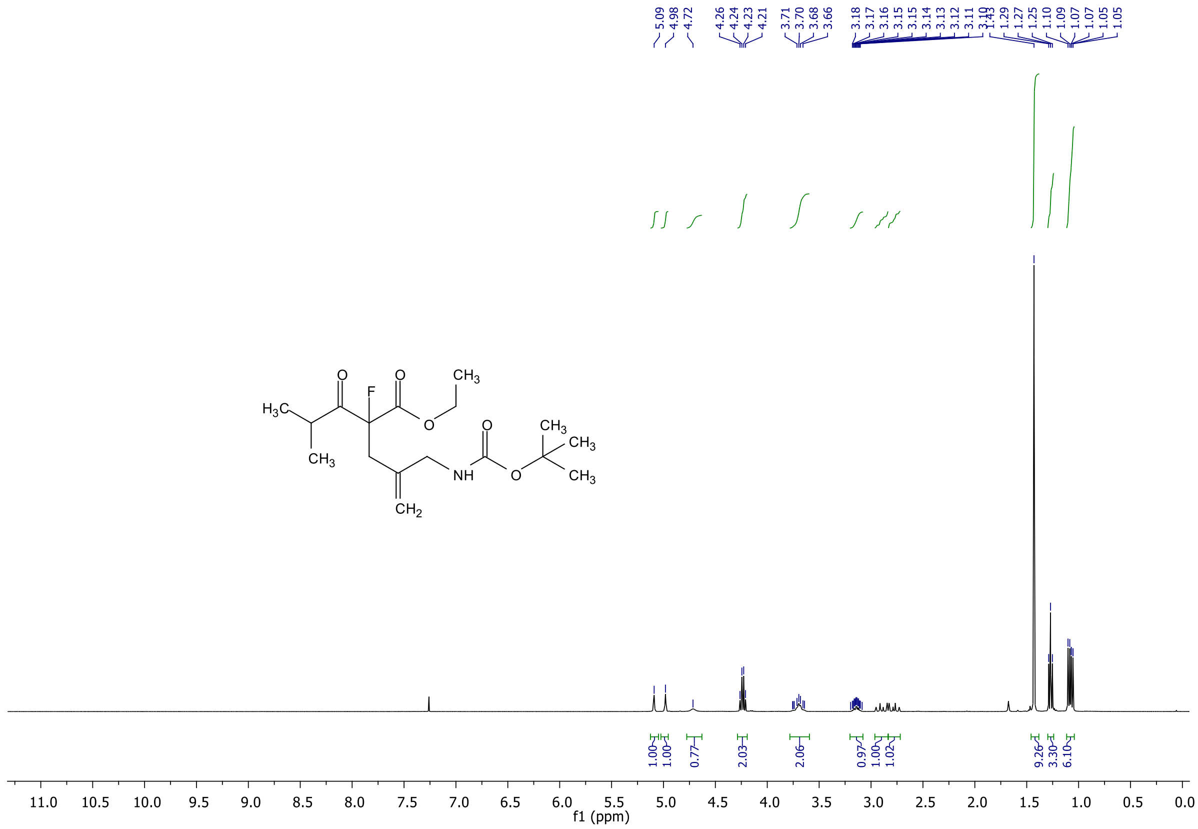

^{19}F NMR, $CDCl_3$, 377 MHz

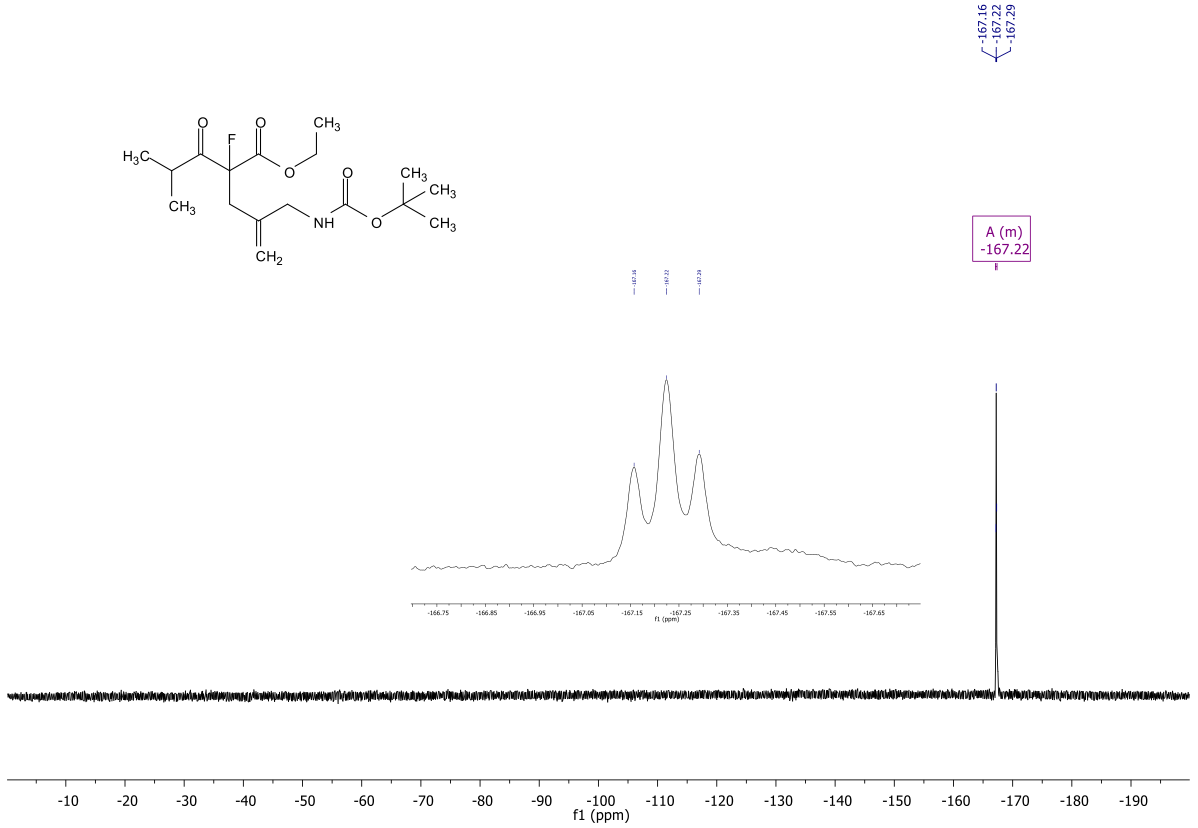

^{13}C NMR, $CDCl_3$, 101 MHz

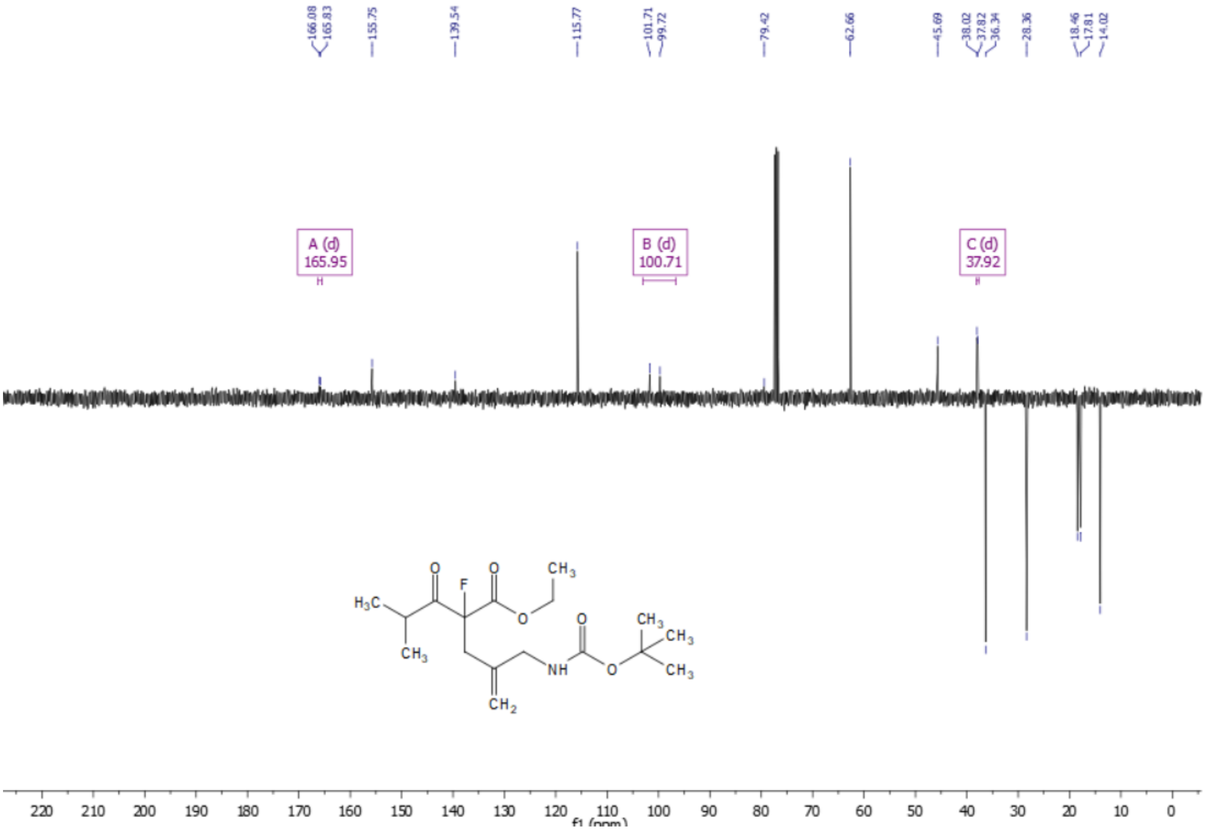

Ethyl 4-{[(*tert*-butoxycarbonyl)amino]methyl}-2-(2,2-dimethylpropanoyl)-2-fluoropent-4-enoate (3p)

^{1}H NMR, CDCl$_3$, 400 MHz

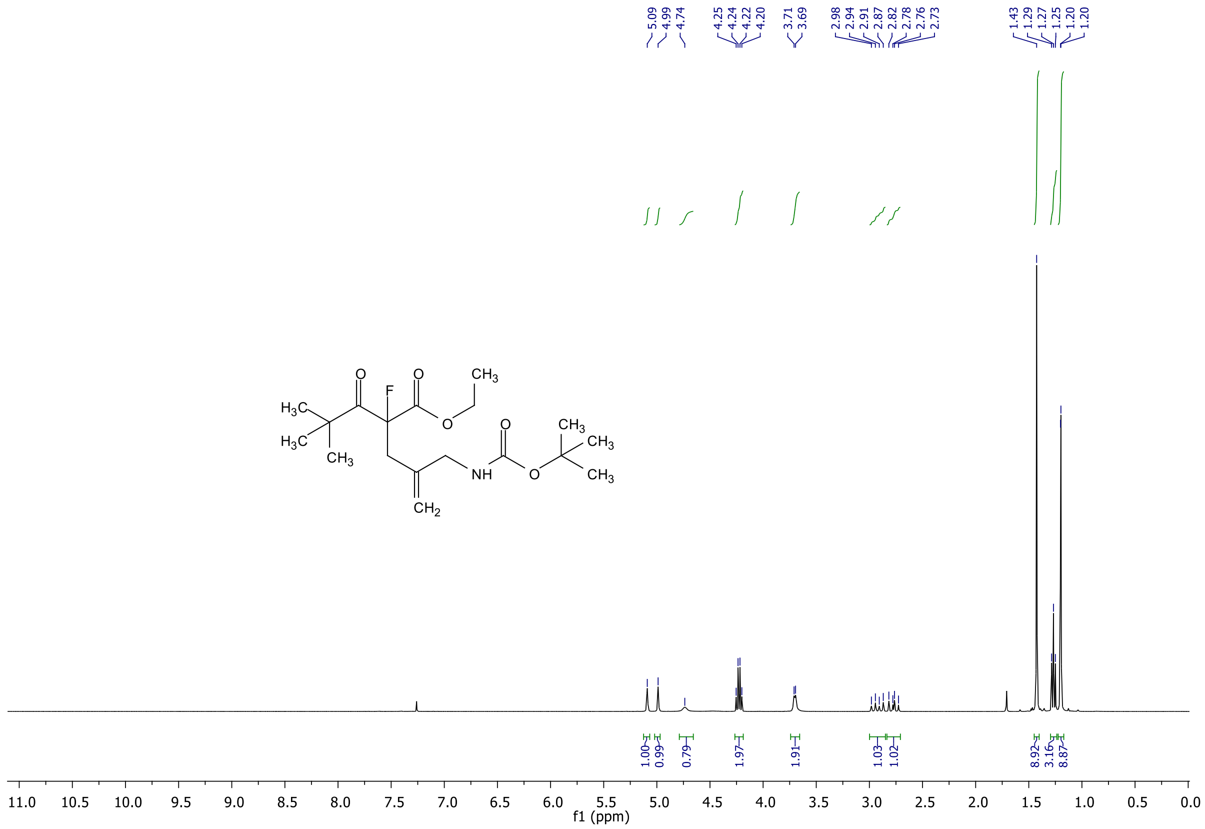

^{19}F NMR, CDCl$_3$, 377 MHz

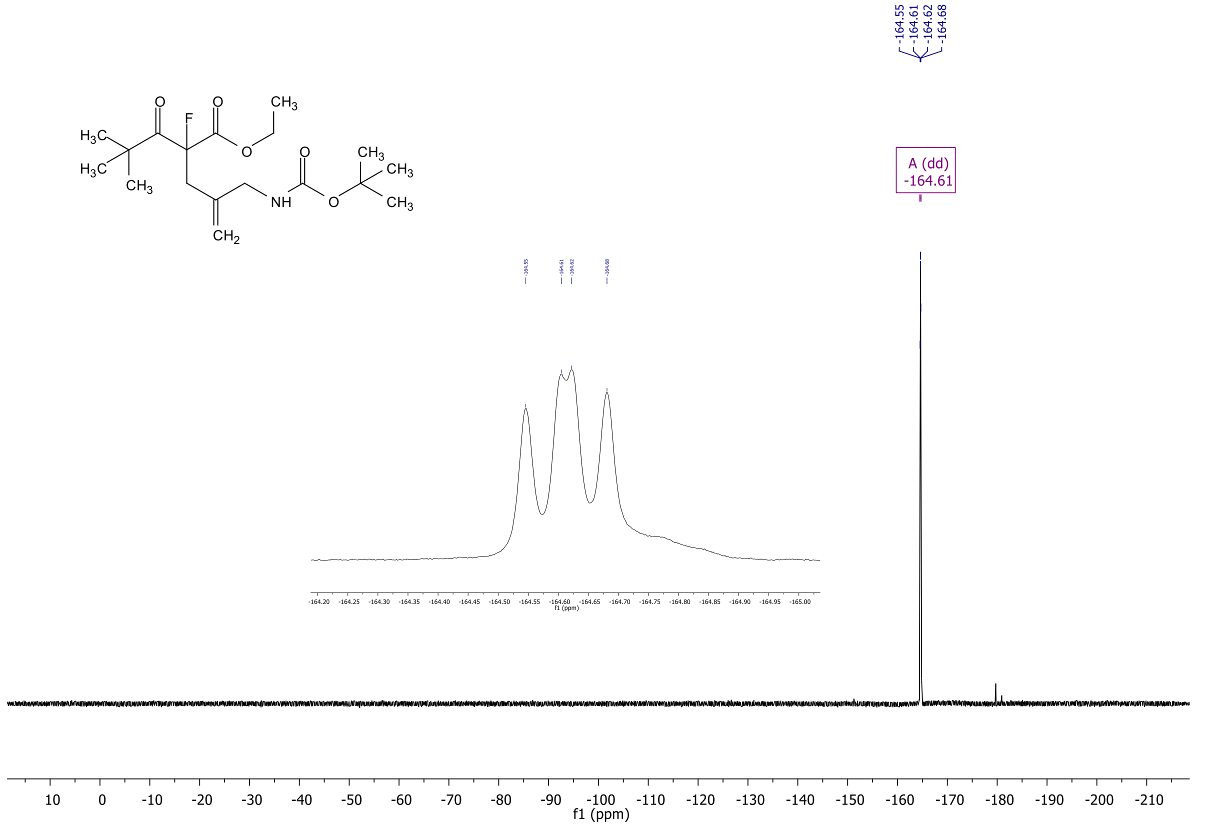

^{13}C NMR, $CDCl_3$, 101 MHz

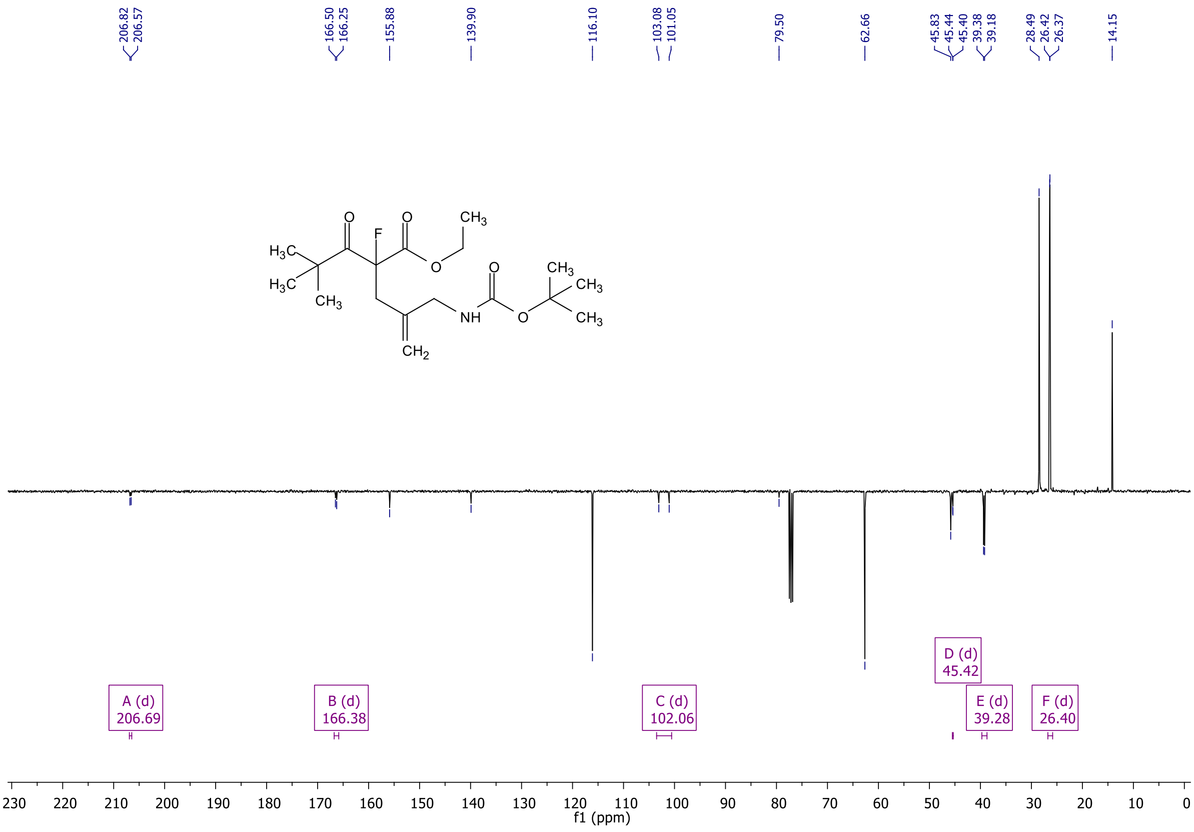

Ethyl 4-({[(*tert*-butoxy)carbonyl]amino}methyl)-2-cyclohexanecarbonyl-2-fluoropent-4-enoate (3q)

^{1}H NMR, $CDCl_3$, 400 MHz

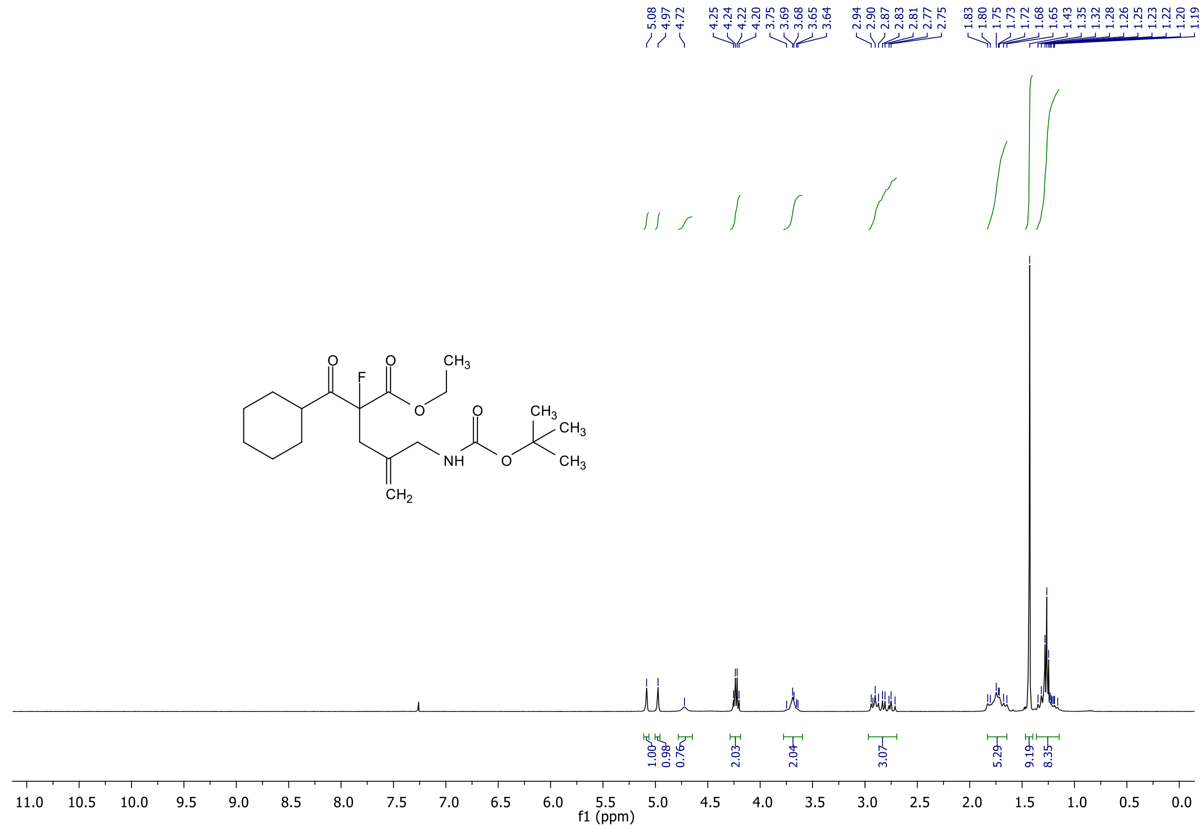

^{19}F NMR, $CDCl_3$, 377 MHz

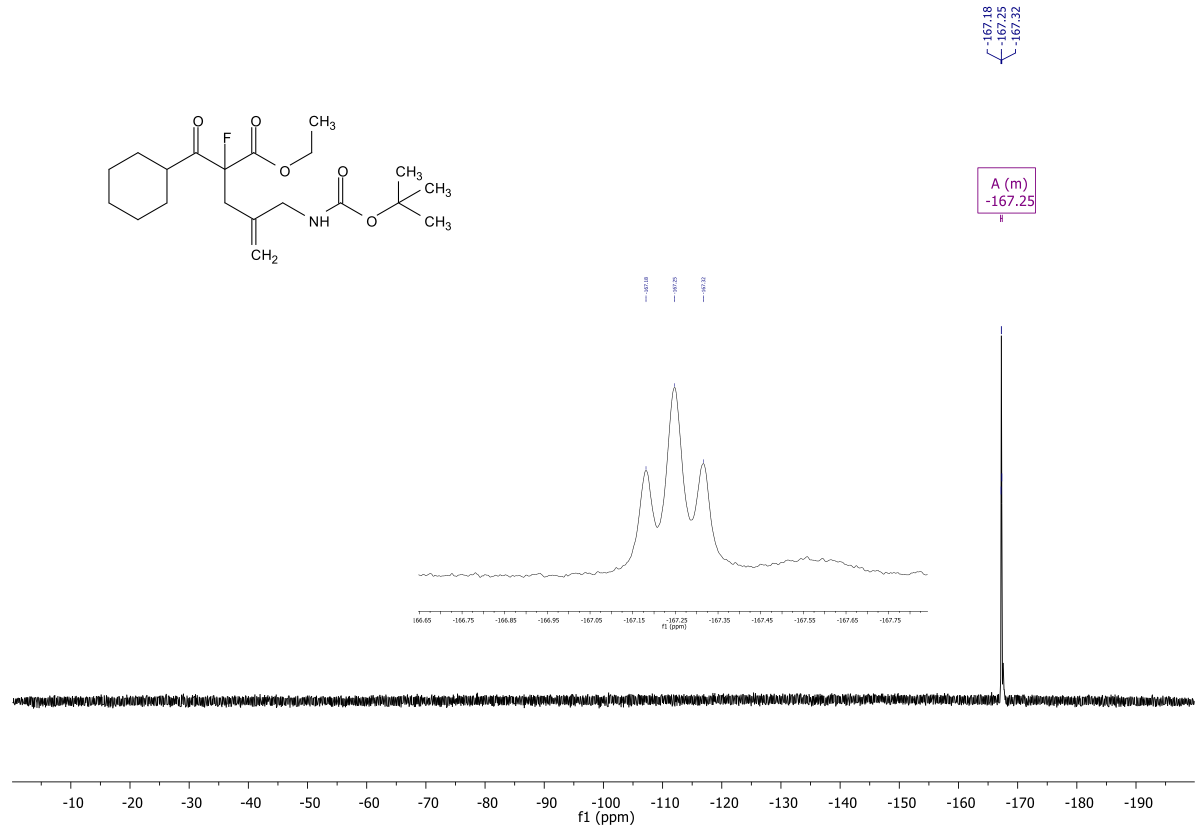

^{13}C NMR, CDCl$_3$, 101 MHz

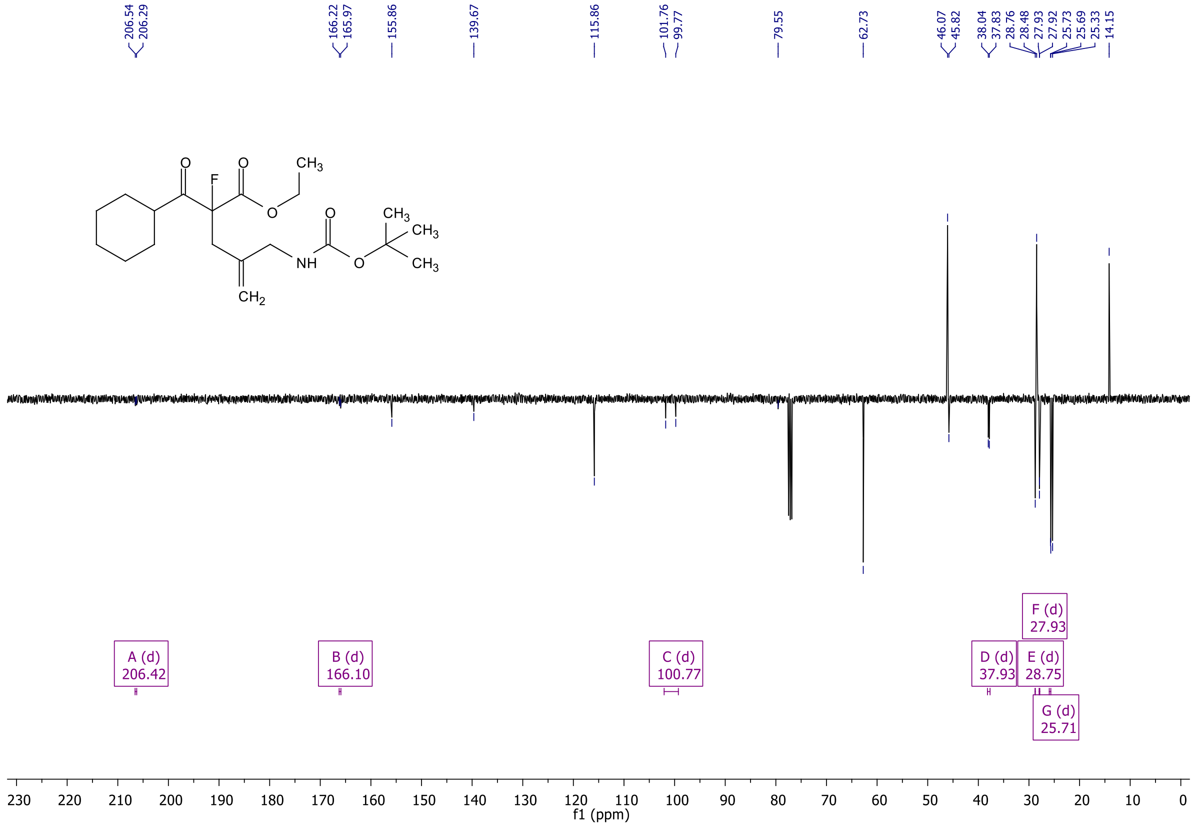

Ethyl 2-(2-(((*tert*-butoxycarbonyl)amino)methyl)allyl)-2-fluoro-3-oxooct-7-enoate (3r)

^{1}H NMR, $CDCl_3$, 400 MHz

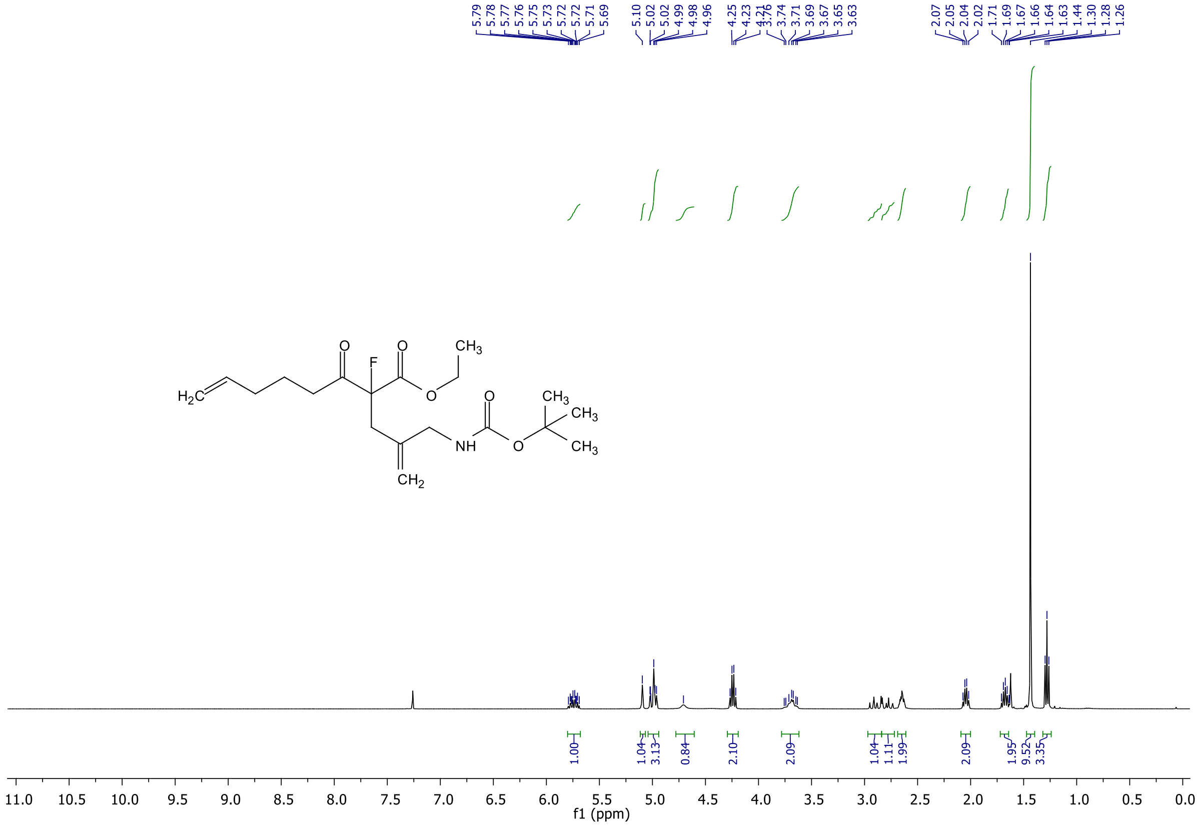

^{19}F NMR, $CDCl_3$, 377 MHz

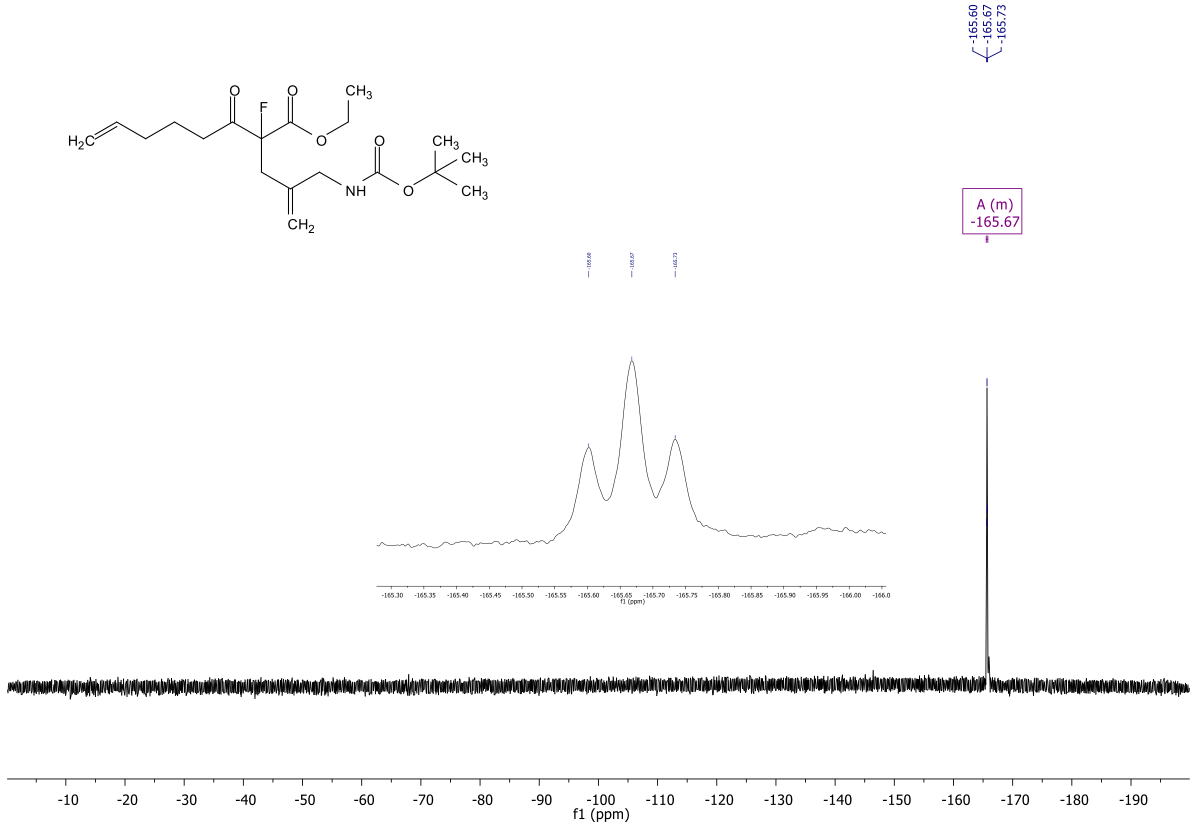

^{13}C NMR, $CDCl_3$, 101 MHz

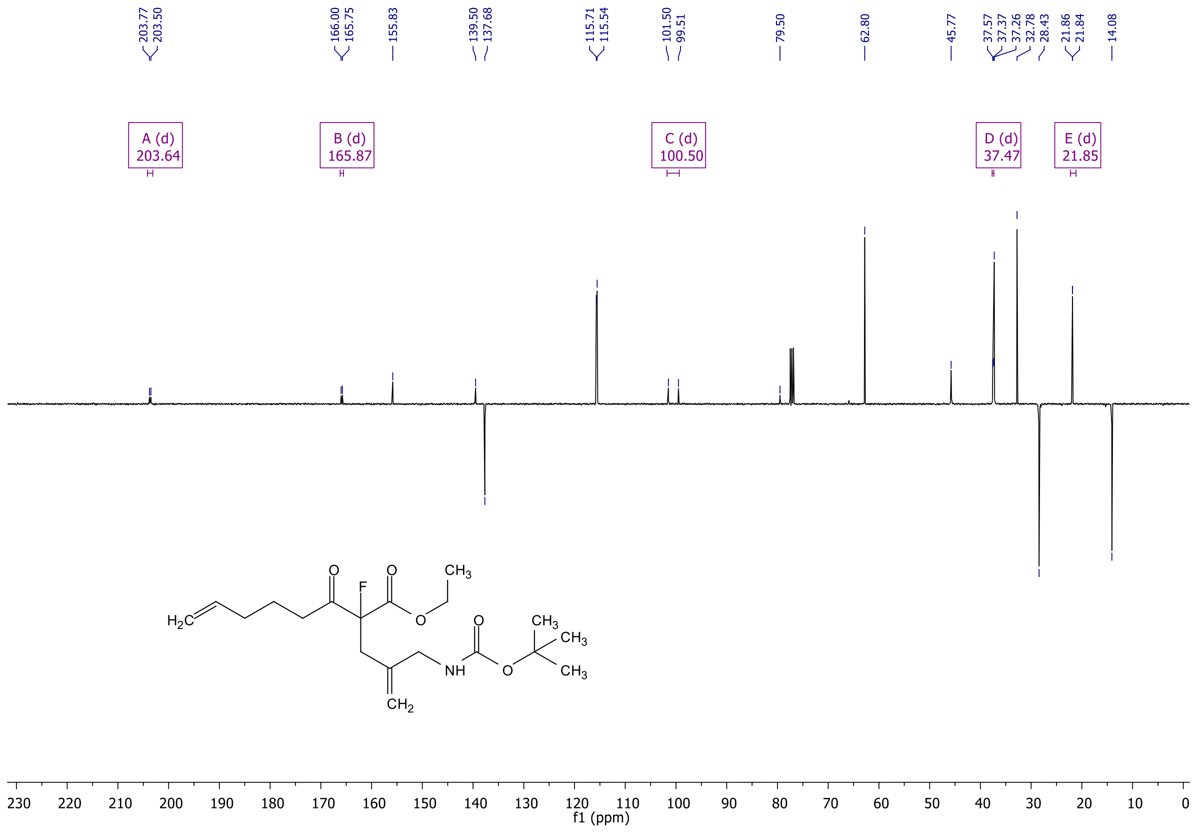

Ethyl 4-(((*tert*-butoxycarbonyl)amino)methyl)-2-fluoro-2-(1-tosylpyrrolidine-2-carbonyl)pent-4-enoate (3s)

^{1}H NMR, $CDCl_3$, 400 MHz

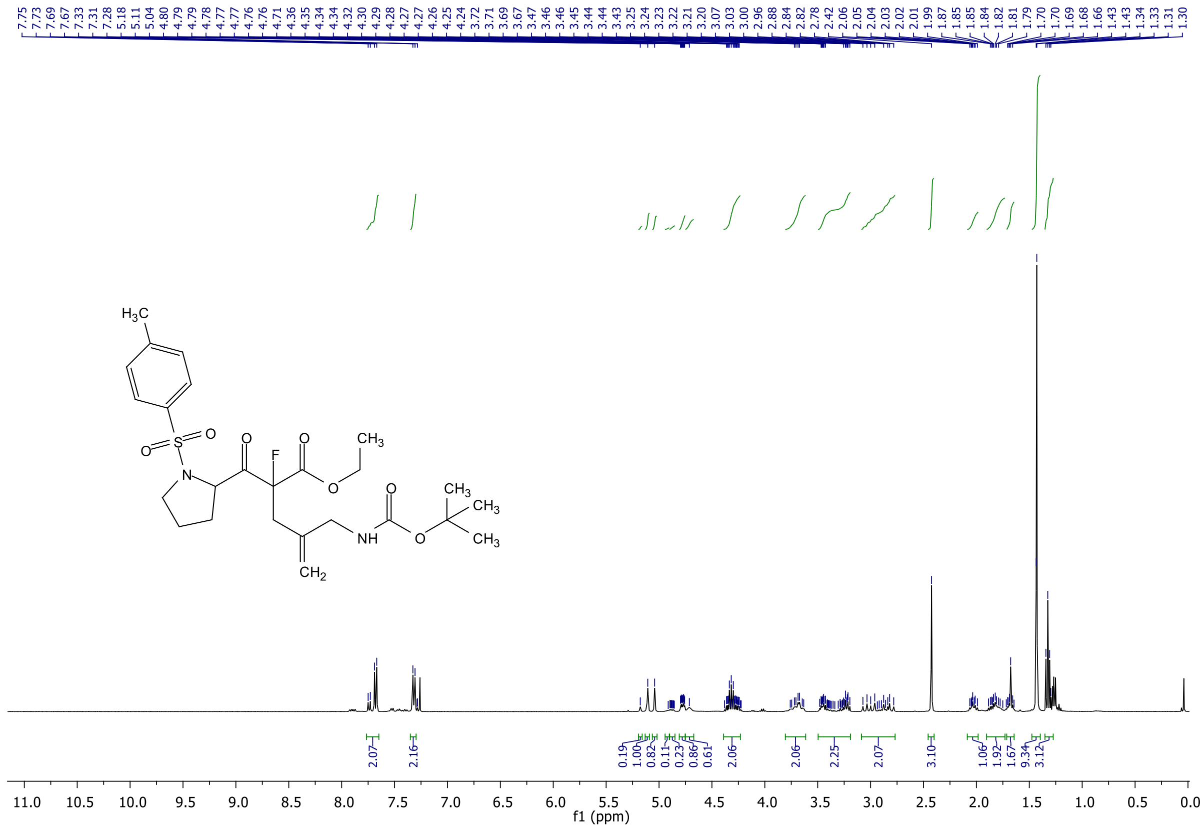

^{19}F NMR, $CDCl_3$, 377 MHz

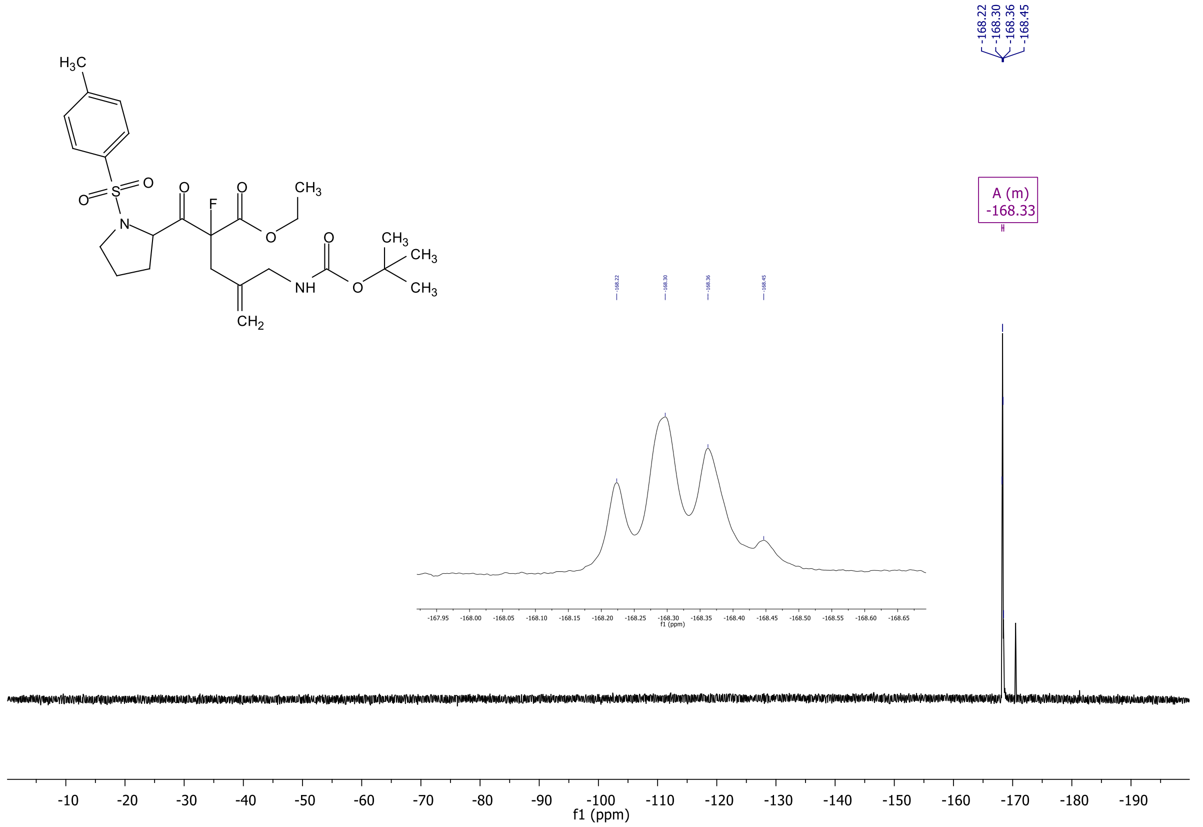

^{13}C NMR, $CDCl_3$, 101 MHz

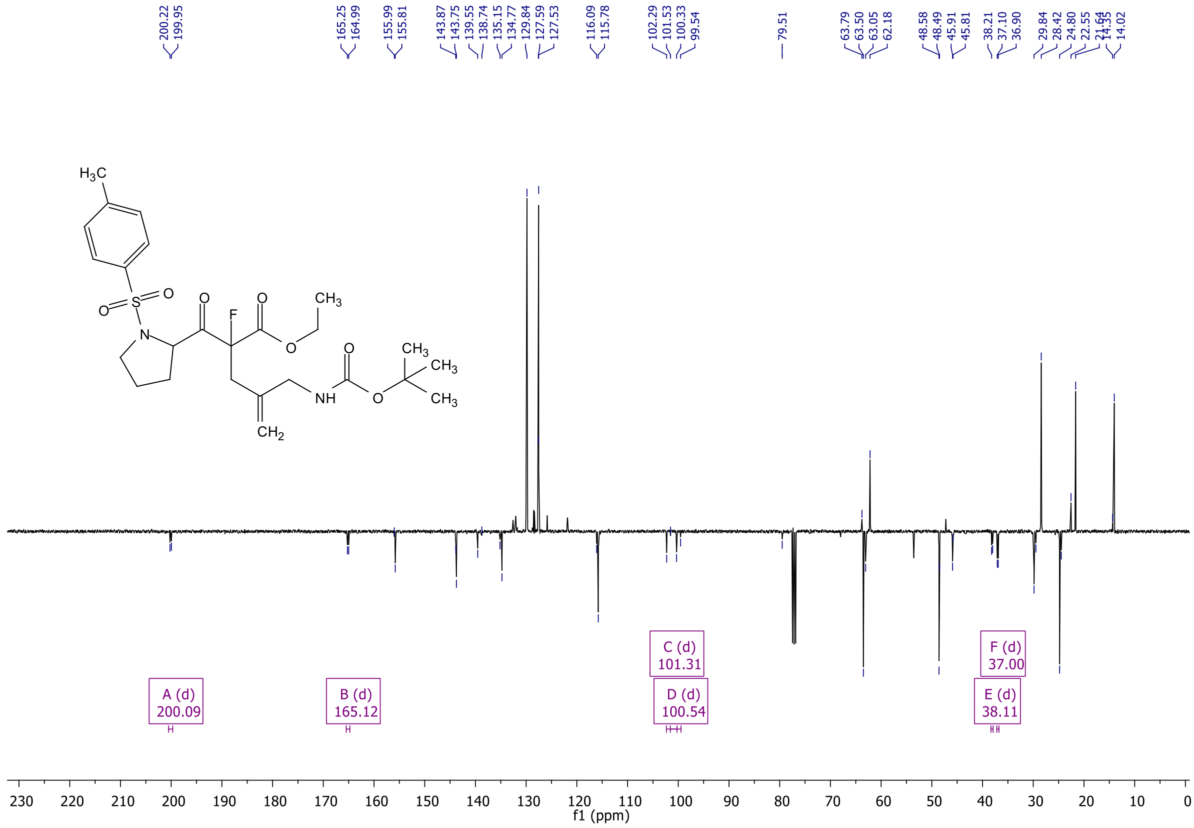

Ethyl 3-fluoro-5-methylene-2-(phenyl)-3,4,5,6-tetrahydropyridine-3-carboxylate (4a)

^{1}H NMR, $CDCl_3$, 400 MHz

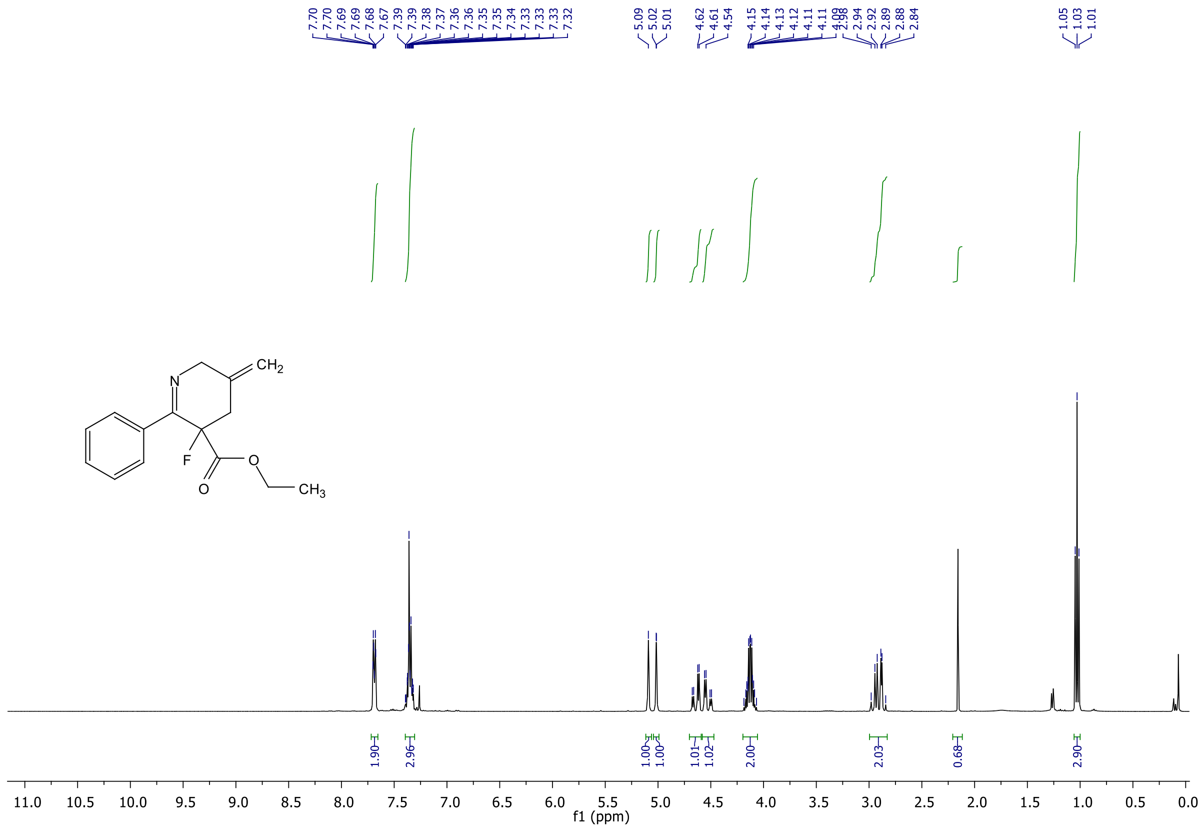

^{19}F NMR, $CDCl_3$, 377 MHz

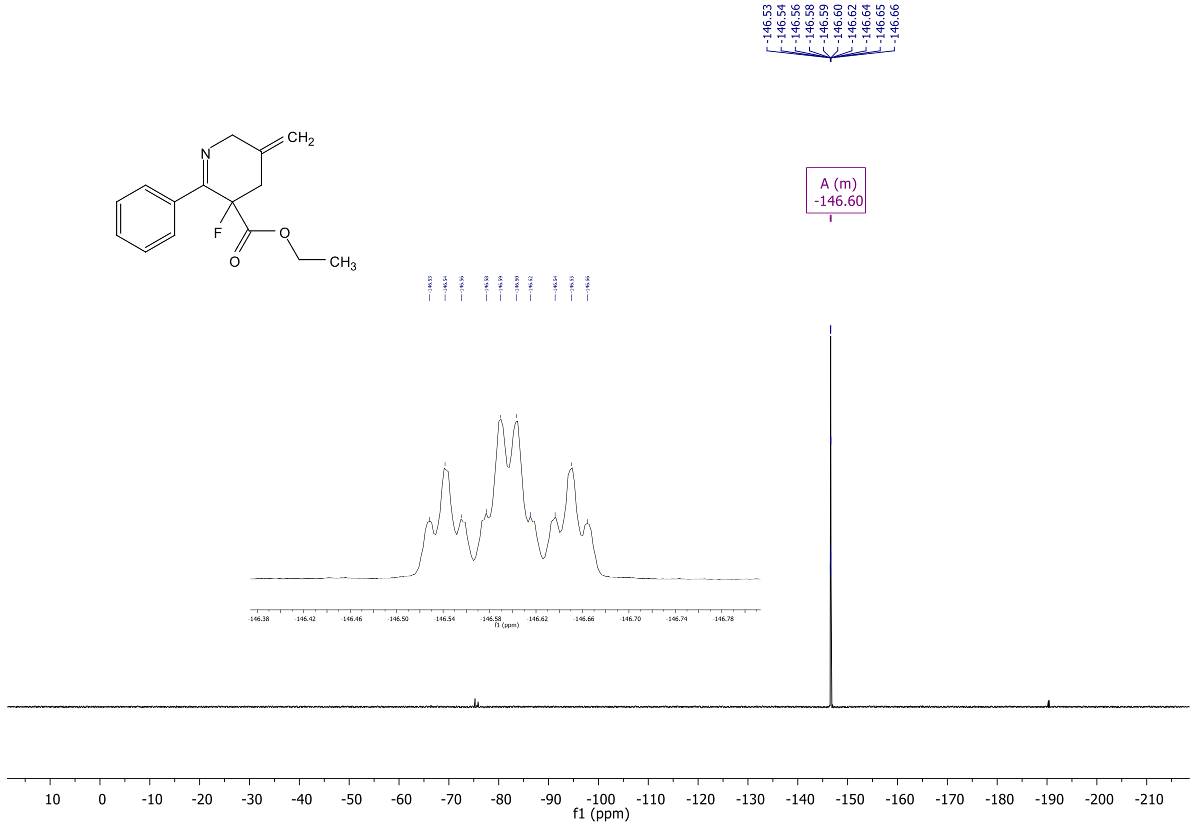

^{13}C NMR, $CDCl_3$, 101 MHz

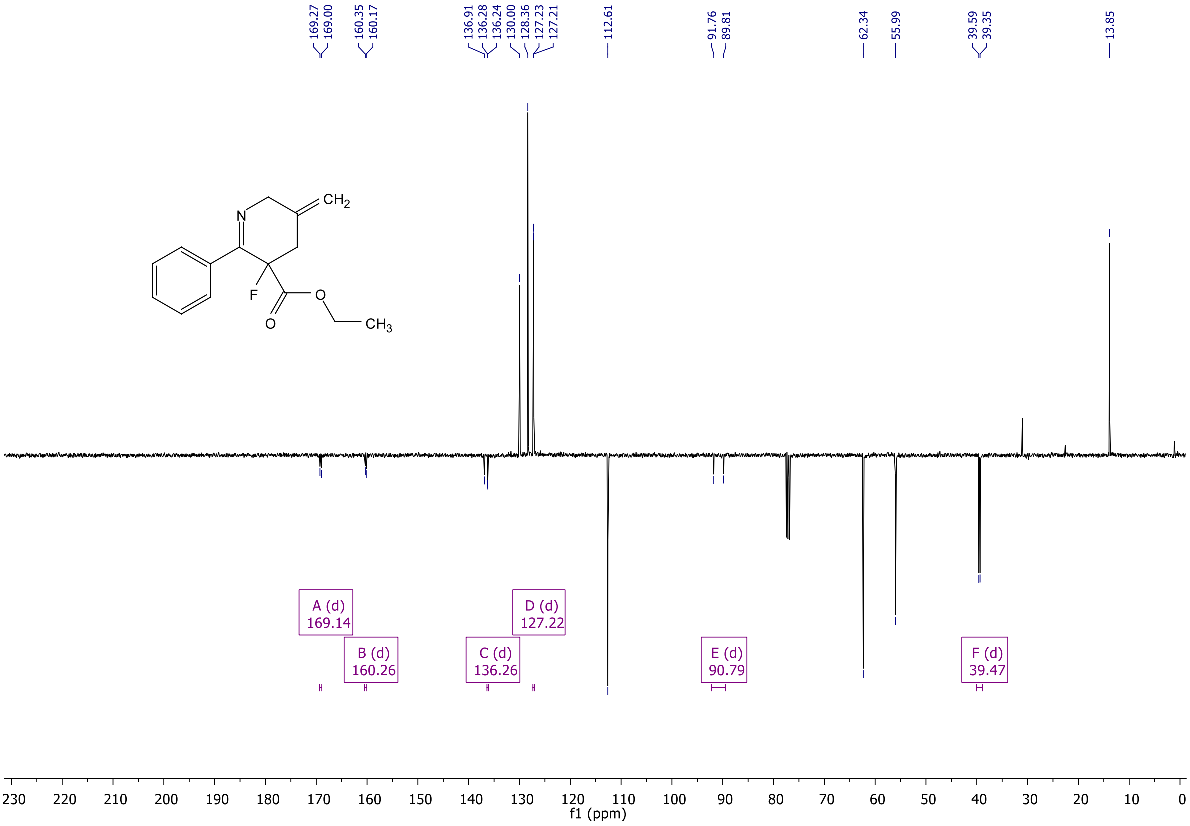

Ethyl 3-fluoro-2-(4-methoxyphenyl)-5-methylidene-3,4,5,6-tetrahydropyridine-3-carboxylate (4b)

^{1}H NMR, $CDCl_3$, 400 MHz

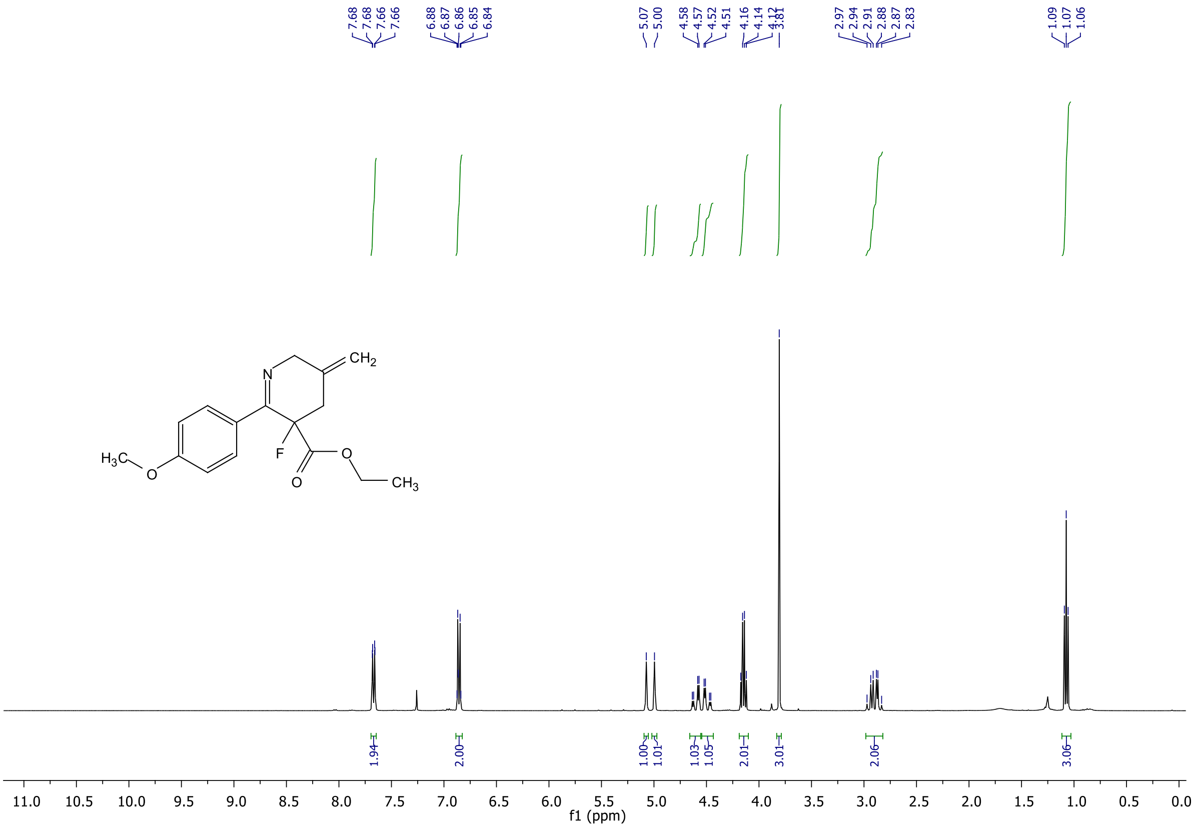

^{19}F NMR, $CDCl_3$, 377 MHz

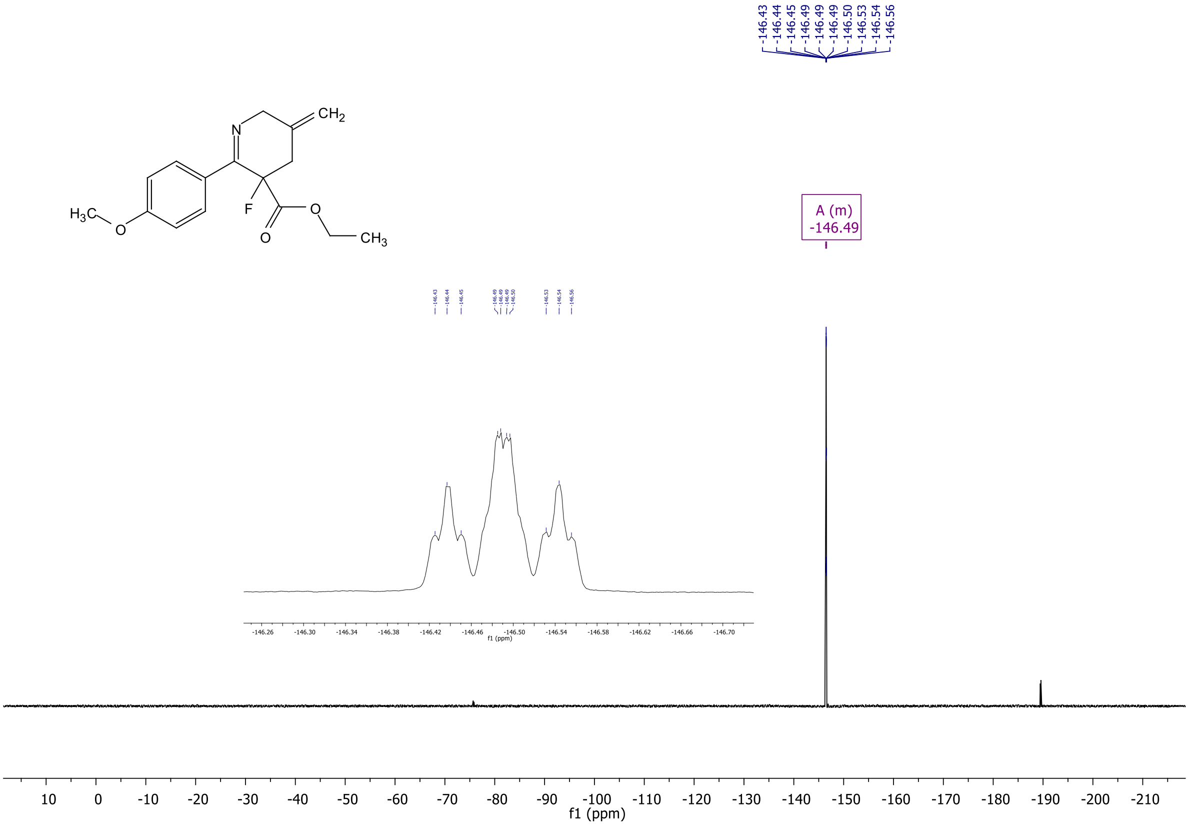

^{13}C NMR, $CDCl_3$, 101 MHz

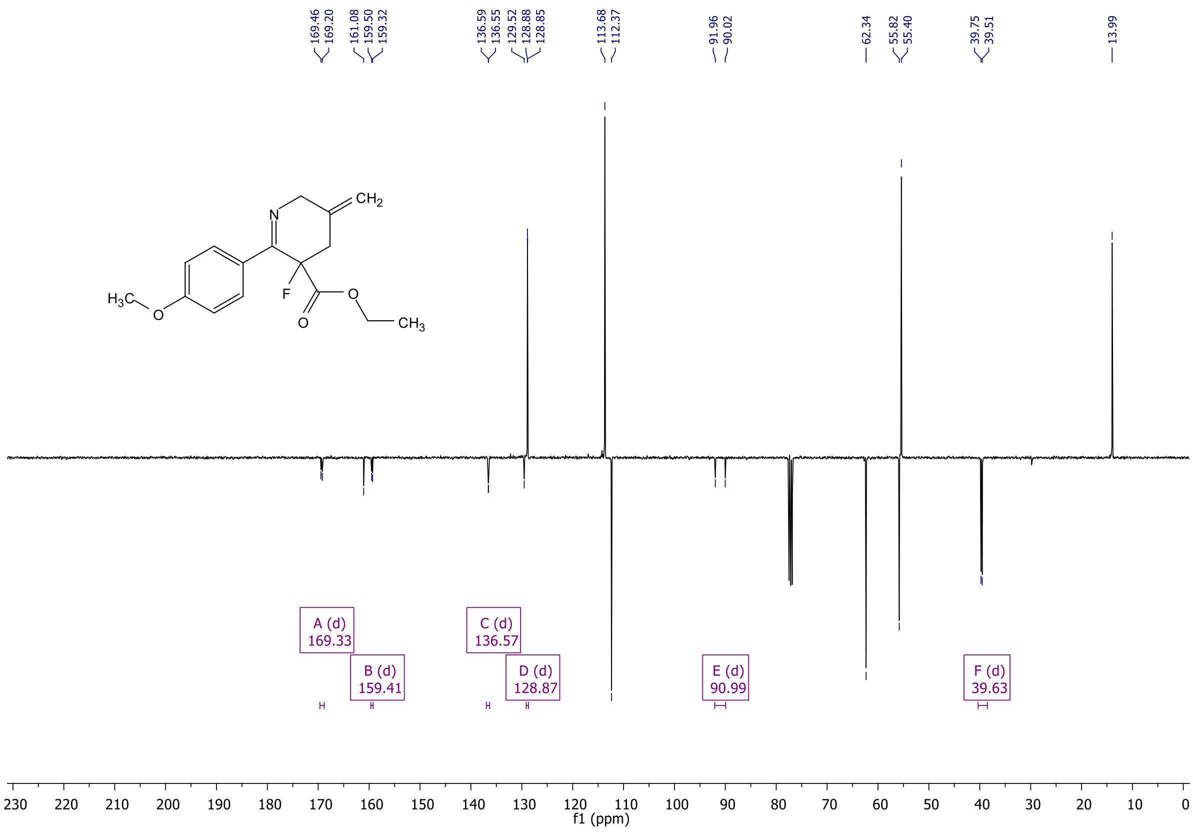

Ethyl 3-fluoro-2-(4-chlorophenyl)-5-methylidene-3,4,5,6-tetrahydropyridine-3-carboxylate (4c)

^{1}H NMR, $CDCl_3$, 400 MHz

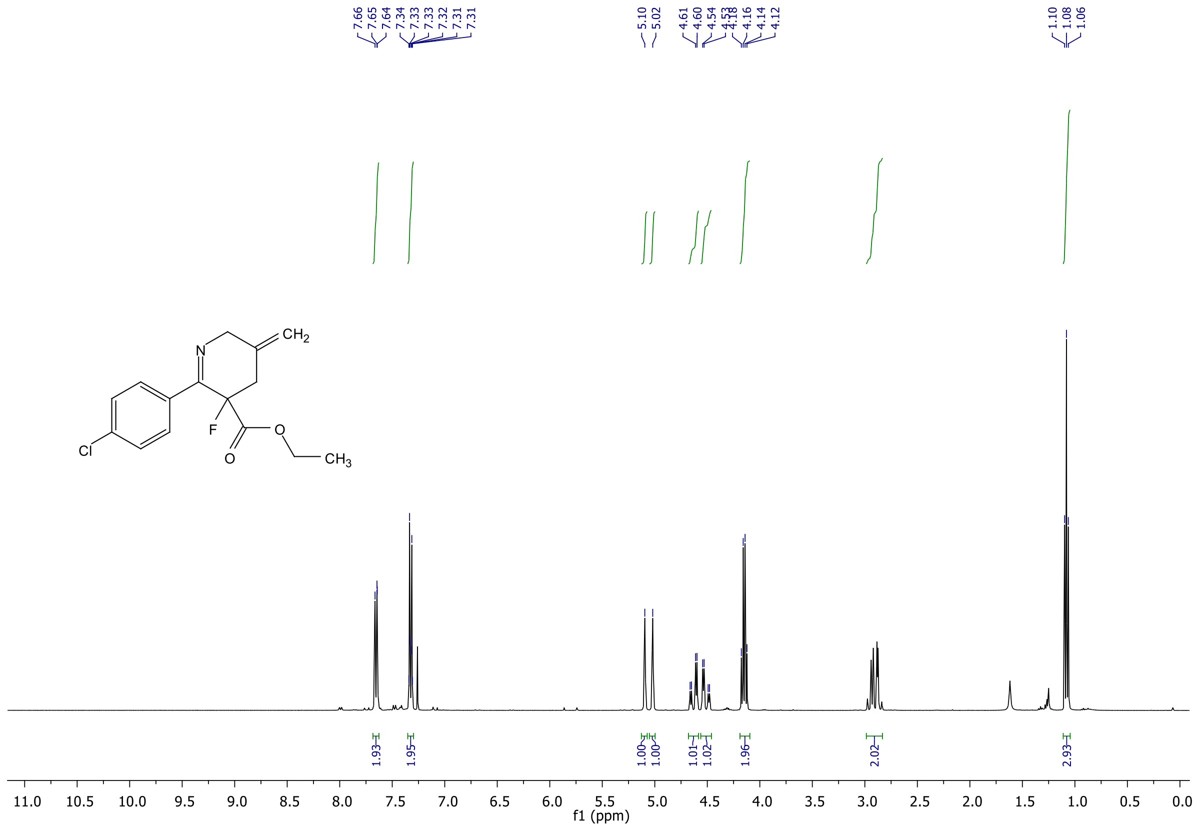

^{19}F NMR, $CDCl_3$, 377 MHz

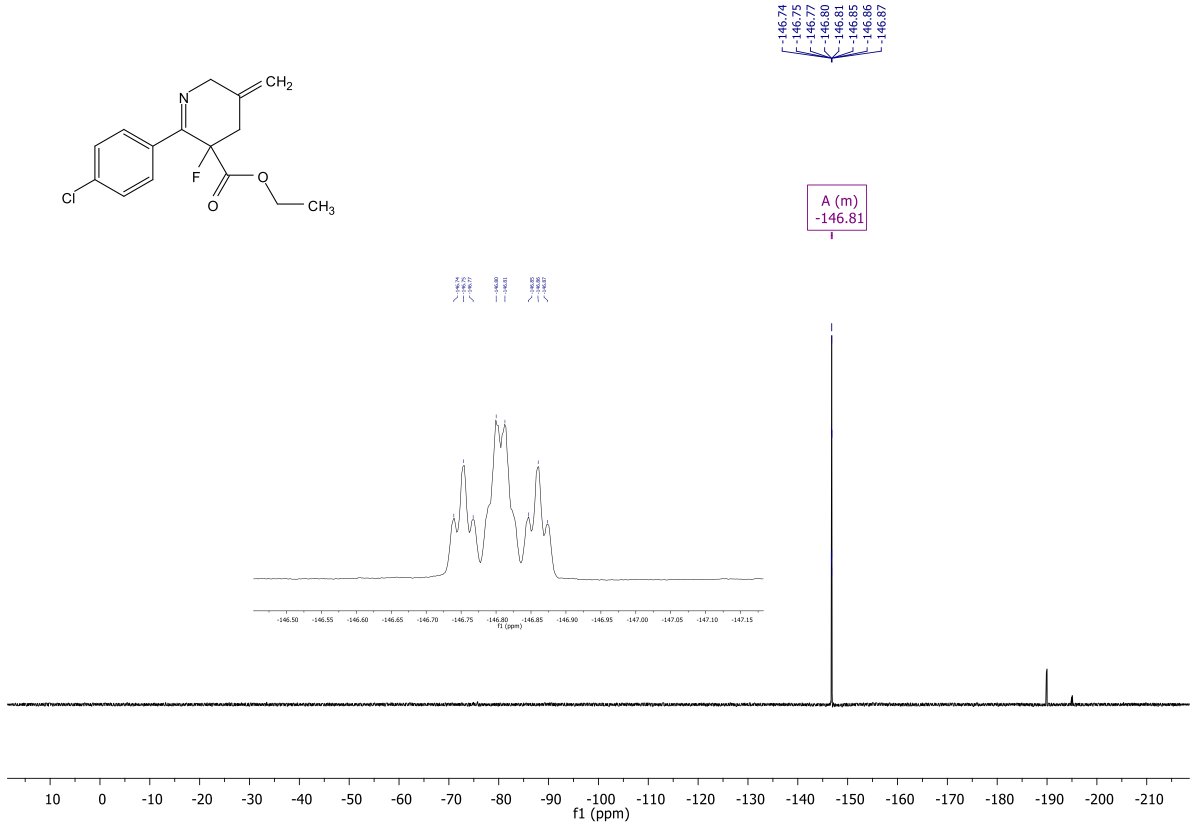

^{13}C NMR, $CDCl_3$, 101 MHz

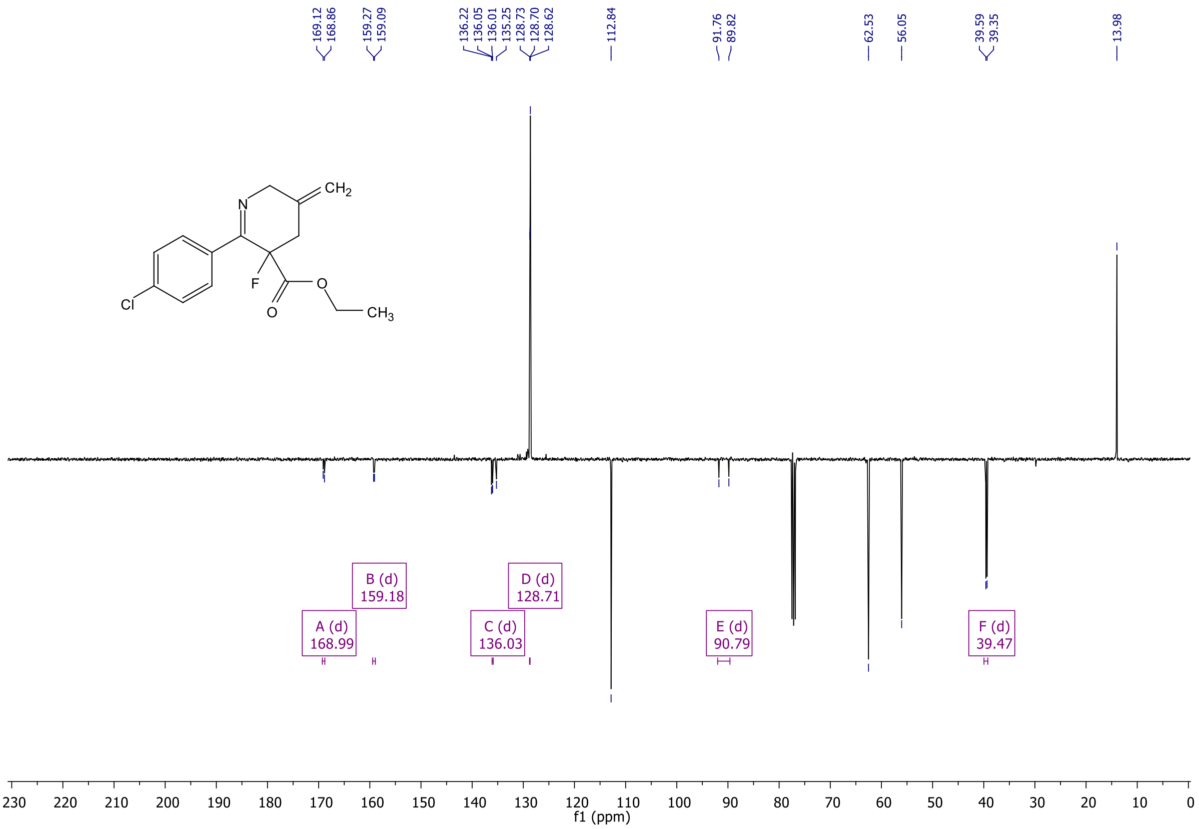

Ethyl 3-fluoro-2-(4-trifluorophenyl)-5-methylidene-3,4,5,6-tetrahydropyridine-3-carboxylate (4d)

^{1}H NMR, $CDCl_3$, 400 MHz

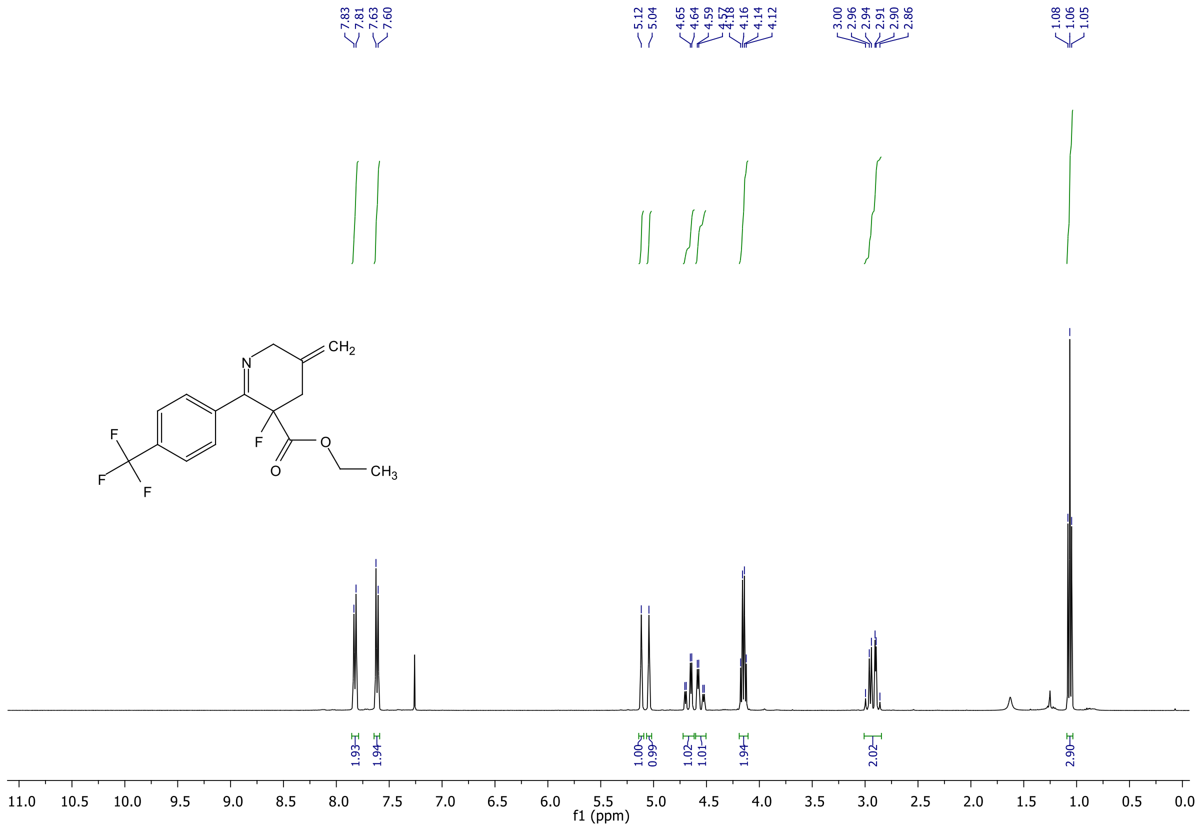

^{19}F NMR, $CDCl_3$, 377 MHz

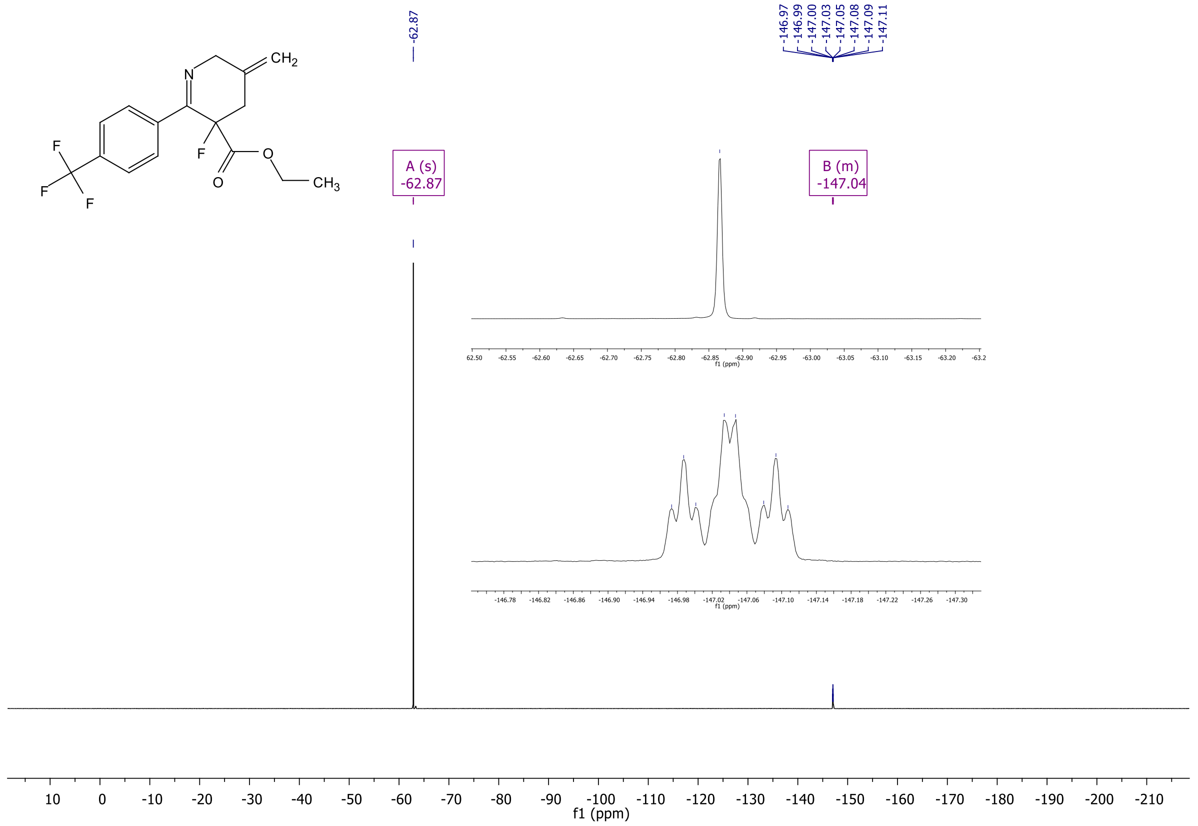

^{13}C NMR, $CDCl_3$, 101 MHz

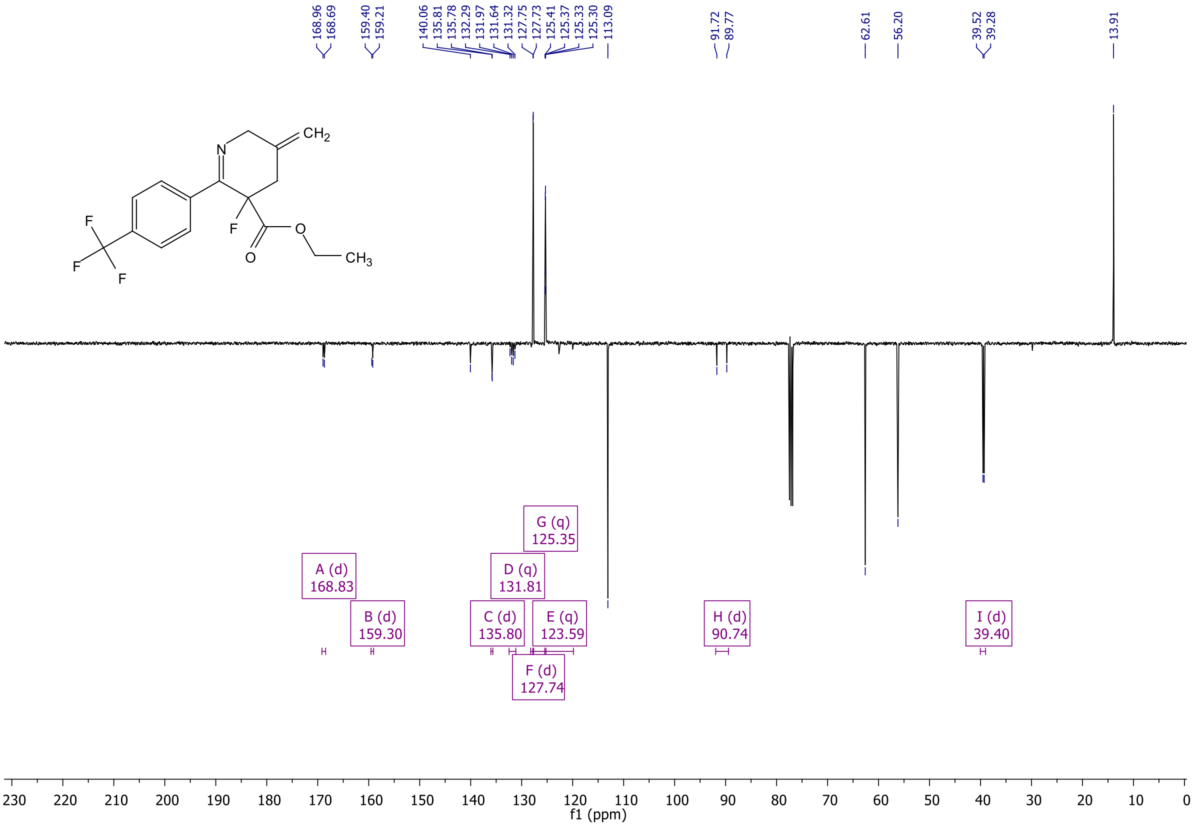

Ethyl 3-fluoro-2-(4-methylphenyl)-5-methylidene-3,4,5,6-tetrahydropyridine-3-carboxylate (4e)

^{1}H NMR, $CDCl_3$, 400 MHz

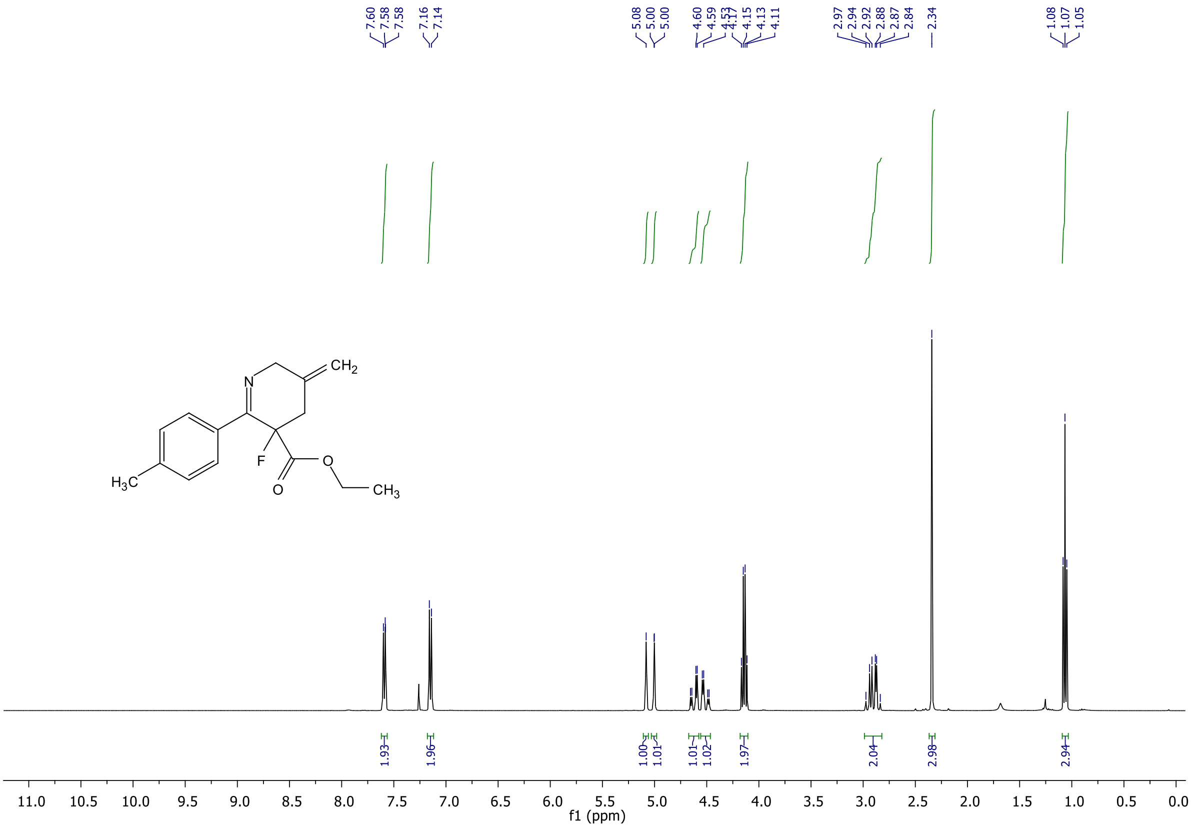

^{19}F NMR, $CDCl_3$, 377 MHz

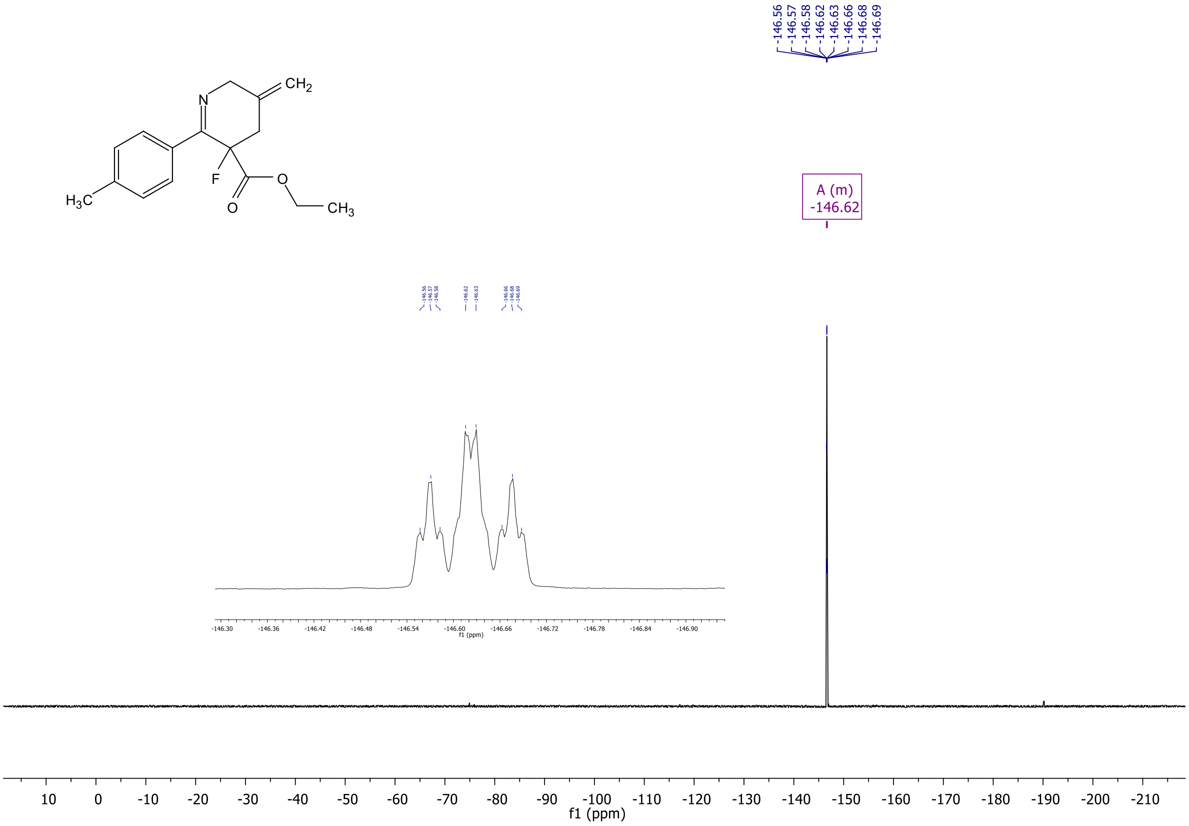

^{13}C NMR, $CDCl_3$, 101 MHz

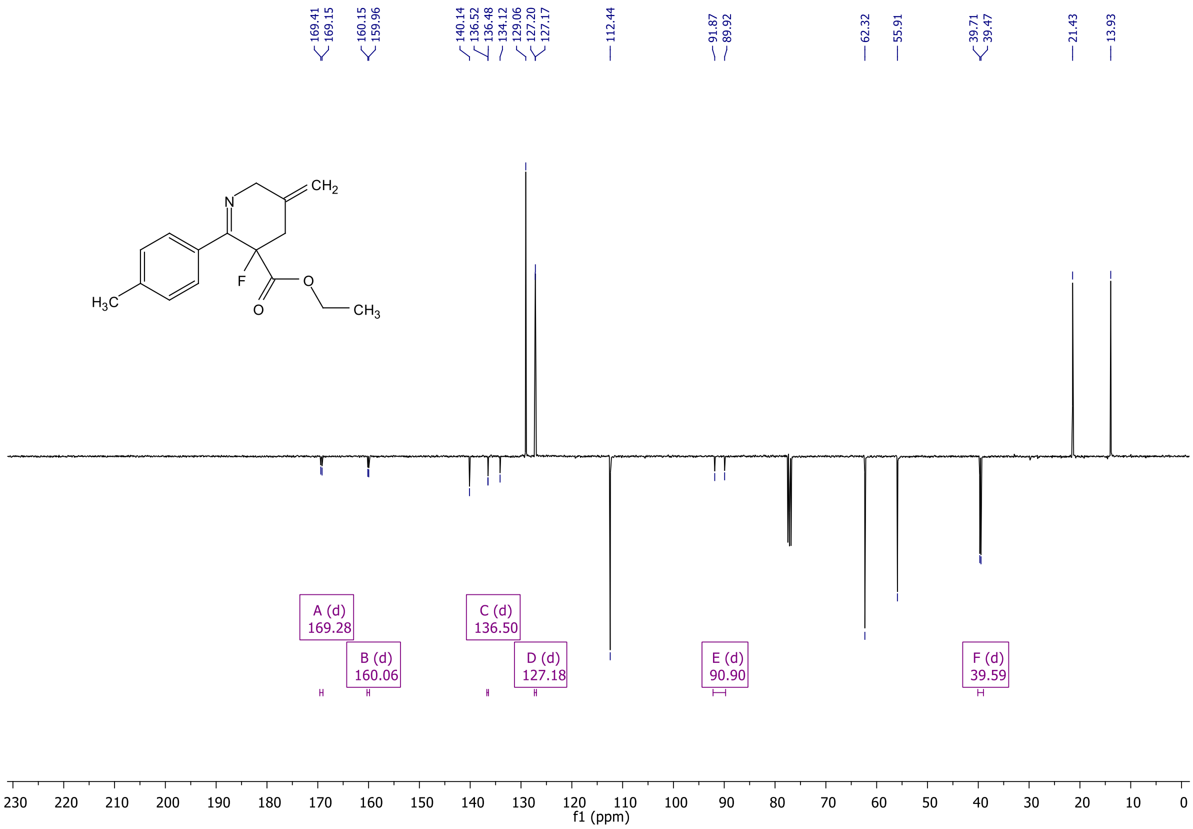

Ethyl 3-fluoro-2-(4-nitrophenyl)-5-methylidene-3,4,5,6-tetrahydropyridine-3-carboxylate (4f)

^{1}H NMR, CDCl$_3$, 400 MHz

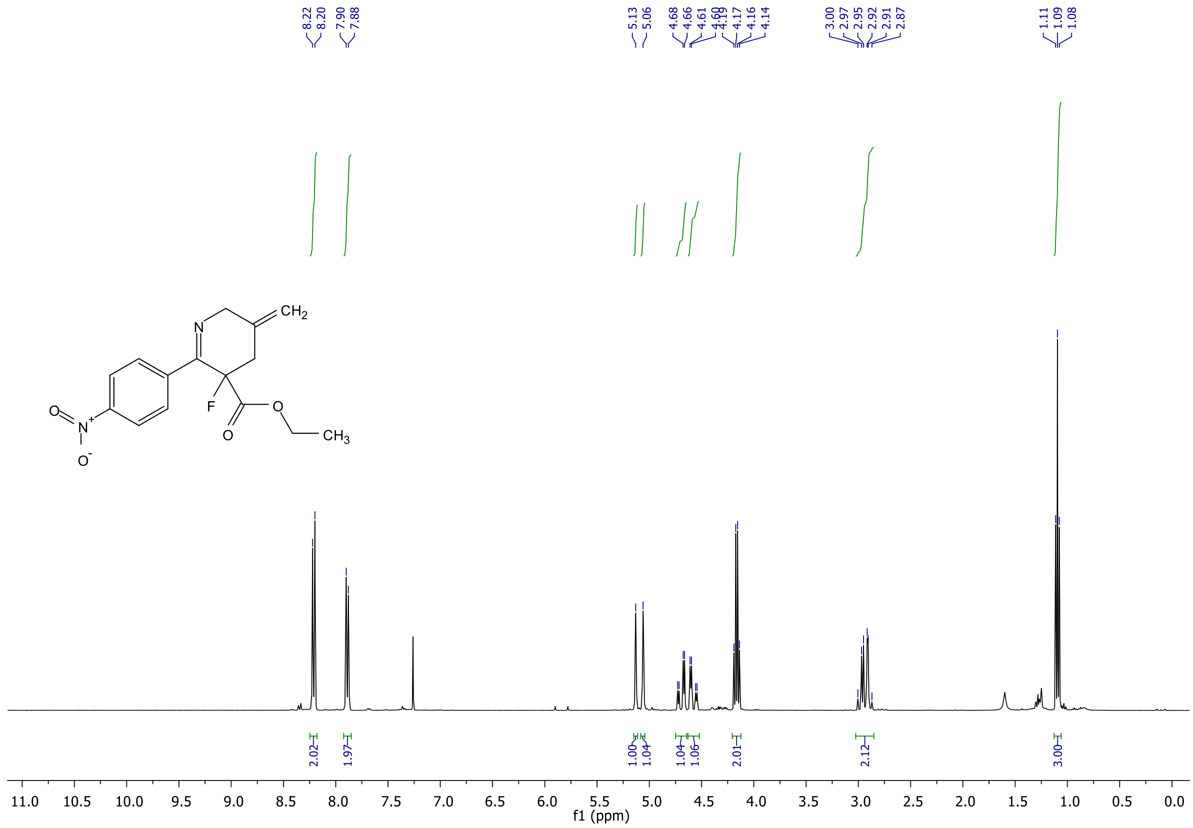

^{19}F NMR, CDCl$_3$, 377 MHz

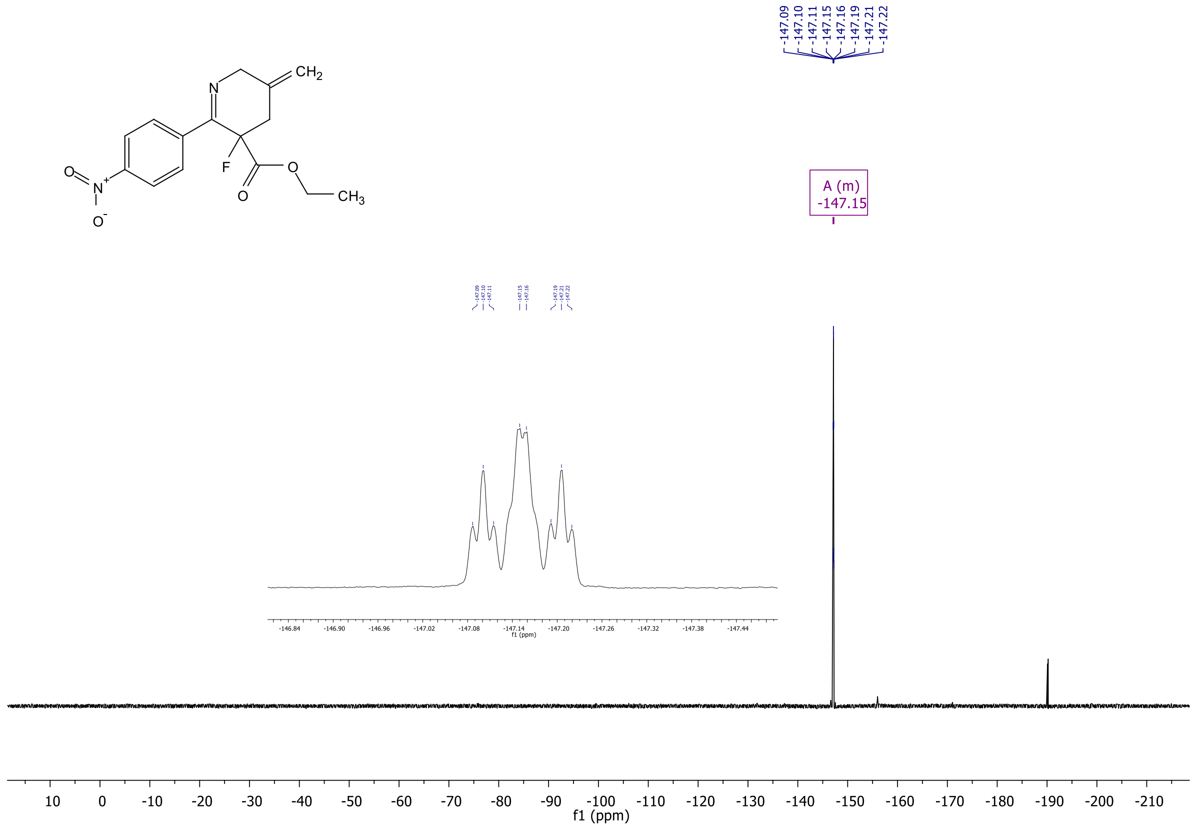

^{13}C NMR, $CDCl_3$, 101 MHz

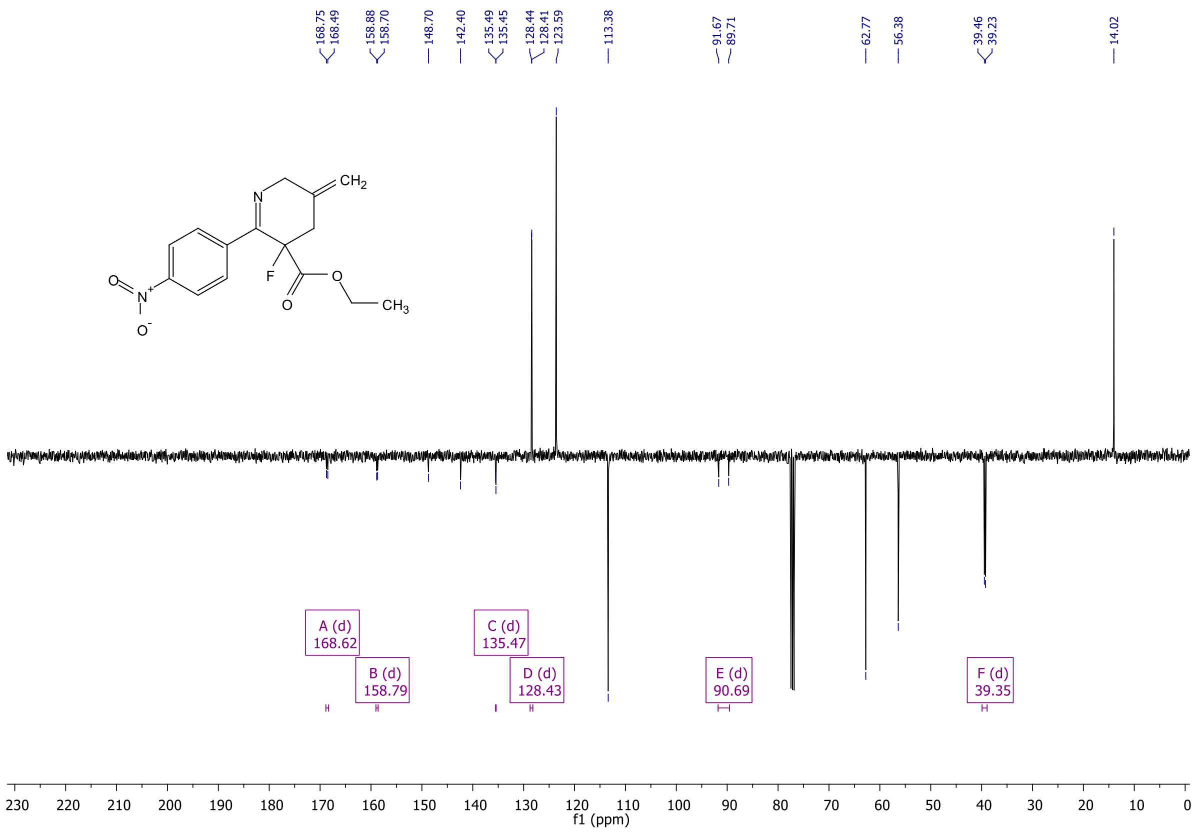

Ethyl 3-fluoro-2-(4-methylphenyl)-5-methylidene-3,4,5,6-tetrahydropyridine-3-carboxylate (4g)

^{1}H NMR, $CDCl_3$, 400 MHz

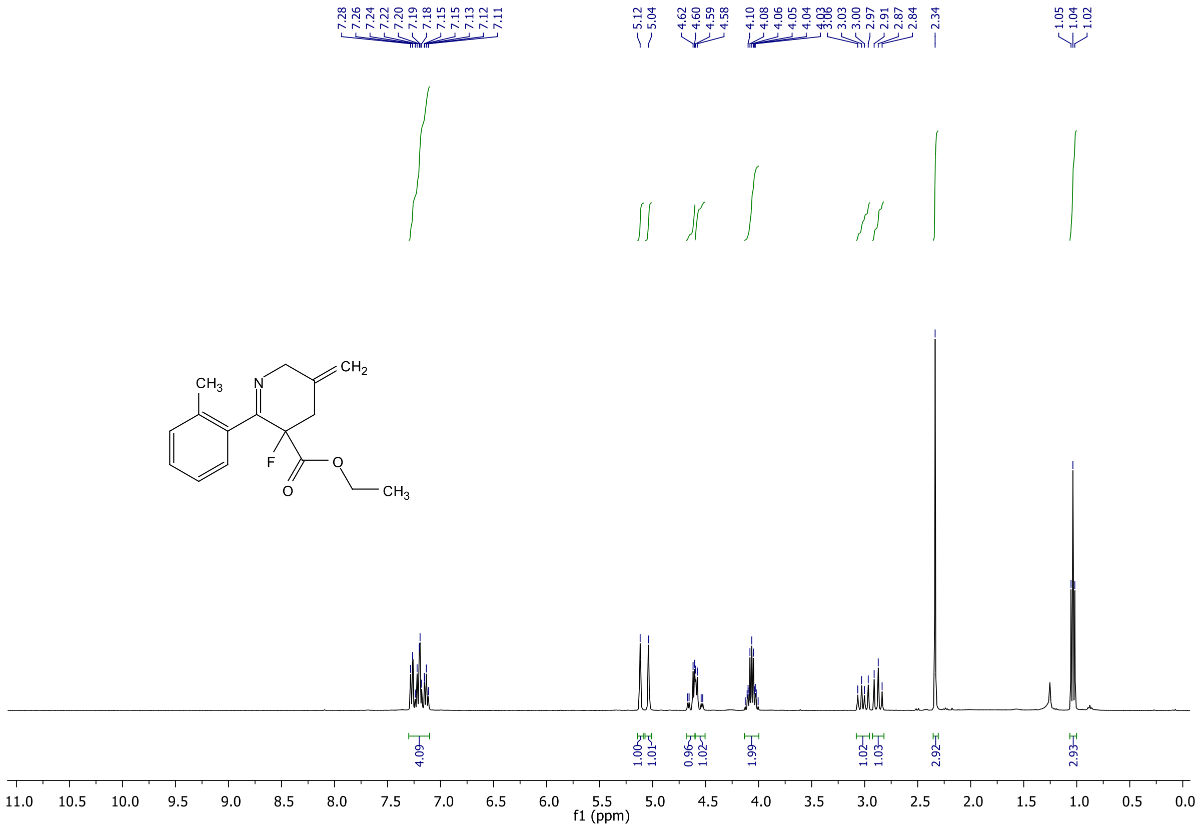

^{19}F NMR, $CDCl_3$, 377 MHz

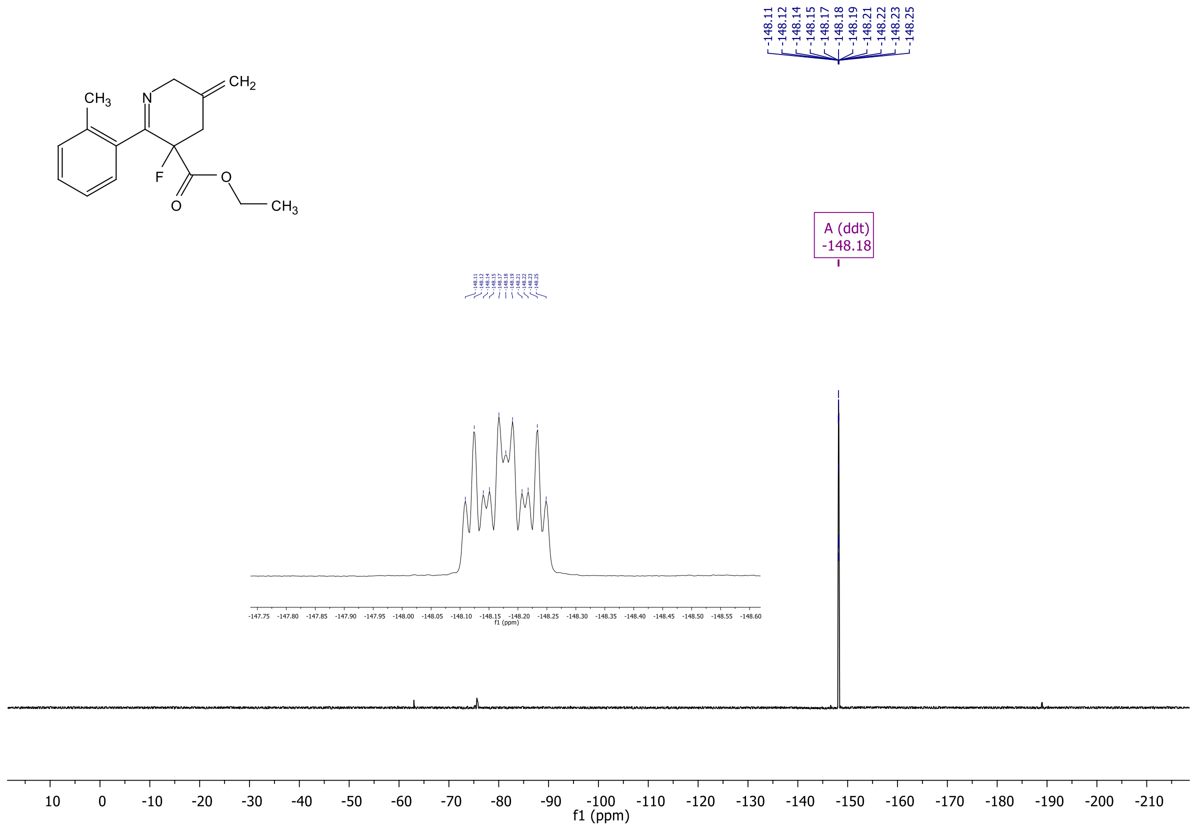

^{13}C NMR, $CDCl_3$, 101 MHz

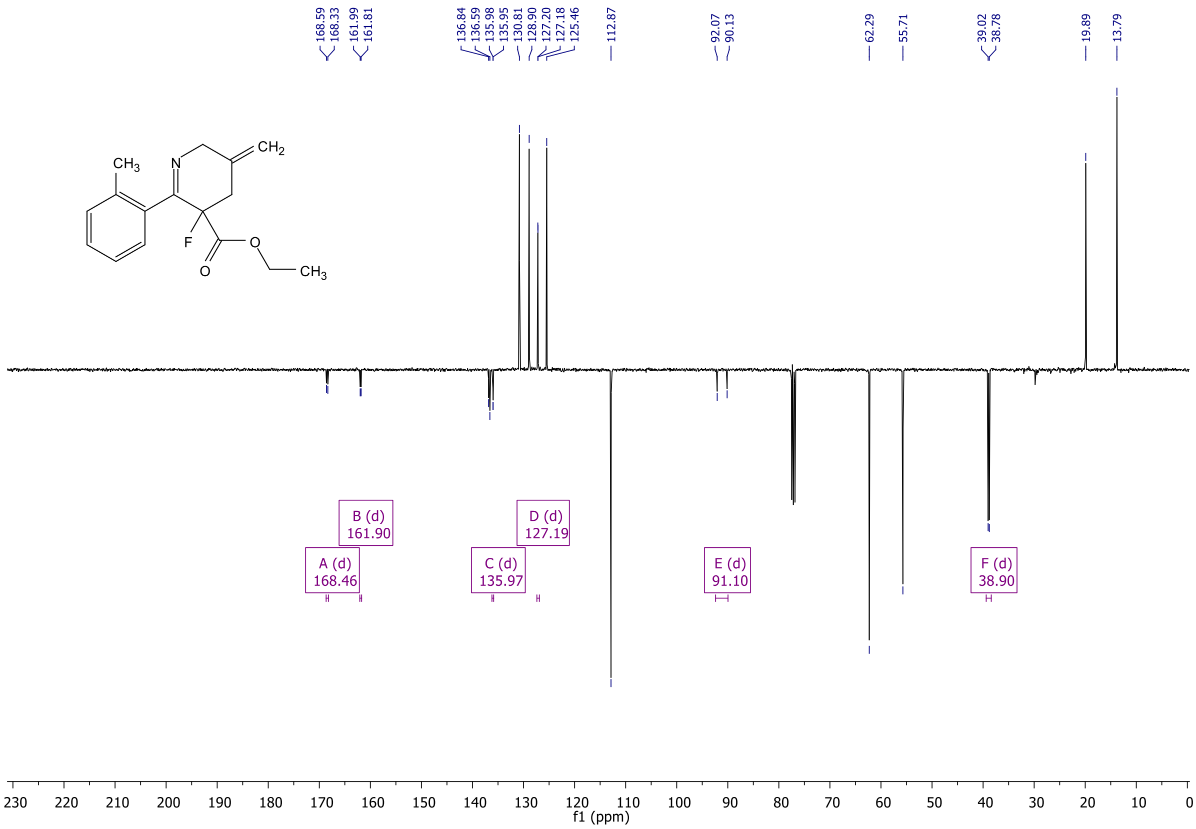

Ethyl 3-fluoro-5-methylene-2-(naphthalen-2-yl)-3,4,5,6-tetrahydropyridine-3-carboxylate (4h)

^{1}H NMR, $CDCl_3$, 400 MHz

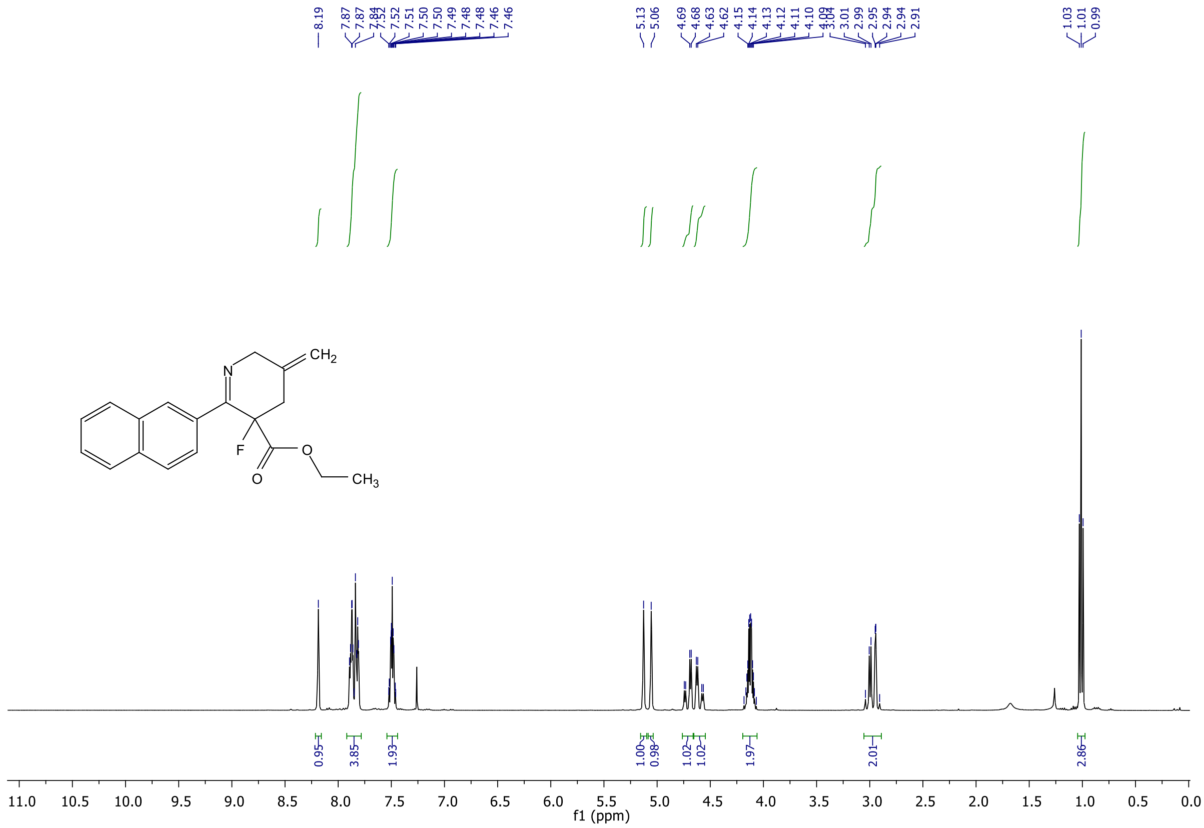

^{19}F NMR, $CDCl_3$, 377 MHz

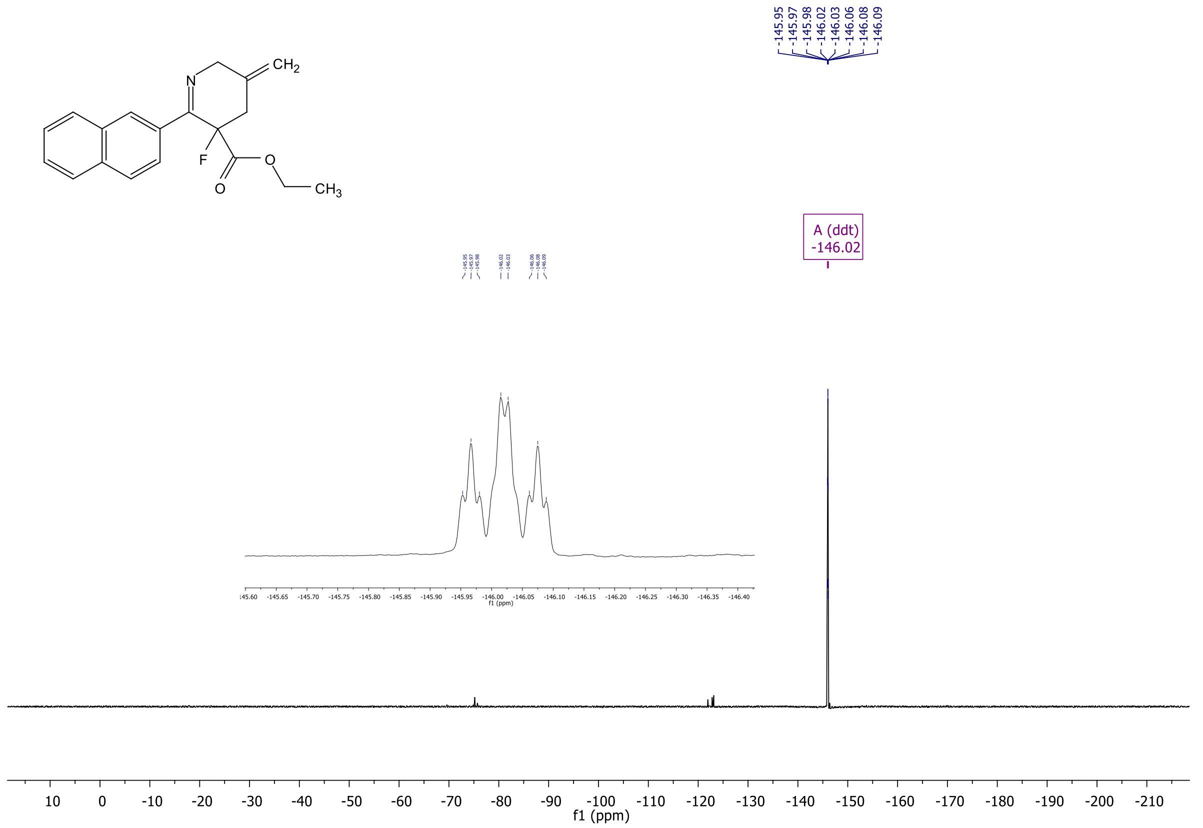

^{13}C NMR, $CDCl_3$, 101 MHz

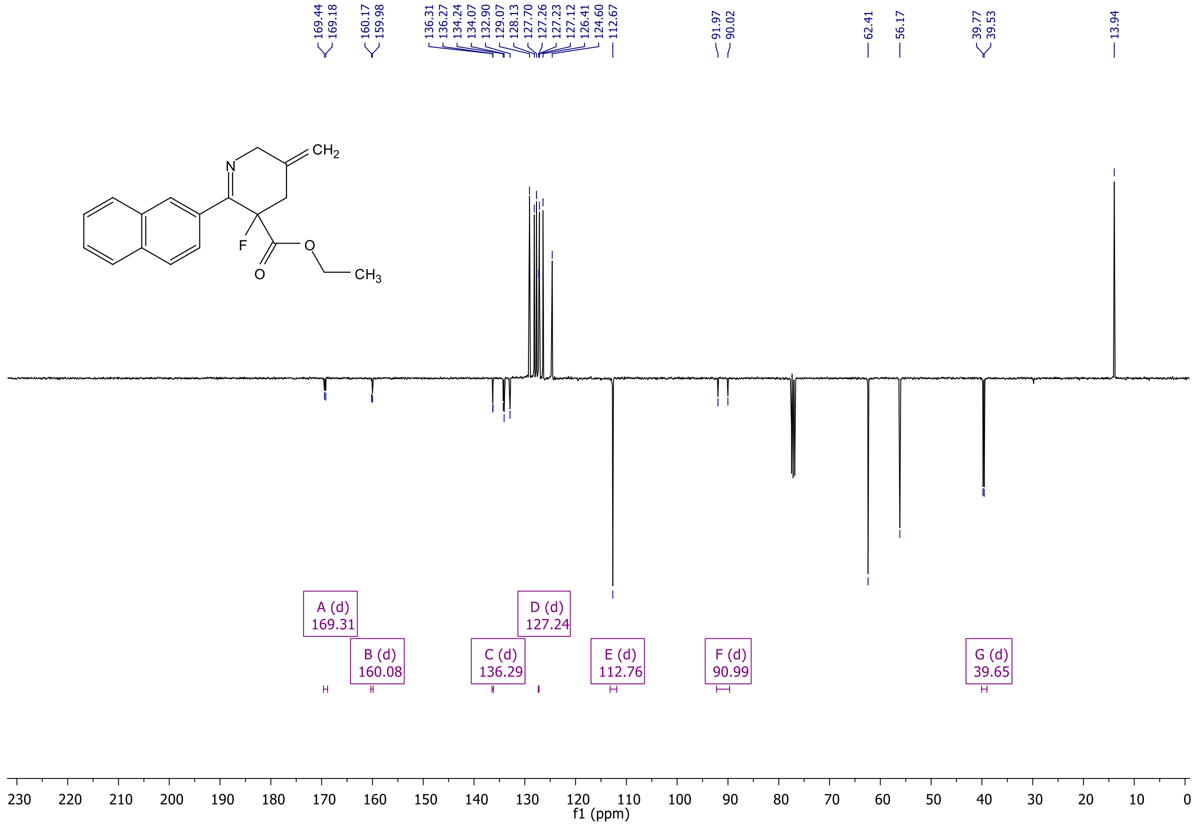

Ethyl 3-fluoro-5-methylidene-2-(thiophen-2-yl)-3,4,5,6-tetrahydropyridine-3-carboxylate (4i)

^{1}H NMR, $CDCl_3$, 400 MHz

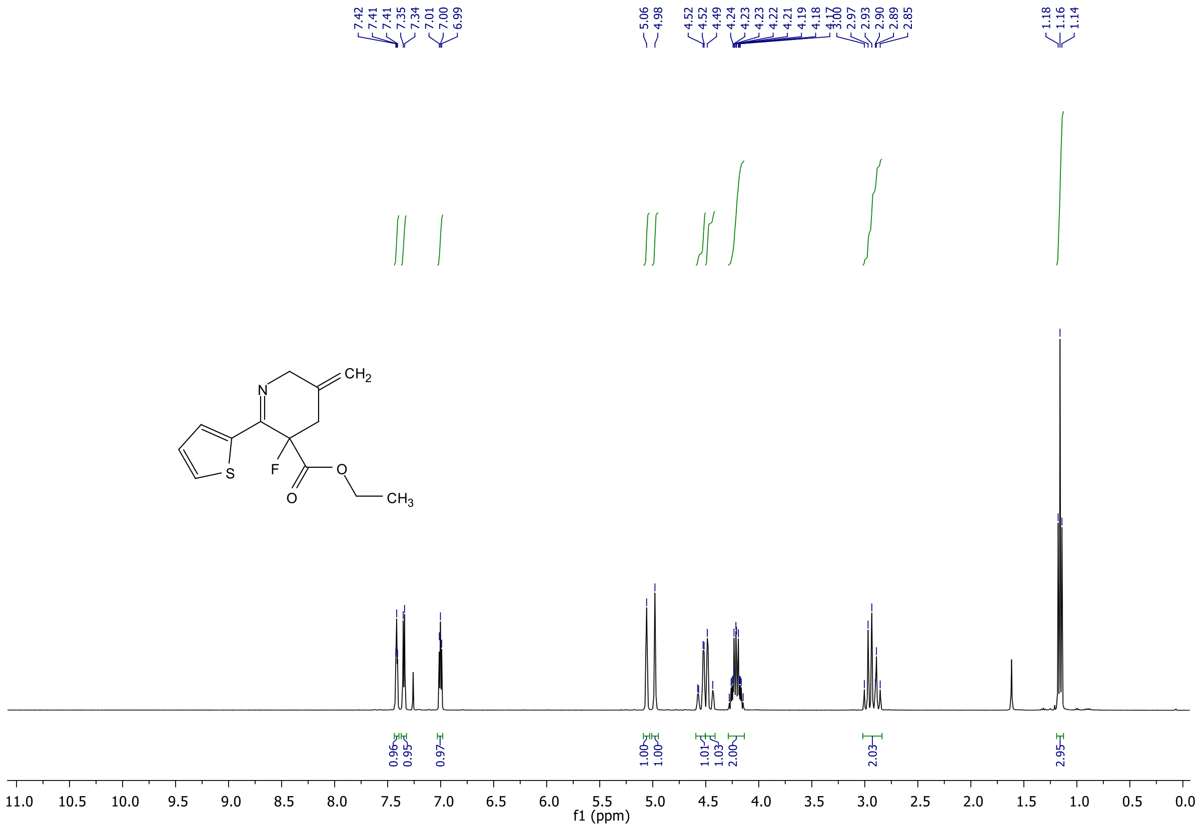

^{19}F NMR, $CDCl_3$, 377 MHz

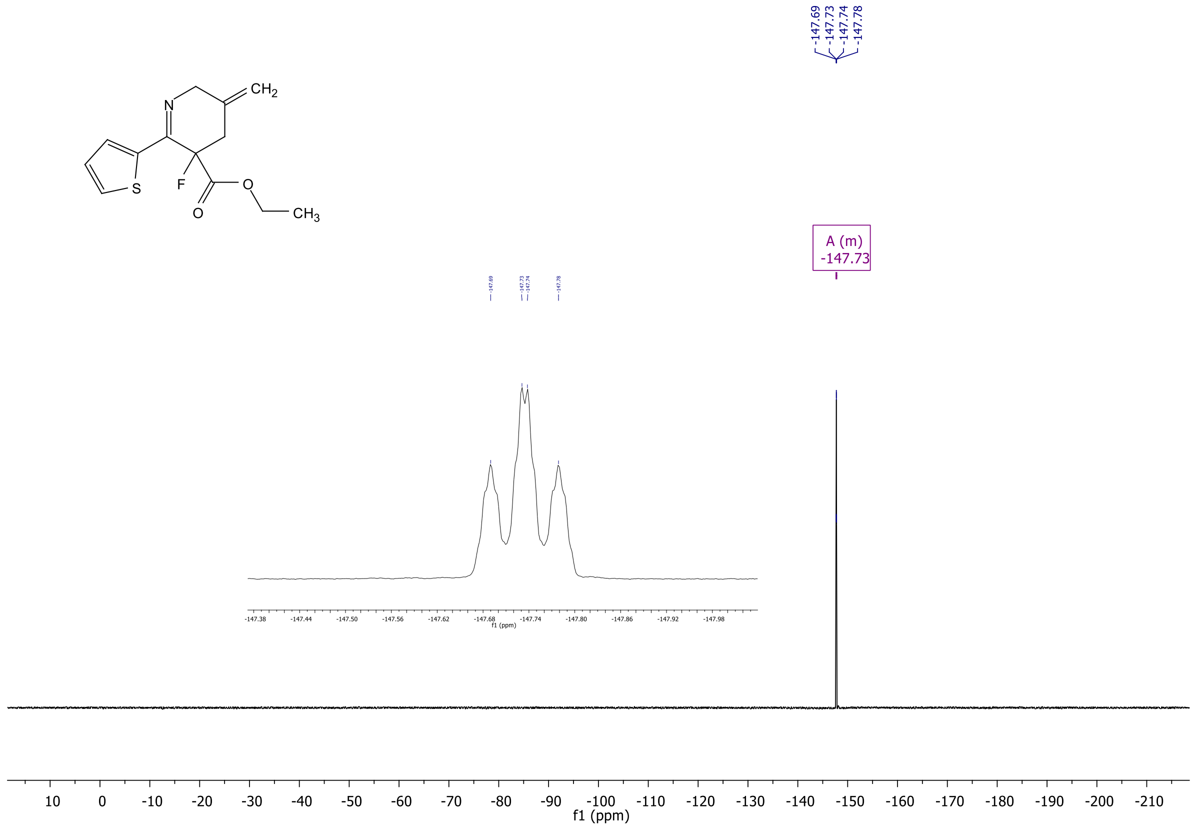

^{13}C NMR, $CDCl_3$, 101 MHz

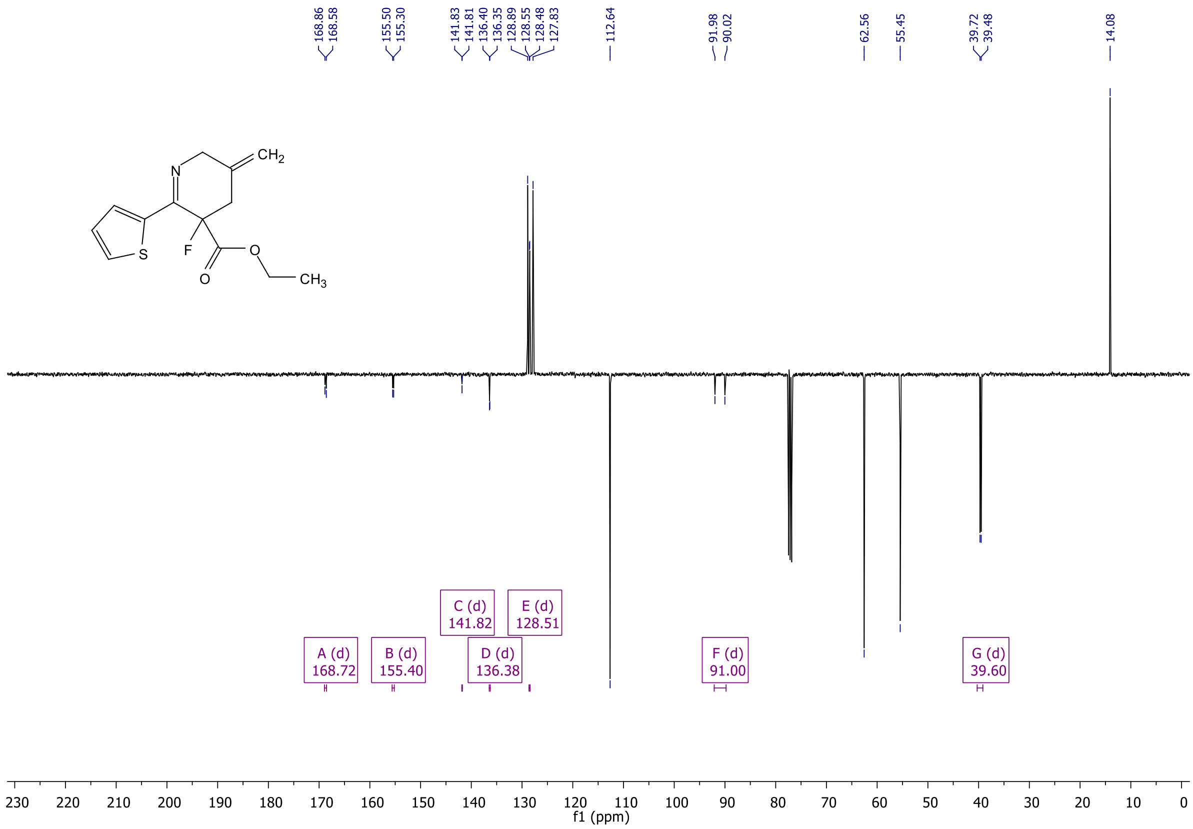

3-Fluoro-5-methylidene-2-phenyl-4,6-dihydropyridine-3-carbonitrile (4j)

^{1}H NMR, CDCl$_3$, 400 MHz

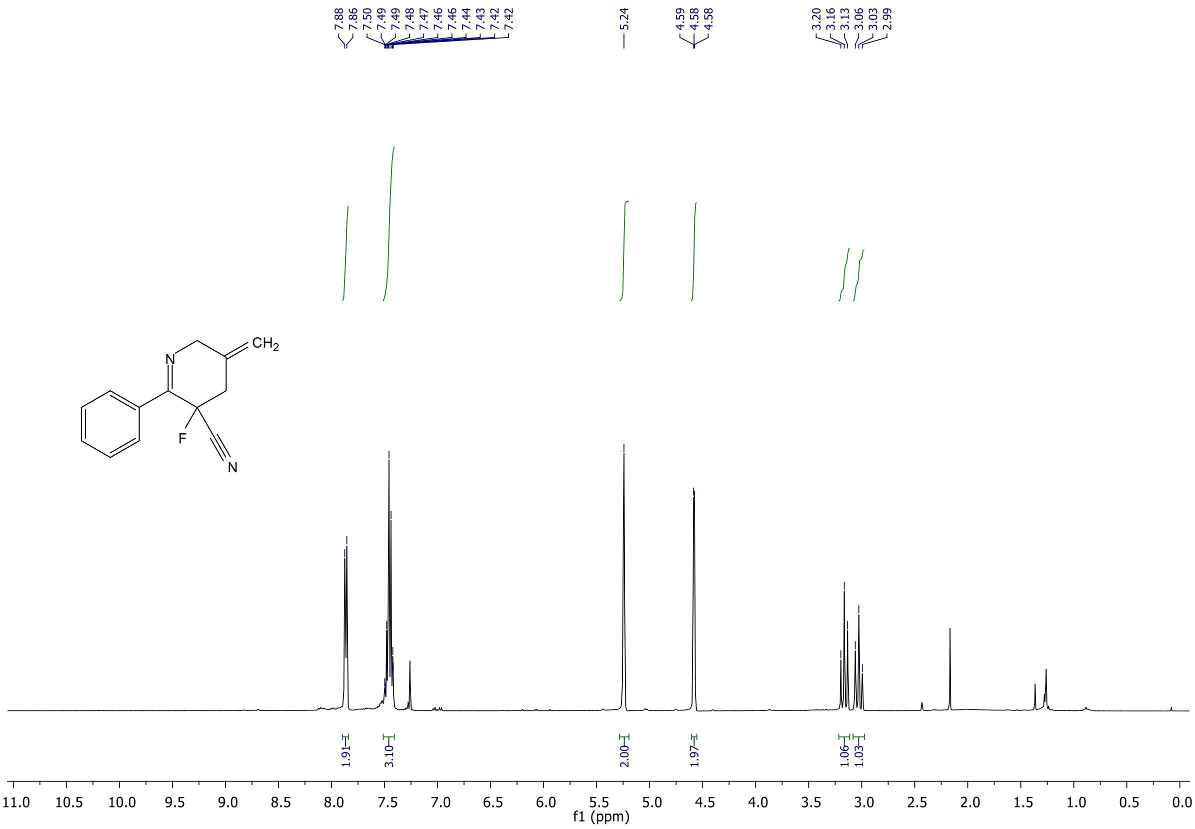

^{19}F NMR, CDCl$_3$, 377 MHz

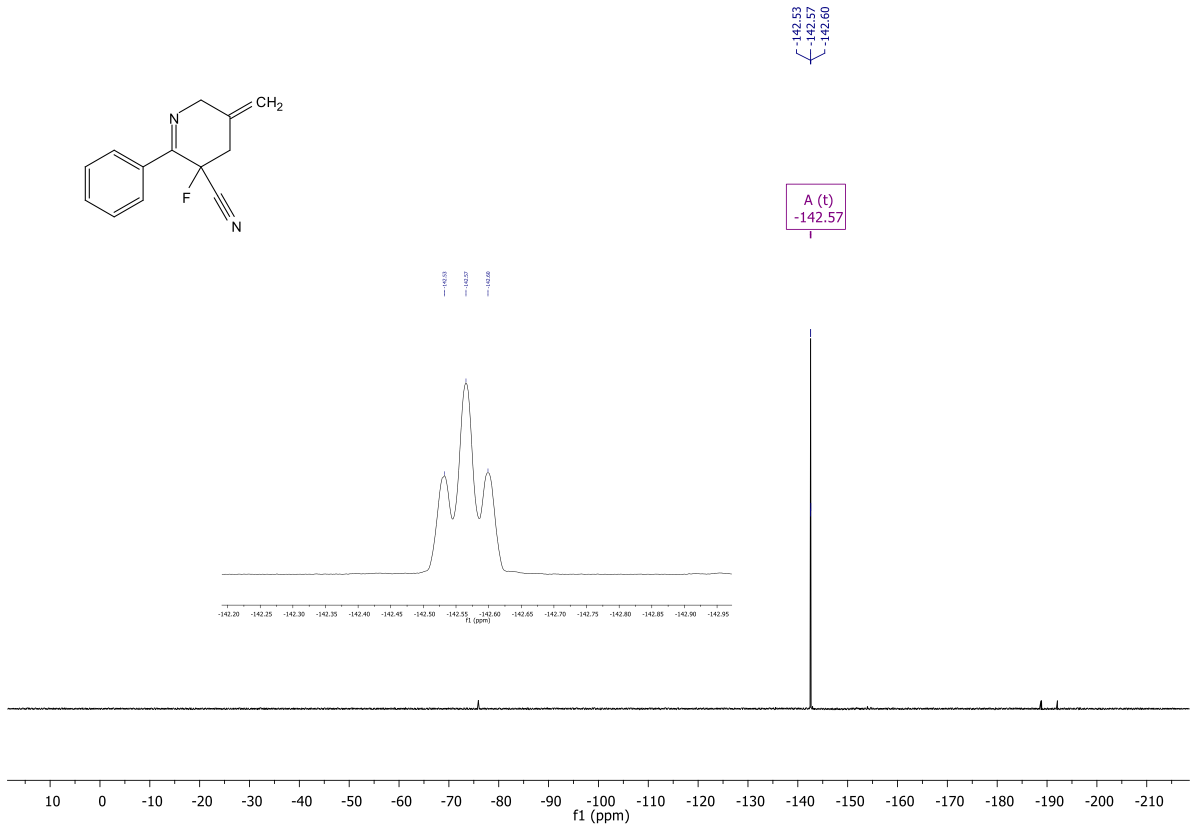

^{13}C NMR, CDCl$_3$, 101 MHz

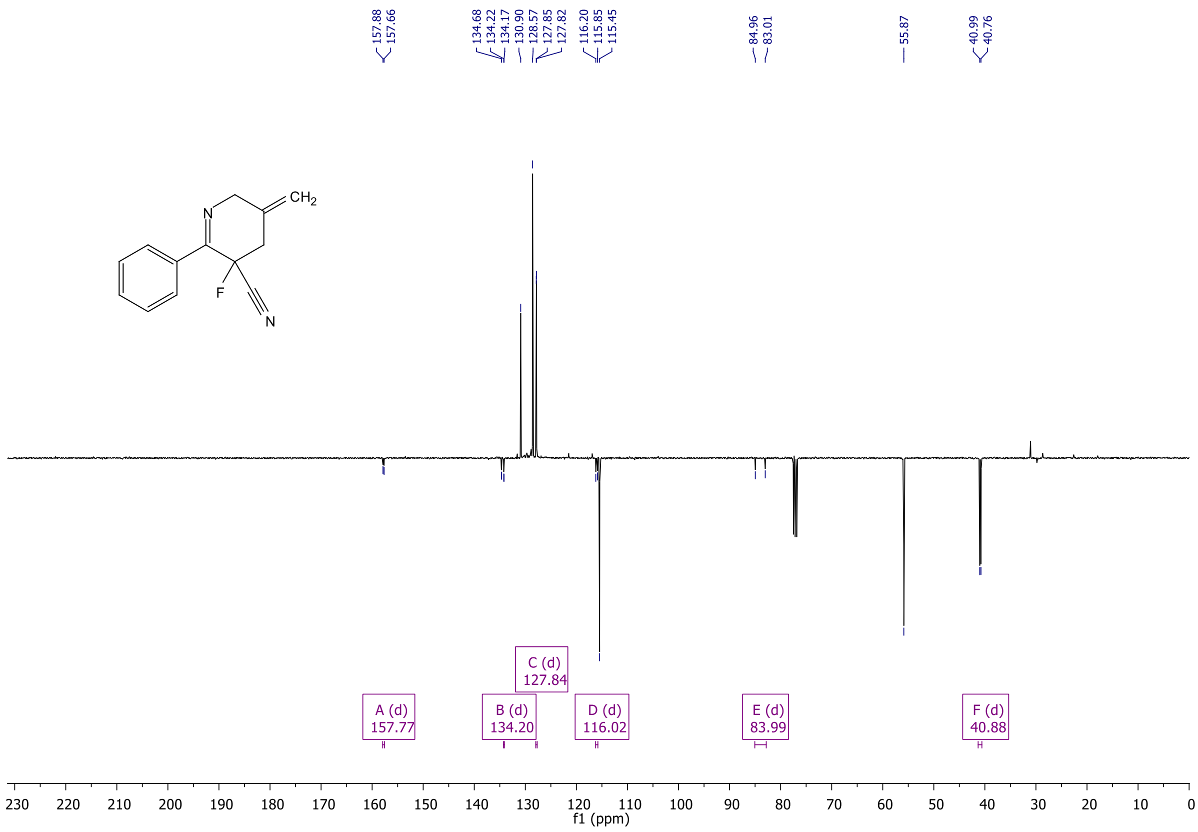

5-Fluoro-3-methylene-6-phenyl-5-tosyl-2,3,4,5-tetrahydropyridine (4k)

^{1}H NMR, $CDCl_3$, 400 MHz

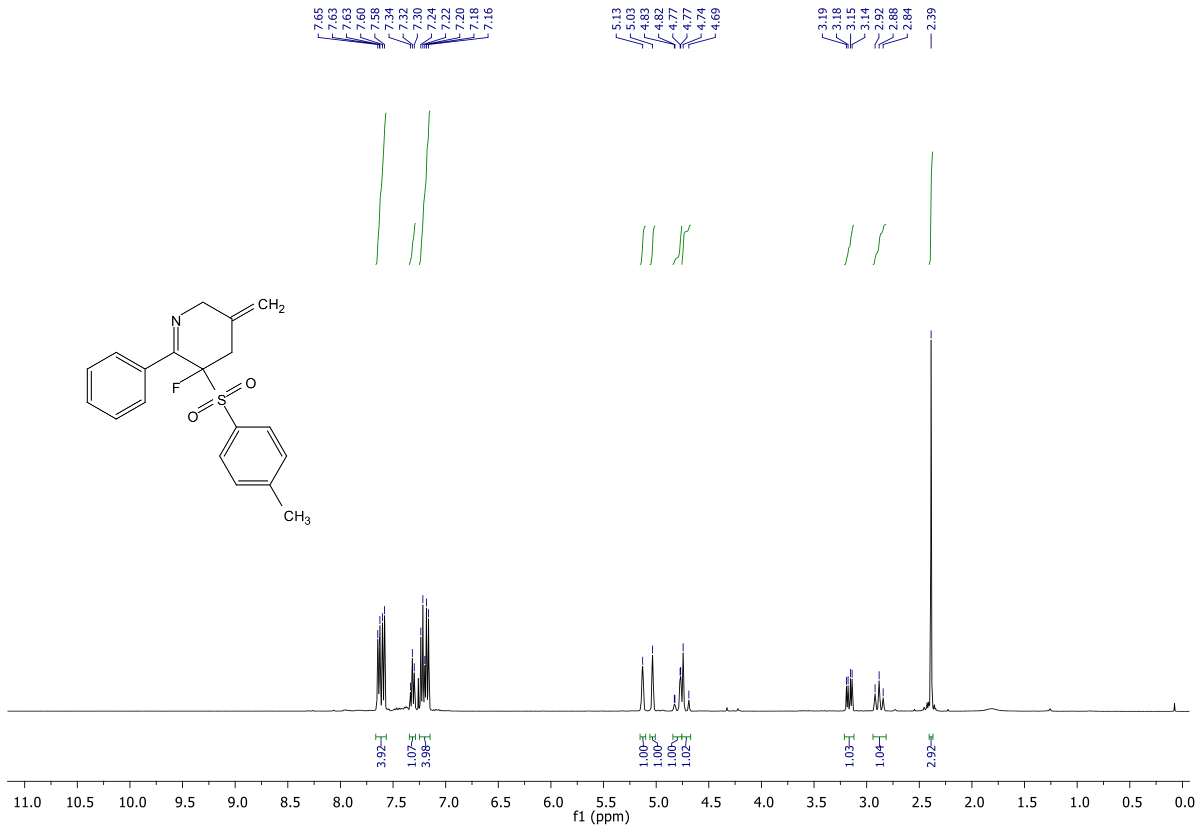

^{19}F NMR, $CDCl_3$, 377 MHz

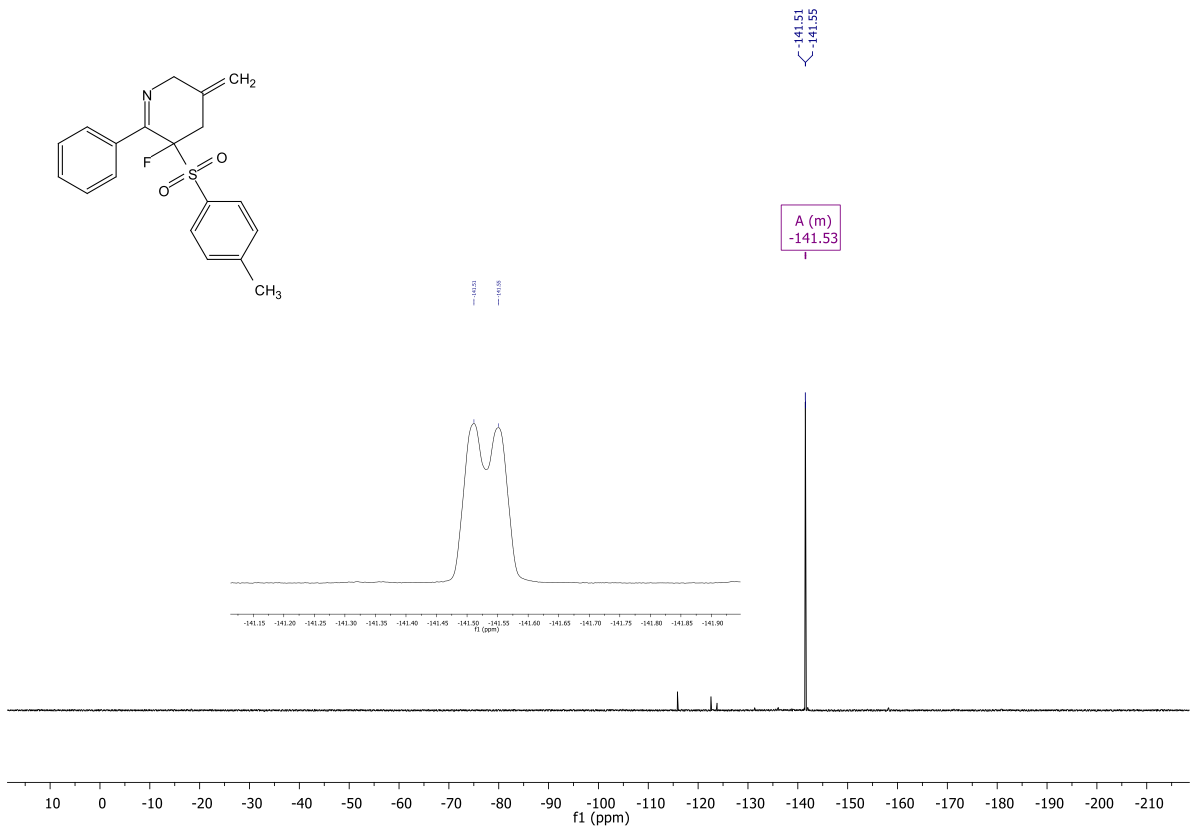

^{13}C NMR, $CDCl_3$, 101 MHz

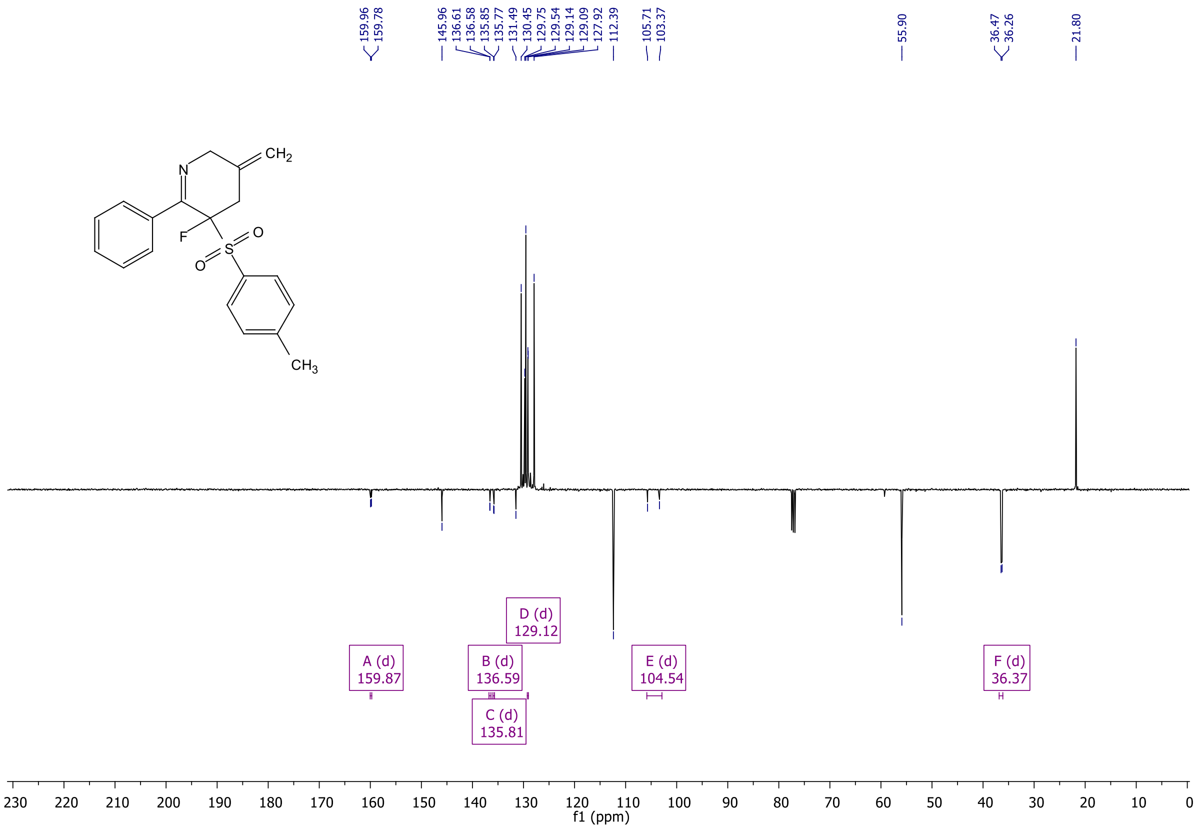

3-Fluoro-*N*-methoxy-*N*-methyl-5-methylene-2-phenyl-3,4,5,6-tetrahydropyridine-3-carboxamide (4l)

^{1}H NMR, $CDCl_3$, 400 MHz

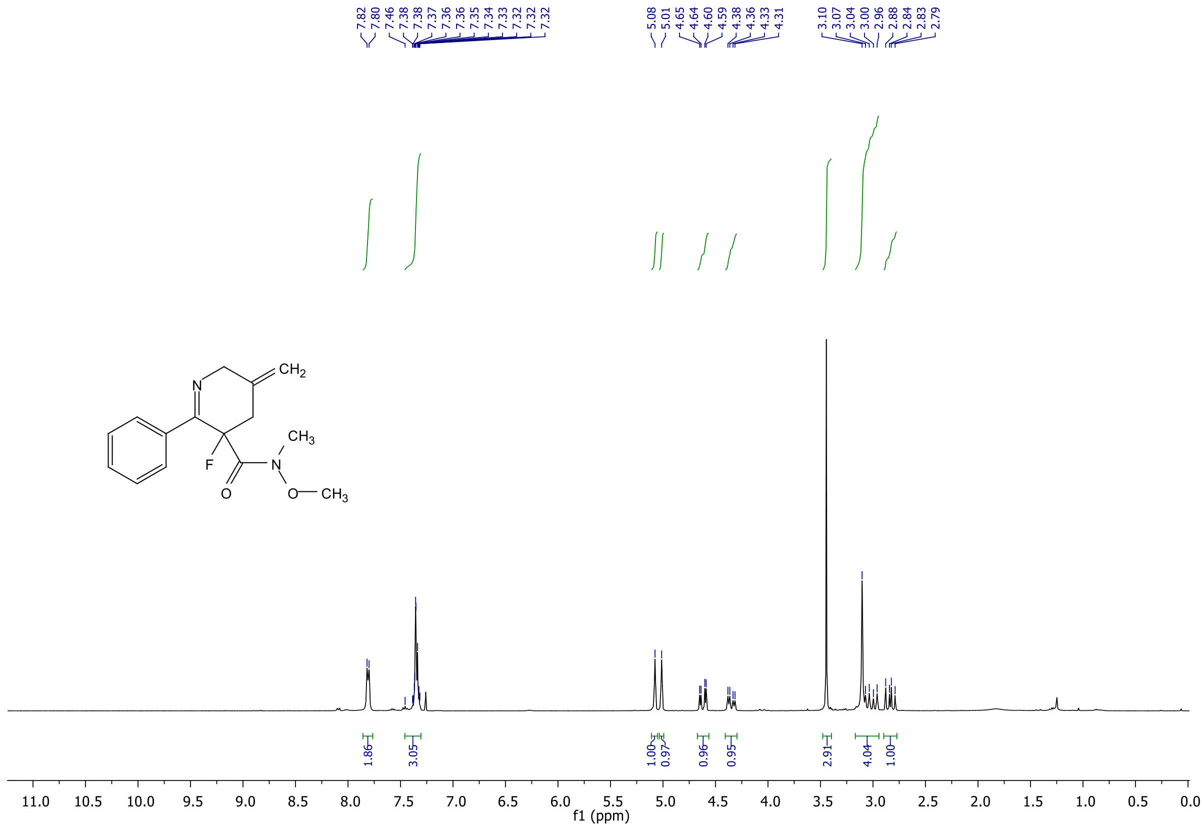

^{19}F NMR, $CDCl_3$, 377 MHz

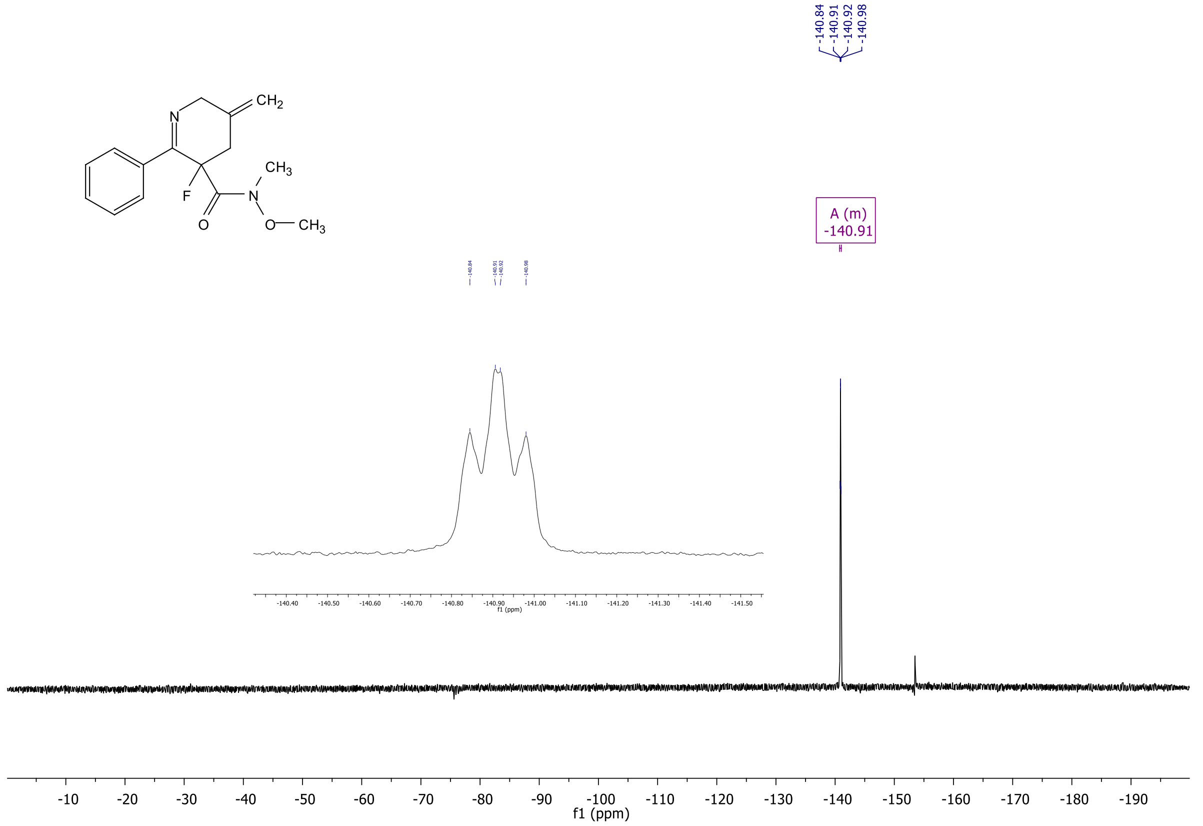

^{13}C NMR, $CDCl_3$, 101 MHz

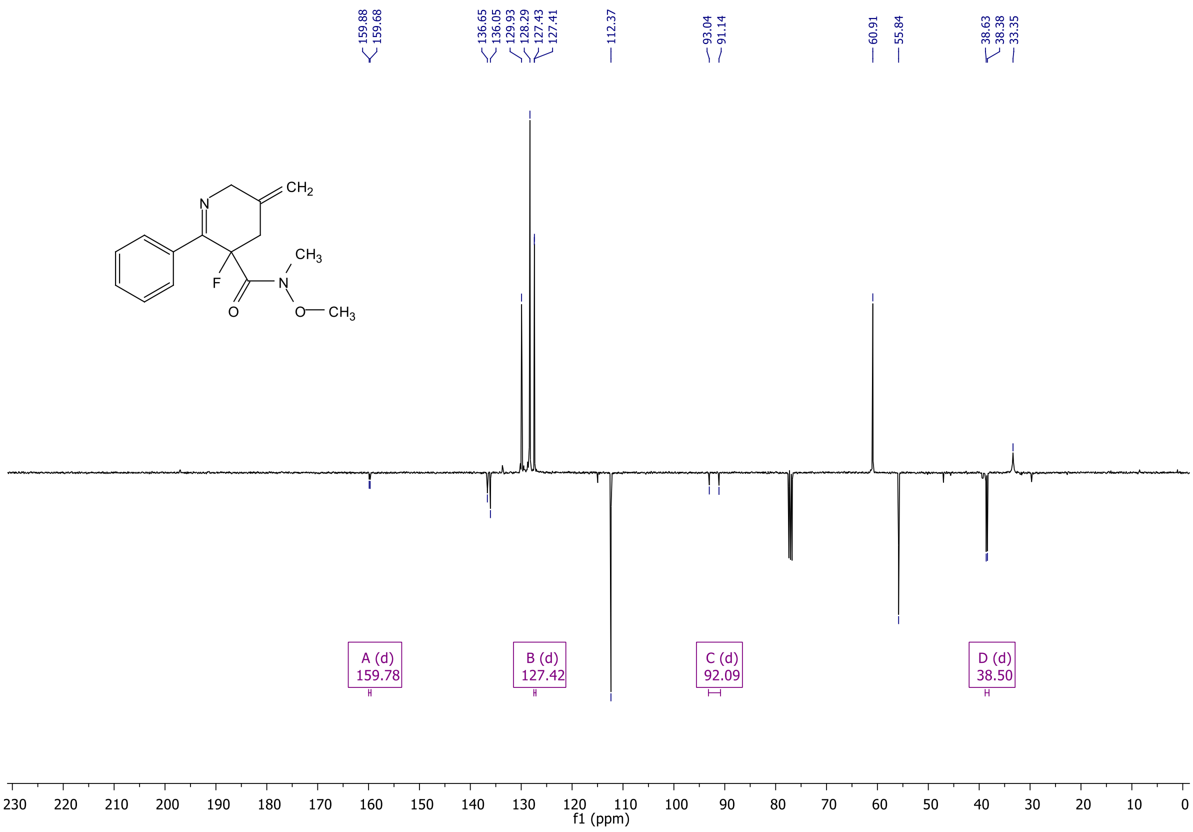

Ethyl 2-methyl-3-fluoro-5-methylidene-4,6-dihydropyridine-3-carboxylate (4m)

^{1}H NMR, $CDCl_3$, 400 MHz

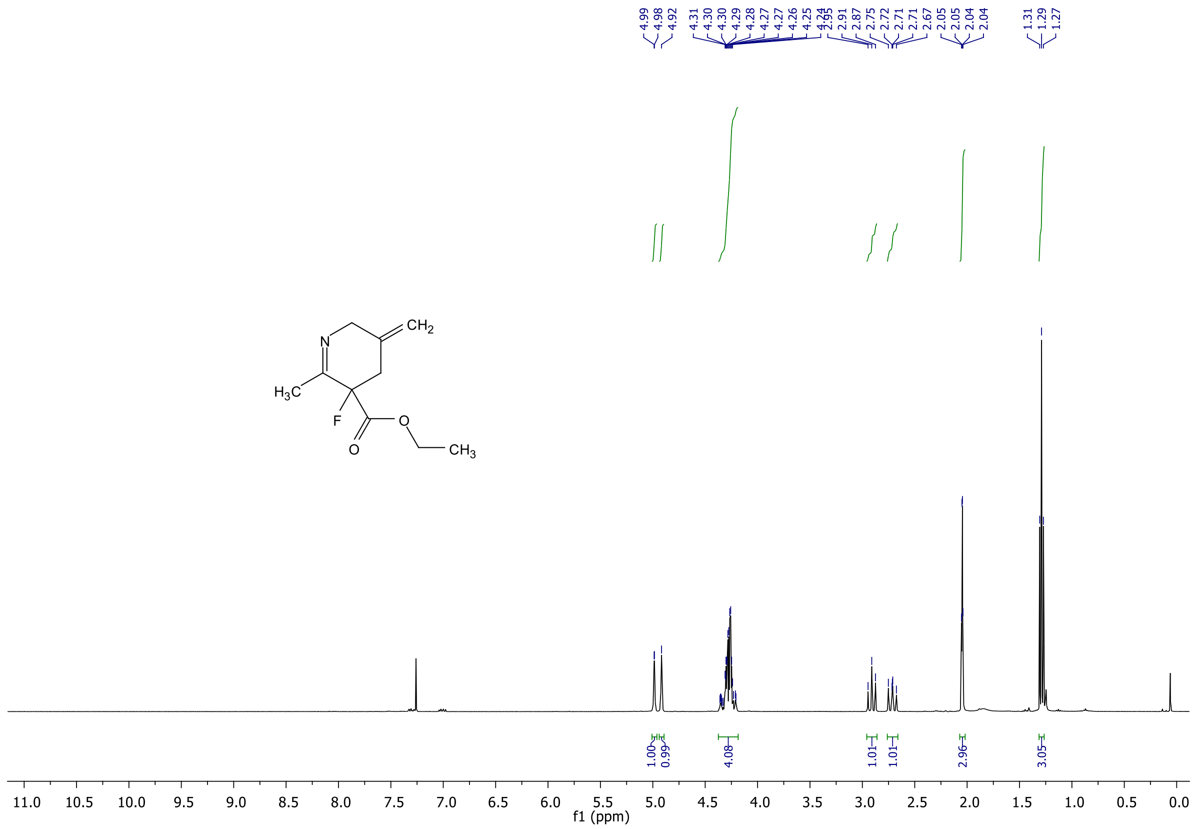

^{19}F NMR, $CDCl_3$, 377 MHz

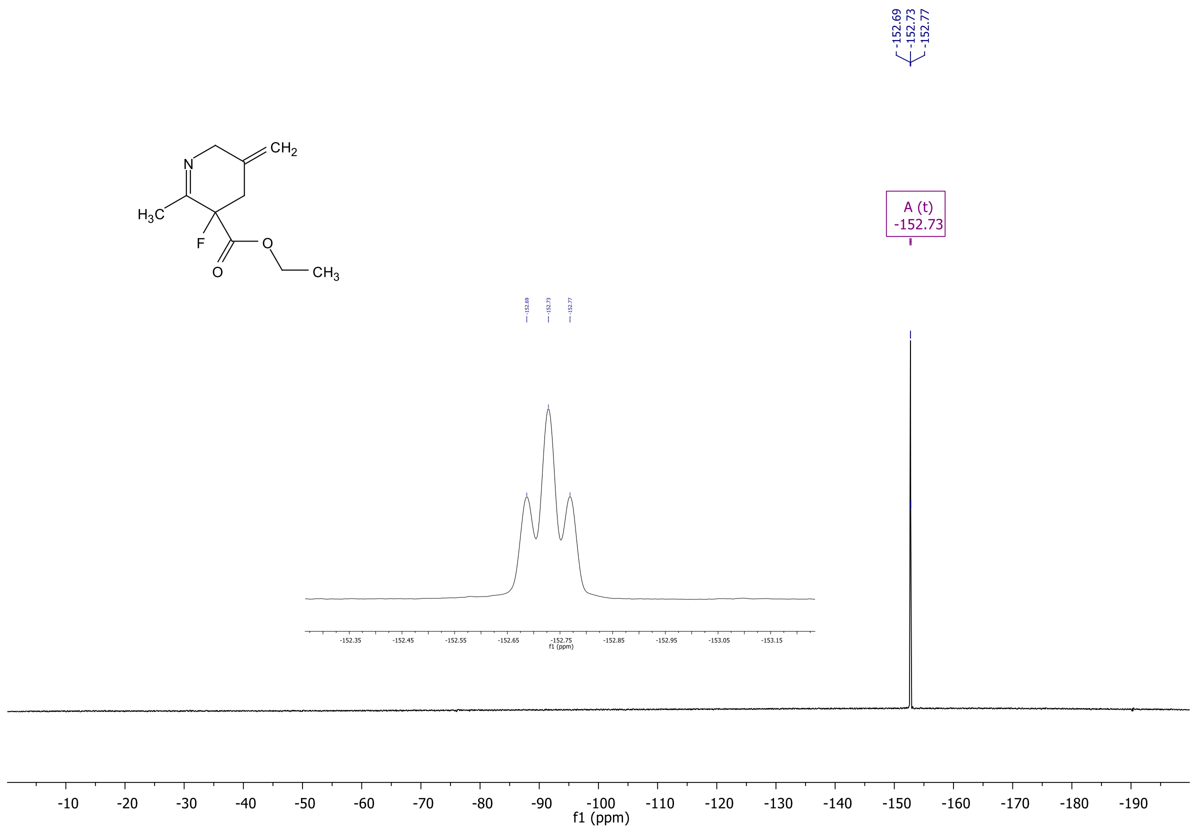

^{13}C NMR, $CDCl_3$, 101 MHz

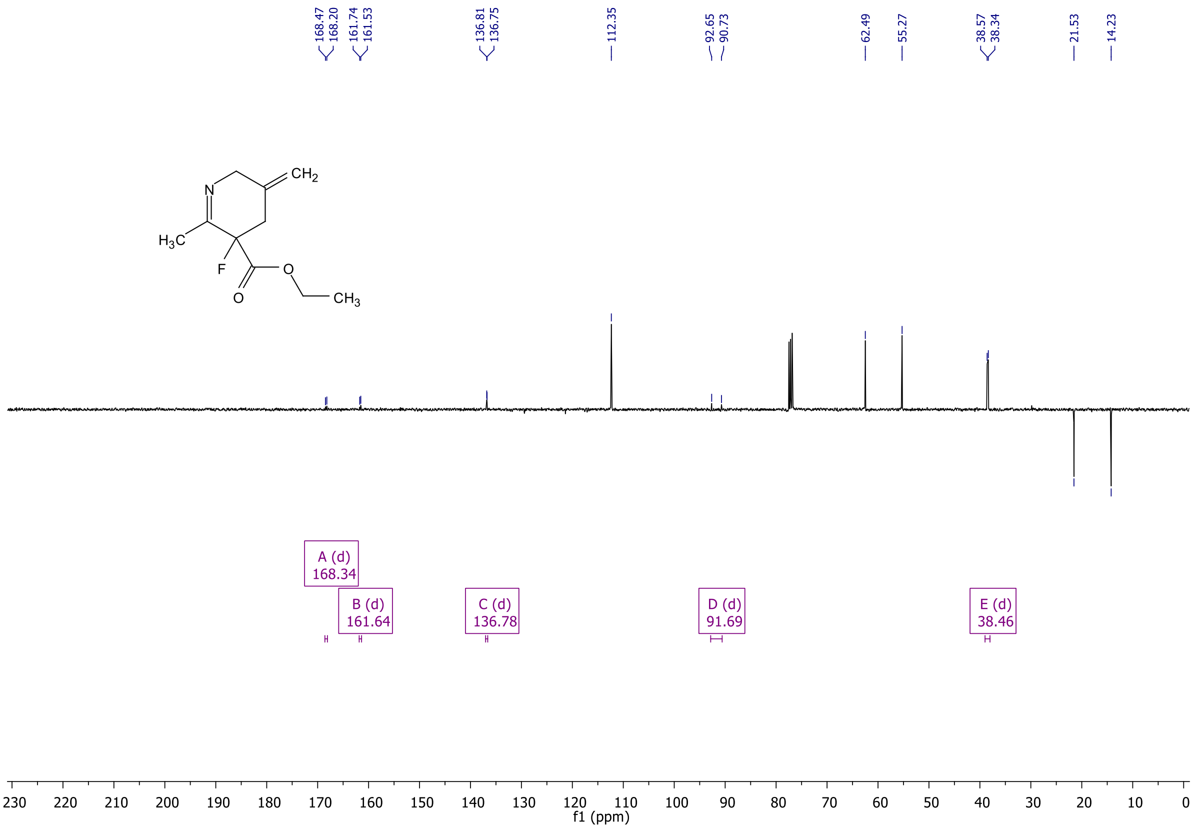

Ethyl 2-ethyl-3-fluoro-5-methylidene-4,6-dihydropyridine-3-carboxylate (4n)

^{1}H NMR, $CDCl_3$, 400 MHz

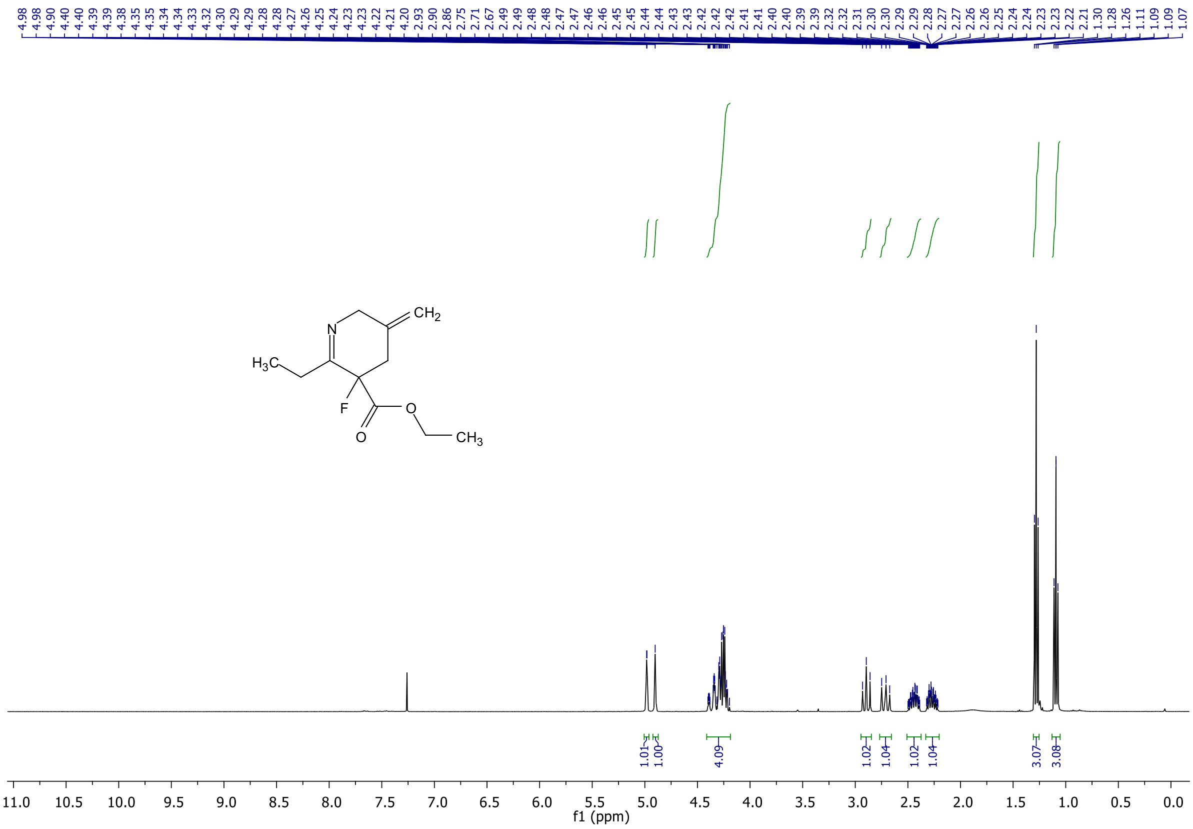

^{19}F NMR, $CDCl_3$, 377 MHz

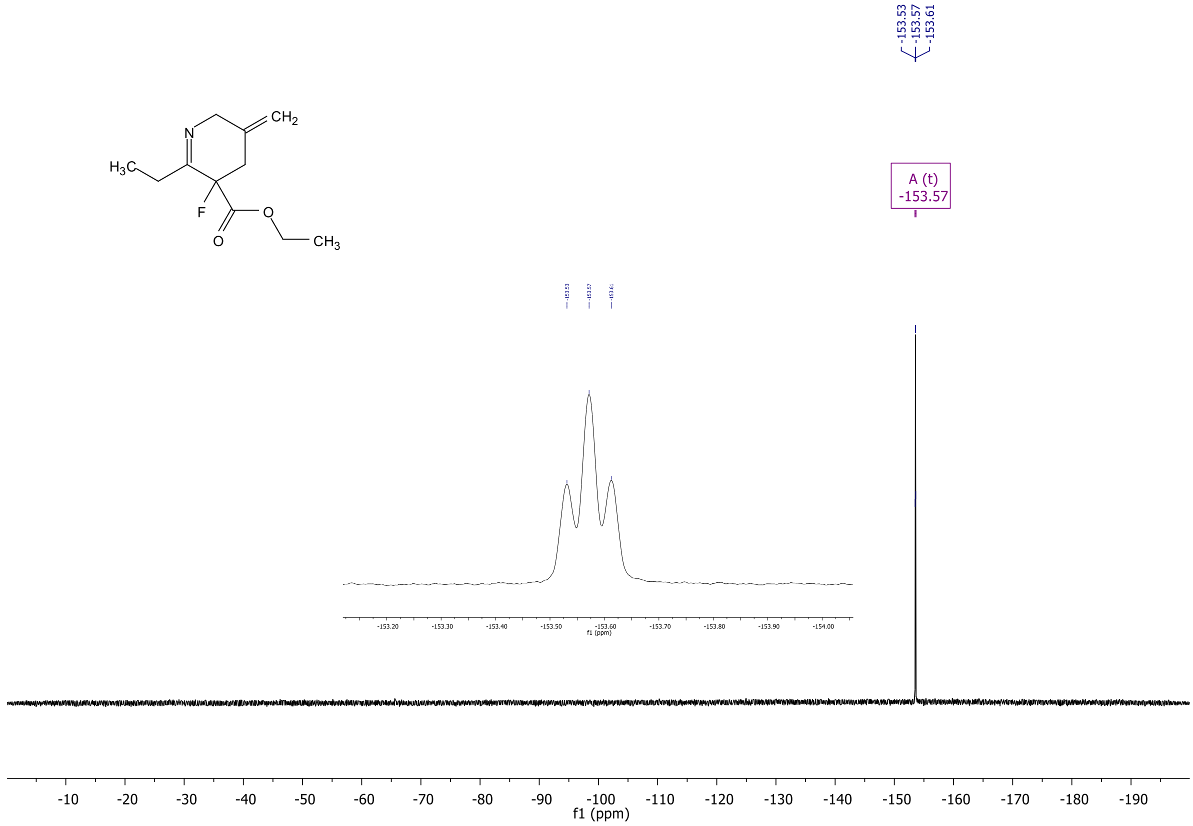

^{13}C NMR, CDCl$_3$, 101 MHz

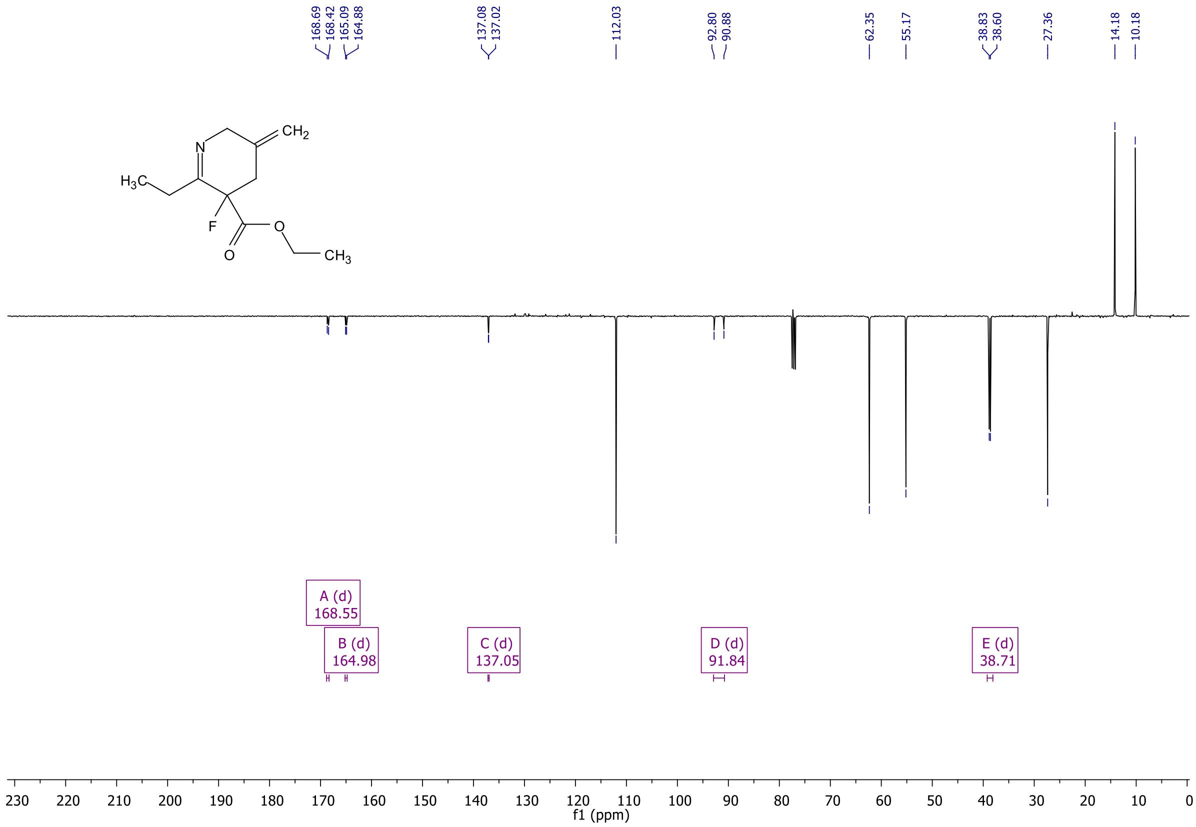

Ethyl 3-fluoro-5-methylidene-2-(propan-2-yl)-3,4,5,6-tetrahydropyridine-3-carboxylate (4o)

^{1}H NMR, CDCl$_3$, 400 MHz

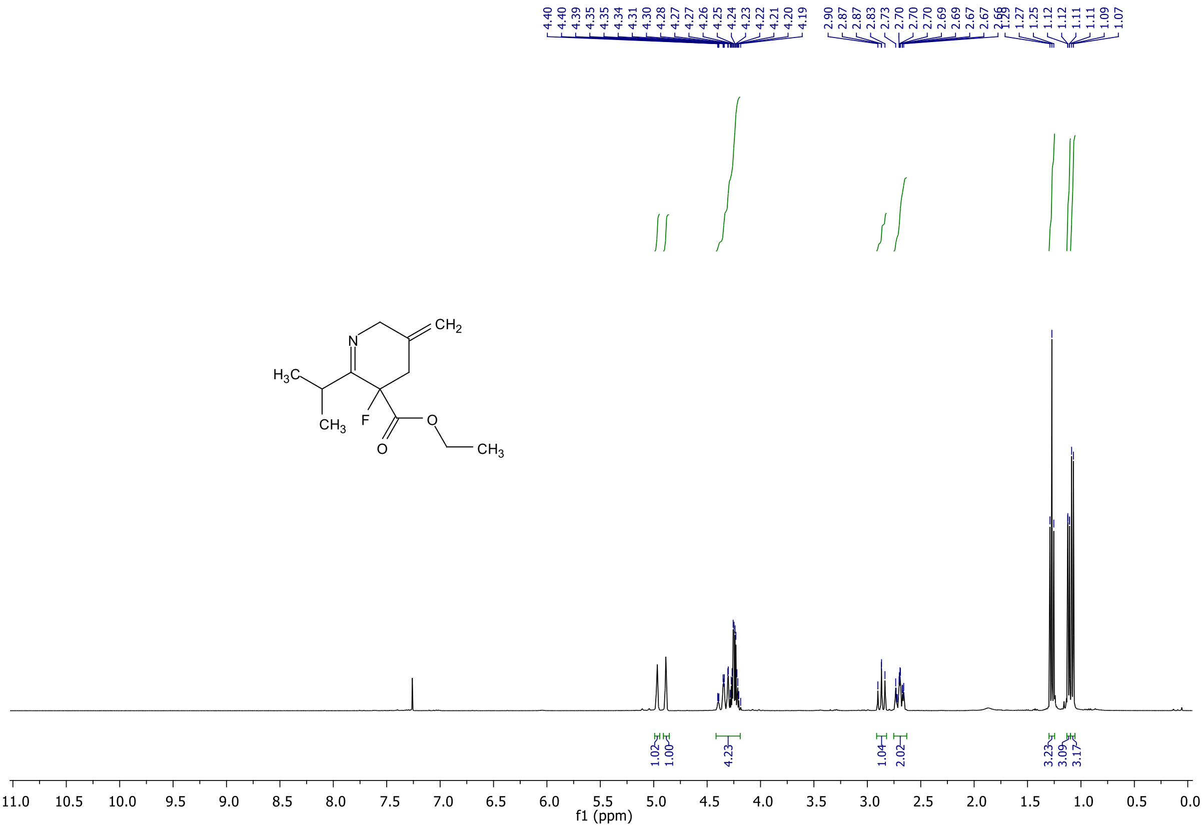

^{19}F NMR, CDCl$_3$, 377 MHz

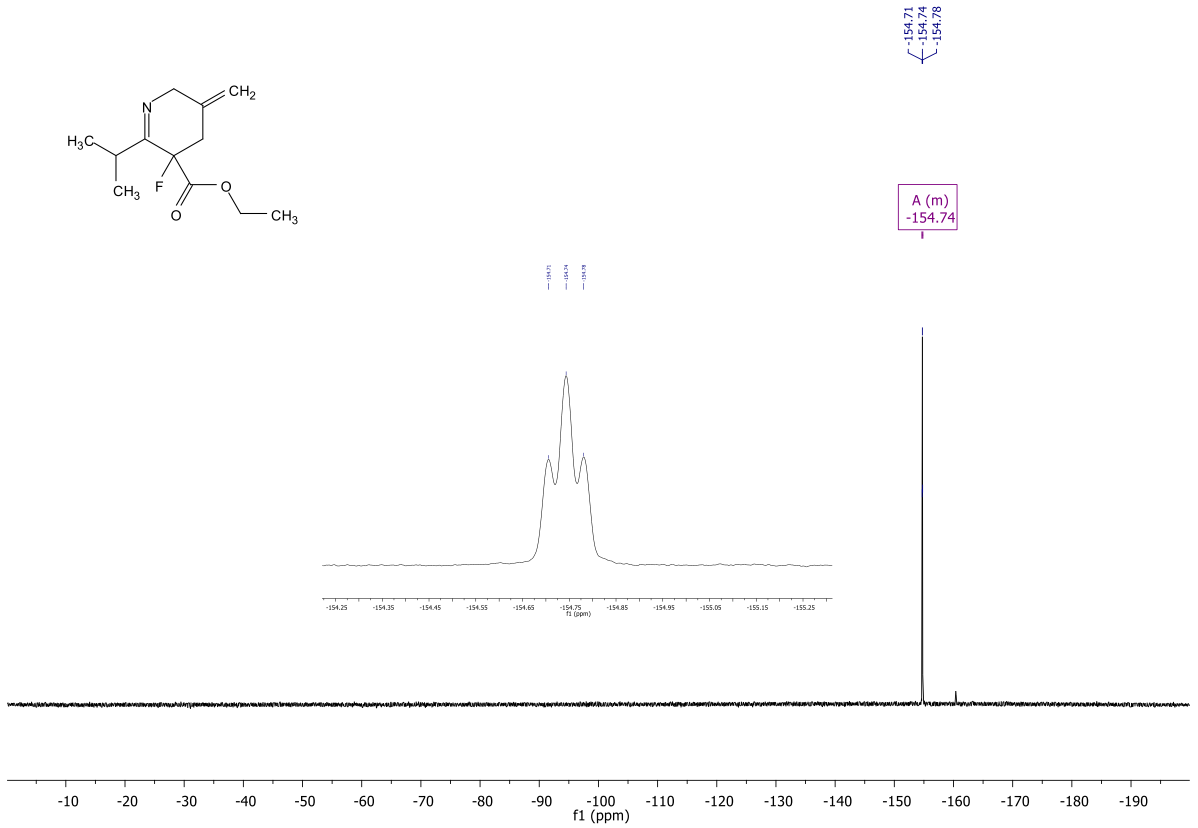

^{13}C NMR, $CDCl_3$, 101 MHz

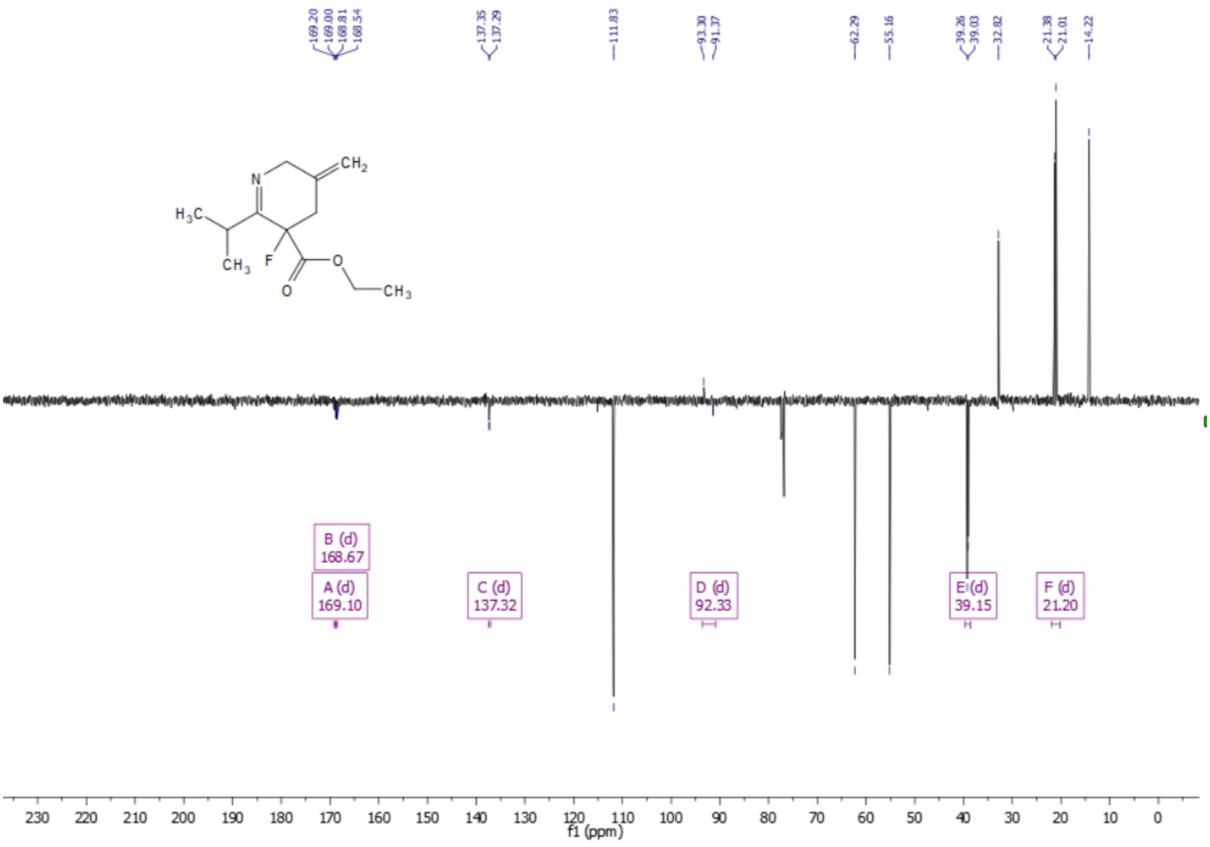

Ethyl 2-tert-butyl-3-fluoro-5-methylidene-4,6-dihydropyridine-3-carboxylate (4p)

^{1}H NMR, $CDCl_3$, 400 MHz

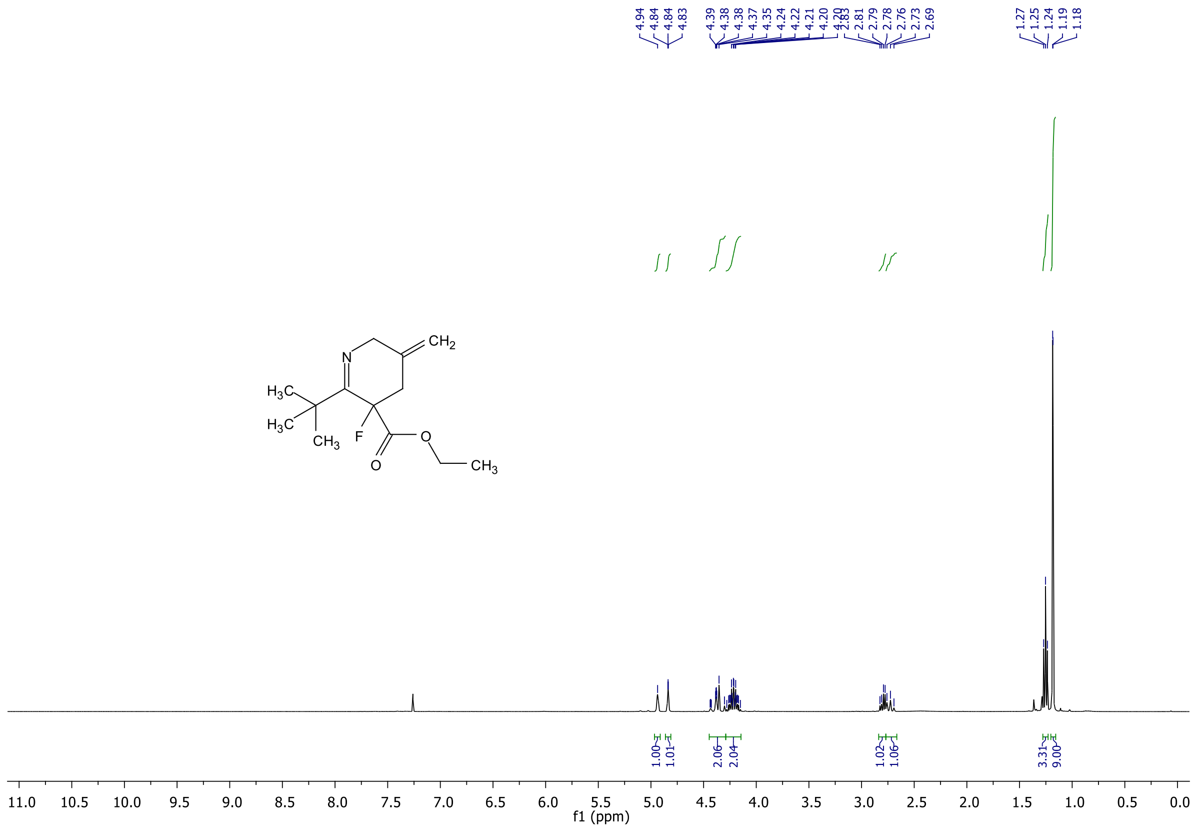

^{19}F NMR, $CDCl_3$, 377 MHz

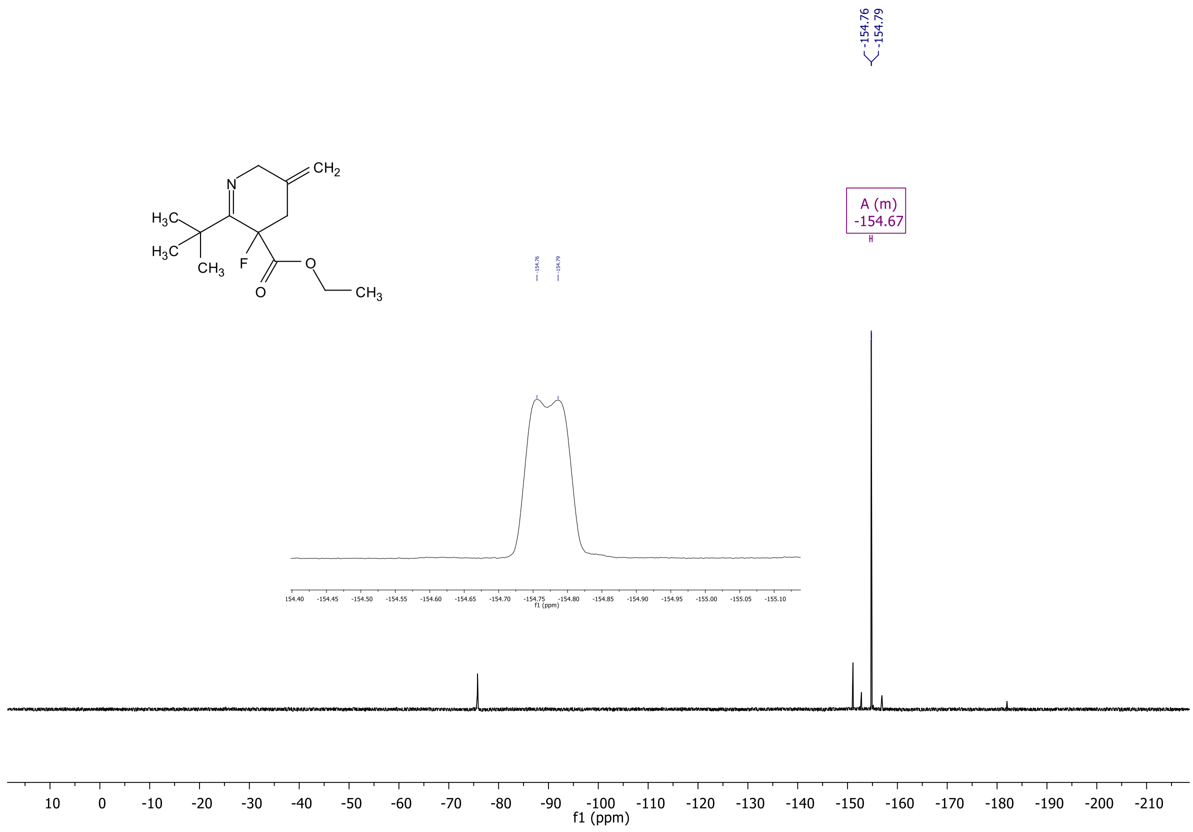

^{13}C NMR, $CDCl_3$, 101 MHz

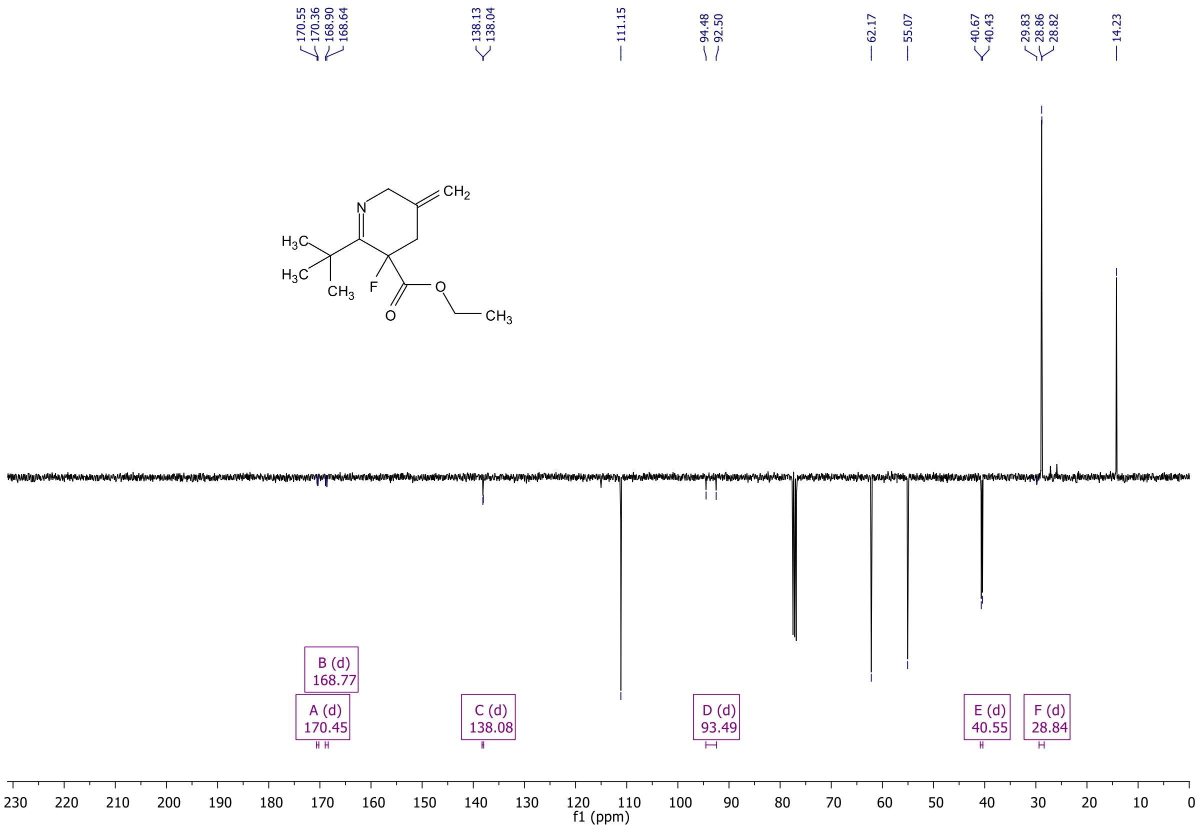

Ethyl 2-cyclohexyl-3-fluoro-5-methylidene-3,4,5,6-tetrahydropyridine-3-carboxylate (4q)

^{1}H NMR, CDCl$_3$, 400 MHz

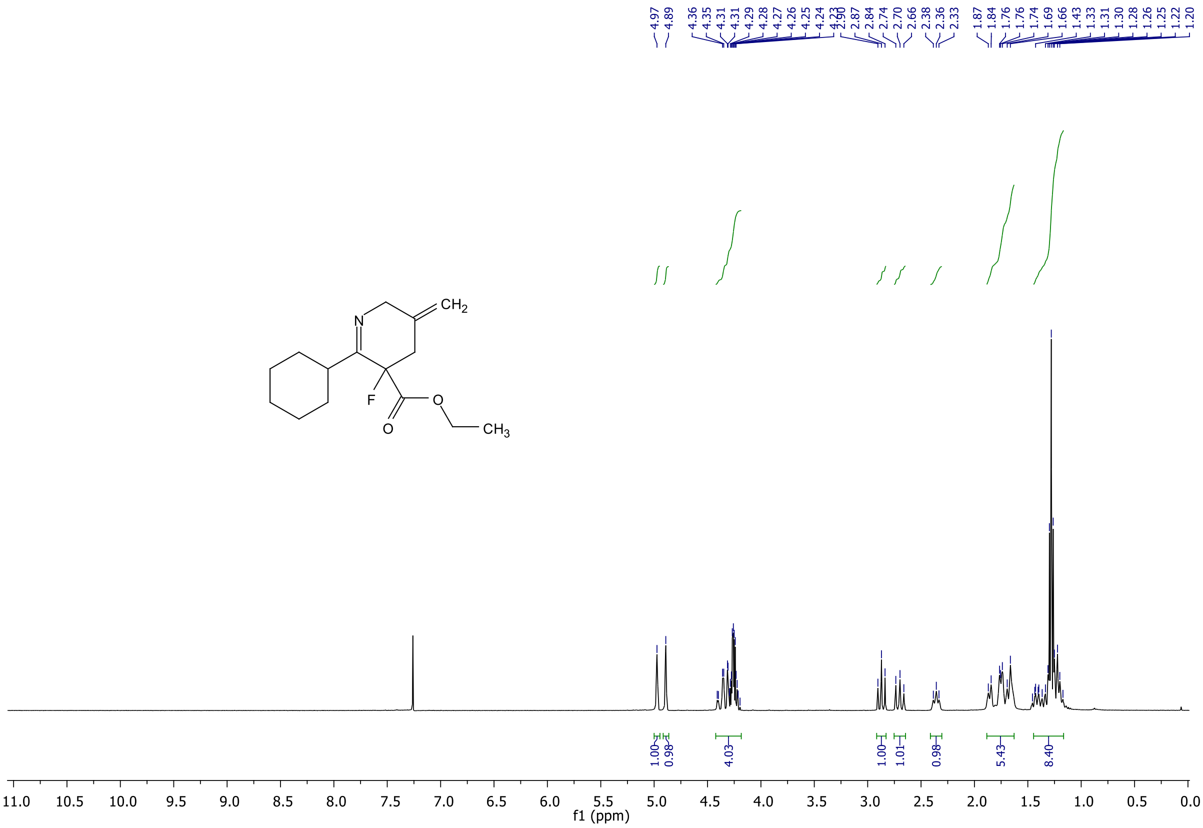

^{19}F NMR, CDCl$_3$, 377 MHz

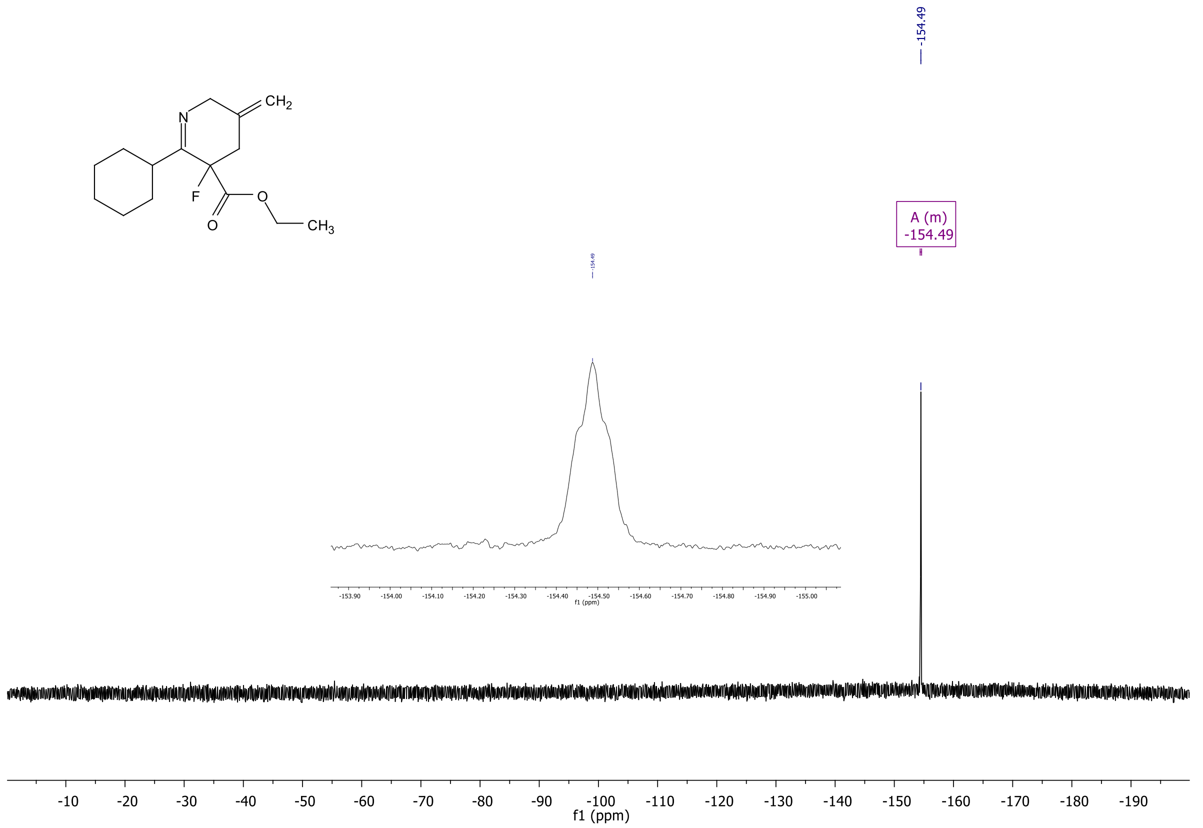

^{13}C NMR, $CDCl_3$, 101 MHz

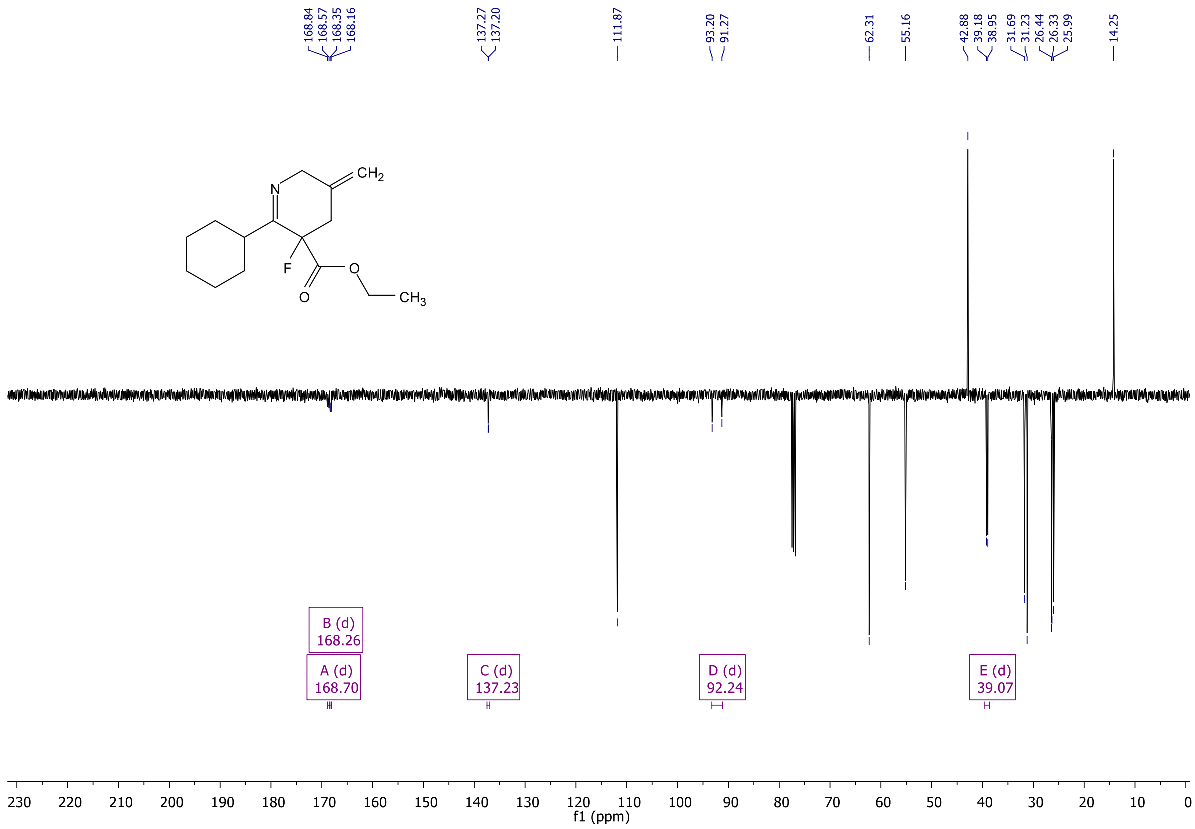

Ethyl 3-fluoro-5-methylene-2-(pent-4-en-1-yl)-3,4,5,6-tetrahydropyridine-3-carboxylate (4r)

^{1}H NMR, $CDCl_3$, 400 MHz

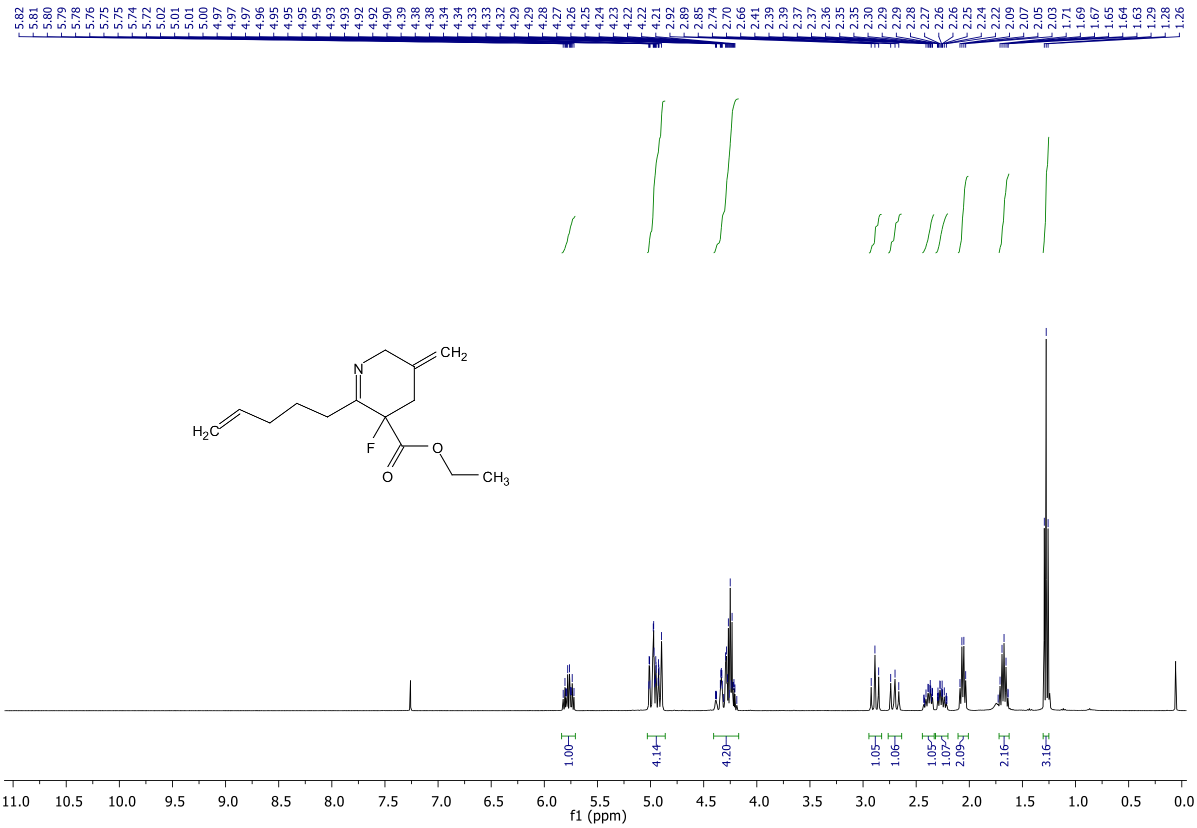

^{19}F NMR, $CDCl_3$, 377 MHz

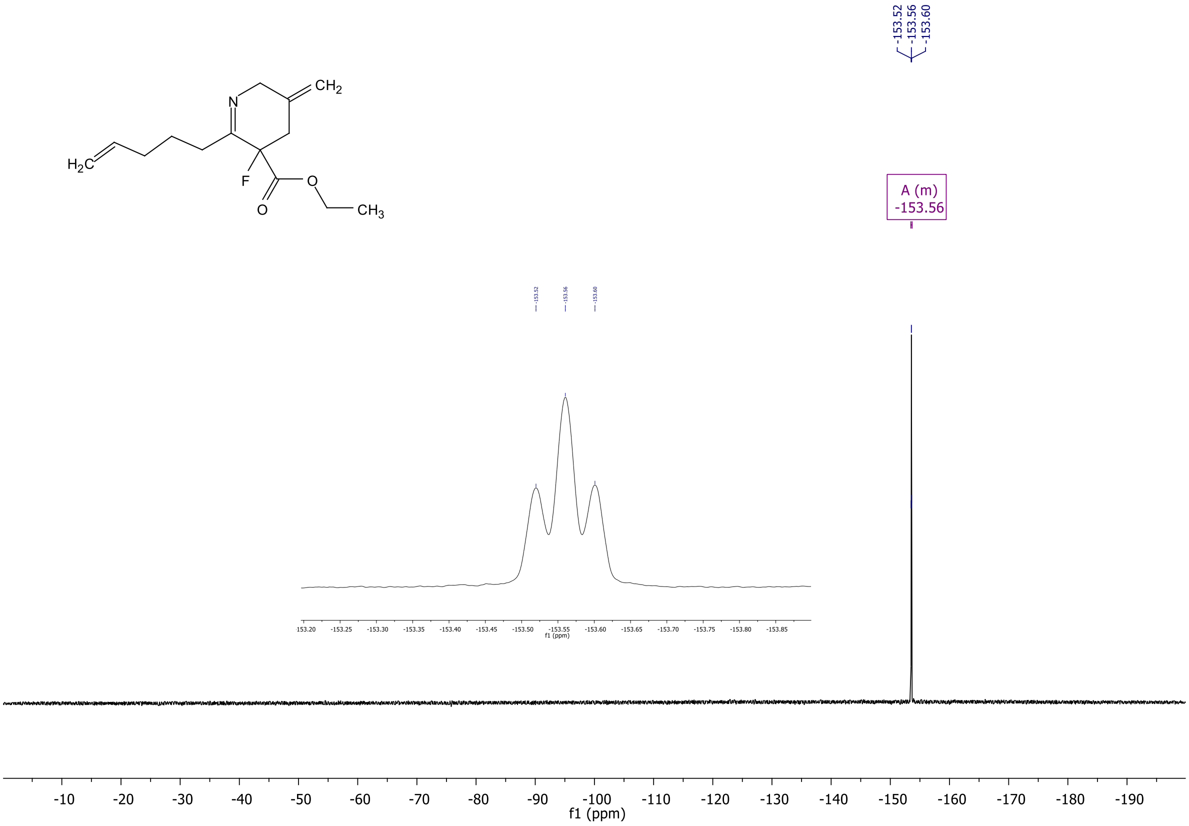

^{13}C NMR, $CDCl_3$, 101 MHz

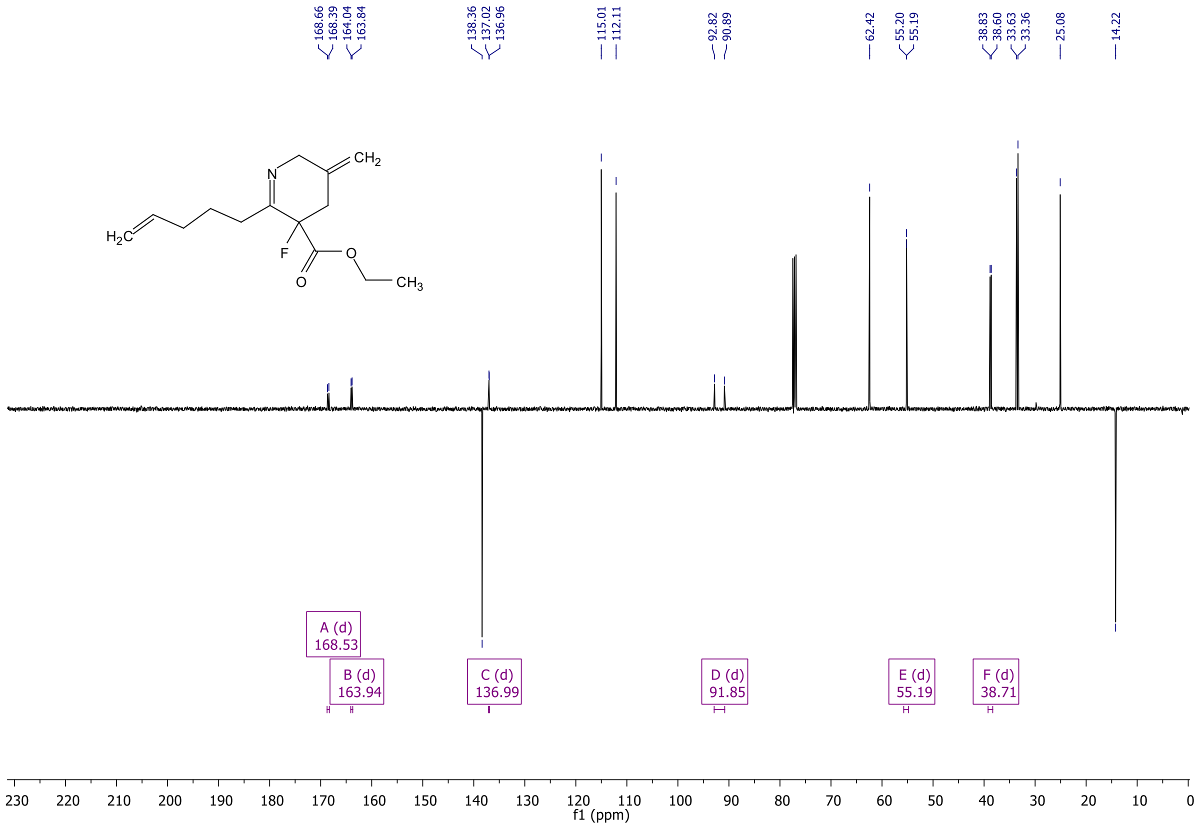

Ethyl 3-fluoro-5-methylene-2-(1-tosylpyrrolidin-2-yl)-3,4,5,6-tetrahydropyridine-3-carboxylate (4s)

^1H NMR, $CDCl_3$, 400 MHz

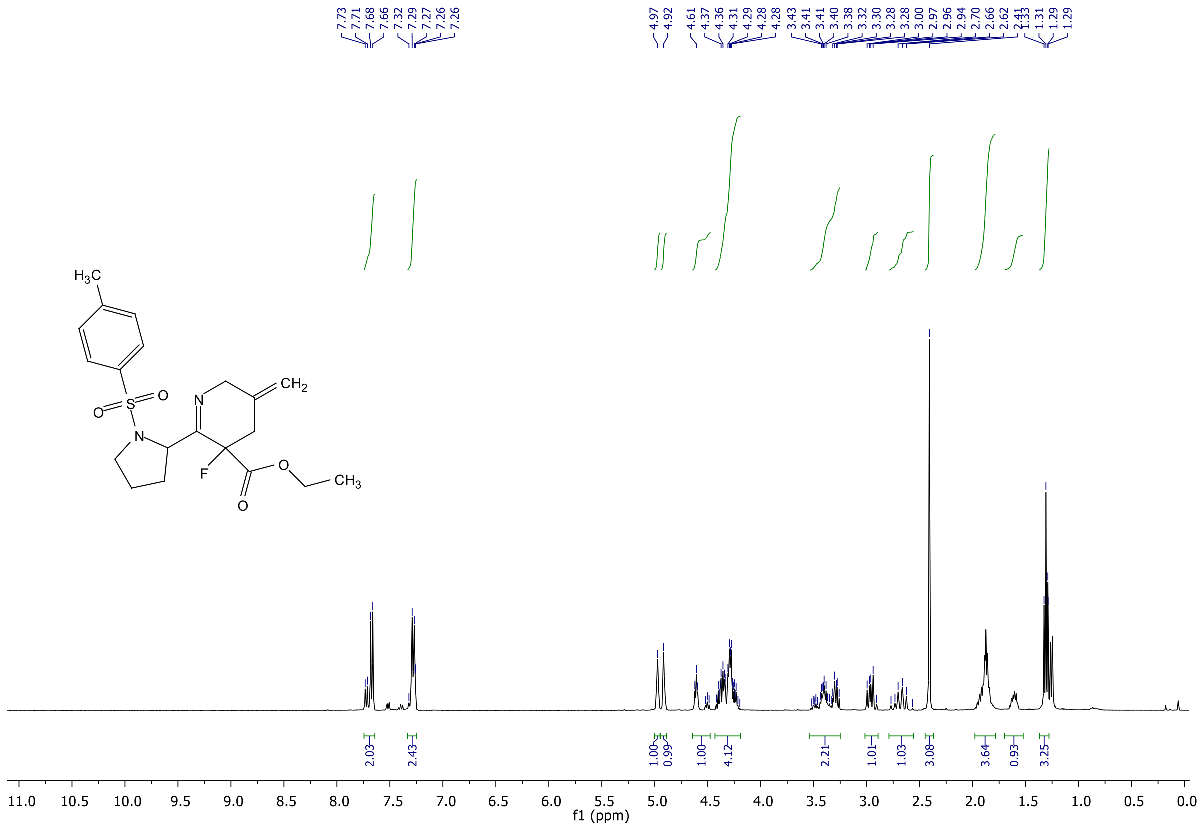

^{19}F NMR, $CDCl_3$, 377 MHz

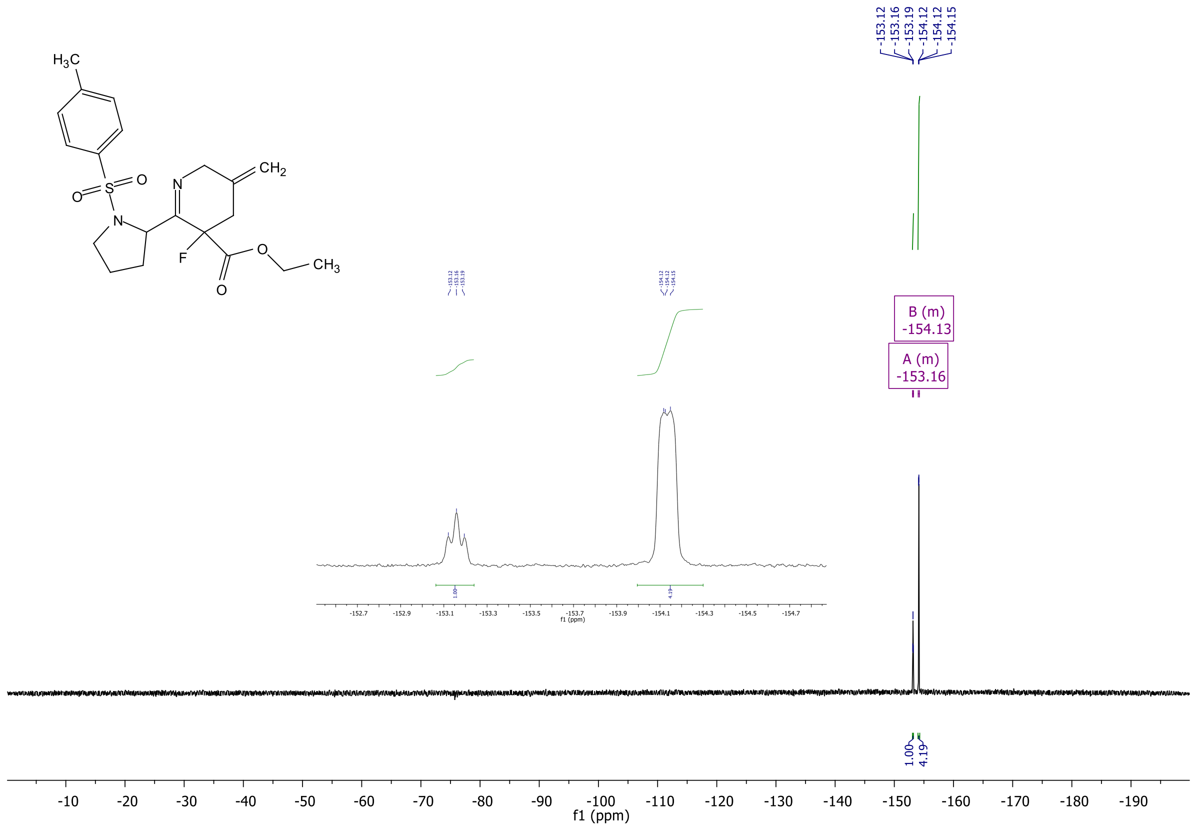

^{13}C NMR, $CDCl_3$, 101 MHz

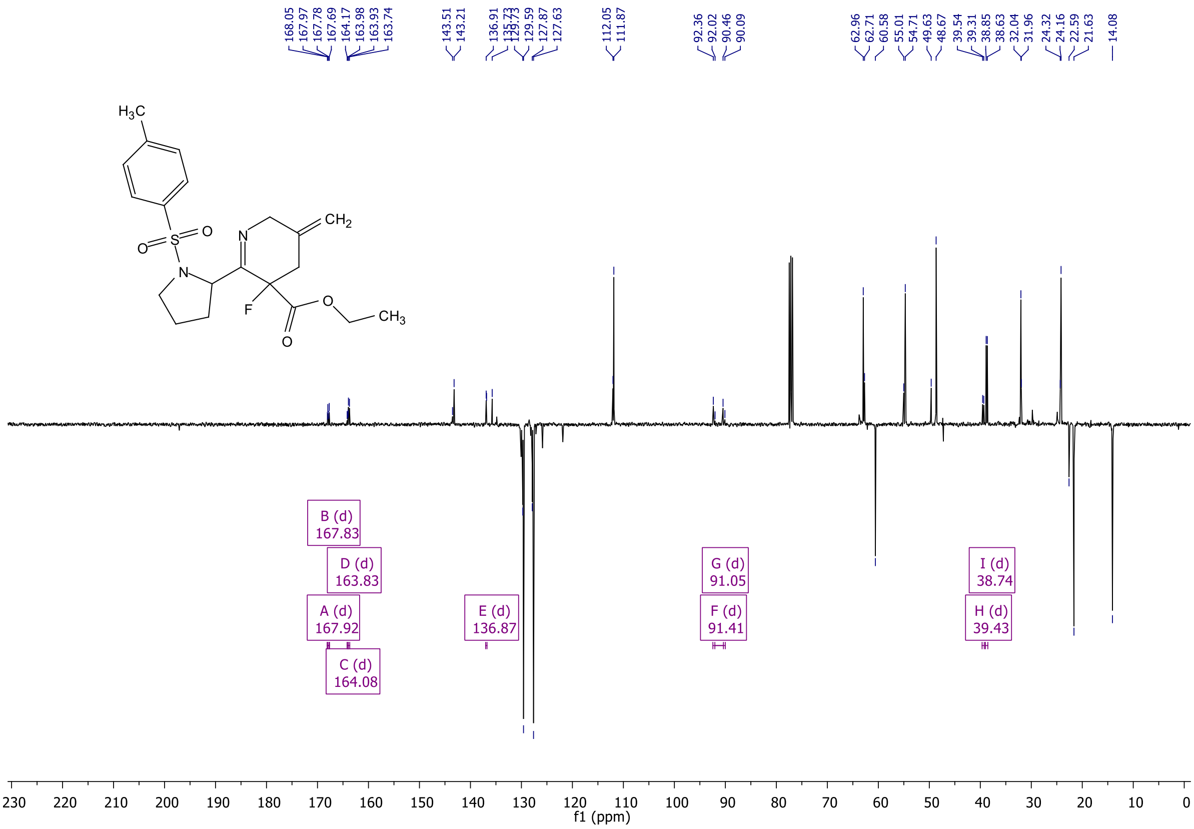

Ethyl 3-fluoro-5-methylene-2-phenylpiperidine-3-carboxylate (5)

^{1}H NMR, $CDCl_3$, 400 MHz

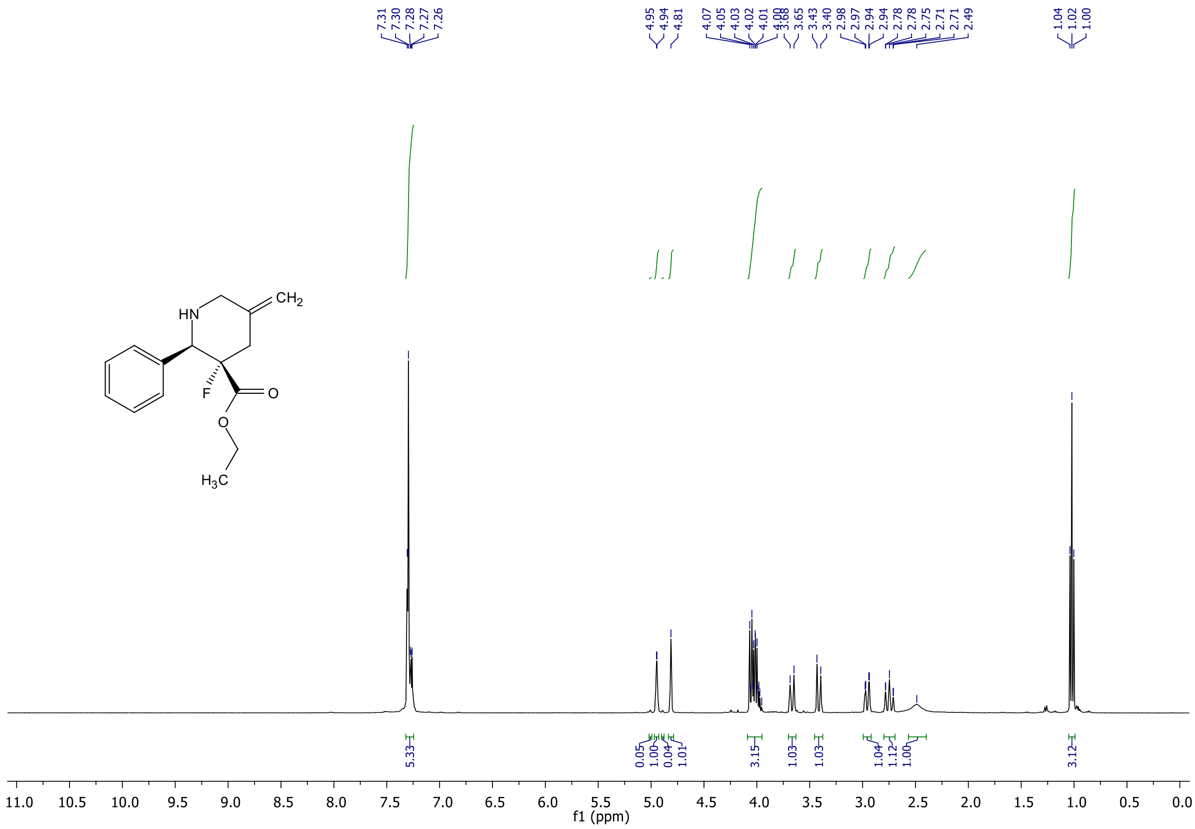

^{19}F NMR, $CDCl_3$, 377 MHz

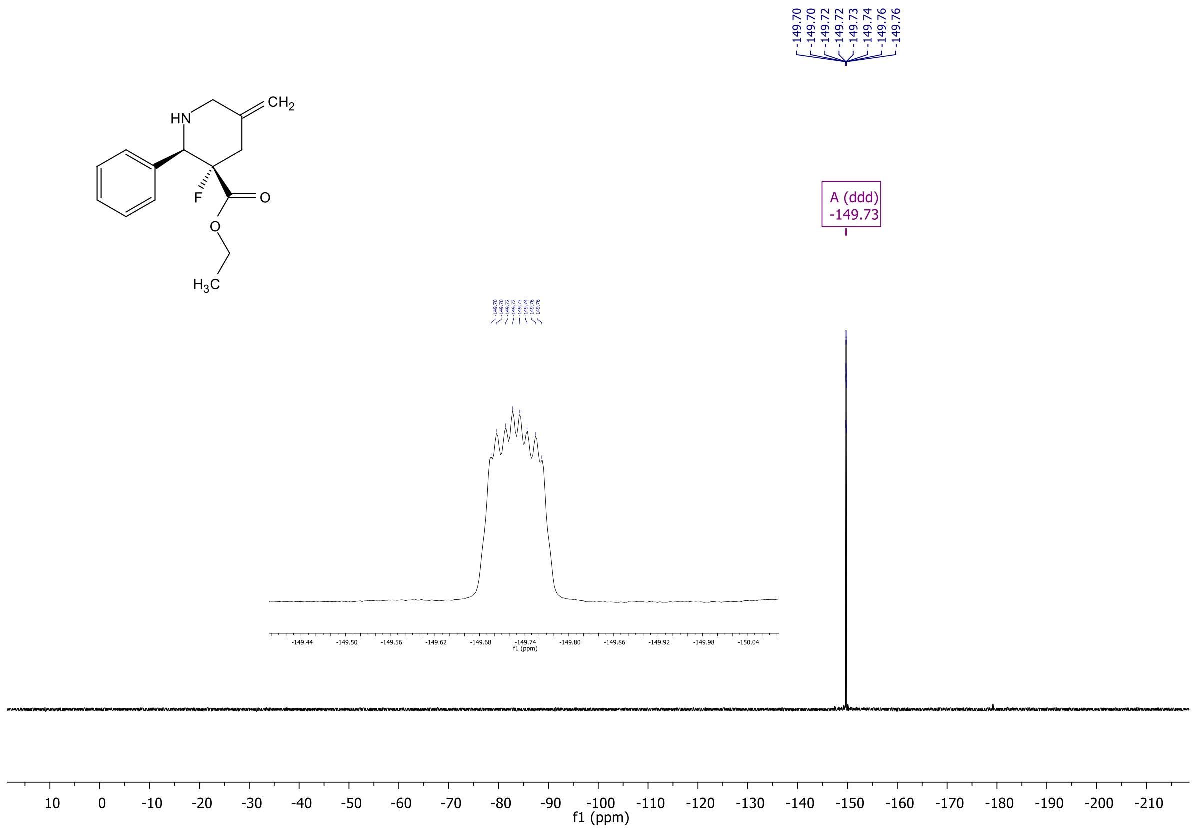

^{13}C NMR, $CDCl_3$, 101 MHz

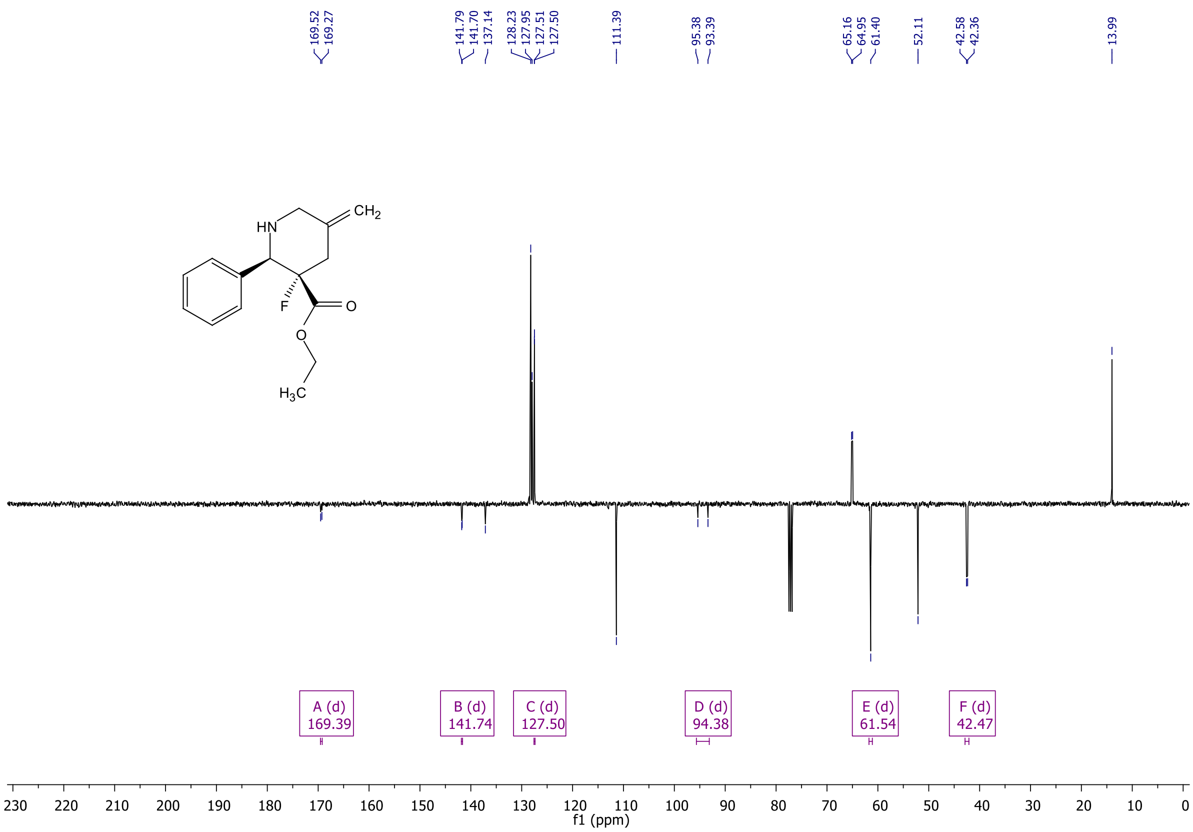

1-*tert*-Butyl 3-ethyl 3-fluoro-5-methylene-2-phenylpiperidine-1,3-dicarboxylate (6)

^{1}H NMR, $CDCl_3$, 400 MHz

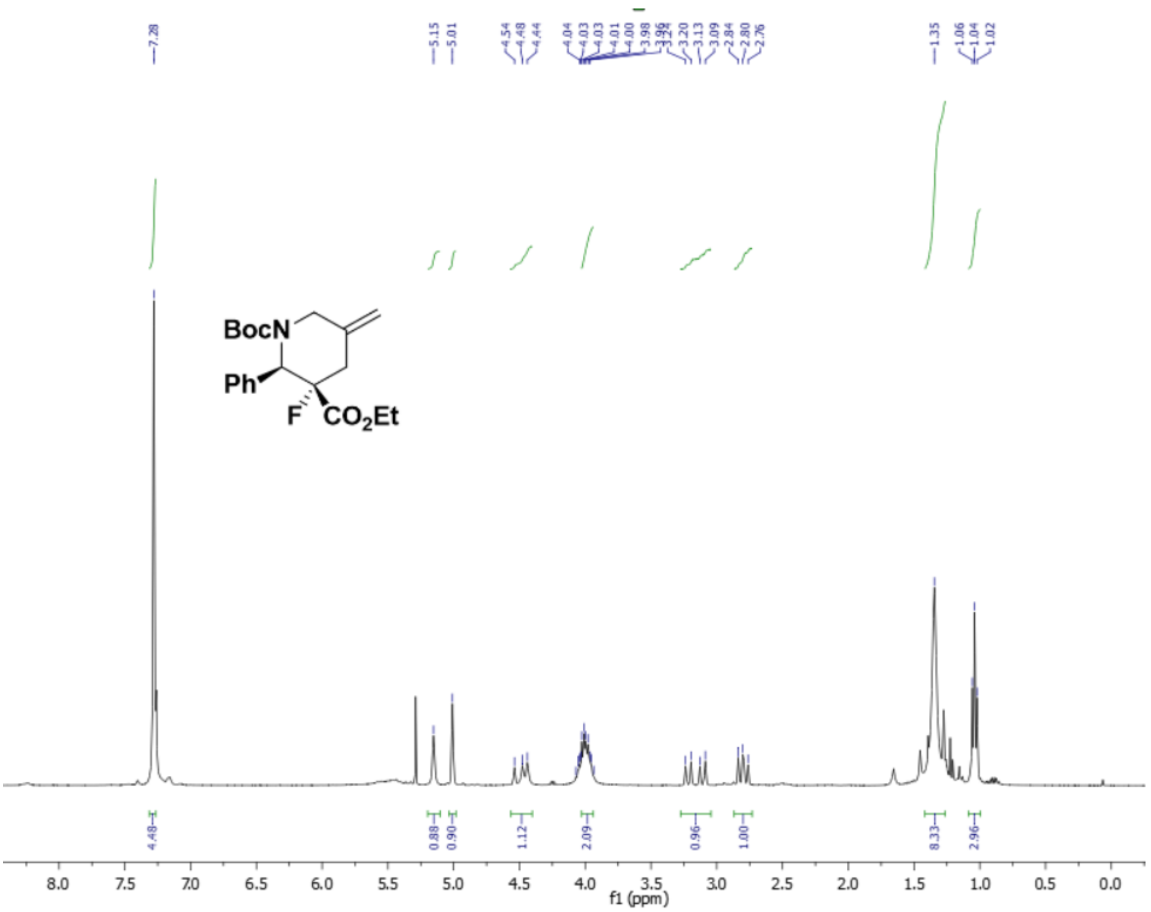

^{19}F NMR, $CDCl_3$, 377 MHz

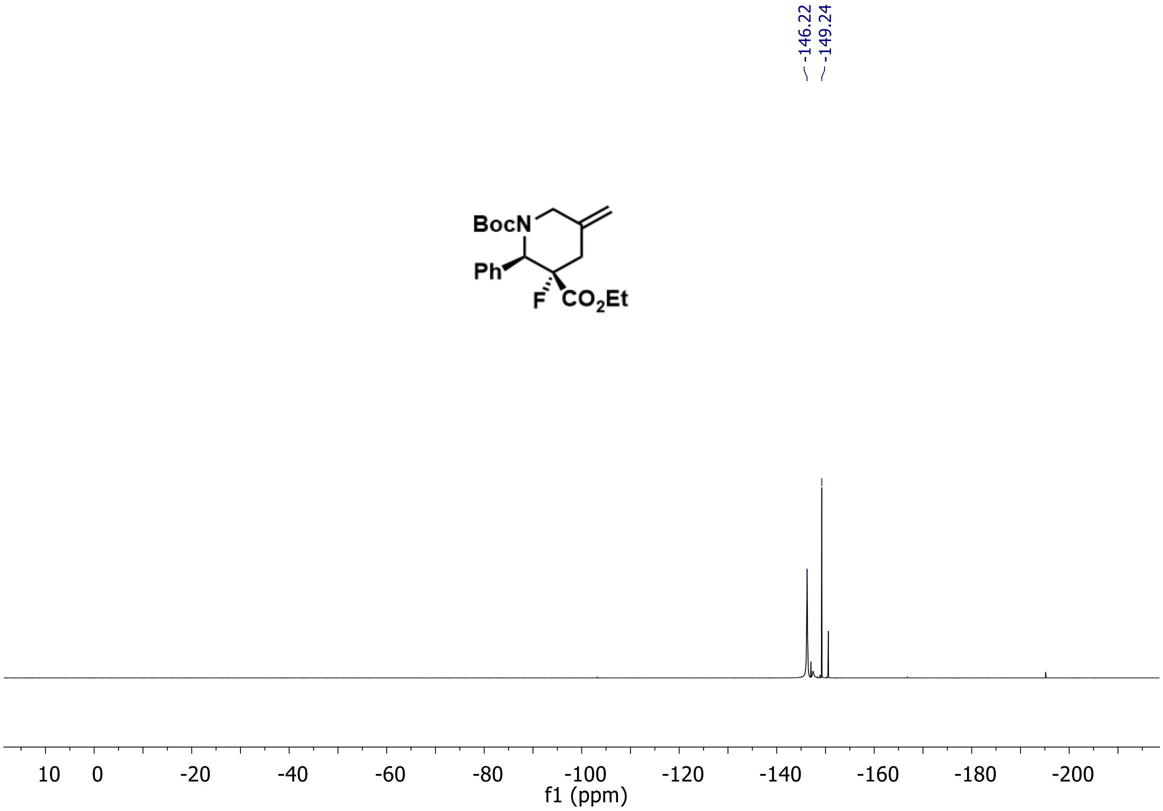

^{13}C NMR, $CDCl_3$, 101 MHz

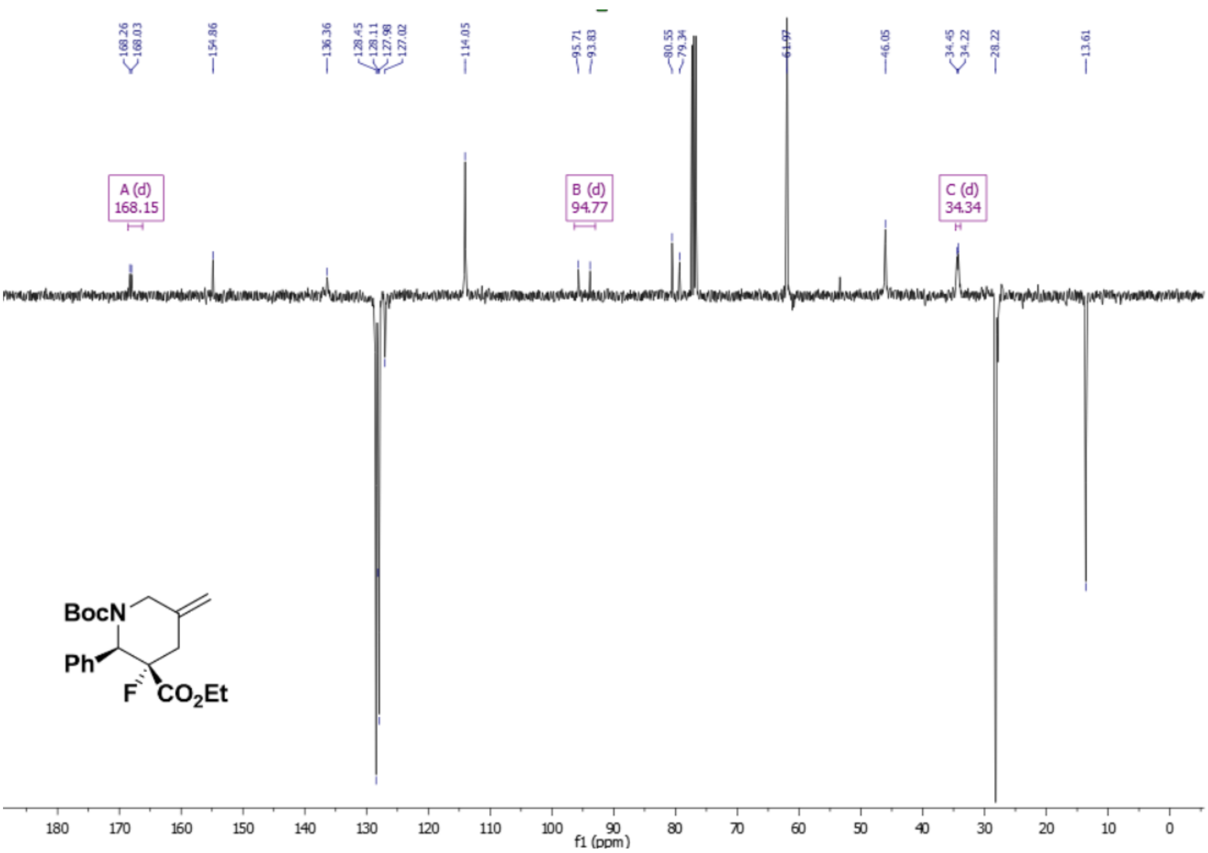

(3-Fluoro-5-methylene-2-phenyl-3,4,5,6-tetrahydropyridin-3-yl)methanol (7)

^{1}H NMR, $CDCl_3$, 400 MHz

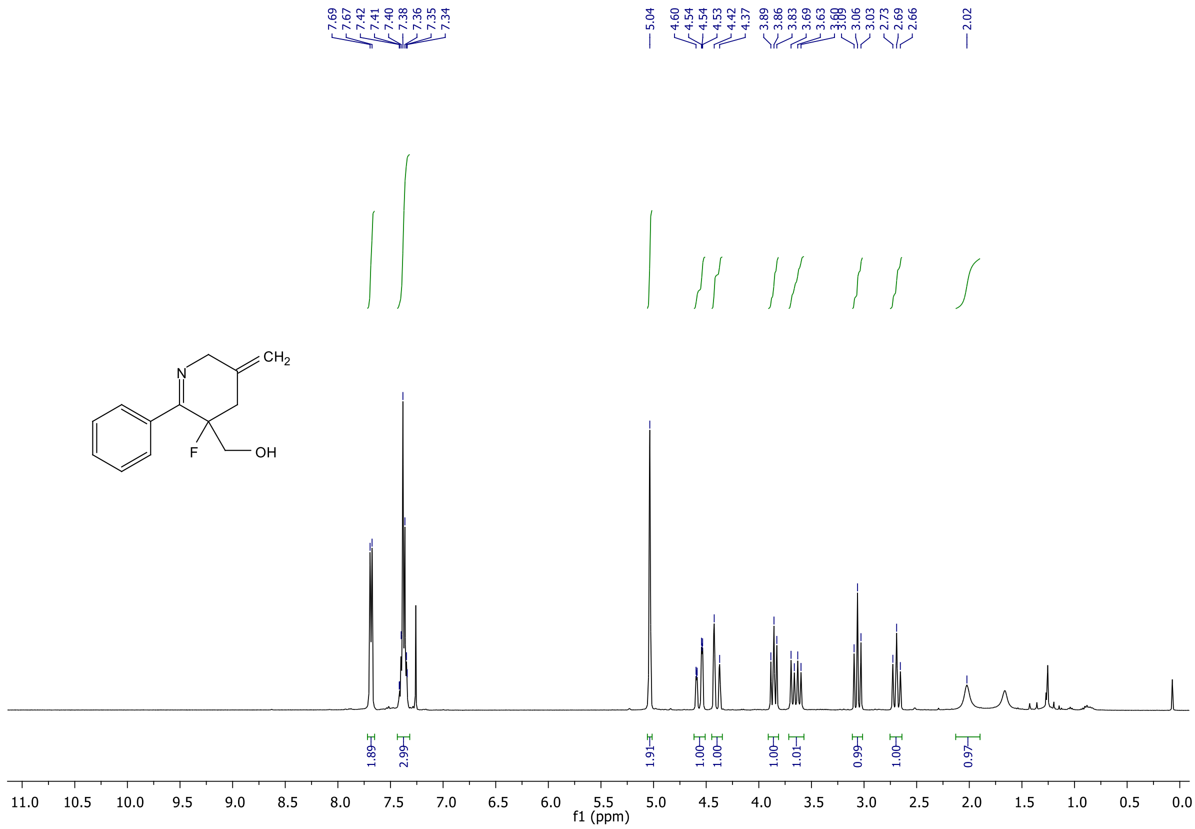

^{19}F NMR, $CDCl_3$, 377 MHz

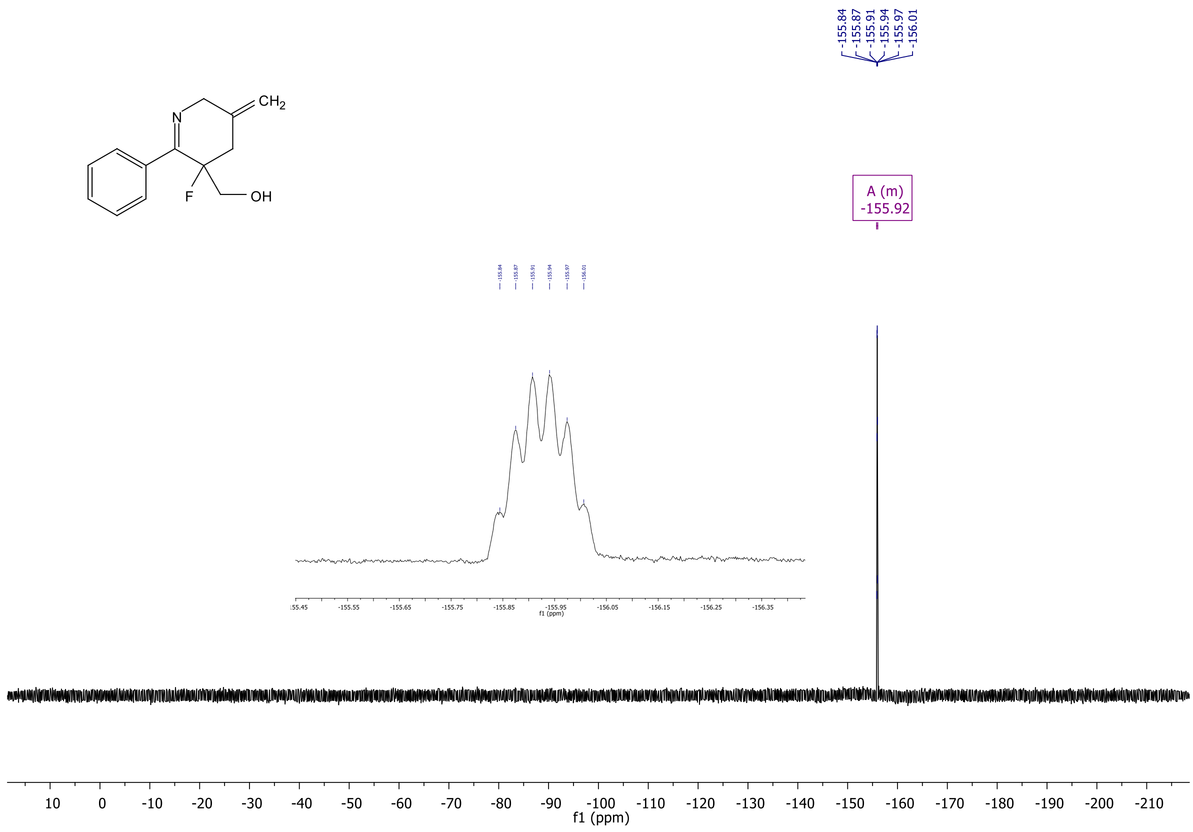

^{13}C NMR, $CDCl_3$, 101 MHz

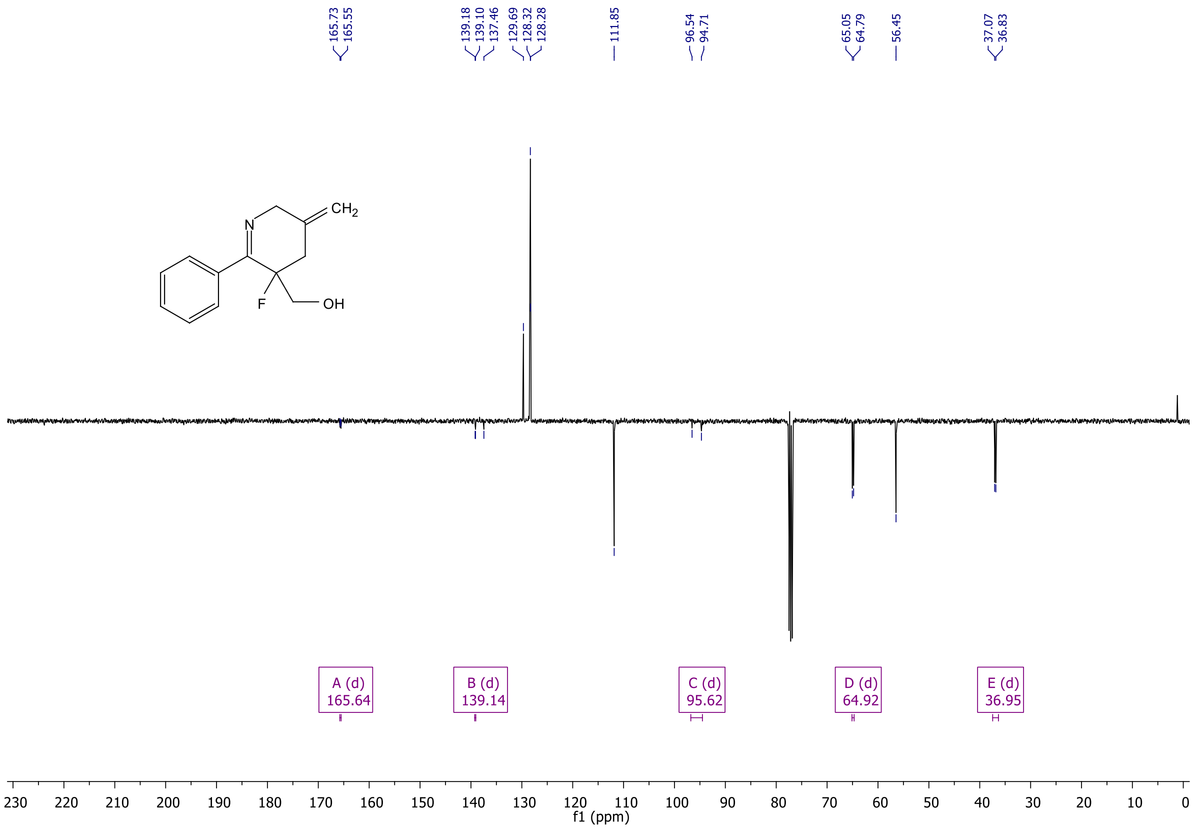

5-Fluoro-3-methylene-6-phenyl-2,3,4,5-tetrahydropyridine (8)

^{1}H NMR, CDCl$_3$, 400 MHz

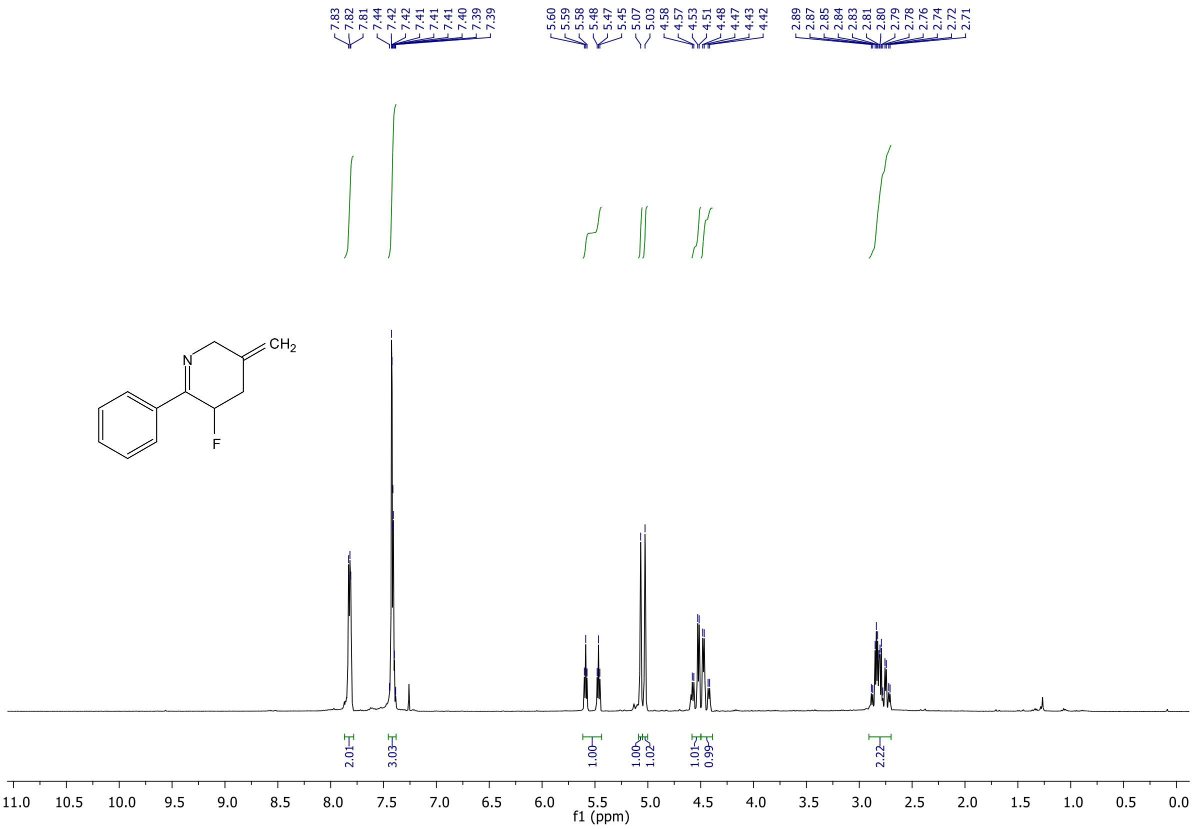

^{19}F NMR, CDCl$_3$, 377 MHz

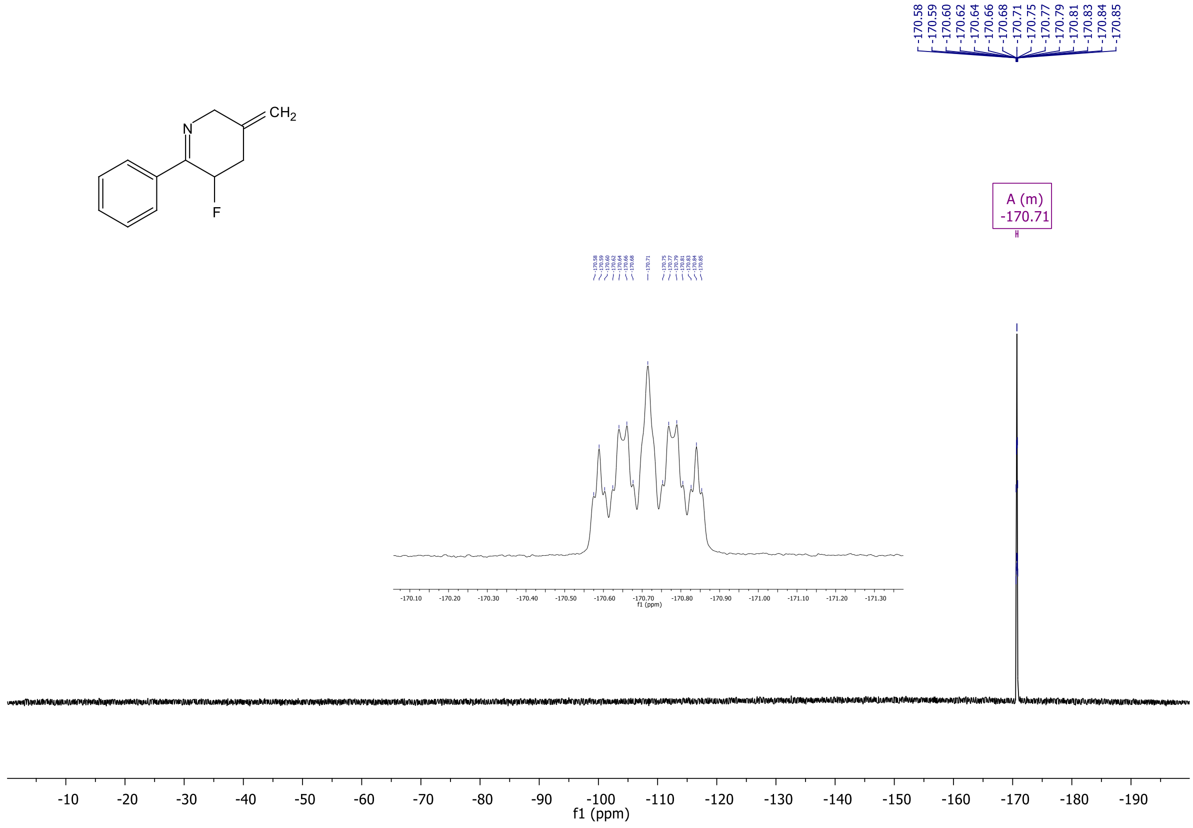

^{13}C NMR, $CDCl_3$, 101 MHz

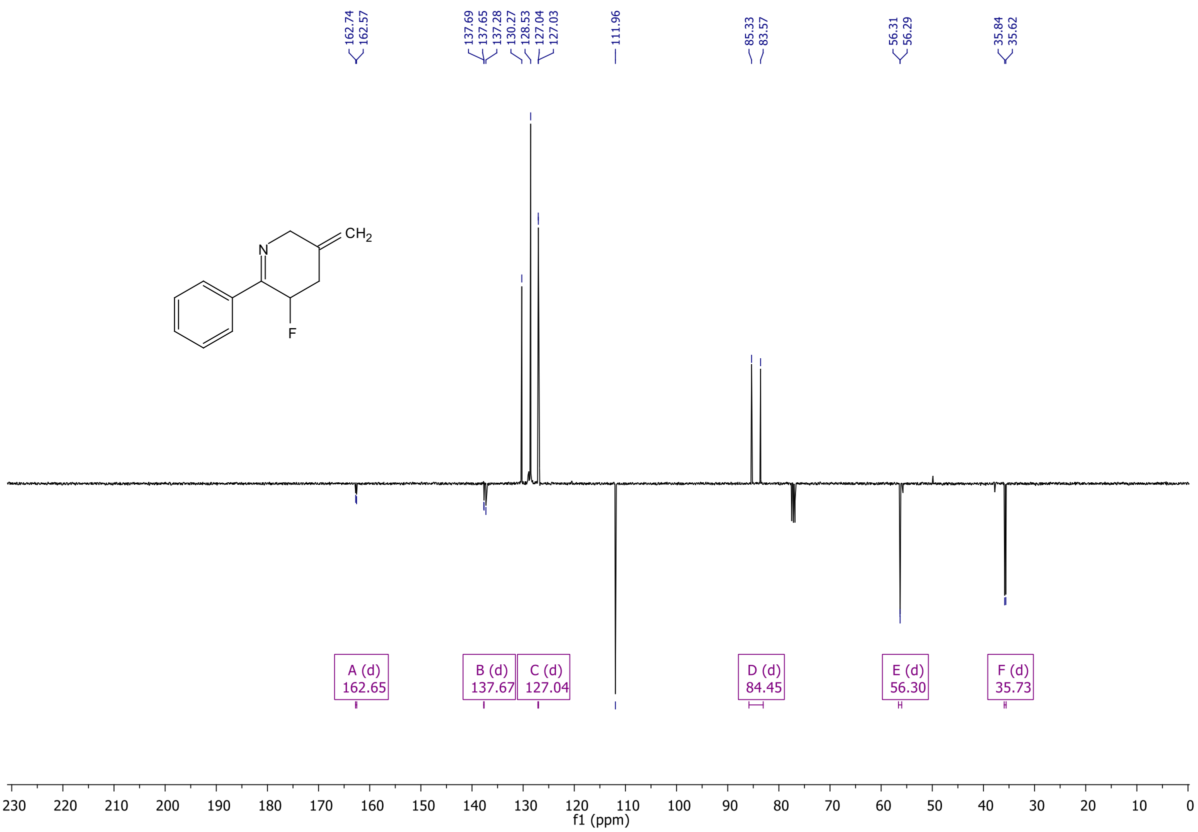

3-Fluoro-5-methylene-2-phenylpiperidine (9)

^{1}H NMR, $CDCl_3$, 400 MHz

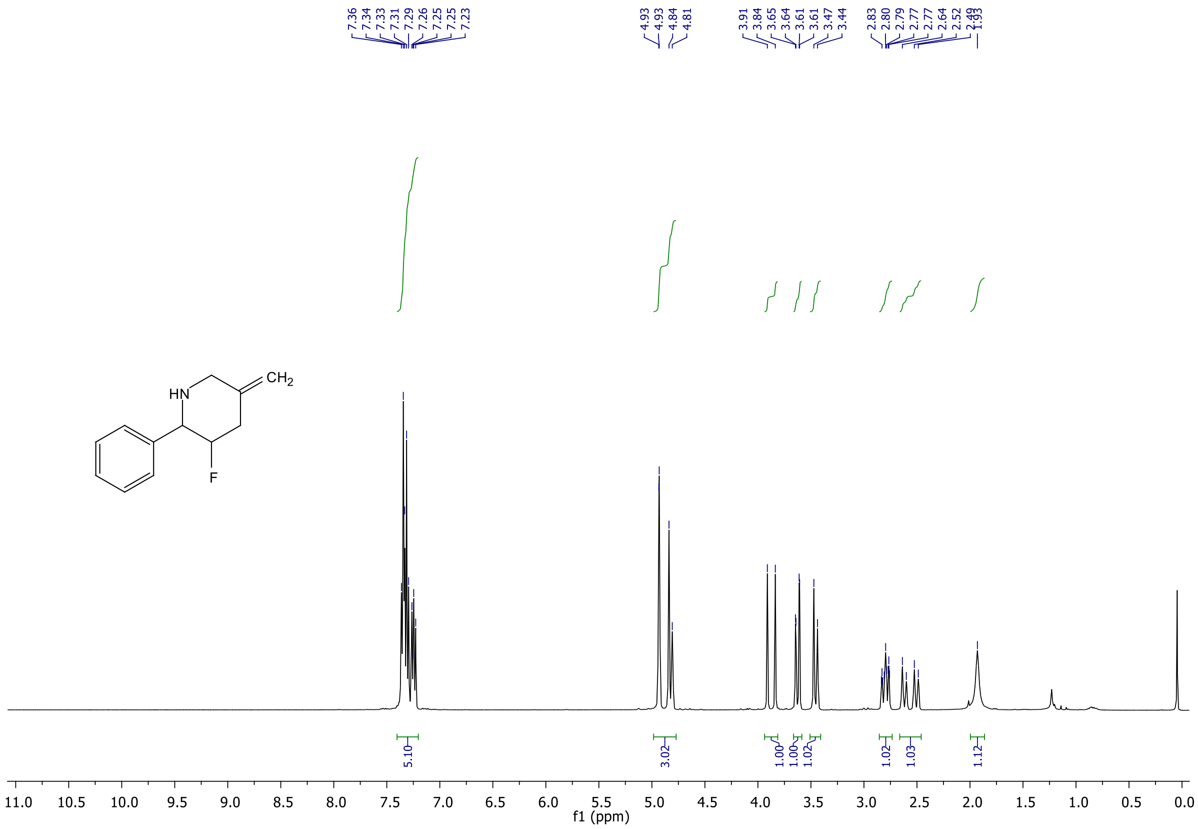

^{19}F NMR, $CDCl_3$, 377 MHz

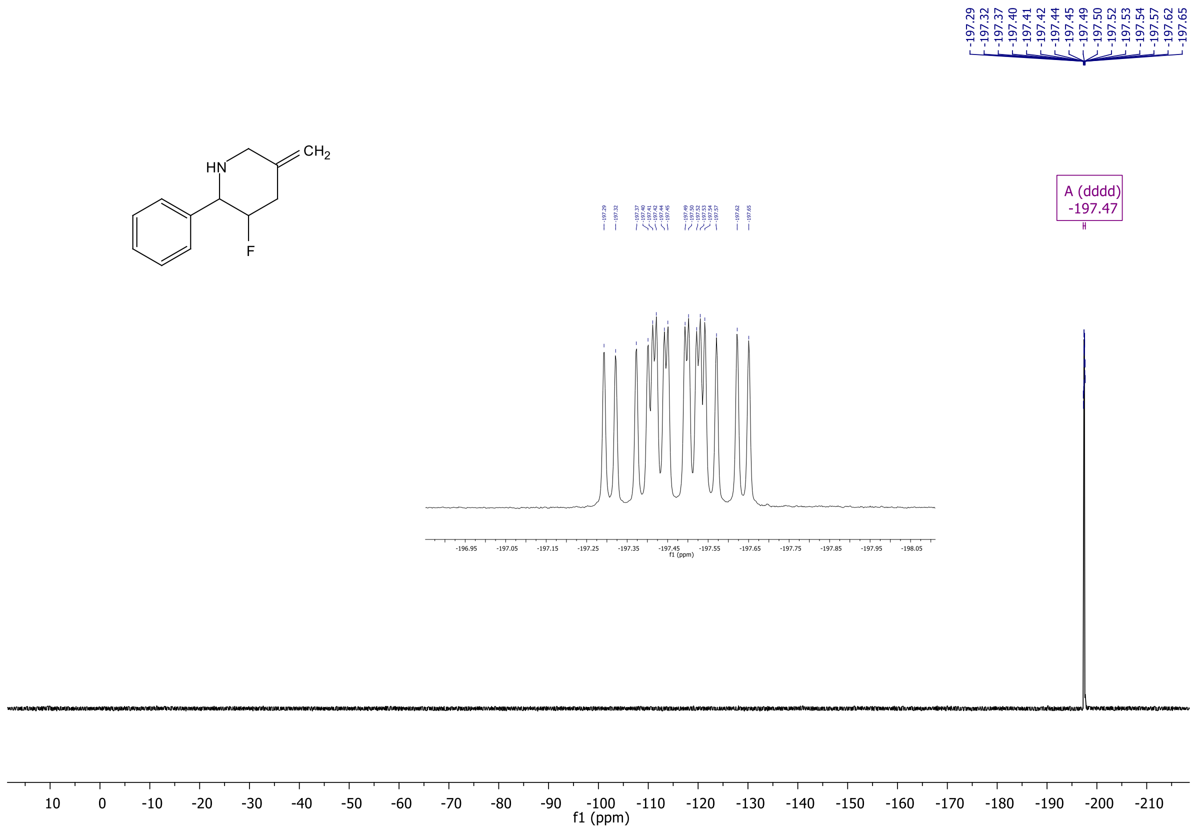

^{13}C NMR, CDCl$_3$, 101 MHz

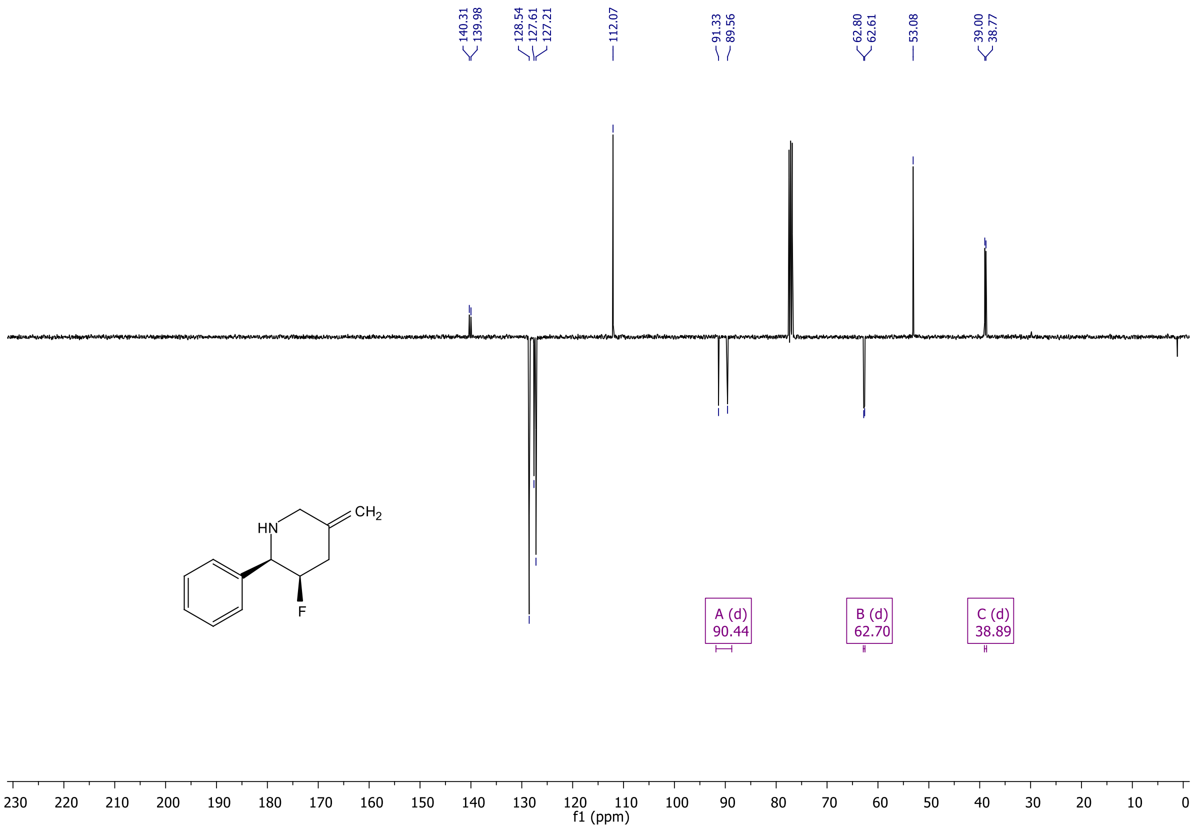

Ethyl 5-(4-acetoxybutylidene)-3-fluoro-2-phenyl-3,4,5,6-tetrahydropyridine-3-carboxylate (10)

^{1}H NMR, $CDCl_3$, 400 MHz

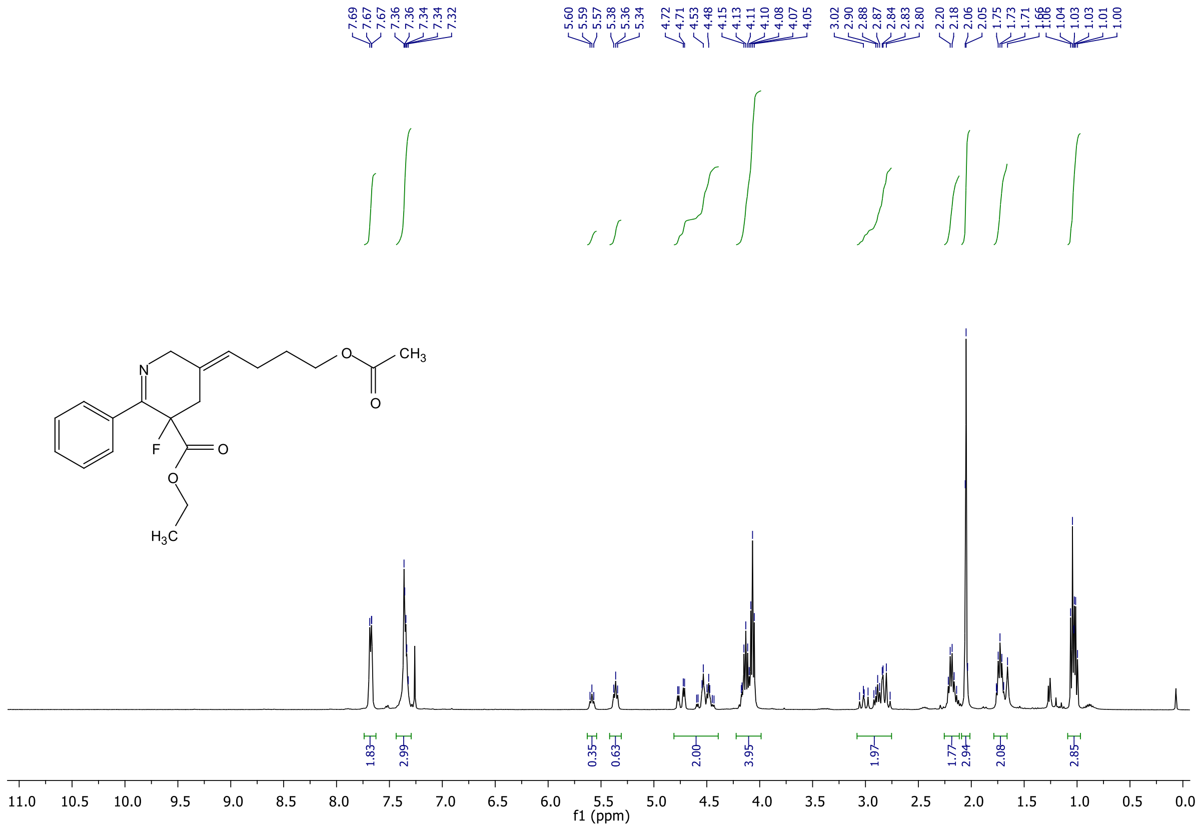

^{19}F NMR, $CDCl_3$, 377 MHz

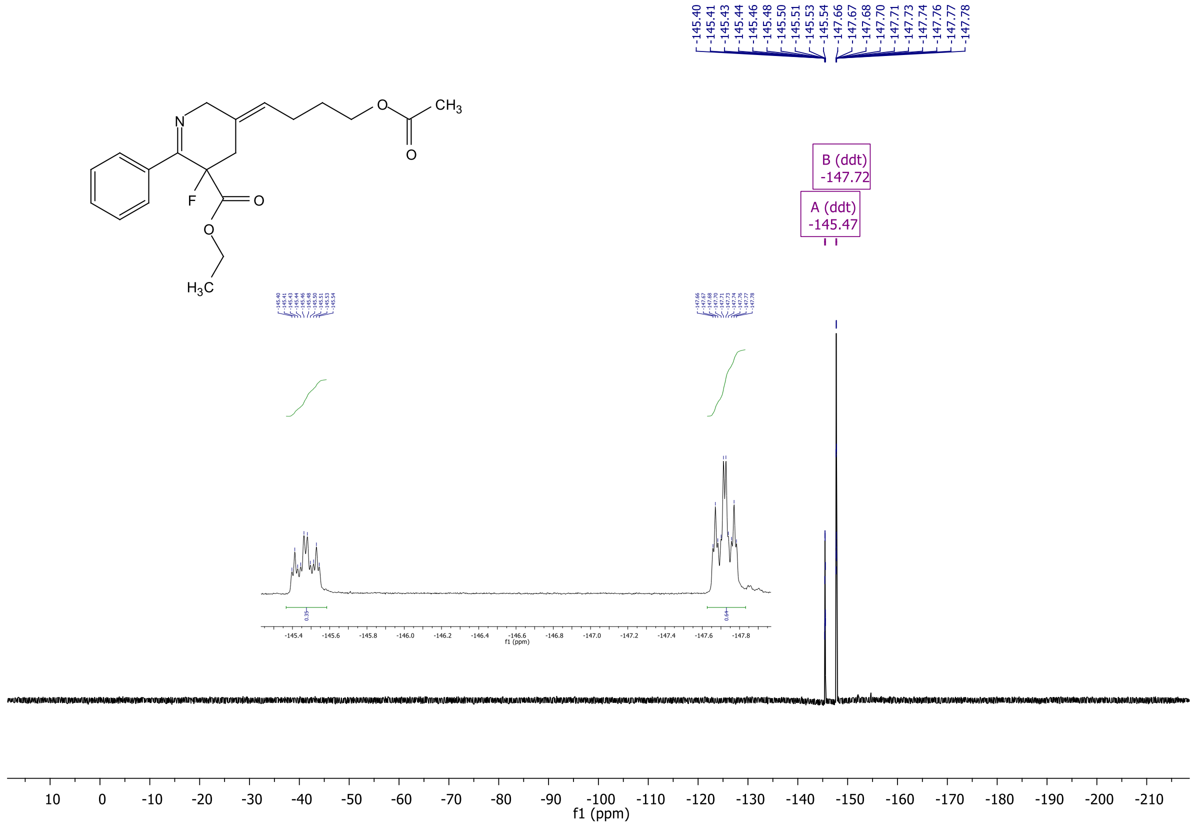

^{13}C NMR, $CDCl_3$, 101 MHz

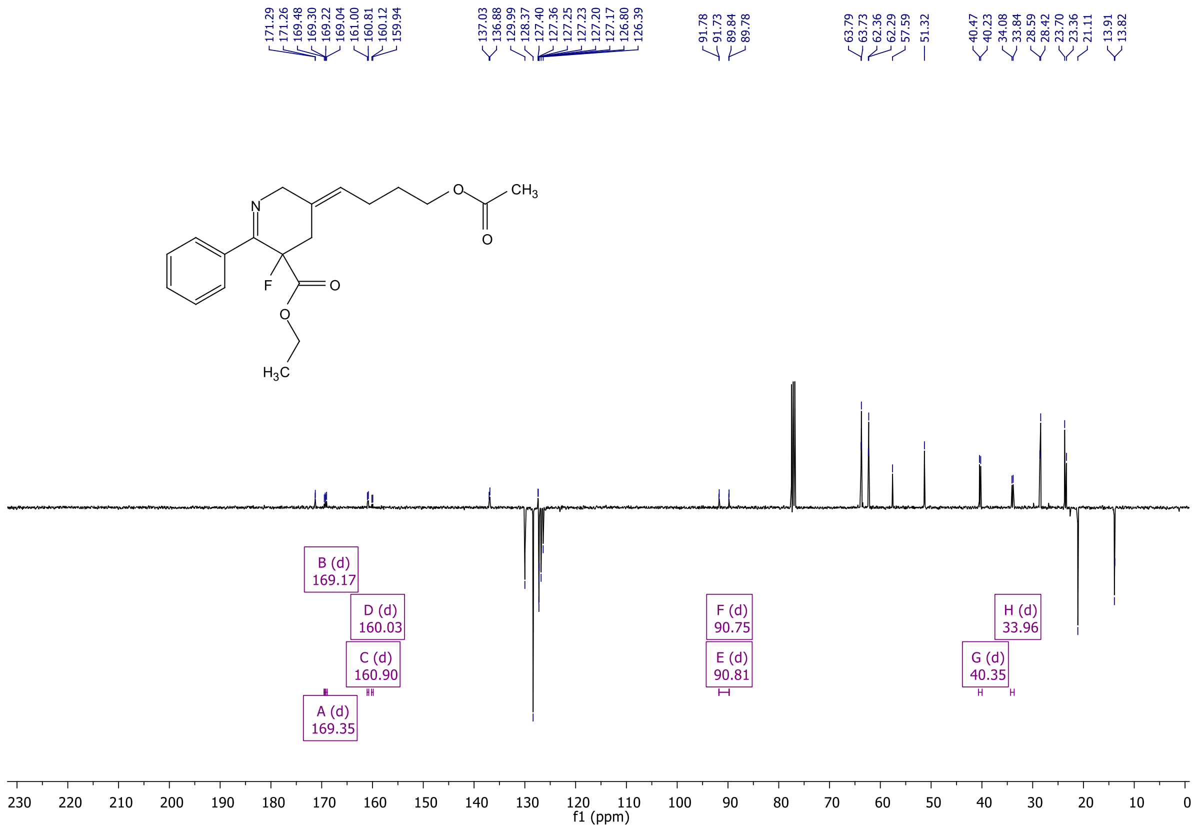

tert-Butyl (2-methylene-5-oxo-5-phenyl-4-((trifluoromethyl)thio)pentyl)carbamate (12a)

^{1}H NMR, $CDCl_3$, 400 MHz

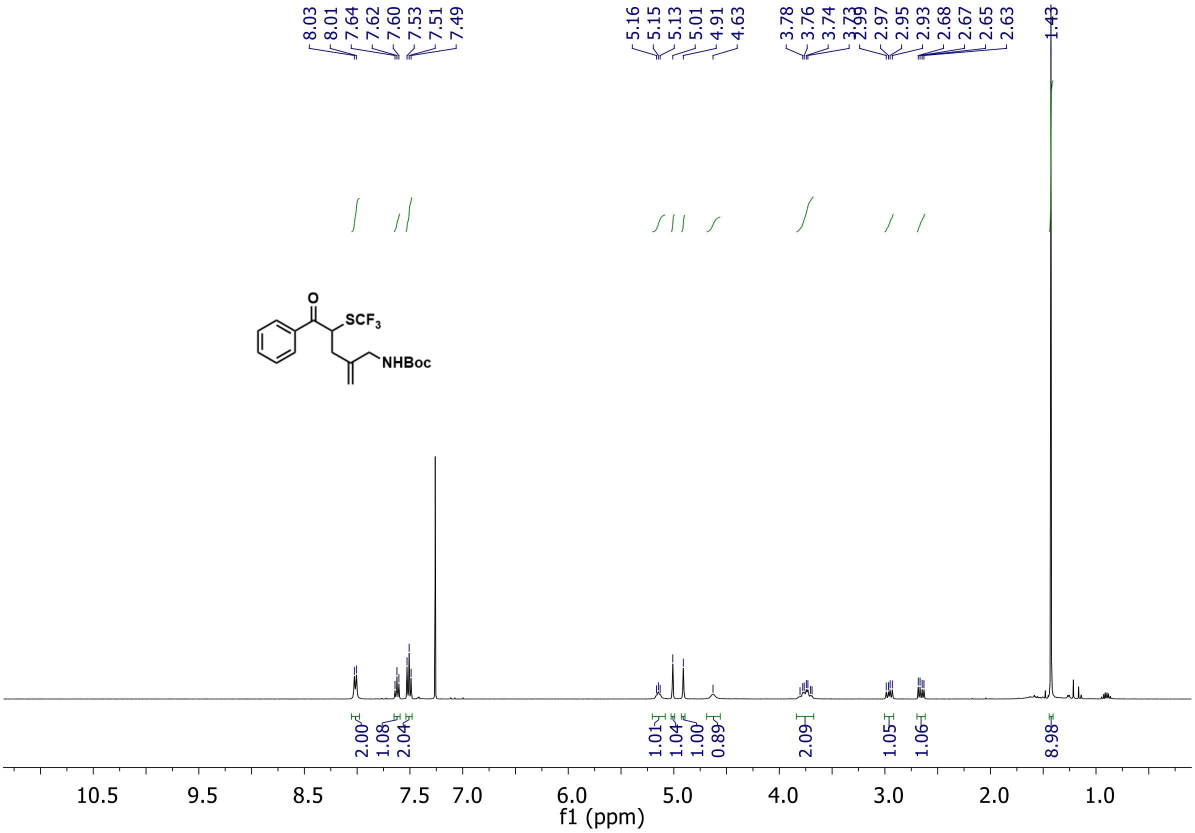

^{19}F NMR, $CDCl_3$, 377 MHz

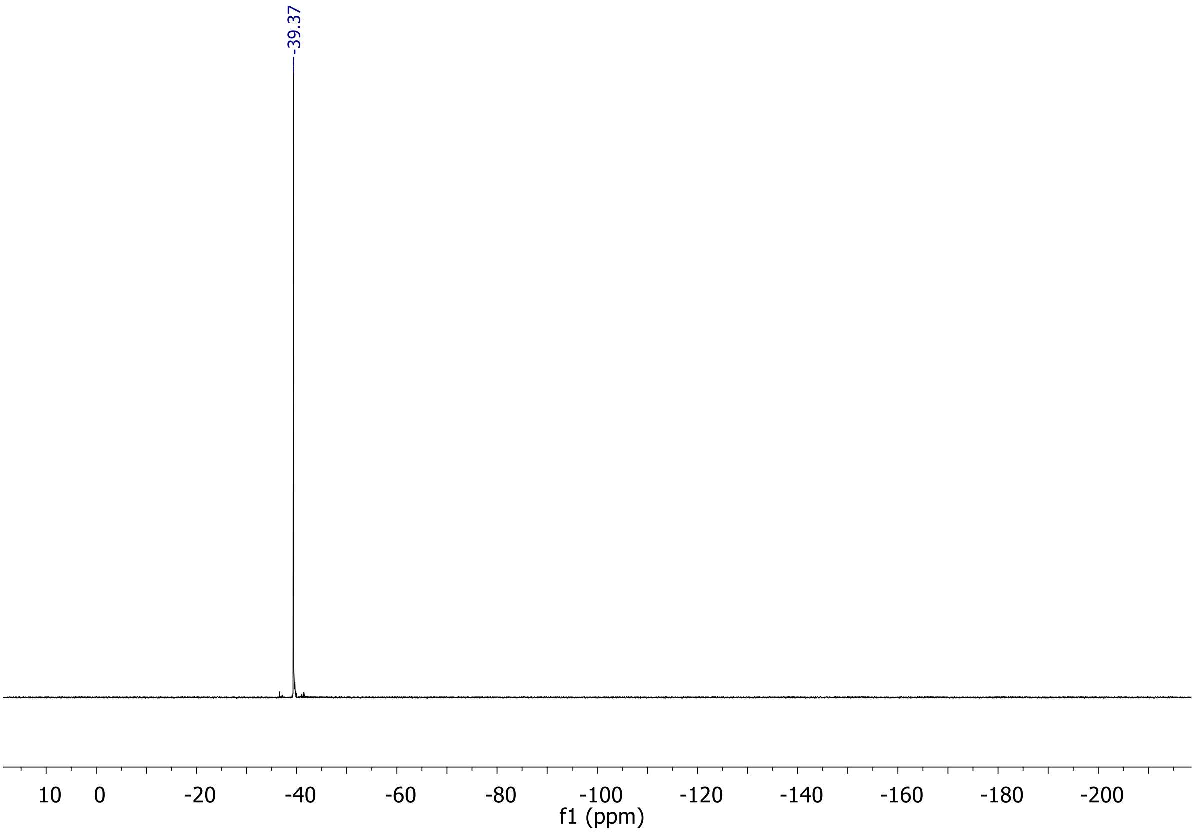

^{13}C NMR, $CDCl_3$, 101 MHz

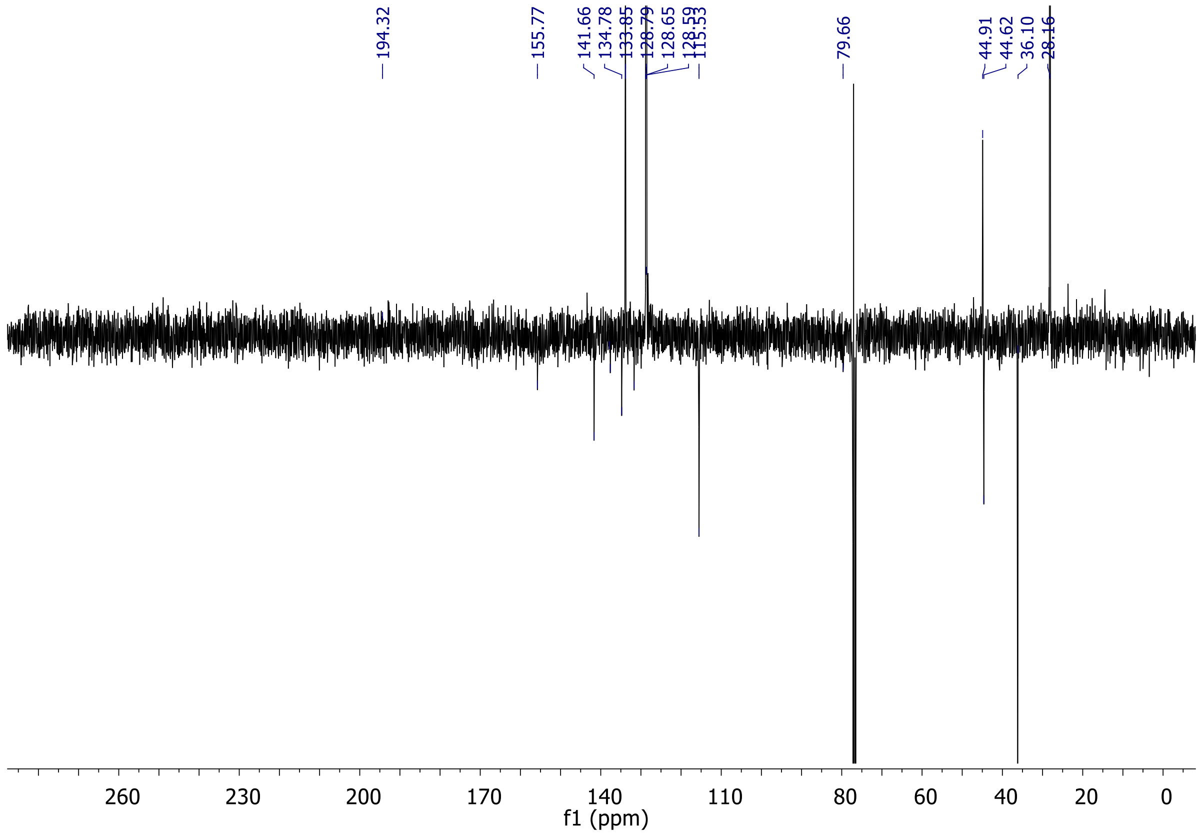

***tert*-Butyl (5-(4-methoxyphenyl)-2-methylene-5-oxo-4-((trifluoromethyl)thio)pentyl) carbamate (12b)**

^{1}H NMR, $CDCl_3$, 400 MHz

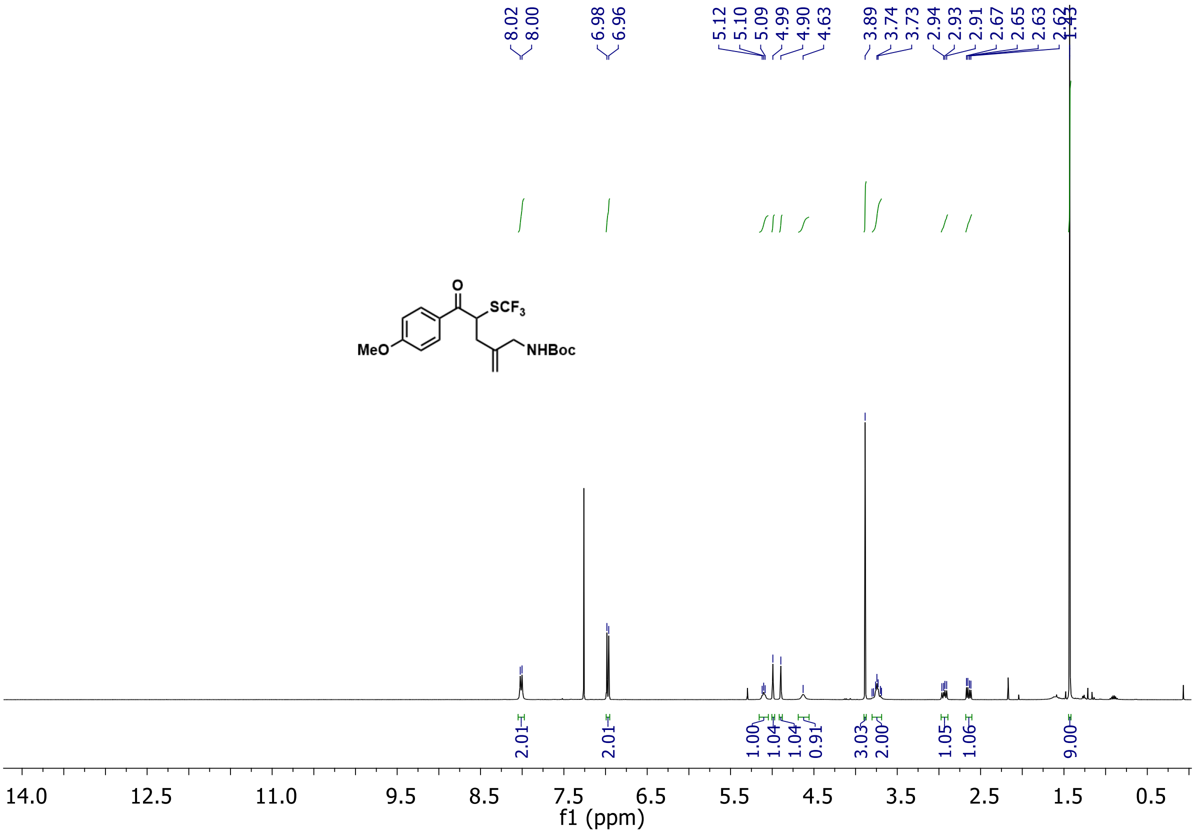

^{19}F NMR, $CDCl_3$, 377 MHz

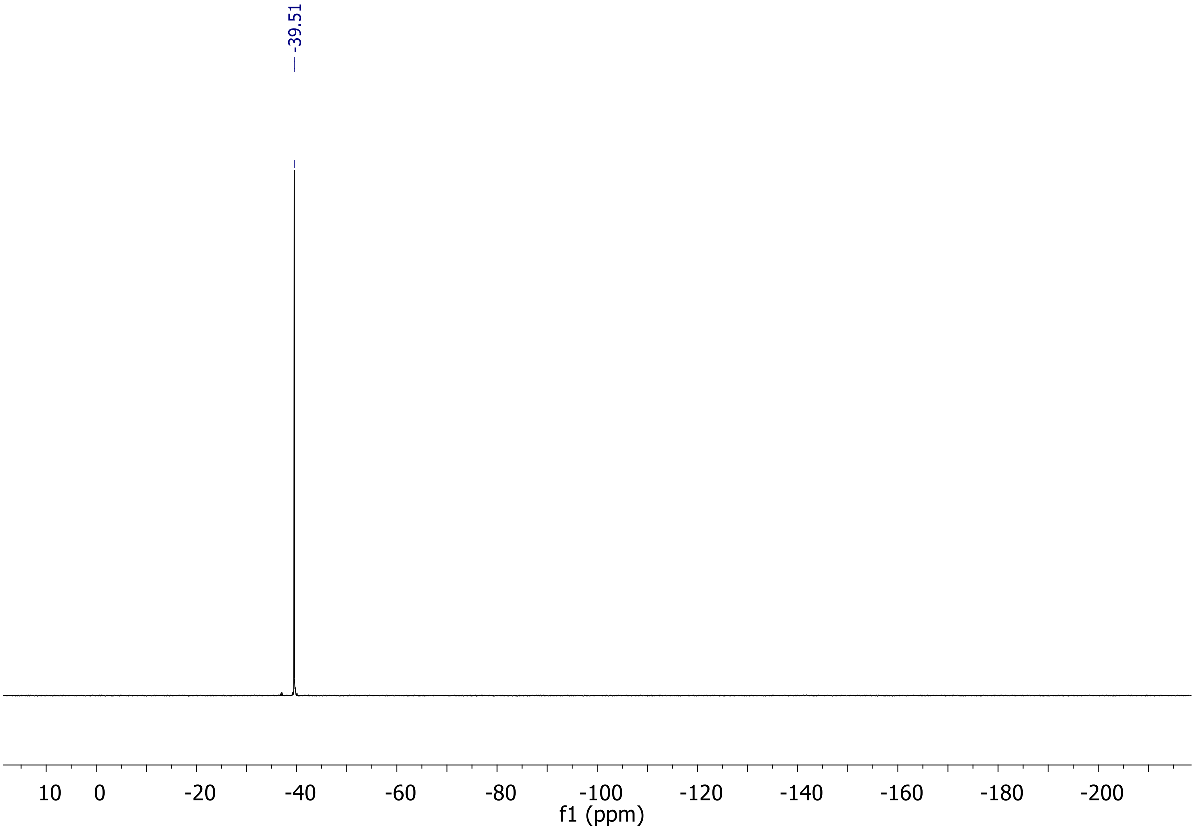

^{13}C NMR, $CDCl_3$, 101 MHz

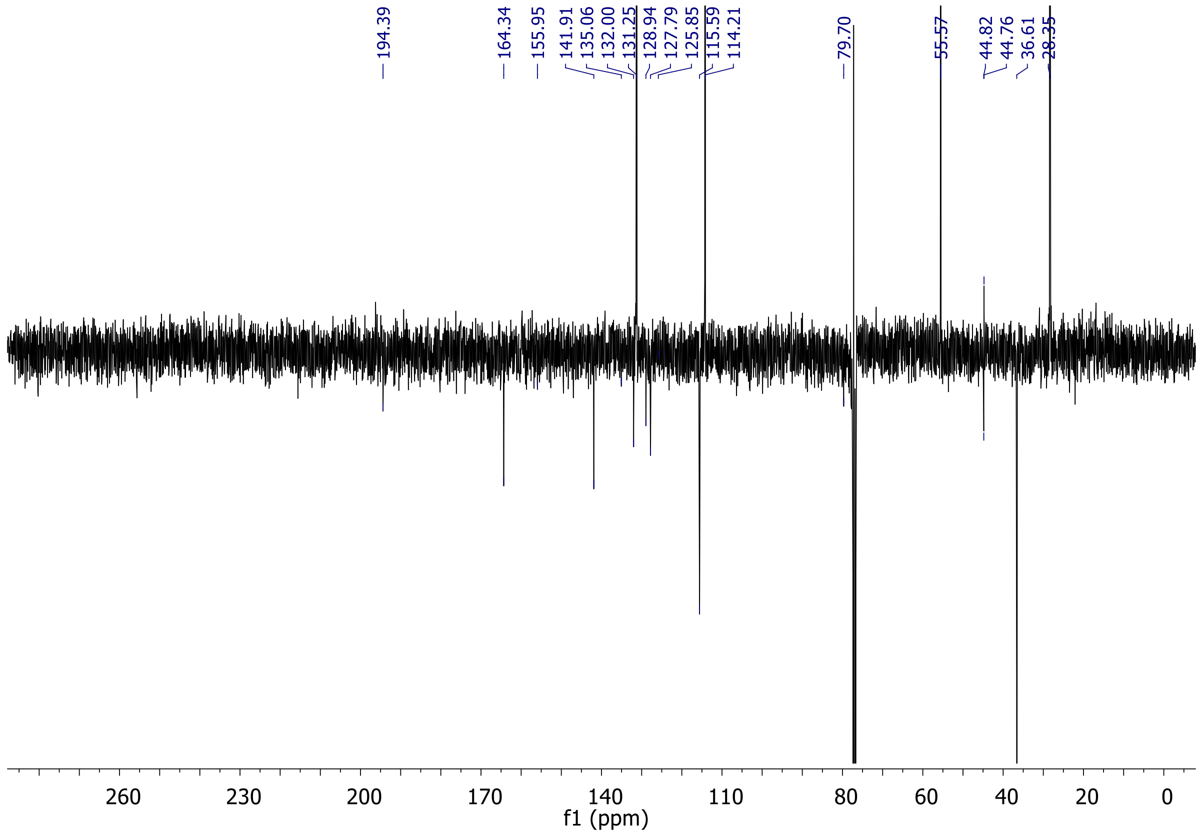

tert-Butyl (2-methylene-5-oxo-5-(p-tolyl)-4-((trifluoromethyl)thio)pentyl)carbamate (12c)

^{1}H NMR, $CDCl_3$, 400 MHz

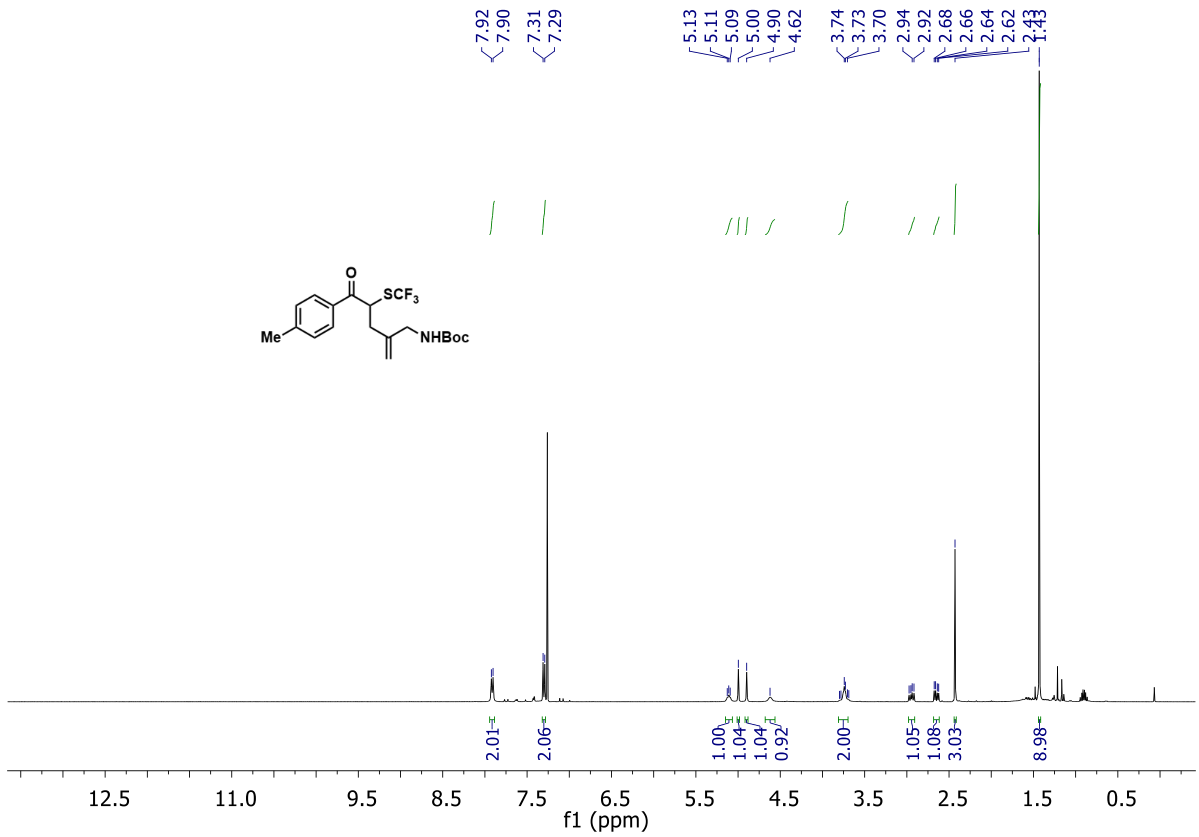

^{19}F NMR, $CDCl_3$, 377 MHz

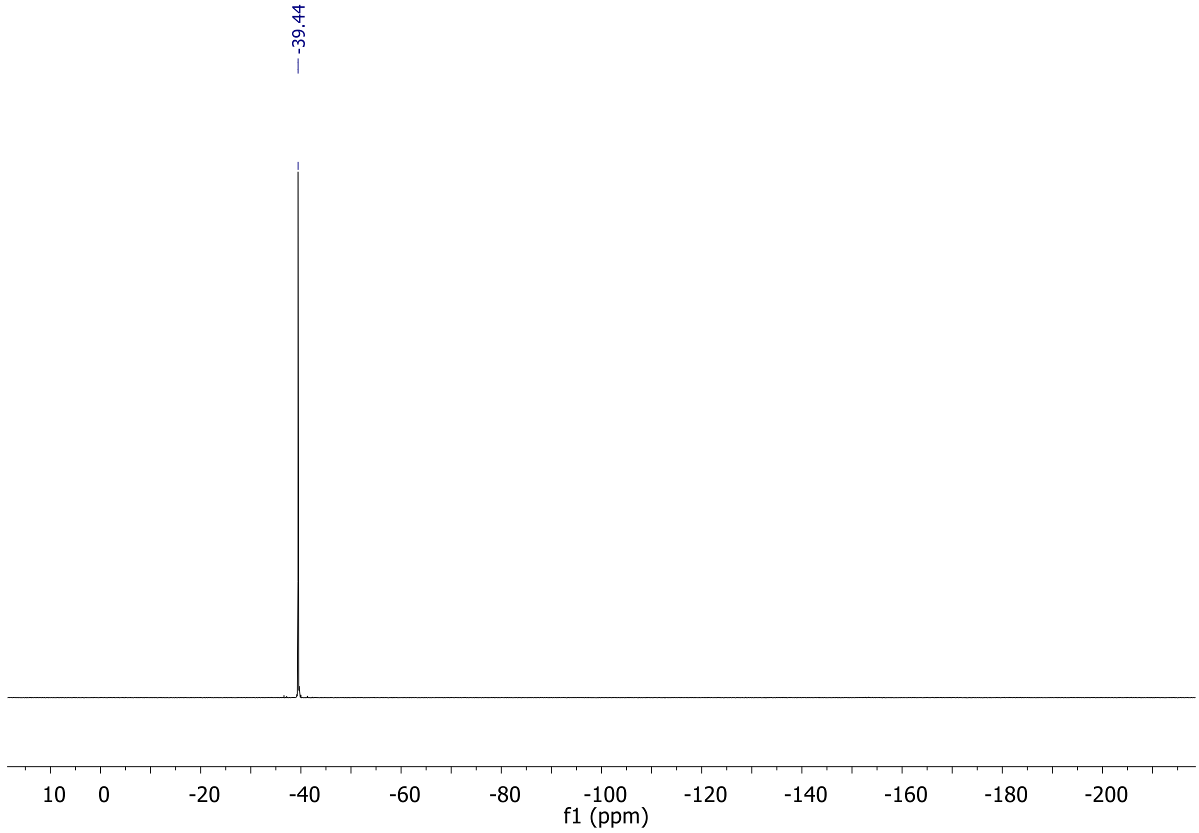

^{13}C NMR, $CDCl_3$, 101 MHz

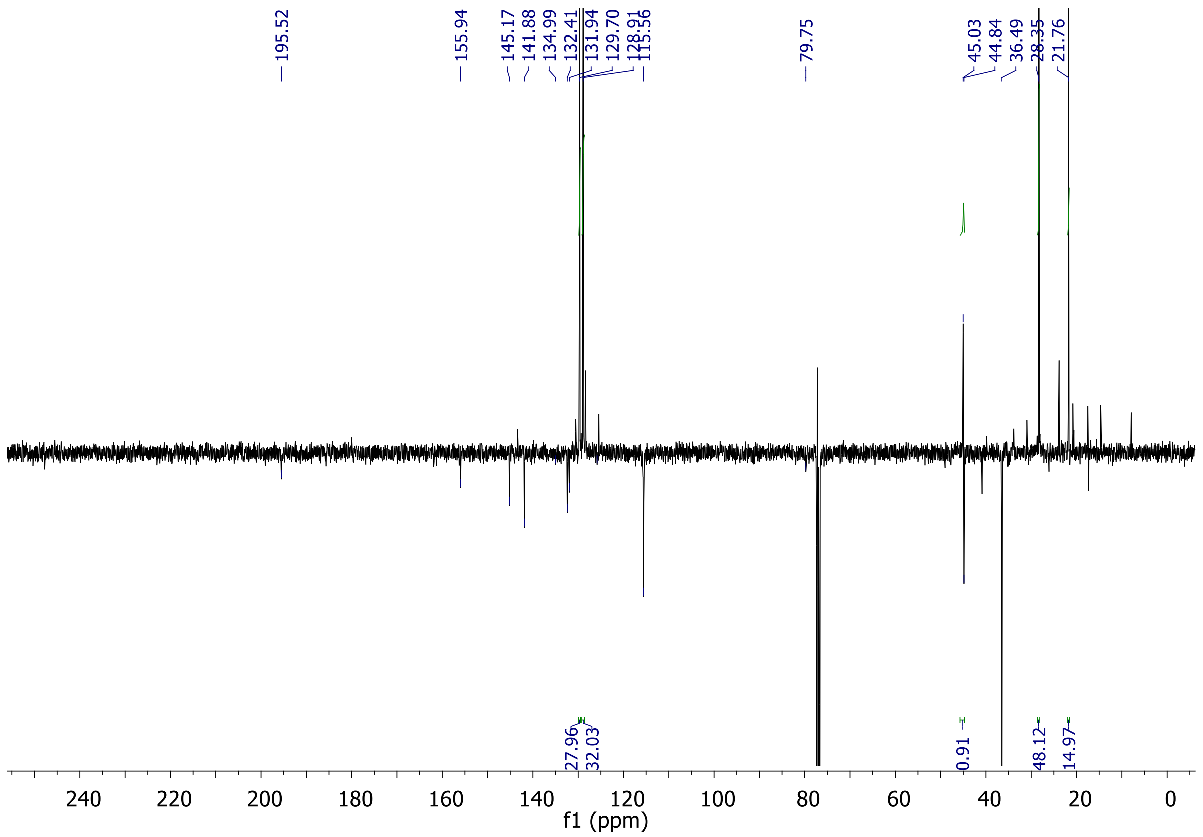

tert-Butyl (5-(4-chlorophenyl)-2-methylene-5-oxo-4-((trifluoromethyl)thio)pentyl) carbamate (12d)

^{1}H NMR, $CDCl_3$, 400 MHz

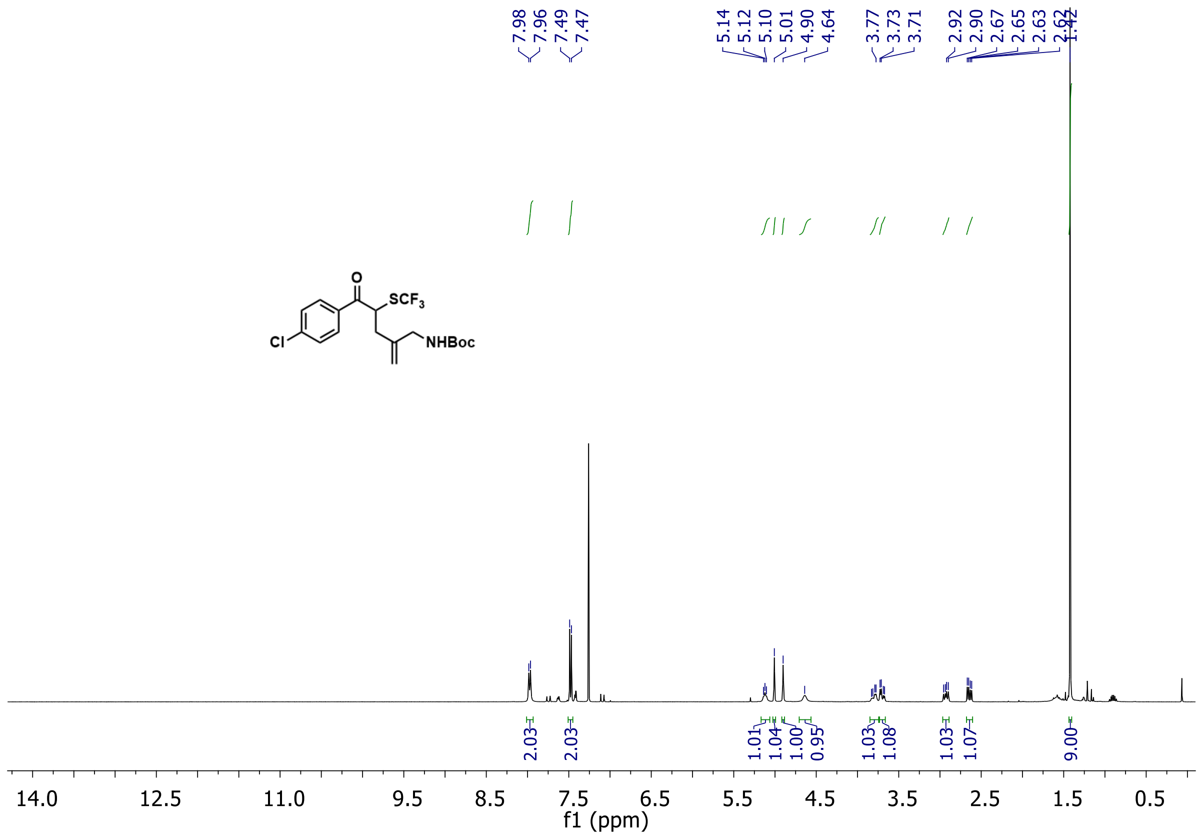

^{19}F NMR, $CDCl_3$, 377 MHz

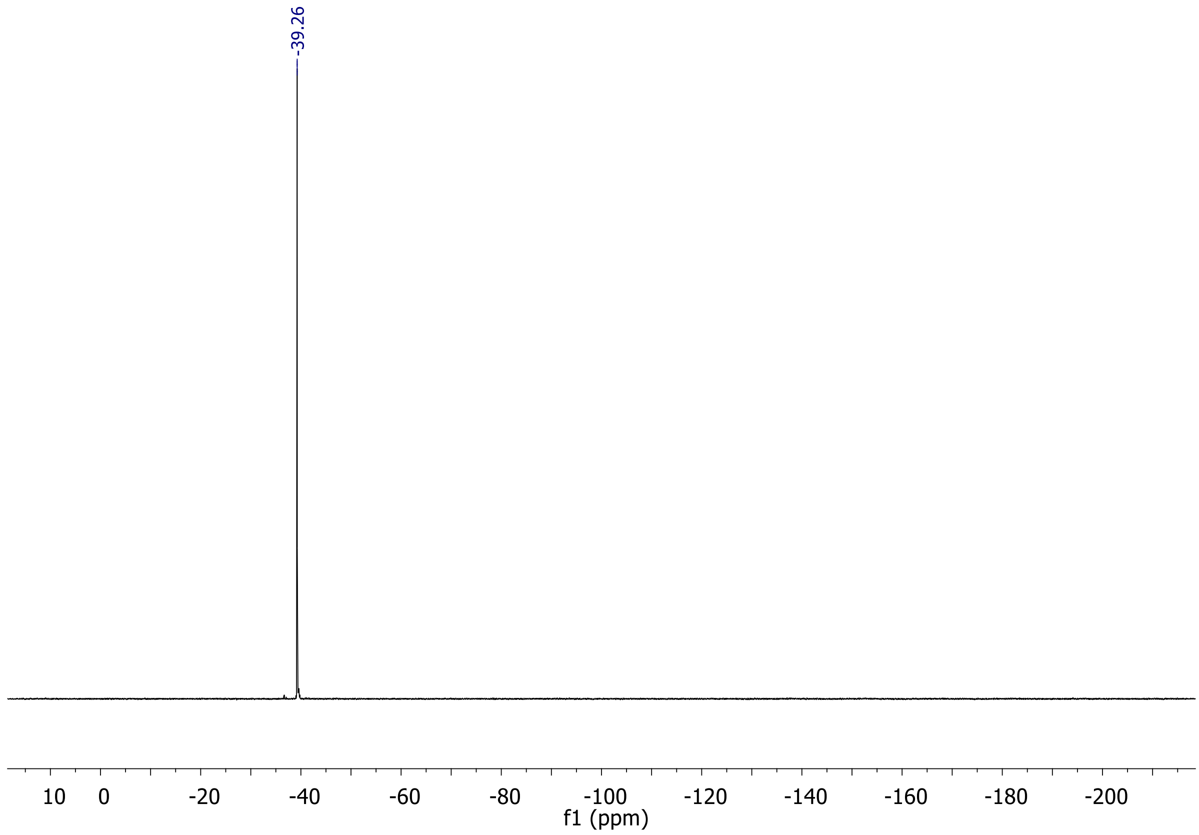

^{13}C NMR, $CDCl_3$, 101 MHz

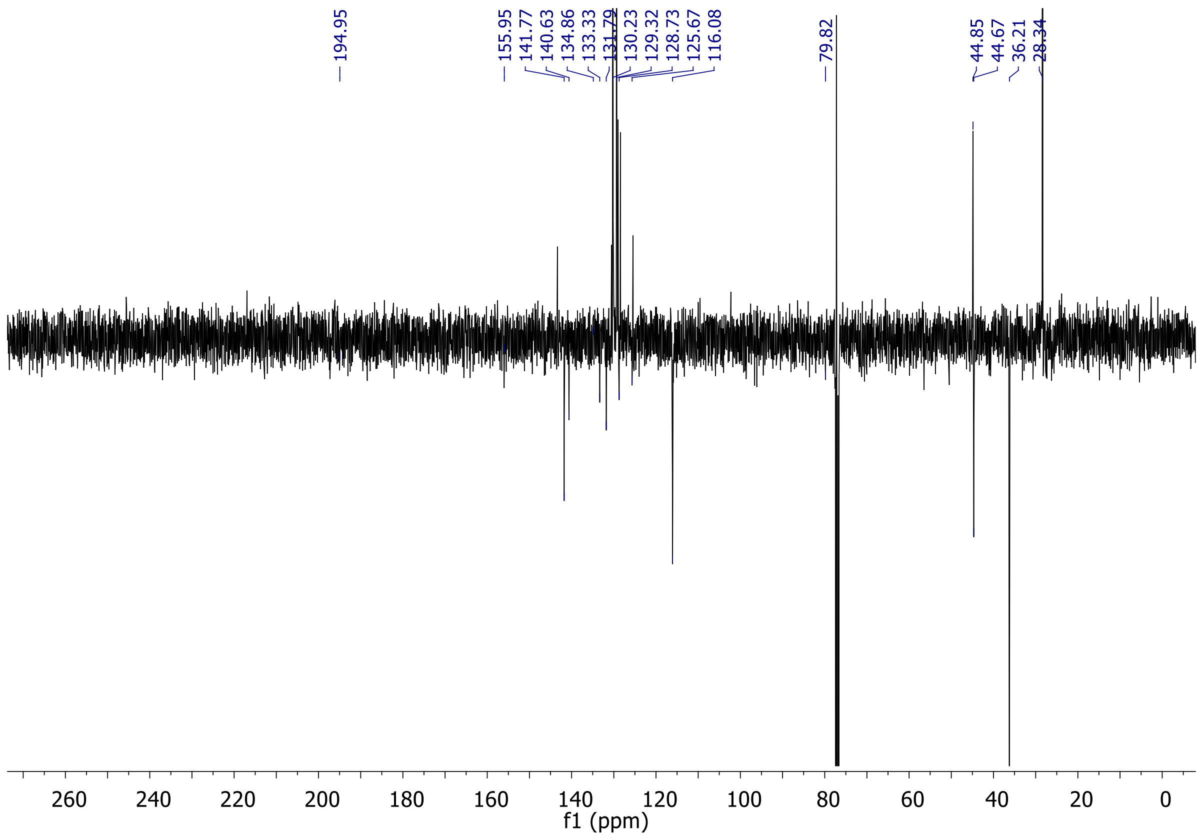

tert-Butyl (5-(2-methoxyphenyl)-2-methylene-5-oxo-4-((trifluoromethyl)thio)pentyl) carbamate (12e)

^{1}H NMR, $CDCl_3$, 400 MHz

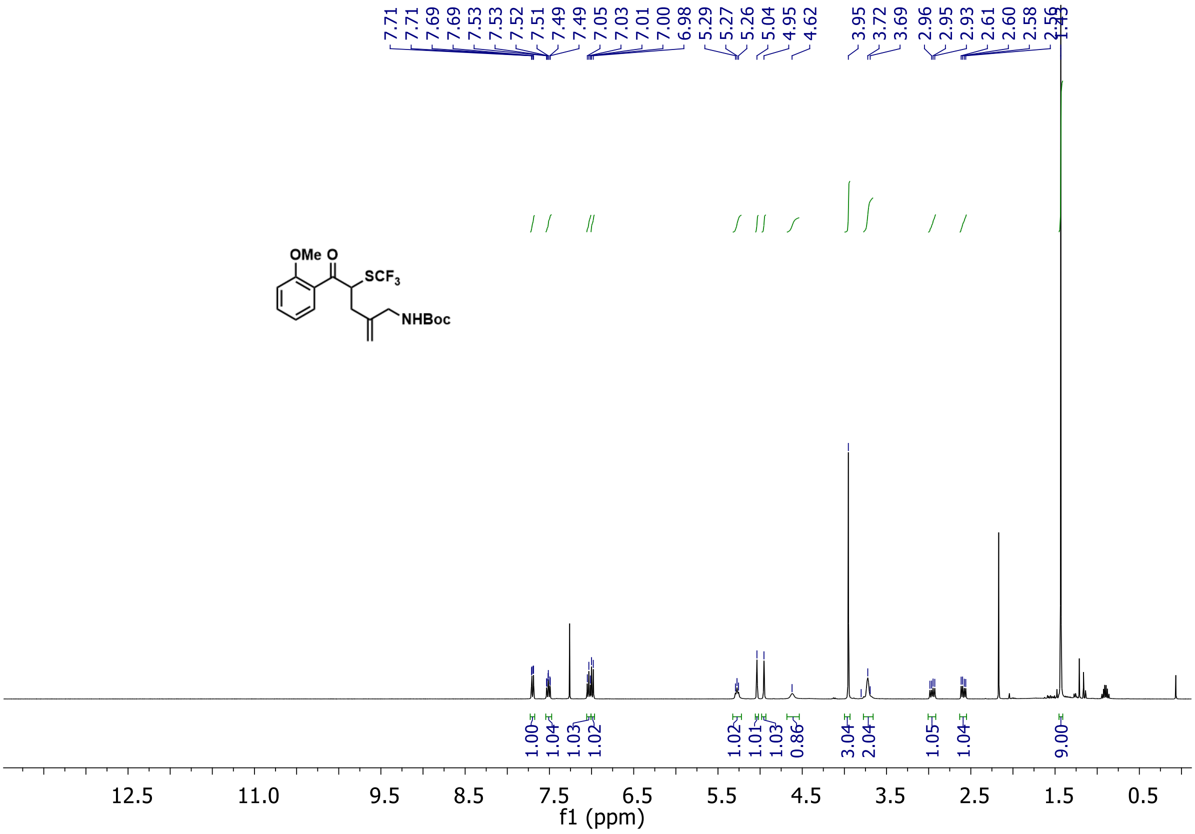

^{19}F NMR, $CDCl_3$, 377 MHz

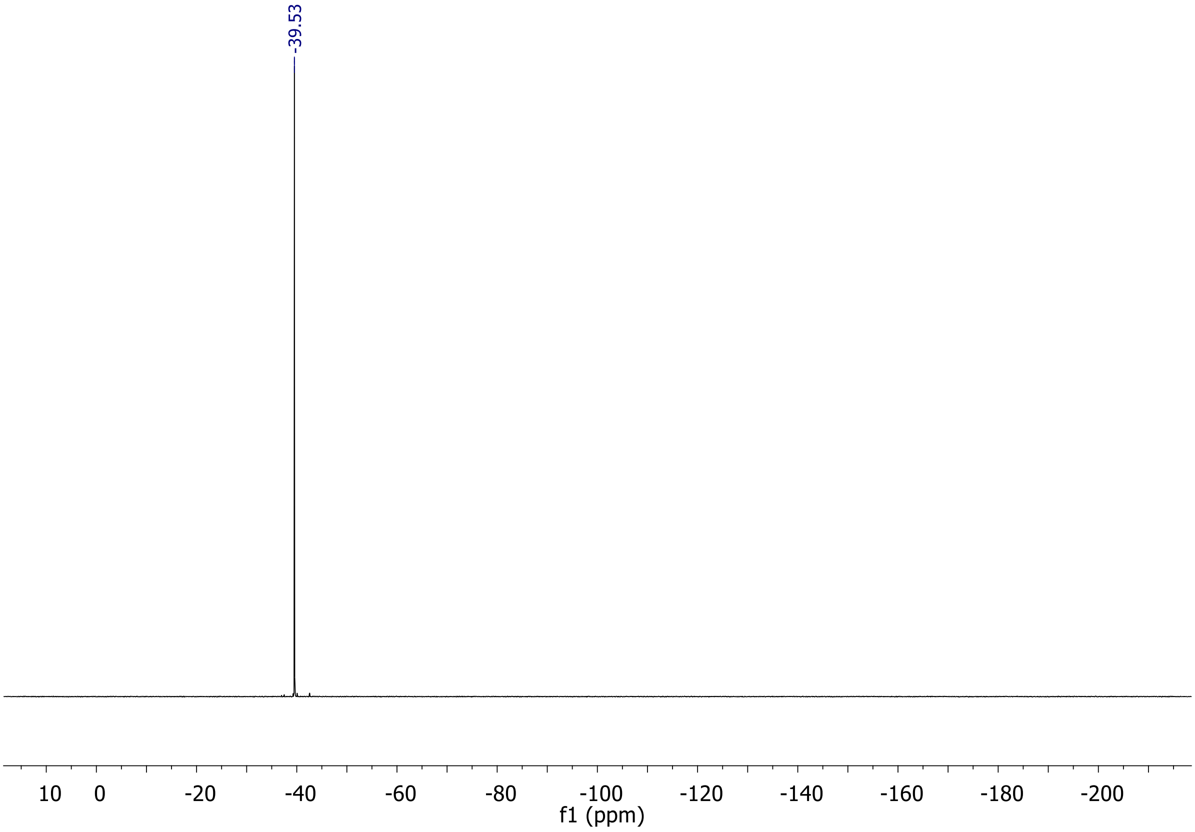

^{13}C NMR, $CDCl_3$, 101 MHz

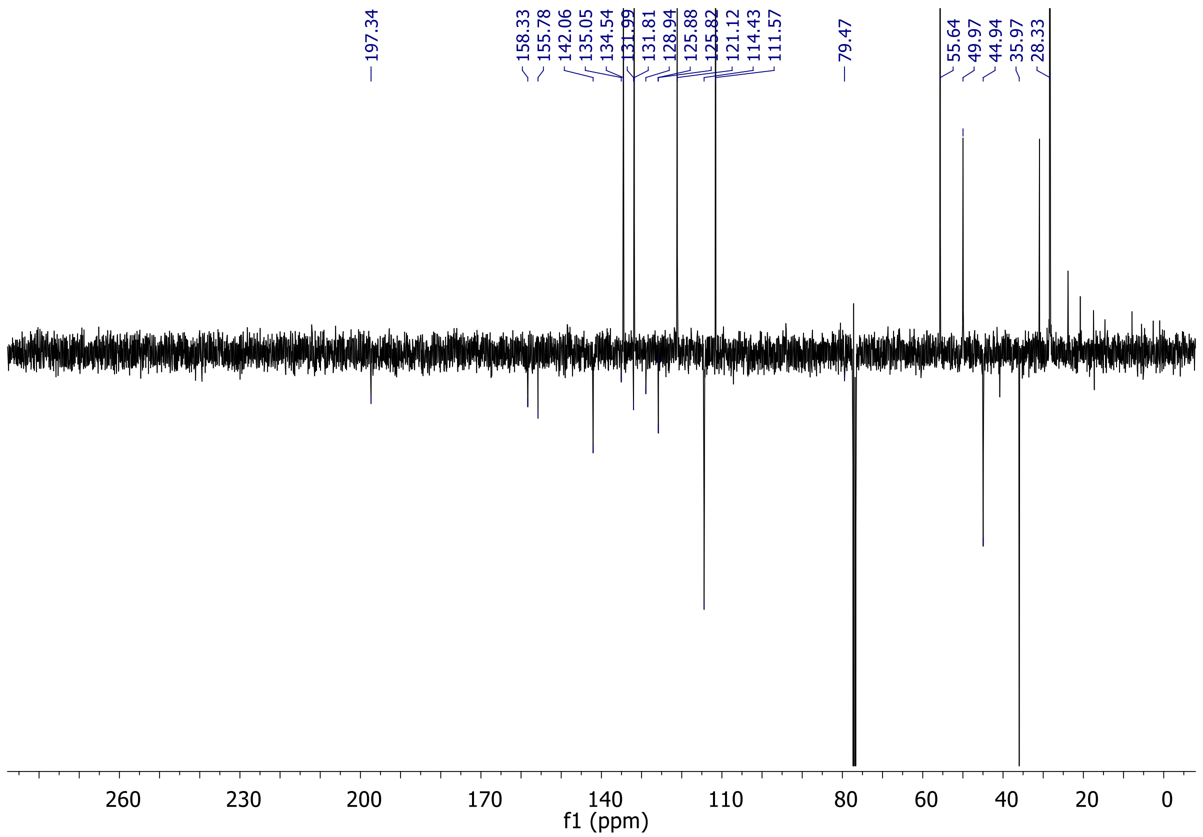

tert-Butyl (5-(3-chlorophenyl)-2-methylene-5-oxo-4-((trifluoromethyl)thio)pentyl) carbamate (12f)

^{1}H NMR, $CDCl_3$, 400 MHz

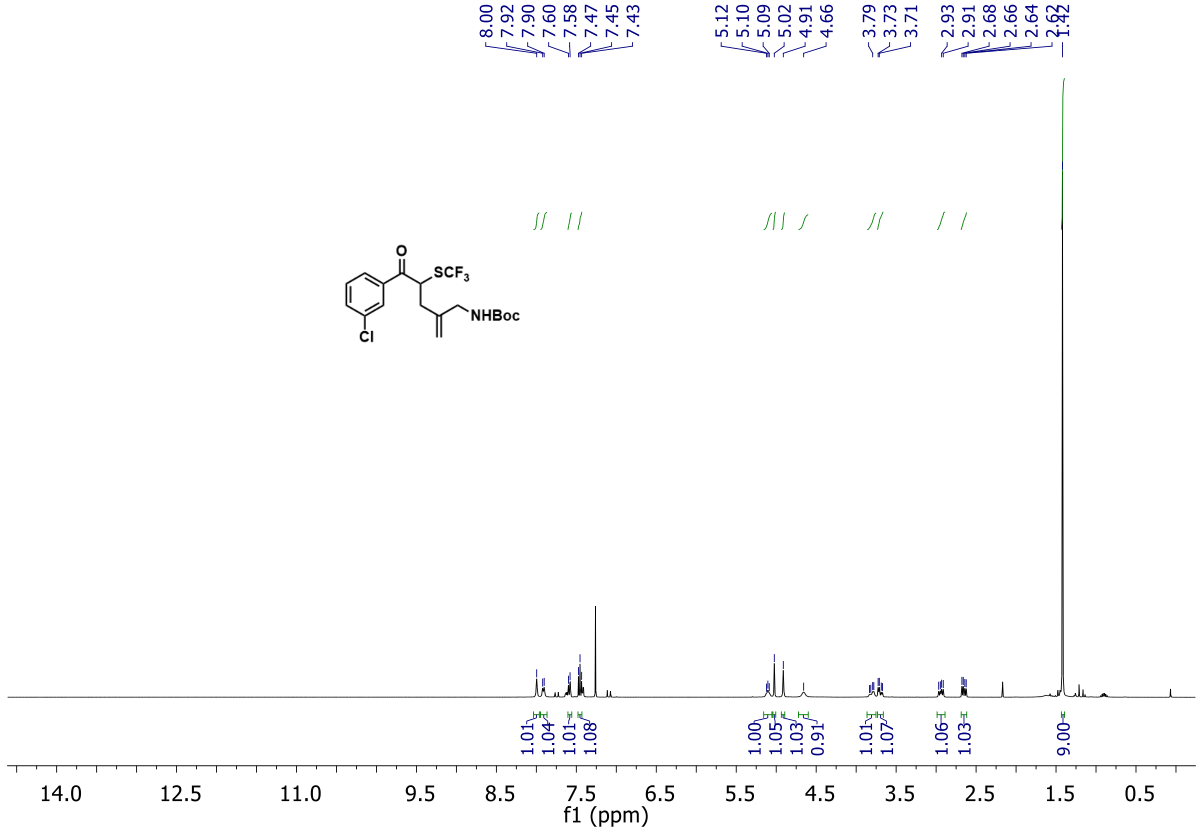

^{19}F NMR, $CDCl_3$, 377 MHz

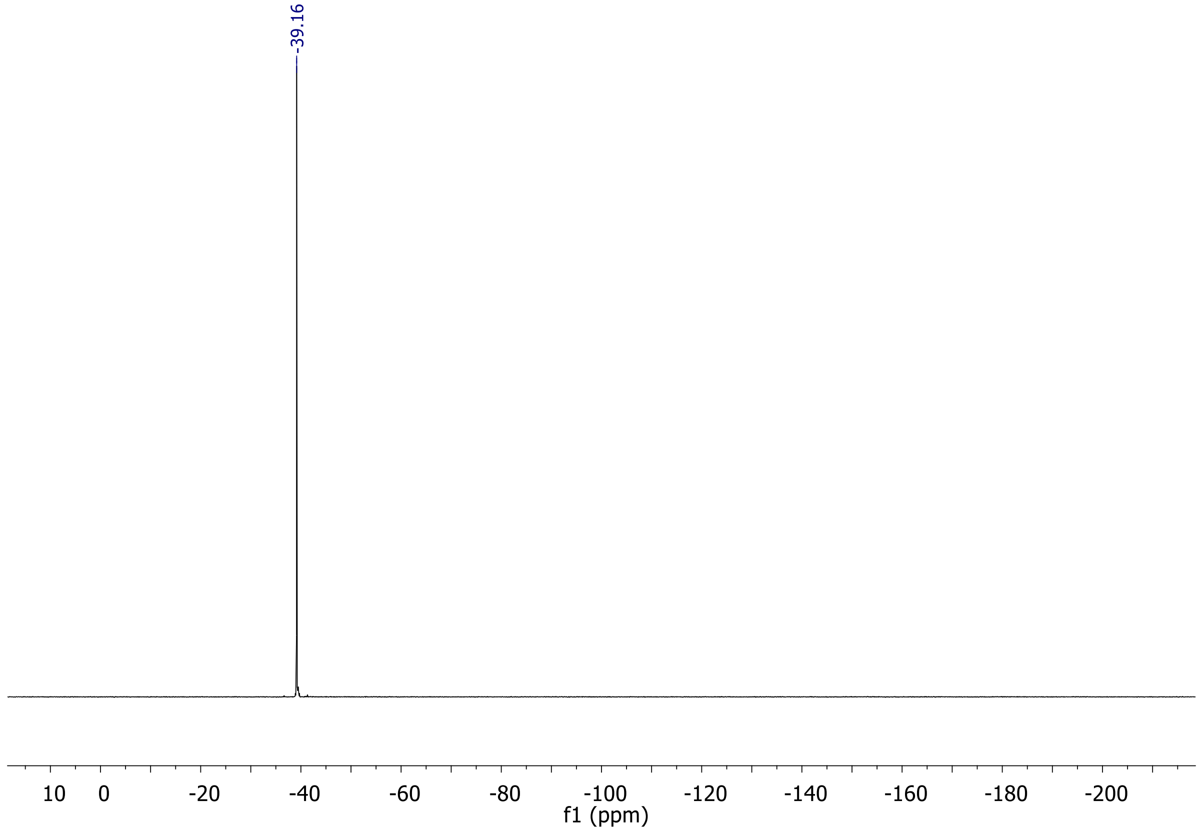

^{13}C NMR, $CDCl_3$, 101 MHz

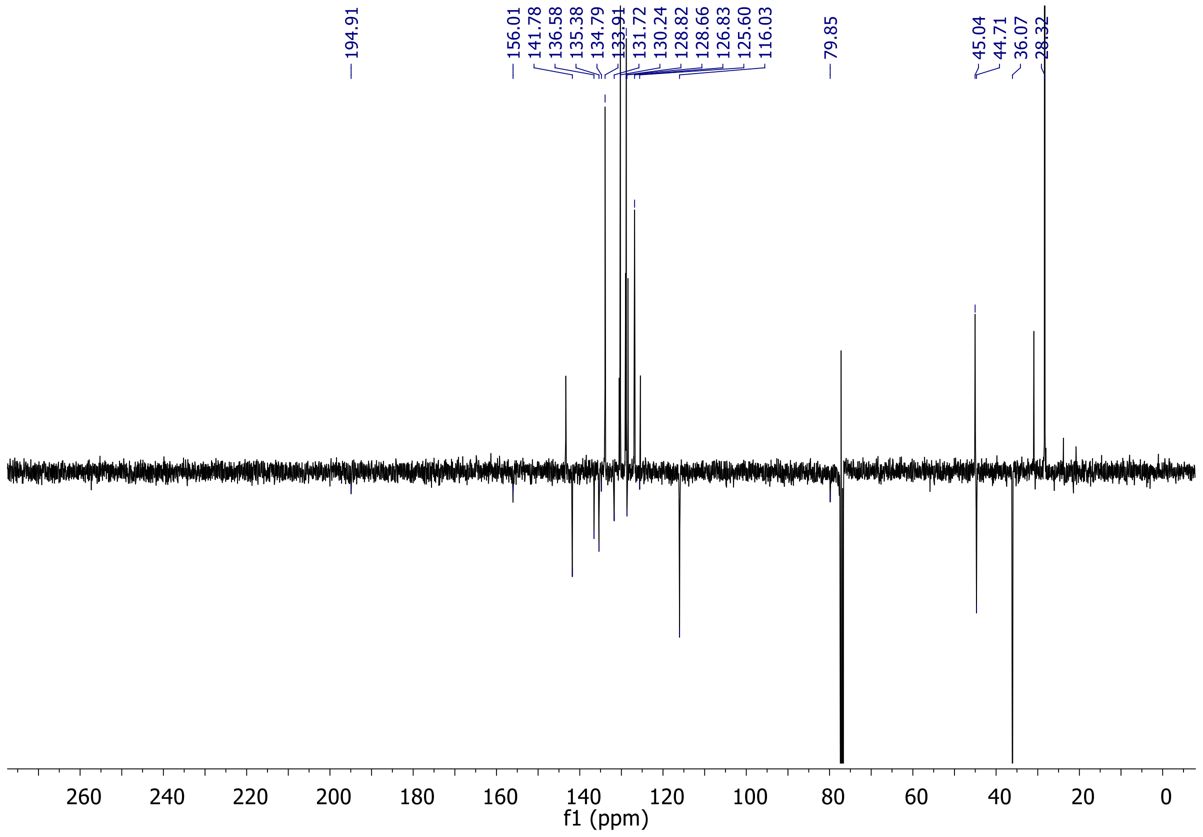

***tert*-Butyl (2-methylene-5-oxo-5-(thiophen-2-yl)-4-((trifluoromethyl)thio)pentyl) carbamate (12g)**

^{1}H NMR, $CDCl_3$, 400 MHz

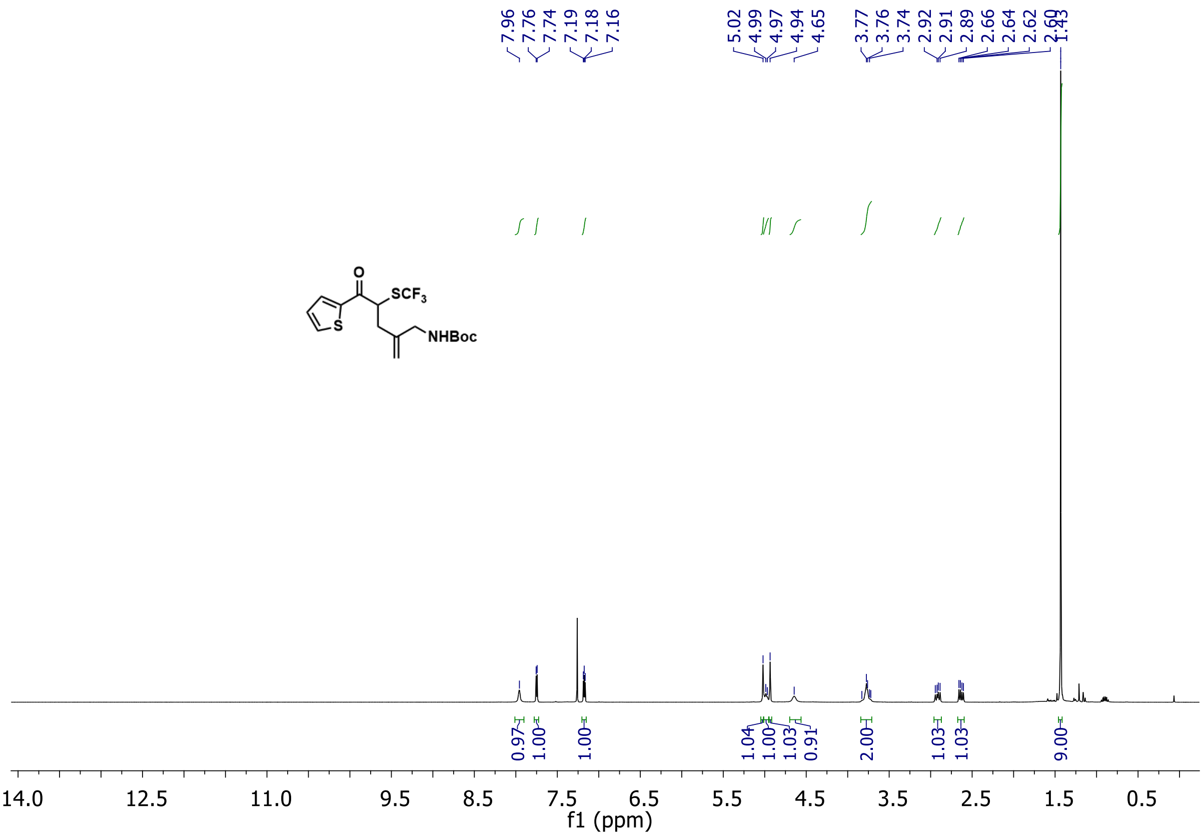

^{19}F NMR, $CDCl_3$, 377 MHz

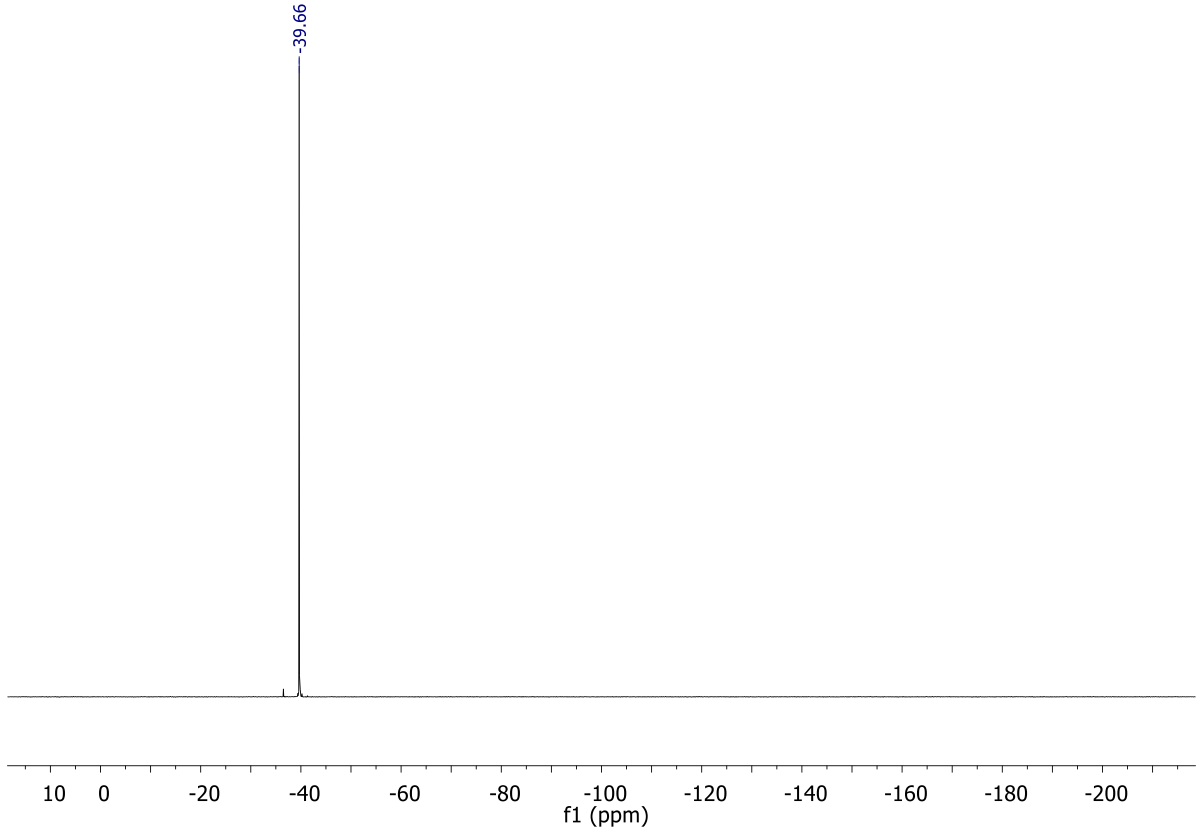

^{13}C NMR, $CDCl_3$, 101 MHz

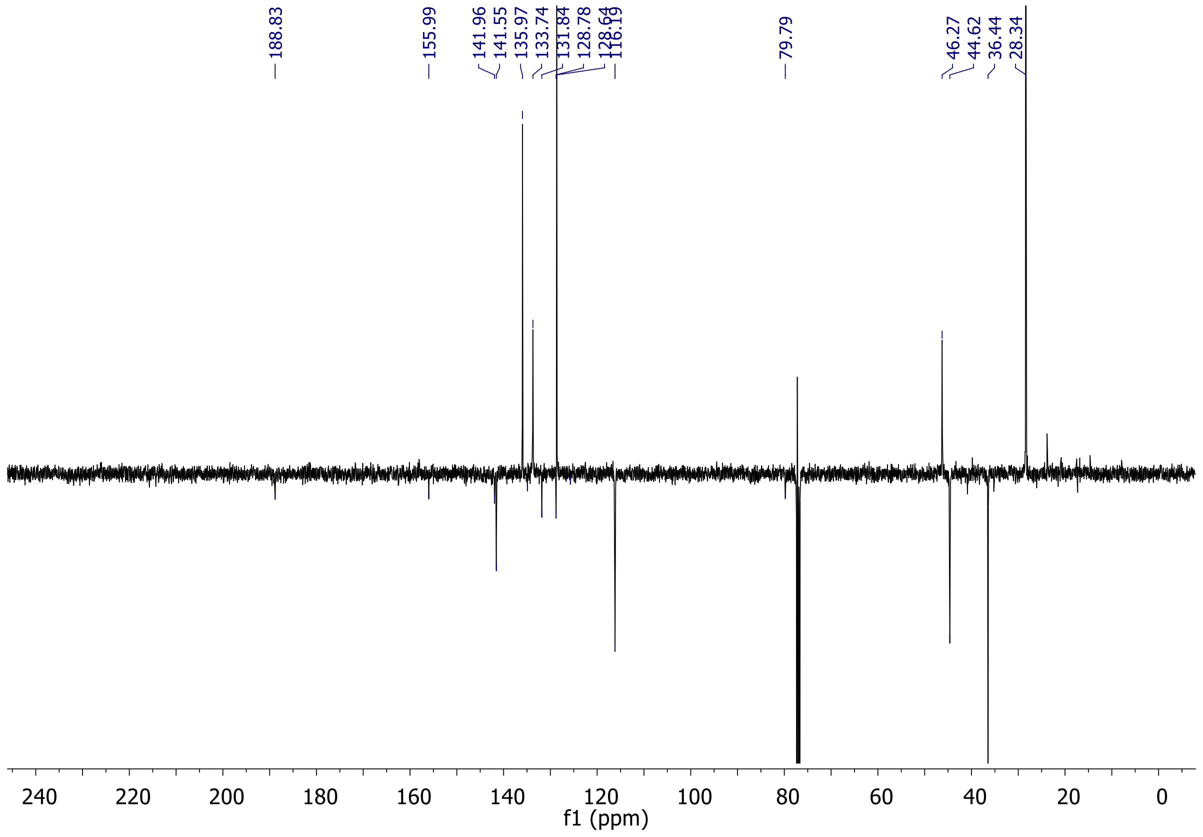

***tert*-Butyl (4-methyl-2-methylene-5-oxo-5-phenyl-4-((trifluoromethyl)thio)pentyl) carbamate (12h)**

^{1}H NMR, $CDCl_3$, 400 MHz

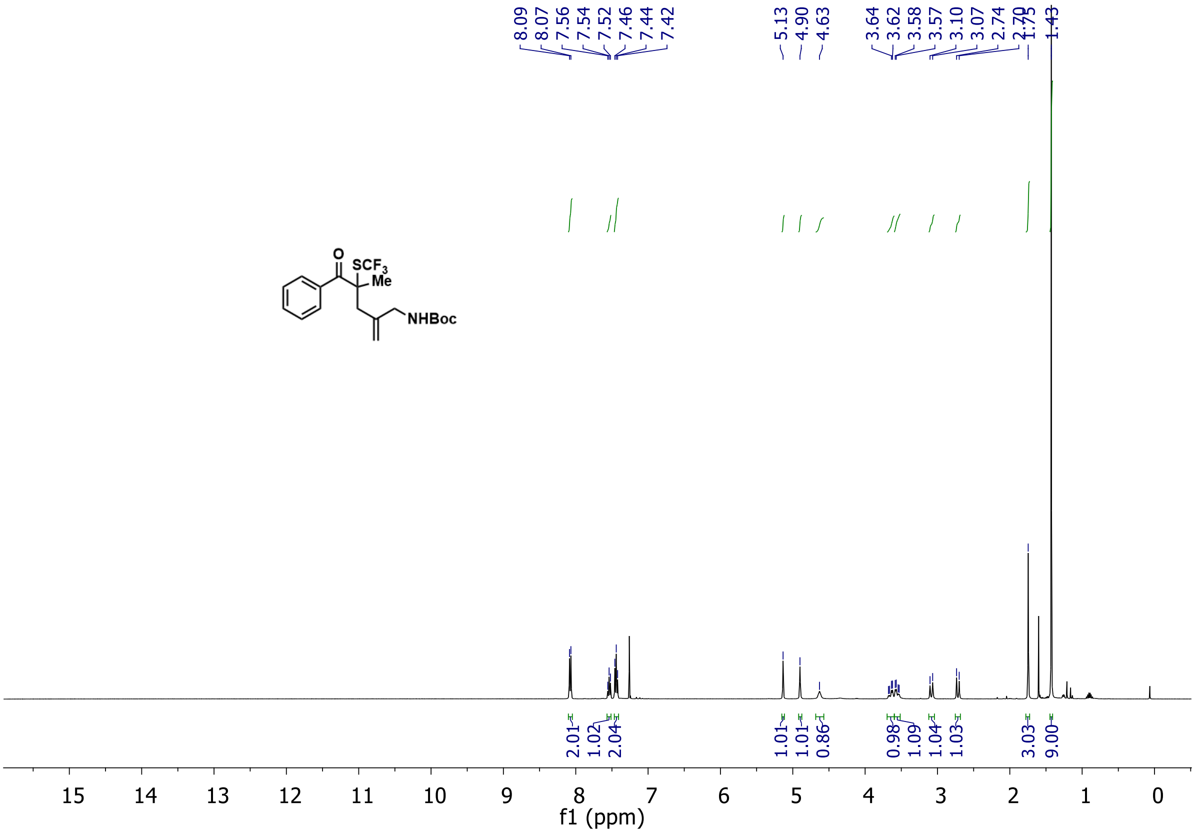

^{19}F NMR, $CDCl_3$, 377 MHz

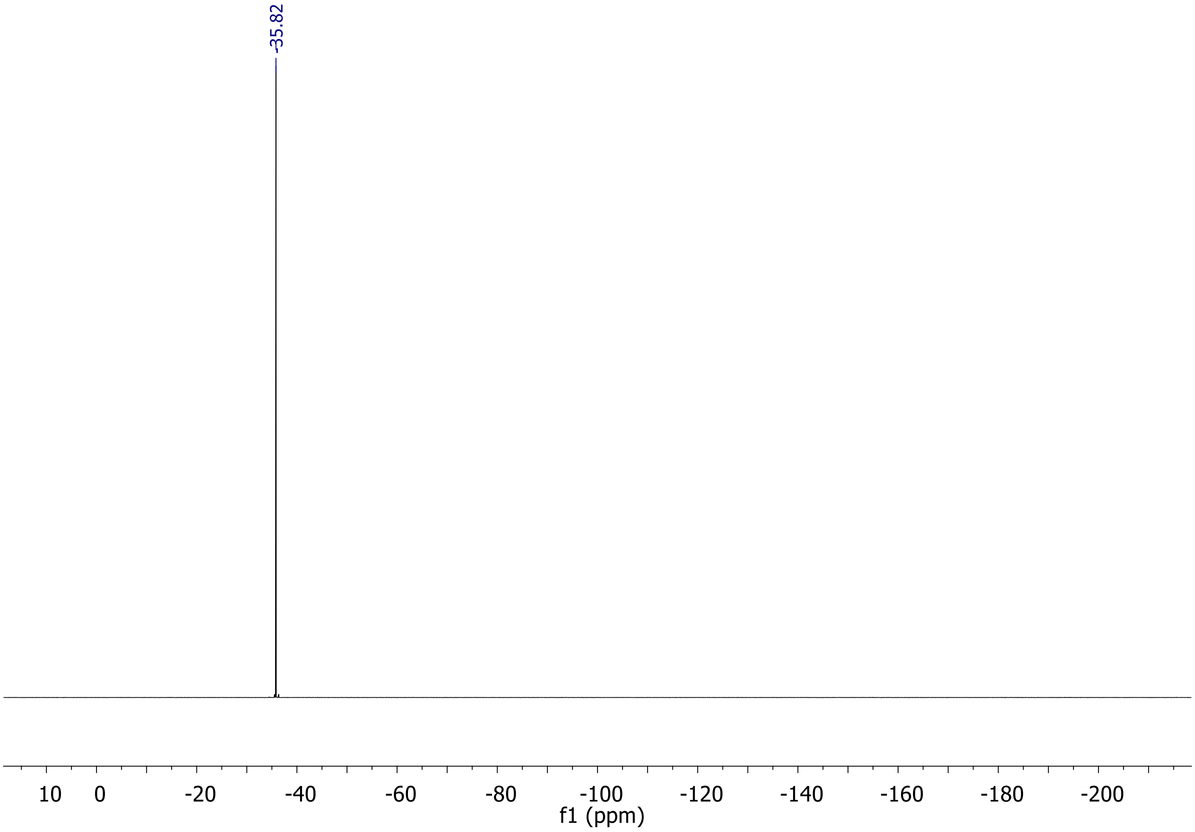

^{13}C NMR, $CDCl_3$, 101 MHz

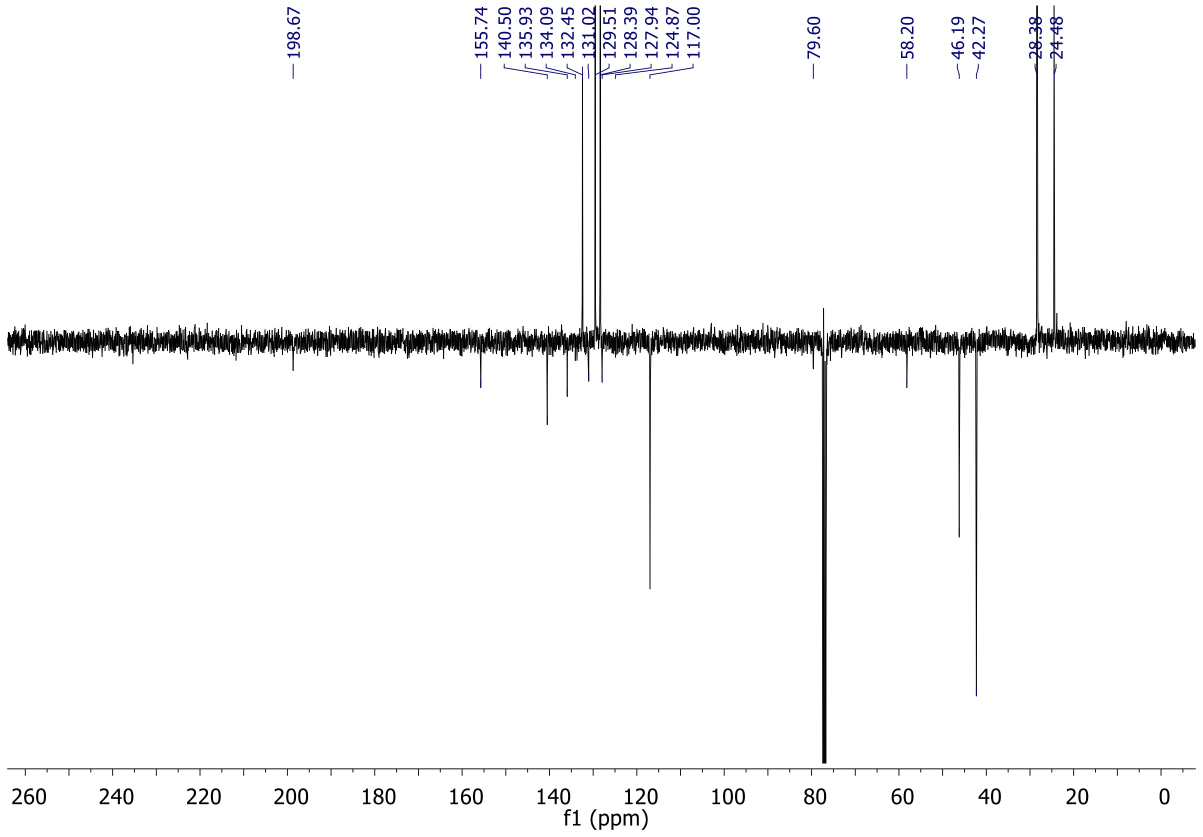

tert-Butyl (2-methylene-5-oxo-4-((trifluoromethyl)thio)heptyl)carbamate (12i)

^{1}H NMR, $CDCl_3$, 400 MHz

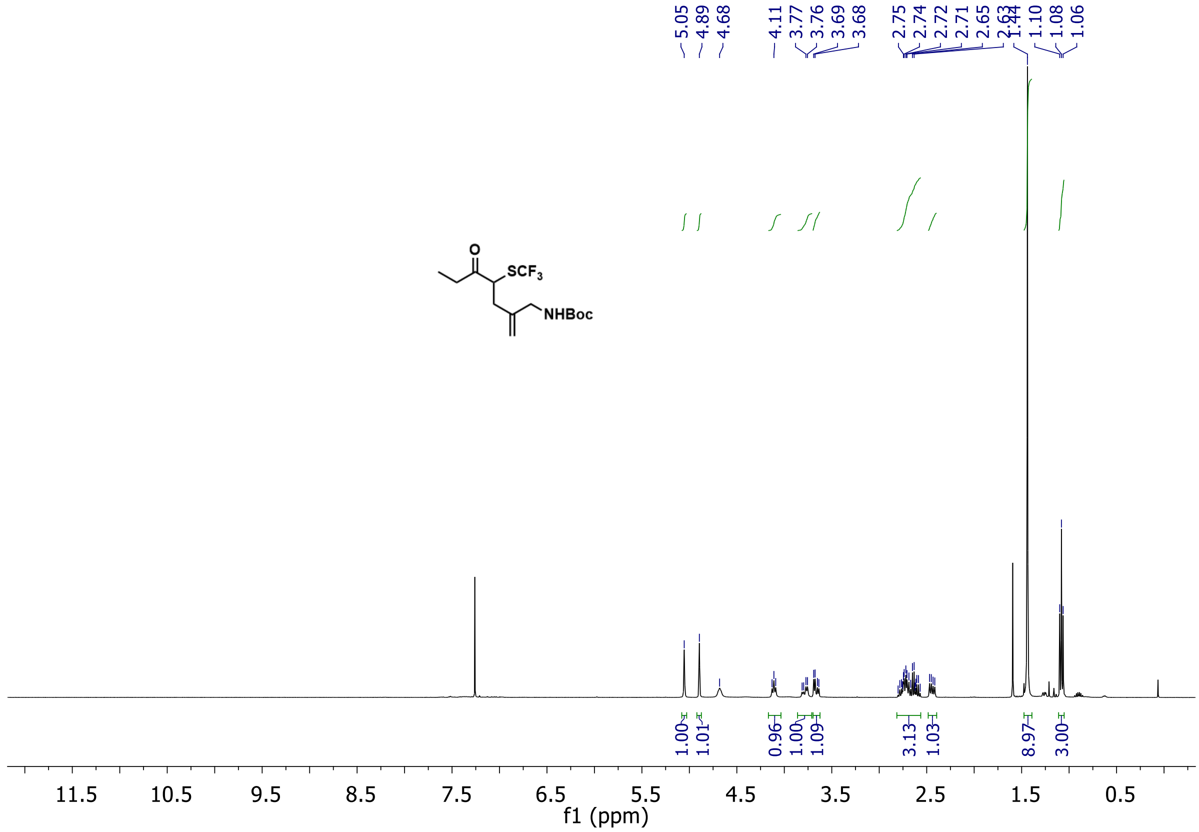

^{19}F NMR, $CDCl_3$, 377 MHz

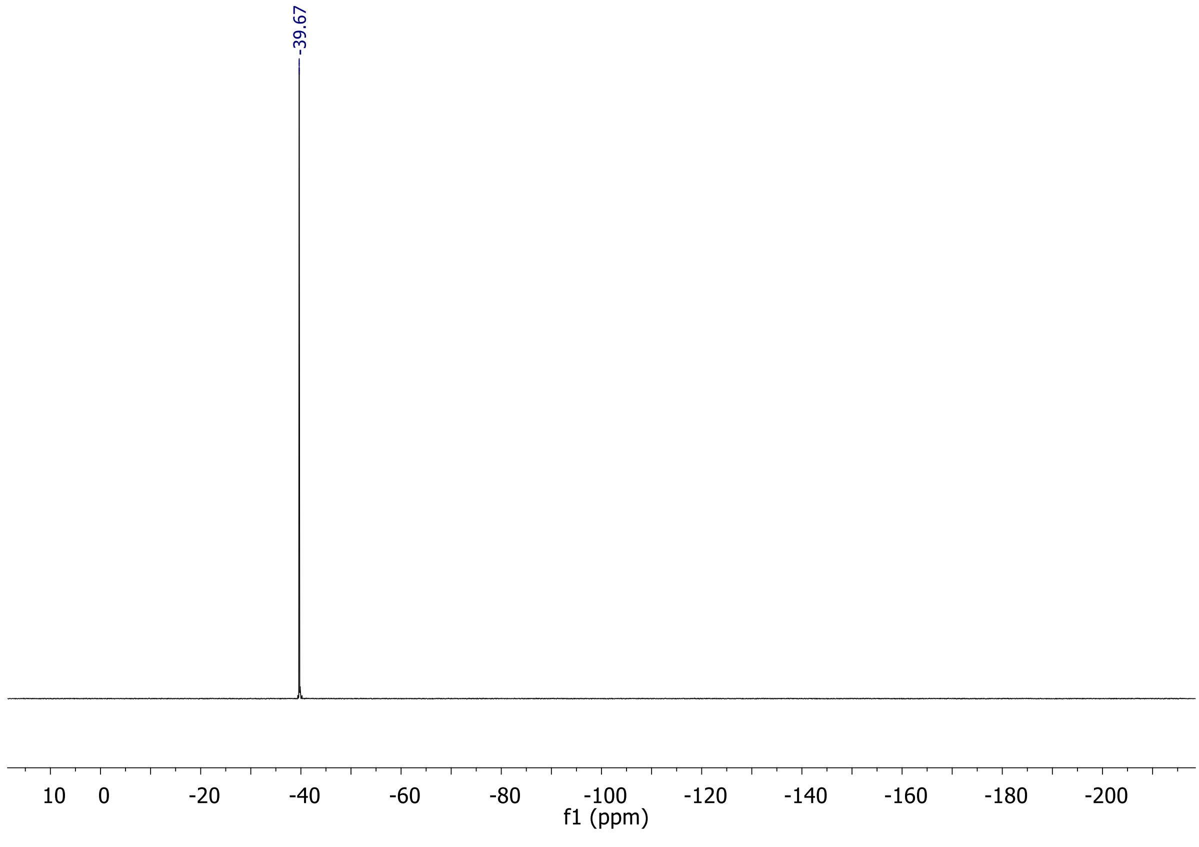

^{13}C NMR, $CDCl_3$, 101 MHz

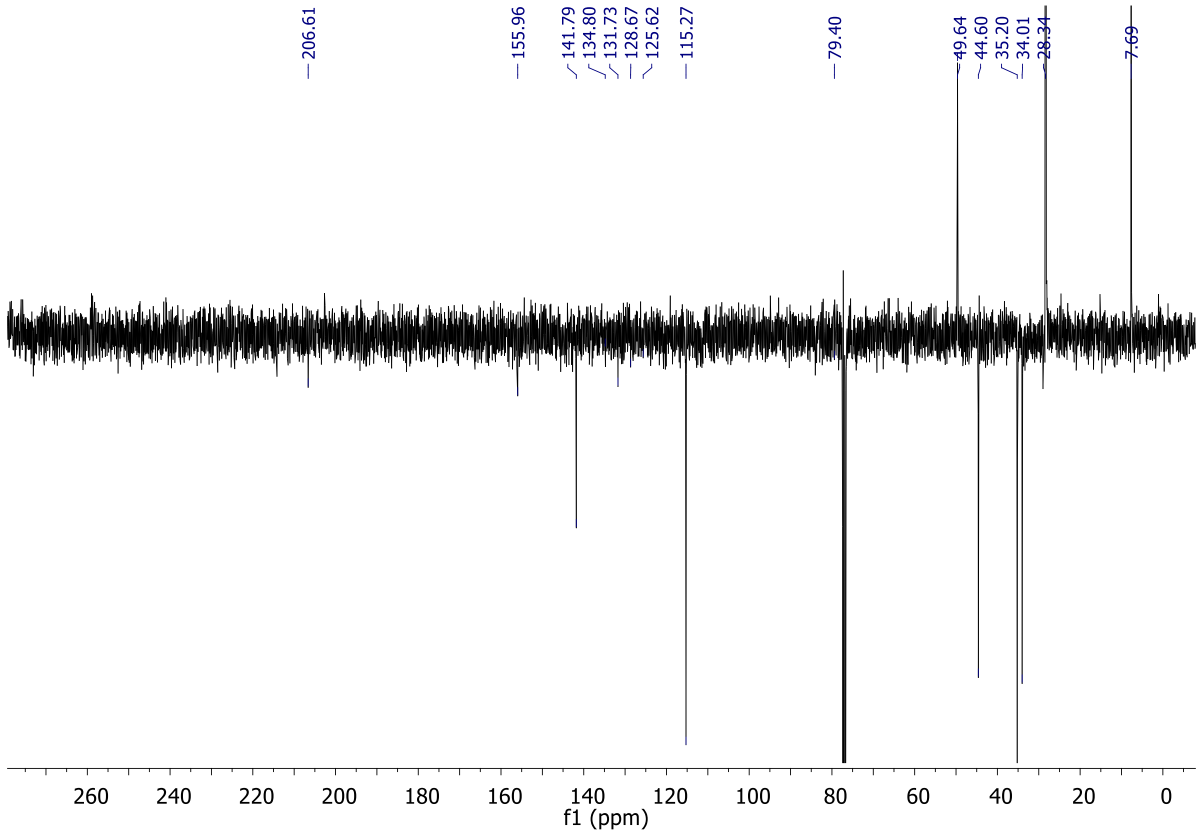

5-Methylene-2-phenyl-3-((trifluoromethyl)thio)piperidine (13a)

^{1}H NMR, $CDCl_3$, 400 MHz

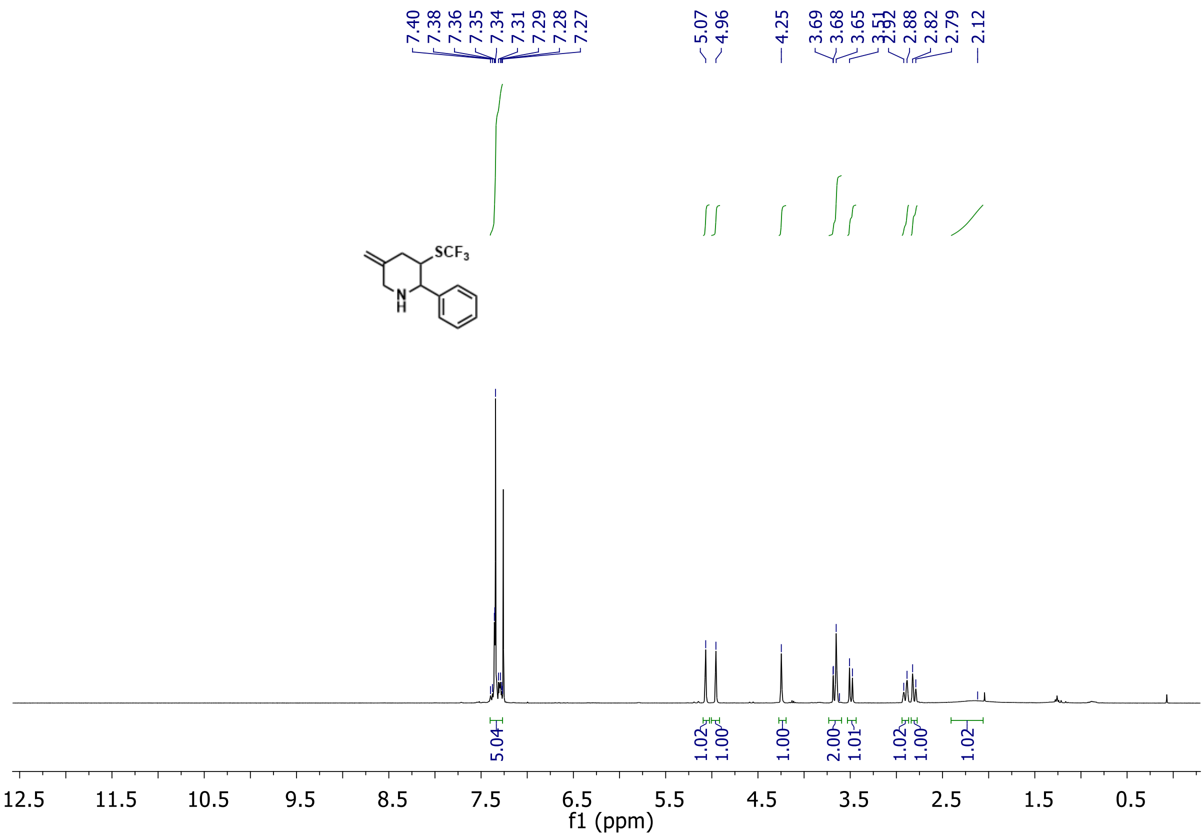

^{19}F NMR, $CDCl_3$, 377 MHz

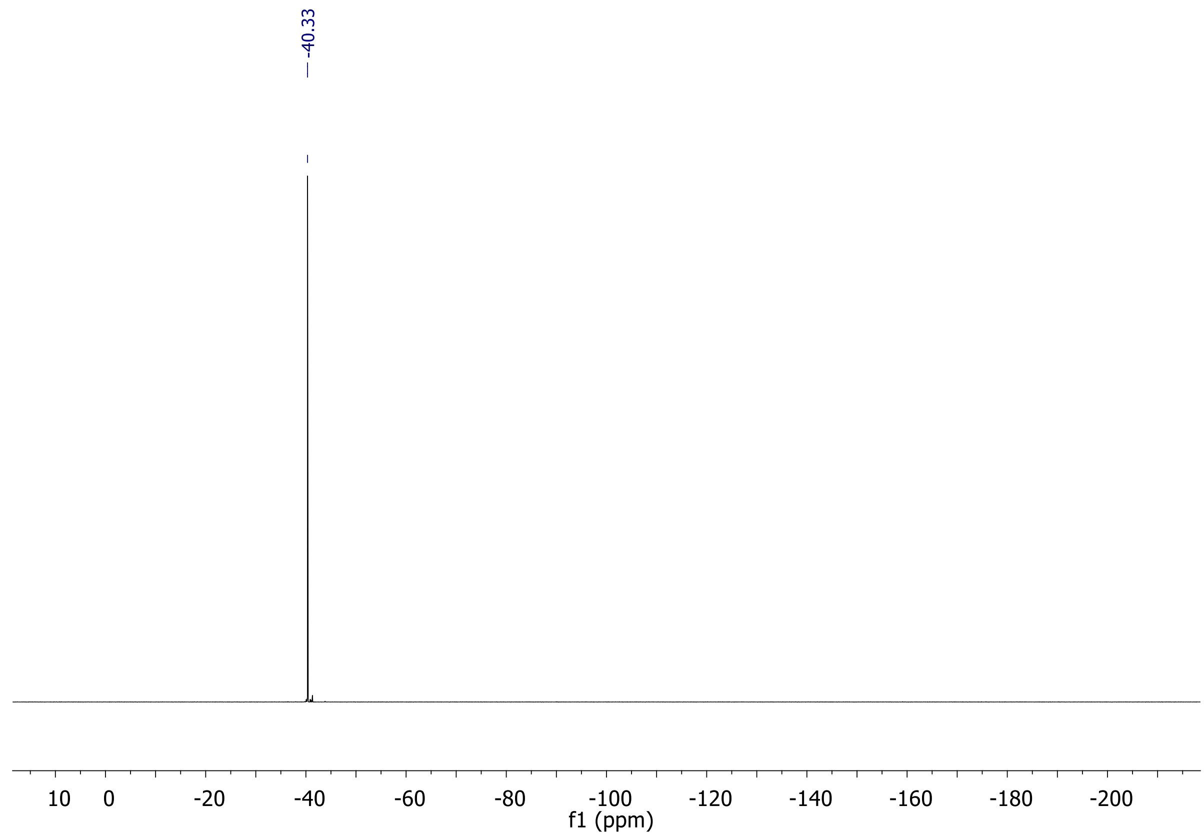

^{13}C NMR, CDCl$_3$, 101 MHz

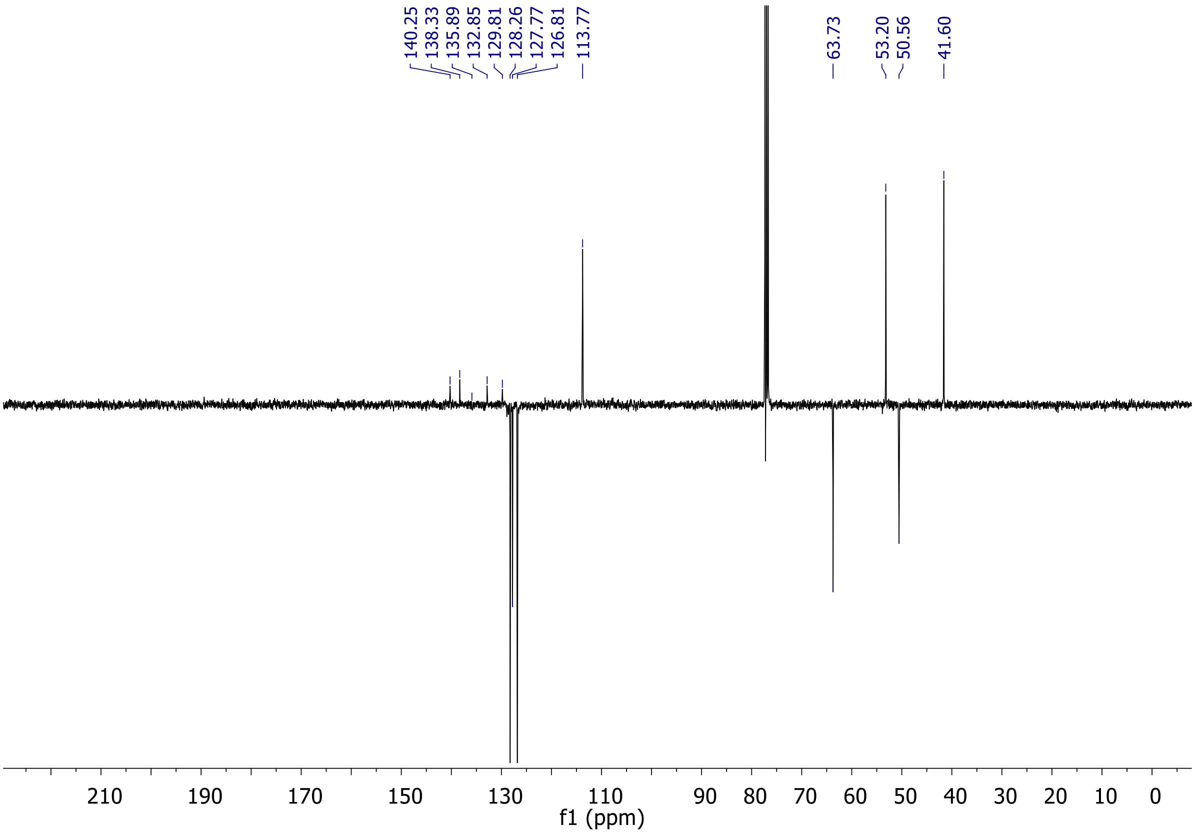

2-(4-Methoxyphenyl)-5-methylene-3-((trifluoromethyl)thio)piperidine (13b)

^{1}H NMR, $CDCl_3$, 400 MHz

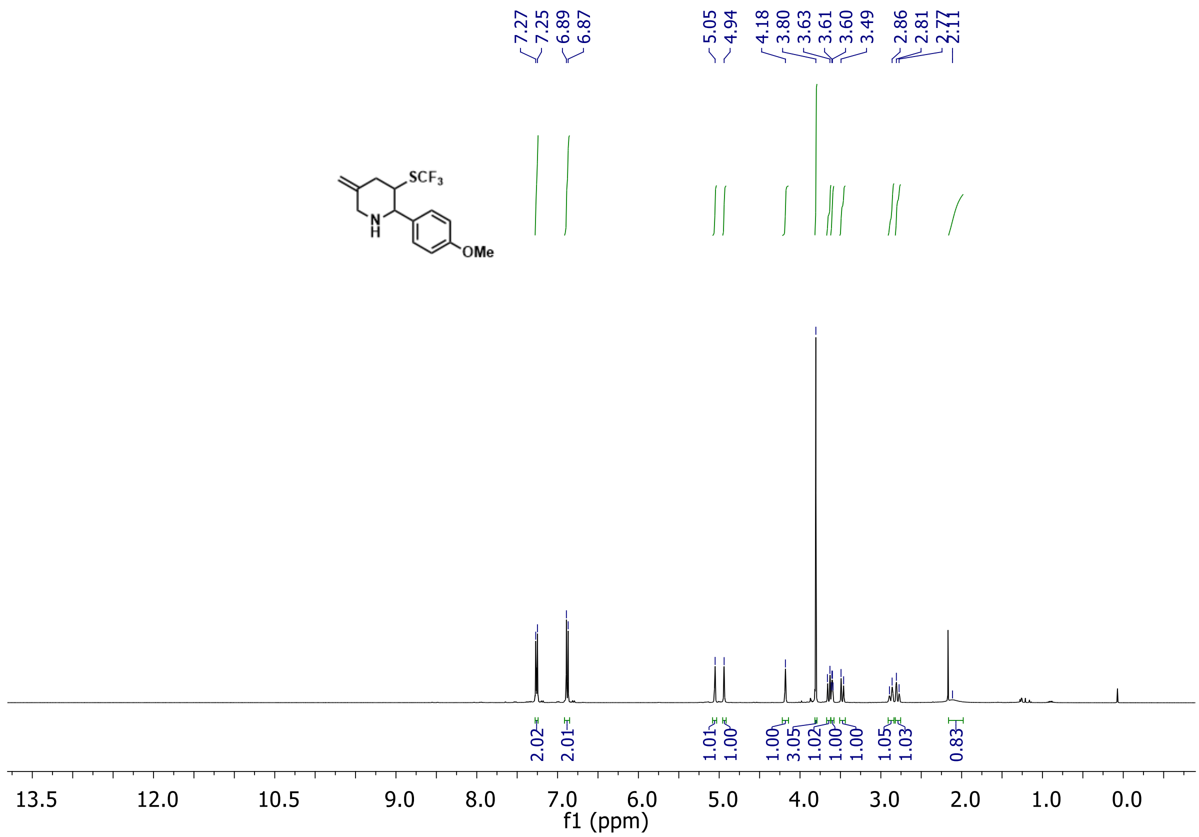

^{19}F NMR, $CDCl_3$, 377 MHz

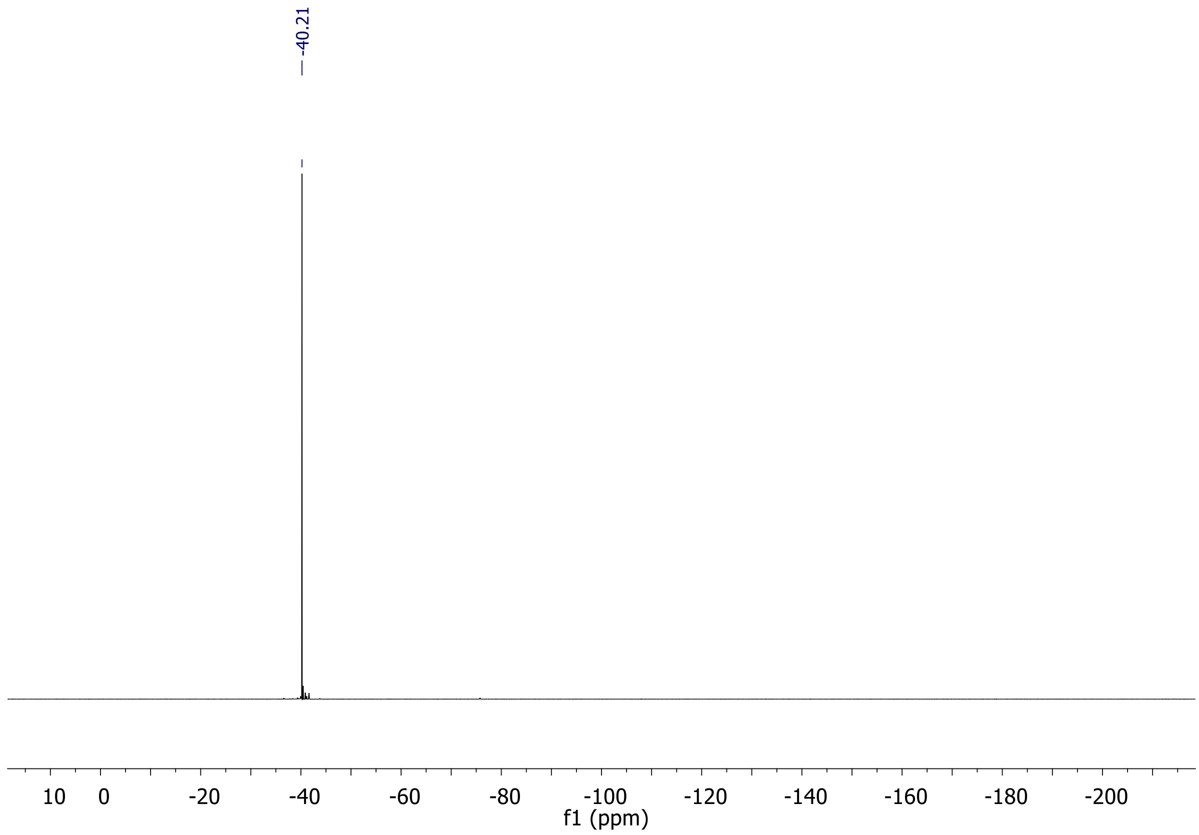

^{13}C NMR, $CDCl_3$, 101 MHz

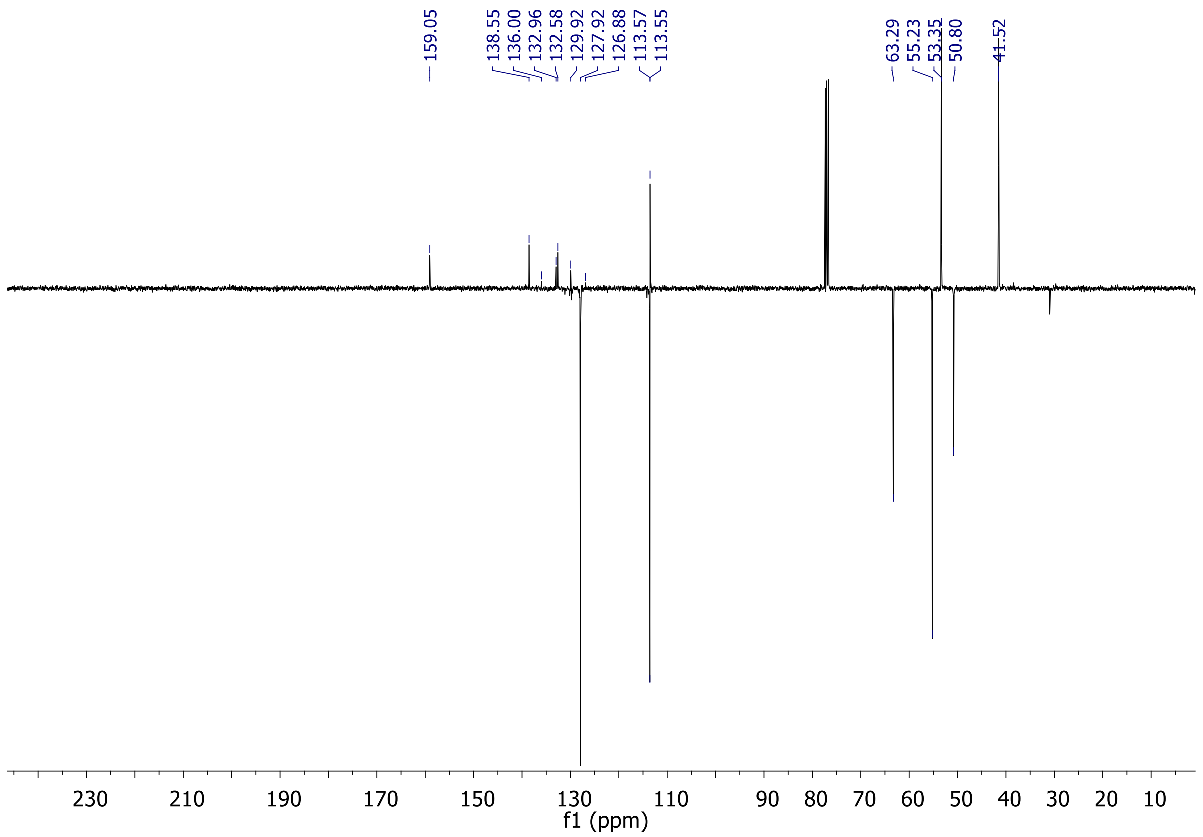

5-Methylene-2-(p-tolyl)-3-((trifluoromethyl)thio)piperidine (13c)

^{1}H NMR, $CDCl_3$, 400 MHz

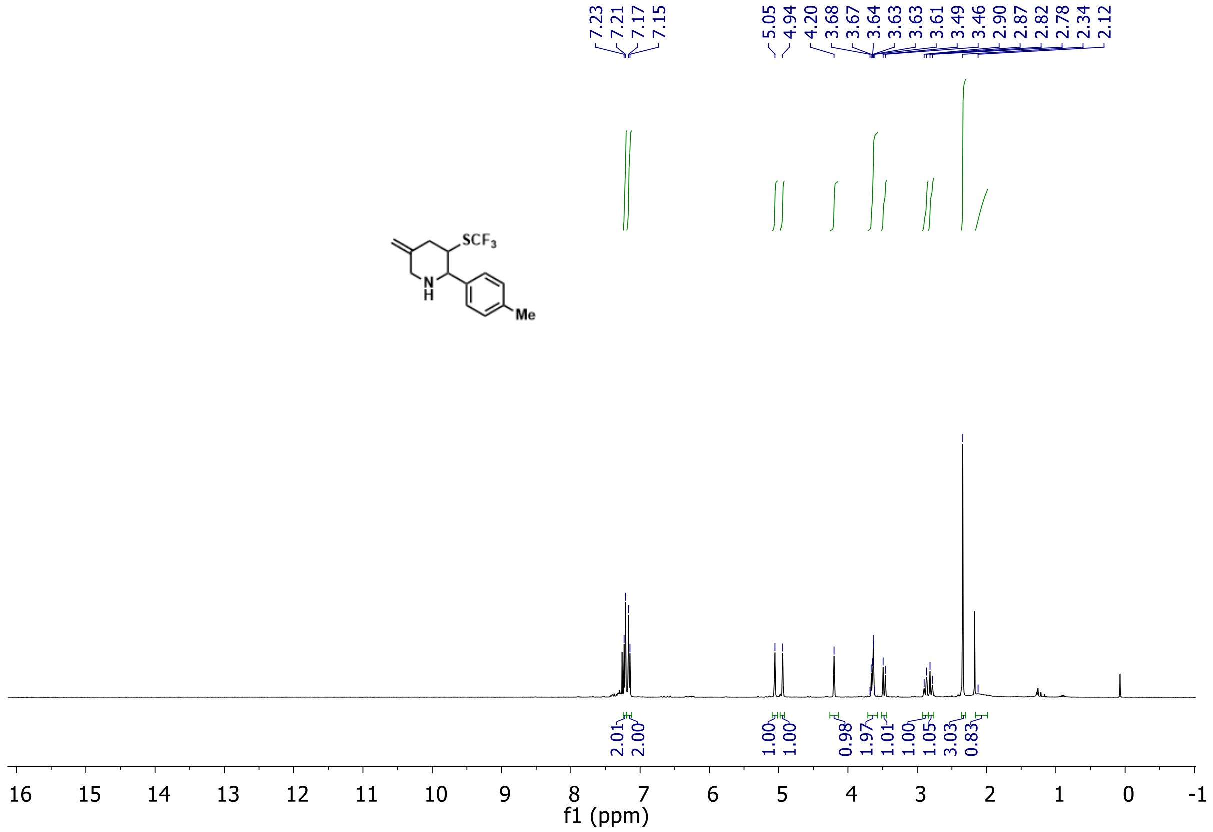

^{19}F NMR, $CDCl_3$, 377 MHz

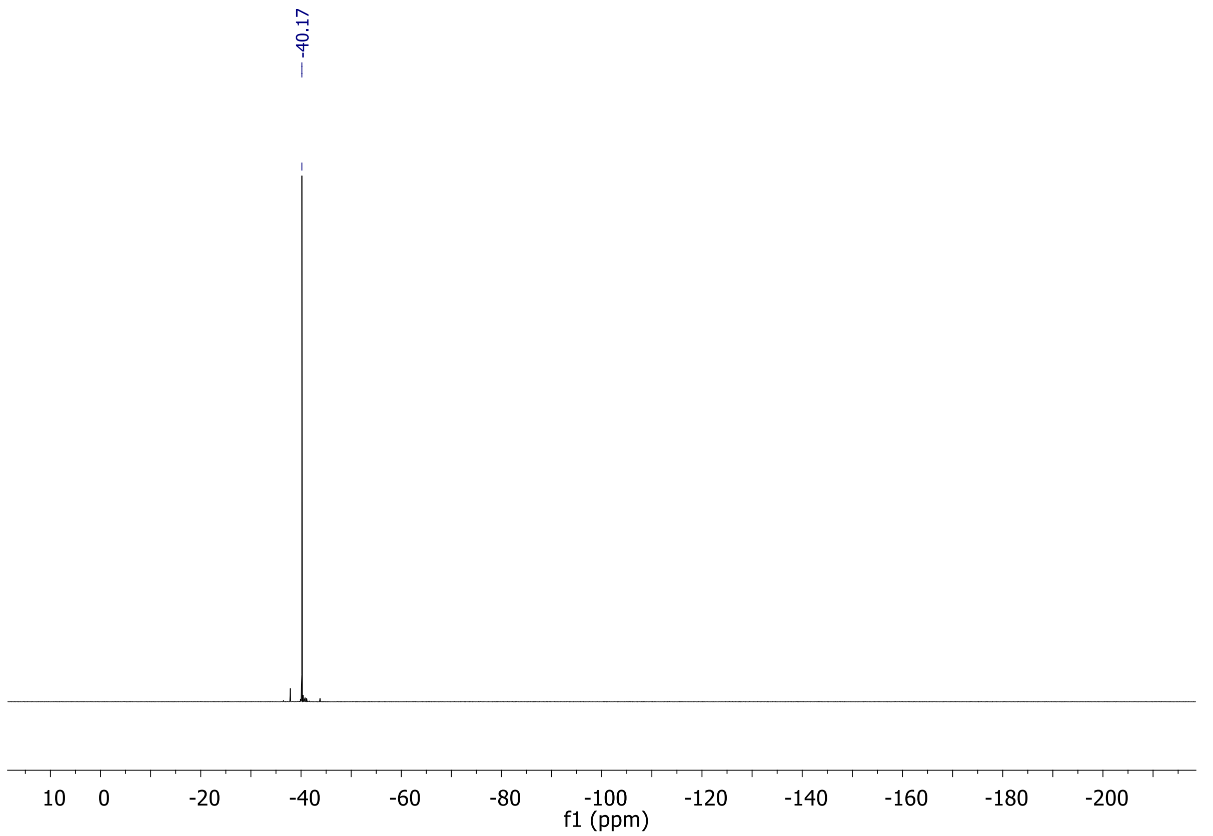

^{13}C NMR, $CDCl_3$, 101 MHz

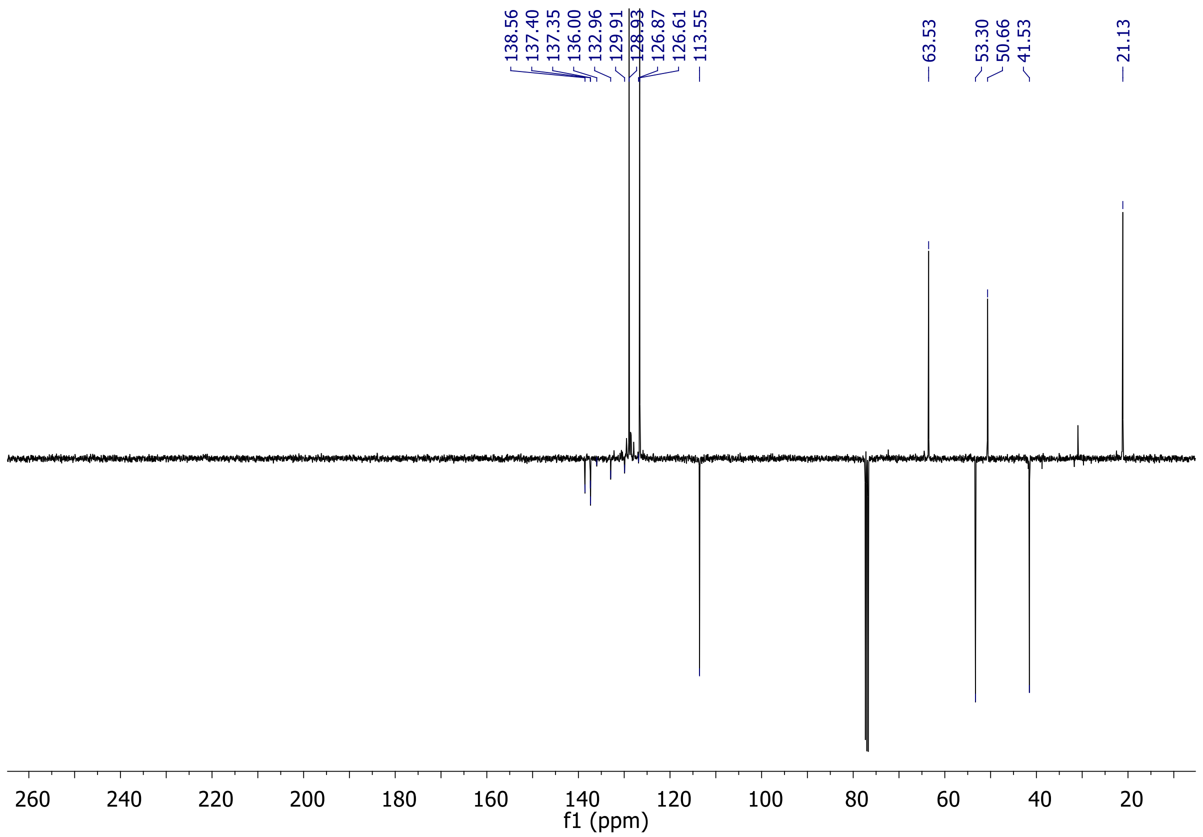

2-(4-Chlorophenyl)-5-methylene-3-((trifluoromethyl)thio)piperidine (13d)

^{1}H NMR, CDCl$_3$, 400 MHz

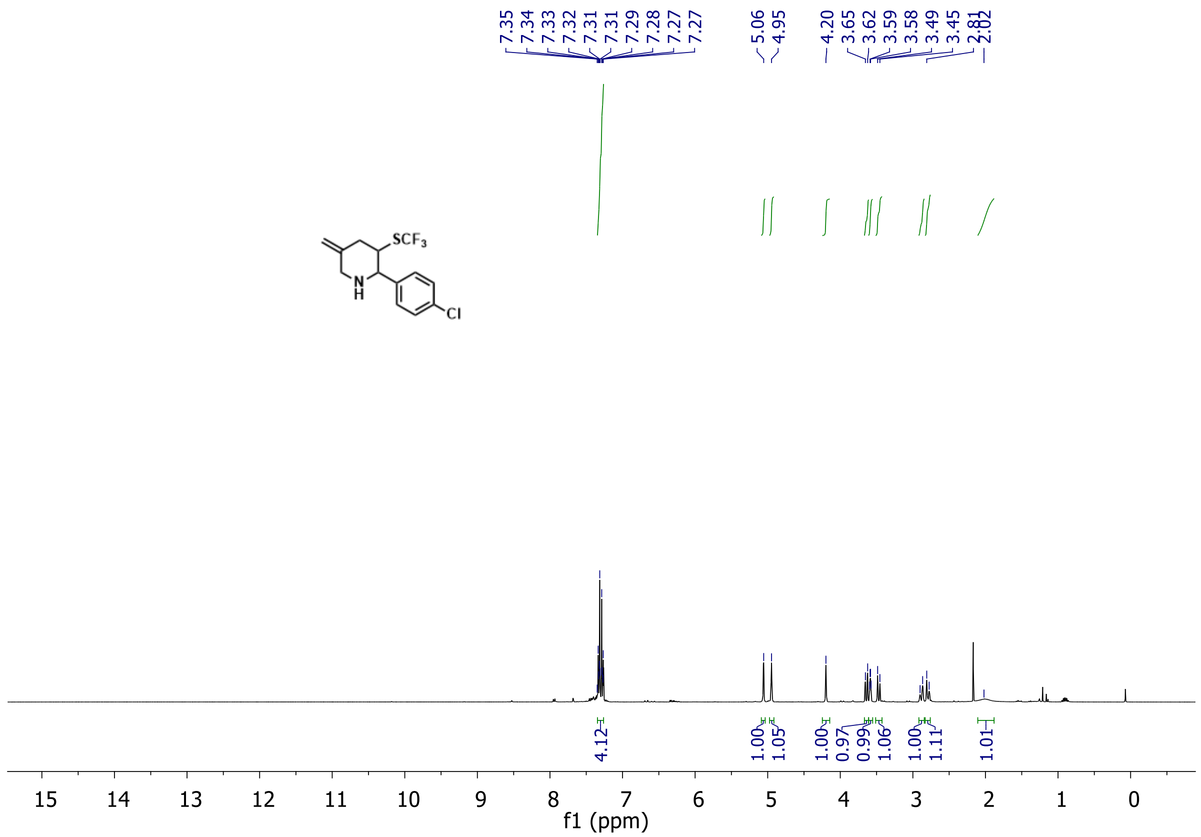

^{19}F NMR, CDCl$_3$, 377 MHz

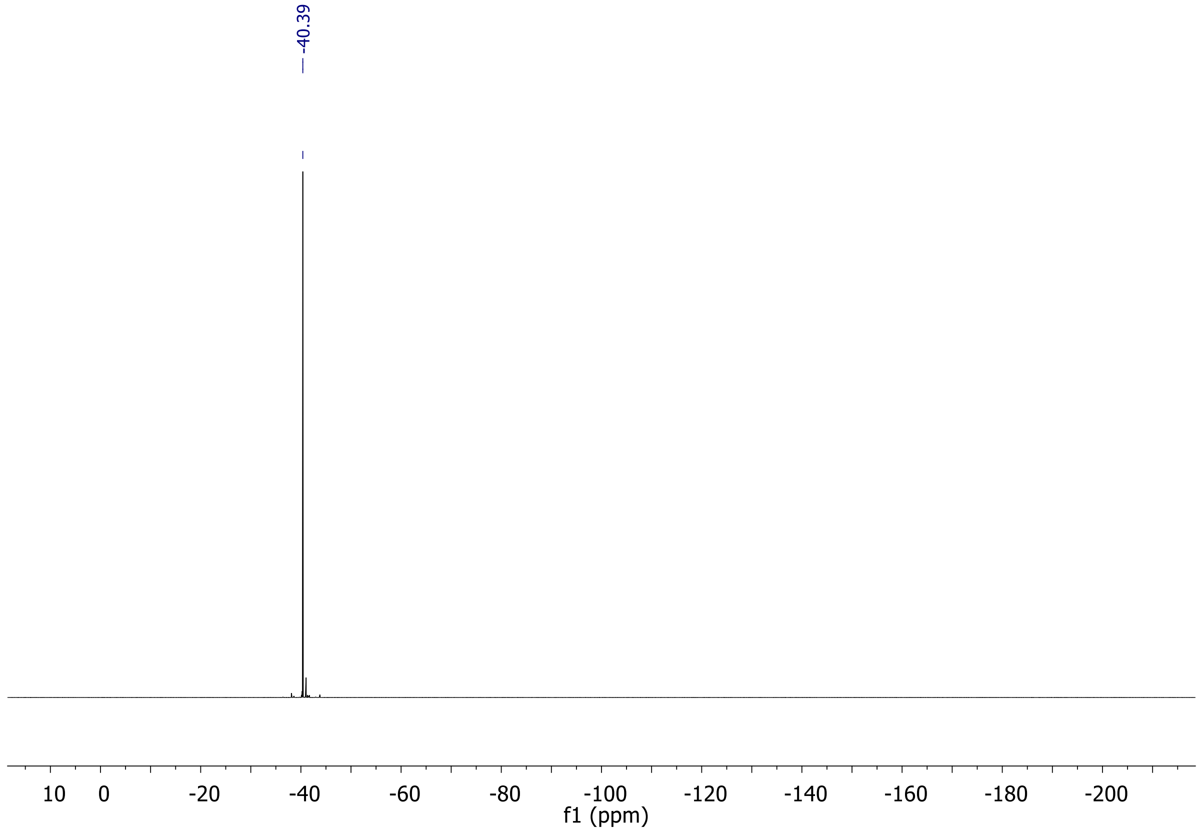

^{13}C NMR, $CDCl_3$, 101 MHz

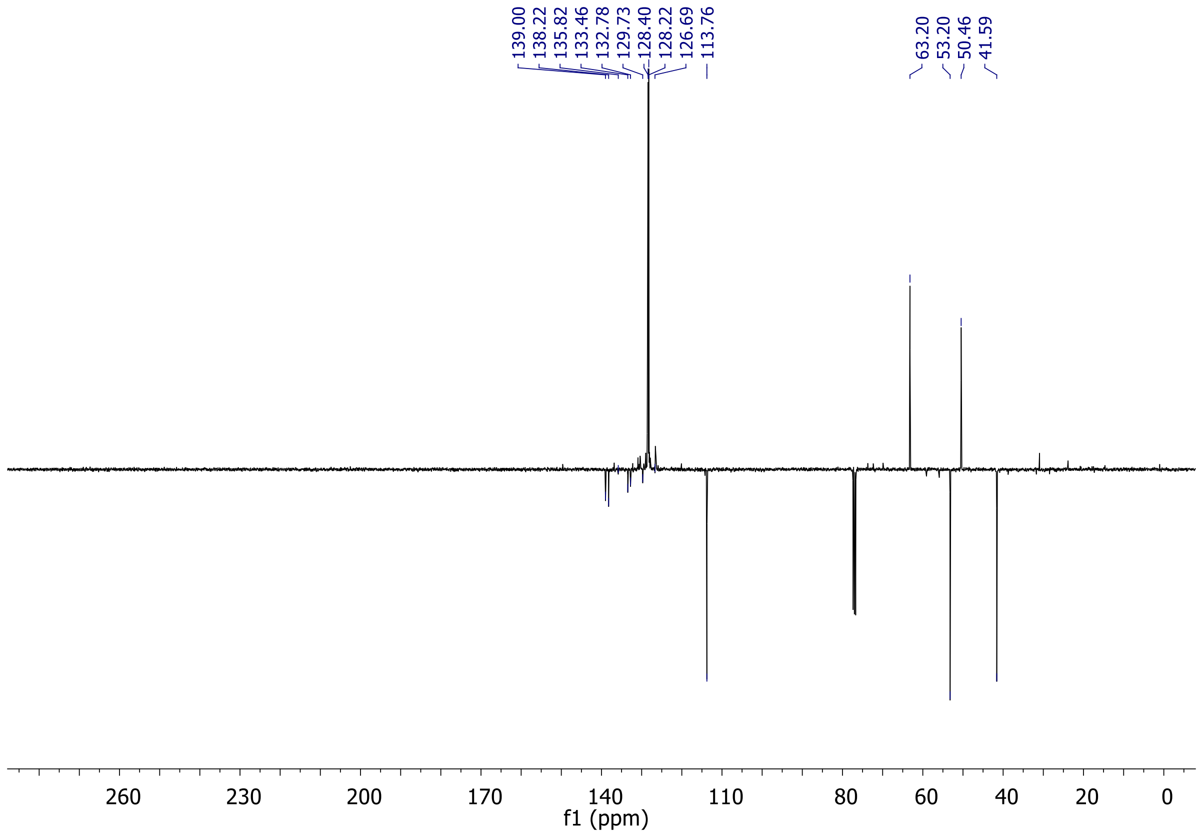

2-(2-Methoxyphenyl)-5-methylene-3-((trifluoromethyl)thio)piperidine (13e)

^{1}H NMR, CDCl$_3$, 400 MHz

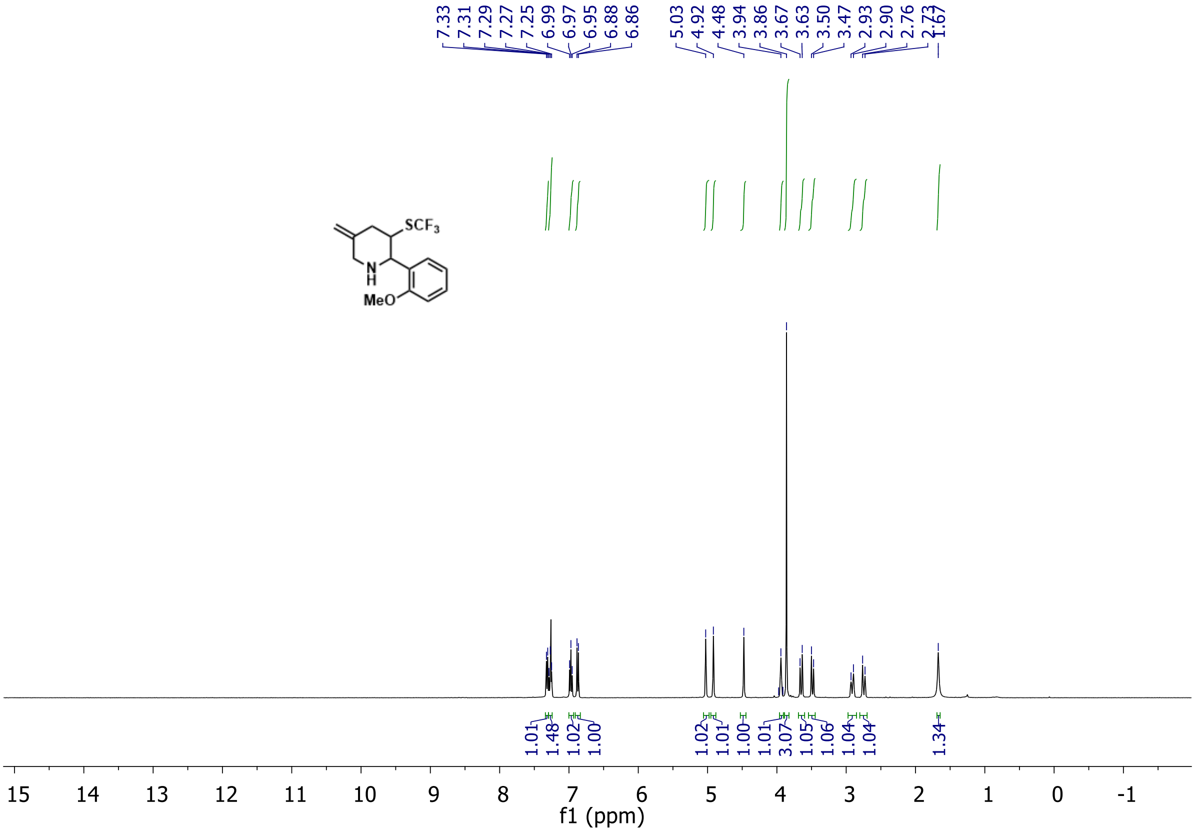

^{19}F NMR, CDCl$_3$, 377 MHz

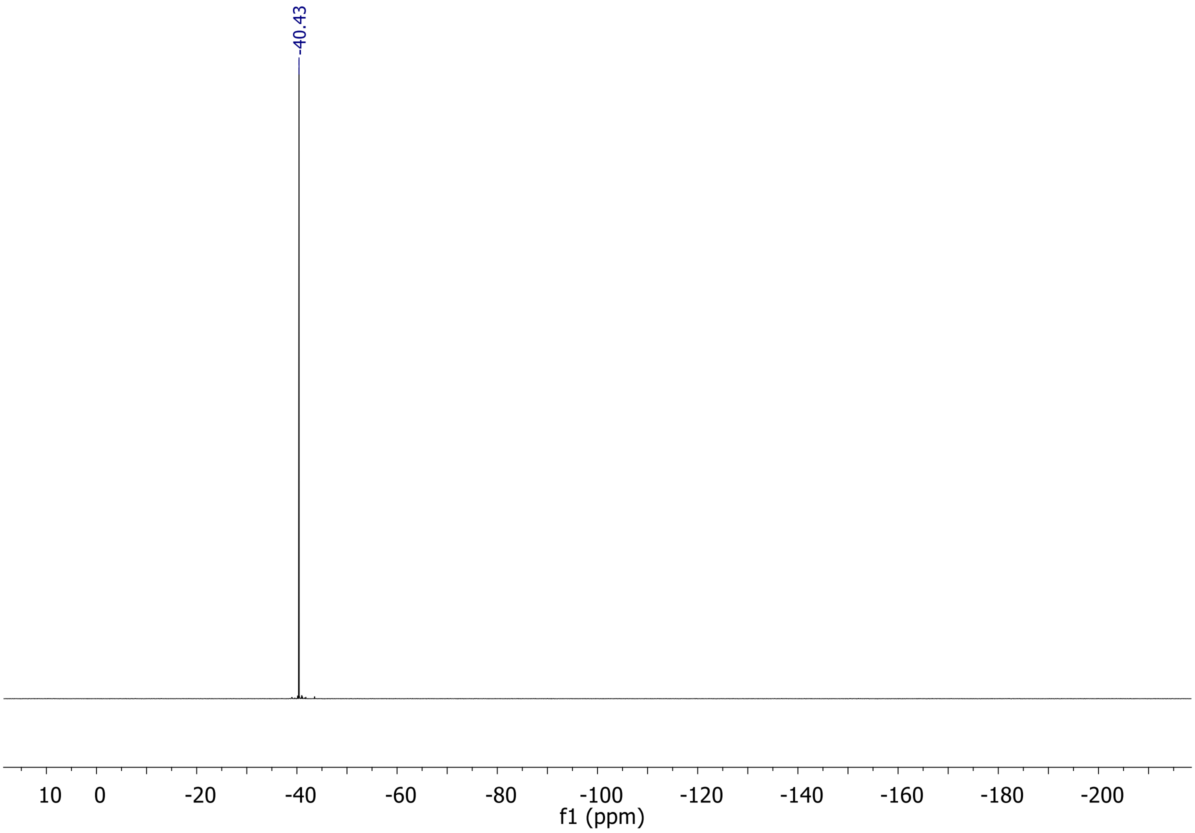

^{13}C NMR, $CDCl_3$, 101 MHz

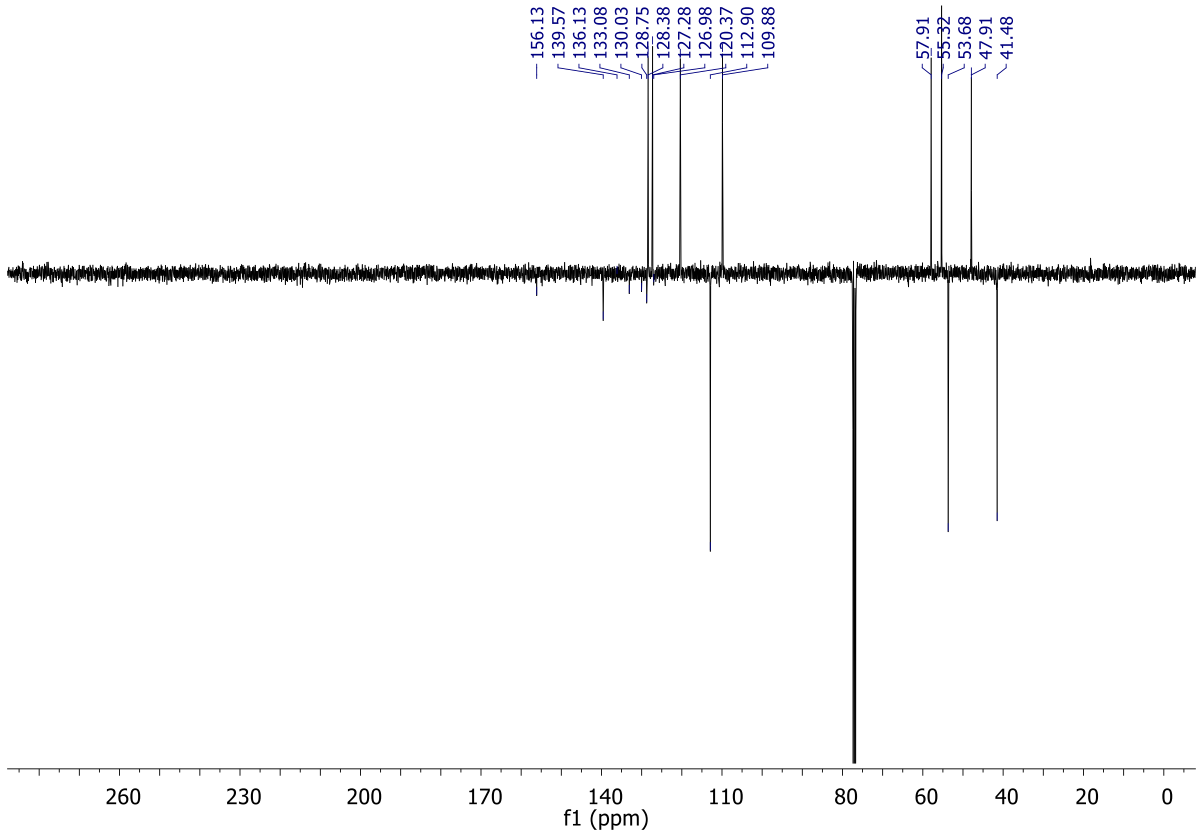

2-(3-Chlorophenyl)-5-methylene-3-((trifluoromethyl)thio)piperidine (13f)

^{1}H NMR, $CDCl_3$, 400 MHz

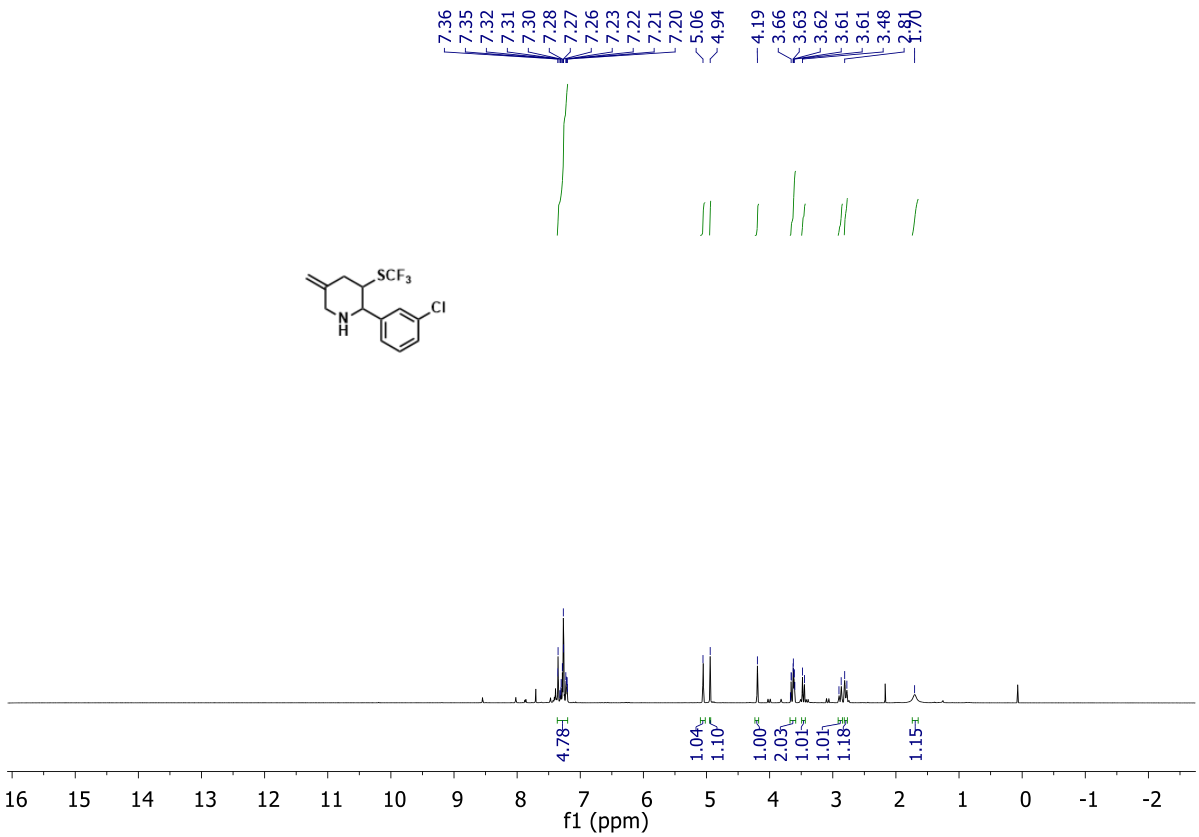

^{19}F NMR, $CDCl_3$, 377 MHz

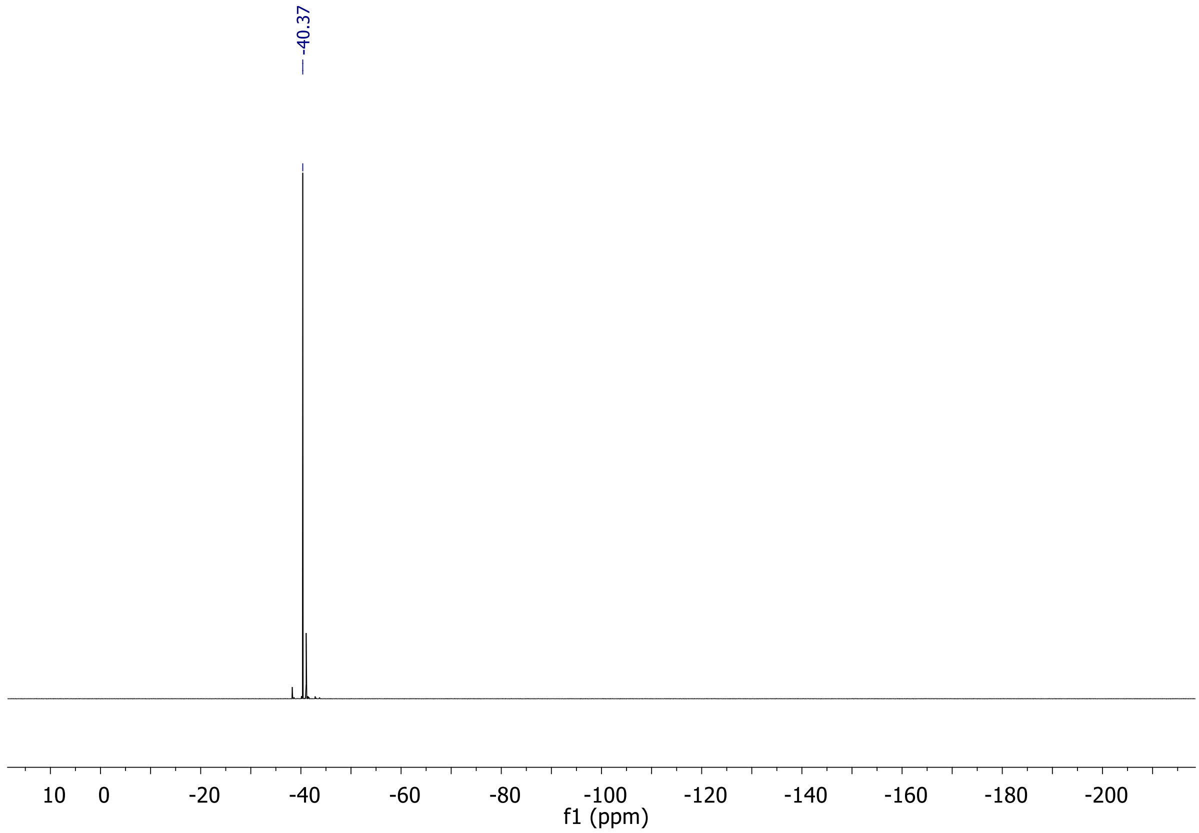

^{13}C NMR, $CDCl_3$, 101 MHz

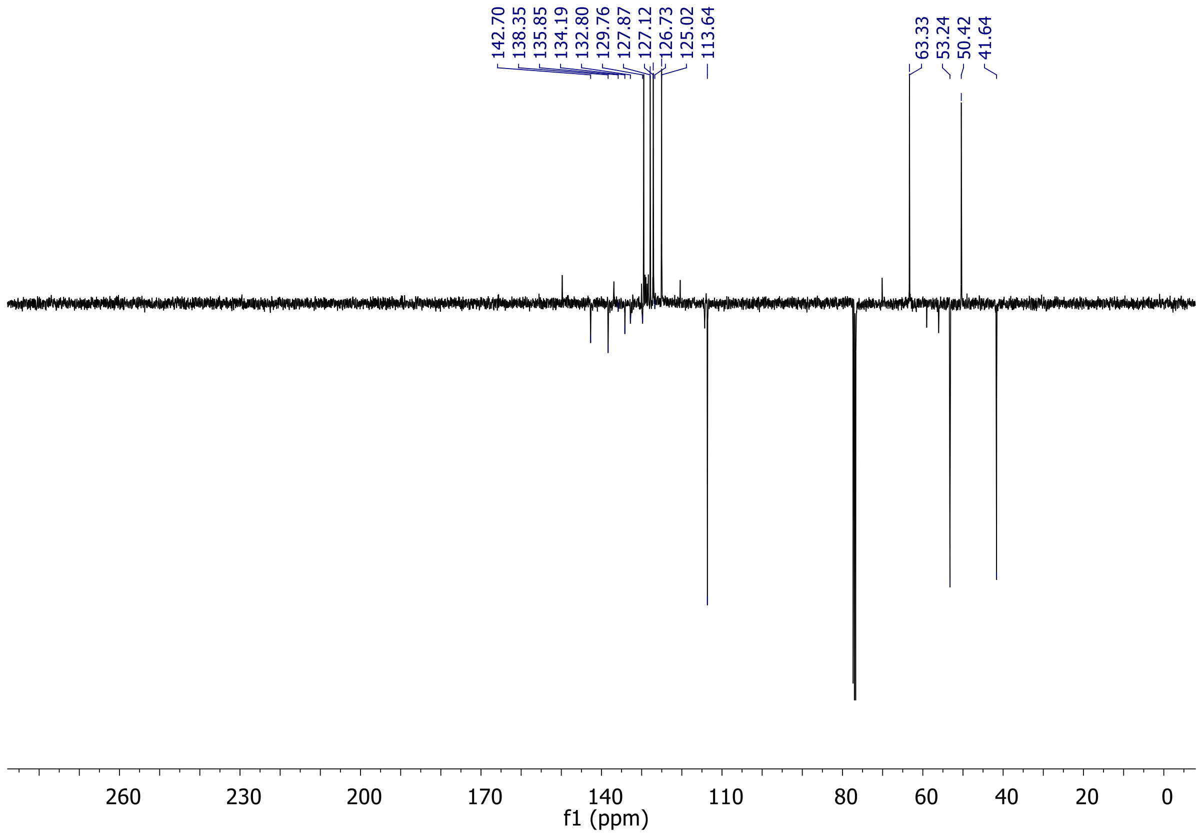

5-Methylene-2-(thiophen-2-yl)-3-((trifluoromethyl)thio)piperidine (13g)

^{1}H NMR, $CDCl_3$, 400 MHz

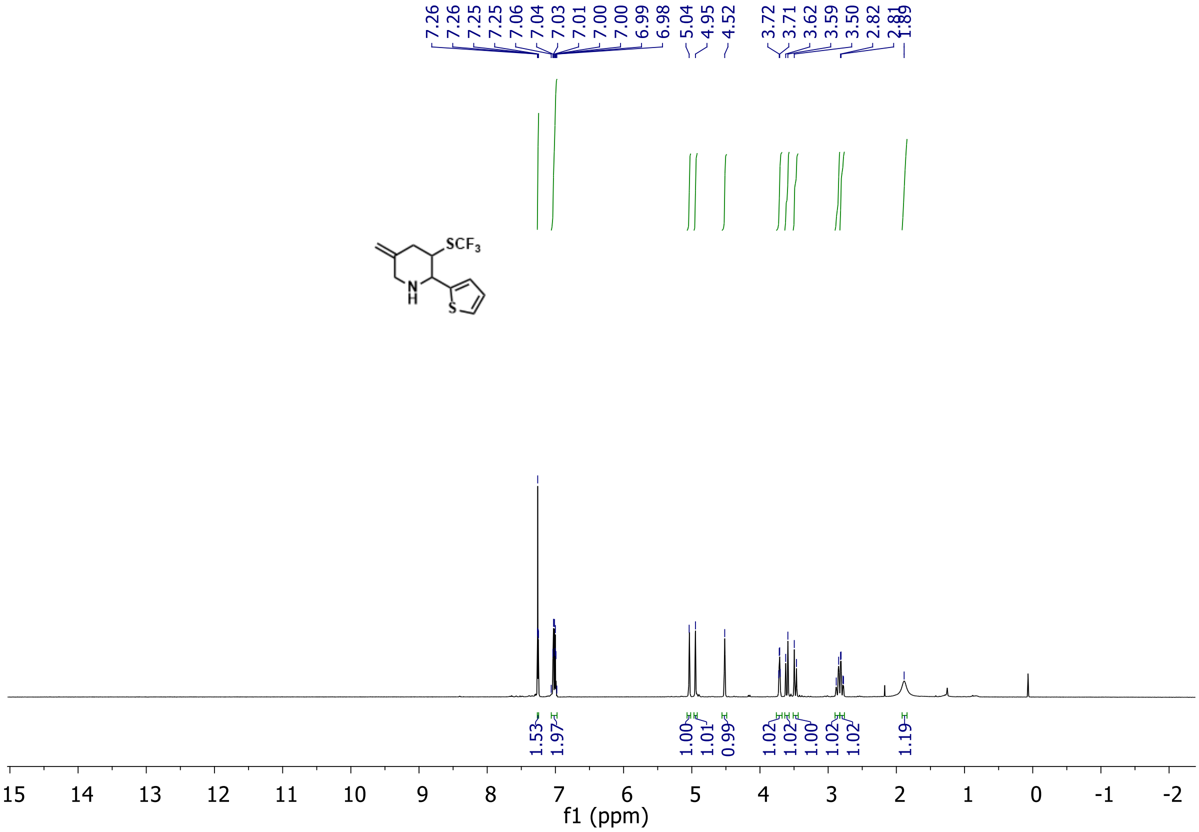

^{19}F NMR, $CDCl_3$, 377 MHz

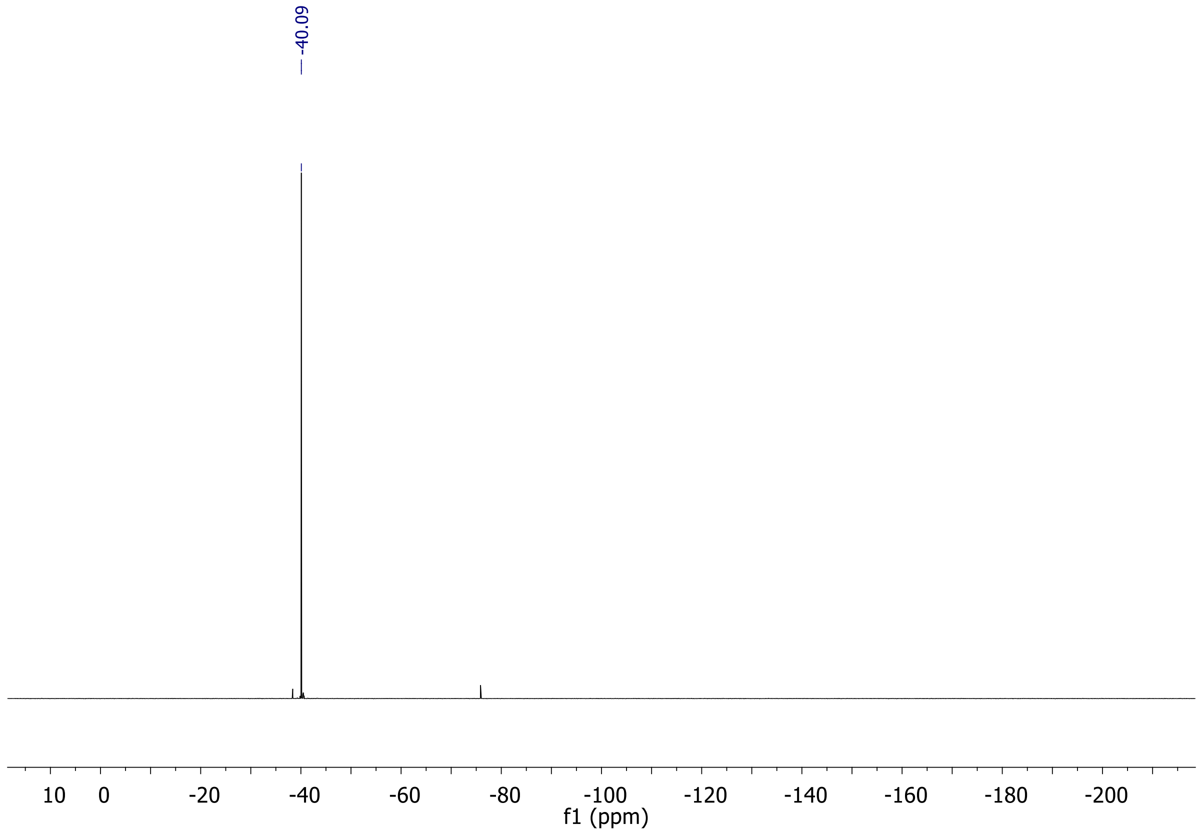

^{13}C NMR, $CDCl_3$, 101 MHz

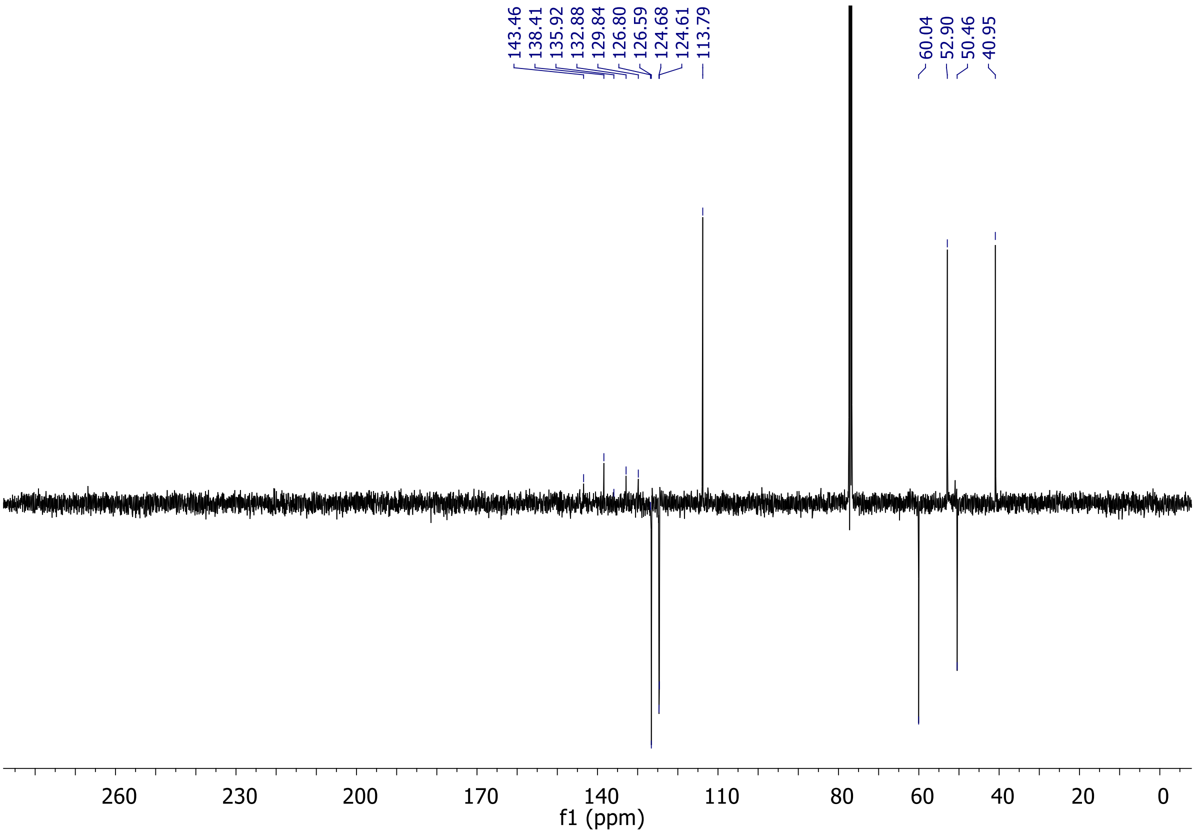

3-Methyl-5-methylene-2-phenyl-3-((trifluoromethyl)thio)piperidine (13h)
^{1}H NMR, CDCl$_3$, 400 MHz

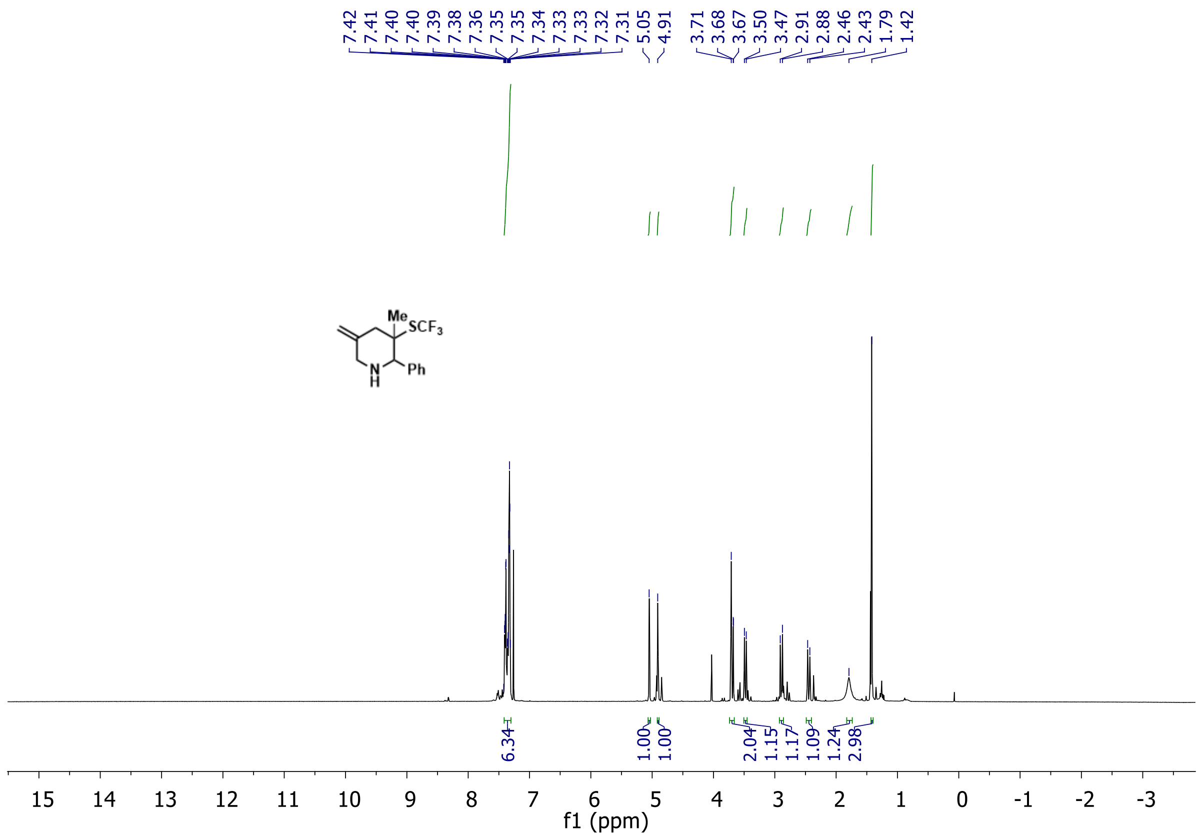

^{19}F NMR, CDCl$_3$, 377 MHz

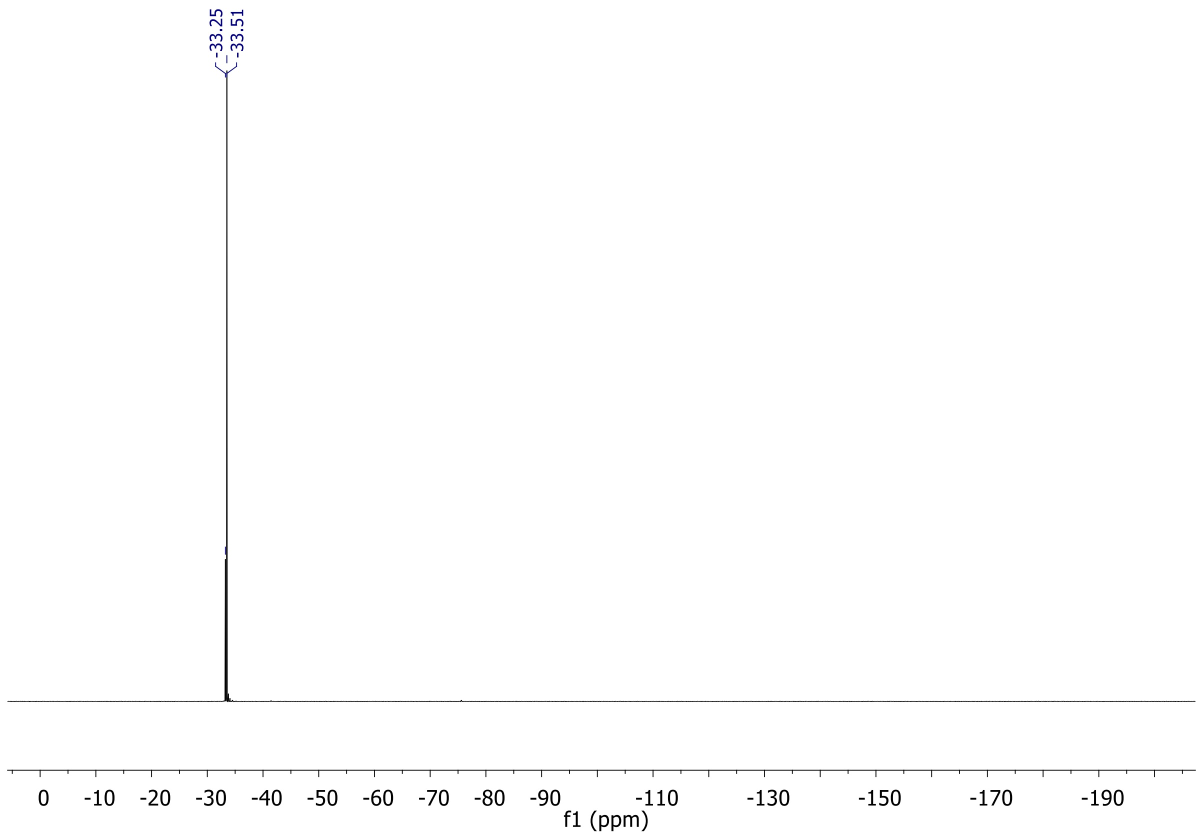

^{13}C NMR, $CDCl_3$, 101 MHz

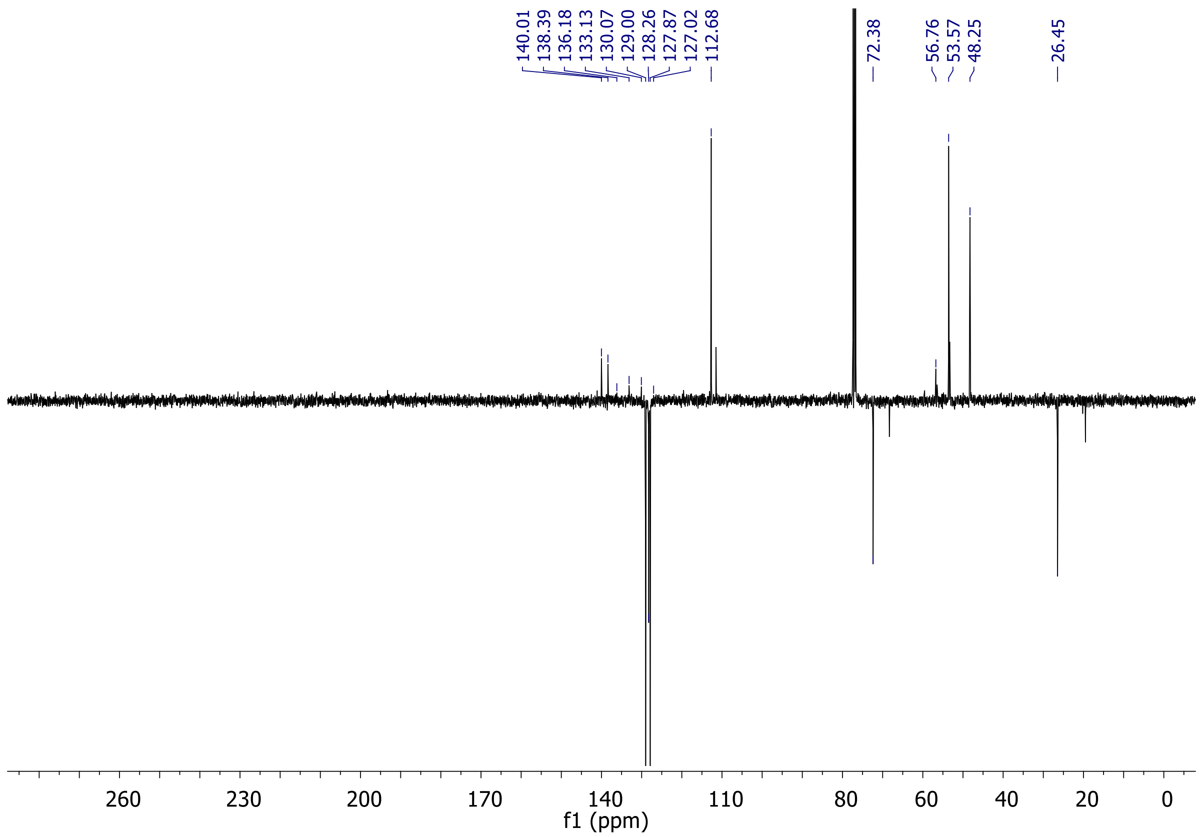

2-Ethyl-5-methylene-3-((trifluoromethyl)thio)piperidine (13i)

^{1}H NMR, $CDCl_3$, 400 MHz

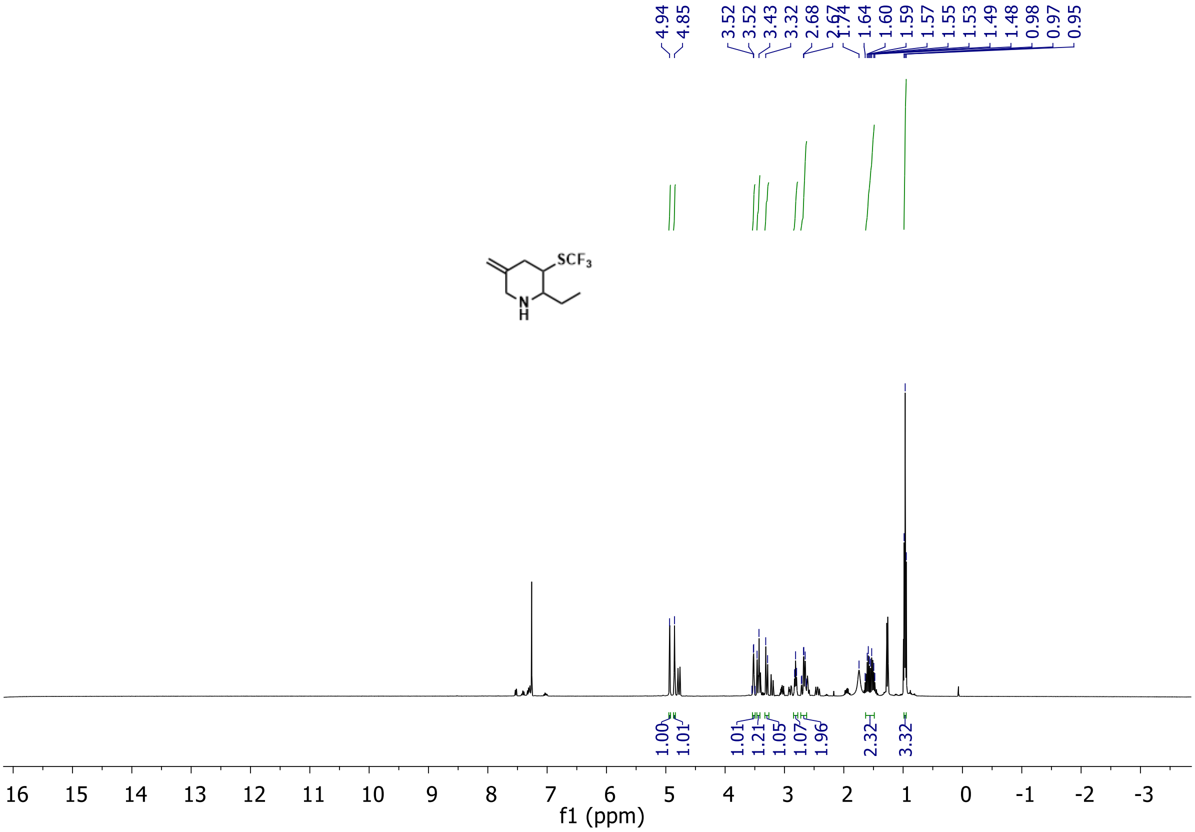

^{19}F NMR, $CDCl_3$, 377 MHz

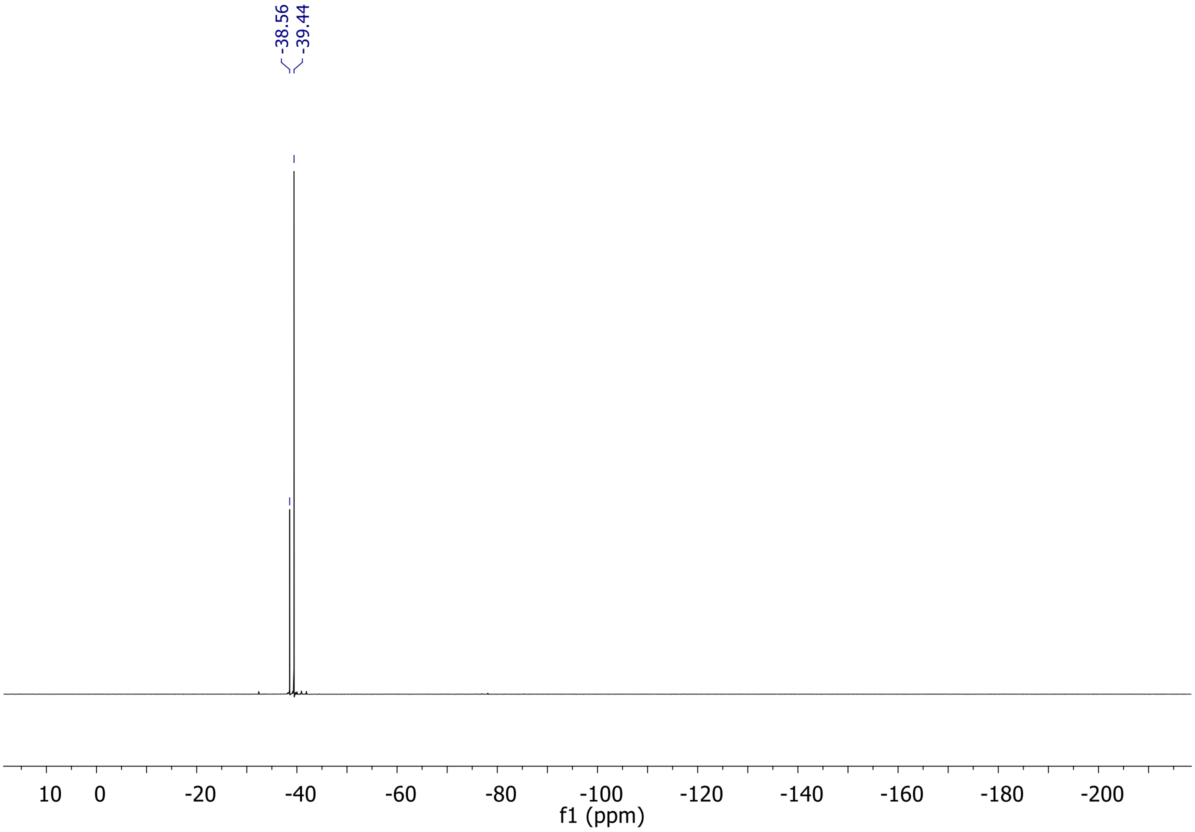

^{13}C NMR, $CDCl_3$, 101 MHz

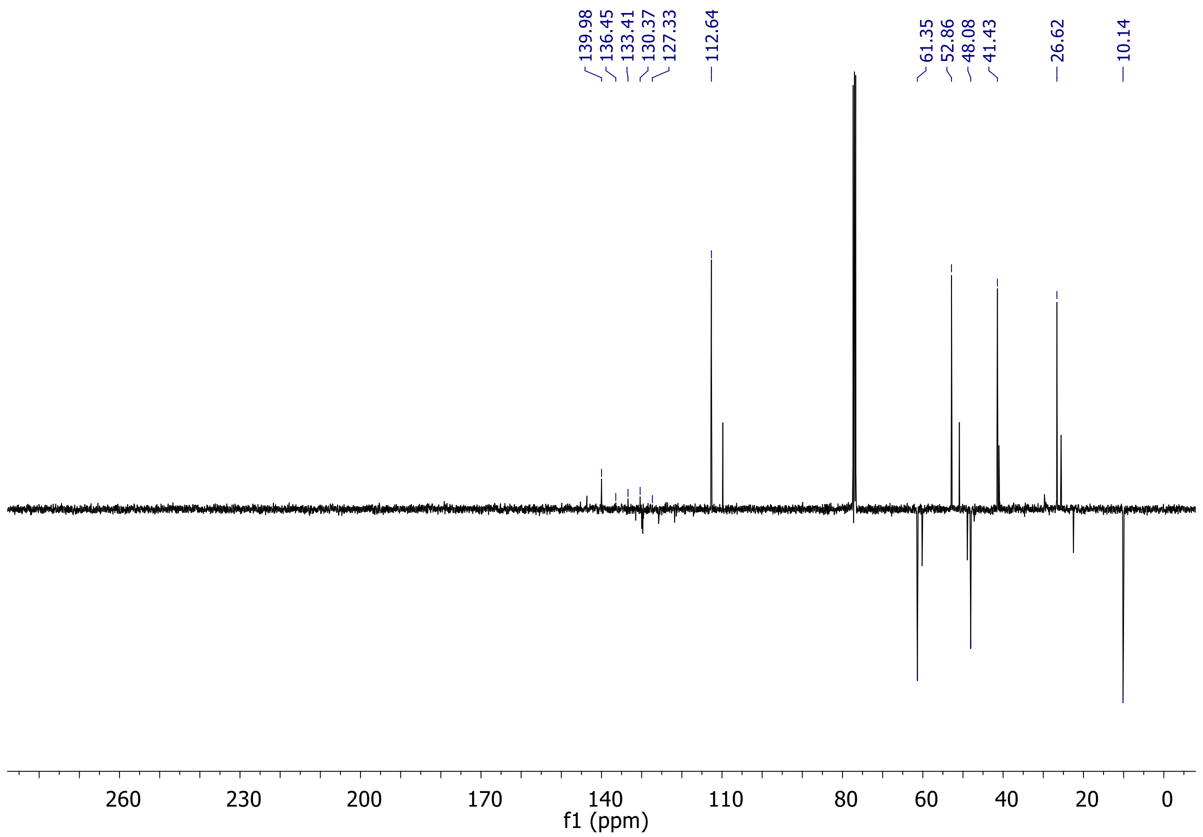

Stereochemical Assignment of 9

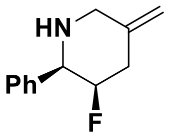

9

^{1}H NMR (400 MHz, CDCl$_3$) δ 7.39 – 7.21 (m, 5H), 4.97 – 4.79 (m, 3H), 3.87 (d, *J* = 30.0 Hz, 1H), 3.63 (dd, *J* = 14.0, 1.5 Hz, 1H), 3.46 (d, *J* = 14.0 Hz, 1H), 2.84 – 2.75 (m, 1H), 2.56 (dd, *J* = 45.0, 15.0 Hz, 1H), 1.93 (s, 1H); **^{13}C NMR (101 MHz, CDCl$_3$)** δ 140.3, 140.0 128.5, 127.6, 127.2, 112.1, 90.4 (d, *J* = 178.5 Hz), 62.7 (d, *J* = 19.0 Hz), 53.1, 38.9 (d, *J* = 23.5 Hz); **^{19}F NMR (377 MHz, CDCl$_3$)** δ -197.47 (dddd, *J* = 48.5, 45.0, 30.0, 11.0 Hz); **FTIR:** ν_{max}/cm^{-1} (neat) 3291, 2937, 1656, 1467, 1261, 1100, 1022 cm^{-1}; **HRMS (ESI$^+$):** calculated for $C_{12}H_{15}FN$ (ES$^+$)(+H$^+$): 192.1183. Found: 192.1186.

The assignment of **9** as containing a relative *cis*-stereochemistry between the fluorine atom and phenyl ring is made on the basis of the magnitude of the proton-fluorine coupling constants in the ^{1}H and ^{19}F NMR spectra. Examination of the current literature reveals a clear trend between molecular conformation and the magnitude of proton-fluorine coupling constants within six membered rings.[17-21] Typically, geminal ($^{2}J_{F\text{-}H}$) and *trans*-diaxial ($^{3}J_{F(ax)\text{-}H(ax)}$) couplings are much larger than the corresponding axial-equatorial ($^{3}J_{F(ax)\text{-}H(eq)}$ or $^{3}J_{F(eq)\text{-}H(ax)}$) and equatorial-equatorial ($^{3}J_{F(eq)\text{-}H(eq)}$) couplings (Figure S1). In the case of **9**, 4 different 3-Dimensional conformations are possible (Scheme S1). Analysis of the ^{1}H and ^{19}F NMR spectra of **9** indicates that there is a geminal coupling (48.5 Hz), 2 *trans*-diaxial couplings (45.0 and 30.0 Hz) and one axial-equatorial or equatorial-equatorial coupling (11.0 Hz). Given that only conformation **B1** would give rise to NMR spectra matching the observed spectra, we assigned the fluorine atom and phenyl ring in **9** in a *cis*-relationship relative to one another.

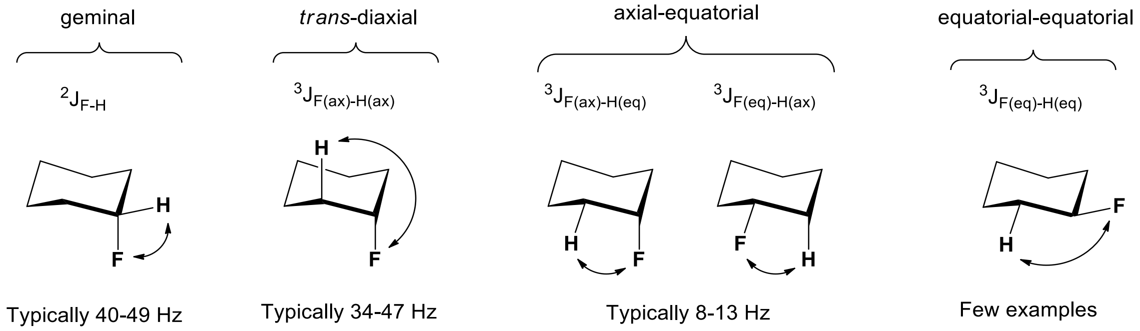

Figure S1 – Typical ranges for proton-fluorine coupling constants reported in the chemical literature

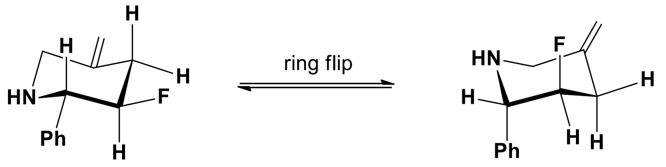

A1 **A2**

1 x geminal coupling
2 x axial-equatorial coupling
1 x equatorial-equatorial coupling

1 x geminal coupling
1 x *trans*-diaxial coupling
2 x axial-equatorial coupling

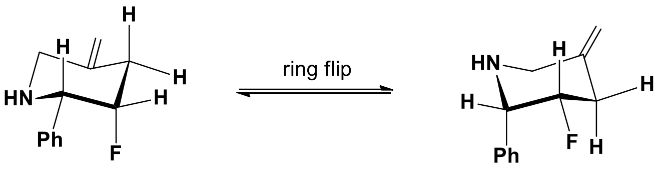

B1 **B2**

1 x geminal coupling
2 x *trans*-diaxial coupling
1 x axial-equatorial coupling

1 x geminal coupling
1 x axial-equatorial coupling
2 x equatorial-equatorial coupling

Scheme S1 – Possible conformations of **9** and their associated proton-fluorine couplings

Crystallographic Data

Crystals suitable for X-ray single-crystal structure determination were grown by slow evaporation of a solution in chloroform. Intensity data for compounds **13a** and **13b** were collected on a Bruker D8 Venture diffractometer equipped with a Photon 100 CMOS detector using a CuKα microfocus X-ray source. Intensity data for compound **6** was collected on a Bruker SMART APEX-II CCD operating with a MoKα sealed-tube X-ray source. Data were collected at 100 K from crystals mounted in fomblin oil on a MiTiGen microloop and cooled in a stream of cold N_2. Data were corrected for absorption using empirical methods (SADABS)[22] based upon symmetry equivalent reflections combined with measurements at different azimuthal angles.[23] The crystal structures were solved and refined against F^2 values using ShelXT[24] for solution and ShelXL[25] for refinement accessed via the Olex2 program.[26] Non-hydrogen atoms were refined anisotropically. Hydrogen atoms were placed in calculated positions with idealized geometries and then refined by employing a riding model and isotropic displacement parameters.

X-ray crystallographic analysis for compound 6

Deposition number CCDC 2063492

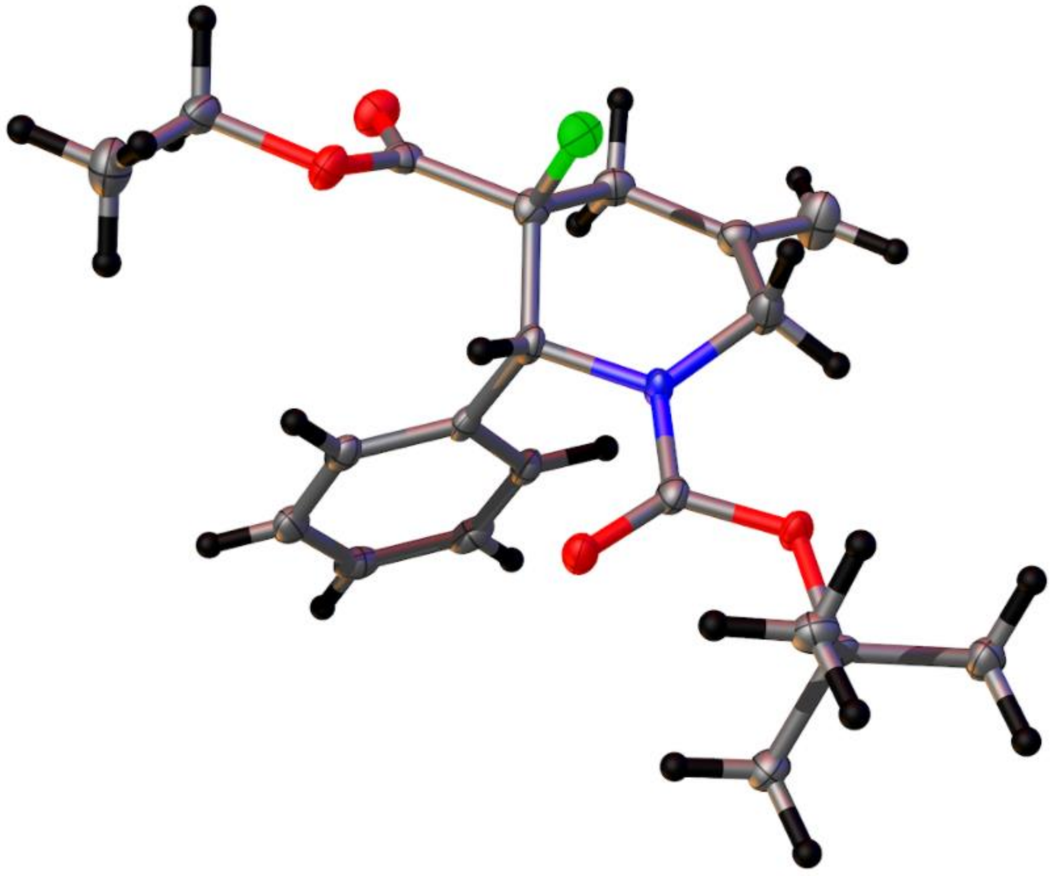

Figure 1. X-ray crystal structure of compound **6**. Displacement ellipsoids are drawn at the 50% probability level.

Table S1. Crystal data and structure refinement for compound **6**.

Empirical formula	$C_{20}H_{26}FNO_4$
Formula weight	363.42
Temperature/K	120
Crystal system	monoclinic
Space group	$P2_1$
a/Å	9.520(8)
b/Å	10.397(10)
c/Å	10.131(9)
α/°	90
β/°	112.109(9)
γ/°	90
Volume/Å^3	929.0(14)
Z	2
ρ_{calc}g/cm^3	1.299
μ/mm^{-1}	0.096
F(000)	388.0
Crystal size/mm^3	0.385 × 0.228 × 0.22
Radiation	MoKα (λ = 0.71073)
2Θ range for data collection/°	4.34 to 55.02
Index ranges	-12 ≤ h ≤ 12, -12 ≤ k ≤ 13, -12 ≤ l ≤ 13
Reflections collected	10682
Independent reflections	3970 [R_{int} = 0.0378, R_{sigma} = 0.0707]
Data/restraints/parameters	3970/202/239
Goodness-of-fit on F^2	1.053
Final R indexes [I>=2σ (I)]	R_1 = 0.0515, wR_2 = 0.0798
Final R indexes [all data]	R_1 = 0.0806, wR_2 = 0.0881
Largest diff. peak/hole / e Å^{-3}	0.27/-0.25
Flack parameter	0.8(5)

__X-ray crystallographic analysis for compound 13a__

Deposition number CCDC 2063487

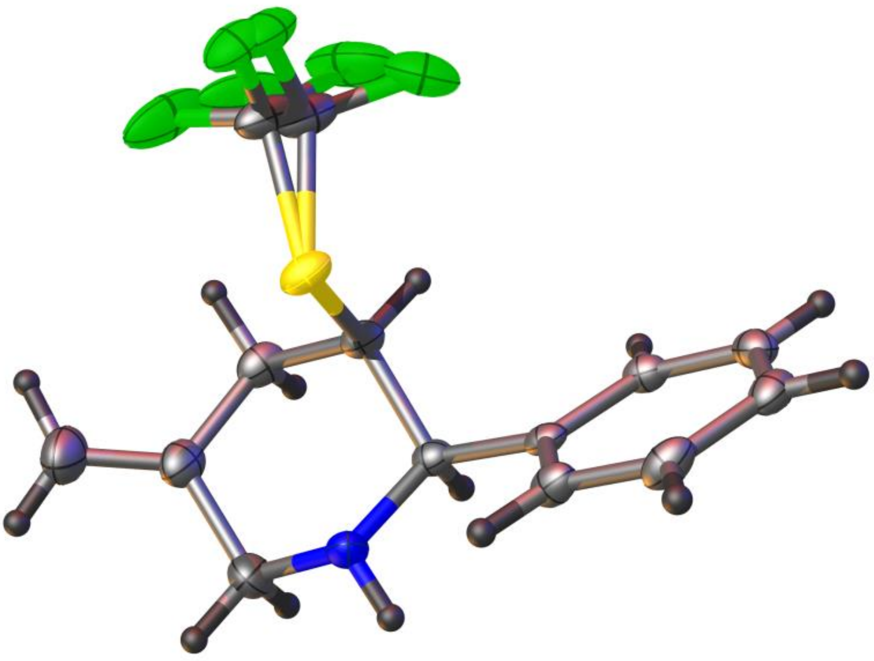

Figure 2. X-ray crystal structure of compound **13a**. Displacement ellipsoids are drawn at the 50% probability level.

Table S2. Crystal data and structure refinement for compound **13a**.

Empirical formula	$C_{13}H_{14}F_3NS$
Formula weight	273.31
Temperature/K	100.0
Crystal system	monoclinic
Space group	$P2_1/n$
a/Å	14.7081(9)
b/Å	5.3695(3)
c/Å	18.0831(11)
α/°	90
β/°	113.266(2)
γ/°	90
Volume/Å^3	1311.98(14)
Z	4
ρ_{calc}g/cm^3	1.384
μ/mm^{-1}	2.383
F(000)	568.0
Crystal size/mm^3	0.33 × 0.172 × 0.076
Radiation	CuKα (λ = 1.54178)
2Θ range for data collection/°	6.604 to 133.054
Index ranges	-17 ≤ h ≤ 17, -6 ≤ k ≤ 6, -21 ≤ l ≤ 20
Reflections collected	17305
Independent reflections	2317 [R_{int} = 0.0375, R_{sigma} = 0.0238]
Data/restraints/parameters	2317/182/198
Goodness-of-fit on F^2	1.115
Final R indexes [I>=2σ (I)]	R_1 = 0.0343, wR_2 = 0.0845
Final R indexes [all data]	R_1 = 0.0360, wR_2 = 0.0855
Largest diff. peak/hole / e Å^{-3}	0.32/-0.21

X-ray crystallographic analysis for compound 13e

Deposition number CCDC 2063489

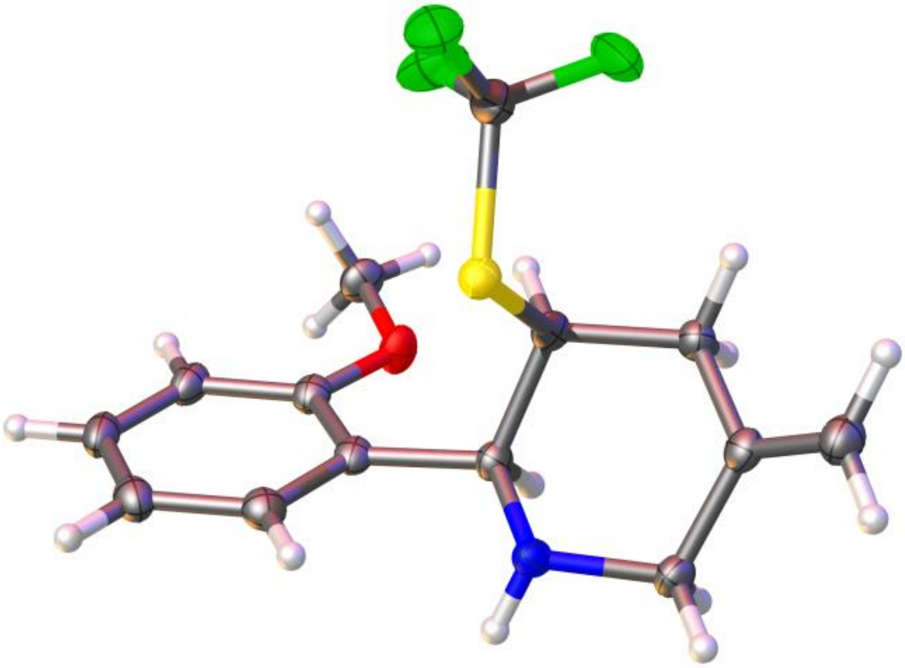

Figure 3. X-ray crystal structure of compound **13e**. Displacement ellipsoids are drawn at the 50% probability level.

Table S3. Crystal data and structure refinement for **13e**.

Empirical formula	$C_{14}H_{16}F_3NOS$
Formula weight	303.34
Temperature/K	100.0
Crystal system	monoclinic
Space group	C2/c
a/Å	27.4272(12)
b/Å	10.8556(5)
c/Å	10.1597(4)
α/°	90
β/°	107.448(2)
γ/°	90
Volume/Å^3	2885.8(2)
Z	8
ρ_{calc}g/cm^3	1.396
μ/mm^{-1}	2.276
F(000)	1264.0
Crystal size/mm^3	0.31 × 0.256 × 0.22
Radiation	CuKα (λ = 1.54178)
2Θ range for data collection/°	6.756 to 133.228
Index ranges	-32 ≤ h ≤ 32, -12 ≤ k ≤ 12, -12 ≤ l ≤ 11
Reflections collected	24510
Independent reflections	2550 [R_{int} = 0.0476, R_{sigma} = 0.0195]
Data/restraints/parameters	2550/0/186
Goodness-of-fit on F^2	1.034
Final R indexes [I>=2σ (I)]	R_1 = 0.0289, wR_2 = 0.0705
Final R indexes [all data]	R_1 = 0.0320, wR_2 = 0.0726
Largest diff. peak/hole / e Å^{-3}	0.35/-0.23

References

1. Jiang, M.; Zhu, F.; Xiang, H.; Xu, X.; Deng, L.; Yang, C., *Org. Biomol. Chem.* **2015**, *13*, 6935-6939.
2. Allen, B. D. W.; Connolly, M. J.; Harrity, J. P. A., *Chem. Eur. J.* **2016**, 22, 13000-13003.
3. Stewart, I. C.; Douglas, C. J.; Grubbs, R. H., *Org. Lett.* **2008**, 10, 441-444.
4. Linderman, R. J.; Graves, D. M., *J. Org. Chem.* **1989**, 54, 661-668.
5. Baumann, M.; Baxendale, I. R.; Martin, L. J.; Ley, S. V, *Tetrahedron* **2009**, 65, 6611-6625.
6. Kim D. Y.; Choi J. S.; Rhie D. Y., *Synth. Commun.* **1997**, *27*, 1097-1103.
7. Kim, D.Y.; Rhie, D.Y.; Oh, D.Y., *Tetrahedron Lett.* **1996**, 37, 653-654.
8. Zeng X.; Lu Z.; Liu S.; Hammond G. B., *Adv. Synth. Catal.* **2017**, *359*, 4062–4066
9. Pasceri, R.; Bartrum, H. E.; Hayes, C. J.; Moody, C. J, *Chem. Commun.* **2012**, 48, 12077-12079.
10. Surmont, R.; Verniest, G.; Kimpe, N., *Org. Lett.*, **2010**, *10*, 4648-4651.
11. Yadav, V. K.; Srivastava, V. P.; Yadav, L. D. S., *Tetrahedron Lett.* **2016**, 57, 2236-2238.
12. Urban, M.; Franc, M.; Hofmanová, M.; Cisařová, I.; Veselý, J., *Org. Biomol. Chem.* **2017**, 15, 9071-9076.
13. Diehl, J.; Brückner, R., *Eur. J. Org. Chem.* **2017**, 278-286.
14. Frantz, R.; Hintermann, L.; Perseghini, M.; Broggini, D.; A. Togni, A., *Org. Lett.* **2003**, *5*, 1709-1712.
15. Ibad, M. F.; Abid, O.; Adeel, M.; Nawaz, M.; Wolf, V.; Villinger, A.; Langer, P., *J. Org. Chem.* **2010,** *75*, 8315-8318.
16. Davis, F. A.; Han, W.; Murphy, C. K., *J. Org. Chem*., **1995**, *60*, 4730-4737.
17. Lankin, D. C.; Chandrakumar, N. S.; Rao, S. N.; Spangler, D. P.; Snyder, J. P., *J. Am. Chem. Soc.* **1993**, *115*, 3356–3357.
18. Snyder, J. P.; Chandrakumar, N. S.; Sato, H.; Lankin, D. C., *J. Am. Chem. Soc.* **2000**, *122*, 544–545.
19. Sun, A.; Lankin, D. C.; Hardcastle, K.; Snyder, J. P., *Chem. – A Eur. J.* **2005**, *11*, 1579–1591.
20. Thibaudeau, C.; Plavec, J.; Chattopadhyaya, J., *J. Org. Chem.* **1998**, *63* , 4967–4984.
21. Alvernhe, G.; Laurent, A.; Touhami, K.; Bartnik, R.; Mloston, G., *J. Fluor. Chem.* **1985**, *29*, 363–384.
22. Bruker, **2016**, SADABS. Bruker Axs Inc., Madison, Wisconsin, USA.
23. Krause, L.; Herbst-Irmer, R.; Sheldrick, G. M.; Stalke, D., *J. Appl. Cryst.* **2015**, 48, 3–10.
24. Sheldrick, G. M., *Acta Cryst*. **2015**, A71, 3-8.
25. Sheldrick, G. M., *Acta Cryst*. **2015**, C71, 3-8.
26. Dolomanov, O.V.; Bourhis, L.J.; Gildea, R.J.; Howard, J.A.K.; Puschmann, H., *J. Appl. Cryst*. **2009**, 42, 339-341
